# Comprehensive analysis of circular RNA profiles in skeletal muscles of aging mice and after aerobic exercise intervention

**DOI:** 10.18632/aging.102932

**Published:** 2020-03-17

**Authors:** Mingwei Guo, Jin Qiu, Fei Shen, Sainan Wang, Jian Yu, Hui Zuo, Jing Yao, Sainan Xu, Tianhui Hu, Dongmei Wang, Yu Zhao, Yepeng Hu, Feixia Shen, Xinran Ma, Jian Lu, Xuejiang Gu, Lingyan Xu

**Affiliations:** 1 Department of Endocrine and Metabolic Diseases, The First Affiliated Hospital of Wenzhou Medical University, Wenzhou, Zhejiang, China; 2 Shanghai Key Laboratory of Regulatory Biology, Institute of Biomedical Sciences and School of Life Sciences, East China Normal University, Shanghai, China; 3 Key Laboratory of Adolescent Health Assessment and Exercise Intervention, Ministry of Education, East China Normal University, Shanghai, China

**Keywords:** circular RNA, aging, aerobic exercise, biomarker, network

## Abstract

Aging induces gradual accumulation of damages in cells and tissues, which leads to physiological dysfunctions. Aging-associated muscle dysfunction is commonly seen in aged population and severely affects their physical activity and life quality, against which aerobic training has been shown to exert antagonizing or alleviating effects. Circular RNAs (circRNAs) play important roles in various physiological processes, yet their involvement in aging-associated muscle dysfunction is not well understood. In this study, we performed comprehensive analysis of circRNAs profiles in quadriceps muscles in sedentary young and aging mice, as well as aging mice with aerobic exercise using RNA sequencing. Our results identified circRNAs altered by factors of aging and aerobic exercise. Their host genes were then predicted and analyzed by gene ontology enrichment analysis. Importantly, we found that circBBS9 featured decreased levels in aging compared to young mice and elevated expression in exercise versus sedentary aging mice. Besides, we performed GO and KEGG analysis on circBBS9 target genes, as well as established the circBBS9-miRNA-mRNAs interaction network. Our results indicate that circBBS9 may play active roles in muscle aging by mediating the benefits of aerobic training intervention, thus may serve as a novel therapeutic target combating aging-associated muscle dysfunction.

## INTRODUCTION

An increase in life expectancy in modern world brings an ‘aged society’, in which a substantial aging population poses challenges both medically and financially [1, 2]. Thus, more and more research interests are piqued toward the combat against aging. Generally, aging is defined as the age-dependent physiological decline that affects all living organisms. Aging undermines multiple major organs and plays a profound role in the onset of neurodegenerative diseases, cardiovascular diseases, metabolic disorders, as well as a loss in muscle and bone mass [3–7].

Recently, metabolic fitness emerges as a novel player in the arena of combating aging. For instance, the prevalence of calorie-enriched diets and sedentary lifestyle of today’s society has been shown to be closely linked with the onset and deteriorating of various age-associated diseases [8, 9]. Evidences from genetic modified mice models revealed that alterations in genes controlling metabolic homeostasis have significant impacts on overall longevity of the animals [10, 11]. Last but not least, metabolic interventions, such as caloric restriction, intermittent fasting and exercise, confer multiple health benefits ranging from stronger skeleton muscle and cardiac function to improved metabolic fitness and cognitive functions, and most importantly, increased lifespan in multiple species [12, 13].

Skeletal muscle comprises about 40% of total body mass in mammals and consumes a large proportion of fuel molecules in both resting and active states. These characteristics attribute a central role to skeletal muscle in maintaining whole body physical fitness and metabolic homeostasis [14–18]. During aging, skeletal muscle undergoes aging-associated muscle dysfunction, which contributes greatly to the disrupted metabolic homeostasis. Multiple factors affect the process of aging-associated muscle dysfunction. Nowadays, the rapid development and wide application of RNA sequencing technology have enabled an extensive understanding of the role of non-coding RNAs in muscle dysfunctions. For instance, a number of micro RNAs are reported to show significant influence on the physiology and pathology of muscle fibers [19–21]. Circular RNA (circRNA), a subfamily of the non-coding RNAs, is ubiquitously expressed in eukaryotes with tissue- and developmental-stage-specific characteristics, which feature a covalently closed loop in its structure. Compared to miRNA, circRNAs are exceptionally stable due to their circular structure that lacking free 5’or 3’ ends, thus endows them superior potential as signaling molecules and biomarkers [22]. Indeed, circRNAs exhibit critical functions under both physiological and pathological scenarios through diverse regulations on gene transcription, pre-mRNA splicing, mRNA translation and protein functions, as well as their classic roles as miRNA sponges [23]. Overall, circRNAs are attractive candidates as biomarkers and therapeutic targets of aging-associated muscle dysfunction [24].

The aim of the study was to find potential circRNA involved in muscle aging and exercise via profiling the expression signature and heterogeneity pattern of circRNAs in quadriceps femoris muscles of sedentary young and aging mice, as well as aging mice with aerobic training. Our results identified circBBS9 and its target gene pathways affected by the advance of age, as well as reversed by aerobic exercise, which may offer novel insights into the biomarkers and the pathogenesis of aging-associated muscle dysfunction.

## RESULTS

### Description of circRNA profiles in quadriceps femoris muscle from young, aging and aging with aerobic exercise group of mice

**Figure 1:**
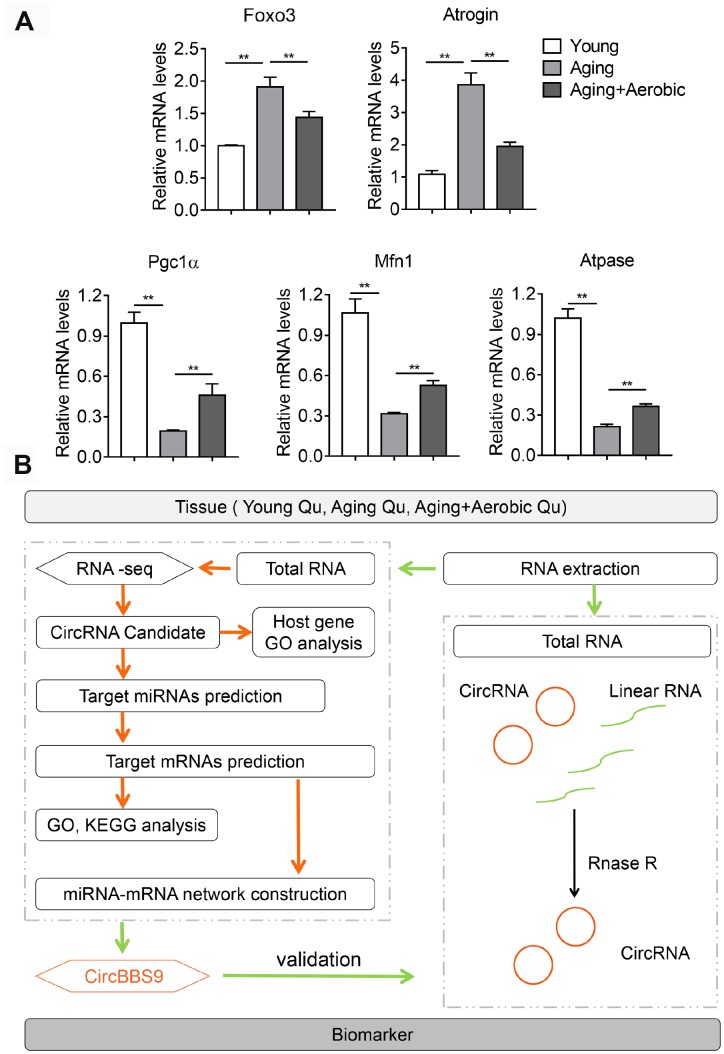
**Validation of aging and aerobic training model in mice and workflow of circRNA analysis scheme.** (**A**) The Qu muscle expression level of genes in atrophy and mitochondrial functionality among groups of young, aging and aging with aerobic exercise (n=6 per group). (**B**) Workflow of circRNA analysis scheme.

**Figure 2:**
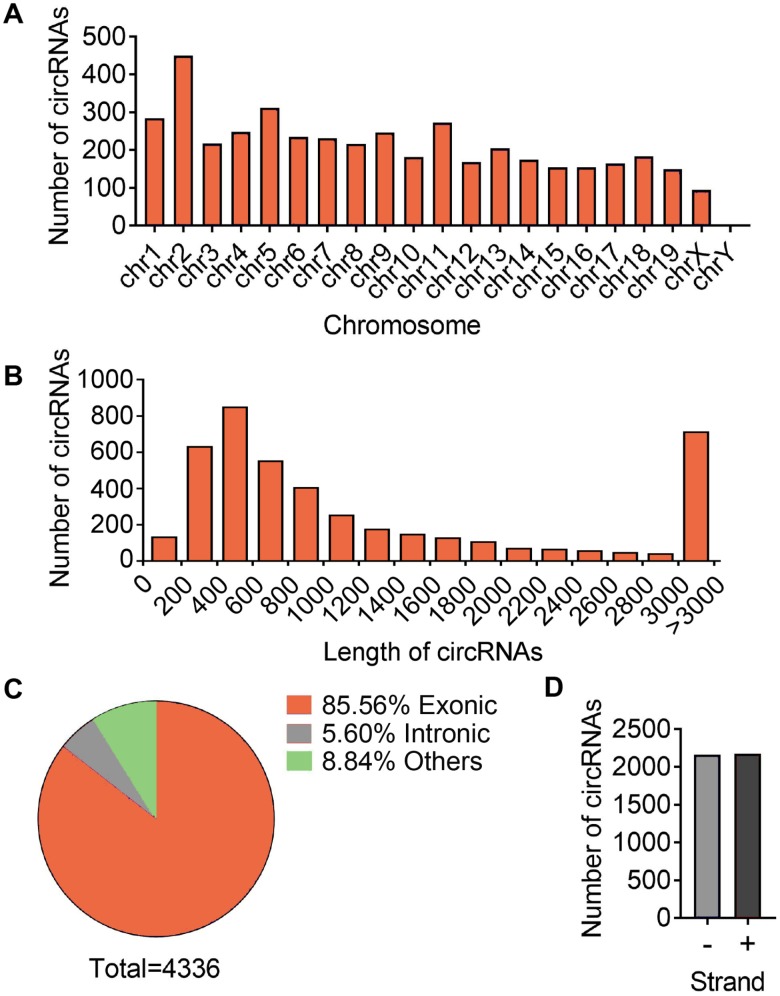
**Differences and characterizations of circRNA expression profile.** (**A**) Chromosomal distributions of annotated circRNAs. (**B**) Predicted spliced length of circRNAs. (**C**) The circRNA were classified into three types according to the relationship of the genomic loci with their associated coding genes. (**D**) Distribution of circRNA in sense (+) and antisense (-) strand of DNA.

To access the extents of muscle dysfunction, six muscle samples per group from mice of young, aging and aging plus aerobic exercise were examined for gene markers indicative of mitochondrial homeostasis and muscle atrophy. Compared to young control mice, we found significant mitigation in mitochondrial program (Pgc1a, Mfn1 and Atpase) and elevation of atrophy markers (Foxo3 and Atrogin) in aging mice, while aging mice with aerobic training showed marked amelioration in these parameters (Figure 1A) as consistent with previous reports [25]. Next, using RNA-seq technique, we evaluated the circRNA profiles of quadriceps femoris muscles in three groups of samples following the workflow shown in Figure 1B. The sequencing identified 4336 circRNAs in total in three groups (Supplementary Table 1). The numbers of circRNA distributed in genome were the highest in chromosome 2 and reached nadir in chromosome Y, with most chromosomes had a synchronized distribution of about 200 counts (Figure 2A). The majority of the identified circRNAs were less than 1500 nucleotide (nt) in length (Figure 2B). The relationship between circRNAs and their coding genes were summarized and classified into three categories: 85.56% were exonic, 5.60% were intronic and 8.84% were others types (Figure 2C). Besides, all circRNAs were evenly located on DNA plus and minus strand (Figure 2D).

### Identification of differential circRNAs in quadriceps muscles

**Figure 3:**
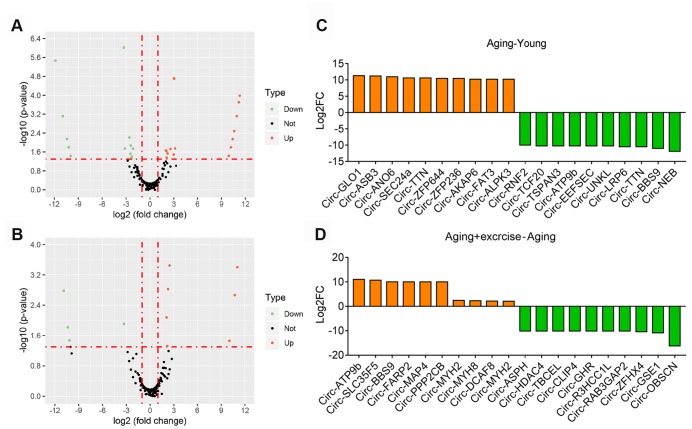
**Overview of altered circRNA in different sequencing group.** (**A**) Volcano plots showing differential expression of circRNAs of Aging group compared with Young group. Differentially expressed circRNAs with fold change > 2 and p < 0.05 were marked in orange and green dots representing up and down regulation separately. (**B**) Volcano plots showing differential expression of circRNAs of Aging plus Exercising compared to Aging group. Differentially expressed circRNAs with fold change > 2 and p < 0.05 were marked in orange and green dots representing up and down regulation separately. (**C**) The top 10 upregulated and 10 downregulated circRNA based on the log2 fold change of Aging group compared with Young group. (**D**) The top 10 upregulated and 10 downregulated circRNA based on the log2 fold change of Aging plus Exercising group compared to Aging group.

We firstly investigated the differential circRNAs in quadriceps muscle between young and aging group, which may be used as biomarkers for aging-associated muscle dysfunction. The volcano plot filtered and identified the differentially expressed circRNAs with statistical significance (Figure 3A). The threshold of exhibiting fold change is 2.0 and p-values below 0.05. Between the young and aging group, 49 circRNAs showed significant differential expression, with 28 circRNAs up-regulated and 21 circRNAs down-regulated (Supplementary Table 2). Meanwhile, comparison between aging mice and aging mice with aerobic treadmill training identified 21 significantly changed circRNAs, among them 10 were up-regulated and 11 were down-regulated (Figure 3B, Supplementary Table 3). The top changed circRNAs were shown in Figure 3C, 3D.

To explore the potential functional roles of the significantly enriched circRNAs and their host genes, we performed Gene Ontology (GO) enrichment analysis on the host genes. GO analysis is divided into three parts as biological process (BP), cell component (CC) and molecular function (MF). We found that many biological processes (BP), i.e. muscle contraction and skeletal muscle thin filament assembly, as well as cell component (CC) including myofibril, muscle myosin complex and myosin filament were altered in aging mice compared to young mice. (Supplementary Figure 1). On the other hand, compared to sedentary aging mice, aging mice with aerobic intervention showed altered biological processes (BP) including muscle filament sliding, neuron remodeling, muscle contraction and cell components (CC) including myofibril, myosin complex, cytoskeleton and muscle myosin complex (Supplementary Figure 2). These data suggested a potential cooperation between circRNAs and their host genes in the regulation of muscle functions during aging and aerobic interventions.

### Validation of circBBS9 expression in skeletal muscle

Based on the sequencing data, we wonder whether we could define circRNA as potential target molecule that mediates the benefits of aerobic training during aging process by highlighting candidates that featured altered expression in aging while reversed by exercise. Hence, we overlapped circRNAs of three sets of sequencing data, looking for those featured opposite expression patterns between young and aging, as well as between sedentary aging and aging with exercise intervention. Importantly, we found three circRNAs that met our criteria, circBBS9, circATP9b and circMYH8 (Supplementary Table 4).

**Figure 4:**
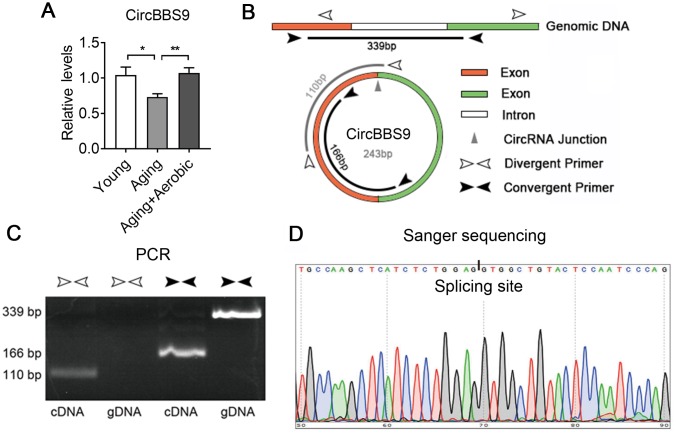
**Verification of the expression of CircBBS9.** (**A**) qRT-PCR verification of the expression of circBBS9 among groups. (**B**) Schematic diagram of primer design of circBBS9. (**C**) Identification of circBBS9 in Qu muscle by PCR amplification. (**D**) Sanger sequencing to verify the amplified products of circBBS9. Data are presented as mean ± SEM and *P<0.05, **P<0.01.

Next, we verified circBBS9, circATP9b and circMYH8 with qRT-PCR. By qRT-PCR using primers across the branch site [26], we found that muscle circBBS9 level was down-regulated in aging versus young sedentary mice and was restored after aerobic training in aging mice, which was the best match for RNA-seq data (Figure 4A), while circMYH8 and circATP9b levels failed to match the sequencing results (Supplementary Figure 3A). Thus we focused on circBBS9 for detailed analysis.

According to UCSC genome browser, circBBS9 was spliced from a 416bp pre-mRNA containing two exons and one intron. CircBBS9 has a length of 243bp derived from the joint of two adjacent exons during spicing (Figure 4B). To ensure assay facticity in circRNA, we used RiboNuclease R for full degradation of preponderant linear RNAs and Y-structure RNAs to preserve the circular RNAs. Next, convergent primers designed specifically against the adjacent ends of two exons (interspaced by the intron) and divergent primers against the far end of two exons were tested in RT-PCR analysis in complementary DNA (cDNA) sample and genomic DNA (gDNA) sample from quadriceps muscles. As shown in Figure 4C, convergent primers yielded PCR products of 339bp in gDNA and 166bp in cDNA sample, indicating the exclude of intron during splicing. Meanwhile, unlike in cDNA sample, divergent primers failed to amplify PCR products in gDNA sample, suggesting circBBS9 is a circular RNA derived from the splicing of pre-mRNA. This was confirmed via Sanger sequencing, which showed the existence of a splicing event on AG-GT site (Figure 4D). Interestingly, circBBS9 levels were not altered in brain and heart, two other critical organs underwent senescence and dysfunction during aging (Supplementary Figure 3B). These data suggest that although aging is a systematic process, circBBS9 might be a reliable and specific biomarker for skeletal muscle sarcopenia and aerobic exercise intervention.

### Construction of circBBS9-miRNA-mRNA Network and validation of circBBS9 predicted target mRNAs

CircRNAs classically function as miRNA sponges to exert their regulatory effects on gene expression [26]. We thus constructed the circBBS9-miRNA-mRNA network to better unravel its role in muscle dysfunction. The Miranda and RNAhybrid bioinformatics tools were utilized to predict the sponge miRNAs for circBBS9, which predicted 10 miRNAs as its targets (mmu-miR-3100-5p, mmu-miR-6930-3p, mmu-miR-7020-5p, mmu-miR-423-3p, mmu-miR-7079-5p, mmu-miR-383-3p, mmu-miR-6911-5p, mmu-miR-3065-3p, mmu-miR-7028-5p, mmu-miR-7662-5p). Next, using database Targetscan, mRNAs predicted as the targets of at least three miRNAs were overlapped and considered as mRNA targets, which rendered 1558 target mRNAs. These genes were analyzed with GO and KEGG analysis to annotate and speculate their potential functions.

**Figure 5:**
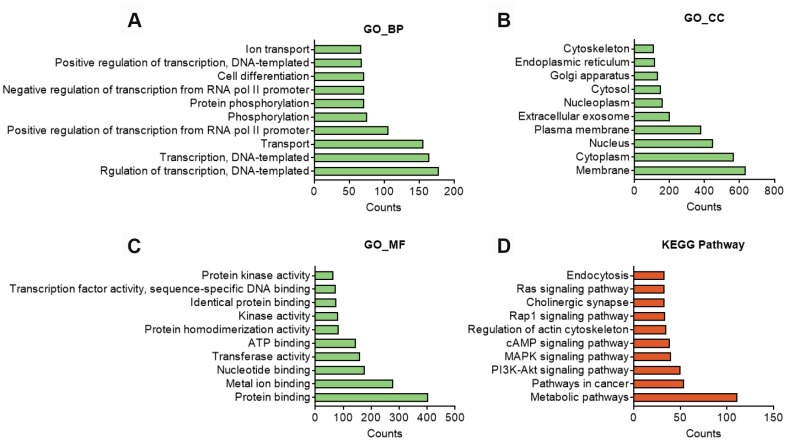
***In silico* analysis of predicted target genes of circBBS9.** (**A–C**) GO analysis of predicted target genes with top 10 differ gene counts. The horizontal axis is the gene counts for the GO terms, and the vertical axis is the GO terms. (**D**) KEGG pathway analysis of predicted target gene with top 10 differ gene counts. Selection counts represent the number of entities of target genes directly associated with the listed Pathway.

**Figure 6:**
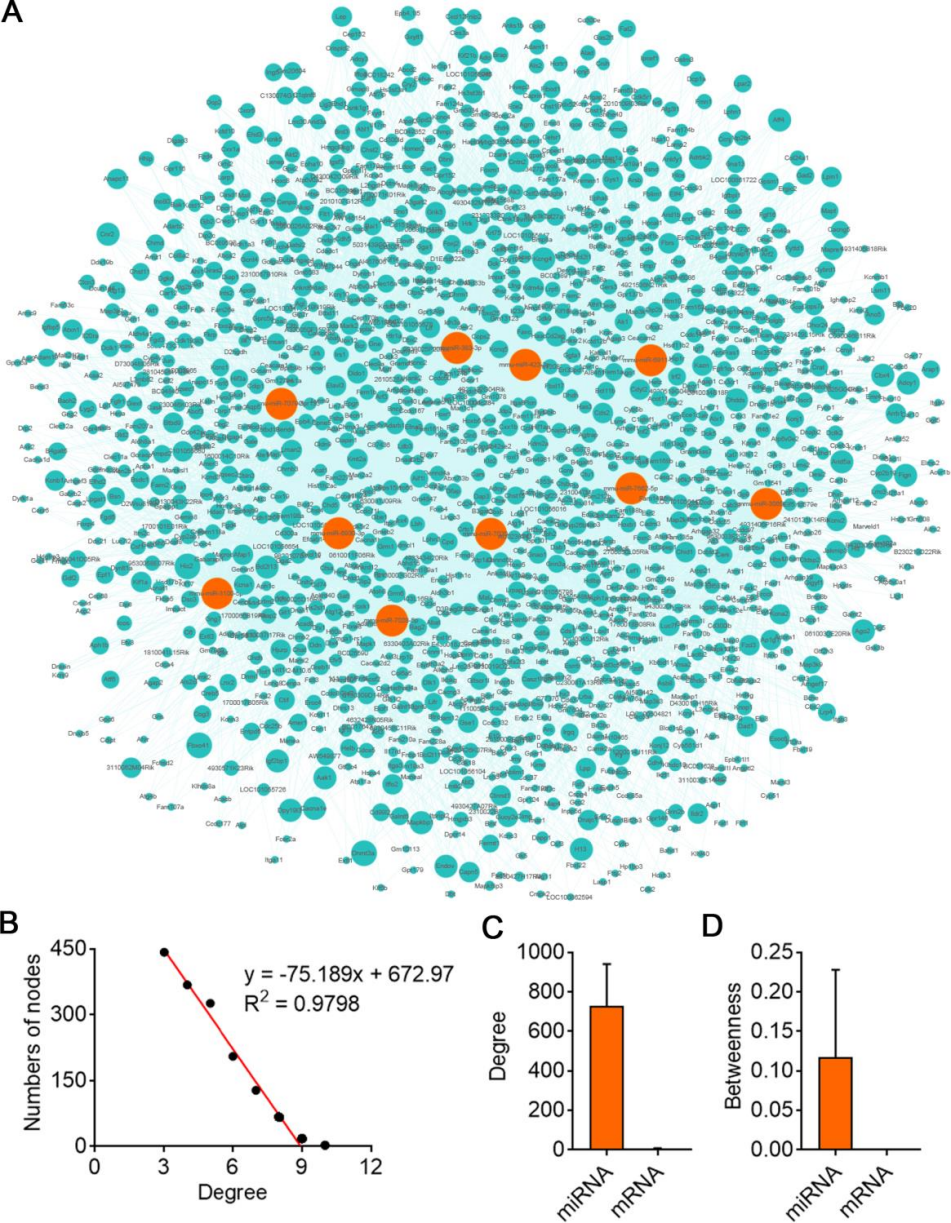
**CircBBS9 target miRNA-mRNA network analysis.** (**A**) Network of the circRNA-miRNA which have greater interaction score with their target mRNAs. The pink nodes represented miRNA and the green nodes represented mRNAs. (**B**) Degree distribution of circBBS9 related miRNA-mRNA network. (**C**, **D**) The degree and betweenness centrality of mRNAs and miRNAs.

GO analysis on the target genes of circBBS9 highlighted various biological processes, for instance, transcription regulation and protein phosphorylation in BP analysis and protein homodimerization and ATP binding in MF analysis (Figure 5A–5C). Subsequent KEGG analysis of target genes emphasized metabolic pathways, PI3K-Akt signaling pathway, MAPK signaling among top 10 enriched pathways (Figure 5D). Of note, metabolic pathways had the highest target gene counts among these signaling pathways, suggesting a critical impact of metabolic changes on muscle functions during aging and exercise intervention. Based on these analyses, the comprehensive atlas of miRNA-mRNA network for circBBS9 was then constructed using Cytoscape software (3.6.1). In detail, we created miRNA-mRNA network if the mRNA were regulated by a greater number (>=3) of miRNAs and had a p-value <0.05. The circBBS9 miRNA-mRNA network was comprised of 7,283 edges between 10 miRNAs and 1,558 mRNAs (Supplementary Table 5, Figure 6A). The degree of distribution of the nodes followed the power-law distribution with a slope of -75.189 and an R-squared value of 0.9798, suggesting the network displayed scale-free characteristics typical of a biological network rather than a random system (Figure 6B). In Figure 6C, compared to mRNAs (4.678), The average degree of the miRNAs was 728.3. Besides, miRNA nodes showed more betweenness centrality compared to mRNA nodes as in Figure 6D. These results suggested that miRNAs have more interactions with other nodes than mRNAs and although miRNAs are small RNAs, they exhibit more topological properties than mRNAs in the network. Interestingly, a few miRNAs acted as major hubs linking other mRNAs, such as mmu-miR-7662-5p, mmu-miR-7028-5p and mmu-miR-6911-5p (degree=940), which were largely beyond the maximum degree node among mRNAs (Hic2, Fbxo41, Dnmt3a, degree=10).

**Figure 7:**
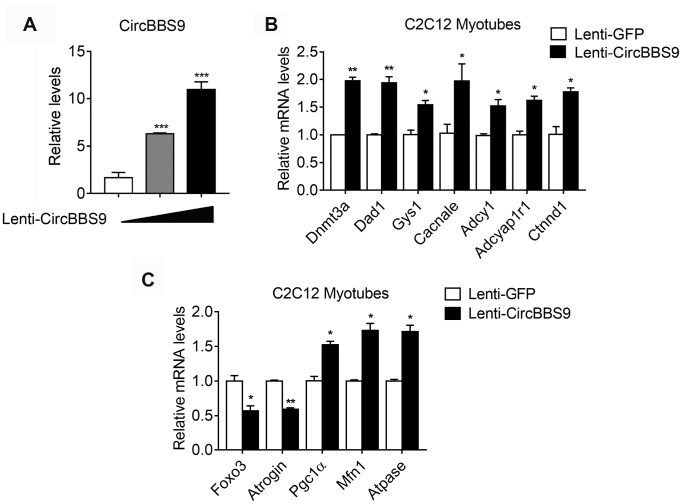
**mRNA alternations upon circBBS9 overexpression in differentiated C2C12 myotubes.** (**A**) Expression levels of circBBS9 in differentiated C2C12 myotubes after infection of lentiviral delivery of circBBS9. (**B**, **C**) Expression levels of predicted circBBS9 target genes (**B**) and general muscle functionality genes (**C**) in differentiated C2C12 myotubes after infection of lentiviral delivery of circBBS9. Data are presented as mean ± SEM and **P<0.01.

In order to confirm the network of circBBS9-miRNA-mRNA, we overexpressed circBBS9 in differentiated C2C12 myotubes and examined a few predicted target mRNAs, including Dnmt3a, Dad1, Gys1, Cacnale, Adcy1, Adcyap1r1 and Ctnnd1, which belong to different top predicted pathways. Importantly, we found that circBBS9 overexpression increased these gene expressions (Figure 7A, 7B), in addition to decreased muscle atrophic genes (Foxo3 and Atrogin) and elevated functional mitochondrial genes (Pgc1α, Mfn1 and Atpase) (Figure 7C). Overall, these data suggested that circBBS9 overexpression improve muscle functionality gene programs and may be involved in muscle aging process.

## DISCUSSION

In this study, we identified and verified circRNAs that showed differential expression between young and old. Analysis on their target genes revealed metabolic pathways among the top affected pathways. We also highlighted circRNAs that featured declined expression in aging compared to young and were reversed by aerobic training. Among them, circBBS9 was verified and its circRNA-miRNA-mRNA regulatory network established. Taken together, our results revealed multiple circRNAs as potential biomarkers of aging and unveiled a previously unappreciated role of circBBS9 in mediating the beneficial effects of aerobic exercise, at lease in aged subjects. Future studies are warranted to study the potential function of circBBS9 as a novel therapeutic target for aging-associated muscle dysfunction.

Aging-associated muscle dysfunction severely affects the health states and life qualities of the elders. As the basic module of skeletal muscle, myocytes, also known as muscle fibers, are packed with mitochondrial to maintain their high ATP demand during exercise for energy sustenance. It is thus vital for myocytes to maintain strict mitochondrial homeostasis for optimal muscle functionality. Notably, muscle mitochondrial homeostasis undergoes dynamic regulation in both. normal and pathological conditions. For example, aged population suffers from aging-associated decrease in muscle mass and strength, with prominent mitochondrial dysfunctions in myocytes. On the other hand, aerobic training is one of the most effective ways in promoting muscle health and mitochondrial functionality by activating key metabolic regulators such as SIRT1, AMPK and PGC1α [17]. However, implementing aerobic exercise in the aged population demands significant caution in frequency and intensity so as to balance its desiring effects with potential damages like bone fractures and cardiovascular events [18]. One alternative in promoting mitochondrial homeostasis and muscle health in aging population would be to use specific activators targeting these regulators, i.e. SIRT1 agonist resveratrol and AMPK activator AICAR, which have shown beneficial effects in promoting muscle function and alleviating aging-associated muscle dysfunction in rodent models [8]. Yet, their clinical applications were hindered by uncertainties in side effects and off-targets in other organs, attesting to the urgency of uncovering novel biomarkers and potential therapeutic targets against aging-associated muscle dysfunction. Previous studies showed that miRNAs and circRNAs played important roles in the myogenic processes and muscle functions, which inspired us to investigate whether circRNAs may mediate the benefits of aerobic exercise on muscle health. Indeed, we found significant alterations in circRNAs expression pattern on the genomic scale in skeletal muscle in aging mice compared to young controls. Interestingly, GO and KEGG analysis indicated that their host genes were related to muscle contraction and development, indicating a potential cooperation between host genes and their circRNAs. Furthermore, KEGG pathway analysis of predicted target genes attributed metabolic pathway as top pathways affected by these circRNAs, unveiling a previously unappreciated role of energy metabolism in aging-associated muscle dysfunction.

Importantly, by comprehensive analysis of circRNAs profiles among young, aging and aging mice with aerobic exercise, we found that compared to young mice, circBBS9 featured decreased levels in aging mice and reversed expression after aerobic exercise and it is annotated in the circBase [22, 27]. Intriguingly, miRNA-mRNA network analysis of circBBS9 annotated top genes in metabolic pathways from KEGG analysis as its potential target genes, which supported a possible regulatory role of circBBS9 in the metabolic programs in myocytes. For example, LDHA, AMD1, DNMT3A and ACADSB were reported to regulate muscle cell proliferation and muscle atrophy [28–31], while ACADS, CS, GATM, CHSY1 were related to muscle energy metabolism [32–34]. Thus, aging and aerobic training, two opposite processes impact muscle functionality, may exert their impacts by modulating the circBBS9-miRNA-mRNA network. Indeed, we have validated the alternations of a few predicted circBBS9 target genes including Dnmt3a, Dad1, Gys1, Cacnale, Adcy1, Adcyap1r1 and Ctnnd1 after circBBS9 overexpression in C2C12 myotubes. Future investigations on circBBS9 in aging muscle would be informative to reveal the exact role and mechanism of its regulation, as well as the potential implication in other models of muscle dysfunction.

Kotb et al. identified and annotated many circRNAs in the skeletal muscles of Rhesus monkey via RNA-seq and verified the downregulation of mmu_circ_017332, mmu_ circ_014269, mmu_circ_015060, mmu_circ_006895, and mmu_circ_014509 during aging in monkeys [35]. In addition, previous reports have identified circFUT10, circLM07, circSVIL, circRBFOXO2, circFGFR2, circZFP609, circFGFR4 and circSNX29 were involved in myogenesis [36–43]. In our study, we highlighted that circBBS9 was a potential biomarker of aging-associated muscle dysfunction and may potentially mediate the amelioration effects exerted by exercise. Of special relevance, our analysis was performed in different animal models than previous studies, and emphasized on unique changes in circRNAs in the reversal of muscle function loss with aerobic exercise, thus may result in different results.

It has to be noticed that the present study was majorly based on bioinformatic analysis of muscle samples from young and aging mice, as well as aging mice with aerobic training. Although circBBS9 was overexpressed in differentiated C2C12 myotubes and several predicted target genes, as well as genes related to mitochondrial and dystrophic functions were confirmed to be altered, suggesting the involvements of circBBS9 in muscle dysfunctions, the detailed molecular evidences of direct binding of circBBS9 with its predicted target miRNAs are warranted and *in vivo* application of circBBS9 in the prevention and treatment of muscle dysfunctions would be attempted in future studies. Besides, it would be interesting to examine the expression levels of circBBS9 in the circulation and its potential changes in serum/plasma under the circumstances of muscle aging and after aerobic training, which would offer circBBS9 as a novel biomarker for muscle aging.

In summary, the present study explored changes in circRNA profiles in skeletal muscle in aging and identified circBBS9 as a possible biomarker of aging-associated muscle dysfunction and potential target of aerobic exercise, which may provide new insights to the mechanism of muscle aging, and promote individualized prevention and treatment for muscle dysfunction.

## MATERIALS AND METHODS

### Animal information and exercise training protocol

C57BL/6J mice at the age of 3-month (n=6) and 18-month (n=12, sedentary or exercise) were defined as young and aging groups, which were acquired from Shanghai Laboratory Animal Company (SLAC, Shanghai, China) and housed in pathogen-free cages at ECNU Animal Center in Shanghai. Mice were located in environment temperature at 22°C and on a 12 hours light/12 hours dark cycles with free access to water and chow diet and all of the procedures were agreed by Animal Care and Use Committee of East China Normal University (M20170316, Shanghai, China).

For exercise training, aging mice were trained with or without treadmill exercise for two months as previously reported [44] (n=6 per group). Briefly, the treadmill running assay was performed on a motorized and speed-controlled treadmill system (ZH-PT, Hangzhou, China). Mice were warmed up at a speed of 8 m/min for 5 min and then the speed was increased by steps of 2 m/min each 2 minutes until 14 m/min without inclination and sustained for 30 minutes. Mice were under aerobic training every other day for two months. The skeletal muscle tissues quadriceps femoris (Qu) were harvested from young mice, aging mice and aging mice with exercise and then frozen in liquid nitrogen and stored at -80 degrees for further studies.

### Library preparation and illumina sequencing

Total RNAs were extracted from mice frozen quadriceps femoris tissues using RNAiso Plus (Takara, Japan) as products descriptions. The total RNA purity and quantity were assayed with Nano Drop 2000(USA). The RNA integrity number (RIN) ≥7 and library was constructed as manufacture’s protocols There were five microgram total RNA were used to eliminate ribosome RNA according to the description of Ribo-Zero rRNA Removal Kit (Illumina, USA). The residual RNAs were dealt with Ribonuclease R, E. coli (Epicenter, USA) to remove liner RNA and enriching circRNA, which were fragmented into oddments with RNA fragment Kit (Ambion, Austin, TX, USA). The oddments were reversely transcribed to produce cDNA. A-bases were added into ends of cDNA strand for connecting the index adaptors linked T-bases. The cDNA libraries were then stablished by PCR and pair-end sequenced (PE150) on an Illumina Hiseq 4000 machine (Genergy Bio company, Shanghai, China) according to vender’s specifications.

### RNA-seq analysis and identification of CircRNAs

Cutadapt (https://cutadapt.readthedocs.io/en/stable/) was used to remove adapter sequences, primers, poly-A tails and other unwanted sequence from high-throughput sequencing reads. FastQC (http://www.bioinformatics.babraham.ac.uk/projects/fastqc/) was used to evaluate sequence quality. circRNA analysis were exerted as described previously [45]. Bowtie2/TopHat2, TopHat-Fusion and CIRCExplorer2 softwares were used to map reads from mouse genome (Ensemble). Differential expression analysis was performed with EB-seq R package. EB-seq R package provides posterior probabilities which are adjusted for multiplicities using Benjamini-Hochberg method [46].

### Gene Ontology (GO) term and KEGG pathway analysis

Gene Ontology (GO: http://www.geneontology.org/) is used to perform functional studies on gene sets [47]. Additionally, the Kyoto Encyclopedia of Genes and Genomes (KEGG: https://www.kegg.jp/) database is used to understand the high-level functions and utilities of the biological system [48]. The Database for Annotation, Visualization and Integrated Discovery (DAVID, https://david.ncifcrf.gov/) is a comprehensive set of functional annotation tools for researchers to understand biological meaning behind large scale of genes [49, 50]. For analysis of cirRNA host genes and target mRNAs, GO enrichment and KEGG pathway analysis were performed using DAVID. P < 0.05 was set as the cut-off criterion.

### Validation of circRNA

Total RNA was digested with RNAse R (epicenter, USA) and then reversely transcribed using random hexamers and reverse transcriptase (Takara, Japan). The quantitative PCR (qPCR) analysis was performed using divergent primers and SYBR Green (ANZY Biotech, China) on a Roche LightCycler 480 II machine. GAPDH were used to internal control for circular RNA qPCR. Besides, conventional PCR was performed using divergent and convergent primers with the templates of cDNA and gDNA respectively and PCR products were size-separated by electrophoresis with 2% of agarose gels and pictured on an UV instrument (Tiangen, China). The primers were listed in Supplementary Table 6.

### Identification of sponge miRNA and miRNA-target interactions

The Miranda and RNAhybrid bioinformatics tools were utilized to predict the sponge miRNAs for circBBS9 with a maximum binding free energy of -25 kcal/mol for the sponge miRNA interaction. Next, using database Targetscan, mRNAs predicted as the targets of at least three miRNAs were overlapped and considered as mRNA targets.

### Network visualization and topological analysis

We used Cytoscape software (version 3.6.1) [51] to construct and visualize the network in this study. Several topological properties such as the node degree, betweenness were analyzed using the built-in Network Analyzer tool (Department of Computational Biology and Applied Algorithmics at the Max Planck Institute for Informatics, Saarbrücken, Germany). The degree of a node is the number of edges that link to this node. Betweenness is a measure of the centrality of the node in a network, which is the number of shortest paths from each node to all others that pass through the node. The power law distribution appears as a straight line with a slope of a power exponent, which is the basis for judging the whether a random variable satisfies a power law and the power-law distribution of node degree is the main parameter used to evaluate the network topology.

### Cell culture

C2C12 cells and HEK293T cells were obtained from ATCC and were cultured in DMEM containing 10% FBS, 100U/ml penicillin and 100ug/ml streptomycin. All cells were incubated at 37°C and 5% CO2 with humid atmosphere. For C2C12 cell differentiation into myotubes, culture plates were pre-incubated with 0.1% gelatin and when C2C12 cells were grown to 70% confluence, differentiated media (2% horse serum) was added and refresh every day for 5 days.

### Plasmids construction and lentivirus infection

circBBS9 was inserted into a pLo-ciR vector (a generous gift from Dr. Junjie Xiao’s lab, Shanghai University). pLo-CirBBS9, psPAX2 and pMD2.G plasmids were co-transfected into HEK293T cells to generate lentiviral particles. Infectious lentivirus was harvested at 36 and 60 hours after transfection and filtered with 0.45 um filters. Differential C2C12 myotubes were infected with circBBS9 lentivirus or control lentivirus (1*10^9^PFU) in the presence of polybrene for 48 hours. To examine circBBS9 and mRNA levels, 18S was used to internal control gene and 2^-ΔΔCt^ methods was applied to calculated relative gene level. The primers were listed in Supplementary Table 6.

### Statistical analysis

Data were analyzed and Graphics with GraphPad prism 7 version (GraphPad Software, Inc, La Jolla, CA) and R 3.3.2 version (CRNA). Data were expressed as the Mean ± SEM. Student’s t-test was used to calculate differences between two groups. p value less than 0.05 was considered as significant difference.

## Supplementary Material

Supplementary Figures

Supplementary Table 1

Supplementary Tables 2-4, 6

Supplementary Table 5
