# Supplementary material for: Comprehensive analysis of circular RNA profiles in skeletal muscles of aging mice and after aerobic exercise intervention
Source: Aging (Albany NY). 2020 Mar 17;12(6):5071–90. doi: 10.18632/aging.102932 (PMC7138574; doi:10.18632/aging.102932)
Supplement: Supplementary Figures [file aging-12-102932-s004..pdf]

SUPPLEMENTARY FIGURES

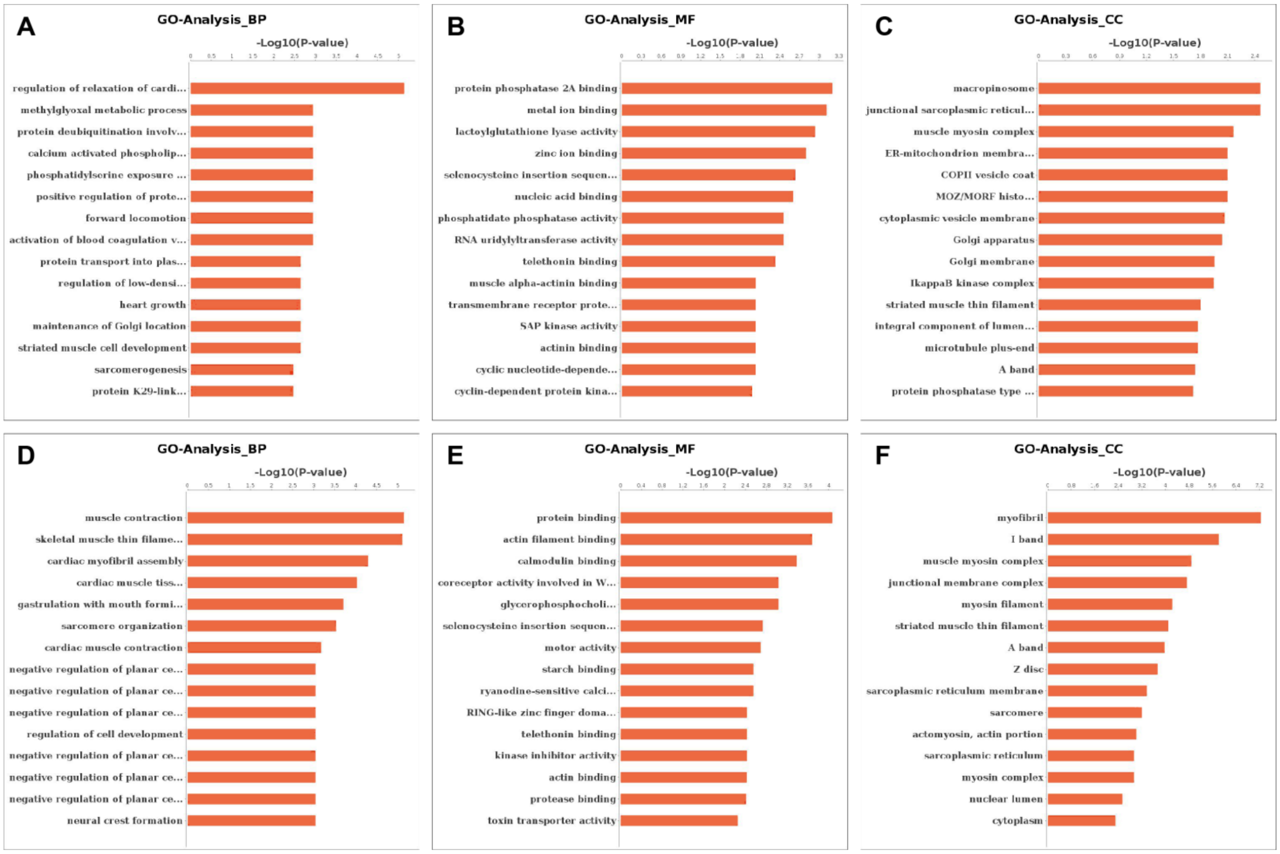

Supplementary Figure 1. GO analysis of host genes of upregulated circRNAs with top 10 enrichment score. GO analysis between aging and young group. (**A**–**C**) or Exercising and Aging group (**D**–**F**). The horizontal axis is the enrichment score for the GO terms, and the vertical axis is the GO terms. The enrichment score was calculated as -log10 (P-value).

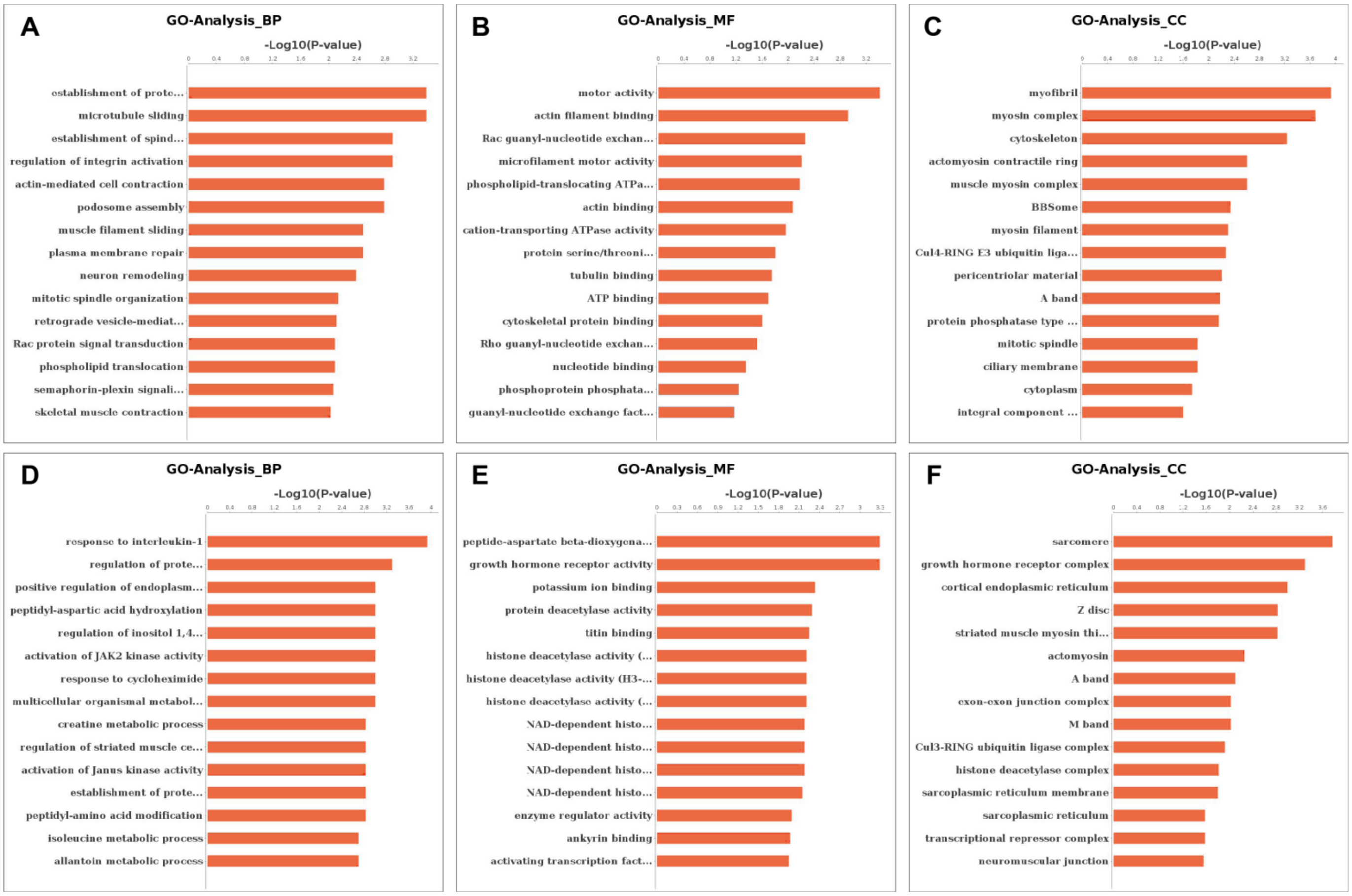

Supplementary Figure 2. GO analysis of host genes of down-regulated circRNAs with top 10 enrichment score. GO analysis between aging and young group. (**A**–**C**) or Exercising and Aging group (**D**–**F**). The horizontal axis is the enrichment score for the GO terms, and the vertical axis is the GO terms. The enrichment score was calculated as -log10 (P-value).

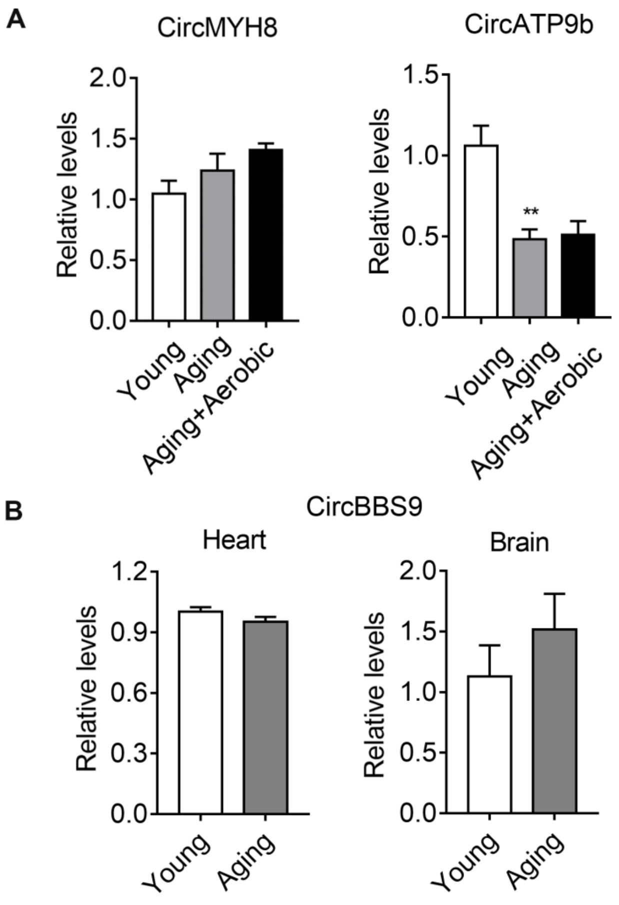

Supplementary Figure 3. Expression levels of circMYH8 and circATP9b in Qu muscle, as well as circBBS9 levels in heart and brain. (**A**) Expression levels of circMYH8 and circATP9b in Qu muscle among groups of young, aging and aging with aerobic exercise (n=6 per group). (**B**) Expression levels of circBBS9 in heart and brain of young and aging mice (n=6 per group). Data are presented as mean ± SEM and **P<0.01.
