# Supplementary material for: Comprehensive analysis of circular RNA profiles in skeletal muscles of aging mice and after aerobic exercise intervention
Source: Aging (Albany NY). 2020 Mar 17;12(6):5071–90. doi: 10.18632/aging.102932 (PMC7138574; doi:10.18632/aging.102932)
Supplement: Supplementary Table 1 [file aging-12-102932-s003..docx]

| **Supplementary Table 1. CircRNA profiles in Qu muscle of young, aging and aging with aerobic exercise.** | | | | | | | |
| --- | --- | --- | --- | --- | --- | --- | --- |
| **ID** | **Chromosome** | **GeneName** | **Young** | **Aging** | **Aging+Exercise** | **Genomic Length** | **Seq Length** |
| chr1_118419918_118419440_+478-Clasp1 | chr1 | Clasp1 | 0 | 0 | 0.180646022 | 479 | 479 |
| chr1_125579327_125568547_+10780-Slc35f5 | chr1 | Slc35f5 | 0.608389058 | 0 | 1.445168179 | 10781 | 571 |
| chr1_127799553_127791605_+7948-Ccnt2 | chr1 | Ccnt2 | 0 | 0.546927538 | 0.180646022 | 7949 | 463 |
| chr1_128223641_128193460_+30181-R3hdm1 | chr1 | R3hdm1 | 0.202796353 | 0.182309179 | 0 | 30182 | 1005 |
| chr1_133753749_133753089_-660-Atp2b4 | chr1 | Atp2b4 | 0.405592706 | 0 | 0 | 661 | 661 |
| chr1_139062971_139040000_+22971-Dennd1b | chr1 | Dennd1b | 1.41957447 | 0.546927538 | 1.445168179 | 22972 | 546 |
| chr1_139062971_139053346_+9625-Dennd1b | chr1 | Dennd1b | 0.811185411 | 0.729236718 | 0.361292045 | 9626 | 376 |
| chr1_151478625_151476140_-2485-Rnf2 | chr1 | Rnf2 | 1.013981764 | 0 | 0.180646022 | 2486 | 466 |
| chr1_155197435_155173986_+23449-Stx6 | chr1 | Stx6 | 0.405592706 | 0.729236718 | 0 | 23450 | 656 |
| chr1_157299264_157286731_-12533-Rasal2 | chr1 | Rasal2 | 0 | 0 | 0.180646022 | 12534 | 252 |
| chr1_16509408_16440323_-69085-Stau2 | chr1 | Stau2 | 0 | 0.182309179 | 0 | 69086 | 648 |
| chr1_165608604_165604610_-3994-Mpzl1 | chr1 | Mpzl1 | 0.202796353 | 0 | 0 | 3995 | 514 |
| chr1_167237648_167234692_-2956-Uck2 | chr1 | Uck2 | 0 | 0.182309179 | 0.180646022 | 2957 | 400 |
| chr1_181832232_181825622_-6610-Lbr | chr1 | Lbr | 0.811185411 | 0 | 0.722584089 | 6611 | 529 |
| chr1_181961919_181923560_-38359-Enah | chr1 | Enah | 0.202796353 | 0 | 0 | 38360 | 779 |
| chr1_185397267_185386246_+11021-Eprs | chr1 | Eprs | 0.405592706 | 0.182309179 | 0.361292045 | 11022 | 622 |
| chr1_189798681_189786636_+12045-Ptpn14 | chr1 | Ptpn14 | 0 | 0.182309179 | 0.722584089 | 12046 | 483 |
| chr1_191763671_191749540_+14131-Lpgat1 | chr1 | Lpgat1 | 0 | 0.364618359 | 0 | 14132 | 616 |
| chr1_38085652_38053616_-32036-Rev1 | chr1 | Rev1 | 0 | 0 | 0.180646022 | 32037 | 3208 |
| chr1_53282092_53256629_-25463-Pms1 | chr1 | Pms1 | 1.216778117 | 0.911545897 | 1.806460223 | 25464 | 602 |
| chr1_53914632_53904249_-10383-Hecw2 | chr1 | Hecw2 | 0.202796353 | 0 | 0 | 10384 | 566 |
| chr1_55702133_55695745_+6388-Plcl1 | chr1 | Plcl1 | 0 | 0 | 0.180646022 | 6389 | 2679 |
| chr1_64576329_64550849_+25480-Creb1 | chr1 | Creb1 | 0 | 0.182309179 | 0 | 25481 | 960 |
| chr1_75196669_75194841_+1828-Ankzf1 | chr1 | Ankzf1 | 0.608389058 | 0 | 0.180646022 | 1829 | 1286 |
| chr1_87380008_87364104_+15904-Gigyf2 | chr1 | Gigyf2 | 8.111854113 | 3.828492767 | 5.41938067 | 15905 | 494 |
| chr1_87881939_87880368_+1571-Dgkd | chr1 | Dgkd | 0 | 0.364618359 | 0 | 1572 | 192 |
| chr1_89634294_89627761_+6533-Agap1 | chr1 | Agap1 | 0.405592706 | 0 | 0 | 6534 | 363 |
| chr1_89671271_89663661_+7610-Agap1 | chr1 | Agap1 | 0.405592706 | 0 | 0 | 7611 | 377 |
| chr1_91385346_91384565_-781-Ilkap | chr1 | Ilkap | 0 | 0.182309179 | 0 | 782 | 201 |
| chr1_92148494_92141520_-6974-Hdac4 | chr1 | Hdac4 | 0.202796353 | 1.093855076 | 0 | 6975 | 301 |
| chr1_92148494_92148256_-238-Hdac4 | chr1 | Hdac4 | 0.202796353 | 0 | 0 | 239 | 239 |
| chr10_123181800_123146794_-35006-Usp15 | chr10 | Usp15 | 0 | 0 | 0.361292045 | 35007 | 826 |
| chr10_12455564_12436281_-19283-Utrn | chr10 | Utrn | 0.608389058 | 0 | 0 | 19284 | 725 |
| chr10_12455564_12439627_-15937-Utrn | chr10 | Utrn | 0.405592706 | 0 | 0.180646022 | 15938 | 613 |
| chr10_18094908_18092845_+2063-Reps1 | chr10 | Reps1 | 0.405592706 | 0 | 0 | 2064 | 703 |
| chr10_18096336_18089448_+6888-Reps1 | chr10 | Reps1 | 0 | 0.546927538 | 0 | 6889 | 982 |
| chr10_22712091_22705693_-6398-Tbpl1 | chr10 | Tbpl1 | 0.202796353 | 0.546927538 | 0 | 6399 | 2039 |
| chr10_25289730_25267308_-22422-Akap7 | chr10 | Akap7 | 0.202796353 | 0 | 0.180646022 | 22423 | 579 |
| chr10_40469955_40466638_+3317-Cdk19 | chr10 | Cdk19 | 0 | 0.729236718 | 0 | 3318 | 346 |
| chr10_43395692_43393871_+1821-Pdss2 | chr10 | Pdss2 | 0.405592706 | 0 | 0 | 1822 | 306 |
| chr10_43413547_43393871_+19676-Pdss2 | chr10 | Pdss2 | 0.506990882 | 0.455772948 | 0 | 19677 | 339 |
| chr10_52436663_52429193_+7470-Nus1 | chr10 | Nus1 | 0 | 0.182309179 | 0 | 7471 | 463 |
| chr10_68751328_68748376_+2952-Tmem26 | chr10 | Tmem26 | 0 | 0.364618359 | 0 | 2953 | 335 |
| chr10_75279961_75274017_+5944-Specc1l | chr10 | Specc1l | 1.013981764 | 0.729236718 | 0.361292045 | 5945 | 344 |
| chr10_75700095_75684292_-15803-Cabin1 | chr10 | Cabin1 | 0.202796353 | 0 | 0 | 15804 | 629 |
| chr11_103055289_103046379_+8910-Nmt1 | chr11 | Nmt1 | 0.202796353 | 0.182309179 | 0.903230112 | 8911 | 356 |
| chr11_107620214_107613851_+6363-Helz | chr11 | Helz | 0.202796353 | 0 | 0 | 6364 | 733 |
| chr11_107649340_107592706_+56634-Helz | chr11 | Helz | 0.405592706 | 0.364618359 | 0 | 56635 | 2938 |
| chr11_109977873_109971685_-6188-Abca8b | chr11 | Abca8b | 0 | 0.364618359 | 0 | 6189 | 976 |
| chr11_116263297_116262860_-437-Srp68 | chr11 | Srp68 | 0.405592706 | 0 | 0.541938067 | 438 | 193 |
| chr11_20727639_20703711_-23928-Aftph | chr11 | Aftph | 0.202796353 | 0 | 0 | 23929 | 2414 |
| chr11_22068506_22053432_-15074-Ehbp1 | chr11 | Ehbp1 | 0 | 0.546927538 | 0.541938067 | 15075 | 751 |
| chr11_3645523_3641211_-4312-Tug1 | chr11 | Tug1 | 0 | 0.182309179 | 0 | 4313 | 4313 |
| chr11_43705477_43704534_+943-Pwwp2a | chr11 | Pwwp2a | 3.853130704 | 1.823091794 | 3.432274424 | 944 | 944 |
| chr11_60275002_60258856_-16146-Tom1l2 | chr11 | Tom1l2 | 0.202796353 | 0.364618359 | 0 | 16147 | 640 |
| chr11_60275002_60270318_-4684-Tom1l2 | chr11 | Tom1l2 | 0 | 0.09115459 | 0.090323011 | 4685 | 229 |
| chr11_6193476_6193154_-322-Nudcd3 | chr11 | Nudcd3 | 0 | 0.182309179 | 0 | 323 | 323 |
| chr11_62161910_62146306_+15604-Specc1 | chr11 | Specc1 | 0.202796353 | 0 | 0 | 15605 | 586 |
| chr11_6317080_6311009_+6071-Ogdh | chr11 | Ogdh | 0 | 0.182309179 | 0.180646022 | 6072 | 6072 |
| chr11_6317080_6313788_+3292-Ogdh | chr11 | Ogdh | 0.811185411 | 0.364618359 | 0.903230112 | 3293 | 295 |
| chr11_69651723_69649728_+1995-Fxr2 | chr11 | Fxr2 | 0 | 0 | 0.180646022 | 1996 | 825 |
| chr11_74990215_74928993_+61222-Smg6 | chr11 | Smg6 | 0 | 0.182309179 | 0 | 61223 | 2778 |
| chr11_75391227_75390072_+1155-Smyd4 | chr11 | Smyd4 | 1.622370823 | 1.640782614 | 1.264522156 | 1156 | 1156 |
| chr11_78009794_78006727_+3067-Phf12 | chr11 | Phf12 | 0 | 0.182309179 | 0 | 3068 | 467 |
| chr11_84195687_84193079_+2608-Acaca | chr11 | Acaca | 0 | 0 | 0.541938067 | 2609 | 297 |
| chr11_8479665_8478225_-1440-Tns3 | chr11 | Tns3 | 0.202796353 | 0.182309179 | 0 | 1441 | 360 |
| chr11_85104026_85077265_-26761-Usp32 | chr11 | Usp32 | 0.202796353 | 0 | 0 | 26762 | 513 |
| chr11_90594841_90571722_-23119-Stxbp4 | chr11 | Stxbp4 | 0 | 0.182309179 | 0 | 23120 | 345 |
| chr11_93912458_93905159_+7299-Mbtd1 | chr11 | Mbtd1 | 0 | 0 | 0.361292045 | 7300 | 547 |
| chr12_111618383_111592577_+25806-Mark3 | chr12 | Mark3 | 0.811185411 | 0.364618359 | 0 | 25807 | 361 |
| chr12_116324210_116317830_+6380-Esyt2 | chr12 | Esyt2 | 1.622370823 | 1.823091794 | 2.167752268 | 6381 | 840 |
| chr12_3648389_3617606_+30783-Dtnb | chr12 | Dtnb | 1.216778117 | 0.364618359 | 1.083876134 | 30784 | 514 |
| chr12_3648389_3632810_+15579-Dtnb | chr12 | Dtnb | 0 | 0 | 0.361292045 | 15580 | 428 |
| chr12_40640445_40631522_+8923-Dock4 | chr12 | Dock4 | 0.811185411 | 0.182309179 | 0 | 8924 | 331 |
| chr12_5006791_4961218_+45573-Atad2b | chr12 | Atad2b | 0.202796353 | 0 | 0.180646022 | 45574 | 1224 |
| chr12_51661713_51647994_-13719-Strn3 | chr12 | Strn3 | 0.608389058 | 2.552328511 | 2.34839829 | 13720 | 703 |
| chr12_51772705_51772274_-431-Hectd1 | chr12 | Hectd1 | 0.202796353 | 0.182309179 | 0.361292045 | 432 | 344 |
| chr12_52519967_52516079_+3888-Arhgap5 | chr12 | Arhgap5 | 0.202796353 | 0.182309179 | 0.361292045 | 3889 | 3889 |
| chr12_53142768_53139381_+3387-Akap6 | chr12 | Akap6 | 0 | 1.093855076 | 1.083876134 | 3388 | 3388 |
| chr12_69303962_69302324_+1638-Klhdc2 | chr12 | Klhdc2 | 0.608389058 | 0.364618359 | 0 | 1639 | 316 |
| chr12_81316104_81314260_-1844-Slc8a3 | chr12 | Slc8a3 | 0.811185411 | 0.546927538 | 0.722584089 | 1845 | 1845 |
| chr12_85034034_85028770_+5264-Ylpm1 | chr12 | Ylpm1 | 0.608389058 | 0.729236718 | 1.625814201 | 5265 | 2236 |
| chr12_98518832_98489905_-28927-Kcnk10 | chr12 | Kcnk10 | 0 | 0 | 0.180646022 | 28928 | 614 |
| chr13_103889285_103884143_-5142-Erbb2ip | chr13 | Erbb2ip | 0.202796353 | 0 | 0 | 5143 | 364 |
| chr13_104052639_104046189_-6450-Nln | chr13 | Nln | 0.202796353 | 0 | 0 | 6451 | 749 |
| chr13_17772957_17738917_-34040-Cdk13 | chr13 | Cdk13 | 1.013981764 | 0.729236718 | 0.180646022 | 34041 | 1566 |
| chr13_17772957_17750257_-22700-Cdk13 | chr13 | Cdk13 | 0.811185411 | 1.458473435 | 0.541938067 | 22701 | 1386 |
| chr13_17772957_17763104_-9853-Cdk13 | chr13 | Cdk13 | 0.811185411 | 0.911545897 | 0.541938067 | 9854 | 1139 |
| chr13_18814293_18810432_+3861-Vps41 | chr13 | Vps41 | 0 | 0.546927538 | 0 | 3862 | 334 |
| chr13_18830793_18810432_+20361-Vps41 | chr13 | Vps41 | 0 | 0 | 0.180646022 | 20362 | 735 |
| chr13_28669994_28668370_-1624-2610307P16Rik | chr13 | 2610307P16Rik | 0.811185411 | 0 | 0 | 1625 | 548 |
| chr13_35816575_35815909_+666-Cdyl | chr13 | Cdyl | 0.202796353 | 0.182309179 | 0 | 667 | 667 |
| chr13_37899787_37888871_+10916-Rreb1 | chr13 | Rreb1 | 0.811185411 | 0.546927538 | 0.180646022 | 10917 | 1890 |
| chr13_43425214_43419576_-5638-Ranbp9 | chr13 | Ranbp9 | 0 | 0 | 0.361292045 | 5639 | 598 |
| chr13_44903258_44902226_+1032-Jarid2 | chr13 | Jarid2 | 0.202796353 | 0.182309179 | 0 | 1033 | 1033 |
| chr13_48606360_48590287_-16073-Ptpdc1 | chr13 | Ptpdc1 | 0 | 0 | 0.180646022 | 16074 | 643 |
| chr13_55248077_55233895_+14182-Nsd1 | chr13 | Nsd1 | 3.853130704 | 1.276164256 | 1.625814201 | 14183 | 2869 |
| chr13_63068285_63061035_+7250-2010111I01Rik | chr13 | 2010111I01Rik | 0 | 0.182309179 | 0.180646022 | 7251 | 406 |
| chr13_64248466_64243634_-4832-Cdc14b | chr13 | Cdc14b | 0.101398176 | 0 | 0 | 4833 | 322 |
| chr13_64248499_64243634_-4865-Cdc14b | chr13 | Cdc14b | 0 | 0 | 0.541938067 | 4866 | 355 |
| chr13_91564694_91524585_+40109-Ssbp2 | chr13 | Ssbp2 | 0.202796353 | 0 | 0 | 40110 | 220 |
| chr13_93356079_93341756_+14323-Homer1 | chr13 | Homer1 | 0.405592706 | 0 | 0 | 14324 | 522 |
| chr13_97150413_97141927_+8486-Gfm2 | chr13 | Gfm2 | 0 | 0.182309179 | 0.180646022 | 8487 | 540 |
| chr13_9735466_9720773_-14693-Zmynd11 | chr13 | Zmynd11 | 0 | 0.729236718 | 0 | 14694 | 295 |
| chr14_13951263_13950132_-1131-Thoc7 | chr14 | Thoc7 | 0 | 0.364618359 | 0 | 1132 | 195 |
| chr14_17963321_17963061_+260-Thrb | chr14 | Thrb | 0 | 0 | 0.541938067 | 261 | 261 |
| chr14_24192954_24190318_-2636-Dlg5 | chr14 | Dlg5 | 0 | 0 | 0.541938067 | 2637 | 491 |
| chr14_25581673_25502655_+79018-Zmiz1 | chr14 | Zmiz1 | 0 | 0 | 0.180646022 | 79019 | 514 |
| chr14_25581673_25572020_+9653-Zmiz1 | chr14 | Zmiz1 | 0 | 0 | 0.180646022 | 9654 | 329 |
| chr14_32262831_32250017_+12814-Parg | chr14 | Parg | 0.405592706 | 0 | 0 | 12815 | 365 |
| chr14_47016795_47016277_+518-Samd4 | chr14 | Samd4 | 3.853130704 | 1.823091794 | 0.722584089 | 519 | 519 |
| chr14_48179276_48168197_+11079-Peli2 | chr14 | Peli2 | 0.405592706 | 0 | 0 | 11080 | 11080 |
| chr14_54759745_54758439_-1306-Slc7a8 | chr14 | Slc7a8 | 0 | 0 | 0.180646022 | 1307 | 357 |
| chr14_60875307_60843208_+32099-Mipep | chr14 | Mipep | 0.811185411 | 0 | 0 | 32100 | 391 |
| chr14_70267506_70235140_-32366-Ppp3cc | chr14 | Ppp3cc | 0 | 0.182309179 | 0 | 32367 | 1089 |
| chr14_87487287_87472076_+15211-Tdrd3 | chr14 | Tdrd3 | 0.811185411 | 0.546927538 | 0.903230112 | 15212 | 949 |
| chr15_4059996_4047465_+12531-Oxct1 | chr15 | Oxct1 | 0.608389058 | 0.729236718 | 0.903230112 | 12532 | 454 |
| chr15_44420873_44388469_-32404-Nudcd1 | chr15 | Nudcd1 | 0 | 0.182309179 | 0 | 32405 | 1181 |
| chr15_50846942_50846014_-928-Trps1 | chr15 | Trps1 | 0 | 0.182309179 | 0.541938067 | 929 | 929 |
| chr15_5170016_5160603_+9413-Prkaa1 | chr15 | Prkaa1 | 0 | 0.364618359 | 0 | 9414 | 2769 |
| chr15_62107465_62107148_+317-Pvt1 | chr15 | Pvt1 | 0.202796353 | 0.911545897 | 0 | 318 | 318 |
| chr15_68170223_68165753_-4470-Zfat | chr15 | Zfat | 0.202796353 | 0 | 0 | 4471 | 412 |
| chr15_73031643_73012806_-18837-Trappc9 | chr15 | Trappc9 | 0 | 0 | 0.180646022 | 18838 | 465 |
| chr15_79543856_79541034_-2822-Ddx17 | chr15 | Ddx17 | 0 | 0.182309179 | 0 | 2823 | 385 |
| chr15_81392322_81382980_-9342-St13 | chr15 | St13 | 0 | 0 | 0.361292045 | 9343 | 269 |
| chr15_82857284_82825231_-32053-Tcf20 | chr15 | Tcf20 | 1.216778117 | 0 | 0.722584089 | 32054 | 5866 |
| chr15_82857284_82851513_-5771-Tcf20 | chr15 | Tcf20 | 4.461519762 | 5.651584561 | 5.238734648 | 5772 | 5772 |
| chr15_85376702_85359454_+17248-Atxn10 | chr15 | Atxn10 | 0.202796353 | 0 | 0 | 17249 | 531 |
| chr15_88812159_88811297_-862-Alg12 | chr15 | Alg12 | 0 | 0.364618359 | 0 | 863 | 328 |
| chr15_91516494_91497393_-19101-Slc2a13 | chr15 | Slc2a13 | 0 | 0 | 0.361292045 | 19102 | 369 |
| chr15_93465245_93424014_+41231-Pphln1 | chr15 | Pphln1 | 0 | 0.182309179 | 0 | 41232 | 672 |
| chr15_98742829_98740337_-2492-Arf3 | chr15 | Arf3 | 0 | 0.182309179 | 0 | 2493 | 631 |
| chr16_10644881_10611003_+33878-Clec16a | chr16 | Clec16a | 0.608389058 | 0.182309179 | 0.722584089 | 33879 | 813 |
| chr16_11128703_11104566_-24137-Txndc11 | chr16 | Txndc11 | 0.202796353 | 0 | 0 | 24138 | 539 |
| chr16_11128703_11116795_-11908-Txndc11 | chr16 | Txndc11 | 1.825167175 | 0.911545897 | 0.361292045 | 11909 | 445 |
| chr16_13647299_13629583_-17716-Parn | chr16 | Parn | 0 | 0.09115459 | 0 | 17717 | 222 |
| chr16_13699075_13697333_+1742-Bfar | chr16 | Bfar | 0 | 0 | 0.361292045 | 1743 | 377 |
| chr16_17389457_17373350_-16107-Pi4ka | chr16 | Pi4ka | 0.202796353 | 0 | 0 | 16108 | 700 |
| chr16_31812824_31796866_+15958-Dlg1 | chr16 | Dlg1 | 0.202796353 | 0 | 0 | 15959 | 423 |
| chr16_32961744_32950292_+11452-Lrch3 | chr16 | Lrch3 | 2.230759881 | 0.364618359 | 0.903230112 | 11453 | 625 |
| chr16_33434974_33421321_+13653-Zfp148 | chr16 | Zfp148 | 1.216778117 | 0.546927538 | 1.445168179 | 13654 | 567 |
| chr16_43577748_43569681_+8067-Zbtb20 | chr16 | Zbtb20 | 0 | 0.911545897 | 0.361292045 | 8068 | 511 |
| chr16_58433463_58424561_+8902-Dcbld2 | chr16 | Dcbld2 | 0 | 0 | 0.180646022 | 8903 | 366 |
| chr16_76352549_76330746_-21803-Nrip1 | chr16 | Nrip1 | 0 | 0 | 0.361292045 | 21804 | 197 |
| chr17_14375519_14344327_+31192-Smoc2 | chr17 | Smoc2 | 0 | 0.182309179 | 0.8129071 | 31193 | 477 |
| chr17_30530365_30513556_-16809-Btbd9 | chr17 | Btbd9 | 0.202796353 | 0.729236718 | 0 | 16810 | 1181 |
| chr17_50611195_50606295_+4900-Plcl2 | chr17 | Plcl2 | 0.608389058 | 0 | 0 | 4901 | 2691 |
| chr17_6139156_6137211_+1945-Tulp4 | chr17 | Tulp4 | 27.78310034 | 21.51248317 | 28.54207153 | 1946 | 1946 |
| chr17_63490887_63471408_-19479-Fbxl17 | chr17 | Fbxl17 | 0.811185411 | 0 | 0 | 19480 | 513 |
| chr17_64625451_64624528_+923-Man2a1 | chr17 | Man2a1 | 0.405592706 | 0.364618359 | 0 | 924 | 397 |
| chr17_64714712_64710704_+4008-Man2a1 | chr17 | Man2a1 | 0 | 0.182309179 | 0 | 4009 | 626 |
| chr17_65595054_65592291_-2763-Vapa | chr17 | Vapa | 0.202796353 | 0 | 0 | 2764 | 338 |
| chr17_78315746_78313124_+2622-Crim1 | chr17 | Crim1 | 0.405592706 | 0.911545897 | 0 | 2623 | 381 |
| chr17_80460047_80445430_-14617-Sos1 | chr17 | Sos1 | 0 | 0 | 0.180646022 | 14618 | 987 |
| chr18_23597558_23583084_+14474-Dtna | chr18 | Dtna | 0 | 0 | 0.180646022 | 14475 | 639 |
| chr18_25344962_25339714_+5248-AW554918 | chr18 | AW554918 | 0 | 0.729236718 | 0.180646022 | 5249 | 770 |
| chr18_31835481_31827262_+8219-Wdr33 | chr18 | Wdr33 | 0.101398176 | 0.364618359 | 0 | 8220 | 747 |
| chr18_35154540_35152601_+1939-Ctnna1 | chr18 | Ctnna1 | 0 | 0.182309179 | 0 | 1940 | 303 |
| chr18_39150118_39120119_+29999-Arhgap26 | chr18 | Arhgap26 | 0.202796353 | 0.729236718 | 0 | 30000 | 510 |
| chr18_39246924_39227368_+19556-Arhgap26 | chr18 | Arhgap26 | 0 | 0.364618359 | 0 | 19557 | 394 |
| chr18_53906669_53895531_+11138-Csnk1g3 | chr18 | Csnk1g3 | 0 | 0.729236718 | 0 | 11139 | 535 |
| chr18_6115850_6111685_-4165-Arhgap12 | chr18 | Arhgap12 | 0 | 1.093855076 | 0 | 4166 | 792 |
| chr18_6450639_6448905_-1734-Epc1 | chr18 | Epc1 | 0 | 0.364618359 | 0 | 1735 | 766 |
| chr18_65656632_65638750_+17882-Zfp532 | chr18 | Zfp532 | 1.216778117 | 0.729236718 | 0.541938067 | 17883 | 1005 |
| chr18_65656632_65644193_+12439-Zfp532 | chr18 | Zfp532 | 1.41957447 | 0.364618359 | 0 | 12440 | 823 |
| chr18_65656632_65651560_+5072-Zfp532 | chr18 | Zfp532 | 2.230759881 | 3.646183588 | 3.07098238 | 5073 | 445 |
| chr18_69564684_69564034_+650-Tcf4 | chr18 | Tcf4 | 0 | 0 | 0.361292045 | 651 | 180 |
| chr18_73678039_73675161_-2878-Smad4 | chr18 | Smad4 | 0.202796353 | 0 | 0 | 2879 | 582 |
| chr18_75063173_75052981_+10192-Dym | chr18 | Dym | 0 | 0.546927538 | 0.180646022 | 10193 | 354 |
| chr18_75082507_75080203_+2304-Dym | chr18 | Dym | 0 | 0.364618359 | 0.541938067 | 2305 | 269 |
| chr18_75126919_75114790_+12129-Dym | chr18 | Dym | 1.41957447 | 1.640782614 | 1.987106246 | 12130 | 697 |
| chr19_17678557_17642493_-36064-Pcsk5 | chr19 | Pcsk5 | 0.202796353 | 0 | 0 | 36065 | 576 |
| chr19_28531986_28530873_-1113-Glis3 | chr19 | Glis3 | 0.405592706 | 0 | 0.180646022 | 1114 | 1114 |
| chr19_29755017_29743533_-11484-9930021J03Rik | chr19 | 9930021J03Rik | 0 | 0.182309179 | 0.180646022 | 11485 | 1544 |
| chr19_37088587_37044522_-44065-Cpeb3 | chr19 | Cpeb3 | 0 | 0 | 0.361292045 | 44066 | 674 |
| chr19_41555701_41545875_+9826-Lcor | chr19 | Lcor | 1.622370823 | 1.093855076 | 1.264522156 | 9827 | 515 |
| chr19_45640521_45630865_-9656-Fbxw4 | chr19 | Fbxw4 | 1.013981764 | 1.458473435 | 0.180646022 | 9657 | 510 |
| chr19_45640521_45635735_-4786-Fbxw4 | chr19 | Fbxw4 | 0.811185411 | 0.182309179 | 0 | 4787 | 415 |
| chr19_45892148_45885952_-6196-9130011E15Rik | chr19 | 9130011E15Rik | 0.405592706 | 0 | 0 | 6197 | 286 |
| chr19_4788042_4784724_-3318-Rbm4 | chr19 | Rbm4 | 0.202796353 | 0 | 0.090323011 | 3319 | 1526 |
| chr19_4794009_4784724_-9285-Rbm4 | chr19 | Rbm4 | 0.304194529 | 0.273463769 | 0.903230112 | 9286 | 2143 |
| chr19_4794009_4787361_-6648-Rbm4 | chr19 | Rbm4 | 1.41957447 | 1.093855076 | 1.806460223 | 6649 | 1299 |
| chr19_53988383_53987454_+929-Shoc2 | chr19 | Shoc2 | 0 | 0.546927538 | 0.361292045 | 930 | 930 |
| chr19_56576284_56570416_+5868-Nhlrc2 | chr19 | Nhlrc2 | 0 | 0 | 0.361292045 | 5869 | 708 |
| chr19_57379175_57378549_+626-Fam160b1 | chr19 | Fam160b1 | 1.622370823 | 0.729236718 | 0.903230112 | 627 | 491 |
| chr19_57777945_57751630_+26315-Atrnl1 | chr19 | Atrnl1 | 0 | 0.364618359 | 0 | 26316 | 394 |
| chr19_57797503_57751630_+45873-Atrnl1 | chr19 | Atrnl1 | 0 | 0 | 0.180646022 | 45874 | 473 |
| chr2_104471847_104470749_-1098-Hipk3 | chr2 | Hipk3 | 2.83914894 | 0.911545897 | 1.445168179 | 1099 | 1099 |
| chr2_104609186_104608871_+315-Cstf3 | chr2 | Cstf3 | 0 | 0 | 0.180646022 | 316 | 316 |
| chr2_120825315_120806759_-18556-Ttbk2 | chr2 | Ttbk2 | 0 | 0 | 0.180646022 | 18557 | 358 |
| chr2_126799234_126797467_-1767-Trpm7 | chr2 | Trpm7 | 0 | 0 | 0.903230112 | 1768 | 433 |
| chr2_129811237_129810488_+749-Stk35 | chr2 | Stk35 | 2.230759881 | 0.911545897 | 1.264522156 | 750 | 750 |
| chr2_131516443_131511984_+4459-Smox | chr2 | Smox | 3.650334351 | 2.734637691 | 2.167752268 | 4460 | 480 |
| chr2_140057499_140042094_-15405-Tasp1 | chr2 | Tasp1 | 0.202796353 | 0.364618359 | 0.180646022 | 15406 | 350 |
| chr2_142740991_142739523_-1468-Kif16b | chr2 | Kif16b | 0.405592706 | 0.546927538 | 1.083876134 | 1469 | 172 |
| chr2_143974609_143963104_-11505-Rrbp1 | chr2 | Rrbp1 | 0 | 0.182309179 | 0 | 11506 | 1042 |
| chr2_152308431_152302152_+6279-Tbc1d20 | chr2 | Tbc1d20 | 0 | 0.182309179 | 0 | 6280 | 556 |
| chr2_156178952_156177633_-1319-Rbm39 | chr2 | Rbm39 | 0.405592706 | 0.364618359 | 0.180646022 | 1320 | 1320 |
| chr2_156749356_156745835_+3521-Dlgap4 | chr2 | Dlgap4 | 0.405592706 | 0 | 0.180646022 | 3522 | 1173 |
| chr2_158058827_158035996_+22831-Rprd1b | chr2 | Rprd1b | 0.405592706 | 0.546927538 | 0 | 22832 | 680 |
| chr2_168571633_168567139_-4494-Nfatc2 | chr2 | Nfatc2 | 1.41957447 | 0.729236718 | 0.903230112 | 4495 | 1208 |
| chr2_169886586_169883526_+3060-Tshz2 | chr2 | Tshz2 | 0.202796353 | 0.182309179 | 0 | 3061 | 3061 |
| chr2_18146872_18101458_+45414-Mllt10 | chr2 | Mllt10 | 0.202796353 | 0.729236718 | 0 | 45415 | 624 |
| chr2_23545579_23537328_-8251-Spopl | chr2 | Spopl | 1.013981764 | 0.729236718 | 2.167752268 | 8252 | 869 |
| chr2_24825067_24813527_-11540-Ehmt1 | chr2 | Ehmt1 | 0.405592706 | 0.182309179 | 0 | 11541 | 346 |
| chr2_32183300_32182458_+842-Prrc2b | chr2 | Prrc2b | 0 | 0 | 0.361292045 | 843 | 347 |
| chr2_33324703_33260478_-64225-Ralgps1 | chr2 | Ralgps1 | 0 | 0.273463769 | 0 | 64226 | 445 |
| chr2_37983105_37936619_-46486-Dennd1a | chr2 | Dennd1a | 0 | 0.182309179 | 0 | 46487 | 274 |
| chr2_44756435_44752047_-4388-Gtdc1 | chr2 | Gtdc1 | 0 | 0 | 0.180646022 | 4389 | 342 |
| chr2_59772539_59771132_+1407-Tanc1 | chr2 | Tanc1 | 0.202796353 | 0 | 0 | 1408 | 372 |
| chr2_59962630_59948087_-14543-Baz2b | chr2 | Baz2b | 0.202796353 | 0 | 0.180646022 | 14544 | 1276 |
| chr2_6721613_6721417_-196-Celf2 | chr2 | Celf2 | 0 | 0.182309179 | 0 | 197 | 197 |
| chr2_69922933_69914414_+8519-Ubr3 | chr2 | Ubr3 | 0 | 0.364618359 | 0 | 8520 | 493 |
| chr2_70786995_70768862_-18133-Tlk1 | chr2 | Tlk1 | 0.202796353 | 0 | 0 | 18134 | 260 |
| chr2_74573626_74547838_-25788-Lnp | chr2 | Lnp | 1.013981764 | 0.182309179 | 0.903230112 | 25789 | 555 |
| chr2_91825352_91805024_+20328-Ambra1 | chr2 | Ambra1 | 0.405592706 | 0.364618359 | 0 | 20329 | 449 |
| chr2_93013889_93012627_-1262-Prdm11 | chr2 | Prdm11 | 0.202796353 | 0 | 0 | 1263 | 492 |
| chr3_106539603_106531137_-8466-Cept1 | chr3 | Cept1 | 1.216778117 | 1.458473435 | 0.722584089 | 8467 | 704 |
| chr3_122748477_122747889_+588-Pde5a | chr3 | Pde5a | 0 | 0.182309179 | 0 | 589 | 589 |
| chr3_133330512_133320936_+9576-Ppa2 | chr3 | Ppa2 | 0.202796353 | 0 | 0 | 9577 | 284 |
| chr3_137906057_137902187_+3870-Dnajb14 | chr3 | Dnajb14 | 0 | 0 | 0.722584089 | 3871 | 564 |
| chr3_152747773_152738186_+9587-Pigk | chr3 | Pigk | 0.405592706 | 0.182309179 | 0.180646022 | 9588 | 888 |
| chr3_153411871_153411462_-409-St6galnac3 | chr3 | St6galnac3 | 0.202796353 | 0.364618359 | 0 | 410 | 410 |
| chr3_32865966_32861771_+4195-Usp13 | chr3 | Usp13 | 0 | 0 | 0.180646022 | 4196 | 328 |
| chr3_51326035_51299783_-26252-Elf2 | chr3 | Elf2 | 3.244741645 | 0.546927538 | 1.083876134 | 26253 | 587 |
| chr3_51326035_51308050_-17985-Elf2 | chr3 | Elf2 | 9.531428583 | 6.198512099 | 5.780672715 | 17986 | 484 |
| chr3_51857076_51855499_-1577-Maml3 | chr3 | Maml3 | 0.202796353 | 0.273463769 | 0 | 1578 | 1578 |
| chr3_66996590_66994517_+2073-Rsrc1 | chr3 | Rsrc1 | 0 | 0.182309179 | 0.361292045 | 2074 | 322 |
| chr3_88349444_88346445_+2999-Smg5 | chr3 | Smg5 | 0.811185411 | 0 | 0 | 3000 | 395 |
| chr3_88985790_88965812_+19978-Ash1l | chr3 | Ash1l | 0.608389058 | 1.276164256 | 0.722584089 | 19979 | 5075 |
| chr3_89007877_89001773_+6104-Ash1l | chr3 | Ash1l | 0 | 0 | 0.361292045 | 6105 | 838 |
| chr4_108719812_108717789_-2023-Zfyve9 | chr4 | Zfyve9 | 1.825167175 | 0.546927538 | 1.083876134 | 2024 | 2024 |
| chr4_108727545_108717789_-9756-Zfyve9 | chr4 | Zfyve9 | 0.202796353 | 0 | 0 | 9757 | 2220 |
| chr4_109935698_109883636_+52062-Faf1 | chr4 | Faf1 | 0 | 0 | 0.541938067 | 52063 | 601 |
| chr4_117593162_117582600_+10562-Eri3 | chr4 | Eri3 | 0 | 0.364618359 | 0.180646022 | 10563 | 1582 |
| chr4_117615241_117564621_+50620-Eri3 | chr4 | Eri3 | 0.405592706 | 0 | 0 | 50621 | 804 |
| chr4_117615241_117582600_+32641-Eri3 | chr4 | Eri3 | 0 | 0.182309179 | 0 | 32642 | 1655 |
| chr4_121190258_121170618_-19640-Rlf | chr4 | Rlf | 0 | 0 | 0.180646022 | 19641 | 573 |
| chr4_121190258_121182606_-7652-Rlf | chr4 | Rlf | 0.405592706 | 0.182309179 | 0.180646022 | 7653 | 370 |
| chr4_132673032_132656693_+16339-Eya3 | chr4 | Eya3 | 0 | 0.182309179 | 0.270969033 | 16340 | 463 |
| chr4_132707123_132689705_+17418-Eya3 | chr4 | Eya3 | 0.811185411 | 0 | 0.180646022 | 17419 | 801 |
| chr4_133723051_133719537_-3514-Arid1a | chr4 | Arid1a | 1.013981764 | 0.729236718 | 0.722584089 | 3515 | 780 |
| chr4_141494398_141483725_-10673-Spen | chr4 | Spen | 0.405592706 | 0.182309179 | 0.270969033 | 10674 | 969 |
| chr4_145152798_145148317_-4481-Vps13d | chr4 | Vps13d | 0.811185411 | 1.093855076 | 1.445168179 | 4482 | 1184 |
| chr4_151835883_151830418_-5465-Camta1 | chr4 | Camta1 | 0 | 0.273463769 | 0 | 5466 | 189 |
| chr4_34839986_34839332_-654-Zfp292 | chr4 | Zfp292 | 0.608389058 | 0 | 0.722584089 | 655 | 370 |
| chr4_48617331_48604581_+12750-Tmeff1 | chr4 | Tmeff1 | 0 | 0 | 0.180646022 | 12751 | 367 |
| chr4_58885498_58861525_-23973-AI314180 | chr4 | AI314180 | 0.202796353 | 0.182309179 | 0.541938067 | 23974 | 1031 |
| chr4_59691338_59690144_+1194-E130308A19Rik | chr4 | E130308A19Rik | 0 | 0.546927538 | 0.180646022 | 1195 | 1195 |
| chr4_74315649_74306965_+8684-Kdm4c | chr4 | Kdm4c | 0 | 0.182309179 | 0.361292045 | 8685 | 292 |
| chr4_82383661_82350389_-33272-Nfib | chr4 | Nfib | 0 | 0 | 0.180646022 | 33273 | 363 |
| chr4_82854115_82835446_-18669-Zdhhc21 | chr4 | Zdhhc21 | 0 | 0.364618359 | 0.361292045 | 18670 | 694 |
| chr4_84293662_84291693_-1969-Bnc2 | chr4 | Bnc2 | 0.608389058 | 1.823091794 | 1.625814201 | 1970 | 1970 |
| chr4_85006524_84971131_+35393-Cntln | chr4 | Cntln | 0 | 0.182309179 | 0.180646022 | 35394 | 1016 |
| chr4_87794011_87782287_-11724-Mllt3 | chr4 | Mllt3 | 0 | 0 | 0.361292045 | 11725 | 450 |
| chr4_9610938_9583812_-27126-Asph | chr4 | Asph | 0.405592706 | 1.093855076 | 0 | 27127 | 576 |
| chr5_100485855_100475689_-10166-Lin54 | chr5 | Lin54 | 0 | 0.364618359 | 0.180646022 | 10167 | 983 |
| chr5_103501912_103501367_+545-Ptpn13 | chr5 | Ptpn13 | 0.202796353 | 0 | 0 | 546 | 546 |
| chr5_103876289_103862918_-13371-Klhl8 | chr5 | Klhl8 | 0.608389058 | 0 | 0.361292045 | 13372 | 1797 |
| chr5_104541262_104538826_+2436-D930016D06Rik | chr5 | D930016D06Rik | 0 | 1.093855076 | 0 | 2437 | 245 |
| chr5_105911508_105891118_+20390-Zfp326 | chr5 | Zfp326 | 0.405592706 | 0 | 0 | 20391 | 786 |
| chr5_106619642_106618071_-1571-Zfp644 | chr5 | Zfp644 | 0 | 0.182309179 | 0 | 1572 | 461 |
| chr5_106638635_106618071_-20564-Zfp644 | chr5 | Zfp644 | 4.055927057 | 7.110057996 | 7.045194871 | 20565 | 4213 |
| chr5_106638635_106634831_-3804-Zfp644 | chr5 | Zfp644 | 2.83914894 | 4.557729485 | 4.877442603 | 3805 | 3632 |
| chr5_106666845_106618071_-48774-Zfp644 | chr5 | Zfp644 | 0 | 0.911545897 | 0.541938067 | 48775 | 4274 |
| chr5_106666845_106634831_-32014-Zfp644 | chr5 | Zfp644 | 0 | 1.276164256 | 0.8129071 | 32015 | 3693 |
| chr5_107799260_107788199_-11061-Evi5 | chr5 | Evi5 | 0 | 0.729236718 | 0 | 11062 | 678 |
| chr5_110756766_110753804_-2962-Ep400 | chr5 | Ep400 | 0 | 0.364618359 | 0 | 2963 | 1481 |
| chr5_110756766_110755397_-1369-Ep400 | chr5 | Ep400 | 4.055927057 | 1.823091794 | 1.625814201 | 1370 | 1370 |
| chr5_111183431_111182924_+507-Ttc28 | chr5 | Ttc28 | 0 | 0.182309179 | 0 | 508 | 508 |
| chr5_118095729_118092496_-3233-Fbxw8 | chr5 | Fbxw8 | 0.202796353 | 0 | 0 | 3234 | 404 |
| chr5_118593570_118593333_+237-Med13l | chr5 | Med13l | 0.608389058 | 0.911545897 | 0 | 238 | 238 |
| chr5_121273799_121263885_+9914-Gm15800 | chr5 | Gm15800 | 0 | 0 | 0.361292045 | 9915 | 503 |
| chr5_123104511_123099837_+4674-Tmem120b | chr5 | Tmem120b | 0 | 0.182309179 | 0 | 4675 | 363 |
| chr5_123745589_123744955_-634-Rsrc2 | chr5 | Rsrc2 | 0 | 0.182309179 | 0 | 635 | 250 |
| chr5_130191043_130187401_+3642-Rabgef1 | chr5 | Rabgef1 | 0.608389058 | 0 | 0.180646022 | 3643 | 353 |
| chr5_130198116_130187401_+10715-Rabgef1 | chr5 | Rabgef1 | 0.405592706 | 0.729236718 | 0.180646022 | 10716 | 523 |
| chr5_134500149_134491893_-8256-Clip2 | chr5 | Clip2 | 0.202796353 | 0 | 0 | 8257 | 708 |
| chr5_136340137_136332624_-7513-Cux1 | chr5 | Cux1 | 0 | 0 | 0.090323011 | 7514 | 154 |
| chr5_147455188_147450653_+4535-Pan3 | chr5 | Pan3 | 0.202796353 | 0 | 0 | 4536 | 260 |
| chr5_148921412_148918596_-2816-Katnal1 | chr5 | Katnal1 | 0.202796353 | 0 | 0 | 2817 | 337 |
| chr5_16333825_16299929_+33896-Cacna2d1 | chr5 | Cacna2d1 | 0 | 0 | 0.180646022 | 33897 | 1014 |
| chr5_23565265_23540375_-24890-Srpk2 | chr5 | Srpk2 | 0.202796353 | 0 | 0 | 24891 | 544 |
| chr5_28368313_28358274_+10039-Rbm33 | chr5 | Rbm33 | 0.202796353 | 0 | 0 | 10040 | 557 |
| chr5_32491189_32478066_+13123-Ppp1cb | chr5 | Ppp1cb | 0.202796353 | 0.182309179 | 0 | 13124 | 1063 |
| chr5_32664400_32640331_+24069-Yes1 | chr5 | Yes1 | 0 | 0.546927538 | 0.361292045 | 24070 | 1425 |
| chr5_33846267_33843112_+3155-Whsc1 | chr5 | Whsc1 | 0.202796353 | 0.364618359 | 0.180646022 | 3156 | 789 |
| chr5_33868878_33843112_+25766-Whsc1 | chr5 | Whsc1 | 0 | 0.182309179 | 0 | 25767 | 1913 |
| chr5_34444913_34419998_+24915-Fam193a | chr5 | Fam193a | 0 | 0 | 0.180646022 | 24916 | 1854 |
| chr5_36525048_36519273_-5775-Tbc1d14 | chr5 | Tbc1d14 | 0.101398176 | 0 | 0 | 5776 | 427 |
| chr5_3772787_3747021_-25766-Ankib1 | chr5 | Ankib1 | 4.258723409 | 3.09925605 | 3.612920447 | 25767 | 873 |
| chr5_65594548_65565989_+28559-Ube2k | chr5 | Ube2k | 0 | 0.364618359 | 0.541938067 | 28560 | 465 |
| chr5_73221072_73194986_-26086-Fryl | chr5 | Fryl | 0.202796353 | 0 | 0 | 26087 | 293 |
| chr6_113409340_113408905_+435-Ttll3 | chr6 | Ttll3 | 0 | 0.182309179 | 0.180646022 | 436 | 436 |
| chr6_115644642_115634553_-10089-Raf1 | chr6 | Raf1 | 0 | 0 | 0.180646022 | 10090 | 607 |
| chr6_115644642_115637599_-7043-Raf1 | chr6 | Raf1 | 0.405592706 | 0.182309179 | 0 | 7044 | 346 |
| chr6_116043805_116042871_-934-Tmcc1 | chr6 | Tmcc1 | 0.811185411 | 0 | 0.722584089 | 935 | 935 |
| chr6_119921028_119920110_+918-Rad52 | chr6 | Rad52 | 0.405592706 | 0.546927538 | 0.722584089 | 919 | 303 |
| chr6_124705656_124705158_-498-Emg1 | chr6 | Emg1 | 0.101398176 | 0 | 0.180646022 | 499 | 201 |
| chr6_134066465_134062064_+4401-Etv6 | chr6 | Etv6 | 0.304194529 | 0 | 0 | 4402 | 4402 |
| chr6_134513419_134506196_-7223-Lrp6 | chr6 | Lrp6 | 1.41957447 | 0 | 0.180646022 | 7224 | 898 |
| chr6_17855028_17834617_+20411-St7 | chr6 | St7 | 0.202796353 | 0 | 0 | 20412 | 729 |
| chr6_31433131_31418931_+14200-Mkln1 | chr6 | Mkln1 | 1.216778117 | 1.640782614 | 1.083876134 | 14201 | 608 |
| chr6_31451584_31418931_+32653-Mkln1 | chr6 | Mkln1 | 0 | 0.364618359 | 0.361292045 | 32654 | 752 |
| chr6_37364143_37353557_-10586-Creb3l2 | chr6 | Creb3l2 | 0 | 0 | 0.180646022 | 10587 | 724 |
| chr6_38819313_38818230_-1083-Hipk2 | chr6 | Hipk2 | 0 | 0 | 0.180646022 | 1084 | 1084 |
| chr6_47577667_47540677_-36990-Ezh2 | chr6 | Ezh2 | 2.230759881 | 2.005400973 | 3.974212491 | 36991 | 1940 |
| chr6_47577667_47576536_-1131-Ezh2 | chr6 | Ezh2 | 0 | 0 | 0.180646022 | 1132 | 253 |
| chr6_65901859_65862915_+38944-Prdm5 | chr6 | Prdm5 | 0.811185411 | 0.364618359 | 0 | 38945 | 758 |
| chr6_65901859_65869181_+32678-Prdm5 | chr6 | Prdm5 | 0.811185411 | 0 | 0 | 32679 | 678 |
| chr6_72132163_72128264_+3899-St3gal5 | chr6 | St3gal5 | 0 | 0.364618359 | 0 | 3900 | 236 |
| chr6_88358642_88355483_-3159-Eefsec | chr6 | Eefsec | 0.202796353 | 1.458473435 | 0.722584089 | 3160 | 262 |
| chr6_92164293_92138987_+25306-Nr2c2 | chr6 | Nr2c2 | 0 | 0.182309179 | 0 | 25307 | 1749 |
| chr6_99435345_99429210_-6135-Foxp1 | chr6 | Foxp1 | 0.202796353 | 0 | 0 | 6136 | 6136 |
| chr6_99456580_99435216_-21364-Foxp1 | chr6 | Foxp1 | 0.405592706 | 0 | 0 | 21365 | 21365 |
| chr7_112759639_112759384_+255-Tead1 | chr7 | Tead1 | 0 | 0 | 0.361292045 | 256 | 256 |
| chr7_121057399_121047838_+9561-Mettl9 | chr7 | Mettl9 | 0.811185411 | 0.546927538 | 0 | 9562 | 586 |
| chr7_121057399_121052087_+5312-Mettl9 | chr7 | Mettl9 | 0.202796353 | 0.182309179 | 0 | 5313 | 395 |
| chr7_125770703_125760301_+10402-D430042O09Rik | chr7 | D430042O09Rik | 0 | 0 | 0.180646022 | 10403 | 400 |
| chr7_126552338_126551975_-363-Eif3c | chr7 | Eif3c | 0.202796353 | 0.182309179 | 0 | 364 | 364 |
| chr7_132779385_132759389_-19996-Fam53b | chr7 | Fam53b | 0.202796353 | 0.546927538 | 0.180646022 | 19997 | 1081 |
| chr7_132950435_132948044_+2391-Zranb1 | chr7 | Zranb1 | 1.013981764 | 3.828492767 | 2.167752268 | 2392 | 1016 |
| chr7_132966694_132949585_+17109-Zranb1 | chr7 | Zranb1 | 0 | 0 | 0.180646022 | 17110 | 1039 |
| chr7_132973623_132948044_+25579-Zranb1 | chr7 | Zranb1 | 0.405592706 | 0 | 0 | 25580 | 1629 |
| chr7_132973623_132949585_+24038-Zranb1 | chr7 | Zranb1 | 0.608389058 | 0 | 0.361292045 | 24039 | 1464 |
| chr7_132973623_132966507_+7116-Zranb1 | chr7 | Zranb1 | 0.405592706 | 0.546927538 | 0.361292045 | 7117 | 613 |
| chr7_132973623_132971186_+2437-Zranb1 | chr7 | Zranb1 | 0 | 0.182309179 | 0 | 2438 | 425 |
| chr7_134876315_134874058_+2257-Dock1 | chr7 | Dock1 | 0 | 0 | 0.361292045 | 2258 | 224 |
| chr7_143527311_143524161_-3150-Nap1l4 | chr7 | Nap1l4 | 0 | 0.364618359 | 0 | 3151 | 289 |
| chr7_34245978_34244966_-1012-4931406P16Rik | chr7 | 4931406P16Rik | 0 | 0.182309179 | 0 | 1013 | 1013 |
| chr7_44916470_44915841_-629-Ap2a1 | chr7 | Ap2a1 | 1.41957447 | 0.182309179 | 0 | 630 | 406 |
| chr7_49806626_49786067_+20559-Prmt3 | chr7 | Prmt3 | 0.202796353 | 0.182309179 | 0 | 20560 | 590 |
| chr7_51897372_51887912_+9460-Gas2 | chr7 | Gas2 | 0 | 0 | 0.180646022 | 9461 | 287 |
| chr7_59247217_59240967_+6250-Ube3a | chr7 | Ube3a | 0 | 1.093855076 | 0 | 6251 | 232 |
| chr7_68004355_68003810_+545-Igf1r | chr7 | Igf1r | 0 | 0.182309179 | 0 | 546 | 546 |
| chr7_84634409_84632656_-1753-Zfand6 | chr7 | Zfand6 | 0.202796353 | 0.182309179 | 0 | 1754 | 381 |
| chr7_97664766_97660813_+3953-Rsf1 | chr7 | Rsf1 | 0.608389058 | 1.276164256 | 1.264522156 | 3954 | 1976 |
| chr7_99955540_99935162_-20378-Rnf169 | chr7 | Rnf169 | 0.811185411 | 1.640782614 | 1.445168179 | 20379 | 340 |
| chr8_108800691_108792202_+8489-Zfhx3 | chr8 | Zfhx3 | 0 | 0.182309179 | 0 | 8490 | 3265 |
| chr8_108856953_108792202_+64751-Zfhx3 | chr8 | Zfhx3 | 0.202796353 | 0 | 0 | 64752 | 3497 |
| chr8_120553802_120553584_+218-Gse1 | chr8 | Gse1 | 1.216778117 | 1.823091794 | 0 | 219 | 219 |
| chr8_123383443_123382527_+916-Tcf25 | chr8 | Tcf25 | 0.608389058 | 0 | 0.180646022 | 917 | 194 |
| chr8_127426753_127415570_+11183-Pard3 | chr8 | Pard3 | 0 | 0 | 0.361292045 | 11184 | 460 |
| chr8_13142598_13136796_+5802-Cul4a | chr8 | Cul4a | 0.405592706 | 0 | 0 | 5803 | 328 |
| chr8_24982122_24978428_-3694-Adam9 | chr8 | Adam9 | 0.405592706 | 0 | 0.180646022 | 3695 | 461 |
| chr8_36938758_36937560_-1198-Dlc1 | chr8 | Dlc1 | 0 | 0 | 0.180646022 | 1199 | 1199 |
| chr8_46818908_46793493_+25415-Irf2 | chr8 | Irf2 | 0.202796353 | 0 | 0 | 25416 | 535 |
| chr8_47900856_47899436_-1420-Wwc2 | chr8 | Wwc2 | 0 | 0 | 0.180646022 | 1421 | 281 |
| chr8_67720953_67713683_-7270-Psd3 | chr8 | Psd3 | 0 | 0.182309179 | 0 | 7271 | 447 |
| chr8_67908889_67882981_-25908-Psd3 | chr8 | Psd3 | 0 | 0.182309179 | 0 | 25909 | 553 |
| chr8_67908889_67904117_-4772-Psd3 | chr8 | Psd3 | 0 | 0 | 0.541938067 | 4773 | 460 |
| chr8_67964354_67960387_-3967-Psd3 | chr8 | Psd3 | 0.608389058 | 0 | 0 | 3968 | 1459 |
| chr8_72380306_72367904_-12402-Eps15l1 | chr8 | Eps15l1 | 0 | 0 | 0.180646022 | 12403 | 4640 |
| chr8_77365160_77310731_-54429-Arhgap10 | chr8 | Arhgap10 | 0.202796353 | 0 | 0 | 54430 | 639 |
| chr8_77365160_77344640_-20520-Arhgap10 | chr8 | Arhgap10 | 2.636352587 | 3.281565229 | 4.877442603 | 20521 | 488 |
| chr8_77450923_77450693_-230-Arhgap10 | chr8 | Arhgap10 | 0.405592706 | 0.182309179 | 0 | 231 | 158 |
| chr8_79136663_79091833_+44830-Zfp827 | chr8 | Zfp827 | 0 | 0 | 0.180646022 | 44831 | 636 |
| chr8_79136663_79118175_+18488-Zfp827 | chr8 | Zfp827 | 0.405592706 | 0.546927538 | 0.180646022 | 18489 | 402 |
| chr8_79372364_79371784_-580-Smad1 | chr8 | Smad1 | 0.405592706 | 0.364618359 | 0 | 581 | 581 |
| chr8_80800395_80800101_-294-Gab1 | chr8 | Gab1 | 0.405592706 | 0.182309179 | 0.180646022 | 295 | 295 |
| chr8_84772315_84771784_-531-Nfix | chr8 | Nfix | 9.734224936 | 6.563130458 | 3.974212491 | 532 | 532 |
| chr8_84864552_84864058_+494-Farsa | chr8 | Farsa | 0.405592706 | 0 | 0 | 495 | 338 |
| chr8_85901957_85875634_+26323-Phkb | chr8 | Phkb | 0.608389058 | 0.182309179 | 0 | 26324 | 518 |
| chr8_85922242_85875634_+46608-Phkb | chr8 | Phkb | 0.405592706 | 0.546927538 | 0.541938067 | 46609 | 634 |
| chr8_85970997_85935408_+35589-Phkb | chr8 | Phkb | 0.608389058 | 1.823091794 | 0.361292045 | 35590 | 748 |
| chr8_86862110_86860426_-1684-N4bp1 | chr8 | N4bp1 | 0.202796353 | 0 | 0 | 1685 | 1685 |
| chr8_88723212_88702305_+20907-Cyld | chr8 | Cyld | 0.202796353 | 0.182309179 | 0 | 20908 | 1419 |
| chr8_90933862_90932249_+1613-Chd9 | chr8 | Chd9 | 0.405592706 | 0.364618359 | 0 | 1614 | 1614 |
| chr8_95366422_95365314_+1108-Mmp15 | chr8 | Mmp15 | 0.202796353 | 0 | 0.361292045 | 1109 | 278 |
| chr9_108534618_108533587_+1031-Qrich1 | chr9 | Qrich1 | 0.405592706 | 0 | 0.180646022 | 1032 | 1032 |
| chr9_116131713_116129816_-1897-Tgfbr2 | chr9 | Tgfbr2 | 0 | 0.546927538 | 0 | 1898 | 360 |
| chr9_120959529_120950173_+9356-Ctnnb1 | chr9 | Ctnnb1 | 0 | 0.182309179 | 0.180646022 | 9357 | 2406 |
| chr9_121731565_121727316_+4249-Nktr | chr9 | Nktr | 0 | 0.09115459 | 0 | 4250 | 316 |
| chr9_122857161_122856699_-462-Zfp445 | chr9 | Zfp445 | 0.405592706 | 0 | 0.541938067 | 463 | 222 |
| chr9_14575439_14571623_-3816-Amotl1 | chr9 | Amotl1 | 2.230759881 | 0.546927538 | 0.722584089 | 3817 | 524 |
| chr9_16378254_16374949_-3305-Fat3 | chr9 | Fat3 | 0 | 1.093855076 | 0.722584089 | 3306 | 3306 |
| chr9_21575518_21569455_+6063-Carm1 | chr9 | Carm1 | 0 | 0.182309179 | 0 | 6064 | 338 |
| chr9_22514093_22490818_+23275-Bbs9 | chr9 | Bbs9 | 0.405592706 | 0 | 0 | 23276 | 453 |
| chr9_22679064_22643745_+35319-Bbs9 | chr9 | Bbs9 | 1.013981764 | 0.182309179 | 0 | 35320 | 786 |
| chr9_22887996_22887581_+415-Bbs9 | chr9 | Bbs9 | 2.027963528 | 0 | 0.903230112 | 416 | 243 |
| chr9_24485729_24462577_-23152-Dpy19l1 | chr9 | Dpy19l1 | 0.202796353 | 0 | 0 | 23153 | 524 |
| chr9_32208265_32153040_+55225-Arhgap32 | chr9 | Arhgap32 | 0.202796353 | 0 | 0 | 55226 | 686 |
| chr9_42443075_42437115_-5960-Tbcel | chr9 | Tbcel | 0.202796353 | 0.182309179 | 0 | 5961 | 501 |
| chr9_42451762_42437115_-14647-Tbcel | chr9 | Tbcel | 0.405592706 | 1.093855076 | 0 | 14648 | 973 |
| chr9_45978729_45972942_-5787-Pafah1b2 | chr9 | Pafah1b2 | 0 | 0.182309179 | 0 | 5788 | 418 |
| chr9_56147578_56145489_-2089-Tspan3 | chr9 | Tspan3 | 1.216778117 | 0 | 0.180646022 | 2090 | 606 |
| chr9_62070613_62068385_-2228-Glce | chr9 | Glce | 0.202796353 | 0 | 0 | 2229 | 845 |
| chr9_63619231_63616686_+2545-Aagab | chr9 | Aagab | 0.608389058 | 0 | 0 | 2546 | 462 |
| chr9_64214609_64204431_-10178-Map2k1 | chr9 | Map2k1 | 0.202796353 | 0 | 0.722584089 | 10179 | 488 |
| chr9_65795495_65794622_-873-Zfp609 | chr9 | Zfp609 | 0.608389058 | 0 | 0 | 874 | 874 |
| chr9_66566903_66540062_-26841-Usp3 | chr9 | Usp3 | 0.405592706 | 0.364618359 | 0 | 26842 | 670 |
| chr9_72726530_72725017_+1513-Nedd4 | chr9 | Nedd4 | 0.202796353 | 0 | 0 | 1514 | 186 |
| chr9_80093641_80087430_+6211-Senp6 | chr9 | Senp6 | 0 | 0 | 0.361292045 | 6212 | 403 |
| chr9_80103764_80087430_+16334-Senp6 | chr9 | Senp6 | 0.202796353 | 0 | 0 | 16335 | 495 |
| chr9_82959851_82945758_-14093-Phip | chr9 | Phip | 0.405592706 | 0 | 0 | 14094 | 411 |
| chr9_84012619_83988776_+23843-Bckdhb | chr9 | Bckdhb | 0.202796353 | 0 | 0 | 23844 | 608 |
| chr9_8658377_8634047_+24330-Trpc6 | chr9 | Trpc6 | 0.405592706 | 0.182309179 | 0 | 24331 | 1356 |
| chr9_96611499_96591950_-19549-Rasa2 | chr9 | Rasa2 | 0 | 0.729236718 | 0 | 19550 | 394 |
| chrX_142834393_142752040_+82353-Tmem164 | chrX | Tmem164 | 0 | 0 | 0.180646022 | 82354 | 506 |
| chrX_151399803_151376748_+23055-Fam120c | chrX | Fam120c | 0.811185411 | 0 | 0 | 23056 | 459 |
| chrX_151843155_151835224_+7931-Huwe1 | chrX | Huwe1 | 0 | 0 | 0.180646022 | 7932 | 636 |
| chrX_47887198_47811158_-76040-Smarca1 | chrX | Smarca1 | 0 | 0.182309179 | 0.180646022 | 76041 | 2946 |
| chr1_10116731_10082253_+34478-Cspp1 | chr1 | Cspp1 | 0 | 0 | 0.180646022 | 34479 | 3436 |
| chr1_105696757_105691906_+4851-2310035C23Rik | chr1 | 2310035C23Rik | 0.202796353 | 0 | 0 | 4852 | 786 |
| chr1_106019905_106011702_+8203-Zcchc2 | chr1 | Zcchc2 | 0 | 0.364618359 | 0.361292045 | 8204 | 350 |
| chr1_106031233_106000931_+30302-Zcchc2 | chr1 | Zcchc2 | 0.202796353 | 0 | 0 | 30303 | 2530 |
| chr1_11142797_11123121_+19676-Prex2 | chr1 | Prex2 | 0 | 0.182309179 | 0 | 19677 | 688 |
| chr1_11142797_11139956_+2841-Prex2 | chr1 | Prex2 | 0 | 0.182309179 | 0 | 2842 | 385 |
| chr1_11266159_11162240_+103919-Prex2 | chr1 | Prex2 | 0.202796353 | 0 | 0 | 103920 | 2108 |
| chr1_118309866_118304691_-5175-Tsn | chr1 | Tsn | 0.202796353 | 0 | 0 | 5176 | 387 |
| chr1_118581463_118570849_+10614-Clasp1 | chr1 | Clasp1 | 0.202796353 | 0 | 0 | 10615 | 586 |
| chr1_118590549_118521827_+68722-Clasp1 | chr1 | Clasp1 | 0 | 0 | 0.361292045 | 68723 | 2477 |
| chr1_118590549_118539266_+51283-Clasp1 | chr1 | Clasp1 | 0 | 0 | 0.180646022 | 51284 | 2012 |
| chr1_121312325_121172433_-139892-Gm41940 | chr1 | Gm41940 | 0 | 0.182309179 | 0 | 139893 | 139893 |
| chr1_127803293_127791605_+11688-Ccnt2 | chr1 | Ccnt2 | 0.202796353 | 0 | 0 | 11689 | 1666 |
| chr1_127803659_127791605_+12054-Ccnt2 | chr1 | Ccnt2 | 0.202796353 | 0.546927538 | 0.903230112 | 12055 | 2032 |
| chr1_127803659_127797841_+5818-Ccnt2 | chr1 | Ccnt2 | 0 | 0.364618359 | 0 | 5819 | 1779 |
| chr1_128211359_128190638_+20721-R3hdm1 | chr1 | R3hdm1 | 0.405592706 | 0.546927538 | 0.361292045 | 20722 | 678 |
| chr1_128211359_128193460_+17899-R3hdm1 | chr1 | R3hdm1 | 0.202796353 | 0 | 0 | 17900 | 576 |
| chr1_12822099_12786079_+36020-Sulf1 | chr1 | Sulf1 | 0 | 0 | 0.180646022 | 36021 | 1509 |
| chr1_128221703_128190638_+31065-R3hdm1 | chr1 | R3hdm1 | 0 | 0.182309179 | 0 | 31066 | 934 |
| chr1_128235626_128190638_+44988-R3hdm1 | chr1 | R3hdm1 | 0 | 0.364618359 | 0 | 44989 | 1492 |
| chr1_128262957_128252203_+10754-Ubxn4 | chr1 | Ubxn4 | 0 | 0 | 0.361292045 | 10755 | 740 |
| chr1_12939200_12894427_-44773-Slco5a1 | chr1 | Slco5a1 | 0 | 0 | 0.180646022 | 44774 | 576 |
| chr1_130684070_130682149_-1921-na | chr1 | na | 0.608389058 | 0 | 0.180646022 | 1922 | 1922 |
| chr1_13167106_13156370_-10736-Ncoa2 | chr1 | Ncoa2 | 0 | 0.182309179 | 0 | 10737 | 945 |
| chr1_132019466_132014383_+5083-Elk4 | chr1 | Elk4 | 0 | 0.182309179 | 0 | 5084 | 1203 |
| chr1_132023838_132019350_+4488-Elk4 | chr1 | Elk4 | 0 | 0 | 0.361292045 | 4489 | 403 |
| chr1_132391394_132391096_-298-na | chr1 | na | 0 | 0 | 0.180646022 | 299 | 299 |
| chr1_132434487_132434099_+388-Dstyk | chr1 | Dstyk | 0 | 0.182309179 | 0 | 389 | 389 |
| chr1_133645846_133637349_-8497-Zc3h11a | chr1 | Zc3h11a | 0 | 0.182309179 | 0 | 8498 | 757 |
| chr1_133647529_133633762_-13767-Zc3h11a | chr1 | Zc3h11a | 0 | 0.182309179 | 0.180646022 | 13768 | 1110 |
| chr1_134842733_134834435_-8298-Ppp1r12b | chr1 | Ppp1r12b | 0.608389058 | 0.182309179 | 0.903230112 | 8299 | 625 |
| chr1_134879745_134835785_-43960-Ppp1r12b | chr1 | Ppp1r12b | 0 | 0 | 0.090323011 | 43961 | 1472 |
| chr1_134879745_134865778_-13967-Ppp1r12b | chr1 | Ppp1r12b | 0.202796353 | 0.182309179 | 0.361292045 | 13968 | 932 |
| chr1_135607904_135602545_-5359-Nav1 | chr1 | Nav1 | 0.202796353 | 0 | 0 | 5360 | 847 |
| chr1_13576452_13569471_-6981-Tram1 | chr1 | Tram1 | 0.202796353 | 0 | 0 | 6982 | 566 |
| chr1_136105576_136100600_+4976-Cacna1s | chr1 | Cacna1s | 0 | 0.182309179 | 0 | 4977 | 381 |
| chr1_138561785_138515625_-46160-Nek7 | chr1 | Nek7 | 0 | 0 | 0.361292045 | 46161 | 615 |
| chr1_139042043_139040000_+2043-Dennd1b | chr1 | Dennd1b | 0 | 0 | 0.361292045 | 2044 | 170 |
| chr1_139110485_139060275_+50210-Dennd1b | chr1 | Dennd1b | 0 | 0.182309179 | 0 | 50211 | 642 |
| chr1_139110485_139081173_+29312-Dennd1b | chr1 | Dennd1b | 0.405592706 | 0.182309179 | 0 | 29313 | 477 |
| chr1_139110485_139085883_+24602-Dennd1b | chr1 | Dennd1b | 0.202796353 | 0 | 0 | 24603 | 376 |
| chr1_139144000_139053346_+90654-Dennd1b | chr1 | Dennd1b | 0 | 0.182309179 | 0 | 90655 | 1219 |
| chr1_14253102_14208895_-44207-Eya1 | chr1 | Eya1 | 0.608389058 | 0.182309179 | 0 | 44208 | 584 |
| chr1_14253102_14229493_-23609-Eya1 | chr1 | Eya1 | 0 | 0.729236718 | 0 | 23610 | 494 |
| chr1_143603858_143603741_+117-na | chr1 | na | 0.202796353 | 0 | 0 | 118 | 118 |
| chr1_151442710_151440806_+1904-Trmt1l | chr1 | Trmt1l | 0 | 0.364618359 | 0 | 1905 | 254 |
| chr1_151443326_151439516_+3810-Trmt1l | chr1 | Trmt1l | 0 | 0.182309179 | 0 | 3811 | 399 |
| chr1_152859502_152855849_-3653-Smg7 | chr1 | Smg7 | 0 | 0.182309179 | 0 | 3654 | 604 |
| chr1_152866713_152840035_-26678-Smg7 | chr1 | Smg7 | 0.202796353 | 0 | 0 | 26679 | 3262 |
| chr1_155191554_155173986_+17568-Stx6 | chr1 | Stx6 | 0.202796353 | 0 | 0 | 17569 | 454 |
| chr1_155624958_155615867_+9091-Acbd6 | chr1 | Acbd6 | 0 | 0 | 0.180646022 | 9092 | 196 |
| chr1_155848727_155847110_-1617-na | chr1 | na | 0.202796353 | 0 | 0 | 1618 | 1618 |
| chr1_155914879_155911888_-2991-Cep350 | chr1 | Cep350 | 0 | 0 | 0.180646022 | 2992 | 275 |
| chr1_160702460_160682041_-20419-Rabgap1l | chr1 | Rabgap1l | 0 | 0.182309179 | 0 | 20420 | 412 |
| chr1_160724189_160627215_-96974-Rabgap1l | chr1 | Rabgap1l | 0 | 0.364618359 | 0 | 96975 | 993 |
| chr1_160724189_160682041_-42148-Rabgap1l | chr1 | Rabgap1l | 0.202796353 | 0 | 0 | 42149 | 748 |
| chr1_160739088_160733529_-5559-Rabgap1l | chr1 | Rabgap1l | 0 | 0.182309179 | 0 | 5560 | 579 |
| chr1_160740931_160627215_-113716-Rabgap1l | chr1 | Rabgap1l | 0.202796353 | 0 | 0 | 113717 | 1752 |
| chr1_160740931_160710147_-30784-Rabgap1l | chr1 | Rabgap1l | 0 | 0 | 0.180646022 | 30785 | 1095 |
| chr1_163262015_163253977_-8038-Prrx1 | chr1 | Prrx1 | 0 | 0.182309179 | 0 | 8039 | 430 |
| chr1_163262015_163257761_-4254-Prrx1 | chr1 | Prrx1 | 0.202796353 | 0.546927538 | 0 | 4255 | 358 |
| chr1_163829295_163825688_+3607-Kifap3 | chr1 | Kifap3 | 0 | 0.182309179 | 0 | 3608 | 342 |
| chr1_16440458_16389821_-50637-Stau2 | chr1 | Stau2 | 0.202796353 | 0 | 0.180646022 | 50638 | 617 |
| chr1_165228447_165220099_-8348-Tiprl | chr1 | Tiprl | 0 | 0.182309179 | 0 | 8349 | 508 |
| chr1_165352076_165350884_-1192-Dcaf6 | chr1 | Dcaf6 | 0.608389058 | 0 | 0.180646022 | 1193 | 582 |
| chr1_165357546_165350884_-6662-Dcaf6 | chr1 | Dcaf6 | 0.202796353 | 0.729236718 | 0 | 6663 | 788 |
| chr1_165367505_165357429_-10076-Dcaf6 | chr1 | Dcaf6 | 0.202796353 | 0.09115459 | 0 | 10077 | 178 |
| chr1_165371248_165350884_-20364-Dcaf6 | chr1 | Dcaf6 | 0 | 0.182309179 | 0 | 20365 | 20365 |
| chr1_165374235_165367446_-6789-Dcaf6 | chr1 | Dcaf6 | 0.405592706 | 0 | 0 | 6790 | 234 |
| chr1_165392232_165367446_-24786-na | chr1 | na | 0.101398176 | 0 | 0 | 24787 | 24787 |
| chr1_165392232_165388658_-3574-Dcaf6 | chr1 | Dcaf6 | 0 | 0.546927538 | 0.180646022 | 3575 | 472 |
| chr1_165399868_165388658_-11210-Dcaf6 | chr1 | Dcaf6 | 0 | 0.364618359 | 0 | 11211 | 687 |
| chr1_165411599_165388658_-22941-Dcaf6 | chr1 | Dcaf6 | 0 | 0.546927538 | 0.541938067 | 22942 | 823 |
| chr1_165424394_165411464_-12930-Dcaf6 | chr1 | Dcaf6 | 0 | 0 | 0.180646022 | 12931 | 529 |
| chr1_165429315_165411464_-17851-Dcaf6 | chr1 | Dcaf6 | 0 | 0 | 0.361292045 | 17852 | 591 |
| chr1_165608604_165601750_-6854-Mpzl1 | chr1 | Mpzl1 | 0.202796353 | 0 | 0 | 6855 | 620 |
| chr1_16585431_16345704_-239727-Stau2 | chr1 | Stau2 | 0 | 0.182309179 | 0 | 239728 | 239728 |
| chr1_16585431_16581927_-3504-Ube2w | chr1 | Ube2w | 0 | 0 | 0.361292045 | 3505 | 232 |
| chr1_165931727_165875382_-56345-Pou2f1 | chr1 | Pou2f1 | 0.202796353 | 0 | 0 | 56346 | 2677 |
| chr1_165931727_165916311_-15416-Pou2f1 | chr1 | Pou2f1 | 0 | 0.182309179 | 0 | 15417 | 15417 |
| chr1_17016157_17016039_-118-Jph1 | chr1 | Jph1 | 0 | 0 | 0.722584089 | 119 | 119 |
| chr1_17017651_16997464_-20187-Jph1 | chr1 | Jph1 | 0 | 0.182309179 | 0 | 20188 | 20188 |
| chr1_170779989_170779446_-543-na | chr1 | na | 0.405592706 | 0 | 0 | 544 | 544 |
| chr1_170794770_170787346_-7424-Atf6 | chr1 | Atf6 | 0 | 0.364618359 | 0 | 7425 | 397 |
| chr1_170799745_170787346_-12399-Atf6 | chr1 | Atf6 | 0.405592706 | 0 | 0 | 12400 | 529 |
| chr1_170841812_170834820_-6992-Atf6 | chr1 | Atf6 | 0 | 0.364618359 | 0 | 6993 | 552 |
| chr1_17092057_16996795_-95262-Jph1 | chr1 | Jph1 | 0.202796353 | 0 | 0 | 95263 | 2288 |
| chr1_17092057_16997464_-94593-Jph1 | chr1 | Jph1 | 0.202796353 | 0 | 0.180646022 | 94594 | 1619 |
| chr1_17092057_17016039_-76018-Jph1 | chr1 | Jph1 | 0.202796353 | 0 | 0.090323011 | 76019 | 879 |
| chr1_17092057_17054066_-37991-Jph1 | chr1 | Jph1 | 0.202796353 | 0 | 0 | 37992 | 37992 |
| chr1_17092057_17091298_-759-Jph1 | chr1 | Jph1 | 1.216778117 | 1.093855076 | 0.180646022 | 760 | 760 |
| chr1_171138755_171135993_-2762-na | chr1 | na | 0.101398176 | 0 | 0 | 2763 | 2763 |
| chr1_17158503_17144109_+14394-Gdap1 | chr1 | Gdap1 | 0.202796353 | 0 | 0 | 14395 | 14395 |
| chr1_17163664_17161720_+1944-na | chr1 | na | 0 | 0.182309179 | 0.180646022 | 1945 | 1945 |
| chr1_172099767_172092245_+7522-Copa | chr1 | Copa | 0.202796353 | 0.182309179 | 0 | 7523 | 320 |
| chr1_172187460_172173943_+13517-Dcaf8 | chr1 | Dcaf8 | 1.825167175 | 0.546927538 | 2.167752268 | 13518 | 586 |
| chr1_172278387_172274569_-3818-Atp1a2 | chr1 | Atp1a2 | 0.202796353 | 0 | 0 | 3819 | 486 |
| chr1_176774910_176755166_-19744-Cep170 | chr1 | Cep170 | 0.405592706 | 0 | 0 | 19745 | 3204 |
| chr1_176831182_176824752_+6430-Sdccag8 | chr1 | Sdccag8 | 0 | 0 | 0.180646022 | 6431 | 479 |
| chr1_177131096_177096967_-34129-Akt3 | chr1 | Akt3 | 0.202796353 | 0 | 0 | 34130 | 515 |
| chr1_177248881_177078774_-170107-Akt3 | chr1 | Akt3 | 0 | 0.182309179 | 0 | 170108 | 856 |
| chr1_177430211_177428205_-2006-na | chr1 | na | 0 | 0 | 0.180646022 | 2007 | 2007 |
| chr1_177776879_177771141_-5738-Adss | chr1 | Adss | 0 | 0 | 0.361292045 | 5739 | 664 |
| chr1_178925298_178913704_-11594-na | chr1 | na | 0.202796353 | 0 | 0 | 11595 | 11595 |
| chr1_179094402_179043737_-50665-Smyd3 | chr1 | Smyd3 | 0 | 0.182309179 | 0 | 50666 | 545 |
| chr1_179233324_179229110_-4214-na | chr1 | na | 0.202796353 | 0 | 0 | 4215 | 4215 |
| chr1_179423418_179405283_-18135-Smyd3 | chr1 | Smyd3 | 0.811185411 | 0 | 0.361292045 | 18136 | 367 |
| chr1_179494864_179405283_-89581-Smyd3 | chr1 | Smyd3 | 0.101398176 | 0 | 0 | 89582 | 89582 |
| chr1_180179284_180178577_-707-Adck3 | chr1 | Adck3 | 0.202796353 | 0 | 0 | 708 | 469 |
| chr1_181930762_181917199_-13563-Enah | chr1 | Enah | 0 | 0.182309179 | 0 | 13564 | 1767 |
| chr1_184834028_184832576_-1452-Marc2 | chr1 | Marc2 | 0 | 0 | 0.180646022 | 1453 | 310 |
| chr1_185267279_185266901_+378-Rab3gap2 | chr1 | Rab3gap2 | 0.202796353 | 1.093855076 | 0 | 379 | 281 |
| chr1_185387118_185369577_+17541-Eprs | chr1 | Eprs | 0 | 0 | 0.180646022 | 17542 | 1363 |
| chr1_185413550_185386246_+27304-Eprs | chr1 | Eprs | 0.202796353 | 0 | 0 | 27305 | 2227 |
| chr1_187322354_187316524_+5830-Gpatch2 | chr1 | Gpatch2 | 0.202796353 | 0 | 0 | 5831 | 111 |
| chr1_188066457_188043334_+23123-Esrrg | chr1 | Esrrg | 0 | 0.364618359 | 0 | 23124 | 533 |
| chr1_189798681_189779506_+19175-Ptpn14 | chr1 | Ptpn14 | 0 | 0.364618359 | 0.361292045 | 19176 | 19176 |
| chr1_189860586_189839472_+21114-Ptpn14 | chr1 | Ptpn14 | 0.405592706 | 0 | 0 | 21115 | 2284 |
| chr1_189882908_189882535_+373-na | chr1 | na | 0.202796353 | 0 | 0 | 374 | 374 |
| chr1_189909914_189896571_-13343-Smyd2 | chr1 | Smyd2 | 0 | 0.182309179 | 0 | 13344 | 361 |
| chr1_190162313_190153416_-8897-Prox1 | chr1 | Prox1 | 0 | 0.182309179 | 0 | 8898 | 1900 |
| chr1_190844602_190782535_-62067-Rps6kc1 | chr1 | Rps6kc1 | 0.202796353 | 0 | 0 | 62068 | 2228 |
| chr1_190885427_190884560_-867-Rps6kc1 | chr1 | Rps6kc1 | 0 | 0.182309179 | 0 | 868 | 868 |
| chr1_190888764_190871592_-17172-Rps6kc1 | chr1 | Rps6kc1 | 0.202796353 | 0 | 0 | 17173 | 570 |
| chr1_191012242_191009549_-2693-Mfsd7b | chr1 | Mfsd7b | 0 | 0 | 0.180646022 | 2694 | 436 |
| chr1_191012242_191011536_-706-Mfsd7b | chr1 | Mfsd7b | 0 | 0.182309179 | 0 | 707 | 215 |
| chr1_191021256_191012139_-9117-Mfsd7b | chr1 | Mfsd7b | 0.405592706 | 0 | 0 | 9118 | 621 |
| chr1_191021256_191015358_-5898-Mfsd7b | chr1 | Mfsd7b | 0 | 0 | 0.180646022 | 5899 | 449 |
| chr1_192126027_192123539_-2488-Rcor3 | chr1 | Rcor3 | 0.202796353 | 0.182309179 | 0.361292045 | 2489 | 366 |
| chr1_195024406_195018548_+5858-A330023F24Rik | chr1 | A330023F24Rik | 0 | 0 | 0.180646022 | 5859 | 5859 |
| chr1_195077836_195064688_-13148-Cd46 | chr1 | Cd46 | 0 | 0.182309179 | 0 | 13149 | 1355 |
| chr1_21593614_21457404_-136210-Kcnq5 | chr1 | Kcnq5 | 0.405592706 | 0 | 0 | 136211 | 1127 |
| chr1_21678782_21424049_-254733-Kcnq5 | chr1 | Kcnq5 | 0 | 0.182309179 | 0 | 254734 | 254734 |
| chr1_24537002_24529928_-7074-Col19a1 | chr1 | Col19a1 | 0 | 0.182309179 | 0 | 7075 | 481 |
| chr1_24575459_24559654_-15805-Col19a1 | chr1 | Col19a1 | 0 | 0 | 0.180646022 | 15806 | 293 |
| chr1_24731899_24685397_+46502-Lmbrd1 | chr1 | Lmbrd1 | 0.202796353 | 0 | 0 | 46503 | 846 |
| chr1_24731899_24711512_+20387-Lmbrd1 | chr1 | Lmbrd1 | 0 | 0 | 0.361292045 | 20388 | 442 |
| chr1_24744358_24705977_+38381-Lmbrd1 | chr1 | Lmbrd1 | 0.202796353 | 0 | 0 | 38382 | 678 |
| chr1_24748878_24711512_+37366-Lmbrd1 | chr1 | Lmbrd1 | 0 | 0.182309179 | 0 | 37367 | 944 |
| chr1_30811967_30810683_-1284-Phf3 | chr1 | Phf3 | 0 | 0 | 0.180646022 | 1285 | 344 |
| chr1_30863255_30829843_-33412-Phf3 | chr1 | Phf3 | 0 | 0 | 0.180646022 | 33413 | 2148 |
| chr1_33786532_33779044_-7488-Zfp451 | chr1 | Zfp451 | 0 | 0 | 0.361292045 | 7489 | 818 |
| chr1_34135256_34086242_+49014-Dst | chr1 | Dst | 0 | 0 | 0.180646022 | 49015 | 526 |
| chr1_34135256_34113933_+21323-Dst | chr1 | Dst | 0 | 0 | 0.180646022 | 21324 | 464 |
| chr1_34135256_34114549_+20707-Dst | chr1 | Dst | 0 | 0.182309179 | 0 | 20708 | 374 |
| chr1_34135256_34135090_+166-Dst | chr1 | Dst | 0 | 0.182309179 | 0 | 167 | 167 |
| chr1_34154632_34114549_+40083-Dst | chr1 | Dst | 0 | 0 | 0.180646022 | 40084 | 714 |
| chr1_34196061_34116118_+79943-Dst | chr1 | Dst | 0 | 0.546927538 | 0.361292045 | 79944 | 10446 |
| chr1_34229246_34197201_+32045-Dst | chr1 | Dst | 0 | 0.182309179 | 0 | 32046 | 5082 |
| chr1_34229246_34223804_+5442-Dst | chr1 | Dst | 0.811185411 | 0.546927538 | 0.361292045 | 5443 | 2168 |
| chr1_34294546_34171116_+123430-Dst | chr1 | Dst | 0 | 0.182309179 | 0 | 123431 | 17836 |
| chr1_34295536_34262020_+33516-Dst | chr1 | Dst | 0 | 0 | 0.180646022 | 33517 | 3771 |
| chr1_36197635_36191529_-6106-Uggt1 | chr1 | Uggt1 | 0 | 0.182309179 | 0.180646022 | 6107 | 449 |
| chr1_36197635_36195850_-1785-Uggt1 | chr1 | Uggt1 | 0 | 0 | 1.083876134 | 1786 | 243 |
| chr1_36201765_36173435_-28330-Uggt1 | chr1 | Uggt1 | 0.202796353 | 0 | 0 | 28331 | 1752 |
| chr1_36202878_36184356_-18522-Uggt1 | chr1 | Uggt1 | 0 | 0 | 0.180646022 | 18523 | 878 |
| chr1_36221324_36207942_-13382-Uggt1 | chr1 | Uggt1 | 0 | 0.182309179 | 0 | 13383 | 565 |
| chr1_36823153_36815455_-7698-Tmem131 | chr1 | Tmem131 | 0.608389058 | 0 | 0 | 7699 | 750 |
| chr1_36825346_36816109_-9237-Tmem131 | chr1 | Tmem131 | 0 | 0 | 0.180646022 | 9238 | 813 |
| chr1_37143067_37128884_+14183-Vwa3b | chr1 | Vwa3b | 0.608389058 | 0 | 0 | 14184 | 825 |
| chr1_37811027_37801720_-9307-Tsga10 | chr1 | Tsga10 | 0 | 0 | 0.361292045 | 9308 | 522 |
| chr1_38099021_38079290_-19731-Rev1 | chr1 | Rev1 | 0 | 0.182309179 | 0 | 19732 | 1560 |
| chr1_38099021_38083704_-15317-Rev1 | chr1 | Rev1 | 0.202796353 | 0 | 0 | 15318 | 1251 |
| chr1_39402923_39389308_-13615-Tbc1d8 | chr1 | Tbc1d8 | 0 | 0 | 0.180646022 | 13616 | 969 |
| chr1_40862225_40859923_+2302-na | chr1 | na | 0 | 0 | 0.180646022 | 2303 | 2303 |
| chr1_43075952_43067474_-8478-Tgfbrap1 | chr1 | Tgfbrap1 | 0 | 0 | 0.180646022 | 8479 | 1052 |
| chr1_43111373_43100715_+10658-AI597479 | chr1 | AI597479 | 0.202796353 | 0 | 0 | 10659 | 543 |
| chr1_43533793_43512963_+20830-Nck2 | chr1 | Nck2 | 0.202796353 | 0 | 0 | 20831 | 20831 |
| chr1_43992394_43960084_+32310-Tpp2 | chr1 | Tpp2 | 0 | 0 | 0.361292045 | 32311 | 2355 |
| chr1_44164084_44161810_+2274-Ercc5 | chr1 | Ercc5 | 0 | 0.364618359 | 0 | 2275 | 352 |
| chr1_44167882_44156937_+10945-Ercc5 | chr1 | Ercc5 | 0.202796353 | 0 | 0 | 10946 | 2411 |
| chr1_44708678_44689144_+19534-Gulp1 | chr1 | Gulp1 | 0 | 0.182309179 | 0 | 19535 | 134 |
| chr1_5098133_5089009_+9124-Atp6v1h | chr1 | Atp6v1h | 0 | 0.546927538 | 0.361292045 | 9125 | 412 |
| chr1_5150061_5117390_+32671-Atp6v1h | chr1 | Atp6v1h | 0 | 0 | 0.180646022 | 32672 | 812 |
| chr1_52215694_52188679_-27015-Gls | chr1 | Gls | 0 | 0 | 0.180646022 | 27016 | 1216 |
| chr1_53282092_53216231_-65861-Pms1 | chr1 | Pms1 | 0 | 0 | 0.180646022 | 65862 | 719 |
| chr1_53933288_53885329_-47959-Hecw2 | chr1 | Hecw2 | 0.405592706 | 0 | 0 | 47960 | 2782 |
| chr1_54681191_54397806_-283385-Pgap1 | chr1 | Pgap1 | 0 | 0.182309179 | 0 | 283386 | 283386 |
| chr1_54743428_54723014_-20414-Ankrd44 | chr1 | Ankrd44 | 0.202796353 | 0 | 0.180646022 | 20415 | 719 |
| chr1_54766984_54752890_-14094-Ankrd44 | chr1 | Ankrd44 | 0.202796353 | 0 | 0 | 14095 | 724 |
| chr1_54835257_54723014_-112243-Ankrd44 | chr1 | Ankrd44 | 0 | 0 | 0.180646022 | 112244 | 112244 |
| chr1_54835257_54792453_-42804-Ankrd44 | chr1 | Ankrd44 | 0 | 0 | 0.180646022 | 42805 | 42805 |
| chr1_54835257_54828612_-6645-Ankrd44 | chr1 | Ankrd44 | 0 | 0.182309179 | 0.180646022 | 6646 | 6646 |
| chr1_54835257_54828612_-6645-na | chr1 | na | 0.101398176 | 0 | 0 | 6646 | 6646 |
| chr1_54877821_54828612_-49209-Ankrd44 | chr1 | Ankrd44 | 0 | 0.182309179 | 0 | 49210 | 432 |
| chr1_55016883_55012102_-4781-Sf3b1 | chr1 | Sf3b1 | 0 | 0 | 0.180646022 | 4782 | 1978 |
| chr1_55715603_55692630_+22973-Plcl1 | chr1 | Plcl1 | 0 | 0 | 0.180646022 | 22974 | 22974 |
| chr1_55715603_55695745_+19858-Plcl1 | chr1 | Plcl1 | 0.202796353 | 0 | 0 | 19859 | 2865 |
| chr1_55717475_55701930_+15545-na | chr1 | na | 0 | 0 | 0.090323011 | 15546 | 15546 |
| chr1_55717475_55701930_+15545-Plcl1 | chr1 | Plcl1 | 0.405592706 | 0.182309179 | 0 | 15546 | 15546 |
| chr1_58992375_58976959_+15416-Stradb | chr1 | Stradb | 0 | 0.182309179 | 0 | 15417 | 815 |
| chr1_59199191_59191889_-7302-Als2 | chr1 | Als2 | 0.202796353 | 0.182309179 | 0 | 7303 | 582 |
| chr1_60109449_60105311_+4138-Carf | chr1 | Carf | 0 | 0.364618359 | 0 | 4139 | 470 |
| chr1_60237216_60234797_+2419-Nbeal1 | chr1 | Nbeal1 | 0 | 0 | 0.722584089 | 2420 | 933 |
| chr1_60244883_60222067_+22816-Nbeal1 | chr1 | Nbeal1 | 0 | 0.182309179 | 0 | 22817 | 1998 |
| chr1_60260793_60260233_+560-Nbeal1 | chr1 | Nbeal1 | 0.202796353 | 0 | 0 | 561 | 561 |
| chr1_60499623_60493538_-6085-Raph1 | chr1 | Raph1 | 0 | 0 | 0.180646022 | 6086 | 618 |
| chr1_62211704_62198577_+13127-Pard3b | chr1 | Pard3b | 0 | 0 | 0.361292045 | 13128 | 357 |
| chr1_62350553_62343934_+6619-Pard3b | chr1 | Pard3b | 0.405592706 | 0 | 0 | 6620 | 6620 |
| chr1_63086398_63085668_-730-Ino80d | chr1 | Ino80d | 0.202796353 | 0 | 0 | 731 | 731 |
| chr1_63165030_63151551_-13479-Ndufs1 | chr1 | Ndufs1 | 0 | 0 | 0.180646022 | 13480 | 1215 |
| chr1_64079334_64078707_-627-Klf7 | chr1 | Klf7 | 0.202796353 | 0 | 0 | 628 | 628 |
| chr1_64892897_64883129_-9768-Plekhm3 | chr1 | Plekhm3 | 0 | 0.182309179 | 0 | 9769 | 340 |
| chr1_65205678_65190319_+15359-Pikfyve | chr1 | Pikfyve | 0 | 0.364618359 | 0 | 15360 | 956 |
| chr1_65205678_65195656_+10022-Pikfyve | chr1 | Pikfyve | 0.202796353 | 0 | 0 | 10023 | 589 |
| chr1_69685797_69570493_-115304-Ikzf2 | chr1 | Ikzf2 | 0 | 0 | 0.180646022 | 115305 | 696 |
| chr1_7120615_7098879_+21736-Pcmtd1 | chr1 | Pcmtd1 | 0 | 0.182309179 | 0 | 21737 | 21737 |
| chr1_7120615_7120194_+421-Pcmtd1 | chr1 | Pcmtd1 | 0.202796353 | 0.364618359 | 0.722584089 | 422 | 422 |
| chr1_72198769_72192043_-6726-Mreg | chr1 | Mreg | 0 | 0 | 0.180646022 | 6727 | 6727 |
| chr1_72383769_72381619_+2150-Xrcc5 | chr1 | Xrcc5 | 0 | 0.364618359 | 0 | 2151 | 275 |
| chr1_73829966_73817217_-12749-Gm29183 | chr1 | Gm29183 | 0 | 0 | 0.180646022 | 12750 | 12750 |
| chr1_73937317_73916439_-20878-Tns1 | chr1 | Tns1 | 0 | 0.182309179 | 0 | 20879 | 2152 |
| chr1_74585697_74585384_-313-Zfp142 | chr1 | Zfp142 | 0 | 0 | 0.180646022 | 314 | 314 |
| chr1_75402473_75401055_+1418-Speg | chr1 | Speg | 0 | 0.364618359 | 0 | 1419 | 441 |
| chr1_75411461_75411286_+175-Speg | chr1 | Speg | 0 | 0 | 0.361292045 | 176 | 176 |
| chr1_75414428_75411286_+3142-Speg | chr1 | Speg | 0 | 0.182309179 | 0 | 3143 | 289 |
| chr1_75415168_75411286_+3882-Speg | chr1 | Speg | 0.405592706 | 0 | 0 | 3883 | 458 |
| chr1_78694346_78688237_+6109-Acsl3 | chr1 | Acsl3 | 0.202796353 | 0 | 0 | 6110 | 578 |
| chr1_80278088_80271524_-6564-Cul3 | chr1 | Cul3 | 0 | 0 | 0.180646022 | 6565 | 544 |
| chr1_80283821_80277964_-5857-Cul3 | chr1 | Cul3 | 0 | 0 | 0.180646022 | 5858 | 727 |
| chr1_82342862_82340319_+2543-Rhbdd1 | chr1 | Rhbdd1 | 0.608389058 | 0 | 0 | 2544 | 645 |
| chr1_84751980_84749158_-2822-Trip12 | chr1 | Trip12 | 0 | 0 | 1.445168179 | 2823 | 610 |
| chr1_84794327_84776317_-18010-Trip12 | chr1 | Trip12 | 0 | 0.182309179 | 0.361292045 | 18011 | 1226 |
| chr1_86083275_86071614_+11661-Psmd1 | chr1 | Psmd1 | 0.202796353 | 0 | 0 | 11662 | 1011 |
| chr1_86606199_86589164_+17035-Cops7b | chr1 | Cops7b | 0 | 0.182309179 | 0 | 17036 | 1794 |
| chr1_86821450_86791416_+30034-Dis3l2 | chr1 | Dis3l2 | 0 | 0.182309179 | 0 | 30035 | 349 |
| chr1_87233249_87232403_+846-Eif4e2 | chr1 | Eif4e2 | 0.202796353 | 0 | 0 | 847 | 847 |
| chr1_87374111_87364104_+10007-Gigyf2 | chr1 | Gigyf2 | 0.811185411 | 0.182309179 | 0 | 10008 | 341 |
| chr1_87403846_87364104_+39742-Gigyf2 | chr1 | Gigyf2 | 0 | 0 | 0.180646022 | 39743 | 674 |
| chr1_87436891_87364104_+72787-Gigyf2 | chr1 | Gigyf2 | 0 | 0 | 0.180646022 | 72788 | 2851 |
| chr1_87918721_87915100_+3621-Dgkd | chr1 | Dgkd | 0.202796353 | 0.364618359 | 0.180646022 | 3622 | 737 |
| chr1_87921681_87915100_+6581-Dgkd | chr1 | Dgkd | 0.202796353 | 0 | 0 | 6582 | 846 |
| chr1_87938265_87936749_+1516-Dgkd | chr1 | Dgkd | 0 | 0 | 0.180646022 | 1517 | 264 |
| chr1_87952458_87948502_-3956-Usp40 | chr1 | Usp40 | 0.202796353 | 0 | 0 | 3957 | 587 |
| chr1_89665189_89665034_+155-Agap1 | chr1 | Agap1 | 0 | 0 | 0.361292045 | 156 | 156 |
| chr1_90612336_90610093_+2243-Cops8 | chr1 | Cops8 | 0.405592706 | 0 | 0 | 2244 | 382 |
| chr1_91105297_91084946_+20351-Lrrfip1 | chr1 | Lrrfip1 | 0.405592706 | 0 | 0 | 20352 | 798 |
| chr1_91108672_91088634_+20038-Lrrfip1 | chr1 | Lrrfip1 | 3.650334351 | 0.546927538 | 0.722584089 | 20039 | 858 |
| chr1_91112297_91084946_+27351-Lrrfip1 | chr1 | Lrrfip1 | 0 | 0 | 0.361292045 | 27352 | 1219 |
| chr1_91112297_91088634_+23663-Lrrfip1 | chr1 | Lrrfip1 | 0.101398176 | 0 | 0 | 23664 | 1093 |
| chr1_91381972_91376318_-5654-Ilkap | chr1 | Ilkap | 1.013981764 | 0 | 0 | 5655 | 324 |
| chr1_92148494_92029955_-118539-Hdac4 | chr1 | Hdac4 | 0 | 0.182309179 | 0 | 118540 | 640 |
| chr1_92148494_92117471_-31023-Hdac4 | chr1 | Hdac4 | 0 | 0 | 0.180646022 | 31024 | 31024 |
| chr1_92148494_92140159_-8335-Hdac4 | chr1 | Hdac4 | 0 | 0.182309179 | 0 | 8336 | 8336 |
| chr1_92152601_92148256_-4345-Hdac4 | chr1 | Hdac4 | 0 | 0.09115459 | 0 | 4346 | 4346 |
| chr1_92470985_92460380_-10605-Ndufa10 | chr1 | Ndufa10 | 0 | 0 | 0.361292045 | 10606 | 815 |
| chr1_93429485_93412363_-17122-Hdlbp | chr1 | Hdlbp | 0.202796353 | 0 | 0 | 17123 | 2208 |
| chr1_93442250_93437094_-5156-Hdlbp | chr1 | Hdlbp | 0 | 0.364618359 | 0 | 5157 | 552 |
| chr1_93500559_93491157_+9402-Sept2 | chr1 | Sept2 | 0 | 0 | 0.180646022 | 9403 | 687 |
| chr1_93576112_93567417_+8695-Farp2 | chr1 | Farp2 | 0.405592706 | 0 | 0.903230112 | 8696 | 8696 |
| chr1_97487800_97476326_-11474-na | chr1 | na | 0.202796353 | 0.546927538 | 0 | 11475 | 11475 |
| chr1_97654689_97645542_-9147-D1Ertd622e | chr1 | D1Ertd622e | 0.811185411 | 0.911545897 | 0 | 9148 | 910 |
| chr1_9889025_9880332_+8693-Sgk3 | chr1 | Sgk3 | 0.202796353 | 0 | 0 | 8694 | 763 |
| chr10_105836220_105779588_-56632-Mettl25 | chr10 | Mettl25 | 0.405592706 | 0 | 0.180646022 | 56633 | 1580 |
| chr10_105836220_105793340_-42880-Mettl25 | chr10 | Mettl25 | 0.202796353 | 0 | 0 | 42881 | 1486 |
| chr10_106988909_106948656_-40253-Acss3 | chr10 | Acss3 | 0 | 0 | 0.541938067 | 40254 | 469 |
| chr10_107004815_106962962_-41853-Acss3 | chr10 | Acss3 | 0 | 0.182309179 | 0 | 41854 | 416 |
| chr10_108243130_108240105_+3025-Ppp1r12a | chr10 | Ppp1r12a | 0 | 0 | 0.541938067 | 3026 | 447 |
| chr10_108249069_108233999_+15070-Ppp1r12a | chr10 | Ppp1r12a | 0.202796353 | 0 | 0.180646022 | 15071 | 15071 |
| chr10_108252924_108240105_+12819-Ppp1r12a | chr10 | Ppp1r12a | 0 | 0 | 0.180646022 | 12820 | 1031 |
| chr10_108261036_108233999_+27037-Ppp1r12a | chr10 | Ppp1r12a | 0 | 0.546927538 | 0 | 27038 | 1639 |
| chr10_108261416_108248956_+12460-Ppp1r12a | chr10 | Ppp1r12a | 0.202796353 | 0 | 0 | 12461 | 12461 |
| chr10_109770324_109751432_-18892-Nav3 | chr10 | Nav3 | 0 | 0 | 0.180646022 | 18893 | 2053 |
| chr10_109883686_109852505_-31181-Nav3 | chr10 | Nav3 | 0.202796353 | 0 | 0 | 31182 | 1496 |
| chr10_11291572_11289520_+2052-Fbxo30 | chr10 | Fbxo30 | 0 | 0.182309179 | 0 | 2053 | 2053 |
| chr10_116549105_116517317_-31788-Cnot2 | chr10 | Cnot2 | 0 | 0.182309179 | 0 | 31789 | 491 |
| chr10_116549175_116527827_-21348-Cnot2 | chr10 | Cnot2 | 0 | 0 | 0.180646022 | 21349 | 494 |
| chr10_117102198_117099943_-2255-na | chr10 | na | 0 | 0 | 0.270969033 | 2256 | 2256 |
| chr10_117361360_117085032_-276328-Cpsf6 | chr10 | Cpsf6 | 0.101398176 | 0 | 0 | 276329 | 276329 |
| chr10_120825136_120821384_-3752-na | chr10 | na | 0 | 0.09115459 | 0 | 3753 | 3753 |
| chr10_120852082_120825108_-26974-Msrb3 | chr10 | Msrb3 | 0 | 0 | 0.180646022 | 26975 | 206 |
| chr10_120852092_120821384_-30708-Msrb3 | chr10 | Msrb3 | 0 | 0 | 0.361292045 | 30709 | 30709 |
| chr10_120852092_120825108_-26984-Msrb3 | chr10 | Msrb3 | 1.013981764 | 3.09925605 | 3.793566469 | 26985 | 216 |
| chr10_120853184_120825108_-28076-Msrb3 | chr10 | Msrb3 | 0 | 0 | 0.180646022 | 28077 | 28077 |
| chr10_121564105_121551251_-12854-Tbk1 | chr10 | Tbk1 | 0 | 0.182309179 | 0 | 12855 | 1932 |
| chr10_123133129_123119109_-14020-Usp15 | chr10 | Usp15 | 0 | 0 | 0.180646022 | 14021 | 1848 |
| chr10_123153127_123146794_-6333-Usp15 | chr10 | Usp15 | 0 | 0 | 0.090323011 | 6334 | 232 |
| chr10_123181800_123153041_-28759-Usp15 | chr10 | Usp15 | 0 | 0 | 0.180646022 | 28760 | 681 |
| chr10_12442093_12436281_-5812-Utrn | chr10 | Utrn | 0 | 0 | 0.361292045 | 5813 | 437 |
| chr10_12621380_12621191_-189-Utrn | chr10 | Utrn | 0.202796353 | 0 | 0 | 190 | 190 |
| chr10_12643611_12633944_-9667-Utrn | chr10 | Utrn | 0.202796353 | 0 | 0 | 9668 | 688 |
| chr10_12654834_12649143_-5691-Utrn | chr10 | Utrn | 0.202796353 | 0 | 0 | 5692 | 362 |
| chr10_12688427_12475271_-213156-Utrn | chr10 | Utrn | 0 | 0 | 0.180646022 | 213157 | 4802 |
| chr10_12688427_12621191_-67236-Utrn | chr10 | Utrn | 0.811185411 | 0.182309179 | 0 | 67237 | 3786 |
| chr10_127278408_127274714_+3694-Dctn2 | chr10 | Dctn2 | 0 | 0 | 0.180646022 | 3695 | 1017 |
| chr10_12727912_12713254_-14658-Utrn | chr10 | Utrn | 0.202796353 | 0 | 0 | 14659 | 985 |
| chr10_127305857_127299949_-5908-Mars | chr10 | Mars | 1.013981764 | 0.546927538 | 0.180646022 | 5909 | 983 |
| chr10_127305857_127304185_-1672-Mars | chr10 | Mars | 0.202796353 | 0.182309179 | 0 | 1673 | 523 |
| chr10_127648189_127646891_+1298-Stat6 | chr10 | Stat6 | 0 | 0 | 0.180646022 | 1299 | 360 |
| chr10_14011215_14004201_+7014-Hivep2 | chr10 | Hivep2 | 0 | 0.182309179 | 0 | 7015 | 7015 |
| chr10_17916032_17908034_-7998-Heca | chr10 | Heca | 0 | 0.182309179 | 0 | 7999 | 1196 |
| chr10_18096336_18092845_+3491-Reps1 | chr10 | Reps1 | 0 | 0 | 0.361292045 | 3492 | 858 |
| chr10_20029203_20023608_+5595-Map3k5 | chr10 | Map3k5 | 0.608389058 | 0.182309179 | 0 | 5596 | 641 |
| chr10_20046484_20024885_+21599-Map3k5 | chr10 | Map3k5 | 0 | 0 | 0.361292045 | 21600 | 560 |
| chr10_20059090_20019558_+39532-Map3k5 | chr10 | Map3k5 | 0.202796353 | 0 | 0 | 39533 | 1092 |
| chr10_20100197_20023608_+76589-Map3k5 | chr10 | Map3k5 | 0.405592706 | 0 | 0 | 76590 | 1803 |
| chr10_21358840_21336967_+21873-Hbs1l | chr10 | Hbs1l | 0.202796353 | 0 | 0 | 21874 | 1373 |
| chr10_22307298_22285691_+21607-H60b | chr10 | H60b | 0 | 0.364618359 | 0 | 21608 | 21608 |
| chr10_22665706_22664363_+1343-Slc2a12 | chr10 | Slc2a12 | 0.608389058 | 0 | 0.361292045 | 1344 | 1344 |
| chr10_22694126_22664363_+29763-Slc2a12 | chr10 | Slc2a12 | 0 | 0.364618359 | 0 | 29764 | 1600 |
| chr10_25443954_25441573_+2381-Epb41l2 | chr10 | Epb41l2 | 0.202796353 | 0 | 0 | 2382 | 698 |
| chr10_25502387_25441573_+60814-Epb41l2 | chr10 | Epb41l2 | 0 | 0 | 0.180646022 | 60815 | 5347 |
| chr10_26352375_26313847_-38528-L3mbtl3 | chr10 | L3mbtl3 | 0.202796353 | 0 | 0 | 38529 | 1608 |
| chr10_26352375_26336233_-16142-L3mbtl3 | chr10 | L3mbtl3 | 0.202796353 | 0 | 0 | 16143 | 954 |
| chr10_27139005_27118479_-20526-Lama2 | chr10 | Lama2 | 0 | 0.182309179 | 0 | 20527 | 728 |
| chr10_27459406_27118479_-340927-Lama2 | chr10 | Lama2 | 0 | 0.182309179 | 0 | 340928 | 5162 |
| chr10_27467374_27422472_-44902-Lama2 | chr10 | Lama2 | 0 | 0 | 0.180646022 | 44903 | 527 |
| chr10_33139283_33139074_+209-Trdn | chr10 | Trdn | 0.405592706 | 0.182309179 | 0 | 210 | 210 |
| chr10_33158278_33139074_+19204-Trdn | chr10 | Trdn | 0.202796353 | 0 | 0 | 19205 | 405 |
| chr10_33200222_33139074_+61148-Trdn | chr10 | Trdn | 0 | 0 | 0.541938067 | 61149 | 825 |
| chr10_33321334_33139074_+182260-Trdn | chr10 | Trdn | 0 | 0.182309179 | 0 | 182261 | 1098 |
| chr10_34012137_34008993_-3144-Rwdd1 | chr10 | Rwdd1 | 0.405592706 | 0 | 0 | 3145 | 197 |
| chr10_39814090_39805600_+8490-Rev3l | chr10 | Rev3l | 0 | 0 | 0.180646022 | 8491 | 462 |
| chr10_39852073_39805600_+46473-Rev3l | chr10 | Rev3l | 0 | 0 | 0.180646022 | 46474 | 7590 |
| chr10_40080807_40076564_-4243-Slc16a10 | chr10 | Slc16a10 | 0 | 0.364618359 | 0 | 4244 | 599 |
| chr10_41647149_41633968_-13181-Ccdc162 | chr10 | Ccdc162 | 0 | 0.182309179 | 0 | 13182 | 873 |
| chr10_41666796_41644571_-22225-Ccdc162 | chr10 | Ccdc162 | 0 | 0 | 0.180646022 | 22226 | 1008 |
| chr10_42438405_42415549_-22856-Lace1 | chr10 | Lace1 | 0 | 0.182309179 | 0 | 22857 | 385 |
| chr10_43074688_43066711_-7977-Sobp | chr10 | Sobp | 0.202796353 | 0.273463769 | 0 | 7978 | 7978 |
| chr10_43160844_43074593_-86251-Sobp | chr10 | Sobp | 0 | 0.182309179 | 0 | 86252 | 573 |
| chr10_43160844_43127779_-33065-Sobp | chr10 | Sobp | 1.216778117 | 0.911545897 | 1.445168179 | 33066 | 477 |
| chr10_43413547_43393871_+19676-na | chr10 | na | 0 | 0 | 0.180646022 | 19677 | 19677 |
| chr10_43936091_43921387_+14704-Rtn4ip1 | chr10 | Rtn4ip1 | 0 | 0 | 0.180646022 | 14705 | 463 |
| chr10_43992705_43975027_-17678-Aim1 | chr10 | Aim1 | 0 | 0.182309179 | 0 | 17679 | 788 |
| chr10_45618629_45588380_+30249-Hace1 | chr10 | Hace1 | 0.202796353 | 0 | 0 | 30250 | 458 |
| chr10_4783973_3463389_+1320584-Mthfd1l | chr10 | Mthfd1l | 0 | 0 | 0.361292045 | 1320585 | 1320585 |
| chr10_49535499_49523682_-11817-Grik2 | chr10 | Grik2 | 0 | 0.182309179 | 0 | 11818 | 410 |
| chr10_50767507_50690115_+77392-Ascc3 | chr10 | Ascc3 | 0 | 0.182309179 | 0 | 77393 | 3399 |
| chr10_50767507_50767091_+416-Ascc3 | chr10 | Ascc3 | 0 | 0 | 0.361292045 | 417 | 247 |
| chr10_5124963_5075789_-49174-Syne1 | chr10 | Syne1 | 0 | 0.364618359 | 0 | 49175 | 2748 |
| chr10_5222007_5146253_-75754-Syne1 | chr10 | Syne1 | 0 | 0 | 0.180646022 | 75755 | 4276 |
| chr10_52290923_52261727_+29196-Dcbld1 | chr10 | Dcbld1 | 0 | 0.182309179 | 0 | 29197 | 473 |
| chr10_5250994_5116967_-134027-Syne1 | chr10 | Syne1 | 0 | 0.182309179 | 0 | 134028 | 10740 |
| chr10_56051458_56040139_-11319-Tbc1d32 | chr10 | Tbc1d32 | 0 | 0.182309179 | 0 | 11320 | 482 |
| chr10_56151853_56123511_-28342-Tbc1d32 | chr10 | Tbc1d32 | 0 | 0 | 0.361292045 | 28343 | 413 |
| chr10_56195500_56177492_-18008-Tbc1d32 | chr10 | Tbc1d32 | 0.202796353 | 0 | 0 | 18009 | 530 |
| chr10_57512079_57495898_+16181-Hsf2 | chr10 | Hsf2 | 0 | 0 | 0.180646022 | 16182 | 1396 |
| chr10_58457784_58451984_+5800-Ranbp2 | chr10 | Ranbp2 | 0.202796353 | 0 | 0 | 5801 | 903 |
| chr10_58485881_58463819_+22062-Ranbp2 | chr10 | Ranbp2 | 0 | 0.182309179 | 0 | 22063 | 6726 |
| chr10_59104491_59083448_+21043-Sh3rf3 | chr10 | Sh3rf3 | 0 | 0 | 0.180646022 | 21044 | 748 |
| chr10_60013774_60004795_+8979-Ascc1 | chr10 | Ascc1 | 0 | 0.364618359 | 0 | 8980 | 510 |
| chr10_62365068_62281033_-84035-Hk1 | chr10 | Hk1 | 0 | 0.182309179 | 0 | 84036 | 2296 |
| chr10_62751016_62746901_-4115-Ccar1 | chr10 | Ccar1 | 0 | 0.364618359 | 0 | 4116 | 534 |
| chr10_62772065_62770855_-1210-Ccar1 | chr10 | Ccar1 | 0.202796353 | 0 | 0 | 1211 | 292 |
| chr10_62791042_62780441_-10601-Ccar1 | chr10 | Ccar1 | 0 | 0.182309179 | 0 | 10602 | 558 |
| chr10_63017610_63015802_-1808-Hnrnph3 | chr10 | Hnrnph3 | 0 | 0 | 0.361292045 | 1809 | 528 |
| chr10_63117026_63116720_-306-na | chr10 | na | 0 | 0 | 0.361292045 | 307 | 307 |
| chr10_63123397_63120029_-3368-Mypn | chr10 | Mypn | 0.202796353 | 0 | 0 | 3369 | 501 |
| chr10_63136295_63116720_-19575-Mypn | chr10 | Mypn | 0 | 0.729236718 | 0 | 19576 | 2121 |
| chr10_63136295_63130983_-5312-Mypn | chr10 | Mypn | 0.202796353 | 0 | 0 | 5313 | 949 |
| chr10_63146208_63118421_-27787-Mypn | chr10 | Mypn | 0 | 0 | 0.180646022 | 27788 | 2187 |
| chr10_63146208_63125669_-20539-Mypn | chr10 | Mypn | 0 | 0.364618359 | 0 | 20540 | 1552 |
| chr10_63169430_63152795_-16635-Mypn | chr10 | Mypn | 0 | 0 | 0.361292045 | 16636 | 554 |
| chr10_63169430_63162222_-7208-Mypn | chr10 | Mypn | 1.013981764 | 1.640782614 | 1.264522156 | 7209 | 412 |
| chr10_63169430_63163513_-5917-Mypn | chr10 | Mypn | 2.433556234 | 1.276164256 | 0.722584089 | 5918 | 340 |
| chr10_63307909_63299120_+8789-Herc4 | chr10 | Herc4 | 0 | 0 | 0.180646022 | 8790 | 411 |
| chr10_64409283_64250698_+158585-Ctnna3 | chr10 | Ctnna3 | 0 | 0.182309179 | 0 | 158586 | 327 |
| chr10_64675916_64250698_+425218-Ctnna3 | chr10 | Ctnna3 | 0 | 0 | 0.180646022 | 425219 | 837 |
| chr10_67248150_67216983_+31167-Jmjd1c | chr10 | Jmjd1c | 0 | 0.364618359 | 0 | 31168 | 6647 |
| chr10_68043261_68035789_+7472-Rtkn2 | chr10 | Rtkn2 | 0 | 0 | 0.180646022 | 7473 | 2154 |
| chr10_70154836_70142579_+12257-Ccdc6 | chr10 | Ccdc6 | 0 | 0.364618359 | 0.180646022 | 12258 | 279 |
| chr10_70167034_70154741_-12293-na | chr10 | na | 0.101398176 | 0 | 0 | 12294 | 12294 |
| chr10_70175167_70154708_+20459-Ccdc6 | chr10 | Ccdc6 | 0.202796353 | 0 | 0 | 20460 | 551 |
| chr10_71381760_71376705_+5055-Ipmk | chr10 | Ipmk | 0.202796353 | 0 | 0 | 5056 | 638 |
| chr10_75261957_75245079_+16878-Specc1l | chr10 | Specc1l | 1.216778117 | 2.005400973 | 0.722584089 | 16879 | 2256 |
| chr10_75263370_75248437_+14933-Specc1l | chr10 | Specc1l | 0.202796353 | 0 | 0 | 14934 | 714 |
| chr10_75267589_75245079_+22510-Specc1l | chr10 | Specc1l | 0.405592706 | 0 | 0 | 22511 | 2439 |
| chr10_75279961_75245079_+34882-Specc1l | chr10 | Specc1l | 0.202796353 | 0 | 0.361292045 | 34883 | 2783 |
| chr10_75279961_75257868_+22093-Specc1l | chr10 | Specc1l | 0.202796353 | 0 | 0 | 22094 | 941 |
| chr10_75279961_75263279_+16682-Specc1l | chr10 | Specc1l | 0 | 0 | 0.180646022 | 16683 | 527 |
| chr10_75916911_75910154_-6757-Smarcb1 | chr10 | Smarcb1 | 0.202796353 | 0 | 0 | 6758 | 535 |
| chr10_76370079_76367308_-2771-Pcnt | chr10 | Pcnt | 0 | 0.546927538 | 0 | 2772 | 522 |
| chr10_76393039_76379905_-13134-Pcnt | chr10 | Pcnt | 0 | 0 | 0.541938067 | 13135 | 2751 |
| chr10_76404651_76399856_-4795-Pcnt | chr10 | Pcnt | 0 | 0.546927538 | 0 | 4796 | 743 |
| chr10_7640815_7640045_-770-Pcmt1 | chr10 | Pcmt1 | 0 | 0.364618359 | 0 | 771 | 207 |
| chr10_76412649_76387427_-25222-Pcnt | chr10 | Pcnt | 0 | 0 | 0.180646022 | 25223 | 3319 |
| chr10_76412649_76399856_-12793-Pcnt | chr10 | Pcnt | 0.202796353 | 0 | 0.180646022 | 12794 | 1689 |
| chr10_76412649_76408698_-3951-Pcnt | chr10 | Pcnt | 0.202796353 | 0 | 0 | 3952 | 913 |
| chr10_7703120_7691335_+11785-Lats1 | chr10 | Lats1 | 0.202796353 | 0 | 0 | 11786 | 2139 |
| chr10_7703120_7701610_+1510-Lats1 | chr10 | Lats1 | 0 | 0.182309179 | 0 | 1511 | 1511 |
| chr10_77096535_77085267_-11268-Col18a1 | chr10 | Col18a1 | 0.202796353 | 0 | 0 | 11269 | 816 |
| chr10_77322583_77302559_-20024-Adarb1 | chr10 | Adarb1 | 0.405592706 | 0 | 0.451615056 | 20025 | 20025 |
| chr10_78204392_78200618_-3774-Trappc10 | chr10 | Trappc10 | 0 | 0.364618359 | 0 | 3775 | 1043 |
| chr10_78204392_78202880_-1512-Trappc10 | chr10 | Trappc10 | 0.608389058 | 0 | 0 | 1513 | 654 |
| chr10_78211622_78210378_-1244-Trappc10 | chr10 | Trappc10 | 0 | 0 | 0.361292045 | 1245 | 339 |
| chr10_7924872_7919114_-5758-Tab2 | chr10 | Tab2 | 0 | 0.364618359 | 0 | 5759 | 1689 |
| chr10_79710920_79709671_+1249-Bsg | chr10 | Bsg | 0 | 0 | 0.180646022 | 1250 | 666 |
| chr10_79862047_79860824_+1223-Ptbp1 | chr10 | Ptbp1 | 2.636352587 | 0 | 0 | 1224 | 180 |
| chr10_81181625_81175953_+5672-Eef2 | chr10 | Eef2 | 0 | 0 | 0.180646022 | 5673 | 5673 |
| chr10_82761913_82751384_-10529-Nfyb | chr10 | Nfyb | 0 | 0 | 0.180646022 | 10530 | 586 |
| chr10_82863640_82863015_+625-na | chr10 | na | 0 | 0 | 0.361292045 | 626 | 626 |
| chr10_83191784_83191635_-149-na | chr10 | na | 0 | 0.182309179 | 0 | 150 | 150 |
| chr10_84673528_84655945_+17583-Polr3b | chr10 | Polr3b | 0.202796353 | 0 | 0 | 17584 | 498 |
| chr10_84674719_84667322_+7397-Polr3b | chr10 | Polr3b | 0 | 0 | 0.361292045 | 7398 | 526 |
| chr10_84680464_84667322_+13142-Polr3b | chr10 | Polr3b | 0 | 0 | 0.541938067 | 13143 | 854 |
| chr10_84684304_84655945_+28359-Polr3b | chr10 | Polr3b | 0 | 0 | 0.180646022 | 28360 | 1117 |
| chr10_84699541_84667344_+32197-Polr3b | chr10 | Polr3b | 0 | 0 | 0.180646022 | 32198 | 1590 |
| chr10_84713365_84655945_+57420-Polr3b | chr10 | Polr3b | 0.202796353 | 0 | 0 | 57421 | 57421 |
| chr10_85009627_85001782_+7845-Ric8b | chr10 | Ric8b | 0 | 0.182309179 | 0 | 7846 | 517 |
| chr10_85009627_85008657_+970-Ric8b | chr10 | Ric8b | 0 | 0.182309179 | 0 | 971 | 313 |
| chr10_85133896_85133591_+305-na | chr10 | na | 0 | 0.182309179 | 0 | 306 | 306 |
| chr10_86731266_86724198_+7068-Ttc41 | chr10 | Ttc41 | 0 | 0.182309179 | 0 | 7069 | 812 |
| chr10_86736999_86729782_+7217-Ttc41 | chr10 | Ttc41 | 0 | 0.364618359 | 0 | 7218 | 1036 |
| chr10_88419560_88400652_+18908-Gnptab | chr10 | Gnptab | 0.202796353 | 0 | 0.180646022 | 18909 | 654 |
| chr10_88582048_88566508_-15540-na | chr10 | na | 0 | 0 | 0.090323011 | 15541 | 15541 |
| chr10_88819349_88795377_-23972-Utp20 | chr10 | Utp20 | 0 | 0.182309179 | 0 | 23973 | 2226 |
| chr10_89535546_89498049_-37497-Nr1h4 | chr10 | Nr1h4 | 0 | 0 | 0.180646022 | 37498 | 37498 |
| chr10_89535546_89530901_-4645-Nr1h4 | chr10 | Nr1h4 | 0 | 0.273463769 | 0 | 4646 | 4646 |
| chr10_89653056_89646759_-6297-Scyl2 | chr10 | Scyl2 | 0 | 0 | 0.361292045 | 6298 | 467 |
| chr10_89816221_89812044_+4177-Uhrf1bp1l | chr10 | Uhrf1bp1l | 0 | 0 | 0.180646022 | 4178 | 291 |
| chr10_92878612_92868723_-9889-Cfap54 | chr10 | Cfap54 | 0 | 0 | 0.541938067 | 9890 | 591 |
| chr10_93265680_93264880_-800-Elk3 | chr10 | Elk3 | 0 | 0 | 0.903230112 | 801 | 801 |
| chr10_93868961_93862377_-6584-Metap2 | chr10 | Metap2 | 0.202796353 | 0 | 0 | 6585 | 412 |
| chr10_93984493_93969089_-15404-Vezt | chr10 | Vezt | 0 | 0 | 0.361292045 | 15405 | 5356 |
| chr10_94167728_94165418_+2310-Nr2c1 | chr10 | Nr2c1 | 0.202796353 | 0 | 0 | 2311 | 419 |
| chr10_94582409_94578423_+3986-Tmcc3 | chr10 | Tmcc3 | 0 | 0.182309179 | 0 | 3987 | 1053 |
| chr10_94766517_94725647_+40870-Cep83 | chr10 | Cep83 | 0 | 0.182309179 | 0 | 40871 | 1095 |
| chr10_95709706_95709592_+114-na | chr10 | na | 0.202796353 | 0 | 0 | 115 | 115 |
| chr10_9812407_9798122_-14285-Stxbp5 | chr10 | Stxbp5 | 0 | 0 | 0.180646022 | 14286 | 935 |
| chr10_9868753_9841871_-26882-Stxbp5 | chr10 | Stxbp5 | 0 | 0 | 0.180646022 | 26883 | 26883 |
| chr11_101044623_101026660_+17963-Atp6v0a1 | chr11 | Atp6v0a1 | 0 | 0.182309179 | 0.180646022 | 17964 | 1605 |
| chr11_102005440_102004635_-805-Mpp3 | chr11 | Mpp3 | 0 | 0 | 0.180646022 | 806 | 806 |
| chr11_102538356_102500803_-37553-Gpatch8 | chr11 | Gpatch8 | 0.202796353 | 0 | 0 | 37554 | 13325 |
| chr11_103056499_103046379_+10120-Nmt1 | chr11 | Nmt1 | 0.405592706 | 0 | 0 | 10121 | 473 |
| chr11_103060248_103052206_+8042-Nmt1 | chr11 | Nmt1 | 0 | 0.182309179 | 0 | 8043 | 779 |
| chr11_104425293_104423922_-1371-Kansl1 | chr11 | Kansl1 | 0 | 0.182309179 | 0.180646022 | 1372 | 1372 |
| chr11_104426468_104368680_-57788-Kansl1 | chr11 | Kansl1 | 0 | 0.182309179 | 0 | 57789 | 1658 |
| chr11_104426468_104423922_-2546-Kansl1 | chr11 | Kansl1 | 1.825167175 | 2.91694687 | 1.083876134 | 2547 | 1417 |
| chr11_105247595_105240361_+7234-Tlk2 | chr11 | Tlk2 | 0 | 0.182309179 | 0.180646022 | 7235 | 437 |
| chr11_106123906_106096991_+26915-Map3k3 | chr11 | Map3k3 | 0.811185411 | 0.364618359 | 0 | 26916 | 498 |
| chr11_106224934_106224081_+853-Ddx42 | chr11 | Ddx42 | 0 | 0.182309179 | 0 | 854 | 389 |
| chr11_106337233_106329997_-7236-Scn4a | chr11 | Scn4a | 0 | 0 | 0.541938067 | 7237 | 1008 |
| chr11_106342520_106341422_-1098-Scn4a | chr11 | Scn4a | 0.202796353 | 0.182309179 | 0 | 1099 | 364 |
| chr11_106345729_106339154_-6575-Scn4a | chr11 | Scn4a | 0.202796353 | 0.911545897 | 0.541938067 | 6576 | 1124 |
| chr11_106349791_106342311_-7480-Scn4a | chr11 | Scn4a | 0.202796353 | 0 | 0 | 7481 | 1836 |
| chr11_106520014_106506867_-13147-Tex2 | chr11 | Tex2 | 0 | 0.364618359 | 0 | 13148 | 590 |
| chr11_106685248_106679549_-5699-Pecam1 | chr11 | Pecam1 | 0.202796353 | 0 | 0 | 5700 | 615 |
| chr11_106775600_106772652_-2948-Polg2 | chr11 | Polg2 | 0 | 0 | 0.180646022 | 2949 | 497 |
| chr11_106828624_106823020_-5604-Smurf2 | chr11 | Smurf2 | 0 | 0.182309179 | 0 | 5605 | 399 |
| chr11_106852649_106845983_-6666-Smurf2 | chr11 | Smurf2 | 0 | 0.182309179 | 0 | 6667 | 447 |
| chr11_107111635_107110813_-822-Bptf | chr11 | Bptf | 0 | 0.182309179 | 0 | 823 | 823 |
| chr11_107555575_106224081_+1331494-Cep95 | chr11 | Cep95 | 0.202796353 | 0 | 0 | 1331495 | 1331495 |
| chr11_107577899_107574938_+2961-Helz | chr11 | Helz | 0.202796353 | 0 | 0 | 2962 | 265 |
| chr11_107602508_107592706_+9802-Helz | chr11 | Helz | 0 | 0 | 0.180646022 | 9803 | 509 |
| chr11_107604363_107592706_+11657-Helz | chr11 | Helz | 0.608389058 | 0.546927538 | 1.445168179 | 11658 | 915 |
| chr11_107614118_107592706_+21412-Helz | chr11 | Helz | 0.405592706 | 0 | 0 | 21413 | 1183 |
| chr11_107620214_107592706_+27508-Helz | chr11 | Helz | 0 | 0.911545897 | 0.903230112 | 27509 | 1648 |
| chr11_107620214_107618983_+1231-Helz | chr11 | Helz | 0.202796353 | 0 | 0 | 1232 | 465 |
| chr11_107632350_107620084_+12266-Helz | chr11 | Helz | 0 | 0 | 0.180646022 | 12267 | 595 |
| chr11_107649340_107632172_+17168-Helz | chr11 | Helz | 0 | 0 | 0.361292045 | 17169 | 1005 |
| chr11_107986385_107961573_-24812-Prkca | chr11 | Prkca | 0.202796353 | 0 | 0 | 24813 | 687 |
| chr11_108057873_108012627_-45246-Prkca | chr11 | Prkca | 0 | 0 | 0.180646022 | 45247 | 630 |
| chr11_108178607_108178441_-166-na | chr11 | na | 0 | 0.182309179 | 0 | 167 | 167 |
| chr11_110310305_110292086_-18219-Abca5 | chr11 | Abca5 | 0 | 0 | 0.180646022 | 18220 | 1893 |
| chr11_110310305_110301731_-8574-Abca5 | chr11 | Abca5 | 0 | 0.364618359 | 0 | 8575 | 865 |
| chr11_110496466_110490145_+6321-Map2k6 | chr11 | Map2k6 | 0 | 0 | 0.541938067 | 6322 | 580 |
| chr11_114902614_114870672_+31942-Cd300a | chr11 | Cd300a | 0.202796353 | 0 | 0 | 31943 | 31943 |
| chr11_115786312_115785440_+872-Tmem94 | chr11 | Tmem94 | 0.202796353 | 0 | 0 | 873 | 385 |
| chr11_115927434_115925407_-2027-Recql5 | chr11 | Recql5 | 0 | 0.546927538 | 0 | 2028 | 218 |
| chr11_115957963_115957361_+602-Sap30bp | chr11 | Sap30bp | 0.202796353 | 0 | 0 | 603 | 603 |
| chr11_116050804_116047677_+3127-Unk | chr11 | Unk | 0.202796353 | 0 | 0 | 3128 | 772 |
| chr11_116160110_116154707_-5403-Fbf1 | chr11 | Fbf1 | 0 | 0.182309179 | 0 | 5404 | 1075 |
| chr11_116183624_116180943_-2681-Acox1 | chr11 | Acox1 | 0 | 0.182309179 | 0 | 2682 | 389 |
| chr11_116189575_116189025_-550-Acox1 | chr11 | Acox1 | 0.608389058 | 0.182309179 | 0 | 551 | 551 |
| chr11_117150738_117077108_+73630-Sec14l1 | chr11 | Sec14l1 | 0.202796353 | 0 | 0 | 73631 | 73631 |
| chr11_117150738_117149146_+1592-Sec14l1 | chr11 | Sec14l1 | 0 | 0.364618359 | 0 | 1593 | 378 |
| chr11_11761353_11748311_+13042-Ikzf1 | chr11 | Ikzf1 | 0 | 0 | 0.180646022 | 13043 | 687 |
| chr11_119154075_119151028_-3047-Tbc1d16 | chr11 | Tbc1d16 | 0.405592706 | 0.546927538 | 0 | 3048 | 367 |
| chr11_119468080_119452511_+15569-Rnf213 | chr11 | Rnf213 | 0.202796353 | 0 | 0.180646022 | 15570 | 2323 |
| chr11_119468153_119452511_+15642-Rnf213 | chr11 | Rnf213 | 0 | 0.182309179 | 0 | 15643 | 15643 |
| chr11_120134882_120128423_-6459-Slc38a10 | chr11 | Slc38a10 | 0 | 0.182309179 | 0 | 6460 | 505 |
| chr11_120141645_120128423_-13222-Slc38a10 | chr11 | Slc38a10 | 0.202796353 | 0 | 0 | 13223 | 914 |
| chr11_120416380_120409612_-6768-Nploc4 | chr11 | Nploc4 | 0.202796353 | 0 | 0 | 6769 | 463 |
| chr11_120416380_120413560_-2820-Nploc4 | chr11 | Nploc4 | 0 | 0 | 0.180646022 | 2821 | 304 |
| chr11_120689341_120678420_+10921-Aspscr1 | chr11 | Aspscr1 | 0.202796353 | 0 | 0 | 10922 | 663 |
| chr11_120973969_120967995_-5974-Csnk1d | chr11 | Csnk1d | 0 | 0 | 0.541938067 | 5975 | 1010 |
| chr11_121219923_121207639_+12284-Hexdc | chr11 | Hexdc | 0.405592706 | 0 | 0 | 12285 | 1035 |
| chr11_16883613_16881464_+2149-Egfr | chr11 | Egfr | 0.202796353 | 0 | 0 | 2150 | 249 |
| chr11_20094445_20077188_-17257-Actr2 | chr11 | Actr2 | 0 | 0.182309179 | 0 | 17258 | 722 |
| chr11_20712315_20703711_-8604-Aftph | chr11 | Aftph | 0.405592706 | 0 | 0 | 8605 | 459 |
| chr11_21268863_21260288_+8575-na | chr11 | na | 0 | 0 | 0.180646022 | 8576 | 8576 |
| chr11_21333810_20741306_-592504-Ugp2 | chr11 | Ugp2 | 0 | 0.182309179 | 0 | 592505 | 592505 |
| chr11_22062887_22053432_-9455-Ehbp1 | chr11 | Ehbp1 | 0 | 0.182309179 | 0 | 9456 | 603 |
| chr11_23364512_23343320_+21192-Usp34 | chr11 | Usp34 | 0.405592706 | 0 | 0 | 21193 | 1844 |
| chr11_23421366_23382554_+38812-Usp34 | chr11 | Usp34 | 0 | 0.182309179 | 0.180646022 | 38813 | 2265 |
| chr11_23426795_23420040_+6755-Usp34 | chr11 | Usp34 | 0 | 0 | 0.180646022 | 6756 | 630 |
| chr11_23484181_23464217_+19964-Usp34 | chr11 | Usp34 | 0 | 0 | 0.180646022 | 19965 | 1541 |
| chr11_26550075_26534211_-15864-na | chr11 | na | 0 | 0 | 0.090323011 | 15865 | 15865 |
| chr11_28505430_28434081_-71349-Ccdc85a | chr11 | Ccdc85a | 0 | 0.364618359 | 0 | 71350 | 71350 |
| chr11_29205704_29198793_+6911-Smek2 | chr11 | Smek2 | 0 | 0.364618359 | 0 | 6912 | 436 |
| chr11_29530692_29529929_+763-Mtif2 | chr11 | Mtif2 | 0.202796353 | 0 | 0 | 764 | 338 |
| chr11_29535266_29533226_+2040-Mtif2 | chr11 | Mtif2 | 0.202796353 | 0 | 0 | 2041 | 510 |
| chr11_30219772_30219579_-193-Sptbn1 | chr11 | Sptbn1 | 0.202796353 | 0 | 0 | 194 | 194 |
| chr11_30812164_30791092_+21072-Psme4 | chr11 | Psme4 | 0.202796353 | 0 | 0 | 21073 | 1261 |
| chr11_30998521_30856030_+142491-Psme4 | chr11 | Psme4 | 0 | 0.182309179 | 0 | 142492 | 142492 |
| chr11_31058999_31055458_+3541-Asb3 | chr11 | Asb3 | 0 | 0.546927538 | 0 | 3542 | 427 |
| chr11_31061586_31055458_+6128-Asb3 | chr11 | Asb3 | 0 | 2.187710153 | 0.722584089 | 6129 | 625 |
| chr11_31104474_31081362_+23112-Asb3 | chr11 | Asb3 | 0.202796353 | 0 | 0 | 23113 | 23113 |
| chr11_31708256_31699872_-8384-na | chr11 | na | 0 | 0 | 0.180646022 | 8385 | 8385 |
| chr11_3221294_3220244_+1050-Eif4enif1 | chr11 | Eif4enif1 | 0 | 0 | 0.180646022 | 1051 | 489 |
| chr11_3254642_3252160_-2482-Drg1 | chr11 | Drg1 | 0 | 0 | 0.180646022 | 2483 | 422 |
| chr11_35570731_35508223_+62508-Slit3 | chr11 | Slit3 | 0 | 0 | 0.180646022 | 62509 | 380 |
| chr11_35651819_35632712_+19107-Slit3 | chr11 | Slit3 | 0 | 0.182309179 | 0 | 19108 | 510 |
| chr11_43705477_43699279_+6198-Pwwp2a | chr11 | Pwwp2a | 0.405592706 | 0 | 0 | 6199 | 1123 |
| chr11_43705567_43704534_+1033-Pwwp2a | chr11 | Pwwp2a | 0 | 0.364618359 | 0 | 1034 | 1034 |
| chr11_43706216_43704534_+1682-Pwwp2a | chr11 | Pwwp2a | 0 | 0.182309179 | 0 | 1683 | 1683 |
| chr11_43706294_43704534_+1760-Pwwp2a | chr11 | Pwwp2a | 0 | 0 | 0.180646022 | 1761 | 1761 |
| chr11_43721963_43686357_+35606-Pwwp2a | chr11 | Pwwp2a | 0.202796353 | 0 | 0 | 35607 | 35607 |
| chr11_43721963_43704534_+17429-Pwwp2a | chr11 | Pwwp2a | 0 | 0 | 0.361292045 | 17430 | 3498 |
| chr11_4531305_4517741_-13564-Mtmr3 | chr11 | Mtmr3 | 0.608389058 | 0.546927538 | 0 | 13565 | 541 |
| chr11_4546268_4517741_-28527-Mtmr3 | chr11 | Mtmr3 | 0 | 0 | 0.180646022 | 28528 | 621 |
| chr11_45895129_45883710_+11419-Clint1 | chr11 | Clint1 | 0 | 0.364618359 | 0 | 11420 | 971 |
| chr11_46977822_46977668_+154-na | chr11 | na | 0.405592706 | 0 | 0.361292045 | 155 | 155 |
| chr11_47195132_47195031_-101-Sgcd | chr11 | Sgcd | 0.202796353 | 0 | 0.090323011 | 102 | 102 |
| chr11_4806336_4780578_-25758-na | chr11 | na | 0 | 0 | 0.180646022 | 25759 | 25759 |
| chr11_4806336_4799861_-6475-Nf2 | chr11 | Nf2 | 0 | 0 | 0.180646022 | 6476 | 294 |
| chr11_4818627_4803678_-14949-Nf2 | chr11 | Nf2 | 0 | 0 | 0.361292045 | 14950 | 435 |
| chr11_4820493_4803678_-16815-Nf2 | chr11 | Nf2 | 0 | 0.182309179 | 0 | 16816 | 561 |
| chr11_4820493_4808257_-12236-Nf2 | chr11 | Nf2 | 0.304194529 | 0 | 0.541938067 | 12237 | 402 |
| chr11_49215945_49212695_+3250-Zfp62 | chr11 | Zfp62 | 0 | 0.182309179 | 0 | 3251 | 2029 |
| chr11_49819254_49809034_+10220-Gfpt2 | chr11 | Gfpt2 | 0 | 0 | 0.361292045 | 10221 | 580 |
| chr11_50267010_50263250_-3760-Maml1 | chr11 | Maml1 | 0.405592706 | 0 | 0 | 3761 | 1650 |
| chr11_50308929_50301758_-7171-Canx | chr11 | Canx | 0.405592706 | 0 | 0 | 7172 | 721 |
| chr11_50308929_50304326_-4603-Canx | chr11 | Canx | 0 | 0.911545897 | 0.180646022 | 4604 | 607 |
| chr11_50308929_50307470_-1459-Canx | chr11 | Canx | 0.405592706 | 0 | 0 | 1460 | 417 |
| chr11_50912401_50910436_-1965-Zfp2 | chr11 | Zfp2 | 0 | 0 | 0.541938067 | 1966 | 383 |
| chr11_51731905_51726485_-5420-Sec24a | chr11 | Sec24a | 0.202796353 | 0 | 0 | 5421 | 340 |
| chr11_51734661_51708935_-25726-Sec24a | chr11 | Sec24a | 0 | 0.364618359 | 0 | 25727 | 1809 |
| chr11_51734661_51721815_-12846-Sec24a | chr11 | Sec24a | 0 | 0.364618359 | 0 | 12847 | 981 |
| chr11_51734661_51729452_-5209-Sec24a | chr11 | Sec24a | 0 | 1.458473435 | 0.361292045 | 5210 | 639 |
| chr11_51736521_51700814_-35707-Sec24a | chr11 | Sec24a | 0 | 0 | 0.180646022 | 35708 | 2492 |
| chr11_51736521_51707128_-29393-Sec24a | chr11 | Sec24a | 0 | 0 | 0.180646022 | 29394 | 2212 |
| chr11_51736521_51712189_-24332-Sec24a | chr11 | Sec24a | 0 | 0.182309179 | 0 | 24333 | 1869 |
| chr11_51736521_51726485_-10036-Sec24a | chr11 | Sec24a | 0 | 0.546927538 | 0 | 10037 | 920 |
| chr11_52119304_52117987_+1317-Ppp2ca | chr11 | Ppp2ca | 0 | 0.182309179 | 0 | 1318 | 426 |
| chr11_52361152_52113120_+248032-Skp1a | chr11 | Skp1a | 0 | 0 | 0.180646022 | 248033 | 248033 |
| chr11_52385733_52374299_+11434-Vdac1 | chr11 | Vdac1 | 0 | 0.182309179 | 0 | 11435 | 705 |
| chr11_5243937_5209939_-33998-Kremen1 | chr11 | Kremen1 | 0.202796353 | 0 | 0 | 33999 | 534 |
| chr11_53684301_53668022_-16279-Rad50 | chr11 | Rad50 | 0 | 0.182309179 | 0.180646022 | 16280 | 1596 |
| chr11_54350701_54335052_+15649-Acsl6 | chr11 | Acsl6 | 0 | 0 | 0.180646022 | 15650 | 941 |
| chr11_54504906_54502259_+2647-Fnip1 | chr11 | Fnip1 | 0 | 0 | 0.180646022 | 2648 | 1586 |
| chr11_54939735_54926704_-13031-Tnip1 | chr11 | Tnip1 | 0 | 0 | 0.180646022 | 13032 | 839 |
| chr11_5803179_5802999_-180-Pgam2 | chr11 | Pgam2 | 0.202796353 | 0 | 0 | 181 | 181 |
| chr11_58115375_58109556_+5819-Cnot8 | chr11 | Cnot8 | 0 | 0.182309179 | 0 | 5820 | 823 |
| chr11_5836374_5831660_-4714-Polm | chr11 | Polm | 0 | 0.182309179 | 0 | 4715 | 645 |
| chr11_59000691_59000537_+154-na | chr11 | na | 0.202796353 | 0 | 0 | 155 | 155 |
| chr11_59052711_59049425_-3286-Obscn | chr11 | Obscn | 0.202796353 | 0 | 0 | 3287 | 1356 |
| chr11_59061687_59055377_-6310-Obscn | chr11 | Obscn | 52.22006085 | 72.74136258 | 0 | 6311 | 1056 |
| chr11_59064450_59015436_-49014-Obscn | chr11 | Obscn | 0 | 0 | 0.180646022 | 49015 | 9916 |
| chr11_59064450_59060844_-3606-Obscn | chr11 | Obscn | 0 | 0.182309179 | 0 | 3607 | 1539 |
| chr11_59108017_59106295_-1722-Obscn | chr11 | Obscn | 91.15696059 | 0 | 0 | 1723 | 552 |
| chr11_59122895_59115659_-7236-Obscn | chr11 | Obscn | 0 | 0 | 46.96796581 | 7237 | 552 |
| chr11_59129845_59106295_-23550-Obscn | chr11 | Obscn | 0 | 90.06073462 | 0 | 23551 | 2208 |
| chr11_5990422_5972217_-18205-Camk2b | chr11 | Camk2b | 0 | 0.182309179 | 0 | 18206 | 1858 |
| chr11_60190622_60185096_+5526-Rai1 | chr11 | Rai1 | 0 | 0 | 0.180646022 | 5527 | 5527 |
| chr11_60280283_60258856_-21427-Tom1l2 | chr11 | Tom1l2 | 0 | 0 | 0.361292045 | 21428 | 725 |
| chr11_60462634_60460787_+1847-Drg2 | chr11 | Drg2 | 0.405592706 | 0 | 0 | 1848 | 430 |
| chr11_60749589_60747866_-1723-Top3a | chr11 | Top3a | 0 | 0 | 0.361292045 | 1724 | 596 |
| chr11_60852511_60844165_+8346-Dhrs7b | chr11 | Dhrs7b | 0 | 0.364618359 | 0 | 8347 | 593 |
| chr11_6113431_6108076_-5355-Nudcd3 | chr11 | Nudcd3 | 0 | 0.182309179 | 0 | 5356 | 469 |
| chr11_6125561_6108076_-17485-Nudcd3 | chr11 | Nudcd3 | 0 | 0 | 0.180646022 | 17486 | 613 |
| chr11_61535383_61532518_-2865-Epn2 | chr11 | Epn2 | 0 | 0.182309179 | 0.180646022 | 2866 | 435 |
| chr11_61791583_61787429_-4154-Ulk2 | chr11 | Ulk2 | 0.202796353 | 0 | 0 | 4155 | 359 |
| chr11_61909946_61886700_-23246-Akap10 | chr11 | Akap10 | 0 | 0 | 0.180646022 | 23247 | 957 |
| chr11_61922829_61915024_-7805-Akap10 | chr11 | Akap10 | 0 | 0.182309179 | 0 | 7806 | 789 |
| chr11_62292218_62283597_+8621-Ttc19 | chr11 | Ttc19 | 0.811185411 | 0 | 0.361292045 | 8622 | 364 |
| chr11_62313178_62284131_+29047-Ttc19 | chr11 | Ttc19 | 0 | 0 | 0.180646022 | 29048 | 719 |
| chr11_62315944_62283597_+32347-Ttc19 | chr11 | Ttc19 | 0 | 0 | 0.180646022 | 32348 | 2614 |
| chr11_62330924_62325487_-5437-Ncor1 | chr11 | Ncor1 | 0.405592706 | 0 | 0 | 5438 | 792 |
| chr11_62345318_62342997_-2321-Ncor1 | chr11 | Ncor1 | 0 | 0 | 0.361292045 | 2322 | 696 |
| chr11_62354688_62351378_-3310-Ncor1 | chr11 | Ncor1 | 0 | 0.364618359 | 0 | 3311 | 490 |
| chr11_62398437_62373076_-25361-Ncor1 | chr11 | Ncor1 | 0 | 0 | 0.180646022 | 25362 | 1838 |
| chr11_62401267_62376635_-24632-Ncor1 | chr11 | Ncor1 | 0 | 0.182309179 | 0 | 24633 | 1358 |
| chr11_62433699_62419598_-14101-Ncor1 | chr11 | Ncor1 | 0.202796353 | 0 | 0 | 14102 | 513 |
| chr11_6317080_6296952_+20128-Ogdh | chr11 | Ogdh | 0.202796353 | 0 | 0 | 20129 | 543 |
| chr11_6334648_6313788_+20860-Ogdh | chr11 | Ogdh | 0 | 0.182309179 | 0 | 20861 | 456 |
| chr11_6342623_6334533_+8090-Ogdh | chr11 | Ogdh | 0 | 0 | 0.180646022 | 8091 | 818 |
| chr11_65032195_65021941_-10254-Arhgap44 | chr11 | Arhgap44 | 0 | 0.182309179 | 0 | 10255 | 662 |
| chr11_65041510_65021941_-19569-Arhgap44 | chr11 | Arhgap44 | 0.405592706 | 0 | 0 | 19570 | 941 |
| chr11_67191466_67096301_+95165-Myh2 | chr11 | Myh2 | 3.244741645 | 6.198512099 | 30.07756272 | 95166 | 95166 |
| chr11_67204463_67176537_+27926-Myh2 | chr11 | Myh2 | 4.258723409 | 5.469275382 | 20.41300052 | 27927 | 27927 |
| chr11_67211584_67210892_+692-Myh1 | chr11 | Myh1 | 0 | 0 | 0.361292045 | 693 | 517 |
| chr11_67243083_67084943_+158140-Myh1 | chr11 | Myh1 | 0 | 0.364618359 | 0 | 158141 | 158141 |
| chr11_67246541_67181035_+65506-Myh1 | chr11 | Myh1 | 99.97860194 | 18.23091794 | 45.07118257 | 65507 | 65507 |
| chr11_67249915_67245445_+4470-Myh4 | chr11 | Myh4 | 0.202796353 | 0 | 0 | 4471 | 909 |
| chr11_67257295_67194348_+62947-Myh1 | chr11 | Myh1 | 0 | 1.458473435 | 0 | 62948 | 62948 |
| chr11_67258964_67249226_+9738-Myh4 | chr11 | Myh4 | 0 | 0.182309179 | 0 | 9739 | 3537 |
| chr11_67297622_67252555_+45067-Myh8 | chr11 | Myh8 | 50.49629185 | 4.648884074 | 19.87106246 | 45068 | 45068 |
| chr11_68721476_68699210_+22266-Myh10 | chr11 | Myh10 | 0 | 0 | 0.180646022 | 22267 | 533 |
| chr11_68836323_68833354_-2969-Ndel1 | chr11 | Ndel1 | 0.202796353 | 0 | 0 | 2970 | 266 |
| chr11_68845424_68829915_-15509-Ndel1 | chr11 | Ndel1 | 0.405592706 | 0 | 0.180646022 | 15510 | 956 |
| chr11_68845424_68836150_-9274-Ndel1 | chr11 | Ndel1 | 0.202796353 | 0.182309179 | 0 | 9275 | 712 |
| chr11_69002615_69001733_-882-Pfas | chr11 | Pfas | 0.202796353 | 0 | 0 | 883 | 242 |
| chr11_69009448_69001733_-7715-Pfas | chr11 | Pfas | 0 | 0 | 0.180646022 | 7716 | 7716 |
| chr11_69028064_69027254_+810-Ctc1 | chr11 | Ctc1 | 0 | 0 | 0.180646022 | 811 | 612 |
| chr11_72075035_72070303_-4732-Pitpnm3 | chr11 | Pitpnm3 | 0.202796353 | 0 | 0 | 4733 | 811 |
| chr11_72404004_72399874_-4130-Smtnl2 | chr11 | Smtnl2 | 3.853130704 | 2.91694687 | 1.264522156 | 4131 | 854 |
| chr11_72590390_72586486_-3904-na | chr11 | na | 0 | 0.182309179 | 0 | 3905 | 3905 |
| chr11_72663173_72645537_+17636-Ube2g1 | chr11 | Ube2g1 | 0 | 0.182309179 | 0 | 17637 | 17637 |
| chr11_72673057_72645537_+27520-Ube2g1 | chr11 | Ube2g1 | 0 | 0 | 0.722584089 | 27521 | 27521 |
| chr11_72679492_72645537_+33955-Ube2g1 | chr11 | Ube2g1 | 0.202796353 | 0 | 0.180646022 | 33956 | 33956 |
| chr11_72789217_72778283_-10934-Cyb5d2 | chr11 | Cyb5d2 | 0 | 0.182309179 | 0 | 10935 | 998 |
| chr11_74946219_74928993_+17226-Smg6 | chr11 | Smg6 | 0 | 0 | 0.903230112 | 17227 | 2570 |
| chr11_75692732_75692197_+535-Crk | chr11 | Crk | 0 | 0 | 0.180646022 | 536 | 536 |
| chr11_76117805_76113941_-3864-Vps53 | chr11 | Vps53 | 0 | 0 | 0.541938067 | 3865 | 429 |
| chr11_76121596_76117059_-4537-Vps53 | chr11 | Vps53 | 0 | 0.182309179 | 0 | 4538 | 366 |
| chr11_76278591_76272376_-6215-Nxn | chr11 | Nxn | 0 | 0.729236718 | 0 | 6216 | 460 |
| chr11_76754800_76748508_-6292-Gosr1 | chr11 | Gosr1 | 0 | 0.546927538 | 0.180646022 | 6293 | 363 |
| chr11_76813652_76805976_-7676-Cpd | chr11 | Cpd | 0 | 0 | 0.180646022 | 7677 | 990 |
| chr11_77398269_77348096_+50173-Ssh2 | chr11 | Ssh2 | 0.202796353 | 0 | 0 | 50174 | 627 |
| chr11_77442058_77425301_+16757-Ssh2 | chr11 | Ssh2 | 0 | 0 | 0.180646022 | 16758 | 618 |
| chr11_77450222_77425301_+24921-Ssh2 | chr11 | Ssh2 | 0 | 0.182309179 | 0 | 24922 | 1453 |
| chr11_77450222_77449388_+834-Ssh2 | chr11 | Ssh2 | 0 | 0 | 0.541938067 | 835 | 835 |
| chr11_77553909_77541625_-12284-Taok1 | chr11 | Taok1 | 0.202796353 | 0 | 0 | 12285 | 1023 |
| chr11_77605638_77541625_-64013-Taok1 | chr11 | Taok1 | 0 | 0 | 0.180646022 | 64014 | 2721 |
| chr11_77812719_77807791_+4928-Myo18a | chr11 | Myo18a | 0 | 0.182309179 | 0 | 4929 | 921 |
| chr11_77812719_77809799_+2920-Myo18a | chr11 | Myo18a | 0 | 0 | 0.180646022 | 2921 | 771 |
| chr11_77821186_77815856_+5330-na | chr11 | na | 0 | 0 | 0.090323011 | 5331 | 5331 |
| chr11_77853402_77853217_+185-Myo18a | chr11 | Myo18a | 0 | 0 | 0.361292045 | 186 | 186 |
| chr11_78321766_78319986_+1780-Spag5 | chr11 | Spag5 | 0 | 0.182309179 | 0 | 1781 | 740 |
| chr11_78336830_78335427_+1403-Pigs | chr11 | Pigs | 0.608389058 | 0 | 0 | 1404 | 351 |
| chr11_79444209_79408414_+35795-Nf1 | chr11 | Nf1 | 0.202796353 | 0 | 0 | 35796 | 2300 |
| chr11_79473475_79418552_+54923-Nf1 | chr11 | Nf1 | 0 | 0.364618359 | 0 | 54924 | 3470 |
| chr11_79943230_79942208_-1022-na | chr11 | na | 0 | 0 | 0.180646022 | 1023 | 1023 |
| chr11_80346886_80319558_+27328-Rhbdl3 | chr11 | Rhbdl3 | 0.202796353 | 0 | 0 | 27329 | 808 |
| chr11_80346886_80330557_+16329-Rhbdl3 | chr11 | Rhbdl3 | 0 | 0.182309179 | 0 | 16330 | 424 |
| chr11_80389821_80387802_+2019-Zfp207 | chr11 | Zfp207 | 0 | 0 | 0.180646022 | 2020 | 431 |
| chr11_80394531_80387802_+6729-Zfp207 | chr11 | Zfp207 | 0 | 0 | 0.361292045 | 6730 | 999 |
| chr11_80404766_80395102_+9664-na | chr11 | na | 0 | 0.09115459 | 0 | 9665 | 9665 |
| chr11_82926187_82919724_+6463-Unc45b | chr11 | Unc45b | 0 | 0 | 0.361292045 | 6464 | 644 |
| chr11_83502852_83501115_+1737-Taf15 | chr11 | Taf15 | 0 | 0.182309179 | 0 | 1738 | 223 |
| chr11_83949694_83948445_+1249-Ddx52 | chr11 | Ddx52 | 0.202796353 | 0 | 0 | 1250 | 256 |
| chr11_83957537_83955289_+2248-Ddx52 | chr11 | Ddx52 | 0 | 0 | 0.180646022 | 2249 | 299 |
| chr11_83994462_83986814_+7648-Synrg | chr11 | Synrg | 0.202796353 | 0 | 0.361292045 | 7649 | 892 |
| chr11_83994462_83987913_+6549-na | chr11 | na | 0 | 0.09115459 | 0 | 6550 | 6550 |
| chr11_83994462_83987913_+6549-Synrg | chr11 | Synrg | 0.202796353 | 0 | 0.180646022 | 6550 | 658 |
| chr11_84009550_83986814_+22736-Synrg | chr11 | Synrg | 0 | 0.364618359 | 0 | 22737 | 2001 |
| chr11_84009550_84008618_+932-Synrg | chr11 | Synrg | 0 | 0 | 0.180646022 | 933 | 933 |
| chr11_85007079_85005808_-1271-Usp32 | chr11 | Usp32 | 0.405592706 | 0 | 0 | 1272 | 208 |
| chr11_8532509_8518199_-14310-Tns3 | chr11 | Tns3 | 0.202796353 | 0 | 0 | 14311 | 551 |
| chr11_85509692_85451661_+58031-Bcas3 | chr11 | Bcas3 | 0 | 0.182309179 | 0 | 58032 | 1010 |
| chr11_85559171_85531793_+27378-Bcas3 | chr11 | Bcas3 | 0 | 0 | 0.180646022 | 27379 | 588 |
| chr11_85576882_85554190_+22692-Bcas3 | chr11 | Bcas3 | 0.202796353 | 0 | 0 | 22693 | 493 |
| chr11_85583994_85451661_+132333-Bcas3 | chr11 | Bcas3 | 0 | 0.182309179 | 0 | 132334 | 1994 |
| chr11_86308912_86298280_-10632-Med13 | chr11 | Med13 | 0 | 0.182309179 | 0 | 10633 | 1835 |
| chr11_8642160_8600555_-41605-Tns3 | chr11 | Tns3 | 0.202796353 | 0 | 0 | 41606 | 41606 |
| chr11_86535460_86504266_-31194-Rps6kb1 | chr11 | Rps6kb1 | 0 | 0.182309179 | 0 | 31195 | 1295 |
| chr11_86561376_86548783_+12593-Tubd1 | chr11 | Tubd1 | 0.405592706 | 0 | 0 | 12594 | 1216 |
| chr11_86665878_86661123_-4755-Vmp1 | chr11 | Vmp1 | 0 | 0.364618359 | 0.361292045 | 4756 | 440 |
| chr11_86711575_86707470_-4105-Cltc | chr11 | Cltc | 0 | 0.182309179 | 0 | 4106 | 504 |
| chr11_90267581_90257520_+10061-Mmd | chr11 | Mmd | 0.202796353 | 0 | 0 | 10062 | 490 |
| chr11_90345978_90340787_-5191-Hlf | chr11 | Hlf | 0.202796353 | 0 | 0 | 5192 | 412 |
| chr11_90548987_90535480_-13507-Stxbp4 | chr11 | Stxbp4 | 0 | 0.182309179 | 0 | 13508 | 478 |
| chr11_90600280_90571722_-28558-Stxbp4 | chr11 | Stxbp4 | 0 | 0 | 0.180646022 | 28559 | 440 |
| chr11_90607451_90535480_-71971-Stxbp4 | chr11 | Stxbp4 | 0.405592706 | 0.364618359 | 0 | 71972 | 1321 |
| chr11_90607451_90548811_-58640-Stxbp4 | chr11 | Stxbp4 | 0.202796353 | 0 | 0 | 58641 | 1020 |
| chr11_90615036_90592332_-22704-Stxbp4 | chr11 | Stxbp4 | 0 | 0 | 0.180646022 | 22705 | 22705 |
| chr11_93918947_93905159_+13788-Mbtd1 | chr11 | Mbtd1 | 0.202796353 | 0 | 0 | 13789 | 13789 |
| chr11_93926326_93905159_+21167-Mbtd1 | chr11 | Mbtd1 | 0 | 0 | 0.180646022 | 21168 | 1285 |
| chr11_94102158_94097218_+4940-Spag9 | chr11 | Spag9 | 0 | 0.182309179 | 0 | 4941 | 690 |
| chr11_94339733_94333901_+5832-Ankrd40 | chr11 | Ankrd40 | 0.608389058 | 0 | 0 | 5833 | 992 |
| chr11_94942114_94941102_+1012-Col1a1 | chr11 | Col1a1 | 0 | 0 | 0.180646022 | 1013 | 351 |
| chr11_95754055_95749000_+5055-Zfp652 | chr11 | Zfp652 | 0.608389058 | 0 | 0 | 5056 | 5056 |
| chr11_96323445_96322111_+1334-na | chr11 | na | 0 | 0 | 0.541938067 | 1335 | 1335 |
| chr11_97329521_97328115_+1406-na | chr11 | na | 0.405592706 | 0 | 0 | 1407 | 1407 |
| chr11_97378711_97373066_+5645-Socs7 | chr11 | Socs7 | 0 | 0 | 0.180646022 | 5646 | 1160 |
| chr11_97378711_97377936_+775-Socs7 | chr11 | Socs7 | 0.405592706 | 0.546927538 | 0 | 776 | 300 |
| chr11_98011497_98009663_-1834-na | chr11 | na | 0.101398176 | 0 | 0 | 1835 | 1835 |
| chr12_100382178_100340497_-41681-Ttc7b | chr12 | Ttc7b | 0 | 0.182309179 | 0 | 41682 | 720 |
| chr12_100385960_100340497_-45463-Ttc7b | chr12 | Ttc7b | 0.202796353 | 0 | 0 | 45464 | 943 |
| chr12_100495440_100446860_-48580-Ttc7b | chr12 | Ttc7b | 0 | 0 | 0.180646022 | 48581 | 422 |
| chr12_100500230_100495272_-4958-Ttc7b | chr12 | Ttc7b | 0 | 0.182309179 | 0 | 4959 | 324 |
| chr12_100616104_100595815_-20289-Rps6ka5 | chr12 | Rps6ka5 | 0 | 0.182309179 | 0 | 20290 | 296 |
| chr12_100619641_100595815_-23826-Rps6ka5 | chr12 | Rps6ka5 | 0.202796353 | 0.546927538 | 0.903230112 | 23827 | 412 |
| chr12_100654489_100615997_-38492-Rps6ka5 | chr12 | Rps6ka5 | 0 | 0.182309179 | 0 | 38493 | 443 |
| chr12_100859652_100838500_+21152-9030617O03Rik | chr12 | 9030617O03Rik | 0 | 0 | 0.180646022 | 21153 | 1304 |
| chr12_100871506_100867104_+4402-9030617O03Rik | chr12 | 9030617O03Rik | 0 | 0.364618359 | 0 | 4403 | 355 |
| chr12_100966461_100940085_-26376-Ccdc88c | chr12 | Ccdc88c | 0.202796353 | 0 | 0 | 26377 | 2359 |
| chr12_100971279_100967808_-3471-Ccdc88c | chr12 | Ccdc88c | 0 | 0 | 0.361292045 | 3472 | 469 |
| chr12_101051662_101044490_-7172-Smek1 | chr12 | Smek1 | 0.202796353 | 0 | 0 | 7173 | 720 |
| chr12_101890618_101871824_-18794-Trip11 | chr12 | Trip11 | 0.202796353 | 0 | 0 | 18795 | 3965 |
| chr12_102484574_102471998_+12576-Golga5 | chr12 | Golga5 | 0 | 0.182309179 | 0 | 12577 | 1515 |
| chr12_102812843_102807900_-4943-Btbd7 | chr12 | Btbd7 | 0.202796353 | 0 | 0 | 4944 | 446 |
| chr12_103338614_103333292_-5322-Asb2 | chr12 | Asb2 | 0 | 0 | 0.180646022 | 5323 | 5323 |
| chr12_103345932_101022294_-2323638-Trip11 | chr12 | Trip11 | 0 | 0 | 0.180646022 | 2323639 | 0 |
| chr12_105641293_105639202_-2091-Atg2b | chr12 | Atg2b | 0 | 0 | 0.361292045 | 2092 | 345 |
| chr12_105651523_105648313_-3210-Atg2b | chr12 | Atg2b | 0 | 0 | 0.180646022 | 3211 | 625 |
| chr12_105662961_105651381_-11580-Atg2b | chr12 | Atg2b | 0.405592706 | 0.182309179 | 0.361292045 | 11581 | 1669 |
| chr12_105716079_105710145_+5934-Ak7 | chr12 | Ak7 | 0 | 0 | 0.180646022 | 5935 | 396 |
| chr12_108167395_108157754_-9641-Setd3 | chr12 | Setd3 | 0 | 0.182309179 | 0 | 9642 | 805 |
| chr12_108515215_108510177_+5038-Eml1 | chr12 | Eml1 | 0.202796353 | 0 | 0 | 5039 | 512 |
| chr12_110554896_110545595_+9301-Ppp2r5c | chr12 | Ppp2r5c | 0 | 0 | 0.180646022 | 9302 | 447 |
| chr12_110561468_110545595_+15873-Ppp2r5c | chr12 | Ppp2r5c | 0.405592706 | 0 | 0 | 15874 | 618 |
| chr12_110779890_110779296_+594-Wdr20 | chr12 | Wdr20 | 0 | 0 | 0.180646022 | 595 | 595 |
| chr12_111255363_111237562_+17801-Traf3 | chr12 | Traf3 | 0 | 0.182309179 | 0 | 17802 | 974 |
| chr12_111647728_111592577_+55151-Mark3 | chr12 | Mark3 | 0.202796353 | 0 | 0 | 55152 | 1952 |
| chr12_111713418_111592577_+120841-Mark3 | chr12 | Mark3 | 0.202796353 | 0 | 0 | 120842 | 120842 |
| chr12_111789459_111778020_+11439-Klc1 | chr12 | Klc1 | 0.405592706 | 0 | 0 | 11440 | 826 |
| chr12_111795678_111793649_+2029-Klc1 | chr12 | Klc1 | 0.202796353 | 0.182309179 | 0.541938067 | 2030 | 182 |
| chr12_111806074_111778020_+28054-Klc1 | chr12 | Klc1 | 0 | 0.182309179 | 0 | 28055 | 5931 |
| chr12_112922057_112920072_-1985-Jag2 | chr12 | Jag2 | 0.608389058 | 0 | 0 | 1986 | 392 |
| chr12_112983849_112969265_-14584-Brf1 | chr12 | Brf1 | 0.202796353 | 0 | 0 | 14585 | 726 |
| chr12_116334256_116317830_+16426-Esyt2 | chr12 | Esyt2 | 0.405592706 | 0 | 0 | 16427 | 896 |
| chr12_13289980_13279388_+10592-Nbas | chr12 | Nbas | 0.202796353 | 0 | 0 | 10593 | 411 |
| chr12_13336410_13306904_+29506-Nbas | chr12 | Nbas | 0 | 0 | 0.180646022 | 29507 | 1454 |
| chr12_13453622_13322856_+130766-Nbas | chr12 | Nbas | 0 | 0 | 0.180646022 | 130767 | 3417 |
| chr12_13484044_13469841_+14203-Nbas | chr12 | Nbas | 0.405592706 | 0 | 0 | 14204 | 927 |
| chr12_13484044_13482386_+1658-Nbas | chr12 | Nbas | 1.41957447 | 0.546927538 | 1.625814201 | 1659 | 586 |
| chr12_16568519_16546721_-21798-Lpin1 | chr12 | Lpin1 | 0.202796353 | 0 | 0 | 21799 | 1500 |
| chr12_16568519_16558409_-10110-Lpin1 | chr12 | Lpin1 | 0 | 0.182309179 | 0 | 10111 | 1057 |
| chr12_16568519_16560982_-7537-Lpin1 | chr12 | Lpin1 | 0.608389058 | 2.370019332 | 1.264522156 | 7538 | 886 |
| chr12_16975558_16957226_+18332-Rock2 | chr12 | Rock2 | 0 | 0.182309179 | 0 | 18333 | 2276 |
| chr12_16978102_16957226_+20876-Rock2 | chr12 | Rock2 | 0 | 0.364618359 | 0 | 20877 | 2831 |
| chr12_16982747_16974842_+7905-Rock2 | chr12 | Rock2 | 0 | 0 | 0.180646022 | 7906 | 7906 |
| chr12_16984859_16957226_+27633-Rock2 | chr12 | Rock2 | 0 | 0.182309179 | 0 | 27634 | 3053 |
| chr12_17416961_17369466_+47495-Nol10 | chr12 | Nol10 | 0 | 0.182309179 | 0 | 47496 | 1085 |
| chr12_17424752_17373562_+51190-Nol10 | chr12 | Nol10 | 0 | 0.182309179 | 0 | 51191 | 1038 |
| chr12_25061278_25052797_+8481-na | chr12 | na | 0 | 0.09115459 | 0 | 8482 | 8482 |
| chr12_28867153_28859252_+7901-Tssc1 | chr12 | Tssc1 | 0.405592706 | 0 | 0 | 7902 | 703 |
| chr12_28867153_28862955_+4198-Tssc1 | chr12 | Tssc1 | 0 | 0.182309179 | 0 | 4199 | 566 |
| chr12_31802483_31790847_+11636-Cog5 | chr12 | Cog5 | 0.202796353 | 0 | 0 | 11637 | 357 |
| chr12_3496936_3496225_+711-Asxl2 | chr12 | Asxl2 | 0 | 0.182309179 | 0 | 712 | 712 |
| chr12_35092913_35078900_+14013-Snx13 | chr12 | Snx13 | 0 | 0 | 0.722584089 | 14014 | 751 |
| chr12_35107556_35085939_+21617-Snx13 | chr12 | Snx13 | 0 | 0.911545897 | 0.541938067 | 21618 | 1379 |
| chr12_38127383_38100364_+27019-Dgkb | chr12 | Dgkb | 0 | 0 | 0.180646022 | 27020 | 540 |
| chr12_3849739_3835027_+14712-Dnmt3a | chr12 | Dnmt3a | 0.202796353 | 0 | 0 | 14713 | 339 |
| chr12_3873428_3835027_+38401-Dnmt3a | chr12 | Dnmt3a | 0.202796353 | 0.546927538 | 0 | 38402 | 798 |
| chr12_3902814_3899193_+3621-Dnmt3a | chr12 | Dnmt3a | 0.405592706 | 0 | 0.180646022 | 3622 | 848 |
| chr12_40212418_40203919_-8499-Ifrd1 | chr12 | Ifrd1 | 0 | 0.182309179 | 0 | 8500 | 469 |
| chr12_40530260_40525829_+4431-na | chr12 | na | 0 | 0.182309179 | 0 | 4432 | 4432 |
| chr12_40640445_40621221_+19224-Dock4 | chr12 | Dock4 | 0.608389058 | 0 | 0 | 19225 | 512 |
| chr12_40649489_40621221_+28268-Dock4 | chr12 | Dock4 | 0.405592706 | 0 | 0 | 28269 | 664 |
| chr12_40649489_40626337_+23152-Dock4 | chr12 | Dock4 | 0 | 0 | 0.361292045 | 23153 | 539 |
| chr12_40649489_40631522_+17967-Dock4 | chr12 | Dock4 | 0 | 0.182309179 | 0 | 17968 | 483 |
| chr12_40676790_40621221_+55569-Dock4 | chr12 | Dock4 | 0 | 0 | 0.180646022 | 55570 | 1029 |
| chr12_4344502_4322868_-21634-Ncoa1 | chr12 | Ncoa1 | 0.202796353 | 0 | 0 | 21635 | 549 |
| chr12_44007841_43998336_-9505-na | chr12 | na | 0 | 0.182309179 | 0 | 9506 | 9506 |
| chr12_44299291_44283538_+15753-Pnpla8 | chr12 | Pnpla8 | 0 | 0.182309179 | 0 | 15754 | 794 |
| chr12_44299291_44288171_+11120-Pnpla8 | chr12 | Pnpla8 | 0 | 0.182309179 | 0 | 11121 | 477 |
| chr12_44305105_44288171_+16934-Pnpla8 | chr12 | Pnpla8 | 0 | 0.182309179 | 0 | 16935 | 672 |
| chr12_4673607_4613243_+60364-Itsn2 | chr12 | Itsn2 | 0 | 0.182309179 | 0 | 60365 | 3573 |
| chr12_4673607_4649983_+23624-Itsn2 | chr12 | Itsn2 | 0 | 0.182309179 | 0 | 23625 | 1594 |
| chr12_4735036_4715571_-19465-na | chr12 | na | 0 | 0 | 0.090323011 | 19466 | 19466 |
| chr12_4974279_4973968_+311-Atad2b | chr12 | Atad2b | 0.202796353 | 0 | 0 | 312 | 312 |
| chr12_4991058_4937390_+53668-Atad2b | chr12 | Atad2b | 0 | 0 | 0.180646022 | 53669 | 2244 |
| chr12_51357192_51351405_+5787-G2e3 | chr12 | G2e3 | 0.202796353 | 0 | 0 | 5788 | 532 |
| chr12_5137935_5137423_-512-Klhl29 | chr12 | Klhl29 | 0.811185411 | 0 | 0 | 513 | 513 |
| chr12_51400098_51384105_+15993-Scfd1 | chr12 | Scfd1 | 0 | 0 | 0.180646022 | 15994 | 794 |
| chr12_51629507_51619814_-9693-Strn3 | chr12 | Strn3 | 0 | 0.182309179 | 0 | 9694 | 897 |
| chr12_51661713_51627110_-34603-Strn3 | chr12 | Strn3 | 0 | 0 | 0.361292045 | 34604 | 1435 |
| chr12_51800972_51776496_-24476-Hectd1 | chr12 | Hectd1 | 0 | 0 | 0.361292045 | 24477 | 1958 |
| chr12_51948005_51905321_-42684-Heatr5a | chr12 | Heatr5a | 0.202796353 | 0 | 0 | 42685 | 2220 |
| chr12_51956270_51950929_-5341-Heatr5a | chr12 | Heatr5a | 0 | 0.182309179 | 0 | 5342 | 742 |
| chr12_52542636_52516079_+26557-Arhgap5 | chr12 | Arhgap5 | 0.202796353 | 0 | 0 | 26558 | 4037 |
| chr12_54765679_54751782_-13897-Snx6 | chr12 | Snx6 | 0 | 0.182309179 | 0 | 13898 | 651 |
| chr12_55414498_55407469_+7029-Psma6 | chr12 | Psma6 | 0 | 0 | 0.180646022 | 7030 | 607 |
| chr12_55709707_55683907_-25800-Ralgapa1 | chr12 | Ralgapa1 | 0 | 0.182309179 | 0 | 25801 | 1371 |
| chr12_55709707_55708994_-713-Ralgapa1 | chr12 | Ralgapa1 | 0 | 0 | 0.180646022 | 714 | 293 |
| chr12_59005316_58997136_-8180-Sec23a | chr12 | Sec23a | 0 | 0.182309179 | 0 | 8181 | 607 |
| chr12_59172592_59136064_+36528-Ctage5 | chr12 | Ctage5 | 0 | 0.364618359 | 0 | 36529 | 1412 |
| chr12_59188545_59136064_+52481-Ctage5 | chr12 | Ctage5 | 0.405592706 | 0 | 0 | 52482 | 2194 |
| chr12_65020400_65006875_+13525-Fam179b | chr12 | Fam179b | 0 | 0.182309179 | 0 | 13526 | 1138 |
| chr12_65122090_65102562_+19528-Fancm | chr12 | Fancm | 0 | 0 | 0.361292045 | 19529 | 3126 |
| chr12_69610826_69603476_-7350-Sos2 | chr12 | Sos2 | 0 | 0.546927538 | 0 | 7351 | 610 |
| chr12_70051250_70038539_-12711-Nin | chr12 | Nin | 0 | 0 | 0.180646022 | 12712 | 3483 |
| chr12_71194466_71184423_+10043-2700049A03Rik | chr12 | 2700049A03Rik | 0 | 0 | 0.180646022 | 10044 | 1194 |
| chr12_73181924_73168000_+13924-Mnat1 | chr12 | Mnat1 | 0 | 0 | 0.541938067 | 13925 | 472 |
| chr12_73188265_73168000_+20265-Mnat1 | chr12 | Mnat1 | 0.405592706 | 0 | 0 | 20266 | 598 |
| chr12_73219142_73168000_+51142-Mnat1 | chr12 | Mnat1 | 0 | 0 | 0.541938067 | 51143 | 720 |
| chr12_73928298_73926557_+1741-Hif1a | chr12 | Hif1a | 0 | 0.364618359 | 0 | 1742 | 422 |
| chr12_75343537_75338163_-5374-na | chr12 | na | 0 | 0.182309179 | 0 | 5375 | 5375 |
| chr12_75343537_75338169_-5368-na | chr12 | na | 0 | 0.182309179 | 0 | 5369 | 5369 |
| chr12_77365359_77323845_+41514-Fut8 | chr12 | Fut8 | 0.608389058 | 0 | 0 | 41515 | 806 |
| chr12_78494057_78492038_+2019-Gphn | chr12 | Gphn | 0 | 0.182309179 | 0 | 2020 | 381 |
| chr12_78590671_78504617_+86054-Gphn | chr12 | Gphn | 0.202796353 | 0 | 0.361292045 | 86055 | 478 |
| chr12_78824872_78819204_+5668-Mpp5 | chr12 | Mpp5 | 0.608389058 | 0 | 0 | 5669 | 424 |
| chr12_79507838_79482529_+25309-na | chr12 | na | 0 | 0.09115459 | 0 | 25310 | 25310 |
| chr12_80376731_80348320_-28411-Dcaf5 | chr12 | Dcaf5 | 0.405592706 | 0.182309179 | 0.361292045 | 28412 | 409 |
| chr12_80404698_80348320_-56378-Dcaf5 | chr12 | Dcaf5 | 0.608389058 | 0.182309179 | 0 | 56379 | 860 |
| chr12_80404698_80364004_-40694-Dcaf5 | chr12 | Dcaf5 | 0 | 0.182309179 | 0.361292045 | 40695 | 732 |
| chr12_80404698_80397653_-7045-Dcaf5 | chr12 | Dcaf5 | 0.202796353 | 0 | 0 | 7046 | 451 |
| chr12_80497013_80484246_+12767-Exd2 | chr12 | Exd2 | 0 | 0.182309179 | 0 | 12768 | 1292 |
| chr12_81316660_81314260_-2400-Slc8a3 | chr12 | Slc8a3 | 0 | 0.182309179 | 0.180646022 | 2401 | 2401 |
| chr12_81359344_81314260_-45084-Slc8a3 | chr12 | Slc8a3 | 0.202796353 | 0 | 0 | 45085 | 45085 |
| chr12_81948415_81928242_+20173-Pcnx | chr12 | Pcnx | 0 | 0.364618359 | 0 | 20174 | 467 |
| chr12_81956263_81928242_+28021-Pcnx | chr12 | Pcnx | 0.202796353 | 0 | 0 | 28022 | 872 |
| chr12_81956263_81946931_+9332-Pcnx | chr12 | Pcnx | 0.202796353 | 0 | 0 | 9333 | 554 |
| chr12_82372542_82357233_+15309-Sipa1l1 | chr12 | Sipa1l1 | 0.202796353 | 0 | 0 | 15310 | 495 |
| chr12_8238615_8227220_+11395-Ldah | chr12 | Ldah | 0.202796353 | 0 | 0.180646022 | 11396 | 317 |
| chr12_8268598_8238443_+30155-Ldah | chr12 | Ldah | 0 | 0.182309179 | 0 | 30156 | 408 |
| chr12_8275890_8232666_+43224-Ldah | chr12 | Ldah | 0 | 0.364618359 | 0 | 43225 | 43225 |
| chr12_83575136_83574632_-504-Zfyve1 | chr12 | Zfyve1 | 0.405592706 | 0 | 0 | 505 | 505 |
| chr12_83777495_83776354_+1141-Papln | chr12 | Papln | 0.405592706 | 0 | 0 | 1142 | 533 |
| chr12_84127017_84121155_+5862-Dnal1 | chr12 | Dnal1 | 0.202796353 | 0 | 0 | 5863 | 333 |
| chr12_84436918_84432227_-4691-Aldh6a1 | chr12 | Aldh6a1 | 0 | 0 | 0.180646022 | 4692 | 973 |
| chr12_84927410_84920069_-7341-Arel1 | chr12 | Arel1 | 0 | 0 | 0.361292045 | 7342 | 1391 |
| chr12_85030887_85028770_+2117-Ylpm1 | chr12 | Ylpm1 | 0 | 0 | 0.180646022 | 2118 | 2118 |
| chr12_85269474_85266152_-3322-Mlh3 | chr12 | Mlh3 | 0 | 0.182309179 | 0 | 3323 | 3323 |
| chr12_85311446_85307930_-3516-Nek9 | chr12 | Nek9 | 0 | 0.182309179 | 0 | 3517 | 502 |
| chr12_85327499_85320714_-6785-Nek9 | chr12 | Nek9 | 0 | 0.364618359 | 0 | 6786 | 420 |
| chr12_85879475_85863564_+15911-Ttll5 | chr12 | Ttll5 | 0.202796353 | 0.182309179 | 0 | 15912 | 457 |
| chr12_85892210_85863564_+28646-Ttll5 | chr12 | Ttll5 | 0 | 0.182309179 | 0 | 28647 | 696 |
| chr12_85939510_85925640_+13870-Ttll5 | chr12 | Ttll5 | 0 | 0 | 0.180646022 | 13871 | 1041 |
| chr12_85956720_85889159_+67561-Ttll5 | chr12 | Ttll5 | 0 | 0.364618359 | 0 | 67562 | 2329 |
| chr12_86267183_86256868_+10315-Gpatch2l | chr12 | Gpatch2l | 0 | 0.364618359 | 0 | 10316 | 430 |
| chr12_86269061_86256481_+12580-Gpatch2l | chr12 | Gpatch2l | 0 | 0.273463769 | 0.361292045 | 12581 | 12581 |
| chr12_86269061_86256868_+12193-Gpatch2l | chr12 | Gpatch2l | 2.636352587 | 0.729236718 | 0.903230112 | 12194 | 531 |
| chr12_86269061_86260577_+8484-Gpatch2l | chr12 | Gpatch2l | 0.709787235 | 1.276164256 | 1.354845167 | 8485 | 466 |
| chr12_86281543_86256868_+24675-Gpatch2l | chr12 | Gpatch2l | 0.202796353 | 0 | 0 | 24676 | 626 |
| chr12_86721380_86720230_-1150-Angel1 | chr12 | Angel1 | 0.608389058 | 0.182309179 | 0 | 1151 | 504 |
| chr12_87251752_87244455_-7297-na | chr12 | na | 0 | 0 | 0.180646022 | 7298 | 7298 |
| chr12_87253258_87252635_-623-Vipas39 | chr12 | Vipas39 | 0 | 0 | 0.090323011 | 624 | 127 |
| chr12_91370196_91364360_-5836-Cep128 | chr12 | Cep128 | 0.405592706 | 0 | 0 | 5837 | 384 |
| chr12_91743878_91740471_-3407-Ston2 | chr12 | Ston2 | 0 | 0.364618359 | 0 | 3408 | 549 |
| chr12_98705180_98698831_-6349-Ptpn21 | chr12 | Ptpn21 | 0.405592706 | 0 | 0 | 6350 | 345 |
| chr12_98785741_98783582_+2159-Zc3h14 | chr12 | Zc3h14 | 0.405592706 | 0 | 0 | 2160 | 454 |
| chr12_98946613_98942218_+4395-Ttc8 | chr12 | Ttc8 | 0 | 0 | 0.180646022 | 4396 | 480 |
| chr12_98964607_98906328_+58279-Ttc8 | chr12 | Ttc8 | 0 | 0.182309179 | 0 | 58280 | 58280 |
| chr12_99291301_99209461_-81840-Foxn3 | chr12 | Foxn3 | 0 | 0 | 0.180646022 | 81841 | 237 |
| chr12_99388917_99209461_-179456-Foxn3 | chr12 | Foxn3 | 0 | 0 | 0.541938067 | 179457 | 907 |
| chr12_99388917_99289870_-99047-Foxn3 | chr12 | Foxn3 | 0.405592706 | 0 | 0 | 99048 | 99048 |
| chr12_99388917_99291237_-97680-Foxn3 | chr12 | Foxn3 | 0.608389058 | 0.546927538 | 0 | 97681 | 735 |
| chr13_100042285_100023600_-18685-Bdp1 | chr13 | Bdp1 | 0 | 0 | 0.361292045 | 18686 | 1721 |
| chr13_101709742_101701447_-8295-Pik3r1 | chr13 | Pik3r1 | 0.202796353 | 0.546927538 | 0.180646022 | 8296 | 582 |
| chr13_101758039_101757326_-713-Pik3r1 | chr13 | Pik3r1 | 0.608389058 | 0.182309179 | 0 | 714 | 714 |
| chr13_101762530_101757326_-5204-Pik3r1 | chr13 | Pik3r1 | 0 | 0.182309179 | 0 | 5205 | 5205 |
| chr13_102853919_101757326_-1096593-Mast4 | chr13 | Mast4 | 0.405592706 | 0 | 0 | 1096594 | 1096594 |
| chr13_103884260_103824720_-59540-Erbb2ip | chr13 | Erbb2ip | 0.202796353 | 0 | 0 | 59541 | 3888 |
| chr13_104400006_104375642_+24364-Adamts6 | chr13 | Adamts6 | 0.202796353 | 0 | 0 | 24365 | 460 |
| chr13_104479675_104444053_+35622-Adamts6 | chr13 | Adamts6 | 0 | 0.182309179 | 0 | 35623 | 1129 |
| chr13_106993942_106990038_-3904-Kif2a | chr13 | Kif2a | 0 | 0 | 0.541938067 | 3905 | 267 |
| chr13_109614457_109550009_+64448-na | chr13 | na | 0 | 0.182309179 | 0 | 64449 | 64449 |
| chr13_109772680_109614419_+158261-na | chr13 | na | 0 | 0.09115459 | 0 | 158262 | 158262 |
| chr13_109772680_109740387_+32293-Pde4d | chr13 | Pde4d | 0 | 0.729236718 | 0 | 32294 | 353 |
| chr13_109772680_109769534_+3146-Pde4d | chr13 | Pde4d | 0 | 0.182309179 | 0 | 3147 | 124 |
| chr13_109903689_109769534_+134155-Pde4d | chr13 | Pde4d | 0 | 0 | 0.180646022 | 134156 | 237 |
| chr13_109939361_109933868_+5493-Pde4d | chr13 | Pde4d | 0.202796353 | 0 | 0 | 5494 | 631 |
| chr13_111768593_111750443_-18150-Map3k1 | chr13 | Map3k1 | 0 | 0 | 0.541938067 | 18151 | 3312 |
| chr13_112498889_112493589_+5300-Il6st | chr13 | Il6st | 0.405592706 | 0 | 0 | 5301 | 867 |
| chr13_112963356_112960323_+3033-Dhx29 | chr13 | Dhx29 | 0 | 0 | 0.180646022 | 3034 | 532 |
| chr13_114216049_114204072_+11977-Gm41072 | chr13 | Gm41072 | 0.405592706 | 0 | 0 | 11978 | 11978 |
| chr13_116895781_116876531_-19250-Parp8 | chr13 | Parp8 | 0.202796353 | 0 | 0 | 19251 | 1117 |
| chr13_116895781_116893000_-2781-Parp8 | chr13 | Parp8 | 0 | 0 | 0.541938067 | 2782 | 832 |
| chr13_116902833_116893000_-9833-na | chr13 | na | 0 | 0.09115459 | 0 | 9834 | 9834 |
| chr13_116902856_116876531_-26325-Parp8 | chr13 | Parp8 | 0.202796353 | 0 | 0 | 26326 | 1184 |
| chr13_116902856_116893000_-9856-Parp8 | chr13 | Parp8 | 0.202796353 | 0.182309179 | 0.361292045 | 9857 | 899 |
| chr13_118388780_118386652_-2128-Mrps30 | chr13 | Mrps30 | 0 | 0.546927538 | 0 | 2129 | 2129 |
| chr13_119404754_119381676_-23078-Nnt | chr13 | Nnt | 0.202796353 | 0 | 0 | 23079 | 1016 |
| chr13_119790287_119775767_-14520-Zfp131 | chr13 | Zfp131 | 0 | 0.182309179 | 0 | 14521 | 1157 |
| chr13_12205357_12188041_-17316-Mtr | chr13 | Mtr | 0 | 0 | 0.180646022 | 17317 | 1306 |
| chr13_12250691_12204743_-45948-Mtr | chr13 | Mtr | 0.405592706 | 0.911545897 | 0 | 45949 | 2348 |
| chr13_12250691_12225462_-25229-Mtr | chr13 | Mtr | 0 | 0.182309179 | 0 | 25230 | 1449 |
| chr13_12250691_12239649_-11042-Mtr | chr13 | Mtr | 0.405592706 | 0 | 0.180646022 | 11043 | 681 |
| chr13_12310961_12288507_-22454-Actn2 | chr13 | Actn2 | 0 | 0 | 0.180646022 | 22455 | 1280 |
| chr13_12459316_12454735_-4581-Lgals8 | chr13 | Lgals8 | 0 | 0.364618359 | 0.541938067 | 4582 | 443 |
| chr13_13991552_13987353_+4199-B3galnt2 | chr13 | B3galnt2 | 0 | 0.546927538 | 0 | 4200 | 374 |
| chr13_14615743_14615396_-347-na | chr13 | na | 0 | 0 | 0.361292045 | 348 | 348 |
| chr13_17801457_17750257_-51200-Cdk13 | chr13 | Cdk13 | 0 | 0.182309179 | 0 | 51201 | 51201 |
| chr13_17897102_17893122_-3980-Rala | chr13 | Rala | 0 | 0.182309179 | 0 | 3981 | 359 |
| chr13_20589726_20449470_+140256-Elmo1 | chr13 | Elmo1 | 0 | 0.364618359 | 0 | 140257 | 605 |
| chr13_24689509_24665409_+24100-Fam65b | chr13 | Fam65b | 0.202796353 | 0 | 0 | 24101 | 938 |
| chr13_29357464_29354641_-2823-na | chr13 | na | 0 | 0 | 0.361292045 | 2824 | 2824 |
| chr13_29552395_29354630_-197765-Cdkal1 | chr13 | Cdkal1 | 0 | 0.182309179 | 0.361292045 | 197766 | 806 |
| chr13_29552395_29517413_-34982-Cdkal1 | chr13 | Cdkal1 | 0 | 0.182309179 | 0.361292045 | 34983 | 313 |
| chr13_29575775_29474529_-101246-Cdkal1 | chr13 | Cdkal1 | 0.202796353 | 0 | 0 | 101247 | 101247 |
| chr13_29579314_29517413_-61901-Cdkal1 | chr13 | Cdkal1 | 0.608389058 | 0 | 0 | 61902 | 61902 |
| chr13_29596988_29517413_-79575-Cdkal1 | chr13 | Cdkal1 | 0 | 0 | 0.451615056 | 79576 | 79576 |
| chr13_29625724_29625621_-103-Cdkal1 | chr13 | Cdkal1 | 0 | 0 | 0.180646022 | 104 | 104 |
| chr13_29854941_29517413_-337528-Cdkal1 | chr13 | Cdkal1 | 0.405592706 | 0 | 0 | 337529 | 1284 |
| chr13_29854941_29846228_-8713-Cdkal1 | chr13 | Cdkal1 | 0 | 0.729236718 | 0 | 8714 | 327 |
| chr13_30900903_30864831_-36072-Exoc2 | chr13 | Exoc2 | 0.202796353 | 0 | 0 | 36073 | 1165 |
| chr13_30940770_30925723_-15047-Exoc2 | chr13 | Exoc2 | 0.202796353 | 0 | 0.180646022 | 15048 | 704 |
| chr13_32717620_32708880_-8740-Mylk4 | chr13 | Mylk4 | 0 | 0.182309179 | 0 | 8741 | 499 |
| chr13_32717620_32712758_-4862-Mylk4 | chr13 | Mylk4 | 0.405592706 | 0 | 0 | 4863 | 432 |
| chr13_32720601_32708880_-11721-Mylk4 | chr13 | Mylk4 | 0.202796353 | 0.364618359 | 0.361292045 | 11722 | 641 |
| chr13_32724884_32708880_-16004-Mylk4 | chr13 | Mylk4 | 0.608389058 | 0 | 0 | 16005 | 951 |
| chr13_32724884_32712758_-12126-Mylk4 | chr13 | Mylk4 | 0 | 0 | 0.361292045 | 12127 | 884 |
| chr13_32724884_32715859_-9025-Mylk4 | chr13 | Mylk4 | 0 | 0.182309179 | 0.180646022 | 9026 | 805 |
| chr13_32727132_32708880_-18252-Mylk4 | chr13 | Mylk4 | 0.202796353 | 0.182309179 | 0 | 18253 | 1241 |
| chr13_32727132_32712758_-14374-Mylk4 | chr13 | Mylk4 | 0 | 0.182309179 | 0 | 14375 | 1174 |
| chr13_32727132_32715859_-11273-Mylk4 | chr13 | Mylk4 | 0.202796353 | 0.182309179 | 0 | 11274 | 1095 |
| chr13_32729154_32708880_-20274-Mylk4 | chr13 | Mylk4 | 0 | 0 | 0.180646022 | 20275 | 1741 |
| chr13_32729154_32712758_-16396-Mylk4 | chr13 | Mylk4 | 0 | 0.364618359 | 0 | 16397 | 1674 |
| chr13_34319104_34298997_-20107-Slc22a23 | chr13 | Slc22a23 | 0 | 0.364618359 | 0 | 20108 | 20108 |
| chr13_35816575_35790101_+26474-Cdyl | chr13 | Cdyl | 0 | 0.364618359 | 0.180646022 | 26475 | 742 |
| chr13_35858248_35815909_+42339-Cdyl | chr13 | Cdyl | 0.405592706 | 0 | 0 | 42340 | 1097 |
| chr13_3600334_3595497_-4837-Fam208b | chr13 | Fam208b | 0 | 0.182309179 | 0 | 4838 | 592 |
| chr13_36246494_36204509_+41985-Fars2 | chr13 | Fars2 | 0 | 0 | 0.180646022 | 41986 | 925 |
| chr13_36342097_36336857_+5240-na | chr13 | na | 0 | 0.09115459 | 0 | 5241 | 5241 |
| chr13_36918635_36892806_-25829-F13a1 | chr13 | F13a1 | 0 | 0 | 0.180646022 | 25830 | 796 |
| chr13_37899787_37858985_+40802-Rreb1 | chr13 | Rreb1 | 0.202796353 | 0 | 0 | 40803 | 772 |
| chr13_37916595_37916022_+573-Rreb1 | chr13 | Rreb1 | 0 | 0 | 0.361292045 | 574 | 276 |
| chr13_38485936_38469614_+16322-Bmp6 | chr13 | Bmp6 | 0 | 0.182309179 | 0 | 16323 | 540 |
| chr13_38943166_38934738_-8428-Slc35b3 | chr13 | Slc35b3 | 0 | 0 | 0.180646022 | 8429 | 509 |
| chr13_38955936_38954109_-1827-Slc35b3 | chr13 | Slc35b3 | 0 | 0.729236718 | 0 | 1828 | 410 |
| chr13_43096806_43094749_+2057-Phactr1 | chr13 | Phactr1 | 0.202796353 | 0 | 0 | 2058 | 405 |
| chr13_43461823_43434814_-27009-Ranbp9 | chr13 | Ranbp9 | 0 | 0 | 0.361292045 | 27010 | 333 |
| chr13_44982873_44930996_-51877-Dtnbp1 | chr13 | Dtnbp1 | 0 | 0 | 0.180646022 | 51878 | 445 |
| chr13_44996006_44970046_-25960-Dtnbp1 | chr13 | Dtnbp1 | 0.405592706 | 0 | 0 | 25961 | 25961 |
| chr13_45580326_45566573_-13753-Atxn1 | chr13 | Atxn1 | 0.202796353 | 0 | 0 | 13754 | 13754 |
| chr13_45735178_45697821_-37357-Atxn1 | chr13 | Atxn1 | 0 | 0 | 0.361292045 | 37358 | 189 |
| chr13_45795637_45795509_-128-Atxn1 | chr13 | Atxn1 | 0 | 0.911545897 | 0 | 129 | 129 |
| chr13_45852456_45795509_-56947-Atxn1 | chr13 | Atxn1 | 0 | 0.09115459 | 0.180646022 | 56948 | 328 |
| chr13_45956575_45566573_-390002-Atxn1 | chr13 | Atxn1 | 0.202796353 | 0 | 0 | 390003 | 2718 |
| chr13_45956575_45697821_-258754-Atxn1 | chr13 | Atxn1 | 0 | 0 | 0.361292045 | 258755 | 710 |
| chr13_45956575_45743835_-212740-Atxn1 | chr13 | Atxn1 | 0 | 0 | 0.180646022 | 212741 | 212741 |
| chr13_45956575_45795509_-161066-Atxn1 | chr13 | Atxn1 | 0.811185411 | 0 | 0.722584089 | 161067 | 521 |
| chr13_45956575_45852328_-104247-Atxn1 | chr13 | Atxn1 | 0.811185411 | 0 | 0.541938067 | 104248 | 392 |
| chr13_45956575_45956455_-120-Atxn1 | chr13 | Atxn1 | 0 | 0.182309179 | 0 | 121 | 121 |
| chr13_46640122_46615250_+24872-Cap2 | chr13 | Cap2 | 0 | 0.182309179 | 0 | 24873 | 676 |
| chr13_46778993_46760263_-18730-Kif13a | chr13 | Kif13a | 0 | 0 | 0.180646022 | 18731 | 18731 |
| chr13_46778993_46761273_-17720-Kif13a | chr13 | Kif13a | 0.202796353 | 0 | 0.541938067 | 17721 | 1215 |
| chr13_46802729_46780626_-22103-Kif13a | chr13 | Kif13a | 0.405592706 | 0 | 0 | 22104 | 1475 |
| chr13_46802729_46785246_-17483-Kif13a | chr13 | Kif13a | 0 | 0 | 0.180646022 | 17484 | 1436 |
| chr13_46832816_46823949_-8867-Kif13a | chr13 | Kif13a | 0 | 0.182309179 | 0 | 8868 | 610 |
| chr13_48506993_48479843_-27150-Zfp169 | chr13 | Zfp169 | 0.202796353 | 0 | 0 | 27151 | 27151 |
| chr13_48902307_48885273_-17034-Fam120a | chr13 | Fam120a | 0.202796353 | 0 | 0 | 17035 | 1136 |
| chr13_49085541_49081904_-3637-Wnk2 | chr13 | Wnk2 | 0 | 0 | 0.361292045 | 3638 | 492 |
| chr13_49102962_49100963_-1999-Wnk2 | chr13 | Wnk2 | 0 | 0.182309179 | 0 | 2000 | 394 |
| chr13_50276670_50273334_+3336-Gm31126 | chr13 | Gm31126 | 0.202796353 | 0 | 0 | 3337 | 410 |
| chr13_51679953_51678834_+1119-Secisbp2 | chr13 | Secisbp2 | 0.608389058 | 0.546927538 | 0 | 1120 | 382 |
| chr13_51682633_51679733_+2900-Secisbp2 | chr13 | Secisbp2 | 0 | 0.182309179 | 0 | 2901 | 2258 |
| chr13_52919266_52887241_-32025-Auh | chr13 | Auh | 0.202796353 | 0 | 0.180646022 | 32026 | 547 |
| chr13_54529223_54523970_+5253-Simc1 | chr13 | Simc1 | 0 | 0 | 0.180646022 | 5254 | 3021 |
| chr13_54655577_54641419_+14158-Faf2 | chr13 | Faf2 | 0 | 0 | 0.180646022 | 14159 | 879 |
| chr13_54655577_54652043_+3534-Faf2 | chr13 | Faf2 | 0.202796353 | 0 | 0.541938067 | 3535 | 350 |
| chr13_54656425_54638250_+18175-Faf2 | chr13 | Faf2 | 0 | 0 | 0.180646022 | 18176 | 1092 |
| chr13_55069807_55034889_-34918-Uimc1 | chr13 | Uimc1 | 0 | 0 | 0.361292045 | 34919 | 678 |
| chr13_55248077_55245518_+2559-Nsd1 | chr13 | Nsd1 | 0.202796353 | 0.364618359 | 0.180646022 | 2560 | 2560 |
| chr13_55263442_55233895_+29547-Nsd1 | chr13 | Nsd1 | 0 | 0 | 0.180646022 | 29548 | 3375 |
| chr13_55451836_55450928_+908-Grk6 | chr13 | Grk6 | 0 | 0.182309179 | 0 | 909 | 392 |
| chr13_55556225_55552962_-3263-Fam193b | chr13 | Fam193b | 0.202796353 | 0 | 0 | 3264 | 863 |
| chr13_55936570_55929539_+7031-na | chr13 | na | 0 | 0.182309179 | 0 | 7032 | 7032 |
| chr13_56039461_55929539_+109922-na | chr13 | na | 0 | 0.182309179 | 0 | 109923 | 109923 |
| chr13_58199070_58191722_-7348-Ubqln1 | chr13 | Ubqln1 | 0 | 0.729236718 | 0.180646022 | 7349 | 934 |
| chr13_59462173_59460469_-1704-Agtpbp1 | chr13 | Agtpbp1 | 0 | 0 | 0.361292045 | 1705 | 338 |
| chr13_59504310_59473688_-30622-Agtpbp1 | chr13 | Agtpbp1 | 0 | 0.182309179 | 0 | 30623 | 1824 |
| chr13_59514295_59495333_-18962-Agtpbp1 | chr13 | Agtpbp1 | 0 | 0 | 0.180646022 | 18963 | 1611 |
| chr13_59544451_59460469_-83982-Agtpbp1 | chr13 | Agtpbp1 | 0 | 0 | 0.361292045 | 83983 | 3662 |
| chr13_59544451_59495333_-49118-Agtpbp1 | chr13 | Agtpbp1 | 0 | 0.364618359 | 0 | 49119 | 2494 |
| chr13_59544451_59512324_-32127-Agtpbp1 | chr13 | Agtpbp1 | 0 | 0 | 0.180646022 | 32128 | 1270 |
| chr13_59555609_59446917_-108692-Agtpbp1 | chr13 | Agtpbp1 | 0 | 0.364618359 | 0 | 108693 | 108693 |
| chr13_59800716_59789848_-10868-Zcchc6 | chr13 | Zcchc6 | 0 | 0.911545897 | 1.625814201 | 10869 | 1667 |
| chr13_60854949_60853100_-1849-4930486L24Rik | chr13 | 4930486L24Rik | 0 | 0 | 0.722584089 | 1850 | 632 |
| chr13_63210166_63190479_+19687-2010111I01Rik | chr13 | 2010111I01Rik | 0 | 0.182309179 | 0 | 19688 | 316 |
| chr13_63872927_63858231_+14696-Ercc6l2 | chr13 | Ercc6l2 | 0 | 0.182309179 | 0 | 14697 | 1562 |
| chr13_64247318_64209107_-38211-Cdc14b | chr13 | Cdc14b | 0.202796353 | 0 | 0 | 38212 | 1183 |
| chr13_64248466_64243634_-4832-na | chr13 | na | 0 | 0.182309179 | 0 | 4833 | 4833 |
| chr13_64248499_64225572_-22927-Cdc14b | chr13 | Cdc14b | 0 | 0 | 0.090323011 | 22928 | 432 |
| chr13_64252756_64209107_-43649-Cdc14b | chr13 | Cdc14b | 0 | 0 | 0.361292045 | 43650 | 43650 |
| chr13_6556702_6552634_+4068-Pitrm1 | chr13 | Pitrm1 | 0 | 0 | 0.541938067 | 4069 | 632 |
| chr13_67494729_67481170_-13559-Zfp874b | chr13 | Zfp874b | 0 | 0.182309179 | 0 | 13560 | 13560 |
| chr13_67711007_67683403_-27604-Zfp65 | chr13 | Zfp65 | 0 | 0.729236718 | 0 | 27605 | 27605 |
| chr13_68796679_68791278_-5401-Adcy2 | chr13 | Adcy2 | 0 | 0.182309179 | 0 | 5402 | 5402 |
| chr13_68796679_68796530_-149-Adcy2 | chr13 | Adcy2 | 0.202796353 | 0 | 0 | 150 | 150 |
| chr13_70615113_70592573_-22540-Ice1 | chr13 | Ice1 | 0 | 0.182309179 | 0 | 22541 | 6010 |
| chr13_74675906_74671308_+4598-Erap1 | chr13 | Erap1 | 0 | 0.182309179 | 0.180646022 | 4599 | 911 |
| chr13_75615048_75611887_-3161-na | chr13 | na | 0 | 0 | 0.180646022 | 3162 | 3162 |
| chr13_75913454_75892906_-20548-Rhobtb3 | chr13 | Rhobtb3 | 1.41957447 | 1.640782614 | 1.625814201 | 20549 | 712 |
| chr13_75917847_75892906_-24941-Rhobtb3 | chr13 | Rhobtb3 | 0.202796353 | 0.729236718 | 0 | 24942 | 867 |
| chr13_75939638_75892906_-46732-Rhobtb3 | chr13 | Rhobtb3 | 0 | 0.182309179 | 0 | 46733 | 1054 |
| chr13_76113113_76106230_+6883-Ttc37 | chr13 | Ttc37 | 0 | 0 | 0.180646022 | 6884 | 552 |
| chr13_76136474_76127497_+8977-Ttc37 | chr13 | Ttc37 | 0 | 0 | 0.180646022 | 8978 | 1413 |
| chr13_76688630_76641712_+46918-Mctp1 | chr13 | Mctp1 | 0 | 0.364618359 | 0 | 46919 | 258 |
| chr13_76759820_76641712_+118108-Mctp1 | chr13 | Mctp1 | 0 | 0.911545897 | 0 | 118109 | 899 |
| chr13_76806262_76641712_+164550-Mctp1 | chr13 | Mctp1 | 0 | 0.182309179 | 0 | 164551 | 1180 |
| chr13_77193223_77183601_+9622-2210408I21Rik | chr13 | 2210408I21Rik | 0 | 0 | 0.180646022 | 9623 | 839 |
| chr13_83323951_83315192_-8759-Gm33217 | chr13 | Gm33217 | 0.202796353 | 0 | 0 | 8760 | 8760 |
| chr13_83348892_83321068_-27824-Gm33217 | chr13 | Gm33217 | 0 | 0.364618359 | 0 | 27825 | 27825 |
| chr13_83362820_83323767_-39053-na | chr13 | na | 0 | 0.182309179 | 0 | 39054 | 39054 |
| chr13_83362820_83347183_-15637-Gm33217 | chr13 | Gm33217 | 0 | 0 | 0.180646022 | 15638 | 15638 |
| chr13_83362820_83347183_-15637-na | chr13 | na | 0 | 0.182309179 | 0 | 15638 | 15638 |
| chr13_83440017_83427844_-12173-Gm41010 | chr13 | Gm41010 | 0 | 0.182309179 | 0 | 12174 | 12174 |
| chr13_83446420_83427844_-18576-Gm41010 | chr13 | Gm41010 | 0 | 0.182309179 | 0 | 18577 | 18577 |
| chr13_83446420_83439903_-6517-Gm33274 | chr13 | Gm33274 | 0 | 0.182309179 | 0.180646022 | 6518 | 6518 |
| chr13_83452301_83427844_-24457-na | chr13 | na | 0 | 0 | 0.180646022 | 24458 | 24458 |
| chr13_83452301_83439903_-12398-Gm41010 | chr13 | Gm41010 | 0 | 0.273463769 | 0 | 12399 | 12399 |
| chr13_83592981_83575538_+17443-Mef2c | chr13 | Mef2c | 0 | 0 | 0.180646022 | 17444 | 397 |
| chr13_83635469_83625470_+9999-Mef2c | chr13 | Mef2c | 0.202796353 | 0 | 0.541938067 | 10000 | 373 |
| chr13_8837023_8835297_-1726-Wdr37 | chr13 | Wdr37 | 0.608389058 | 0 | 0 | 1727 | 383 |
| chr13_8849669_8819863_-29806-Wdr37 | chr13 | Wdr37 | 0.202796353 | 0 | 0 | 29807 | 963 |
| chr13_8849669_8835297_-14372-Wdr37 | chr13 | Wdr37 | 0 | 0 | 0.361292045 | 14373 | 713 |
| chr13_8861265_8819863_-41402-Wdr37 | chr13 | Wdr37 | 0.202796353 | 0 | 0 | 41403 | 1399 |
| chr13_8861265_8844787_-16478-Wdr37 | chr13 | Wdr37 | 0.202796353 | 0 | 0 | 16479 | 689 |
| chr13_91040988_91022570_-18418-Atg10 | chr13 | Atg10 | 0 | 0 | 0.090323011 | 18419 | 237 |
| chr13_9145518_9122108_+23410-Larp4b | chr13 | Larp4b | 0 | 0 | 0.180646022 | 23411 | 690 |
| chr13_92300358_92256695_-43663-Msh3 | chr13 | Msh3 | 0 | 0 | 0.180646022 | 43664 | 975 |
| chr13_92312686_92293474_-19212-Msh3 | chr13 | Msh3 | 0 | 0 | 0.090323011 | 19213 | 19213 |
| chr13_92312686_92299153_-13533-Msh3 | chr13 | Msh3 | 0 | 0 | 0.541938067 | 13534 | 423 |
| chr13_92342412_92285877_-56535-Msh3 | chr13 | Msh3 | 0 | 0.182309179 | 0 | 56536 | 723 |
| chr13_92345220_92299153_-46067-Msh3 | chr13 | Msh3 | 0.202796353 | 0 | 0 | 46068 | 855 |
| chr13_93349297_93341756_+7541-Homer1 | chr13 | Homer1 | 0 | 0 | 0.361292045 | 7542 | 382 |
| chr13_93393108_93387524_+5584-Homer1 | chr13 | Homer1 | 0.202796353 | 0.364618359 | 0.361292045 | 5585 | 349 |
| chr13_93940708_93939227_+1481-Arsb | chr13 | Arsb | 0 | 0 | 0.180646022 | 1482 | 366 |
| chr13_94233180_94224895_-8285-Scamp1 | chr13 | Scamp1 | 0.608389058 | 0.546927538 | 0 | 8286 | 497 |
| chr13_94418143_94394719_+23424-Ap3b1 | chr13 | Ap3b1 | 0 | 0 | 0.180646022 | 23425 | 582 |
| chr13_95005842_94995250_+10592-Wdr41 | chr13 | Wdr41 | 0 | 0.182309179 | 0 | 10593 | 356 |
| chr13_9576918_9560671_+16247-Dip2c | chr13 | Dip2c | 0.202796353 | 0 | 0 | 16248 | 934 |
| chr13_96504642_96489182_-15460-Polk | chr13 | Polk | 0.405592706 | 0.364618359 | 0 | 15461 | 948 |
| chr13_9736882_9720773_-16109-Zmynd11 | chr13 | Zmynd11 | 0 | 0 | 0.180646022 | 16110 | 16110 |
| chr13_9748797_9720773_-28024-Zmynd11 | chr13 | Zmynd11 | 0 | 0 | 0.270969033 | 28025 | 28025 |
| chr13_98272852_98266151_+6701-Ankra2 | chr13 | Ankra2 | 0.405592706 | 0.364618359 | 0.361292045 | 6702 | 992 |
| chr13_98272852_98267316_+5536-Ankra2 | chr13 | Ankra2 | 0.202796353 | 0.182309179 | 0 | 5537 | 597 |
| chr13_99403582_99362965_+40617-Mrps27 | chr13 | Mrps27 | 0 | 0.09115459 | 0 | 40618 | 447 |
| chr13_99415201_99400262_+14939-Mrps27 | chr13 | Mrps27 | 0.202796353 | 0 | 0 | 14940 | 1313 |
| chr14_100109860_100022622_-87238-Klf12 | chr14 | Klf12 | 0 | 0.09115459 | 0 | 87239 | 791 |
| chr14_101465764_101447527_-18237-Tbc1d4 | chr14 | Tbc1d4 | 0 | 0.182309179 | 0 | 18238 | 1481 |
| chr14_101507690_101501012_-6678-Tbc1d4 | chr14 | Tbc1d4 | 0 | 0.364618359 | 0.180646022 | 6679 | 798 |
| chr14_101888706_101886937_+1769-na | chr14 | na | 0.101398176 | 0 | 0 | 1770 | 1770 |
| chr14_101914337_101886937_+27400-Lmo7 | chr14 | Lmo7 | 0.202796353 | 0 | 0 | 27401 | 2571 |
| chr14_101929451_101886937_+42514-Lmo7 | chr14 | Lmo7 | 0 | 0 | 0.180646022 | 42515 | 3904 |
| chr14_101932672_101918612_+14060-Lmo7 | chr14 | Lmo7 | 0.202796353 | 0 | 0 | 14061 | 1357 |
| chr14_103252526_103248362_-4164-Mycbp2 | chr14 | Mycbp2 | 0.202796353 | 0 | 0 | 4165 | 323 |
| chr14_103291362_103182354_-109008-Mycbp2 | chr14 | Mycbp2 | 0 | 0 | 0.180646022 | 109009 | 6302 |
| chr14_106397826_106360437_+37389-Gm41240 | chr14 | Gm41240 | 0.202796353 | 0 | 0 | 37390 | 37390 |
| chr14_118654374_118627452_-26922-Abcc4 | chr14 | Abcc4 | 0 | 0 | 0.180646022 | 26923 | 855 |
| chr14_118669107_118490499_-178608-Abcc4 | chr14 | Abcc4 | 0 | 0.182309179 | 0 | 178609 | 3796 |
| chr14_118669107_118654150_-14957-Abcc4 | chr14 | Abcc4 | 0.202796353 | 0 | 0 | 14958 | 457 |
| chr14_118960860_118953109_+7751-Dnajc3 | chr14 | Dnajc3 | 0 | 0 | 0.361292045 | 7752 | 311 |
| chr14_120325412_120324794_+618-Mbnl2 | chr14 | Mbnl2 | 0 | 0 | 0.361292045 | 619 | 619 |
| chr14_120923585_120917356_+6229-Ipo5 | chr14 | Ipo5 | 0 | 0.364618359 | 0 | 6230 | 579 |
| chr14_12110133_12037341_+72792-Ptprg | chr14 | Ptprg | 0 | 0.182309179 | 0 | 72793 | 321 |
| chr14_121591948_121591311_-637-Dock9 | chr14 | Dock9 | 0 | 0.09115459 | 0 | 638 | 181 |
| chr14_12166877_12153658_+13219-Ptprg | chr14 | Ptprg | 0.202796353 | 0 | 0 | 13220 | 902 |
| chr14_121908320_121905123_+3197-Ubac2 | chr14 | Ubac2 | 0 | 0.546927538 | 0 | 3198 | 358 |
| chr14_122370377_122370119_-258-na | chr14 | na | 0.202796353 | 0 | 0 | 259 | 259 |
| chr14_122616885_120379150_+2237735-Ipo5 | chr14 | Ipo5 | 0.202796353 | 0 | 0 | 2237736 | longer than 2000000bp |
| chr14_122714125_122658678_+55447-Pcca | chr14 | Pcca | 0 | 0 | 0.180646022 | 55448 | 927 |
| chr14_122714125_122684882_+29243-Pcca | chr14 | Pcca | 0 | 0.182309179 | 0 | 29244 | 578 |
| chr14_123827898_123827752_+146-Itgbl1 | chr14 | Itgbl1 | 0.202796353 | 0 | 0 | 147 | 147 |
| chr14_13954656_13951206_-3450-Thoc7 | chr14 | Thoc7 | 0 | 0 | 0.180646022 | 3451 | 391 |
| chr14_14053023_13995007_+58016-Atxn7 | chr14 | Atxn7 | 0 | 0.364618359 | 0 | 58017 | 560 |
| chr14_14090109_14087283_+2826-Atxn7 | chr14 | Atxn7 | 0 | 0.364618359 | 0.180646022 | 2827 | 593 |
| chr14_14103507_14052919_+50588-Atxn7 | chr14 | Atxn7 | 0 | 0.182309179 | 0 | 50589 | 2499 |
| chr14_14104137_14013073_+91064-Atxn7 | chr14 | Atxn7 | 0 | 0 | 0.180646022 | 91065 | 3495 |
| chr14_16283473_16270475_+12998-Ngly1 | chr14 | Ngly1 | 0 | 0.182309179 | 0 | 12999 | 768 |
| chr14_16283473_16277799_+5674-Ngly1 | chr14 | Ngly1 | 0 | 0.182309179 | 0.180646022 | 5675 | 602 |
| chr14_16294673_16277799_+16874-Ngly1 | chr14 | Ngly1 | 0 | 0 | 0.180646022 | 16875 | 953 |
| chr14_16294673_16280693_+13980-Ngly1 | chr14 | Ngly1 | 0 | 0 | 0.180646022 | 13981 | 730 |
| chr14_16294673_16290771_+3902-Ngly1 | chr14 | Ngly1 | 0 | 0 | 0.361292045 | 3903 | 351 |
| chr14_16429432_16408827_+20605-Top2b | chr14 | Top2b | 0 | 0.364618359 | 0 | 20606 | 2483 |
| chr14_18011270_18008569_+2701-Thrb | chr14 | Thrb | 0.405592706 | 0.182309179 | 0 | 2702 | 354 |
| chr14_18222253_18211779_-10474-Nr1d2 | chr14 | Nr1d2 | 0 | 0.182309179 | 0 | 10475 | 1307 |
| chr14_20307799_20300587_+7212-Nudt13 | chr14 | Nudt13 | 0.202796353 | 0 | 0 | 7213 | 607 |
| chr14_20464946_20460159_-4787-Anxa7 | chr14 | Anxa7 | 0 | 0.182309179 | 0.180646022 | 4788 | 730 |
| chr14_20501662_20329954_-171708-Cfap70 | chr14 | Cfap70 | 0 | 0 | 0.180646022 | 171709 | 171709 |
| chr14_20562242_20556247_-5995-Usp54 | chr14 | Usp54 | 0.202796353 | 0 | 0 | 5996 | 1516 |
| chr14_20757728_20742705_-15023-Camk2g | chr14 | Camk2g | 0 | 0.182309179 | 0 | 15024 | 303 |
| chr14_20760480_20653624_-106856-Ndst2 | chr14 | Ndst2 | 0.405592706 | 0 | 0 | 106857 | 106857 |
| chr14_20770129_20760144_-9985-Camk2g | chr14 | Camk2g | 0 | 0.182309179 | 0 | 9986 | 671 |
| chr14_21318183_21234833_+83350-Adk | chr14 | Adk | 0 | 0 | 0.180646022 | 83351 | 453 |
| chr14_21318183_21240463_+77720-Adk | chr14 | Adk | 0 | 0.364618359 | 0 | 77721 | 280 |
| chr14_21517498_21516626_+872-Kat6b | chr14 | Kat6b | 0.608389058 | 0.546927538 | 0 | 873 | 873 |
| chr14_21609862_21516626_+93236-Kat6b | chr14 | Kat6b | 0.202796353 | 0 | 0 | 93237 | 982 |
| chr14_21637640_21618891_+18749-Kat6b | chr14 | Kat6b | 0.811185411 | 0.546927538 | 0.541938067 | 18750 | 1896 |
| chr14_21637640_21626719_+10921-Kat6b | chr14 | Kat6b | 1.825167175 | 13.49087927 | 5.238734648 | 10922 | 636 |
| chr14_21664748_21626719_+38029-Kat6b | chr14 | Kat6b | 0 | 0.182309179 | 0.090323011 | 38030 | 1712 |
| chr14_23391007_23330840_-60167-Kcnma1 | chr14 | Kcnma1 | 0 | 0.182309179 | 0 | 60168 | 1049 |
| chr14_23543106_23435950_-107156-Kcnma1 | chr14 | Kcnma1 | 0 | 0.182309179 | 0 | 107157 | 1296 |
| chr14_25406932_25406150_-782-Zmiz1os1 | chr14 | Zmiz1os1 | 0 | 0.182309179 | 0 | 783 | 221 |
| chr14_25581673_25544786_+36887-Zmiz1 | chr14 | Zmiz1 | 0.202796353 | 0 | 0.361292045 | 36888 | 36888 |
| chr14_25636017_25572020_+63997-Zmiz1 | chr14 | Zmiz1 | 0.202796353 | 0 | 0 | 63998 | 474 |
| chr14_26428316_26414919_-13397-Slmap | chr14 | Slmap | 0 | 0.182309179 | 0 | 13398 | 981 |
| chr14_26428316_26426565_-1751-Slmap | chr14 | Slmap | 0 | 0 | 0.180646022 | 1752 | 514 |
| chr14_26592437_26585339_+7098-Dennd6a | chr14 | Dennd6a | 0 | 0.09115459 | 0 | 7099 | 176 |
| chr14_26606960_26604674_+2286-Dennd6a | chr14 | Dennd6a | 0.202796353 | 0 | 0.541938067 | 2287 | 199 |
| chr14_26901225_26900847_+378-Asb14 | chr14 | Asb14 | 0 | 0 | 0.361292045 | 379 | 291 |
| chr14_26914330_26900847_+13483-Asb14 | chr14 | Asb14 | 0 | 0.364618359 | 0 | 13484 | 1407 |
| chr14_26958511_26949442_-9069-Appl1 | chr14 | Appl1 | 0 | 0.182309179 | 0 | 9070 | 637 |
| chr14_27777242_27776159_+1083-Erc2 | chr14 | Erc2 | 0 | 0 | 0.361292045 | 1084 | 441 |
| chr14_30787607_30765963_+21644-Sfmbt1 | chr14 | Sfmbt1 | 0.202796353 | 0 | 0 | 21645 | 1153 |
| chr14_30790816_30765963_+24853-Sfmbt1 | chr14 | Sfmbt1 | 0.202796353 | 0 | 0 | 24854 | 1236 |
| chr14_30975827_30963933_+11894-Nek4 | chr14 | Nek4 | 0 | 0 | 0.180646022 | 11895 | 905 |
| chr14_31040531_31030844_+9687-Pbrm1 | chr14 | Pbrm1 | 0 | 0 | 0.180646022 | 9688 | 577 |
| chr14_31074982_31061469_+13513-Pbrm1 | chr14 | Pbrm1 | 0 | 0.182309179 | 0 | 13514 | 1238 |
| chr14_31089622_31061469_+28153-Pbrm1 | chr14 | Pbrm1 | 0.608389058 | 0.364618359 | 0.180646022 | 28154 | 2150 |
| chr14_31089622_31061525_+28097-Pbrm1 | chr14 | Pbrm1 | 0 | 0 | 0.722584089 | 28098 | 2094 |
| chr14_31185425_31184942_-483-Nisch | chr14 | Nisch | 0 | 0.729236718 | 0 | 484 | 223 |
| chr14_31191548_31184942_-6606-Nisch | chr14 | Nisch | 0 | 0 | 0.180646022 | 6607 | 1119 |
| chr14_31248134_31241726_-6408-Phf7 | chr14 | Phf7 | 0 | 0 | 0.722584089 | 6409 | 194 |
| chr14_31366243_31355265_+10978-Capn7 | chr14 | Capn7 | 0 | 0.364618359 | 0 | 10979 | 1141 |
| chr14_31369851_31363479_+6372-Capn7 | chr14 | Capn7 | 0 | 0 | 0.180646022 | 6373 | 797 |
| chr14_31492456_31482791_-9665-Mettl6 | chr14 | Mettl6 | 0 | 0 | 0.361292045 | 9666 | 448 |
| chr14_32552710_32526149_+26561-Ercc6 | chr14 | Ercc6 | 0.202796353 | 0.364618359 | 1.625814201 | 26562 | 1154 |
| chr14_32563569_32557839_+5730-Ercc6 | chr14 | Ercc6 | 0 | 0 | 0.180646022 | 5731 | 5731 |
| chr14_32650933_32650671_-262-na | chr14 | na | 0 | 0.182309179 | 0 | 263 | 263 |
| chr14_34429800_34422560_-7240-Bmpr1a | chr14 | Bmpr1a | 0 | 0.364618359 | 0 | 7241 | 636 |
| chr14_34460612_34441099_-19513-Bmpr1a | chr14 | Bmpr1a | 0 | 0.182309179 | 0 | 19514 | 587 |
| chr14_34552322_34541868_-10454-Ldb3 | chr14 | Ldb3 | 0 | 0.182309179 | 0 | 10455 | 893 |
| chr14_34567567_34566857_-710-Ldb3 | chr14 | Ldb3 | 0 | 0 | 0.180646022 | 711 | 207 |
| chr14_34571921_34566857_-5064-Ldb3 | chr14 | Ldb3 | 0 | 0 | 0.361292045 | 5065 | 426 |
| chr14_34695105_34691682_+3423-Wapl | chr14 | Wapl | 0 | 0 | 0.361292045 | 3424 | 1172 |
| chr14_45340691_45330987_+9704-Psmc6 | chr14 | Psmc6 | 0 | 0.182309179 | 0 | 9705 | 426 |
| chr14_45343727_45334653_+9074-Psmc6 | chr14 | Psmc6 | 0 | 0.182309179 | 0 | 9075 | 721 |
| chr14_45629750_45614163_-15587-Ddhd1 | chr14 | Ddhd1 | 0.202796353 | 0 | 0 | 15588 | 764 |
| chr14_46097166_46082710_-14456-na | chr14 | na | 0 | 0.546927538 | 0 | 14457 | 14457 |
| chr14_46727661_46718453_-9208-na | chr14 | na | 0.202796353 | 0 | 0 | 9209 | 9209 |
| chr14_46917051_46908987_+8064-na | chr14 | na | 0 | 0.182309179 | 0.180646022 | 8065 | 8065 |
| chr14_47016795_46896018_+120777-Samd4 | chr14 | Samd4 | 0.405592706 | 0 | 0.180646022 | 120778 | 120778 |
| chr14_47016795_46907915_+108880-Samd4 | chr14 | Samd4 | 0.202796353 | 0 | 0.541938067 | 108881 | 108881 |
| chr14_47026840_47016277_+10563-Samd4 | chr14 | Samd4 | 0 | 0 | 0.180646022 | 10564 | 10564 |
| chr14_47053113_47016277_+36836-Samd4 | chr14 | Samd4 | 0 | 0.182309179 | 0 | 36837 | 933 |
| chr14_47083615_47064220_+19395-Samd4 | chr14 | Samd4 | 0.405592706 | 0 | 0 | 19396 | 715 |
| chr14_47083615_47073836_+9779-Samd4 | chr14 | Samd4 | 0 | 0 | 0.180646022 | 9780 | 518 |
| chr14_47089170_47073836_+15334-Samd4 | chr14 | Samd4 | 0 | 0 | 0.180646022 | 15335 | 847 |
| chr14_47096174_47073836_+22338-Samd4 | chr14 | Samd4 | 0 | 0.182309179 | 0 | 22339 | 931 |
| chr14_47673070_47663756_+9314-Ktn1 | chr14 | Ktn1 | 0 | 0.364618359 | 0 | 9315 | 1003 |
| chr14_47693943_47683383_+10560-Ktn1 | chr14 | Ktn1 | 0 | 0.364618359 | 0 | 10561 | 762 |
| chr14_50935359_50929575_+5784-na | chr14 | na | 0 | 0 | 0.180646022 | 5785 | 5785 |
| chr14_52176513_52170827_-5686-Supt16 | chr14 | Supt16 | 0 | 0 | 0.180646022 | 5687 | 1149 |
| chr14_54667161_54662168_-4993-Acin1 | chr14 | Acin1 | 1.216778117 | 0.364618359 | 0.361292045 | 4994 | 1687 |
| chr14_54679352_54662168_-17184-Acin1 | chr14 | Acin1 | 0 | 0 | 0.361292045 | 17185 | 1919 |
| chr14_54991994_54963896_-28098-Myh7 | chr14 | Myh7 | 0 | 0 | 0.180646022 | 28099 | 28099 |
| chr14_57450741_57444355_+6386-Ift88 | chr14 | Ift88 | 0 | 0.182309179 | 0 | 6387 | 521 |
| chr14_57734418_57733878_-540-Lats2 | chr14 | Lats2 | 0 | 0 | 0.361292045 | 541 | 541 |
| chr14_57878459_57856610_-21849-Zdhhc20 | chr14 | Zdhhc20 | 0 | 0.546927538 | 0 | 21850 | 476 |
| chr14_59419561_59406646_-12915-Setdb2-phf11c | chr14 | Setdb2-phf11c | 0 | 0 | 0.361292045 | 12916 | 1671 |
| chr14_59431268_59406646_-24622-Setdb2-phf11c | chr14 | Setdb2-phf11c | 0 | 0.182309179 | 0 | 24623 | 1960 |
| chr14_59539205_59504451_+34754-Cab39l | chr14 | Cab39l | 0.202796353 | 0 | 0 | 34755 | 34755 |
| chr14_61193479_61189602_+3877-Sacs | chr14 | Sacs | 0 | 0 | 0.722584089 | 3878 | 1734 |
| chr14_61236895_61225195_-11700-Sgcg | chr14 | Sgcg | 0.202796353 | 0.182309179 | 0 | 11701 | 405 |
| chr14_64757584_64746299_+11285-Kif13b | chr14 | Kif13b | 0.202796353 | 0 | 0 | 11286 | 1162 |
| chr14_64757584_64748446_+9138-Kif13b | chr14 | Kif13b | 0.202796353 | 0 | 0 | 9139 | 760 |
| chr14_64779768_64748446_+31322-Kif13b | chr14 | Kif13b | 0 | 0 | 0.180646022 | 31323 | 2134 |
| chr14_64788519_64748446_+40073-Kif13b | chr14 | Kif13b | 0.608389058 | 0.911545897 | 0.722584089 | 40074 | 2300 |
| chr14_64791074_64776237_+14837-Kif13b | chr14 | Kif13b | 0 | 0.364618359 | 0 | 14838 | 14838 |
| chr14_64800407_64748446_+51961-Kif13b | chr14 | Kif13b | 0 | 0 | 0.180646022 | 51962 | 2497 |
| chr14_65080475_65075584_-4891-Extl3 | chr14 | Extl3 | 0.202796353 | 0 | 0 | 4892 | 2702 |
| chr14_67390566_67371693_+18873-Ebf2 | chr14 | Ebf2 | 0 | 0 | 0.361292045 | 18874 | 458 |
| chr14_67729733_67724558_+5175-Kctd9 | chr14 | Kctd9 | 0 | 0 | 0.361292045 | 5176 | 578 |
| chr14_69798324_69793904_-4420-Rhobtb2 | chr14 | Rhobtb2 | 0 | 0 | 0.361292045 | 4421 | 1475 |
| chr14_69851754_69847483_+4271-Pebp4 | chr14 | Pebp4 | 1.216778117 | 0 | 0 | 4272 | 336 |
| chr14_70142531_70141611_-920-Ccar2 | chr14 | Ccar2 | 0.202796353 | 0.364618359 | 0 | 921 | 637 |
| chr14_70152757_70151880_-877-Ccar2 | chr14 | Ccar2 | 0.202796353 | 0 | 0 | 878 | 278 |
| chr14_70267506_70256360_-11146-Ppp3cc | chr14 | Ppp3cc | 0 | 0 | 0.361292045 | 11147 | 435 |
| chr14_70707433_70701693_-5740-Xpo7 | chr14 | Xpo7 | 0.811185411 | 0.182309179 | 0.180646022 | 5741 | 501 |
| chr14_70707433_70701720_-5713-Xpo7 | chr14 | Xpo7 | 0.811185411 | 1.093855076 | 0.541938067 | 5714 | 474 |
| chr14_72683771_72599066_-84705-Fndc3a | chr14 | Fndc3a | 0 | 0.09115459 | 0 | 84706 | 291 |
| chr14_74796091_74775581_-20510-Lrch1 | chr14 | Lrch1 | 0 | 0 | 0.180646022 | 20511 | 639 |
| chr14_75294002_75291569_+2433-Zc3h13 | chr14 | Zc3h13 | 0.202796353 | 0 | 0.180646022 | 2434 | 348 |
| chr14_75316078_75291572_+24506-Zc3h13 | chr14 | Zc3h13 | 0 | 0 | 0.180646022 | 24507 | 950 |
| chr14_75328144_75291569_+36575-Zc3h13 | chr14 | Zc3h13 | 0 | 0.182309179 | 0 | 36576 | 2705 |
| chr14_78589981_78569904_-20077-Dgkh | chr14 | Dgkh | 0.202796353 | 0 | 0 | 20078 | 1334 |
| chr14_78947272_78933666_+13606-Vwa8 | chr14 | Vwa8 | 0 | 0 | 0.180646022 | 13607 | 561 |
| chr14_78995163_78933666_+61497-Vwa8 | chr14 | Vwa8 | 0 | 0 | 0.361292045 | 61498 | 1296 |
| chr14_79009279_78982236_+27043-Vwa8 | chr14 | Vwa8 | 0 | 0.364618359 | 0 | 27044 | 638 |
| chr14_79018713_78933666_+85047-Vwa8 | chr14 | Vwa8 | 0 | 0 | 0.180646022 | 85048 | 1519 |
| chr14_79020686_78933666_+87020-Vwa8 | chr14 | Vwa8 | 0 | 0 | 0.180646022 | 87021 | 1626 |
| chr14_79026077_78933666_+92411-Vwa8 | chr14 | Vwa8 | 0.405592706 | 0 | 0.180646022 | 92412 | 1698 |
| chr14_79026077_79009164_+16913-Vwa8 | chr14 | Vwa8 | 0 | 0.182309179 | 0.180646022 | 16914 | 402 |
| chr14_79065005_79056647_+8358-Vwa8 | chr14 | Vwa8 | 0.405592706 | 0 | 0 | 8359 | 690 |
| chr14_79065005_79058673_+6332-Vwa8 | chr14 | Vwa8 | 0.202796353 | 0 | 0.180646022 | 6333 | 614 |
| chr14_79098429_79064854_+33575-Vwa8 | chr14 | Vwa8 | 0 | 0.182309179 | 0 | 33576 | 1363 |
| chr14_79103696_79056647_+47049-Vwa8 | chr14 | Vwa8 | 0 | 0.364618359 | 0 | 47050 | 2188 |
| chr14_79103696_79086620_+17076-Vwa8 | chr14 | Vwa8 | 0.202796353 | 0 | 0.361292045 | 17077 | 1250 |
| chr14_7915384_7907153_+8231-Flnb | chr14 | Flnb | 0 | 0.364618359 | 0 | 8232 | 790 |
| chr14_79387106_79381495_-5611-Naa16 | chr14 | Naa16 | 0 | 0.182309179 | 0.361292045 | 5612 | 483 |
| chr14_79573618_79567150_+6468-Elf1 | chr14 | Elf1 | 0 | 0 | 0.180646022 | 6469 | 889 |
| chr14_79580789_79580185_+604-na | chr14 | na | 0 | 0 | 0.180646022 | 605 | 605 |
| chr14_8141544_8136869_+4675-Pxk | chr14 | Pxk | 0 | 0.364618359 | 0 | 4676 | 434 |
| chr14_87480883_87472076_+8807-Tdrd3 | chr14 | Tdrd3 | 0 | 0.182309179 | 0 | 8808 | 525 |
| chr14_87506606_87472076_+34530-Tdrd3 | chr14 | Tdrd3 | 0 | 0 | 0.180646022 | 34531 | 1797 |
| chr14_87511770_87472076_+39694-Tdrd3 | chr14 | Tdrd3 | 0 | 0 | 0.180646022 | 39695 | 1923 |
| chr14_97969968_97827742_-142226-Dach1 | chr14 | Dach1 | 0.202796353 | 0 | 0 | 142227 | 1275 |
| chr14_99156619_99133236_+23383-Pibf1 | chr14 | Pibf1 | 0 | 0.364618359 | 0 | 23384 | 708 |
| chr14_99211070_99179315_+31755-Pibf1 | chr14 | Pibf1 | 0 | 0.364618359 | 0 | 31756 | 642 |
| chr14_99211070_99186481_+24589-Pibf1 | chr14 | Pibf1 | 0 | 0 | 0.180646022 | 24590 | 476 |
| chr15_100412262_100395270_-16992-Slc11a2 | chr15 | Slc11a2 | 0 | 0.364618359 | 0 | 16993 | 1667 |
| chr15_100476886_100469685_+7201-Letmd1 | chr15 | Letmd1 | 1.013981764 | 0 | 0.541938067 | 7202 | 890 |
| chr15_100525672_100520563_-5109-Tfcp2 | chr15 | Tfcp2 | 0 | 0.546927538 | 0 | 5110 | 366 |
| chr15_101195057_101193932_+1125-Acvr1b | chr15 | Acvr1b | 0.202796353 | 0 | 0 | 1126 | 489 |
| chr15_101202145_101198784_+3361-Acvr1b | chr15 | Acvr1b | 0.811185411 | 0.546927538 | 0.541938067 | 3362 | 399 |
| chr15_101204577_101193932_+10645-Acvr1b | chr15 | Acvr1b | 0.202796353 | 0.729236718 | 0.361292045 | 10646 | 1170 |
| chr15_101204577_101198784_+5793-Acvr1b | chr15 | Acvr1b | 0.608389058 | 0.182309179 | 0.903230112 | 5794 | 681 |
| chr15_101211402_101193932_+17470-Acvr1b | chr15 | Acvr1b | 0 | 0.182309179 | 0 | 17471 | 1967 |
| chr15_102087219_102084161_+3058-Eif4b | chr15 | Eif4b | 0.202796353 | 0 | 0 | 3059 | 3059 |
| chr15_102547872_102538183_-9689-Atf7 | chr15 | Atf7 | 0.202796353 | 0 | 0 | 9690 | 674 |
| chr15_12140652_12136396_+4256-Zfr | chr15 | Zfr | 0 | 0.182309179 | 0 | 4257 | 647 |
| chr15_12154565_12136396_+18169-Zfr | chr15 | Zfr | 0.608389058 | 0 | 0 | 18170 | 1696 |
| chr15_12171854_12136396_+35458-Zfr | chr15 | Zfr | 0.405592706 | 0 | 0 | 35459 | 2698 |
| chr15_12180940_12136396_+44544-Zfr | chr15 | Zfr | 0 | 0.364618359 | 0 | 44545 | 2908 |
| chr15_12184166_12136396_+47770-Zfr | chr15 | Zfr | 0.405592706 | 0 | 0 | 47771 | 3116 |
| chr15_12445665_12406467_-39198-Pdzd2 | chr15 | Pdzd2 | 0 | 0 | 0.180646022 | 39199 | 928 |
| chr15_12867670_12859373_+8297-Drosha | chr15 | Drosha | 0 | 0.182309179 | 0 | 8298 | 403 |
| chr15_12905701_12889619_+16082-Drosha | chr15 | Drosha | 0.202796353 | 0.182309179 | 0 | 16083 | 571 |
| chr15_12926309_12912511_+13798-na | chr15 | na | 0 | 0 | 0.180646022 | 13799 | 13799 |
| chr15_25744256_25737450_+6806-Myo10 | chr15 | Myo10 | 0 | 0.182309179 | 0 | 6807 | 435 |
| chr15_25786046_25782918_+3128-Myo10 | chr15 | Myo10 | 0 | 0.364618359 | 0 | 3129 | 368 |
| chr15_27571654_27562724_+8930-Ank | chr15 | Ank | 0 | 0 | 0.722584089 | 8931 | 495 |
| chr15_27905331_27897834_-7497-Trio | chr15 | Trio | 0.608389058 | 0.182309179 | 0 | 7498 | 1021 |
| chr15_28007495_27995081_-12414-Trio | chr15 | Trio | 0 | 0.182309179 | 0 | 12415 | 12415 |
| chr15_32271404_32269683_+1721-Sema5a | chr15 | Sema5a | 0 | 0.182309179 | 0 | 1722 | 1722 |
| chr15_33250133_33233664_+16469-Cpq | chr15 | Cpq | 0 | 0 | 0.180646022 | 16470 | 16470 |
| chr15_3333525_3325898_-7627-Ghr | chr15 | Ghr | 0 | 0 | 0.541938067 | 7628 | 439 |
| chr15_3347601_3327974_-19627-Ghr | chr15 | Ghr | 0 | 0.364618359 | 0 | 19628 | 675 |
| chr15_34123766_34114024_+9742-Mtdh | chr15 | Mtdh | 0 | 0 | 0.361292045 | 9743 | 748 |
| chr15_34139897_34117880_+22017-Mtdh | chr15 | Mtdh | 0.202796353 | 0 | 0 | 22018 | 951 |
| chr15_34355685_34355563_+122-Matn2 | chr15 | Matn2 | 0 | 0 | 0.180646022 | 123 | 123 |
| chr15_35438978_35417511_+21467-Vps13b | chr15 | Vps13b | 0 | 0 | 0.180646022 | 21468 | 912 |
| chr15_3551685_3388648_-163037-Ghr | chr15 | Ghr | 0.202796353 | 1.093855076 | 0 | 163038 | 163038 |
| chr15_3551685_3457930_-93755-Ghr | chr15 | Ghr | 0.202796353 | 0 | 0 | 93756 | 93756 |
| chr15_35534360_35417511_+116849-Vps13b | chr15 | Vps13b | 0 | 0.182309179 | 0 | 116850 | 2530 |
| chr15_35576526_35533296_+43230-Vps13b | chr15 | Vps13b | 0 | 0 | 0.180646022 | 43231 | 567 |
| chr15_3559042_3544025_-15017-na | chr15 | na | 0 | 0 | 0.361292045 | 15018 | 15018 |
| chr15_3560054_3544025_-16029-na | chr15 | na | 0.202796353 | 0 | 0.180646022 | 16030 | 16030 |
| chr15_3560054_3551533_-8521-na | chr15 | na | 0 | 0 | 0.180646022 | 8522 | 8522 |
| chr15_35623805_35616040_+7765-Vps13b | chr15 | Vps13b | 0 | 0 | 0.180646022 | 7766 | 7766 |
| chr15_35623805_35617598_+6207-Vps13b | chr15 | Vps13b | 0.202796353 | 0 | 0 | 6208 | 425 |
| chr15_35674952_35668701_+6251-Vps13b | chr15 | Vps13b | 0 | 0.364618359 | 0 | 6252 | 475 |
| chr15_35834380_35665215_+169165-Vps13b | chr15 | Vps13b | 0.405592706 | 0 | 0 | 169166 | 169166 |
| chr15_37989652_37972903_-16749-Ubr5 | chr15 | Ubr5 | 0.405592706 | 1.640782614 | 1.445168179 | 16750 | 1958 |
| chr15_38048149_36596563_-1451586-Ubr5 | chr15 | Ubr5 | 0.202796353 | 0 | 0 | 1451587 | 1451587 |
| chr15_38048149_38037816_-10333-Ubr5 | chr15 | Ubr5 | 0 | 0.182309179 | 0 | 10334 | 1036 |
| chr15_4057775_4047465_+10310-Oxct1 | chr15 | Oxct1 | 0.202796353 | 0 | 0 | 10311 | 393 |
| chr15_4101879_4091168_+10711-Oxct1 | chr15 | Oxct1 | 0.202796353 | 0 | 0 | 10712 | 516 |
| chr15_41826052_41797474_+28578-Oxr1 | chr15 | Oxr1 | 0.608389058 | 0.364618359 | 1.083876134 | 28579 | 1715 |
| chr15_42530710_42512284_-18426-Angpt1 | chr15 | Angpt1 | 0 | 0 | 0.180646022 | 18427 | 361 |
| chr15_43513849_43492170_+21679-Emc2 | chr15 | Emc2 | 0 | 0.182309179 | 0 | 21680 | 767 |
| chr15_44405992_44380060_-25932-Nudcd1 | chr15 | Nudcd1 | 0.202796353 | 0 | 0 | 25933 | 1186 |
| chr15_44405992_44388469_-17523-Nudcd1 | chr15 | Nudcd1 | 0 | 0.182309179 | 0 | 17524 | 1026 |
| chr15_44405992_44396511_-9481-Nudcd1 | chr15 | Nudcd1 | 0 | 0 | 0.180646022 | 9482 | 900 |
| chr15_48874558_48874167_+391-na | chr15 | na | 0 | 0.182309179 | 0 | 392 | 392 |
| chr15_50831958_50822105_-9853-Trps1 | chr15 | Trps1 | 0.202796353 | 0 | 0 | 9854 | 1734 |
| chr15_55218273_55208731_+9542-Deptor | chr15 | Deptor | 0.608389058 | 0 | 0 | 9543 | 497 |
| chr15_58055082_58031984_-23098-Zhx1 | chr15 | Zhx1 | 0.202796353 | 0 | 0 | 23099 | 23099 |
| chr15_58055082_58051606_-3476-Zhx1 | chr15 | Zhx1 | 0 | 0.182309179 | 0 | 3477 | 3477 |
| chr15_58055082_58052224_-2858-Zhx1 | chr15 | Zhx1 | 0.202796353 | 0.364618359 | 0.722584089 | 2859 | 2859 |
| chr15_58064180_58052224_-11956-Zhx1 | chr15 | Zhx1 | 0.202796353 | 0.182309179 | 0 | 11957 | 2985 |
| chr15_58208067_58191285_-16782-Fbxo32 | chr15 | Fbxo32 | 0 | 0 | 0.180646022 | 16783 | 535 |
| chr15_58208067_58205213_-2854-Fbxo32 | chr15 | Fbxo32 | 0 | 0 | 0.180646022 | 2855 | 256 |
| chr15_58342570_58341988_+582-na | chr15 | na | 0 | 0.182309179 | 0.180646022 | 583 | 583 |
| chr15_60955192_60953306_+1886-4930402D18Rik | chr15 | 4930402D18Rik | 0 | 0.09115459 | 0 | 1887 | 258 |
| chr15_60967345_60953306_+14039-4930402D18Rik | chr15 | 4930402D18Rik | 0 | 0.273463769 | 0 | 14040 | 14040 |
| chr15_61043354_60927962_+115392-4930402D18Rik | chr15 | 4930402D18Rik | 0 | 0 | 0.180646022 | 115393 | 115393 |
| chr15_62107465_62102345_+5120-Pvt1 | chr15 | Pvt1 | 0.202796353 | 0 | 0.180646022 | 5121 | 5121 |
| chr15_64152930_64135806_-17124-Asap1 | chr15 | Asap1 | 0 | 0.364618359 | 0 | 17125 | 308 |
| chr15_64166545_64124315_-42230-Asap1 | chr15 | Asap1 | 0 | 0 | 0.180646022 | 42231 | 1265 |
| chr15_65848309_65829727_+18582-Efr3a | chr15 | Efr3a | 0.405592706 | 0.364618359 | 0 | 18583 | 665 |
| chr15_66632901_66594807_+38094-Phf20l1 | chr15 | Phf20l1 | 0.202796353 | 0.182309179 | 0 | 38095 | 5229 |
| chr15_66637032_66604027_+33005-Phf20l1 | chr15 | Phf20l1 | 0.202796353 | 0.364618359 | 0 | 33006 | 5142 |
| chr15_6745580_6735365_+10215-Rictor | chr15 | Rictor | 0 | 0 | 0.180646022 | 10216 | 295 |
| chr15_68224943_68165753_-59190-Zfat | chr15 | Zfat | 0 | 0 | 0.361292045 | 59191 | 2853 |
| chr15_6842917_6836916_-6001-Osmr | chr15 | Osmr | 0 | 0 | 0.361292045 | 6002 | 582 |
| chr15_72946185_72936962_-9223-Trappc9 | chr15 | Trappc9 | 0 | 0.182309179 | 0 | 9224 | 424 |
| chr15_72953119_72925506_-27613-Trappc9 | chr15 | Trappc9 | 0 | 0.182309179 | 0 | 27614 | 809 |
| chr15_73276913_73215979_-60934-Ptk2 | chr15 | Ptk2 | 0 | 0.182309179 | 0 | 60935 | 1553 |
| chr15_73343359_73325749_-17610-Ptk2 | chr15 | Ptk2 | 0 | 0.364618359 | 0 | 17611 | 482 |
| chr15_75670184_75667420_-2764-Top1mt | chr15 | Top1mt | 0.202796353 | 0 | 0 | 2765 | 786 |
| chr15_76348004_76347664_-340-Sharpin | chr15 | Sharpin | 0.202796353 | 0 | 0 | 341 | 263 |
| chr15_76601337_76600938_-399-Cpsf1 | chr15 | Cpsf1 | 0.202796353 | 0 | 0 | 400 | 246 |
| chr15_77102939_77091812_-11127-na | chr15 | na | 0 | 0.09115459 | 0 | 11128 | 11128 |
| chr15_77102939_77094607_-8332-Rbfox2 | chr15 | Rbfox2 | 0 | 0.182309179 | 0 | 8333 | 8333 |
| chr15_77102939_77097940_-4999-Rbfox2 | chr15 | Rbfox2 | 0 | 0.182309179 | 0 | 5000 | 353 |
| chr15_77104780_77091812_-12968-Rbfox2 | chr15 | Rbfox2 | 0.405592706 | 0 | 0 | 12969 | 12969 |
| chr15_77813231_77807868_-5363-Myh9 | chr15 | Myh9 | 0 | 0 | 0.180646022 | 5364 | 509 |
| chr15_78055722_78044130_+11592-LOC108168245 | chr15 | LOC108168245 | 0 | 0.182309179 | 0 | 11593 | 11593 |
| chr15_78191638_78191473_+165-na | chr15 | na | 0.608389058 | 0 | 0 | 166 | 166 |
| chr15_78202653_78201226_-1427-Pvalb | chr15 | Pvalb | 0.202796353 | 0 | 0 | 1428 | 243 |
| chr15_79374184_79363658_-10526-Tmem184b | chr15 | Tmem184b | 0 | 0.182309179 | 0 | 10527 | 10527 |
| chr15_79542586_79541034_-1552-Ddx17 | chr15 | Ddx17 | 0 | 0.182309179 | 0 | 1553 | 234 |
| chr15_79708043_79706992_+1051-Gtpbp1 | chr15 | Gtpbp1 | 0.202796353 | 0 | 0 | 1052 | 530 |
| chr15_79824880_79824599_+281-na | chr15 | na | 0.202796353 | 0 | 0 | 282 | 282 |
| chr15_80890276_80884207_+6069-Tnrc6b | chr15 | Tnrc6b | 0.202796353 | 0 | 0 | 6070 | 6070 |
| chr15_80894454_80878867_+15587-Tnrc6b | chr15 | Tnrc6b | 0.202796353 | 0.182309179 | 0 | 15588 | 3059 |
| chr15_80894454_80884207_+10247-Tnrc6b | chr15 | Tnrc6b | 0.202796353 | 0 | 0 | 10248 | 560 |
| chr15_80918097_80884207_+33890-Tnrc6b | chr15 | Tnrc6b | 0 | 0.364618359 | 0.180646022 | 33891 | 1446 |
| chr15_81438180_81430690_+7490-Xpnpep3 | chr15 | Xpnpep3 | 0.202796353 | 0 | 0.180646022 | 7491 | 380 |
| chr15_81601545_81600908_+637-Ep300 | chr15 | Ep300 | 0 | 0 | 0.180646022 | 638 | 638 |
| chr15_81611523_81610355_+1168-Ep300 | chr15 | Ep300 | 0 | 0 | 0.541938067 | 1169 | 376 |
| chr15_81627534_81624525_+3009-Ep300 | chr15 | Ep300 | 0 | 0.182309179 | 0 | 3010 | 680 |
| chr15_81722026_81716567_-5459-Rangap1 | chr15 | Rangap1 | 0.405592706 | 0 | 0 | 5460 | 503 |
| chr15_81823653_81814944_+8709-Tef | chr15 | Tef | 0 | 0.182309179 | 0 | 8710 | 844 |
| chr15_83924461_83879431_-45030-Efcab6 | chr15 | Efcab6 | 0 | 0.182309179 | 0 | 45031 | 1401 |
| chr15_84176876_84171082_+5794-Pnpla3 | chr15 | Pnpla3 | 0.202796353 | 0 | 0 | 5795 | 570 |
| chr15_84181052_84171082_+9970-Pnpla3 | chr15 | Pnpla3 | 0 | 0.364618359 | 0 | 9971 | 1063 |
| chr15_84186081_84171082_+14999-Pnpla3 | chr15 | Pnpla3 | 0.202796353 | 0.729236718 | 0.361292045 | 15000 | 1168 |
| chr15_84206941_84195744_+11197-Samm50 | chr15 | Samm50 | 0.202796353 | 0 | 0 | 11198 | 986 |
| chr15_84955491_84953130_-2361-5031439G07Rik | chr15 | 5031439G07Rik | 0 | 0 | 0.180646022 | 2362 | 435 |
| chr15_89096819_89094137_+2682-Selo | chr15 | Selo | 0.202796353 | 0 | 0 | 2683 | 599 |
| chr15_89101916_89101153_-763-Tubgcp6 | chr15 | Tubgcp6 | 0.202796353 | 0 | 0.180646022 | 764 | 470 |
| chr15_89109056_89101153_-7903-Tubgcp6 | chr15 | Tubgcp6 | 0 | 0.546927538 | 0 | 7904 | 3316 |
| chr15_89253261_89246258_+7003-Ppp6r2 | chr15 | Ppp6r2 | 0 | 0.182309179 | 0.180646022 | 7004 | 326 |
| chr15_89262568_89253019_+9549-Ppp6r2 | chr15 | Ppp6r2 | 0 | 0.182309179 | 0 | 9550 | 784 |
| chr15_89294071_89293945_-126-Sbf1 | chr15 | Sbf1 | 0 | 0.364618359 | 0 | 127 | 127 |
| chr15_89306293_89305544_-749-Sbf1 | chr15 | Sbf1 | 0.202796353 | 0 | 0 | 750 | 306 |
| chr15_89352527_89351983_-544-Lmf2 | chr15 | Lmf2 | 0 | 0.729236718 | 0 | 545 | 378 |
| chr15_90995633_90990247_-5386-Kif21a | chr15 | Kif21a | 0 | 0.364618359 | 0.180646022 | 5387 | 5387 |
| chr15_9103379_9100106_+3273-Nadk2 | chr15 | Nadk2 | 0 | 0.364618359 | 0 | 3274 | 206 |
| chr15_91780009_91772822_+7187-Lrrk2 | chr15 | Lrrk2 | 0 | 0 | 0.180646022 | 7188 | 631 |
| chr15_91814733_91786966_+27767-Lrrk2 | chr15 | Lrrk2 | 0.709787235 | 1.002700487 | 0 | 27768 | 3775 |
| chr15_91814733_91786966_+27767-na | chr15 | na | 0 | 0 | 0.361292045 | 27768 | 27768 |
| chr15_93441712_93424014_+17698-Pphln1 | chr15 | Pphln1 | 0.405592706 | 0 | 0 | 17699 | 274 |
| chr15_93465245_93452117_+13128-Pphln1 | chr15 | Pphln1 | 0 | 0 | 0.180646022 | 13129 | 398 |
| chr15_94333725_94324660_-9065-Adamts20 | chr15 | Adamts20 | 0 | 0.182309179 | 0 | 9066 | 1514 |
| chr15_95920378_95864225_+56153-Ano6 | chr15 | Ano6 | 0 | 1.823091794 | 1.806460223 | 56154 | 928 |
| chr15_95931843_95864225_+67618-Ano6 | chr15 | Ano6 | 0 | 0 | 0.180646022 | 67619 | 1095 |
| chr15_95931843_95912305_+19538-Ano6 | chr15 | Ano6 | 0.202796353 | 0 | 0 | 19539 | 883 |
| chr15_95931843_95913372_+18471-Ano6 | chr15 | Ano6 | 0.202796353 | 0 | 0 | 18472 | 817 |
| chr15_95949956_95948272_+1684-Ano6 | chr15 | Ano6 | 0.405592706 | 0 | 0 | 1685 | 494 |
| chr15_96370194_96356693_+13501-Arid2 | chr15 | Arid2 | 0 | 0 | 0.180646022 | 13502 | 1769 |
| chr15_97019861_97005820_-14041-Slc38a4 | chr15 | Slc38a4 | 0 | 0.182309179 | 0 | 14042 | 1318 |
| chr15_97811628_97810853_-775-Hdac7 | chr15 | Hdac7 | 0 | 0 | 0.180646022 | 776 | 394 |
| chr15_98122915_98121197_+1718-na | chr15 | na | 0 | 0 | 0.090323011 | 1719 | 1719 |
| chr15_98563873_98528018_+35855-na | chr15 | na | 0 | 0.182309179 | 0 | 35856 | 35856 |
| chr15_98604735_98592127_-12608-Adcy6 | chr15 | Adcy6 | 0 | 0 | 0.361292045 | 12609 | 3967 |
| chr16_10513287_10506196_+7091-Ciita | chr16 | Ciita | 0.202796353 | 0 | 0 | 7092 | 2221 |
| chr16_10638915_10611003_+27912-Clec16a | chr16 | Clec16a | 0 | 0.364618359 | 0 | 27913 | 692 |
| chr16_10694790_10611003_+83787-Clec16a | chr16 | Clec16a | 0 | 0.364618359 | 0 | 83788 | 1341 |
| chr16_10742931_10741614_+1317-na | chr16 | na | 0.202796353 | 0 | 0.180646022 | 1318 | 1318 |
| chr16_11128703_11093816_-34887-Txndc11 | chr16 | Txndc11 | 0.202796353 | 0 | 0 | 34888 | 652 |
| chr16_11420783_11370803_+49980-Snx29 | chr16 | Snx29 | 0 | 0.455772948 | 0 | 49981 | 49981 |
| chr16_11447508_11445165_+2343-Snx29 | chr16 | Snx29 | 0.101398176 | 0.09115459 | 0 | 2344 | 193 |
| chr16_11660627_11639374_+21253-Snx29 | chr16 | Snx29 | 0 | 1.093855076 | 0 | 21254 | 21254 |
| chr16_13628449_13566570_-61879-Parn | chr16 | Parn | 0.202796353 | 0 | 0 | 61880 | 644 |
| chr16_13647299_13603028_-44271-Parn | chr16 | Parn | 0 | 0.182309179 | 0 | 44272 | 697 |
| chr16_13800016_13790621_+9395-Rrn3 | chr16 | Rrn3 | 0.202796353 | 0 | 0 | 9396 | 596 |
| chr16_13811609_13810504_+1105-Rrn3 | chr16 | Rrn3 | 0 | 0 | 0.722584089 | 1106 | 241 |
| chr16_13872523_13854395_-18128-Pdxdc1 | chr16 | Pdxdc1 | 0 | 0 | 0.541938067 | 18129 | 561 |
| chr16_14140372_14127056_-13316-Marf1 | chr16 | Marf1 | 0.405592706 | 0 | 0 | 13317 | 1684 |
| chr16_14413382_14403990_+9392-Abcc1 | chr16 | Abcc1 | 0 | 0 | 0.180646022 | 9393 | 768 |
| chr16_14413382_14410700_+2682-Abcc1 | chr16 | Abcc1 | 0.202796353 | 0 | 0 | 2683 | 574 |
| chr16_14423260_14389807_+33453-Abcc1 | chr16 | Abcc1 | 0 | 0.182309179 | 0 | 33454 | 1632 |
| chr16_14423260_14403990_+19270-Abcc1 | chr16 | Abcc1 | 0 | 0 | 0.361292045 | 19271 | 1065 |
| chr16_14423260_14410700_+12560-Abcc1 | chr16 | Abcc1 | 0.405592706 | 0 | 0 | 12561 | 871 |
| chr16_14445469_14436507_+8962-Abcc1 | chr16 | Abcc1 | 0 | 0.182309179 | 0.361292045 | 8963 | 612 |
| chr16_14461208_14436507_+24701-Abcc1 | chr16 | Abcc1 | 0.202796353 | 0 | 0 | 24702 | 1701 |
| chr16_14461208_14445293_+15915-Abcc1 | chr16 | Abcc1 | 0.202796353 | 0 | 0 | 15916 | 1266 |
| chr16_15706978_15697123_+9855-Prkdc | chr16 | Prkdc | 0 | 0 | 0.180646022 | 9856 | 919 |
| chr16_15719512_15648720_+70792-Prkdc | chr16 | Prkdc | 0 | 0 | 0.180646022 | 70793 | 5072 |
| chr16_15816894_15799867_+17027-Prkdc | chr16 | Prkdc | 0.202796353 | 0 | 0.180646022 | 17028 | 1342 |
| chr16_15904261_15902909_-1352-Spidr | chr16 | Spidr | 0 | 0 | 0.180646022 | 1353 | 352 |
| chr16_15915387_15912519_-2868-Spidr | chr16 | Spidr | 0 | 0.182309179 | 0 | 2869 | 289 |
| chr16_16475161_16469704_-5457-Fgd4 | chr16 | Fgd4 | 0 | 0.182309179 | 0 | 5458 | 442 |
| chr16_17026489_17023452_+3037-Mapk1 | chr16 | Mapk1 | 0 | 0.182309179 | 0 | 3038 | 364 |
| chr16_17356216_17348715_-7501-Pi4ka | chr16 | Pi4ka | 0.405592706 | 0 | 0 | 7502 | 737 |
| chr16_20107643_20101992_+5651-Klhl24 | chr16 | Klhl24 | 0 | 0 | 0.361292045 | 5652 | 1044 |
| chr16_20123152_20101992_+21160-Klhl24 | chr16 | Klhl24 | 0 | 0 | 0.361292045 | 21161 | 1973 |
| chr16_20357558_20338876_-18682-Abcc5 | chr16 | Abcc5 | 0 | 0 | 0.361292045 | 18683 | 429 |
| chr16_20400039_20362182_-37857-Abcc5 | chr16 | Abcc5 | 0 | 0 | 0.180646022 | 37858 | 3105 |
| chr16_23970073_23968307_-1766-Bcl6 | chr16 | Bcl6 | 0.405592706 | 0 | 0 | 1767 | 299 |
| chr16_23978589_23974813_-3776-Bcl6 | chr16 | Bcl6 | 0.405592706 | 0.182309179 | 0 | 3777 | 433 |
| chr16_24456942_24442416_+14526-Lpp | chr16 | Lpp | 0.202796353 | 0.182309179 | 0.180646022 | 14527 | 14527 |
| chr16_24559657_24555505_+4152-Lpp | chr16 | Lpp | 0.405592706 | 0 | 0 | 4153 | 4153 |
| chr16_24889959_24761593_+128366-Lpp | chr16 | Lpp | 0 | 0.182309179 | 0 | 128367 | 981 |
| chr16_24889959_24845091_+44868-Lpp | chr16 | Lpp | 0.202796353 | 0 | 0 | 44869 | 297 |
| chr16_25764296_25763266_+1030-Trp63 | chr16 | Trp63 | 0 | 0 | 0.361292045 | 1031 | 262 |
| chr16_29293538_29266944_-26594-Atp13a5 | chr16 | Atp13a5 | 0 | 0.182309179 | 0 | 26595 | 754 |
| chr16_29298254_29232320_-65934-Atp13a5 | chr16 | Atp13a5 | 0 | 0.364618359 | 0 | 65935 | 2260 |
| chr16_29298254_29293421_-4833-Atp13a5 | chr16 | Atp13a5 | 0.304194529 | 0 | 0 | 4834 | 362 |
| chr16_31127433_31116002_-11431-Acap2 | chr16 | Acap2 | 0 | 0 | 0.361292045 | 11432 | 393 |
| chr16_31805734_31796866_+8868-Dlg1 | chr16 | Dlg1 | 0 | 0 | 0.361292045 | 8869 | 266 |
| chr16_31812824_31805614_+7210-Dlg1 | chr16 | Dlg1 | 0.202796353 | 0 | 0 | 7211 | 278 |
| chr16_31990465_31977680_-12785-Senp5 | chr16 | Senp5 | 0.202796353 | 0 | 0.541938067 | 12786 | 1897 |
| chr16_32033899_32027712_-6187-Pak2 | chr16 | Pak2 | 0.608389058 | 0 | 0.180646022 | 6188 | 331 |
| chr16_32044000_32033787_-10213-Pak2 | chr16 | Pak2 | 0 | 0 | 0.541938067 | 10214 | 647 |
| chr16_32844706_32833644_-11062-Rubcn | chr16 | Rubcn | 0 | 0.182309179 | 0 | 11063 | 1243 |
| chr16_32961744_32952057_+9687-Lrch3 | chr16 | Lrch3 | 0.202796353 | 0.364618359 | 0.180646022 | 9688 | 480 |
| chr16_33206002_33204672_+1330-Osbpl11 | chr16 | Osbpl11 | 0 | 0.182309179 | 0.180646022 | 1331 | 256 |
| chr16_33216859_33209926_+6933-Osbpl11 | chr16 | Osbpl11 | 0 | 0.182309179 | 0 | 6934 | 525 |
| chr16_33216859_33214342_+2517-Osbpl11 | chr16 | Osbpl11 | 0 | 0.364618359 | 0 | 2518 | 348 |
| chr16_33236408_33234423_+1985-Osbpl11 | chr16 | Osbpl11 | 0 | 0 | 0.722584089 | 1986 | 337 |
| chr16_33468183_33466855_+1328-Zfp148 | chr16 | Zfp148 | 0.405592706 | 0.546927538 | 0.361292045 | 1329 | 208 |
| chr16_33865652_33706916_+158736-Heg1 | chr16 | Heg1 | 0 | 0.182309179 | 0 | 158737 | 158737 |
| chr16_35735651_35719312_-16339-Dirc2 | chr16 | Dirc2 | 0.202796353 | 0 | 0 | 16340 | 701 |
| chr16_36919796_36904553_+15243-Golgb1 | chr16 | Golgb1 | 0.405592706 | 0 | 0 | 15244 | 7206 |
| chr16_36978778_36968827_-9951-Fbxo40 | chr16 | Fbxo40 | 0.202796353 | 0 | 0 | 9952 | 1947 |
| chr16_37567391_37559995_+7396-Rabl3 | chr16 | Rabl3 | 0.202796353 | 0 | 0 | 7397 | 338 |
| chr16_37878564_37877049_+1515-Lrrc58 | chr16 | Lrrc58 | 0 | 0.182309179 | 0 | 1516 | 407 |
| chr16_3919564_3911451_+8113-Cluap1 | chr16 | Cluap1 | 0 | 0 | 0.180646022 | 8114 | 557 |
| chr16_3942121_3937559_+4562-na | chr16 | na | 0.304194529 | 0 | 0 | 4563 | 4563 |
| chr16_4125762_4107428_-18334-Crebbp | chr16 | Crebbp | 0 | 0 | 0.180646022 | 18335 | 1881 |
| chr16_4155033_4137366_-17667-Crebbp | chr16 | Crebbp | 0 | 0.546927538 | 0.361292045 | 17668 | 532 |
| chr16_4180133_4154857_-25276-Crebbp | chr16 | Crebbp | 0 | 0.182309179 | 0 | 25277 | 887 |
| chr16_4312789_4304377_-8412-Adcy9 | chr16 | Adcy9 | 0.202796353 | 0 | 0 | 8413 | 692 |
| chr16_43462986_43024938_+438048-na | chr16 | na | 0 | 0.09115459 | 0 | 438049 | 438049 |
| chr16_43610931_43609327_+1604-Zbtb20 | chr16 | Zbtb20 | 0.405592706 | 0 | 0 | 1605 | 1605 |
| chr16_44205080_44200614_+4466-Usf3 | chr16 | Usf3 | 0.506990882 | 0.273463769 | 0.361292045 | 4467 | 177 |
| chr16_44521335_44520225_-1110-Boc | chr16 | Boc | 0 | 0.182309179 | 0 | 1111 | 456 |
| chr16_4493880_4482538_-11342-Srl | chr16 | Srl | 0.202796353 | 0 | 0 | 11343 | 1296 |
| chr16_4911468_4907365_+4103-Mgrn1 | chr16 | Mgrn1 | 0.202796353 | 0 | 0 | 4104 | 358 |
| chr16_4953954_4942211_-11743-Anks3 | chr16 | Anks3 | 0.202796353 | 0 | 0 | 11744 | 3672 |
| chr16_50280539_50274547_-5992-Bbx | chr16 | Bbx | 0 | 0.364618359 | 0 | 5993 | 414 |
| chr16_5173166_5171861_+1305-Sec14l5 | chr16 | Sec14l5 | 0.202796353 | 0.364618359 | 0 | 1306 | 303 |
| chr16_5180897_5180216_+681-Sec14l5 | chr16 | Sec14l5 | 0.202796353 | 0 | 0.361292045 | 682 | 526 |
| chr16_5180897_5180394_+503-Sec14l5 | chr16 | Sec14l5 | 0.202796353 | 0 | 0 | 504 | 371 |
| chr16_52166351_52164296_+2055-Cblb | chr16 | Cblb | 0.202796353 | 0 | 0 | 2056 | 549 |
| chr16_52194278_52152511_+41767-Cblb | chr16 | Cblb | 0 | 0.364618359 | 0 | 41768 | 1486 |
| chr16_52194278_52174354_+19924-Cblb | chr16 | Cblb | 0.202796353 | 0 | 0.180646022 | 19925 | 733 |
| chr16_52194278_52183212_+11066-Cblb | chr16 | Cblb | 0.202796353 | 0 | 0 | 11067 | 638 |
| chr16_55819706_55812635_-7071-Nfkbiz | chr16 | Nfkbiz | 0 | 0.182309179 | 0 | 7072 | 1996 |
| chr16_55982406_55980595_+1811-Zbtb11 | chr16 | Zbtb11 | 0 | 0.182309179 | 0 | 1812 | 468 |
| chr16_56002826_55990259_+12567-Zbtb11 | chr16 | Zbtb11 | 0 | 0.364618359 | 0 | 12568 | 1522 |
| chr16_56166012_56138985_+27027-Senp7 | chr16 | Senp7 | 0 | 0.182309179 | 0 | 27028 | 1148 |
| chr16_56596836_56586929_+9907-Abi3bp | chr16 | Abi3bp | 0.811185411 | 0 | 0.722584089 | 9908 | 583 |
| chr16_57513444_57506599_+6845-Filip1l | chr16 | Filip1l | 0.202796353 | 0 | 0.361292045 | 6846 | 609 |
| chr16_57572425_57569650_+2775-na | chr16 | na | 2.433556234 | 2.187710153 | 3.612920447 | 2776 | 2776 |
| chr16_58493861_58484763_-9098-St3gal6 | chr16 | St3gal6 | 0 | 0.364618359 | 0 | 9099 | 428 |
| chr16_58507614_58472311_-35303-St3gal6 | chr16 | St3gal6 | 0 | 0 | 0.361292045 | 35304 | 1169 |
| chr16_58507614_58484763_-22851-St3gal6 | chr16 | St3gal6 | 0.608389058 | 2.187710153 | 3.251628402 | 22852 | 595 |
| chr16_58507614_58507236_-378-na | chr16 | na | 0 | 0.09115459 | 0 | 379 | 379 |
| chr16_59489433_59480291_+9142-Mina | chr16 | Mina | 0.405592706 | 0 | 0 | 9143 | 597 |
| chr16_59524970_59503125_-21845-Crybg3 | chr16 | Crybg3 | 0.202796353 | 0 | 0 | 21846 | 504 |
| chr16_59544165_59522084_-22081-Crybg3 | chr16 | Crybg3 | 0.202796353 | 0 | 0 | 22082 | 1095 |
| chr16_59560242_59552172_-8070-Crybg3 | chr16 | Crybg3 | 0 | 0 | 0.180646022 | 8071 | 6297 |
| chr16_59565643_59550402_-15241-Crybg3 | chr16 | Crybg3 | 0.202796353 | 0 | 0 | 15242 | 6876 |
| chr16_65839733_65828059_+11674-Vgll3 | chr16 | Vgll3 | 0.405592706 | 0 | 0 | 11675 | 643 |
| chr16_65839733_65839209_+524-Vgll3 | chr16 | Vgll3 | 0 | 0.182309179 | 0 | 525 | 525 |
| chr16_70441319_70424167_+17152-Gbe1 | chr16 | Gbe1 | 0 | 0.729236718 | 0 | 17153 | 17153 |
| chr16_70441319_70433545_+7774-Gbe1 | chr16 | Gbe1 | 0 | 0.364618359 | 0.180646022 | 7775 | 563 |
| chr16_70495399_70478306_+17093-Gbe1 | chr16 | Gbe1 | 0 | 0.182309179 | 0.180646022 | 17094 | 626 |
| chr16_7224550_7224311_+239-Rbfox1 | chr16 | Rbfox1 | 0 | 0.546927538 | 0 | 240 | 240 |
| chr16_7277168_7224311_+52857-Rbfox1 | chr16 | Rbfox1 | 0 | 0.182309179 | 0 | 52858 | 384 |
| chr16_7306461_7285478_+20983-Rbfox1 | chr16 | Rbfox1 | 0 | 0.182309179 | 0 | 20984 | 262 |
| chr16_7375969_7224311_+151658-Rbfox1 | chr16 | Rbfox1 | 0.405592706 | 0 | 0 | 151659 | 1008 |
| chr16_7375969_7352932_+23037-Rbfox1 | chr16 | Rbfox1 | 0 | 0.364618359 | 0 | 23038 | 281 |
| chr16_7392040_7352932_+39108-Rbfox1 | chr16 | Rbfox1 | 0.405592706 | 0.729236718 | 0.180646022 | 39109 | 334 |
| chr16_7409768_7224311_+185457-Rbfox1 | chr16 | Rbfox1 | 0.304194529 | 0.364618359 | 0.180646022 | 185458 | 2390 |
| chr16_77596959_77594622_+2337-na | chr16 | na | 0 | 0 | 0.180646022 | 2338 | 2338 |
| chr16_78941804_78941225_+579-Chodl | chr16 | Chodl | 0 | 0.182309179 | 0 | 580 | 468 |
| chr16_85043743_85013627_-30116-App | chr16 | App | 0.405592706 | 0 | 0.180646022 | 30117 | 822 |
| chr16_85465691_85103206_-362485-App | chr16 | App | 0.405592706 | 0 | 0 | 362486 | 362486 |
| chr16_87483016_87475417_+7599-Usp16 | chr16 | Usp16 | 0 | 0 | 0.180646022 | 7600 | 1234 |
| chr16_87722608_87715331_+7277-Bach1 | chr16 | Bach1 | 0.202796353 | 0 | 0.722584089 | 7278 | 1845 |
| chr16_87758413_87755016_+3397-Gm32925 | chr16 | Gm32925 | 0.405592706 | 0.364618359 | 0 | 3398 | 488 |
| chr16_87767062_87755016_+12046-na | chr16 | na | 0 | 0.09115459 | 0 | 12047 | 12047 |
| chr16_8889631_8879745_+9886-Gm30310 | chr16 | Gm30310 | 0 | 0 | 0.180646022 | 9887 | 9887 |
| chr16_90252450_90249627_-2823-Scaf4 | chr16 | Scaf4 | 0 | 0 | 0.180646022 | 2824 | 773 |
| chr16_90475982_90447310_+28672-Hunk | chr16 | Hunk | 0 | 0 | 0.180646022 | 28673 | 619 |
| chr16_90969374_90946792_-22582-Synj1 | chr16 | Synj1 | 0 | 0 | 0.090323011 | 22583 | 2045 |
| chr16_91793767_91782161_+11606-Itsn1 | chr16 | Itsn1 | 0 | 0.364618359 | 0 | 11607 | 378 |
| chr16_91812153_91781778_+30375-Itsn1 | chr16 | Itsn1 | 0.405592706 | 0 | 0 | 30376 | 30376 |
| chr16_91812153_91801527_+10626-Itsn1 | chr16 | Itsn1 | 0 | 0 | 0.180646022 | 10627 | 442 |
| chr16_91820819_91801527_+19292-Itsn1 | chr16 | Itsn1 | 0 | 0.182309179 | 0 | 19293 | 1247 |
| chr16_91820819_91815402_+5417-Itsn1 | chr16 | Itsn1 | 0 | 0.182309179 | 0 | 5418 | 805 |
| chr16_91926982_91926459_-523-Atp5o | chr16 | Atp5o | 0 | 0.546927538 | 0 | 524 | 243 |
| chr16_93764044_93762033_+2011-Dopey2 | chr16 | Dopey2 | 0.202796353 | 0.182309179 | 0.180646022 | 2012 | 1104 |
| chr16_93766316_93762033_+4283-Dopey2 | chr16 | Dopey2 | 0.405592706 | 0 | 0 | 4284 | 1396 |
| chr16_93774109_93769394_+4715-Dopey2 | chr16 | Dopey2 | 0 | 0.182309179 | 0.180646022 | 4716 | 1934 |
| chr16_93780812_93769394_+11418-Dopey2 | chr16 | Dopey2 | 0 | 0 | 0.180646022 | 11419 | 2323 |
| chr16_93781724_93769394_+12330-na | chr16 | na | 0.101398176 | 0 | 0 | 12331 | 12331 |
| chr16_93793231_93769394_+23837-Dopey2 | chr16 | Dopey2 | 0.405592706 | 0 | 0 | 23838 | 2752 |
| chr16_93806471_93801561_+4910-na | chr16 | na | 0 | 0.09115459 | 0 | 4911 | 4911 |
| chr16_93809421_93803462_+5959-Dopey2 | chr16 | Dopey2 | 0.202796353 | 0 | 0.180646022 | 5960 | 1834 |
| chr16_93853898_93849130_+4768-Morc3 | chr16 | Morc3 | 0.608389058 | 0.364618359 | 0 | 4769 | 397 |
| chr16_97870280_97868592_-1688-C2cd2 | chr16 | C2cd2 | 0.608389058 | 0.546927538 | 0 | 1689 | 453 |
| chr16_97872383_97868592_-3791-na | chr16 | na | 0 | 0.182309179 | 0 | 3792 | 3792 |
| chr16_97912704_97909762_-2942-C2cd2 | chr16 | C2cd2 | 0 | 0 | 0.180646022 | 2943 | 2943 |
| chr17_10274063_10273947_-116-Qk | chr17 | Qk | 0 | 0.364618359 | 0 | 117 | 117 |
| chr17_10738983_10736076_-2907-na | chr17 | na | 0 | 0.364618359 | 0 | 2908 | 2908 |
| chr17_11303711_11067129_+236582-Park2 | chr17 | Park2 | 0.405592706 | 0 | 0 | 236583 | 527 |
| chr17_11434633_11067129_+367504-Park2 | chr17 | Park2 | 0 | 0 | 0.180646022 | 367505 | 611 |
| chr17_12272220_12270863_-1357-Map3k4 | chr17 | Map3k4 | 0 | 0 | 0.180646022 | 1358 | 1358 |
| chr17_12714140_12705688_-8452-Igf2r | chr17 | Igf2r | 0 | 0.182309179 | 0 | 8453 | 748 |
| chr17_12714140_12712019_-2121-Igf2r | chr17 | Igf2r | 0 | 0.182309179 | 0 | 2122 | 282 |
| chr17_12719950_12713961_-5989-Igf2r | chr17 | Igf2r | 0.405592706 | 0 | 0 | 5990 | 1208 |
| chr17_13829023_13818121_+10902-Mllt4 | chr17 | Mllt4 | 0 | 0 | 0.361292045 | 10903 | 578 |
| chr17_13859976_13832368_+27608-Mllt4 | chr17 | Mllt4 | 0 | 0 | 0.541938067 | 27609 | 1432 |
| chr17_14375519_14344327_+31192-na | chr17 | na | 0.101398176 | 0 | 0 | 31193 | 31193 |
| chr17_15131489_15120593_-10896-na | chr17 | na | 0 | 0.364618359 | 0 | 10897 | 10897 |
| chr17_15234002_15154790_-79212-Gm5091 | chr17 | Gm5091 | 0 | 0.546927538 | 0 | 79213 | 79213 |
| chr17_15269931_15232159_-37772-Gm5091 | chr17 | Gm5091 | 0 | 0 | 0.180646022 | 37773 | 37773 |
| chr17_15743230_15742157_+1073-Chd1 | chr17 | Chd1 | 0 | 0.364618359 | 0 | 1074 | 316 |
| chr17_17390064_17377719_+12345-Riok2 | chr17 | Riok2 | 0 | 0 | 0.180646022 | 12346 | 1413 |
| chr17_17579372_17529701_-49671-Lnpep | chr17 | Lnpep | 0.811185411 | 0 | 0 | 49672 | 3107 |
| chr17_24100908_24088004_-12904-Pdpk1 | chr17 | Pdpk1 | 0 | 0 | 0.180646022 | 12905 | 732 |
| chr17_24100908_24091421_-9487-Pdpk1 | chr17 | Pdpk1 | 0.202796353 | 0 | 0 | 9488 | 514 |
| chr17_24436324_24433133_+3191-Eci1 | chr17 | Eci1 | 0 | 0 | 0.180646022 | 3192 | 275 |
| chr17_24437610_24436178_+1432-Eci1 | chr17 | Eci1 | 0 | 0.182309179 | 0 | 1433 | 448 |
| chr17_24445551_24445446_-105-E4f1 | chr17 | E4f1 | 0 | 0.182309179 | 0 | 106 | 106 |
| chr17_25213106_25199767_+13339-Unkl | chr17 | Unkl | 0 | 0 | 0.903230112 | 13340 | 793 |
| chr17_25218755_25199767_+18988-Unkl | chr17 | Unkl | 1.216778117 | 0 | 0.180646022 | 18989 | 971 |
| chr17_25381573_25380225_-1348-Cacna1h | chr17 | Cacna1h | 0.811185411 | 0 | 0 | 1349 | 467 |
| chr17_25830061_25829785_+276-Jmjd8 | chr17 | Jmjd8 | 0 | 0 | 0.180646022 | 277 | 189 |
| chr17_26024466_26013774_-10692-Rab11fip3 | chr17 | Rab11fip3 | 0.202796353 | 0 | 0 | 10693 | 448 |
| chr17_26027779_26026130_-1649-Rab11fip3 | chr17 | Rab11fip3 | 0.202796353 | 0 | 0 | 1650 | 1650 |
| chr17_26641704_26634417_+7287-Ergic1 | chr17 | Ergic1 | 0 | 0.182309179 | 0 | 7288 | 390 |
| chr17_26743131_26736789_+6342-Crebrf | chr17 | Crebrf | 2.636352587 | 1.458473435 | 0.541938067 | 6343 | 1411 |
| chr17_26743131_26736816_+6315-Crebrf | chr17 | Crebrf | 0.608389058 | 0 | 0 | 6316 | 1384 |
| chr17_26743131_26739543_+3588-Crebrf | chr17 | Crebrf | 6.286686938 | 4.557729485 | 8.129071005 | 3589 | 1216 |
| chr17_29850688_29849157_-1531-Mdga1 | chr17 | Mdga1 | 0.202796353 | 0 | 0 | 1532 | 600 |
| chr17_30530365_30524713_-5652-Btbd9 | chr17 | Btbd9 | 0 | 0.182309179 | 0 | 5653 | 841 |
| chr17_30885534_30513556_-371978-Glo1 | chr17 | Glo1 | 0 | 0 | 0.903230112 | 371979 | 371979 |
| chr17_30923856_30511699_-412157-Glo1 | chr17 | Glo1 | 0 | 0.364618359 | 0 | 412158 | 412158 |
| chr17_30923856_30513556_-410300-Glo1 | chr17 | Glo1 | 0 | 2.370019332 | 0.903230112 | 410301 | 410301 |
| chr17_31602862_31592105_+10757-Pknox1 | chr17 | Pknox1 | 0 | 0.182309179 | 0 | 10758 | 575 |
| chr17_3164277_3145075_+19202-Scaf8 | chr17 | Scaf8 | 0 | 0 | 0.180646022 | 19203 | 576 |
| chr17_3198223_3145075_+53148-Scaf8 | chr17 | Scaf8 | 0 | 0.182309179 | 0 | 53149 | 3790 |
| chr17_32225713_32220161_-5552-Brd4 | chr17 | Brd4 | 0 | 0.182309179 | 0 | 5553 | 1187 |
| chr17_32253200_32220161_-33039-Brd4 | chr17 | Brd4 | 0 | 0.182309179 | 0 | 33040 | 1666 |
| chr17_32272373_32253041_-19332-Brd4 | chr17 | Brd4 | 0 | 0.182309179 | 0 | 19333 | 19333 |
| chr17_32338823_32332479_-6344-Akap8l | chr17 | Akap8l | 0 | 0 | 0.180646022 | 6345 | 1448 |
| chr17_32777527_32773505_-4022-Zfp871 | chr17 | Zfp871 | 0 | 0 | 0.541938067 | 4023 | 2692 |
| chr17_32777527_32774441_-3086-Zfp871 | chr17 | Zfp871 | 0.405592706 | 0.364618359 | 0.722584089 | 3087 | 1756 |
| chr17_40955217_40938836_+16381-na | chr17 | na | 0 | 0.09115459 | 0 | 16382 | 16382 |
| chr17_42816592_42811040_-5552-Cd2ap | chr17 | Cd2ap | 0 | 0 | 0.361292045 | 5553 | 386 |
| chr17_42834285_42820595_-13690-Cd2ap | chr17 | Cd2ap | 0 | 0.364618359 | 0 | 13691 | 688 |
| chr17_45048392_45033238_+15154-Supt3 | chr17 | Supt3 | 0 | 0.182309179 | 0 | 15155 | 579 |
| chr17_46238987_46238375_+612-Xpo5 | chr17 | Xpo5 | 0 | 0.546927538 | 0 | 613 | 143 |
| chr17_46543783_46543180_-603-Cul9 | chr17 | Cul9 | 0 | 0 | 0.361292045 | 604 | 604 |
| chr17_46858698_46824450_-34248-Gltscr1l | chr17 | Gltscr1l | 0 | 0 | 0.180646022 | 34249 | 2101 |
| chr17_46974045_46964757_-9288-Ubr2 | chr17 | Ubr2 | 0.202796353 | 0 | 0 | 9289 | 917 |
| chr17_46983341_46961499_-21842-Ubr2 | chr17 | Ubr2 | 0.202796353 | 0 | 0 | 21843 | 1905 |
| chr17_50086676_50036941_-49735-Rftn1 | chr17 | Rftn1 | 0 | 0.182309179 | 0 | 49736 | 820 |
| chr17_50546483_50541177_+5306-na | chr17 | na | 0.202796353 | 0 | 0.180646022 | 5307 | 5307 |
| chr17_50800195_50782261_-17934-Tbc1d5 | chr17 | Tbc1d5 | 0 | 0 | 0.180646022 | 17935 | 559 |
| chr17_50920603_50917527_-3076-Tbc1d5 | chr17 | Tbc1d5 | 0.202796353 | 0.364618359 | 0.180646022 | 3077 | 383 |
| chr17_51809287_51803431_-5856-Satb1 | chr17 | Satb1 | 0.405592706 | 0 | 0 | 5857 | 673 |
| chr17_5279382_5242828_+36554-Arid1b | chr17 | Arid1b | 0.202796353 | 0 | 0 | 36555 | 514 |
| chr17_5291283_5279203_+12080-Arid1b | chr17 | Arid1b | 0.202796353 | 0 | 0 | 12081 | 508 |
| chr17_56356056_56344334_+11722-Kdm4b | chr17 | Kdm4b | 0 | 0.182309179 | 0 | 11723 | 759 |
| chr17_5731574_5712388_+19186-na | chr17 | na | 0 | 0.09115459 | 0 | 19187 | 19187 |
| chr17_5891899_5886997_+4902-Snx9 | chr17 | Snx9 | 1.622370823 | 0.729236718 | 0.903230112 | 4903 | 288 |
| chr17_5899495_5886997_+12498-Snx9 | chr17 | Snx9 | 0.202796353 | 0.182309179 | 0 | 12499 | 457 |
| chr17_5908699_5899327_+9372-Snx9 | chr17 | Snx9 | 0 | 0.182309179 | 0 | 9373 | 649 |
| chr17_5920673_5886997_+33676-Snx9 | chr17 | Snx9 | 0.202796353 | 0 | 0 | 33677 | 1276 |
| chr17_6067660_6060203_-7457-Serac1 | chr17 | Serac1 | 0.202796353 | 0 | 0.180646022 | 7458 | 660 |
| chr17_6139156_6106392_+32764-Tulp4 | chr17 | Tulp4 | 1.013981764 | 0.546927538 | 0 | 32765 | 32765 |
| chr17_6139156_6118296_+20860-Tulp4 | chr17 | Tulp4 | 0 | 0 | 0.180646022 | 20861 | 2017 |
| chr17_6143082_6137211_+5871-Tulp4 | chr17 | Tulp4 | 0 | 0 | 0.180646022 | 5872 | 5872 |
| chr17_6185298_6137211_+48087-Tulp4 | chr17 | Tulp4 | 1.825167175 | 2.005400973 | 0.722584089 | 48088 | 2237 |
| chr17_6207057_6176829_+30228-Tulp4 | chr17 | Tulp4 | 0.405592706 | 0 | 0 | 30229 | 774 |
| chr17_63269896_63257128_-12768-na | chr17 | na | 0.101398176 | 0 | 0 | 12769 | 12769 |
| chr17_63487969_63471408_-16561-Fbxl17 | chr17 | Fbxl17 | 0 | 0.182309179 | 0 | 16562 | 390 |
| chr17_63957317_63896018_+61299-Fer | chr17 | Fer | 0 | 0.182309179 | 0 | 61300 | 61300 |
| chr17_64293024_64287001_-6023-Pja2 | chr17 | Pja2 | 0.202796353 | 0.364618359 | 0.361292045 | 6024 | 535 |
| chr17_64297909_64287001_-10908-Pja2 | chr17 | Pja2 | 0 | 0 | 0.180646022 | 10909 | 721 |
| chr17_64313118_64287001_-26117-Pja2 | chr17 | Pja2 | 0 | 0.182309179 | 0 | 26118 | 2085 |
| chr17_64675247_64636484_+38763-Man2a1 | chr17 | Man2a1 | 0.202796353 | 0 | 0 | 38764 | 1225 |
| chr17_64740883_64731204_+9679-Man2a1 | chr17 | Man2a1 | 0.202796353 | 0 | 0 | 9680 | 614 |
| chr17_65816050_65803160_+12890-Ppp4r1 | chr17 | Ppp4r1 | 0 | 0 | 0.541938067 | 12891 | 994 |
| chr17_65817027_65803211_+13816-Ppp4r1 | chr17 | Ppp4r1 | 0 | 0.182309179 | 0.180646022 | 13817 | 1920 |
| chr17_65979600_65970150_-9450-Ankrd12 | chr17 | Ankrd12 | 0 | 0.182309179 | 0 | 9451 | 527 |
| chr17_65987493_65979502_-7991-Ankrd12 | chr17 | Ankrd12 | 0.202796353 | 0 | 0 | 7992 | 4835 |
| chr17_66817977_66809466_-8511-Ptprm | chr17 | Ptprm | 0.202796353 | 0 | 0.361292045 | 8512 | 478 |
| chr17_67063157_67063042_-115-Ptprm | chr17 | Ptprm | 0 | 0 | 0.180646022 | 116 | 116 |
| chr17_67095695_67042095_-53600-Ptprm | chr17 | Ptprm | 0.202796353 | 0 | 0.180646022 | 53601 | 936 |
| chr17_67304272_67042095_-262177-Ptprm | chr17 | Ptprm | 0 | 0.182309179 | 0 | 262178 | 262178 |
| chr17_67304272_67300284_-3988-na | chr17 | na | 0 | 0 | 0.180646022 | 3989 | 3989 |
| chr17_67304272_67300697_-3575-na | chr17 | na | 0 | 0 | 0.180646022 | 3576 | 3576 |
| chr17_67304272_67303725_-547-na | chr17 | na | 0 | 0.182309179 | 0 | 548 | 548 |
| chr17_67562150_67531860_-30290-Gm36201 | chr17 | Gm36201 | 0 | 0 | 0.180646022 | 30291 | 30291 |
| chr17_67643066_67642236_+830-na | chr17 | na | 0.101398176 | 0 | 0 | 831 | 831 |
| chr17_67861355_67855434_-5921-Arhgap28 | chr17 | Arhgap28 | 0 | 0.182309179 | 0 | 5922 | 452 |
| chr17_71052775_71052614_+161-Myom1 | chr17 | Myom1 | 0 | 0.182309179 | 0.180646022 | 162 | 162 |
| chr17_71067369_71052614_+14755-Myom1 | chr17 | Myom1 | 0 | 0 | 0.180646022 | 14756 | 561 |
| chr17_71092214_71087444_+4770-Myom1 | chr17 | Myom1 | 0 | 0 | 0.541938067 | 4771 | 427 |
| chr17_71126688_71122557_+4131-Myom1 | chr17 | Myom1 | 0 | 0.182309179 | 0.361292045 | 4132 | 669 |
| chr17_71632141_71625353_+6788-Wdr43 | chr17 | Wdr43 | 0 | 0 | 0.180646022 | 6789 | 518 |
| chr17_71810982_71789868_+21114-Clip4 | chr17 | Clip4 | 0 | 0.182309179 | 0 | 21115 | 1036 |
| chr17_71810982_71798957_+12025-Clip4 | chr17 | Clip4 | 0 | 0 | 0.180646022 | 12026 | 888 |
| chr17_71810982_71800799_+10183-Clip4 | chr17 | Clip4 | 0.202796353 | 1.093855076 | 0 | 10184 | 748 |
| chr17_7282869_7249292_+33577-Rps6ka2 | chr17 | Rps6ka2 | 0 | 0.182309179 | 0 | 33578 | 953 |
| chr17_73907745_73906010_-1735-Xdh | chr17 | Xdh | 0.405592706 | 0.364618359 | 0 | 1736 | 434 |
| chr17_73912540_73909247_-3293-Xdh | chr17 | Xdh | 0 | 0.182309179 | 0 | 3294 | 341 |
| chr17_74245087_74217027_-28060-Memo1 | chr17 | Memo1 | 0 | 0.182309179 | 0 | 28061 | 776 |
| chr17_74369285_74367248_+2037-Spast | chr17 | Spast | 0.202796353 | 0 | 0 | 2038 | 416 |
| chr17_74371504_74361070_+10434-Spast | chr17 | Spast | 0.202796353 | 0 | 0 | 10435 | 571 |
| chr17_74382040_74372259_+9781-Spast | chr17 | Spast | 0 | 0 | 0.180646022 | 9782 | 514 |
| chr17_74573631_74557901_+15730-Birc6 | chr17 | Birc6 | 0.202796353 | 0 | 0 | 15731 | 832 |
| chr17_74599967_74557901_+42066-Birc6 | chr17 | Birc6 | 0 | 0.182309179 | 0 | 42067 | 3739 |
| chr17_74612345_74557901_+54444-Birc6 | chr17 | Birc6 | 0 | 0.364618359 | 0 | 54445 | 5557 |
| chr17_74662818_74618436_+44382-Birc6 | chr17 | Birc6 | 0 | 0.182309179 | 0 | 44383 | 6221 |
| chr17_74747764_74724618_+23146-Ttc27 | chr17 | Ttc27 | 0 | 0 | 0.180646022 | 23147 | 656 |
| chr17_75227241_75225156_+2085-Ltbp1 | chr17 | Ltbp1 | 0.608389058 | 1.093855076 | 0.722584089 | 2086 | 500 |
| chr17_75252415_75225156_+27259-Ltbp1 | chr17 | Ltbp1 | 0 | 0 | 0.180646022 | 27260 | 675 |
| chr17_75290140_75282328_+7812-Ltbp1 | chr17 | Ltbp1 | 0 | 0.182309179 | 0 | 7813 | 325 |
| chr17_75296343_75273867_+22476-Ltbp1 | chr17 | Ltbp1 | 0.202796353 | 0 | 0 | 22477 | 1113 |
| chr17_75503389_75488817_+14572-Rasgrp3 | chr17 | Rasgrp3 | 0 | 0.182309179 | 0 | 14573 | 1349 |
| chr17_78315746_78302987_+12759-Crim1 | chr17 | Crim1 | 0.405592706 | 0 | 0 | 12760 | 503 |
| chr17_78335355_78237742_+97613-Crim1 | chr17 | Crim1 | 0 | 0 | 0.180646022 | 97614 | 1170 |
| chr17_78818917_78788205_-30712-Heatr5b | chr17 | Heatr5b | 0 | 0.364618359 | 0 | 30713 | 2520 |
| chr17_79659473_79627462_+32011-Rmdn2 | chr17 | Rmdn2 | 0.405592706 | 0 | 0 | 32012 | 1257 |
| chr17_79659473_79650302_+9171-Rmdn2 | chr17 | Rmdn2 | 0.405592706 | 0 | 0.541938067 | 9172 | 339 |
| chr17_79672573_79650302_+22271-Rmdn2 | chr17 | Rmdn2 | 0.811185411 | 0.364618359 | 0.541938067 | 22272 | 727 |
| chr17_79876046_79864703_-11343-Atl2 | chr17 | Atl2 | 0.608389058 | 0 | 0 | 11344 | 485 |
| chr17_80053641_80030341_-23300-Hnrnpll | chr17 | Hnrnpll | 0 | 0.182309179 | 0 | 23301 | 1667 |
| chr17_80053641_80044494_-9147-Hnrnpll | chr17 | Hnrnpll | 0 | 0.546927538 | 0 | 9148 | 768 |
| chr17_80150170_80138013_+12157-Galm | chr17 | Galm | 0 | 0 | 0.180646022 | 12158 | 444 |
| chr17_80644845_80636720_-8125-Map4k3 | chr17 | Map4k3 | 0.202796353 | 0 | 0 | 8126 | 467 |
| chr17_81445593_81408067_-37526-Slc8a1 | chr17 | Slc8a1 | 0 | 0.182309179 | 0 | 37527 | 737 |
| chr17_81445593_81428182_-17411-Slc8a1 | chr17 | Slc8a1 | 0 | 0.546927538 | 0 | 17412 | 461 |
| chr17_81649638_81647809_-1829-Slc8a1 | chr17 | Slc8a1 | 2.83914894 | 3.828492767 | 2.167752268 | 1830 | 1830 |
| chr17_82956485_82942428_-14057-Gm34265 | chr17 | Gm34265 | 0 | 0.182309179 | 0 | 14058 | 447 |
| chr17_84444509_84443818_-691-Thada | chr17 | Thada | 0 | 0.182309179 | 0.361292045 | 692 | 692 |
| chr17_84464501_84451709_-12792-Thada | chr17 | Thada | 0 | 0.182309179 | 0 | 12793 | 790 |
| chr17_84740668_84731145_-9523-Lrpprc | chr17 | Lrpprc | 0 | 0.182309179 | 0 | 9524 | 392 |
| chr17_84756285_84748961_-7324-Lrpprc | chr17 | Lrpprc | 0 | 0.182309179 | 0.180646022 | 7325 | 859 |
| chr17_84769702_84753405_-16297-Lrpprc | chr17 | Lrpprc | 0 | 0 | 0.180646022 | 16298 | 581 |
| chr17_85083315_85070428_-12887-Prepl | chr17 | Prepl | 0 | 0 | 0.180646022 | 12888 | 1310 |
| chr17_85083315_85075866_-7449-Prepl | chr17 | Prepl | 0 | 0 | 0.180646022 | 7450 | 936 |
| chr17_85783451_84766993_-1016458-Lrpprc | chr17 | Lrpprc | 0 | 0.09115459 | 0 | 1016459 | 1016459 |
| chr17_86001607_85997506_-4101-Srbd1 | chr17 | Srbd1 | 0 | 0 | 0.361292045 | 4102 | 372 |
| chr17_86109334_86095867_-13467-na | chr17 | na | 0.202796353 | 0 | 0 | 13468 | 13468 |
| chr17_86120746_86051086_-69660-Srbd1 | chr17 | Srbd1 | 0.202796353 | 0 | 0 | 69661 | 977 |
| chr17_86120746_86098512_-22234-Srbd1 | chr17 | Srbd1 | 0.202796353 | 1.640782614 | 1.083876134 | 22235 | 802 |
| chr17_86120746_86109227_-11519-Srbd1 | chr17 | Srbd1 | 0 | 0 | 0.180646022 | 11520 | 445 |
| chr17_86496111_86488160_+7951-Prkce | chr17 | Prkce | 0.405592706 | 0.364618359 | 0 | 7952 | 614 |
| chr17_86809789_86809358_+431-Epas1 | chr17 | Epas1 | 0 | 0.182309179 | 0 | 432 | 325 |
| chr17_87992356_87991929_-427-Fbxo11 | chr17 | Fbxo11 | 0.101398176 | 0 | 0 | 428 | 346 |
| chr17_88015712_88008594_-7118-Fbxo11 | chr17 | Fbxo11 | 0.202796353 | 0.182309179 | 0.180646022 | 7119 | 1163 |
| chr17_88580551_88542845_+37706-Ppp1r21 | chr17 | Ppp1r21 | 0 | 0.182309179 | 0 | 37707 | 2028 |
| chr18_10140888_10129305_-11583-Rock1 | chr18 | Rock1 | 0 | 0.364618359 | 0 | 11584 | 876 |
| chr18_10586041_10582055_-3986-na | chr18 | na | 0 | 0.09115459 | 0 | 3987 | 3987 |
| chr18_10752027_10747356_+4671-Mib1 | chr18 | Mib1 | 0 | 0 | 0.361292045 | 4672 | 377 |
| chr18_12070131_12064204_-5927-na | chr18 | na | 0 | 0.09115459 | 0 | 5928 | 5928 |
| chr18_12106003_12074618_-31385-Tmem241 | chr18 | Tmem241 | 0.202796353 | 0 | 0 | 31386 | 336 |
| chr18_12191023_12188209_+2814-3110002H16Rik | chr18 | 3110002H16Rik | 0 | 0 | 0.180646022 | 2815 | 2815 |
| chr18_13533397_13526697_-6700-Gm36802 | chr18 | Gm36802 | 0 | 0.182309179 | 0 | 6701 | 444 |
| chr18_13542289_13526697_-15592-Gm36802 | chr18 | Gm36802 | 0 | 0 | 0.361292045 | 15593 | 15593 |
| chr18_13546838_13526697_-20141-Gm36802 | chr18 | Gm36802 | 0.405592706 | 0 | 0 | 20142 | 20142 |
| chr18_13689024_13683062_-5962-Zfp521 | chr18 | Zfp521 | 0.405592706 | 0 | 0 | 5963 | 5963 |
| chr18_13717747_13715141_-2606-Zfp521 | chr18 | Zfp521 | 0 | 0 | 0.361292045 | 2607 | 248 |
| chr18_13847134_13817238_-29896-Zfp521 | chr18 | Zfp521 | 0 | 0.182309179 | 0 | 29897 | 3438 |
| chr18_13847134_13843782_-3352-Zfp521 | chr18 | Zfp521 | 0.202796353 | 0 | 0 | 3353 | 3353 |
| chr18_13939141_13938962_-179-Zfp521 | chr18 | Zfp521 | 0.202796353 | 0 | 0 | 180 | 180 |
| chr18_14813690_14813091_+599-Taf4b | chr18 | Taf4b | 0.202796353 | 0 | 0 | 600 | 600 |
| chr18_14844470_14796913_+47557-Taf4b | chr18 | Taf4b | 0 | 0 | 0.180646022 | 47558 | 1958 |
| chr18_21148904_21147732_-1172-Garem | chr18 | Garem | 0 | 0.182309179 | 0 | 1173 | 1173 |
| chr18_23621474_23583084_+38390-Dtna | chr18 | Dtna | 0 | 0 | 0.361292045 | 38391 | 991 |
| chr18_24612525_24609630_+2895-Elp2 | chr18 | Elp2 | 0 | 0.182309179 | 0 | 2896 | 306 |
| chr18_25028169_25020662_+7507-Fhod3 | chr18 | Fhod3 | 0.811185411 | 0.182309179 | 0.180646022 | 7508 | 558 |
| chr18_25066425_25020662_+45763-Fhod3 | chr18 | Fhod3 | 0 | 0.182309179 | 0 | 45764 | 953 |
| chr18_25420075_25384865_+35210-AW554918 | chr18 | AW554918 | 0 | 0 | 0.090323011 | 35211 | 35211 |
| chr18_30303074_30294336_+8738-Pik3c3 | chr18 | Pik3c3 | 0 | 0.182309179 | 0 | 8739 | 434 |
| chr18_31607264_31594577_-12687-Slc25a46 | chr18 | Slc25a46 | 0 | 0.182309179 | 0 | 12688 | 457 |
| chr18_31636099_31619365_+16734-Sap130 | chr18 | Sap130 | 0 | 0 | 0.361292045 | 16735 | 16735 |
| chr18_31636099_31633790_+2309-Sap130 | chr18 | Sap130 | 0.202796353 | 0.364618359 | 0 | 2310 | 2310 |
| chr18_31636099_31635982_+117-Sap130 | chr18 | Sap130 | 0 | 0.182309179 | 0 | 118 | 118 |
| chr18_31772114_31771670_+444-Ammecr1l | chr18 | Ammecr1l | 0 | 0.364618359 | 0 | 445 | 445 |
| chr18_31881282_31878024_+3258-Wdr33 | chr18 | Wdr33 | 0 | 0 | 0.180646022 | 3259 | 472 |
| chr18_32424967_32412421_+12546-Bin1 | chr18 | Bin1 | 0 | 0.182309179 | 0 | 12547 | 885 |
| chr18_32424967_32419843_+5124-Bin1 | chr18 | Bin1 | 0 | 0.182309179 | 0 | 5125 | 438 |
| chr18_32432093_32424820_+7273-Bin1 | chr18 | Bin1 | 0.202796353 | 0 | 0 | 7274 | 700 |
| chr18_32432425_32424820_+7605-Bin1 | chr18 | Bin1 | 2.027963528 | 1.640782614 | 1.445168179 | 7606 | 802 |
| chr18_32863262_32861027_+2235-Wdr36 | chr18 | Wdr36 | 0 | 0 | 0.541938067 | 2236 | 446 |
| chr18_32863262_32861050_+2212-Wdr36 | chr18 | Wdr36 | 0 | 0.182309179 | 0 | 2213 | 423 |
| chr18_33861237_33824459_-36778-Epb41l4a | chr18 | Epb41l4a | 0 | 0.364618359 | 0 | 36779 | 707 |
| chr18_33891476_33874142_-17334-Epb41l4a | chr18 | Epb41l4a | 0 | 0.182309179 | 0 | 17335 | 595 |
| chr18_34221276_32406100_+1815176-Wdr36 | chr18 | Wdr36 | 0 | 0.364618359 | 0 | 1815177 | 1815177 |
| chr18_34289942_34268298_+21644-Apc | chr18 | Apc | 0.202796353 | 0 | 0 | 21645 | 693 |
| chr18_34300061_34266058_+34003-Apc | chr18 | Apc | 0 | 0 | 0.180646022 | 34004 | 1432 |
| chr18_34301315_34296031_+5284-Apc | chr18 | Apc | 0.405592706 | 0 | 0 | 5285 | 628 |
| chr18_34417049_34412681_+4368-Pkd2l2 | chr18 | Pkd2l2 | 0.405592706 | 0 | 0 | 4369 | 613 |
| chr18_34494776_34451208_-43568-Fam13b | chr18 | Fam13b | 0 | 0 | 0.361292045 | 43569 | 1694 |
| chr18_34498159_34473550_-24609-Fam13b | chr18 | Fam13b | 0 | 0.182309179 | 0 | 24610 | 1118 |
| chr18_34813561_34793004_+20557-Kdm3b | chr18 | Kdm3b | 0.202796353 | 0 | 0 | 20558 | 3007 |
| chr18_34813561_34803450_+10111-Kdm3b | chr18 | Kdm3b | 0.202796353 | 0 | 0 | 10112 | 2419 |
| chr18_34824958_34812372_+12586-Kdm3b | chr18 | Kdm3b | 0 | 0.182309179 | 0 | 12587 | 1141 |
| chr18_36581215_36575019_+6196-Ankhd1 | chr18 | Ankhd1 | 0 | 0 | 0.180646022 | 6197 | 456 |
| chr18_36589581_36581068_+8513-Ankhd1 | chr18 | Ankhd1 | 0 | 0 | 0.361292045 | 8514 | 530 |
| chr18_36591541_36581068_+10473-Ankhd1 | chr18 | Ankhd1 | 0.405592706 | 0 | 0.541938067 | 10474 | 625 |
| chr18_36594322_36581068_+13254-Ankhd1 | chr18 | Ankhd1 | 0 | 0 | 0.180646022 | 13255 | 863 |
| chr18_36634805_36632067_+2738-Ankhd1 | chr18 | Ankhd1 | 0 | 0 | 0.180646022 | 2739 | 761 |
| chr18_36638708_36632067_+6641-Ankhd1 | chr18 | Ankhd1 | 0 | 0 | 0.180646022 | 6642 | 1100 |
| chr18_36643094_36632067_+11027-Ankhd1 | chr18 | Ankhd1 | 0 | 0 | 0.180646022 | 11028 | 1482 |
| chr18_36648740_36632067_+16673-Ankhd1 | chr18 | Ankhd1 | 0.405592706 | 0 | 0 | 16674 | 3831 |
| chr18_36658138_36632067_+26071-Ankhd1 | chr18 | Ankhd1 | 0.202796353 | 0.182309179 | 0.180646022 | 26072 | 4574 |
| chr18_36788670_36787502_+1168-Hars2 | chr18 | Hars2 | 0 | 0 | 0.361292045 | 1169 | 427 |
| chr18_38143327_38120117_-23210-Gm30188 | chr18 | Gm30188 | 0 | 0.182309179 | 0 | 23211 | 556 |
| chr18_38456121_38451555_+4566-Ndfip1 | chr18 | Ndfip1 | 0.405592706 | 0 | 0 | 4567 | 280 |
| chr18_42246077_42217468_-28609-Lars | chr18 | Lars | 0 | 0 | 0.361292045 | 28610 | 2475 |
| chr18_42324198_42320764_+3434-Rbm27 | chr18 | Rbm27 | 0.202796353 | 0 | 0 | 3435 | 595 |
| chr18_42523066_42519368_+3698-Tcerg1 | chr18 | Tcerg1 | 0 | 0.182309179 | 0 | 3699 | 379 |
| chr18_42538056_42519368_+18688-Tcerg1 | chr18 | Tcerg1 | 0 | 0 | 0.361292045 | 18689 | 1566 |
| chr18_44354281_44336889_+17392-Myot | chr18 | Myot | 0 | 0.182309179 | 0 | 17393 | 1256 |
| chr18_44354935_44345012_+9923-Myot | chr18 | Myot | 0 | 0.182309179 | 0 | 9924 | 641 |
| chr18_44468521_44459799_-8722-Mcc | chr18 | Mcc | 0 | 0 | 0.180646022 | 8723 | 429 |
| chr18_45592424_45558290_+34134-Kcnn2 | chr18 | Kcnn2 | 0 | 0.182309179 | 0 | 34135 | 1988 |
| chr18_46434366_46423309_-11057-na | chr18 | na | 0 | 0 | 0.180646022 | 11058 | 11058 |
| chr18_46434366_46433132_-1234-na | chr18 | na | 0.405592706 | 0.364618359 | 0 | 1235 | 1235 |
| chr18_47300144_47270609_-29535-Sema6a | chr18 | Sema6a | 0 | 0.182309179 | 0 | 29536 | 1727 |
| chr18_49729326_49723622_-5704-Dtwd2 | chr18 | Dtwd2 | 0 | 0.364618359 | 0 | 5705 | 536 |
| chr18_5058774_5040148_+18626-Svil | chr18 | Svil | 0 | 0 | 0.180646022 | 18627 | 2128 |
| chr18_5064690_5063194_+1496-Svil | chr18 | Svil | 0 | 0.182309179 | 0 | 1497 | 797 |
| chr18_5074004_5037108_+36896-Svil | chr18 | Svil | 0.202796353 | 0 | 0 | 36897 | 3587 |
| chr18_5074004_5055980_+18024-Svil | chr18 | Svil | 0 | 0 | 0.361292045 | 18025 | 2533 |
| chr18_5088814_5063194_+25620-Svil | chr18 | Svil | 0 | 0.729236718 | 0 | 25621 | 1472 |
| chr18_5108708_5082783_+25925-Svil | chr18 | Svil | 0 | 0 | 0.361292045 | 25926 | 2461 |
| chr18_5117097_5115978_+1119-Svil | chr18 | Svil | 0 | 0 | 0.722584089 | 1120 | 269 |
| chr18_5214919_5210877_-4042-Zfp438 | chr18 | Zfp438 | 6.286686938 | 5.469275382 | 3.612920447 | 4043 | 2123 |
| chr18_53203119_53189655_+13464-Snx2 | chr18 | Snx2 | 0 | 0 | 0.180646022 | 13465 | 614 |
| chr18_54988706_54987611_-1095-Zfp608 | chr18 | Zfp608 | 0.405592706 | 0 | 0 | 1096 | 1096 |
| chr18_5641518_5620401_+21117-Zeb1 | chr18 | Zeb1 | 0.405592706 | 0 | 0 | 21118 | 21118 |
| chr18_5641518_5627139_+14379-Zeb1 | chr18 | Zeb1 | 1.216778117 | 1.276164256 | 0.722584089 | 14380 | 14380 |
| chr18_5641518_5633204_+8314-Zeb1 | chr18 | Zeb1 | 0 | 0.729236718 | 0 | 8315 | 8315 |
| chr18_56491999_56488751_+3248-Gramd3 | chr18 | Gramd3 | 0 | 0 | 0.180646022 | 3249 | 428 |
| chr18_5705243_5627139_+78104-na | chr18 | na | 0 | 0 | 0.180646022 | 78105 | 78105 |
| chr18_5705243_5633204_+72039-Zeb1 | chr18 | Zeb1 | 0.202796353 | 0.729236718 | 0 | 72040 | 355 |
| chr18_5773094_5766221_+6873-Zeb1 | chr18 | Zeb1 | 0 | 0.911545897 | 0 | 6874 | 2652 |
| chr18_57906104_57898059_+8045-Slc12a2 | chr18 | Slc12a2 | 0 | 0 | 0.180646022 | 8046 | 821 |
| chr18_58673366_58671208_+2158-Isoc1 | chr18 | Isoc1 | 0 | 0 | 0.180646022 | 2159 | 441 |
| chr18_60556370_60546221_+10149-Dctn4 | chr18 | Dctn4 | 0 | 0 | 0.180646022 | 10150 | 701 |
| chr18_6070091_6033141_-36950-Arhgap12 | chr18 | Arhgap12 | 0.202796353 | 0 | 0 | 36951 | 1264 |
| chr18_6070091_6057517_-12574-Arhgap12 | chr18 | Arhgap12 | 0 | 0.182309179 | 0 | 12575 | 684 |
| chr18_6070091_6061847_-8244-Arhgap12 | chr18 | Arhgap12 | 0.405592706 | 0.182309179 | 0 | 8245 | 609 |
| chr18_60713632_60712723_-909-Ndst1 | chr18 | Ndst1 | 0.202796353 | 0.182309179 | 0 | 910 | 910 |
| chr18_6073286_6061847_-11439-Arhgap12 | chr18 | Arhgap12 | 0.405592706 | 0 | 0 | 11440 | 11440 |
| chr18_6073286_6061847_-11439-na | chr18 | na | 0 | 0 | 0.180646022 | 11440 | 11440 |
| chr18_6073286_6064341_-8945-na | chr18 | na | 0 | 0.09115459 | 0 | 8946 | 8946 |
| chr18_61159212_61140791_-18421-Hmgxb3 | chr18 | Hmgxb3 | 0 | 0.364618359 | 0 | 18422 | 1645 |
| chr18_61309682_61302619_-7063-Ppargc1b | chr18 | Ppargc1b | 0 | 0 | 0.180646022 | 7064 | 1105 |
| chr18_61524003_61517995_-6008-Arhgef37 | chr18 | Arhgef37 | 0.405592706 | 0 | 0 | 6009 | 469 |
| chr18_61577252_61575410_+1842-Csnk1a1 | chr18 | Csnk1a1 | 0 | 0.546927538 | 0 | 1843 | 294 |
| chr18_61580487_61575410_+5077-Csnk1a1 | chr18 | Csnk1a1 | 0.608389058 | 0 | 0 | 5078 | 550 |
| chr18_61857185_61839690_-17495-Ablim3 | chr18 | Ablim3 | 0.405592706 | 0.182309179 | 0 | 17496 | 606 |
| chr18_62083529_62044835_-38694-Gm35887 | chr18 | Gm35887 | 0.202796353 | 0 | 0 | 38695 | 996 |
| chr18_62541046_62516964_-24082-Fbxo38 | chr18 | Fbxo38 | 0.202796353 | 0 | 0 | 24083 | 2102 |
| chr18_63677001_63671582_-5419-Txnl1 | chr18 | Txnl1 | 0 | 0.182309179 | 0 | 5420 | 471 |
| chr18_63760785_63735579_+25206-Wdr7 | chr18 | Wdr7 | 0 | 0 | 0.180646022 | 25207 | 1272 |
| chr18_63796281_63777528_+18753-Wdr7 | chr18 | Wdr7 | 0.202796353 | 0 | 0 | 18754 | 1198 |
| chr18_65198726_65174045_+24681-Nedd4l | chr18 | Nedd4l | 0.608389058 | 0.182309179 | 1.083876134 | 24682 | 975 |
| chr18_65198726_65178919_+19807-Nedd4l | chr18 | Nedd4l | 0 | 0.911545897 | 0 | 19808 | 777 |
| chr18_65198726_65191437_+7289-Nedd4l | chr18 | Nedd4l | 0 | 0 | 0.180646022 | 7290 | 585 |
| chr18_65350708_65349028_-1680-Alpk2 | chr18 | Alpk2 | 0 | 0.729236718 | 0 | 1681 | 1681 |
| chr18_65464115_65444832_+19283-Malt1 | chr18 | Malt1 | 0 | 0 | 0.361292045 | 19284 | 1105 |
| chr18_65656632_65582897_+73735-Zfp532 | chr18 | Zfp532 | 0 | 0 | 0.180646022 | 73736 | 6399 |
| chr18_67434229_67420986_-13243-Afg3l2 | chr18 | Afg3l2 | 0.202796353 | 0 | 0 | 13244 | 1000 |
| chr18_68106691_68064217_+42474-Ldlrad4 | chr18 | Ldlrad4 | 0.202796353 | 0 | 0 | 42475 | 611 |
| chr18_69465447_69461862_+3585-na | chr18 | na | 0 | 0.09115459 | 0 | 3586 | 3586 |
| chr18_70593340_70580653_+12687-Mbd2 | chr18 | Mbd2 | 0 | 0 | 0.722584089 | 12688 | 298 |
| chr18_73658898_73648885_-10013-Smad4 | chr18 | Smad4 | 0 | 0.182309179 | 0 | 10014 | 641 |
| chr18_73662867_73648885_-13982-Smad4 | chr18 | Smad4 | 0.202796353 | 0.182309179 | 0.180646022 | 13983 | 851 |
| chr18_73662867_73657195_-5672-Smad4 | chr18 | Smad4 | 0.405592706 | 0 | 0.722584089 | 5673 | 498 |
| chr18_74274773_74273735_+1038-Mbd1 | chr18 | Mbd1 | 0.202796353 | 0 | 0 | 1039 | 400 |
| chr18_7444105_7403186_-40919-Mpp7 | chr18 | Mpp7 | 0 | 0.182309179 | 0 | 40920 | 883 |
| chr18_74799174_74798312_+862-Acaa2 | chr18 | Acaa2 | 0 | 0.182309179 | 0.180646022 | 863 | 306 |
| chr18_75063173_75043079_+20094-Dym | chr18 | Dym | 0 | 0.364618359 | 0 | 20095 | 545 |
| chr18_75063173_75055281_+7892-Dym | chr18 | Dym | 0 | 0.364618359 | 0 | 7893 | 301 |
| chr18_75082507_75052981_+29526-Dym | chr18 | Dym | 0.202796353 | 0.182309179 | 0 | 29527 | 623 |
| chr18_75126919_75080203_+46716-Dym | chr18 | Dym | 0.405592706 | 0 | 0 | 46717 | 966 |
| chr18_75126919_75119111_+7808-Dym | chr18 | Dym | 0.202796353 | 0 | 0 | 7809 | 514 |
| chr18_75199265_75114790_+84475-Dym | chr18 | Dym | 0 | 0.182309179 | 0.180646022 | 84476 | 800 |
| chr18_75521869_75519739_-2130-Ctif | chr18 | Ctif | 0.202796353 | 0.182309179 | 0 | 2131 | 862 |
| chr18_77007883_76977219_-30664-Katnal2 | chr18 | Katnal2 | 0 | 0.182309179 | 0 | 30665 | 1375 |
| chr18_77009139_76977219_-31920-Katnal2 | chr18 | Katnal2 | 0 | 0 | 0.361292045 | 31921 | 31921 |
| chr18_77105889_77097249_+8640-Pias2 | chr18 | Pias2 | 0 | 0.546927538 | 0.541938067 | 8641 | 560 |
| chr18_77674684_77673900_-784-8030462N17Rik | chr18 | 8030462N17Rik | 0 | 1.093855076 | 0 | 785 | 785 |
| chr18_77875919_77871568_+4351-Pstpip2 | chr18 | Pstpip2 | 0 | 0.182309179 | 0 | 4352 | 404 |
| chr18_77962538_77959030_+3508-Epg5 | chr18 | Epg5 | 0 | 0.546927538 | 0 | 3509 | 580 |
| chr18_77981495_77975837_+5658-Epg5 | chr18 | Epg5 | 0 | 0 | 0.180646022 | 5659 | 741 |
| chr18_78924042_78916692_-7350-Setbp1 | chr18 | Setbp1 | 0.202796353 | 0 | 0 | 7351 | 7351 |
| chr18_80667160_80649832_-17328-Nfatc1 | chr18 | Nfatc1 | 0.202796353 | 0 | 0 | 17329 | 706 |
| chr18_80667160_80663295_-3865-Nfatc1 | chr18 | Nfatc1 | 0.608389058 | 0.364618359 | 0 | 3866 | 517 |
| chr18_80682318_80635691_-46627-Nfatc1 | chr18 | Nfatc1 | 0 | 0.182309179 | 0 | 46628 | 1252 |
| chr18_80698656_80635691_-62965-Nfatc1 | chr18 | Nfatc1 | 0.811185411 | 0 | 0 | 62966 | 2354 |
| chr18_80698656_80682159_-16497-Nfatc1 | chr18 | Nfatc1 | 0.405592706 | 0 | 0.180646022 | 16498 | 1262 |
| chr18_80698656_80697555_-1101-Nfatc1 | chr18 | Nfatc1 | 0.405592706 | 0 | 0.361292045 | 1102 | 1102 |
| chr18_80909705_80836355_-73350-Atp9b | chr18 | Atp9b | 0 | 0 | 0.180646022 | 73351 | 726 |
| chr18_80917878_80858544_-59334-Atp9b | chr18 | Atp9b | 1.216778117 | 0 | 1.806460223 | 59335 | 817 |
| chr18_80917878_80869742_-48136-Atp9b | chr18 | Atp9b | 0 | 0.546927538 | 0 | 48137 | 722 |
| chr18_80917878_80877315_-40563-Atp9b | chr18 | Atp9b | 0 | 0 | 1.083876134 | 40564 | 607 |
| chr18_80917878_80909592_-8286-Atp9b | chr18 | Atp9b | 0.811185411 | 0.729236718 | 0 | 8287 | 439 |
| chr18_82646241_82620318_-25923-Zfp236 | chr18 | Zfp236 | 0.202796353 | 0.182309179 | 0 | 25924 | 2759 |
| chr18_82682287_82656949_-25338-Zfp236 | chr18 | Zfp236 | 0 | 0.364618359 | 0 | 25339 | 1362 |
| chr18_82682287_82664838_-17449-Zfp236 | chr18 | Zfp236 | 0 | 1.276164256 | 1.083876134 | 17450 | 929 |
| chr18_82682287_82668572_-13715-Zfp236 | chr18 | Zfp236 | 0.405592706 | 0 | 0.541938067 | 13716 | 785 |
| chr18_82682287_82671686_-10601-Zfp236 | chr18 | Zfp236 | 0 | 0 | 0.361292045 | 10602 | 612 |
| chr18_82682287_82674307_-7980-Zfp236 | chr18 | Zfp236 | 0 | 0.182309179 | 0 | 7981 | 487 |
| chr18_82685339_82668572_-16767-Zfp236 | chr18 | Zfp236 | 0 | 0.182309179 | 0 | 16768 | 941 |
| chr18_82687138_82664838_-22300-Zfp236 | chr18 | Zfp236 | 0.202796353 | 0 | 0 | 22301 | 22301 |
| chr18_82910992_82500727_+410265-Mbp | chr18 | Mbp | 0 | 0.182309179 | 0 | 410266 | 410266 |
| chr18_82957449_82955524_+1925-Zfp516 | chr18 | Zfp516 | 2.027963528 | 1.640782614 | 0.722584089 | 1926 | 1926 |
| chr18_82988198_82986744_+1454-Zfp516 | chr18 | Zfp516 | 0.405592706 | 0.182309179 | 0.361292045 | 1455 | 1455 |
| chr18_82994537_82986744_+7793-Zfp516 | chr18 | Zfp516 | 0.405592706 | 0 | 0.180646022 | 7794 | 1643 |
| chr18_84354691_84342946_-11745-Zfp407 | chr18 | Zfp407 | 0 | 0.182309179 | 0 | 11746 | 372 |
| chr18_84563046_84432360_-130686-Zfp407 | chr18 | Zfp407 | 0 | 0.182309179 | 0 | 130687 | 4910 |
| chr18_84563046_84552870_-10176-Zfp407 | chr18 | Zfp407 | 0 | 0 | 0.180646022 | 10177 | 4835 |
| chr19_10623638_10621541_+2097-Ddb1 | chr19 | Ddb1 | 0.202796353 | 0 | 0 | 2098 | 867 |
| chr19_11925226_11923801_+1425-Patl1 | chr19 | Patl1 | 0 | 0.182309179 | 0 | 1426 | 308 |
| chr19_11931656_11929875_+1781-Patl1 | chr19 | Patl1 | 0 | 0 | 0.180646022 | 1782 | 305 |
| chr19_11937379_11932138_+5241-Patl1 | chr19 | Patl1 | 0.405592706 | 0 | 0 | 5242 | 467 |
| chr19_11990782_11978237_+12545-Osbp | chr19 | Osbp | 0.405592706 | 0 | 0 | 12546 | 1039 |
| chr19_14468271_14464309_-3962-Tle4 | chr19 | Tle4 | 0.202796353 | 0 | 0 | 3963 | 731 |
| chr19_16746078_16744824_-1254-Vps13a | chr19 | Vps13a | 0 | 0 | 0.090323011 | 1255 | 369 |
| chr19_19251363_19244067_+7296-na | chr19 | na | 0.101398176 | 0.182309179 | 0.180646022 | 7297 | 7297 |
| chr19_19258635_19244067_+14568-na | chr19 | na | 0 | 0.09115459 | 0 | 14569 | 14569 |
| chr19_19284625_19231756_+52869-Gm41815 | chr19 | Gm41815 | 0.202796353 | 0 | 0 | 52870 | 52870 |
| chr19_19439426_19420206_+19220-na | chr19 | na | 0.202796353 | 0 | 0 | 19221 | 19221 |
| chr19_21684305_21678353_+5952-Abhd17b | chr19 | Abhd17b | 0.202796353 | 0 | 0 | 5953 | 858 |
| chr19_24120903_24112746_-8157-Tjp2 | chr19 | Tjp2 | 0 | 0.182309179 | 0 | 8158 | 538 |
| chr19_24733677_24727669_-6008-Pgm5 | chr19 | Pgm5 | 0 | 0.364618359 | 0 | 6009 | 320 |
| chr19_25411764_25409086_+2678-Kank1 | chr19 | Kank1 | 0.202796353 | 0.364618359 | 0.180646022 | 2679 | 2679 |
| chr19_26654511_26647021_+7490-Smarca2 | chr19 | Smarca2 | 0.405592706 | 0 | 0 | 7491 | 646 |
| chr19_26683922_26676634_+7288-Smarca2 | chr19 | Smarca2 | 0.202796353 | 0 | 0 | 7289 | 699 |
| chr19_26706231_26671084_+35147-Smarca2 | chr19 | Smarca2 | 0.405592706 | 0 | 0 | 35148 | 1521 |
| chr19_26752019_26720851_-31168-na | chr19 | na | 0 | 0 | 0.180646022 | 31169 | 31169 |
| chr19_27248026_27217543_+30483-Vldlr | chr19 | Vldlr | 0 | 0.182309179 | 0.361292045 | 30484 | 2658 |
| chr19_27813690_27793611_-20079-Rfx3 | chr19 | Rfx3 | 0.202796353 | 0 | 0 | 20080 | 841 |
| chr19_28540408_28530873_-9535-Glis3 | chr19 | Glis3 | 0 | 0.182309179 | 0.180646022 | 9536 | 1322 |
| chr19_29134503_29115723_+18780-Rcl1 | chr19 | Rcl1 | 0.202796353 | 0.182309179 | 0 | 18781 | 835 |
| chr19_29602780_29577583_+25197-Ric1 | chr19 | Ric1 | 0.202796353 | 0 | 0 | 25198 | 2885 |
| chr19_29776310_29753600_-22710-na | chr19 | na | 0 | 0 | 0.090323011 | 22711 | 22711 |
| chr19_29786449_29753600_-32849-9930021J03Rik | chr19 | 9930021J03Rik | 0 | 0.182309179 | 0.180646022 | 32850 | 1619 |
| chr19_29850668_29838861_-11807-Gm41832 | chr19 | Gm41832 | 0 | 0.364618359 | 0 | 11808 | 11808 |
| chr19_32068615_32043481_-25134-Asah2 | chr19 | Asah2 | 0 | 0 | 0.180646022 | 25135 | 1173 |
| chr19_32160363_32159524_-839-Sgms1 | chr19 | Sgms1 | 0 | 0.182309179 | 0 | 840 | 840 |
| chr19_32269981_32223509_-46472-Sgms1 | chr19 | Sgms1 | 0.202796353 | 0 | 0 | 46473 | 46473 |
| chr19_32269981_32247994_-21987-Sgms1 | chr19 | Sgms1 | 0 | 0.364618359 | 0 | 21988 | 21988 |
| chr19_32289631_32247994_-41637-na | chr19 | na | 0.101398176 | 0 | 0 | 41638 | 41638 |
| chr19_34841109_34821100_-20009-Pank1 | chr19 | Pank1 | 0 | 0 | 0.180646022 | 20010 | 908 |
| chr19_3507355_3469641_-37714-Ppp6r3 | chr19 | Ppp6r3 | 0 | 0.182309179 | 0 | 37715 | 1307 |
| chr19_3518382_3478278_-40104-Ppp6r3 | chr19 | Ppp6r3 | 0 | 0.364618359 | 0.361292045 | 40105 | 1218 |
| chr19_3605494_3604002_-1492-Lrp5 | chr19 | Lrp5 | 0 | 0.182309179 | 0 | 1493 | 400 |
| chr19_3615879_3597244_-18635-Lrp5 | chr19 | Lrp5 | 0 | 0.364618359 | 0 | 18636 | 1909 |
| chr19_3652330_3647528_-4802-Lrp5 | chr19 | Lrp5 | 0 | 0 | 0.180646022 | 4803 | 527 |
| chr19_36587430_36569373_+18057-Hectd2 | chr19 | Hectd2 | 0 | 0 | 0.361292045 | 18058 | 527 |
| chr19_36615643_36583936_+31707-Hectd2 | chr19 | Hectd2 | 0.405592706 | 0 | 0 | 31708 | 1945 |
| chr19_36676799_36645684_-31115-1500017E21Rik | chr19 | 1500017E21Rik | 0.101398176 | 0 | 0 | 31116 | 31116 |
| chr19_36676799_36645684_-31115-na | chr19 | na | 0 | 0.09115459 | 0 | 31116 | 31116 |
| chr19_36676799_36675832_-967-na | chr19 | na | 0 | 0.182309179 | 0.180646022 | 968 | 968 |
| chr19_36888895_36879235_+9660-Tnks2 | chr19 | Tnks2 | 0.202796353 | 0.182309179 | 0.361292045 | 9661 | 923 |
| chr19_36926401_36411956_+514445-Tnks2 | chr19 | Tnks2 | 0 | 0 | 0.180646022 | 514446 | 514446 |
| chr19_36970038_36956371_+13667-Btaf1 | chr19 | Btaf1 | 0 | 0 | 0.361292045 | 13668 | 1129 |
| chr19_36970038_36958373_+11665-Btaf1 | chr19 | Btaf1 | 0.608389058 | 0.729236718 | 0.722584089 | 11666 | 965 |
| chr19_36973018_36956371_+16647-Btaf1 | chr19 | Btaf1 | 0 | 0 | 0.541938067 | 16648 | 1250 |
| chr19_36983652_36956371_+27281-Btaf1 | chr19 | Btaf1 | 0 | 0 | 0.180646022 | 27282 | 2027 |
| chr19_36983652_36958373_+25279-Btaf1 | chr19 | Btaf1 | 0 | 0.182309179 | 0 | 25280 | 1863 |
| chr19_36994966_36951034_+43932-Btaf1 | chr19 | Btaf1 | 0 | 0.182309179 | 0 | 43933 | 3847 |
| chr19_37004643_36994270_+10373-Btaf1 | chr19 | Btaf1 | 0 | 0.364618359 | 0 | 10374 | 1415 |
| chr19_37054374_37044522_-9852-Cpeb3 | chr19 | Cpeb3 | 0.202796353 | 0 | 0 | 9853 | 297 |
| chr19_37068291_37044522_-23769-Cpeb3 | chr19 | Cpeb3 | 1.216778117 | 0.546927538 | 0.361292045 | 23770 | 416 |
| chr19_37139551_37087538_-52013-Cpeb3 | chr19 | Cpeb3 | 0 | 0.182309179 | 0 | 52014 | 52014 |
| chr19_37174985_37126332_-48653-Cpeb3 | chr19 | Cpeb3 | 0 | 0.364618359 | 0 | 48654 | 1236 |
| chr19_37330612_37207295_-123317-Ide | chr19 | Ide | 0 | 0 | 0.361292045 | 123318 | 123318 |
| chr19_37330612_37325148_-5464-Ide | chr19 | Ide | 0.811185411 | 1.640782614 | 1.264522156 | 5465 | 563 |
| chr19_37330612_37328981_-1631-Ide | chr19 | Ide | 0.405592706 | 0 | 0 | 1632 | 393 |
| chr19_3805053_3793068_+11985-Suv420h1 | chr19 | Suv420h1 | 0.405592706 | 0 | 0 | 11986 | 817 |
| chr19_3807421_3793068_+14353-Suv420h1 | chr19 | Suv420h1 | 0 | 0.182309179 | 0.541938067 | 14354 | 1014 |
| chr19_38525452_38523915_+1537-Plce1 | chr19 | Plce1 | 0.405592706 | 0.364618359 | 0.180646022 | 1538 | 1538 |
| chr19_38799390_38798898_-492-Noc3l | chr19 | Noc3l | 0 | 0.364618359 | 0 | 493 | 182 |
| chr19_40324832_40321793_-3039-Sorbs1 | chr19 | Sorbs1 | 0 | 0.364618359 | 0 | 3040 | 254 |
| chr19_40337035_40311669_-25366-Sorbs1 | chr19 | Sorbs1 | 0 | 0 | 0.361292045 | 25367 | 2427 |
| chr19_40365166_40336986_-28180-na | chr19 | na | 0 | 0 | 0.090323011 | 28181 | 28181 |
| chr19_40377016_40340685_-36331-na | chr19 | na | 0 | 0 | 0.090323011 | 36332 | 36332 |
| chr19_40377016_40340685_-36331-Sorbs1 | chr19 | Sorbs1 | 0 | 0.182309179 | 0 | 36332 | 1362 |
| chr19_41586716_41581778_+4938-na | chr19 | na | 0.405592706 | 0 | 0 | 4939 | 4939 |
| chr19_42564341_42562552_+1789-R3hcc1l | chr19 | R3hcc1l | 1.41957447 | 1.093855076 | 0 | 1790 | 1790 |
| chr19_42576201_42562552_+13649-R3hcc1l | chr19 | R3hcc1l | 0.608389058 | 2.005400973 | 1.083876134 | 13650 | 1966 |
| chr19_42583543_42562552_+20991-R3hcc1l | chr19 | R3hcc1l | 0.608389058 | 0.182309179 | 0 | 20992 | 2274 |
| chr19_4290871_4288412_-2459-Adrbk1 | chr19 | Adrbk1 | 0.405592706 | 0 | 0 | 2460 | 844 |
| chr19_4325046_4320152_-4894-Kdm2a | chr19 | Kdm2a | 0 | 0 | 0.722584089 | 4895 | 1249 |
| chr19_4362914_4342847_-20067-Kdm2a | chr19 | Kdm2a | 0 | 0 | 0.180646022 | 20068 | 1437 |
| chr19_43705030_43704199_+831-Entpd7 | chr19 | Entpd7 | 0 | 0 | 0.180646022 | 832 | 357 |
| chr19_43875020_43852224_-22796-Dnmbp | chr19 | Dnmbp | 0.202796353 | 0 | 0 | 22797 | 1749 |
| chr19_43875020_43866589_-8431-Dnmbp | chr19 | Dnmbp | 1.41957447 | 1.276164256 | 0.180646022 | 8432 | 908 |
| chr19_43903058_43901070_-1988-Dnmbp | chr19 | Dnmbp | 0.202796353 | 0 | 0 | 1989 | 1989 |
| chr19_43910847_43901070_-9777-Dnmbp | chr19 | Dnmbp | 0.608389058 | 0.182309179 | 0.361292045 | 9778 | 2112 |
| chr19_4394892_4342847_-52045-Kdm2a | chr19 | Kdm2a | 0.608389058 | 0 | 0.180646022 | 52046 | 1538 |
| chr19_4394892_4361538_-33354-Kdm2a | chr19 | Kdm2a | 0 | 0.182309179 | 0 | 33355 | 366 |
| chr19_44532693_44528666_-4027-Sec31b | chr19 | Sec31b | 0.202796353 | 0 | 0 | 4028 | 583 |
| chr19_44943519_44934112_+9407-Fam178a | chr19 | Fam178a | 0 | 0 | 0.180646022 | 9408 | 2385 |
| chr19_44949139_44934112_+15027-Fam178a | chr19 | Fam178a | 0 | 0 | 0.180646022 | 15028 | 2676 |
| chr19_4530446_4530249_-197-na | chr19 | na | 0 | 0 | 0.180646022 | 198 | 198 |
| chr19_45532062_45527264_+4798-Btrc | chr19 | Btrc | 0 | 0 | 0.180646022 | 4799 | 3283 |
| chr19_45640521_45626516_-14005-Fbxw4 | chr19 | Fbxw4 | 0.405592706 | 0 | 0 | 14006 | 14006 |
| chr19_45768007_45757905_-10102-Mgea5 | chr19 | Mgea5 | 0 | 0.364618359 | 0.722584089 | 10103 | 1259 |
| chr19_45777017_45770051_-6966-Mgea5 | chr19 | Mgea5 | 0.202796353 | 0 | 0 | 6967 | 846 |
| chr19_45846892_45846108_-784-9130011E15Rik | chr19 | 9130011E15Rik | 0.202796353 | 0 | 0 | 785 | 785 |
| chr19_45946103_45921617_-24486-9130011E15Rik | chr19 | 9130011E15Rik | 0 | 0.182309179 | 0 | 24487 | 447 |
| chr19_45978712_45972823_-5889-9130011E15Rik | chr19 | 9130011E15Rik | 0 | 0.182309179 | 0 | 5890 | 425 |
| chr19_46280731_46279210_+1521-Gbf1 | chr19 | Gbf1 | 0.202796353 | 0 | 0 | 1522 | 828 |
| chr19_47075128_47070981_+4147-Taf5 | chr19 | Taf5 | 0 | 0 | 0.180646022 | 4148 | 554 |
| chr19_47343468_47314035_-29433-na | chr19 | na | 0.101398176 | 0 | 0 | 29434 | 29434 |
| chr19_4785567_4783838_-1729-Rbm4 | chr19 | Rbm4 | 0 | 0.182309179 | 0 | 1730 | 1730 |
| chr19_4785567_4784724_-843-na | chr19 | na | 1.622370823 | 0.364618359 | 0.541938067 | 844 | 844 |
| chr19_4794009_4776967_-17042-na | chr19 | na | 0 | 0.09115459 | 0 | 17043 | 17043 |
| chr19_4794009_4790157_-3852-Rbm4 | chr19 | Rbm4 | 0.405592706 | 0 | 0 | 3853 | 3853 |
| chr19_4804015_4787361_-16654-Rbm4 | chr19 | Rbm4 | 0 | 0 | 0.541938067 | 16655 | 16655 |
| chr19_4870993_4869358_-1635-Actn3 | chr19 | Actn3 | 0 | 0.364618359 | 0.361292045 | 1636 | 168 |
| chr19_4871160_4868763_-2397-na | chr19 | na | 0 | 0.09115459 | 0 | 2398 | 2398 |
| chr19_5288413_5279269_-9144-Sf3b2 | chr19 | Sf3b2 | 0 | 0.182309179 | 0 | 9145 | 1663 |
| chr19_53818020_53813248_+4772-Rbm20 | chr19 | Rbm20 | 1.216778117 | 0.182309179 | 0.722584089 | 4773 | 1345 |
| chr19_53834779_53813248_+21531-Rbm20 | chr19 | Rbm20 | 0.405592706 | 0.364618359 | 0 | 21532 | 1621 |
| chr19_53853635_53850102_+3533-Rbm20 | chr19 | Rbm20 | 0.811185411 | 0.911545897 | 1.625814201 | 3534 | 859 |
| chr19_5418251_5417394_+857-4930481A15Rik | chr19 | 4930481A15Rik | 0 | 0.273463769 | 0 | 858 | 858 |
| chr19_5418251_5417394_+857-na | chr19 | na | 0.101398176 | 0 | 0 | 858 | 858 |
| chr19_55391868_55380942_+10926-Vti1a | chr19 | Vti1a | 0.202796353 | 0.546927538 | 0 | 10927 | 184 |
| chr19_56355586_56350289_-5297-Nrap | chr19 | Nrap | 0 | 0 | 0.180646022 | 5298 | 630 |
| chr19_56378171_56378055_-116-Nrap | chr19 | Nrap | 0 | 0.182309179 | 0 | 117 | 117 |
| chr19_56380313_56380206_-107-Nrap | chr19 | Nrap | 0 | 0.364618359 | 0 | 108 | 108 |
| chr19_56792160_56790919_-1241-Ccdc186 | chr19 | Ccdc186 | 0 | 0 | 0.361292045 | 1242 | 348 |
| chr19_57173459_57152273_-21186-Ablim1 | chr19 | Ablim1 | 0 | 0.182309179 | 0 | 21187 | 429 |
| chr19_57173459_57155152_-18307-Ablim1 | chr19 | Ablim1 | 0.202796353 | 0 | 0 | 18308 | 319 |
| chr19_57755618_57751630_+3988-Atrnl1 | chr19 | Atrnl1 | 0.202796353 | 1.093855076 | 0.722584089 | 3989 | 332 |
| chr19_57777945_57691767_+86178-Atrnl1 | chr19 | Atrnl1 | 0 | 0.182309179 | 0 | 86179 | 1616 |
| chr19_57777945_57751630_+26315-na | chr19 | na | 0 | 0 | 0.090323011 | 26316 | 26316 |
| chr19_5798896_4288412_-1510484-Pcnx3 | chr19 | Pcnx3 | 0 | 0.182309179 | 0 | 1510485 | 1510485 |
| chr19_5798896_5798036_-860-na | chr19 | na | 0.202796353 | 0.182309179 | 0 | 861 | 861 |
| chr19_5839222_5836706_-2516-na | chr19 | na | 0.202796353 | 0 | 0 | 2517 | 2517 |
| chr19_5840226_5839972_-254-na | chr19 | na | 0 | 0.364618359 | 0 | 255 | 255 |
| chr19_59913448_59871373_-42075-Rab11fip2 | chr19 | Rab11fip2 | 0 | 0 | 0.361292045 | 42076 | 42076 |
| chr19_59913448_59896985_-16463-Rab11fip2 | chr19 | Rab11fip2 | 0.202796353 | 0 | 0 | 16464 | 364 |
| chr19_59937430_59896985_-40445-Rab11fip2 | chr19 | Rab11fip2 | 0 | 0 | 0.180646022 | 40446 | 1276 |
| chr19_7458423_7456300_-2123-Rtn3 | chr19 | Rtn3 | 0.811185411 | 1.093855076 | 0.180646022 | 2124 | 2124 |
| chr19_7474798_7456300_-18498-Rtn3 | chr19 | Rtn3 | 0.202796353 | 0 | 0 | 18499 | 18499 |
| chr19_7518596_7509348_+9248-Atl3 | chr19 | Atl3 | 0 | 0 | 0.180646022 | 9249 | 515 |
| chr19_7529361_7520787_+8574-Atl3 | chr19 | Atl3 | 0.608389058 | 0.911545897 | 0 | 8575 | 8575 |
| chr19_8829950_8825972_+3978-Hnrnpul2 | chr19 | Hnrnpul2 | 0 | 0.182309179 | 0 | 3979 | 499 |
| chr19_8931503_8931047_+456-Eml3 | chr19 | Eml3 | 0 | 0.364618359 | 0 | 457 | 372 |
| chr19_9102719_9076576_+26143-Gm41805 | chr19 | Gm41805 | 0.405592706 | 0 | 0 | 26144 | 26144 |
| chr2_10064311_10058996_-5315-Atp5c1 | chr2 | Atp5c1 | 0.202796353 | 0 | 0 | 5316 | 667 |
| chr2_101901400_101895135_+6265-Commd9 | chr2 | Commd9 | 0 | 0 | 0.180646022 | 6266 | 878 |
| chr2_103776157_103775417_-740-Caprin1 | chr2 | Caprin1 | 0 | 0.182309179 | 0 | 741 | 405 |
| chr2_104435057_104433168_-1889-Hipk3 | chr2 | Hipk3 | 0.405592706 | 0.364618359 | 0.903230112 | 1890 | 844 |
| chr2_104441364_104433168_-8196-Hipk3 | chr2 | Hipk3 | 0.202796353 | 0 | 0 | 8197 | 1517 |
| chr2_104446635_104430727_-15908-Hipk3 | chr2 | Hipk3 | 0 | 0.182309179 | 0 | 15909 | 2005 |
| chr2_104778253_104776172_-2081-Qser1 | chr2 | Qser1 | 0 | 0 | 0.722584089 | 2082 | 469 |
| chr2_104780260_104760494_-19766-Qser1 | chr2 | Qser1 | 0 | 0 | 0.180646022 | 19767 | 1280 |
| chr2_104780260_104770043_-10217-Qser1 | chr2 | Qser1 | 0.202796353 | 0 | 0 | 10218 | 933 |
| chr2_104780260_104776172_-4088-Qser1 | chr2 | Qser1 | 0 | 0.182309179 | 0.180646022 | 4089 | 795 |
| chr2_104790197_104786520_-3677-Qser1 | chr2 | Qser1 | 0 | 0 | 0.541938067 | 3678 | 3678 |
| chr2_104795294_104776172_-19122-Qser1 | chr2 | Qser1 | 0.202796353 | 0 | 0 | 19123 | 4580 |
| chr2_104795294_104786520_-8774-Qser1 | chr2 | Qser1 | 0.202796353 | 0 | 0 | 8775 | 3785 |
| chr2_104830684_104826176_+4508-na | chr2 | na | 0 | 0.364618359 | 0 | 4509 | 4509 |
| chr2_105981120_105966708_-14412-Dnajc24 | chr2 | Dnajc24 | 0.202796353 | 0 | 0 | 14413 | 595 |
| chr2_109137532_109131485_-6047-Mettl15 | chr2 | Mettl15 | 0 | 0 | 0.180646022 | 6048 | 371 |
| chr2_112355144_112354174_+970-Slc12a6 | chr2 | Slc12a6 | 0 | 0 | 0.180646022 | 971 | 366 |
| chr2_11251996_11250529_-1467-na | chr2 | na | 0 | 0.182309179 | 0.180646022 | 1468 | 1468 |
| chr2_112912403_112900800_-11603-Ryr3 | chr2 | Ryr3 | 0.202796353 | 0 | 0 | 11604 | 941 |
| chr2_114058656_114050289_-8367-Actc1 | chr2 | Actc1 | 35.89495445 | 7.292367175 | 11.74199145 | 8368 | 8368 |
| chr2_114151583_114132967_-18616-Aqr | chr2 | Aqr | 0.608389058 | 0 | 0 | 18617 | 1283 |
| chr2_114151583_114132967_-18616-na | chr2 | na | 0 | 0.09115459 | 0 | 18617 | 18617 |
| chr2_11490356_11489671_-685-Pfkfb3 | chr2 | Pfkfb3 | 0 | 0 | 0.361292045 | 686 | 142 |
| chr2_11494028_11474393_-19635-Pfkfb3 | chr2 | Pfkfb3 | 0 | 0 | 0.180646022 | 19636 | 1753 |
| chr2_11730646_11723413_+7233-Il15ra | chr2 | Il15ra | 0.304194529 | 0 | 0 | 7234 | 511 |
| chr2_118420872_118408352_+12520-Eif2ak4 | chr2 | Eif2ak4 | 0 | 0.182309179 | 0 | 12521 | 654 |
| chr2_118432280_118412935_+19345-Eif2ak4 | chr2 | Eif2ak4 | 0 | 0.546927538 | 0 | 19346 | 1719 |
| chr2_118448512_118442015_+6497-Eif2ak4 | chr2 | Eif2ak4 | 0 | 0 | 0.180646022 | 6498 | 473 |
| chr2_118877947_118877661_+286-Ivd | chr2 | Ivd | 0.405592706 | 0 | 0 | 287 | 178 |
| chr2_118921696_118915888_+5808-Bahd1 | chr2 | Bahd1 | 0 | 0 | 0.180646022 | 5809 | 2533 |
| chr2_119412627_119402330_-10297-Ino80 | chr2 | Ino80 | 0 | 0.364618359 | 0 | 10298 | 449 |
| chr2_119445656_119434149_-11507-Ino80 | chr2 | Ino80 | 0.202796353 | 0 | 0 | 11508 | 711 |
| chr2_119447059_119402330_-44729-Ino80 | chr2 | Ino80 | 0.202796353 | 0.182309179 | 0 | 44730 | 2573 |
| chr2_119447059_119425259_-21800-Ino80 | chr2 | Ino80 | 0 | 0 | 0.361292045 | 21801 | 1811 |
| chr2_119447059_119433191_-13868-Ino80 | chr2 | Ino80 | 0 | 0 | 0.361292045 | 13869 | 1058 |
| chr2_119451450_119402330_-49120-Ino80 | chr2 | Ino80 | 0.811185411 | 0.364618359 | 0.541938067 | 49121 | 2963 |
| chr2_119451450_119412402_-39048-Ino80 | chr2 | Ino80 | 0 | 0.182309179 | 0 | 39049 | 2740 |
| chr2_119903733_119902606_+1127-Mga | chr2 | Mga | 0 | 0 | 1.445168179 | 1128 | 1128 |
| chr2_119924086_119916431_+7655-Mga | chr2 | Mga | 0 | 0.729236718 | 0 | 7656 | 2014 |
| chr2_120185349_120185000_-349-Pla2g4e | chr2 | Pla2g4e | 0.101398176 | 0 | 0.361292045 | 350 | 248 |
| chr2_120209923_120200213_-9710-Pla2g4e | chr2 | Pla2g4e | 0 | 0.182309179 | 0 | 9711 | 290 |
| chr2_120328715_120328573_-142-Vps39 | chr2 | Vps39 | 0 | 0.182309179 | 0 | 143 | 143 |
| chr2_120394475_120377893_-16582-Tmem87a | chr2 | Tmem87a | 0.608389058 | 0 | 0 | 16583 | 828 |
| chr2_120397447_120377893_-19554-Tmem87a | chr2 | Tmem87a | 0 | 0 | 0.180646022 | 19555 | 914 |
| chr2_120397447_120385774_-11673-Tmem87a | chr2 | Tmem87a | 0 | 0.182309179 | 0 | 11674 | 568 |
| chr2_120402770_120371524_-31246-Tmem87a | chr2 | Tmem87a | 0 | 0 | 0.180646022 | 31247 | 1250 |
| chr2_120532222_120522685_-9537-Zfp106 | chr2 | Zfp106 | 0 | 0 | 0.180646022 | 9538 | 2209 |
| chr2_120790355_120760197_-30158-Ttbk2 | chr2 | Ttbk2 | 0 | 0 | 0.361292045 | 30159 | 763 |
| chr2_120825826_120773229_-52597-Ttbk2 | chr2 | Ttbk2 | 0.202796353 | 0 | 0 | 52598 | 52598 |
| chr2_121343577_121321655_-21922-Ppip5k1 | chr2 | Ppip5k1 | 0 | 0.182309179 | 0 | 21923 | 3098 |
| chr2_121347642_121347264_-378-Ppip5k1 | chr2 | Ppip5k1 | 0 | 0.182309179 | 0.180646022 | 379 | 284 |
| chr2_121351013_121331200_-19813-Ppip5k1 | chr2 | Ppip5k1 | 0 | 0.182309179 | 0 | 19814 | 3379 |
| chr2_122689552_122682321_-7231-Slc30a4 | chr2 | Slc30a4 | 0 | 0.182309179 | 0 | 7232 | 3613 |
| chr2_12397566_12382103_-15463-Fam188a | chr2 | Fam188a | 0 | 0 | 0.361292045 | 15464 | 306 |
| chr2_12405920_12396179_-9741-Fam188a | chr2 | Fam188a | 0 | 0 | 0.180646022 | 9742 | 579 |
| chr2_125775731_125771409_-4322-Secisbp2l | chr2 | Secisbp2l | 0.202796353 | 0 | 0.361292045 | 4323 | 640 |
| chr2_125836239_125833724_-2515-Cops2 | chr2 | Cops2 | 0 | 0.182309179 | 0 | 2516 | 240 |
| chr2_125983363_125975260_+8103-Galk2 | chr2 | Galk2 | 0 | 0.182309179 | 0 | 8104 | 511 |
| chr2_126799234_126797111_-2123-Trpm7 | chr2 | Trpm7 | 0 | 0 | 0.180646022 | 2124 | 639 |
| chr2_126799234_126797662_-1572-Trpm7 | chr2 | Trpm7 | 0 | 0.182309179 | 0 | 1573 | 350 |
| chr2_126830263_126812696_-17567-Trpm7 | chr2 | Trpm7 | 0 | 0.182309179 | 0 | 17568 | 2686 |
| chr2_126830263_126825412_-4851-Trpm7 | chr2 | Trpm7 | 0 | 0 | 0.361292045 | 4852 | 945 |
| chr2_126846245_126844300_-1945-Trpm7 | chr2 | Trpm7 | 0.202796353 | 0 | 0 | 1946 | 347 |
| chr2_127222254_127212349_+9905-Snrnp200 | chr2 | Snrnp200 | 0 | 0 | 0.180646022 | 9906 | 1586 |
| chr2_127284581_127284095_+486-Stard7 | chr2 | Stard7 | 0 | 0 | 0.180646022 | 487 | 487 |
| chr2_127295568_127284095_+11473-Stard7 | chr2 | Stard7 | 0 | 0.729236718 | 0 | 11474 | 866 |
| chr2_127297256_127290772_+6484-Stard7 | chr2 | Stard7 | 0.202796353 | 0 | 0 | 6485 | 529 |
| chr2_128676247_128675671_-576-Anapc1 | chr2 | Anapc1 | 0 | 0 | 0.361292045 | 577 | 367 |
| chr2_128680445_128675671_-4774-Anapc1 | chr2 | Anapc1 | 0 | 0 | 0.180646022 | 4775 | 677 |
| chr2_128686060_128680109_-5951-Anapc1 | chr2 | Anapc1 | 0 | 0.182309179 | 0 | 5952 | 552 |
| chr2_128784010_128771347_+12663-Mertk | chr2 | Mertk | 0.202796353 | 0 | 0 | 12664 | 783 |
| chr2_128794578_128771347_+23231-Mertk | chr2 | Mertk | 0.202796353 | 0 | 0 | 23232 | 1190 |
| chr2_130504335_130503474_+861-Ptpra | chr2 | Ptpra | 0 | 0 | 0.361292045 | 862 | 421 |
| chr2_130713503_130712260_-1243-4930402H24Rik | chr2 | 4930402H24Rik | 0 | 0.182309179 | 0 | 1244 | 385 |
| chr2_131274263_131271559_+2704-Pank2 | chr2 | Pank2 | 1.013981764 | 0.546927538 | 0 | 2705 | 446 |
| chr2_131287597_131271559_+16038-Pank2 | chr2 | Pank2 | 0 | 0 | 0.180646022 | 16039 | 1001 |
| chr2_131287597_131273911_+13686-Pank2 | chr2 | Pank2 | 0.202796353 | 0.364618359 | 0 | 13687 | 908 |
| chr2_131525073_131511984_+13089-Smox | chr2 | Smox | 0 | 0 | 0.180646022 | 13090 | 1996 |
| chr2_132101726_132089096_-12630-Slc23a2 | chr2 | Slc23a2 | 0 | 0 | 0.361292045 | 12631 | 629 |
| chr2_132530090_132529869_-221-na | chr2 | na | 0.202796353 | 0 | 0 | 222 | 222 |
| chr2_132558682_132544392_-14290-Gpcpd1 | chr2 | Gpcpd1 | 1.013981764 | 0 | 0.361292045 | 14291 | 834 |
| chr2_132558682_132546841_-11841-Gpcpd1 | chr2 | Gpcpd1 | 0 | 0.364618359 | 0.361292045 | 11842 | 779 |
| chr2_132568716_132550314_-18402-Gpcpd1 | chr2 | Gpcpd1 | 0 | 0 | 0.361292045 | 18403 | 665 |
| chr2_140048309_140030601_-17708-Tasp1 | chr2 | Tasp1 | 0 | 0.364618359 | 0 | 17709 | 400 |
| chr2_140625817_140596066_+29751-na | chr2 | na | 0 | 0 | 0.180646022 | 29752 | 29752 |
| chr2_142690701_142648428_-42273-Kif16b | chr2 | Kif16b | 0 | 0.364618359 | 0 | 42274 | 297 |
| chr2_14329924_14276287_+53637-Mrc1 | chr2 | Mrc1 | 0.405592706 | 0 | 0 | 53638 | 2599 |
| chr2_144394378_144380707_+13671-Csrp2bp | chr2 | Csrp2bp | 0 | 0.182309179 | 0 | 13672 | 1284 |
| chr2_146361519_146314434_-47085-Ralgapa2 | chr2 | Ralgapa2 | 0.202796353 | 0 | 0 | 47086 | 47086 |
| chr2_146412868_146380249_-32619-Ralgapa2 | chr2 | Ralgapa2 | 0 | 0 | 0.090323011 | 32620 | 32620 |
| chr2_146412868_146387228_-25640-Ralgapa2 | chr2 | Ralgapa2 | 0 | 0.364618359 | 0.541938067 | 25641 | 1064 |
| chr2_146412868_146404863_-8005-Ralgapa2 | chr2 | Ralgapa2 | 0 | 0.364618359 | 0 | 8006 | 615 |
| chr2_146895293_146889021_+6272-Kiz | chr2 | Kiz | 0.506990882 | 0.364618359 | 0 | 6273 | 6273 |
| chr2_146942979_146935803_+7176-Kiz | chr2 | Kiz | 1.013981764 | 0 | 0 | 7177 | 311 |
| chr2_147026439_147024715_+1724-Xrn2 | chr2 | Xrn2 | 0 | 0.364618359 | 0 | 1725 | 283 |
| chr2_148690983_148686514_+4469-Gzf1 | chr2 | Gzf1 | 0.202796353 | 0.729236718 | 0 | 4470 | 698 |
| chr2_148690983_148688004_+2979-Gzf1 | chr2 | Gzf1 | 0.202796353 | 0 | 0 | 2980 | 603 |
| chr2_148690983_148690076_+907-Gzf1 | chr2 | Gzf1 | 0.202796353 | 0 | 0 | 908 | 435 |
| chr2_148691678_148686514_+5164-Gzf1 | chr2 | Gzf1 | 0 | 0.364618359 | 0 | 5165 | 1393 |
| chr2_148707023_148703061_-3962-Napb | chr2 | Napb | 0 | 0 | 1.625814201 | 3963 | 219 |
| chr2_152917666_152913445_+4221-Mylk2 | chr2 | Mylk2 | 0 | 0.182309179 | 0 | 4222 | 748 |
| chr2_152919453_152913445_+6008-Mylk2 | chr2 | Mylk2 | 5.881094232 | 5.469275382 | 6.322610782 | 6009 | 948 |
| chr2_153204624_153200643_+3981-Tm9sf4 | chr2 | Tm9sf4 | 0 | 0 | 0.361292045 | 3982 | 450 |
| chr2_154360767_154345866_-14901-Cdk5rap1 | chr2 | Cdk5rap1 | 0 | 0 | 0.361292045 | 14902 | 998 |
| chr2_154863663_154861977_+1686-Raly | chr2 | Raly | 0 | 0 | 0.090323011 | 1687 | 144 |
| chr2_155185604_155179042_+6562-Itch | chr2 | Itch | 0 | 0 | 0.180646022 | 6563 | 671 |
| chr2_155192210_155182158_+10052-Itch | chr2 | Itch | 0.202796353 | 0.182309179 | 0 | 10053 | 622 |
| chr2_155213102_155209059_+4043-Itch | chr2 | Itch | 0 | 0.182309179 | 0 | 4044 | 661 |
| chr2_155331258_155292690_-38568-Pigu | chr2 | Pigu | 0 | 0 | 0.180646022 | 38569 | 876 |
| chr2_155335423_155314502_-20921-Pigu | chr2 | Pigu | 0 | 0 | 0.361292045 | 20922 | 372 |
| chr2_155402740_155395720_-7020-Ncoa6 | chr2 | Ncoa6 | 0.202796353 | 0 | 0 | 7021 | 258 |
| chr2_155911836_155887109_-24727-Uqcc1 | chr2 | Uqcc1 | 0 | 0 | 0.180646022 | 24728 | 447 |
| chr2_156103397_156096418_-6979-na | chr2 | na | 0 | 0.09115459 | 0 | 6980 | 6980 |
| chr2_156103429_156096826_-6603-Rbm12 | chr2 | Rbm12 | 0.202796353 | 0 | 0 | 6604 | 1628 |
| chr2_156267542_156264281_+3261-Phf20 | chr2 | Phf20 | 0 | 0.364618359 | 0 | 3262 | 468 |
| chr2_156514210_156508813_+5397-Epb41l1 | chr2 | Epb41l1 | 0.405592706 | 0 | 0 | 5398 | 642 |
| chr2_158050213_158035996_+14217-Rprd1b | chr2 | Rprd1b | 0 | 0.364618359 | 0.180646022 | 14218 | 504 |
| chr2_158058827_158047919_+10908-Rprd1b | chr2 | Rprd1b | 0.202796353 | 0 | 0 | 10909 | 413 |
| chr2_158482501_158439529_+42972-Ralgapb | chr2 | Ralgapb | 0 | 0 | 0.180646022 | 42973 | 2600 |
| chr2_158493013_158492364_+649-Ralgapb | chr2 | Ralgapb | 0.101398176 | 0 | 0.090323011 | 650 | 188 |
| chr2_158636800_158631463_+5337-Actr5 | chr2 | Actr5 | 0.202796353 | 0 | 0 | 5338 | 794 |
| chr2_158832822_158826305_+6517-Dhx35 | chr2 | Dhx35 | 0.202796353 | 0.364618359 | 0 | 6518 | 1243 |
| chr2_160705321_160670100_+35221-Top1 | chr2 | Top1 | 0 | 0.182309179 | 0.361292045 | 35222 | 1256 |
| chr2_163376375_163375604_-771-Jph2 | chr2 | Jph2 | 1.825167175 | 0.182309179 | 0.180646022 | 772 | 772 |
| chr2_163637004_163629127_-7877-Serinc3 | chr2 | Serinc3 | 0 | 0 | 0.180646022 | 7878 | 673 |
| chr2_163639327_163629127_-10200-Serinc3 | chr2 | Serinc3 | 0 | 0 | 0.180646022 | 10201 | 835 |
| chr2_164098576_164083630_+14946-Stk4 | chr2 | Stk4 | 0.202796353 | 0 | 0 | 14947 | 715 |
| chr2_164778227_164759229_-18998-Tnnc2 | chr2 | Tnnc2 | 0 | 0.182309179 | 0 | 18999 | 18999 |
| chr2_16548263_16512022_+36241-Plxdc2 | chr2 | Plxdc2 | 0.202796353 | 0 | 0 | 36242 | 359 |
| chr2_16565405_16512022_+53383-Plxdc2 | chr2 | Plxdc2 | 0 | 0.364618359 | 0 | 53384 | 429 |
| chr2_165767158_165761904_+5254-Eya2 | chr2 | Eya2 | 0 | 0.364618359 | 0 | 5255 | 457 |
| chr2_166068548_166054520_+14028-Ncoa3 | chr2 | Ncoa3 | 0.202796353 | 0 | 0 | 14029 | 2542 |
| chr2_16660873_16512022_+148851-Plxdc2 | chr2 | Plxdc2 | 0.202796353 | 0 | 0 | 148852 | 771 |
| chr2_166836457_166834471_+1986-Arfgef2 | chr2 | Arfgef2 | 0.608389058 | 0.911545897 | 0.361292045 | 1987 | 583 |
| chr2_166836457_166835613_+844-Arfgef2 | chr2 | Arfgef2 | 0 | 0 | 0.541938067 | 845 | 436 |
| chr2_166869043_166865680_+3363-Arfgef2 | chr2 | Arfgef2 | 0 | 0.182309179 | 0 | 3364 | 611 |
| chr2_166893618_166885779_+7839-Arfgef2 | chr2 | Arfgef2 | 0.405592706 | 0.182309179 | 0 | 7840 | 557 |
| chr2_167309336_167307544_-1792-B4galt5 | chr2 | B4galt5 | 0 | 0 | 0.722584089 | 1793 | 239 |
| chr2_167471602_167467685_+3917-Slc9a8 | chr2 | Slc9a8 | 0.202796353 | 0 | 0 | 3918 | 416 |
| chr2_167511326_167507047_+4279-Rnf114 | chr2 | Rnf114 | 0.811185411 | 0.182309179 | 0.180646022 | 4280 | 373 |
| chr2_168223154_168212960_-10194-Dpm1-adnp | chr2 | Dpm1-adnp | 0 | 0.182309179 | 0 | 10195 | 302 |
| chr2_168507370_168504445_-2925-Nfatc2 | chr2 | Nfatc2 | 2.433556234 | 1.823091794 | 1.264522156 | 2926 | 873 |
| chr2_168571113_168567139_-3974-Nfatc2 | chr2 | Nfatc2 | 0.608389058 | 0.182309179 | 0 | 3975 | 688 |
| chr2_168710976_168668006_-42970-Atp9a | chr2 | Atp9a | 0 | 0.546927538 | 0 | 42971 | 1438 |
| chr2_169886459_169883526_+2933-Tshz2 | chr2 | Tshz2 | 0 | 0 | 0.180646022 | 2934 | 2934 |
| chr2_170120753_170114061_-6692-Zfp217 | chr2 | Zfp217 | 0 | 0.182309179 | 0 | 6693 | 3364 |
| chr2_174426946_174426489_+457-Nelfcd | chr2 | Nelfcd | 0.405592706 | 0 | 0 | 458 | 271 |
| chr2_179921967_179921152_-815-Taf4 | chr2 | Taf4 | 0.608389058 | 0 | 0 | 816 | 177 |
| chr2_179923079_179921152_-1927-Taf4 | chr2 | Taf4 | 0 | 0.182309179 | 0 | 1928 | 303 |
| chr2_179936011_179921152_-14859-Taf4 | chr2 | Taf4 | 0 | 0 | 0.180646022 | 14860 | 1446 |
| chr2_179938530_179935029_-3501-Taf4 | chr2 | Taf4 | 0 | 0.182309179 | 0 | 3502 | 363 |
| chr2_179939954_179921152_-18802-Taf4 | chr2 | Taf4 | 0.405592706 | 0 | 0 | 18803 | 1727 |
| chr2_179939954_179931886_-8068-Taf4 | chr2 | Taf4 | 0.405592706 | 0 | 0 | 8069 | 8069 |
| chr2_179939954_179935029_-4925-Taf4 | chr2 | Taf4 | 0.608389058 | 0.546927538 | 0 | 4926 | 524 |
| chr2_180038849_180037383_-1466-Psma7 | chr2 | Psma7 | 4.258723409 | 1.276164256 | 0.903230112 | 1467 | 368 |
| chr2_180685176_180673231_-11945-Dido1 | chr2 | Dido1 | 0 | 0 | 0.361292045 | 11946 | 1275 |
| chr2_181155481_181152784_-2697-Eef1a2 | chr2 | Eef1a2 | 0 | 0 | 0.180646022 | 2698 | 696 |
| chr2_18126229_18109782_+16447-Mllt10 | chr2 | Mllt10 | 0 | 0 | 0.180646022 | 16448 | 553 |
| chr2_181352848_181346832_+6016-Rtel1 | chr2 | Rtel1 | 0.202796353 | 0 | 0 | 6017 | 1461 |
| chr2_18146872_18092168_+54704-Mllt10 | chr2 | Mllt10 | 0 | 0 | 0.180646022 | 54705 | 1291 |
| chr2_18146872_18122245_+24627-Mllt10 | chr2 | Mllt10 | 0 | 0.364618359 | 0 | 24628 | 459 |
| chr2_18146872_18132951_+13921-Mllt10 | chr2 | Mllt10 | 0 | 0.182309179 | 0.361292045 | 13922 | 13922 |
| chr2_181547553_181546371_+1182-Dnajc5 | chr2 | Dnajc5 | 0.405592706 | 0.182309179 | 0 | 1183 | 332 |
| chr2_181622317_181615977_+6340-Prpf6 | chr2 | Prpf6 | 0.202796353 | 0 | 0 | 6341 | 531 |
| chr2_18171075_18132951_+38124-Mllt10 | chr2 | Mllt10 | 0 | 0.182309179 | 0 | 38125 | 38125 |
| chr2_18171075_18146777_+24298-Mllt10 | chr2 | Mllt10 | 0 | 0.182309179 | 0 | 24299 | 1000 |
| chr2_18186396_18159456_+26940-Mllt10 | chr2 | Mllt10 | 0 | 0 | 0.361292045 | 26941 | 1083 |
| chr2_18197997_18159456_+38541-Mllt10 | chr2 | Mllt10 | 0 | 0.182309179 | 0 | 38542 | 1274 |
| chr2_18197997_18162749_+35248-Mllt10 | chr2 | Mllt10 | 0 | 0 | 0.180646022 | 35249 | 586 |
| chr2_18203755_18132951_+70804-Mllt10 | chr2 | Mllt10 | 0.202796353 | 0.182309179 | 0.361292045 | 70805 | 70805 |
| chr2_18203755_18186218_+17537-Mllt10 | chr2 | Mllt10 | 0 | 0 | 0.180646022 | 17538 | 525 |
| chr2_18222761_18216375_-6386-Dnajc1 | chr2 | Dnajc1 | 0 | 0.182309179 | 0 | 6387 | 1449 |
| chr2_20766611_20760215_+6396-Etl4 | chr2 | Etl4 | 0 | 0 | 0.180646022 | 6397 | 217 |
| chr2_20859972_20855354_-4618-Arhgap21 | chr2 | Arhgap21 | 0 | 0 | 0.180646022 | 4619 | 503 |
| chr2_20860036_20854935_-5101-Arhgap21 | chr2 | Arhgap21 | 1.41957447 | 5.104657023 | 2.167752268 | 5102 | 673 |
| chr2_20860036_20855354_-4682-Arhgap21 | chr2 | Arhgap21 | 3.447537998 | 5.286966202 | 4.335504536 | 4683 | 567 |
| chr2_20881851_20879961_-1890-Arhgap21 | chr2 | Arhgap21 | 0 | 0 | 0.361292045 | 1891 | 1891 |
| chr2_20889953_20879961_-9992-Arhgap21 | chr2 | Arhgap21 | 0.405592706 | 0.182309179 | 0 | 9993 | 2055 |
| chr2_20892177_20871392_-20785-Arhgap21 | chr2 | Arhgap21 | 0 | 0 | 0.180646022 | 20786 | 2410 |
| chr2_20892177_20887106_-5071-Arhgap21 | chr2 | Arhgap21 | 0.202796353 | 0.364618359 | 0 | 5072 | 227 |
| chr2_22971259_22957018_-14241-Abi1 | chr2 | Abi1 | 0.202796353 | 0 | 0 | 14242 | 535 |
| chr2_23545579_23506909_-38670-Spopl | chr2 | Spopl | 0 | 0 | 0.180646022 | 38671 | 5741 |
| chr2_24815832_24801507_-14325-Ehmt1 | chr2 | Ehmt1 | 0.202796353 | 0 | 0 | 14326 | 1035 |
| chr2_24825067_24801507_-23560-Ehmt1 | chr2 | Ehmt1 | 0.608389058 | 0 | 0.180646022 | 23561 | 1174 |
| chr2_24858250_24836396_-21854-Ehmt1 | chr2 | Ehmt1 | 0 | 0.182309179 | 0 | 21855 | 1212 |
| chr2_24884702_24838701_-46001-Ehmt1 | chr2 | Ehmt1 | 0 | 0 | 0.180646022 | 46002 | 2098 |
| chr2_24884702_24852665_-32037-Ehmt1 | chr2 | Ehmt1 | 0.202796353 | 0 | 0.361292045 | 32038 | 1413 |
| chr2_24887069_24824945_-62124-Ehmt1 | chr2 | Ehmt1 | 0 | 0.182309179 | 0 | 62125 | 2484 |
| chr2_25049743_24997227_+52516-Pnpla7 | chr2 | Pnpla7 | 0 | 0.182309179 | 0 | 52517 | 52517 |
| chr2_25050971_25049999_+972-Pnpla7 | chr2 | Pnpla7 | 0 | 0.364618359 | 0 | 973 | 304 |
| chr2_25206345_25200659_-5686-Nelfb | chr2 | Nelfb | 0 | 0.182309179 | 0 | 5687 | 999 |
| chr2_25206345_25203141_-3204-Nelfb | chr2 | Nelfb | 0 | 0.546927538 | 0.361292045 | 3205 | 748 |
| chr2_25278505_25273800_+4705-Anapc2 | chr2 | Anapc2 | 0.202796353 | 0 | 0 | 4706 | 728 |
| chr2_25345594_25341607_+3987-Man1b1 | chr2 | Man1b1 | 0.202796353 | 0 | 0 | 3988 | 634 |
| chr2_25588712_25586868_-1844-Rabl6 | chr2 | Rabl6 | 0 | 0.182309179 | 0 | 1845 | 608 |
| chr2_25602555_25596433_-6122-Rabl6 | chr2 | Rabl6 | 0 | 0 | 0.180646022 | 6123 | 345 |
| chr2_25941469_25937946_-3523-Camsap1 | chr2 | Camsap1 | 0.608389058 | 0 | 0 | 3524 | 2545 |
| chr2_25966948_25944426_-22522-Camsap1 | chr2 | Camsap1 | 0.405592706 | 0 | 0 | 22523 | 1123 |
| chr2_25966948_25952678_-14270-Camsap1 | chr2 | Camsap1 | 0 | 0 | 0.541938067 | 14271 | 708 |
| chr2_26014965_26007719_-7246-Ubac1 | chr2 | Ubac1 | 0 | 1.093855076 | 0 | 7247 | 629 |
| chr2_27741943_27737299_+4644-Rxra | chr2 | Rxra | 0 | 0 | 0.180646022 | 4645 | 597 |
| chr2_28665162_28658605_+6557-Tsc1 | chr2 | Tsc1 | 0.405592706 | 0 | 0 | 6558 | 743 |
| chr2_29922371_29893001_+29370-Odf2 | chr2 | Odf2 | 0.811185411 | 1.640782614 | 1.264522156 | 29371 | 1945 |
| chr2_29922371_29912569_+9802-Odf2 | chr2 | Odf2 | 1.013981764 | 0.911545897 | 0.361292045 | 9803 | 1068 |
| chr2_29922371_29920775_+1596-Odf2 | chr2 | Odf2 | 0.202796353 | 0 | 0 | 1597 | 375 |
| chr2_30111332_30110205_-1127-Zer1 | chr2 | Zer1 | 0.405592706 | 0.364618359 | 0 | 1128 | 804 |
| chr2_30304401_30298416_+5985-Nup188 | chr2 | Nup188 | 0 | 0 | 0.180646022 | 5986 | 498 |
| chr2_30329413_30315116_+14297-Nup188 | chr2 | Nup188 | 0.202796353 | 0 | 0 | 14298 | 1437 |
| chr2_31097150_31096027_-1123-na | chr2 | na | 0 | 0 | 0.090323011 | 1124 | 1124 |
| chr2_31174239_31167021_+7218-Gpr107 | chr2 | Gpr107 | 0 | 0.182309179 | 0.541938067 | 7219 | 435 |
| chr2_31989139_31988154_+985-Nup214 | chr2 | Nup214 | 0 | 0 | 0.090323011 | 986 | 174 |
| chr2_32034969_32017026_+17943-Nup214 | chr2 | Nup214 | 0 | 0 | 0.180646022 | 17944 | 2646 |
| chr2_32187553_32182458_+5095-Prrc2b | chr2 | Prrc2b | 0.405592706 | 0 | 0 | 5096 | 523 |
| chr2_32194996_32182458_+12538-Prrc2b | chr2 | Prrc2b | 0.202796353 | 0 | 0.361292045 | 12539 | 1171 |
| chr2_32204324_32182458_+21866-Prrc2b | chr2 | Prrc2b | 0 | 0 | 0.180646022 | 21867 | 1771 |
| chr2_32808062_32802754_-5308-Stxbp1 | chr2 | Stxbp1 | 0.202796353 | 0 | 0 | 5309 | 347 |
| chr2_32981883_32980002_-1881-Slc2a8 | chr2 | Slc2a8 | 0 | 0 | 0.180646022 | 1882 | 470 |
| chr2_33834633_33825048_-9585-Mvb12b | chr2 | Mvb12b | 0 | 0.364618359 | 0 | 9586 | 350 |
| chr2_34444406_34432003_+12403-Mapkap1 | chr2 | Mapkap1 | 0 | 0.182309179 | 0 | 12404 | 567 |
| chr2_34695608_34691106_-4502-Gapvd1 | chr2 | Gapvd1 | 0.202796353 | 0 | 0 | 4503 | 363 |
| chr2_34725513_34725043_-470-Gapvd1 | chr2 | Gapvd1 | 0 | 0.364618359 | 0 | 471 | 325 |
| chr2_36240954_36240596_+358-Ptgs1 | chr2 | Ptgs1 | 0.811185411 | 0 | 0 | 359 | 266 |
| chr2_36322923_36322418_+505-na | chr2 | na | 0 | 0 | 0.361292045 | 506 | 506 |
| chr2_37473783_37469377_+4406-Rabgap1 | chr2 | Rabgap1 | 0 | 0.182309179 | 0 | 4407 | 625 |
| chr2_37489833_37469377_+20456-Rabgap1 | chr2 | Rabgap1 | 0 | 0 | 0.180646022 | 20457 | 1239 |
| chr2_37627526_37580673_-46853-Strbp | chr2 | Strbp | 0 | 0.182309179 | 0 | 46854 | 4841 |
| chr2_37647285_37580673_-66612-Strbp | chr2 | Strbp | 0.202796353 | 0 | 0 | 66613 | 5543 |
| chr2_37647285_37624173_-23112-Strbp | chr2 | Strbp | 0.202796353 | 0.182309179 | 0.722584089 | 23113 | 1212 |
| chr2_37647285_37640851_-6434-Strbp | chr2 | Strbp | 0 | 0 | 0.180646022 | 6435 | 557 |
| chr2_37697911_37580673_-117238-Strbp | chr2 | Strbp | 0.202796353 | 0 | 0 | 117239 | 117239 |
| chr2_37844908_37844777_-131-Dennd1a | chr2 | Dennd1a | 0 | 0 | 0.180646022 | 132 | 132 |
| chr2_39024298_39022701_-1597-Golga1 | chr2 | Golga1 | 0.202796353 | 0 | 0 | 1598 | 471 |
| chr2_44828067_44825351_-2716-Gtdc1 | chr2 | Gtdc1 | 0 | 0.182309179 | 0 | 2717 | 2717 |
| chr2_4484132_4431199_+52933-Frmd4a | chr2 | Frmd4a | 0.405592706 | 0 | 0 | 52934 | 254 |
| chr2_48155892_48155316_-576-Gm13481 | chr2 | Gm13481 | 0 | 0.546927538 | 0 | 577 | 577 |
| chr2_48156266_48155316_-950-Gm13481 | chr2 | Gm13481 | 0 | 0.182309179 | 0 | 951 | 951 |
| chr2_48940400_48927096_-13304-Orc4 | chr2 | Orc4 | 0 | 0 | 0.180646022 | 13305 | 596 |
| chr2_49170013_49064705_+105308-Mbd5 | chr2 | Mbd5 | 0 | 0 | 0.180646022 | 105309 | 105309 |
| chr2_49170013_49073671_+96342-na | chr2 | na | 0 | 0 | 0.090323011 | 96343 | 96343 |
| chr2_49170013_49086590_+83423-Mbd5 | chr2 | Mbd5 | 0.202796353 | 0 | 0 | 83424 | 438 |
| chr2_49279783_49272041_+7742-Mbd5 | chr2 | Mbd5 | 0.405592706 | 0 | 0 | 7743 | 2432 |
| chr2_50334658_50332881_+1777-na | chr2 | na | 0 | 0.182309179 | 0 | 1778 | 1778 |
| chr2_5035083_5024067_-11016-Optn | chr2 | Optn | 0.202796353 | 0 | 0 | 11017 | 733 |
| chr2_50370969_50355025_+15944-Gm13483 | chr2 | Gm13483 | 0.405592706 | 0.729236718 | 0.541938067 | 15945 | 15945 |
| chr2_50387728_50355025_+32703-Gm13483 | chr2 | Gm13483 | 0.202796353 | 0 | 0 | 32704 | 32704 |
| chr2_50406520_50355025_+51495-Gm13486 | chr2 | Gm13486 | 0 | 0.364618359 | 0 | 51496 | 51496 |
| chr2_5054716_5045987_-8729-Optn | chr2 | Optn | 0 | 0 | 0.180646022 | 8730 | 593 |
| chr2_52078520_52074247_+4273-Rif1 | chr2 | Rif1 | 0.202796353 | 0 | 0 | 4274 | 610 |
| chr2_52150629_52149160_-1469-Neb | chr2 | Neb | 0 | 0 | 0.361292045 | 1470 | 319 |
| chr2_52151059_52150537_-522-Neb | chr2 | Neb | 0 | 0 | 4.516150558 | 523 | 186 |
| chr2_52157924_52150967_-6957-Neb | chr2 | Neb | 0 | 0 | 0.541938067 | 6958 | 744 |
| chr2_52157924_52151364_-6560-Neb | chr2 | Neb | 0 | 0 | 0.180646022 | 6561 | 651 |
| chr2_52157924_52153056_-4868-Neb | chr2 | Neb | 0 | 0.364618359 | 0.180646022 | 4869 | 558 |
| chr2_52159569_52151364_-8205-Neb | chr2 | Neb | 0.202796353 | 0 | 0.180646022 | 8206 | 744 |
| chr2_52163009_52162905_-104-Neb | chr2 | Neb | 0.405592706 | 0 | 0 | 105 | 105 |
| chr2_52164702_52161371_-3331-Neb | chr2 | Neb | 0 | 0.182309179 | 0 | 3332 | 540 |
| chr2_52165277_52165167_-110-Neb | chr2 | Neb | 0 | 0.364618359 | 0.180646022 | 111 | 111 |
| chr2_52179566_52177522_-2044-Neb | chr2 | Neb | 2.027963528 | 1.276164256 | 1.806460223 | 2045 | 315 |
| chr2_52183836_52167560_-16276-Neb | chr2 | Neb | 0.202796353 | 0 | 0 | 16277 | 1488 |
| chr2_52204333_52194380_-9953-Neb | chr2 | Neb | 0.405592706 | 0 | 0 | 9954 | 1260 |
| chr2_52204728_52204307_-421-na | chr2 | na | 0.101398176 | 0.182309179 | 0 | 422 | 422 |
| chr2_52227555_52227244_-311-Neb | chr2 | Neb | 0.202796353 | 0 | 0 | 312 | 312 |
| chr2_52249514_52245281_-4233-Neb | chr2 | Neb | 15.00693011 | 0 | 0 | 4234 | 729 |
| chr2_52258904_52232921_-25983-Neb | chr2 | Neb | 0 | 0.364618359 | 0 | 25984 | 3645 |
| chr2_52279948_52277358_-2590-Neb | chr2 | Neb | 0.202796353 | 0 | 0 | 2591 | 732 |
| chr2_52280642_52279632_-1010-Neb | chr2 | Neb | 0 | 0.182309179 | 0 | 1011 | 420 |
| chr2_52289730_52288771_-959-Neb | chr2 | Neb | 0 | 0 | 0.180646022 | 960 | 213 |
| chr2_52303735_52302442_-1293-Neb | chr2 | Neb | 3.853130704 | 0 | 0.541938067 | 1294 | 204 |
| chr2_53056817_53014803_+42014-Fmnl2 | chr2 | Fmnl2 | 0 | 0 | 0.180646022 | 42015 | 42015 |
| chr2_53126597_53114112_+12485-na | chr2 | na | 0.101398176 | 0 | 0 | 12486 | 12486 |
| chr2_5350745_5350641_-104-na | chr2 | na | 0.202796353 | 0 | 0 | 105 | 105 |
| chr2_57268082_57264156_+3926-Gpd2 | chr2 | Gpd2 | 0.608389058 | 0 | 0 | 3927 | 3927 |
| chr2_57290016_57267973_+22043-Gpd2 | chr2 | Gpd2 | 0 | 0.364618359 | 0.541938067 | 22044 | 282 |
| chr2_57307095_57289845_+17250-Gpd2 | chr2 | Gpd2 | 0.202796353 | 0 | 0.541938067 | 17251 | 559 |
| chr2_57345424_57289845_+55579-Gpd2 | chr2 | Gpd2 | 0.202796353 | 0 | 0 | 55580 | 1063 |
| chr2_57345424_57338847_+6577-Gpd2 | chr2 | Gpd2 | 0.405592706 | 0 | 0 | 6578 | 504 |
| chr2_5877001_5876364_-637-Sec61a2 | chr2 | Sec61a2 | 0 | 0.182309179 | 0 | 638 | 359 |
| chr2_5932542_5923869_-8673-Dhtkd1 | chr2 | Dhtkd1 | 0.202796353 | 0 | 0 | 8674 | 833 |
| chr2_59920538_59912402_-8136-Baz2b | chr2 | Baz2b | 0 | 0 | 0.180646022 | 8137 | 1541 |
| chr2_59923755_59911682_-12073-Baz2b | chr2 | Baz2b | 0 | 0.182309179 | 0 | 12074 | 1992 |
| chr2_59932170_59907439_-24731-Baz2b | chr2 | Baz2b | 1.013981764 | 0.546927538 | 0.361292045 | 24732 | 2765 |
| chr2_59936751_59900658_-36093-Baz2b | chr2 | Baz2b | 0 | 0.182309179 | 0 | 36094 | 3813 |
| chr2_59958492_59948087_-10405-Baz2b | chr2 | Baz2b | 0.608389058 | 0.182309179 | 0 | 10406 | 381 |
| chr2_59962630_59922143_-40487-Baz2b | chr2 | Baz2b | 0 | 0.182309179 | 0 | 40488 | 2393 |
| chr2_60006189_60001506_-4683-Baz2b | chr2 | Baz2b | 0.202796353 | 0.273463769 | 0 | 4684 | 198 |
| chr2_60012323_60001506_-10817-Baz2b | chr2 | Baz2b | 0 | 0.182309179 | 0 | 10818 | 10818 |
| chr2_60029135_60001506_-27629-na | chr2 | na | 0.101398176 | 0 | 0 | 27630 | 27630 |
| chr2_6010428_5982992_+27436-Upf2 | chr2 | Upf2 | 0 | 0.182309179 | 0 | 27437 | 413 |
| chr2_60443380_60441086_-2294-Pla2r1 | chr2 | Pla2r1 | 0 | 0.182309179 | 0 | 2295 | 241 |
| chr2_60674728_60668288_-6440-Itgb6 | chr2 | Itgb6 | 0 | 0 | 0.361292045 | 6441 | 742 |
| chr2_60797742_60779731_-18011-Rbms1 | chr2 | Rbms1 | 0 | 0 | 0.180646022 | 18012 | 389 |
| chr2_61175581_61127694_+47887-Gm39833 | chr2 | Gm39833 | 0.202796353 | 0 | 0 | 47888 | 47888 |
| chr2_61785559_61776673_+8886-Psmd14 | chr2 | Psmd14 | 0 | 0 | 1.083876134 | 8887 | 309 |
| chr2_62286963_62267401_+19562-Slc4a10 | chr2 | Slc4a10 | 0 | 0.182309179 | 0 | 19563 | 823 |
| chr2_62313349_62267401_+45948-Slc4a10 | chr2 | Slc4a10 | 0 | 0 | 0.180646022 | 45949 | 1785 |
| chr2_6394555_6391055_+3500-Usp6nl | chr2 | Usp6nl | 0 | 0.729236718 | 0 | 3501 | 151 |
| chr2_67519353_67476718_+42635-Xirp2 | chr2 | Xirp2 | 0 | 0.182309179 | 0 | 42636 | 9983 |
| chr2_67519353_67505034_+14319-Xirp2 | chr2 | Xirp2 | 0.811185411 | 0.911545897 | 0.722584089 | 14320 | 9696 |
| chr2_69696971_69690063_-6908-Fastkd1 | chr2 | Fastkd1 | 0 | 0 | 0.180646022 | 6909 | 620 |
| chr2_69938312_69914414_+23898-Ubr3 | chr2 | Ubr3 | 0 | 0 | 0.180646022 | 23899 | 920 |
| chr2_69959663_69951343_+8320-Ubr3 | chr2 | Ubr3 | 0.202796353 | 0 | 0 | 8321 | 797 |
| chr2_69979468_69965374_+14094-Ubr3 | chr2 | Ubr3 | 0 | 0.182309179 | 0 | 14095 | 1121 |
| chr2_69983225_69973149_+10076-Ubr3 | chr2 | Ubr3 | 0 | 0 | 0.361292045 | 10077 | 798 |
| chr2_69983261_69938084_+45177-Ubr3 | chr2 | Ubr3 | 0 | 0 | 0.541938067 | 45178 | 2682 |
| chr2_69988896_69973149_+15747-Ubr3 | chr2 | Ubr3 | 0 | 0 | 0.180646022 | 15748 | 945 |
| chr2_69988896_69977719_+11177-Ubr3 | chr2 | Ubr3 | 0.202796353 | 0 | 0 | 11178 | 484 |
| chr2_69993812_69914414_+79398-Ubr3 | chr2 | Ubr3 | 0 | 0.182309179 | 0 | 79399 | 3834 |
| chr2_70786995_70754440_-32555-Tlk1 | chr2 | Tlk1 | 0 | 0.182309179 | 0 | 32556 | 383 |
| chr2_70786995_70786877_-118-Tlk1 | chr2 | Tlk1 | 0 | 0.546927538 | 0 | 119 | 119 |
| chr2_71282610_71275094_-7516-Slc25a12 | chr2 | Slc25a12 | 0.202796353 | 0 | 0 | 7517 | 790 |
| chr2_71512168_71506785_+5383-Metap1d | chr2 | Metap1d | 0.202796353 | 0.182309179 | 0.361292045 | 5384 | 2021 |
| chr2_71888994_71875406_+13588-Pdk1 | chr2 | Pdk1 | 0.202796353 | 0 | 0 | 13589 | 854 |
| chr2_72384205_72371727_+12478-Zak | chr2 | Zak | 0.202796353 | 0 | 0 | 12479 | 329 |
| chr2_72971514_72970155_-1359-Sp3 | chr2 | Sp3 | 0.202796353 | 0 | 0 | 1360 | 1360 |
| chr2_76548372_76540104_+8268-Osbpl6 | chr2 | Osbpl6 | 0 | 0.182309179 | 0 | 8269 | 270 |
| chr2_76579310_76564784_+14526-Osbpl6 | chr2 | Osbpl6 | 0 | 0.182309179 | 0 | 14527 | 570 |
| chr2_76718597_76716749_-1848-Ttn | chr2 | Ttn | 0.202796353 | 0 | 0 | 1849 | 1494 |
| chr2_76728602_76716749_-11853-Ttn | chr2 | Ttn | 0 | 0.182309179 | 0 | 11854 | 8019 |
| chr2_76731285_76730778_+507-na | chr2 | na | 0.202796353 | 0.182309179 | 0.361292045 | 508 | 508 |
| chr2_76735100_76720480_-14620-Ttn | chr2 | Ttn | 0 | 0.182309179 | 0 | 14621 | 9786 |
| chr2_76761385_76756267_-5118-Ttn | chr2 | Ttn | 0.202796353 | 0 | 0 | 5119 | 2361 |
| chr2_76761385_76759838_-1547-Ttn | chr2 | Ttn | 0 | 0.364618359 | 0 | 1548 | 588 |
| chr2_76768684_76761086_-7598-Ttn | chr2 | Ttn | 0 | 0.182309179 | 0 | 7599 | 5355 |
| chr2_76769456_76762152_-7304-Ttn | chr2 | Ttn | 0 | 0.182309179 | 0 | 7305 | 5346 |
| chr2_76770577_76769710_+867-na | chr2 | na | 0 | 0.182309179 | 0 | 868 | 868 |
| chr2_76770641_76769540_-1101-Ttn | chr2 | Ttn | 0 | 0 | 0.541938067 | 1102 | 894 |
| chr2_76771806_76771124_-682-Ttn | chr2 | Ttn | 0.202796353 | 0 | 0 | 683 | 585 |
| chr2_76776964_76769938_-7026-Ttn | chr2 | Ttn | 0 | 0.182309179 | 0 | 7027 | 3294 |
| chr2_76782489_76781964_-525-Ttn | chr2 | Ttn | 0.202796353 | 0 | 0 | 526 | 434 |
| chr2_76788819_76788514_-305-Ttn | chr2 | Ttn | 0.202796353 | 0.182309179 | 0 | 306 | 306 |
| chr2_76789652_76789261_+391-na | chr2 | na | 0.202796353 | 0 | 0 | 392 | 392 |
| chr2_76790384_76779006_-11378-Ttn | chr2 | Ttn | 0 | 0.729236718 | 0 | 11379 | 5737 |
| chr2_76791319_76784472_+6847-na | chr2 | na | 0 | 0 | 0.180646022 | 6848 | 6848 |
| chr2_76802497_76795479_-7018-Ttn | chr2 | Ttn | 0.202796353 | 0 | 0 | 7019 | 3024 |
| chr2_76812603_76811142_-1461-Ttn | chr2 | Ttn | 0 | 0 | 0.180646022 | 1462 | 922 |
| chr2_76827078_76802231_-24847-Ttn | chr2 | Ttn | 0 | 0.182309179 | 0 | 24848 | 5175 |
| chr2_76831874_76826447_-5427-Ttn | chr2 | Ttn | 0 | 0 | 0.451615056 | 5428 | 1902 |
| chr2_76847808_76826075_-21733-Ttn | chr2 | Ttn | 0 | 0.364618359 | 0 | 21734 | 4563 |
| chr2_76848356_76847725_-631-Ttn | chr2 | Ttn | 0.709787235 | 0 | 0.632261078 | 632 | 162 |
| chr2_76848642_76848279_-363-Ttn | chr2 | Ttn | 0 | 0.546927538 | 1.083876134 | 364 | 162 |
| chr2_76851764_76822451_-29313-Ttn | chr2 | Ttn | 0 | 0.729236718 | 0 | 29314 | 6027 |
| chr2_76852137_76831788_-20349-Ttn | chr2 | Ttn | 0.101398176 | 0.911545897 | 0 | 20350 | 3699 |
| chr2_76852137_76847388_-4749-Ttn | chr2 | Ttn | 0.811185411 | 1.458473435 | 0.722584089 | 4750 | 1296 |
| chr2_76852137_76850570_-1567-Ttn | chr2 | Ttn | 0 | 0 | 0.903230112 | 1568 | 366 |
| chr2_76853688_76803293_-50395-Ttn | chr2 | Ttn | 0 | 0 | 0.361292045 | 50396 | 10494 |
| chr2_76853928_76852834_-1094-Ttn | chr2 | Ttn | 0 | 0 | 0.361292045 | 1095 | 330 |
| chr2_76856366_76847388_-8978-Ttn | chr2 | Ttn | 0 | 1.458473435 | 0.361292045 | 8979 | 1878 |
| chr2_76856366_76850570_-5796-Ttn | chr2 | Ttn | 0 | 0 | 1.083876134 | 5797 | 948 |
| chr2_76856366_76853605_-2761-Ttn | chr2 | Ttn | 0.202796353 | 1.640782614 | 1.264522156 | 2762 | 414 |
| chr2_76856904_76848559_-8345-Ttn | chr2 | Ttn | 0 | 0.182309179 | 0 | 8346 | 1734 |
| chr2_76857987_76847388_-10599-Ttn | chr2 | Ttn | 0 | 0 | 0.361292045 | 10600 | 2298 |
| chr2_76857987_76854150_-3837-Ttn | chr2 | Ttn | 0 | 0 | 0.180646022 | 3838 | 672 |
| chr2_76859997_76853851_-6146-Ttn | chr2 | Ttn | 0 | 0.182309179 | 0 | 6147 | 921 |
| chr2_76861177_76857904_-3273-Ttn | chr2 | Ttn | 0 | 0.364618359 | 0 | 3274 | 336 |
| chr2_76862427_76861097_-1330-Ttn | chr2 | Ttn | 0 | 0.546927538 | 0 | 1331 | 324 |
| chr2_76868320_76867194_+1126-na | chr2 | na | 0 | 0.182309179 | 0 | 1127 | 1127 |
| chr2_76873998_76857904_-16094-Ttn | chr2 | Ttn | 0 | 0 | 0.180646022 | 16095 | 3099 |
| chr2_76884197_76847388_-36809-Ttn | chr2 | Ttn | 0.202796353 | 0 | 0 | 36810 | 8715 |
| chr2_76884197_76857904_-26293-Ttn | chr2 | Ttn | 2.433556234 | 0.729236718 | 0 | 26294 | 6501 |
| chr2_76884633_76881064_-3569-Ttn | chr2 | Ttn | 0.202796353 | 0 | 0 | 3570 | 1137 |
| chr2_76885403_76878674_-6729-Ttn | chr2 | Ttn | 0.405592706 | 0 | 0 | 6730 | 2823 |
| chr2_76885775_76881064_-4711-Ttn | chr2 | Ttn | 0 | 0 | 0.361292045 | 4712 | 1974 |
| chr2_76893177_76857904_-35273-Ttn | chr2 | Ttn | 0 | 0.364618359 | 0 | 35274 | 11577 |
| chr2_76893177_76892899_-278-Ttn | chr2 | Ttn | 15.51392099 | 5.83389374 | 2.167752268 | 279 | 279 |
| chr2_76894423_76857904_-36519-Ttn | chr2 | Ttn | 0.202796353 | 0 | 0 | 36520 | 11859 |
| chr2_76894423_76894142_-281-Ttn | chr2 | Ttn | 0.202796353 | 0 | 0 | 282 | 282 |
| chr2_76894902_76894142_-760-Ttn | chr2 | Ttn | 0.608389058 | 0.546927538 | 0.180646022 | 761 | 561 |
| chr2_76894902_76894624_-278-Ttn | chr2 | Ttn | 0.608389058 | 0.820391307 | 0 | 279 | 279 |
| chr2_76896202_76894142_-2060-Ttn | chr2 | Ttn | 0.405592706 | 0 | 0 | 2061 | 1407 |
| chr2_76896202_76895142_-1060-Ttn | chr2 | Ttn | 0 | 0 | 0.180646022 | 1061 | 846 |
| chr2_76896980_76896320_-660-Ttn | chr2 | Ttn | 0 | 0.182309179 | 0 | 661 | 561 |
| chr2_76897400_76897122_-278-Ttn | chr2 | Ttn | 1.41957447 | 0 | 0.722584089 | 279 | 279 |
| chr2_76898723_76898445_-278-Ttn | chr2 | Ttn | 0.202796353 | 0.182309179 | 0 | 279 | 279 |
| chr2_76902122_76888886_-13236-Ttn | chr2 | Ttn | 0.202796353 | 0 | 0.180646022 | 13237 | 7995 |
| chr2_76902122_76894624_-7498-Ttn | chr2 | Ttn | 0 | 0.182309179 | 0 | 7499 | 5172 |
| chr2_76902122_76901841_-281-Ttn | chr2 | Ttn | 0 | 0 | 0.722584089 | 282 | 282 |
| chr2_76906532_76853605_-52927-Ttn | chr2 | Ttn | 0.202796353 | 0 | 0 | 52928 | 18624 |
| chr2_76906532_76857904_-48628-Ttn | chr2 | Ttn | 0.202796353 | 0 | 0.180646022 | 48629 | 17874 |
| chr2_76906532_76859914_-46618-Ttn | chr2 | Ttn | 0.202796353 | 0 | 0 | 46619 | 17703 |
| chr2_76906532_76903063_-3469-Ttn | chr2 | Ttn | 0.202796353 | 0.182309179 | 0.180646022 | 3470 | 843 |
| chr2_76906532_76906254_-278-Ttn | chr2 | Ttn | 0.608389058 | 0 | 0 | 279 | 279 |
| chr2_76927275_76927087_-188-Ttn | chr2 | Ttn | 0.202796353 | 0 | 0.180646022 | 189 | 189 |
| chr2_76934370_76934139_-231-Ttn | chr2 | Ttn | 0.202796353 | 0 | 0 | 232 | 232 |
| chr2_76937833_76936382_-1451-Ttn | chr2 | Ttn | 0 | 0.182309179 | 0 | 1452 | 569 |
| chr2_76939307_76937573_-1734-Ttn | chr2 | Ttn | 0 | 0 | 0.180646022 | 1735 | 1047 |
| chr2_76939307_76939044_-263-Ttn | chr2 | Ttn | 3.041945292 | 0.911545897 | 0.903230112 | 264 | 264 |
| chr2_76940110_76934139_-5971-Ttn | chr2 | Ttn | 0 | 0 | 0.361292045 | 5972 | 1848 |
| chr2_76942439_76934139_-8300-Ttn | chr2 | Ttn | 0 | 0.182309179 | 0 | 8301 | 2373 |
| chr2_76943467_76943021_-446-Ttn | chr2 | Ttn | 0.202796353 | 0 | 0.722584089 | 447 | 366 |
| chr2_76948072_76946392_-1680-Ttn | chr2 | Ttn | 0 | 0.182309179 | 0 | 1681 | 694 |
| chr2_76951707_76921551_-30156-Ttn | chr2 | Ttn | 0 | 0 | 0.180646022 | 30157 | 7644 |
| chr2_76952049_76951315_-734-Ttn | chr2 | Ttn | 0.202796353 | 0 | 0 | 735 | 407 |
| chr2_76952178_76906254_-45924-Ttn | chr2 | Ttn | 0 | 0.182309179 | 0 | 45925 | 10828 |
| chr2_76952178_76942847_-9331-Ttn | chr2 | Ttn | 0 | 0.182309179 | 0 | 9332 | 4498 |
| chr2_76955088_76724778_-230310-Ttn | chr2 | Ttn | 0 | 0 | 0.361292045 | 230311 | 89911 |
| chr2_76955088_76921551_-33537-Ttn | chr2 | Ttn | 0.202796353 | 0 | 0 | 33538 | 8674 |
| chr2_76955088_76927087_-28001-Ttn | chr2 | Ttn | 0.202796353 | 0 | 0.180646022 | 28002 | 8242 |
| chr2_76969292_76969140_-152-Ttn | chr2 | Ttn | 0 | 0.182309179 | 0 | 153 | 153 |
| chr2_76977296_76921551_-55745-na | chr2 | na | 0 | 0 | 0.180646022 | 55746 | 55746 |
| chr2_76977296_76967054_-10242-Ttn | chr2 | Ttn | 0 | 0 | 0.903230112 | 10243 | 1574 |
| chr2_76980195_76977093_-3102-Ttn | chr2 | Ttn | 0 | 0 | 0.180646022 | 3103 | 308 |
| chr2_79658349_79657147_+1202-Ssfa2 | chr2 | Ssfa2 | 0 | 0 | 0.180646022 | 1203 | 1203 |
| chr2_80530303_80512677_-17626-Nckap1 | chr2 | Nckap1 | 0 | 0.182309179 | 0 | 17627 | 1377 |
| chr2_80540225_80535320_-4905-na | chr2 | na | 0 | 0 | 0.090323011 | 4906 | 4906 |
| chr2_80545146_80530158_-14988-Nckap1 | chr2 | Nckap1 | 0 | 0 | 0.180646022 | 14989 | 838 |
| chr2_84377045_84368547_-8498-Calcrl | chr2 | Calcrl | 0 | 0.364618359 | 0 | 8499 | 749 |
| chr2_84855649_84854412_+1237-Slc43a1 | chr2 | Slc43a1 | 0 | 0.182309179 | 0 | 1238 | 313 |
| chr2_91004862_91001006_+3856-Celf1 | chr2 | Celf1 | 0 | 0 | 0.722584089 | 3857 | 509 |
| chr2_91262943_91260404_+2539-Pacsin3 | chr2 | Pacsin3 | 0 | 0.182309179 | 0 | 2540 | 725 |
| chr2_91591791_91585722_+6069-Ckap5 | chr2 | Ckap5 | 0.202796353 | 0 | 0 | 6070 | 546 |
| chr2_91712865_91712080_+785-Harbi1 | chr2 | Harbi1 | 0 | 0.364618359 | 0 | 786 | 786 |
| chr2_91769087_91766447_+2640-Ambra1 | chr2 | Ambra1 | 0.202796353 | 0 | 0 | 2641 | 670 |
| chr2_92230724_92214049_+16675-na | chr2 | na | 0.304194529 | 0.546927538 | 0.451615056 | 16676 | 16676 |
| chr2_92236842_92214049_+22793-na | chr2 | na | 0 | 0.182309179 | 0 | 22794 | 22794 |
| chr2_92252922_92214049_+38873-na | chr2 | na | 0 | 0.09115459 | 0 | 38874 | 38874 |
| chr2_93806141_93795635_-10506-Ext2 | chr2 | Ext2 | 0 | 0.364618359 | 0 | 10507 | 453 |
| chr2_93813964_93795635_-18329-Ext2 | chr2 | Ext2 | 0 | 0 | 0.180646022 | 18330 | 1109 |
| chr2_93813964_93806025_-7939-Ext2 | chr2 | Ext2 | 0.202796353 | 0 | 0 | 7940 | 773 |
| chr2_94008592_94003053_-5539-Alkbh3 | chr2 | Alkbh3 | 0.202796353 | 0 | 0 | 5540 | 441 |
| chr3_100182753_100169936_+12817-Gdap2 | chr3 | Gdap2 | 0 | 0.182309179 | 0 | 12818 | 700 |
| chr3_103029212_103028789_+423-Csde1 | chr3 | Csde1 | 0.202796353 | 0 | 0 | 424 | 424 |
| chr3_103052931_103039933_+12998-Csde1 | chr3 | Csde1 | 0 | 0.182309179 | 0 | 12999 | 1674 |
| chr3_103092464_103085307_+7157-Ampd1 | chr3 | Ampd1 | 0 | 0.182309179 | 0 | 7158 | 1007 |
| chr3_103095762_103079029_+16733-Ampd1 | chr3 | Ampd1 | 0.405592706 | 0 | 0 | 16734 | 1760 |
| chr3_104479850_104465487_-14363-Lrig2 | chr3 | Lrig2 | 0.202796353 | 0 | 0 | 14364 | 836 |
| chr3_104497554_104479782_-17772-Lrig2 | chr3 | Lrig2 | 0.202796353 | 0 | 0 | 17773 | 1074 |
| chr3_104497554_104480109_-17445-Lrig2 | chr3 | Lrig2 | 0 | 0.182309179 | 0 | 17446 | 1005 |
| chr3_104892311_104882793_+9518-St7l | chr3 | St7l | 0 | 0.182309179 | 0 | 9519 | 447 |
| chr3_104895798_104882793_+13005-St7l | chr3 | St7l | 0 | 0 | 0.541938067 | 13006 | 623 |
| chr3_106539603_106530183_-9420-Cept1 | chr3 | Cept1 | 0 | 0.273463769 | 0 | 9421 | 9421 |
| chr3_107326304_107324752_-1552-Rbm15 | chr3 | Rbm15 | 0 | 0.546927538 | 0 | 1553 | 1553 |
| chr3_108435966_108431465_-4501-Sars | chr3 | Sars | 0.202796353 | 0 | 0 | 4502 | 540 |
| chr3_108908083_108907787_-296-Prpf38b | chr3 | Prpf38b | 0 | 0.364618359 | 0 | 297 | 213 |
| chr3_113570057_113558093_-11964-Amy1 | chr3 | Amy1 | 0 | 0 | 0.361292045 | 11965 | 1391 |
| chr3_116332882_116322082_-10800-Cdc14a | chr3 | Cdc14a | 0 | 0.182309179 | 0 | 10801 | 458 |
| chr3_116351272_116322082_-29190-Cdc14a | chr3 | Cdc14a | 0.202796353 | 0 | 0 | 29191 | 588 |
| chr3_116404618_116348454_-56164-Cdc14a | chr3 | Cdc14a | 0.405592706 | 0 | 0 | 56165 | 379 |
| chr3_116404618_116392822_-11796-Cdc14a | chr3 | Cdc14a | 0 | 0.182309179 | 0.180646022 | 11797 | 169 |
| chr3_116590292_116589491_-801-Trmt13 | chr3 | Trmt13 | 0.304194529 | 0 | 0 | 802 | 240 |
| chr3_116621508_116618256_+3252-Sass6 | chr3 | Sass6 | 0 | 0 | 0.180646022 | 3253 | 810 |
| chr3_116651499_116645366_-6133-Mfsd14a | chr3 | Mfsd14a | 0 | 0.182309179 | 0.180646022 | 6134 | 402 |
| chr3_116757805_116754873_-2932-Agl | chr3 | Agl | 0 | 0.182309179 | 0 | 2933 | 2933 |
| chr3_116776659_116757555_-19104-Agl | chr3 | Agl | 0 | 0.182309179 | 0 | 19105 | 19105 |
| chr3_116779380_116768786_-10594-Agl | chr3 | Agl | 0.202796353 | 0 | 0 | 10595 | 1054 |
| chr3_116781344_116776529_-4815-Agl | chr3 | Agl | 0.202796353 | 0.182309179 | 0.180646022 | 4816 | 913 |
| chr3_116782643_116776529_-6114-Agl | chr3 | Agl | 8.720243171 | 6.198512099 | 6.683902826 | 6115 | 1201 |
| chr3_116782643_116778619_-4024-Agl | chr3 | Agl | 1.622370823 | 2.005400973 | 2.890336357 | 4025 | 1070 |
| chr3_116793822_116786530_-7292-Agl | chr3 | Agl | 0.202796353 | 0 | 0 | 7293 | 1201 |
| chr3_121235764_121228310_-7454-Tmem56 | chr3 | Tmem56 | 0.202796353 | 0.182309179 | 0 | 7455 | 544 |
| chr3_121235764_121235043_-721-Tmem56 | chr3 | Tmem56 | 0 | 0.182309179 | 0 | 722 | 300 |
| chr3_121796485_121779555_-16930-Abcd3 | chr3 | Abcd3 | 0 | 0 | 0.180646022 | 16931 | 717 |
| chr3_122969249_122947361_-21888-Usp53 | chr3 | Usp53 | 0 | 0 | 0.180646022 | 21889 | 2837 |
| chr3_126965162_126958883_-6279-Ank2 | chr3 | Ank2 | 0.202796353 | 0 | 0 | 6280 | 896 |
| chr3_126981944_126962251_-19693-Ank2 | chr3 | Ank2 | 0 | 0.182309179 | 0 | 19694 | 903 |
| chr3_127011051_127002678_-8373-Ank2 | chr3 | Ank2 | 0 | 0 | 0.180646022 | 8374 | 396 |
| chr3_127057091_127032116_-24975-Ank2 | chr3 | Ank2 | 0 | 0 | 0.361292045 | 24976 | 804 |
| chr3_127617294_127583584_+33710-Zgrf1 | chr3 | Zgrf1 | 0 | 0 | 0.180646022 | 33711 | 3676 |
| chr3_130005096_129996681_-8415-Sec24b | chr3 | Sec24b | 0.202796353 | 0 | 0 | 8416 | 572 |
| chr3_130011872_129996681_-15191-Sec24b | chr3 | Sec24b | 0 | 0 | 0.180646022 | 15192 | 1071 |
| chr3_130020642_129996681_-23961-Sec24b | chr3 | Sec24b | 3.041945292 | 2.005400973 | 1.445168179 | 23962 | 1176 |
| chr3_130041447_129754817_-286630-Sec24b | chr3 | Sec24b | 0.202796353 | 0 | 0 | 286631 | 286631 |
| chr3_132752669_132705513_+47156-Tbck | chr3 | Tbck | 0.405592706 | 0 | 0 | 47157 | 1927 |
| chr3_132774596_132705513_+69083-Tbck | chr3 | Tbck | 0 | 0 | 0.180646022 | 69084 | 2103 |
| chr3_132774596_132751187_+23409-Tbck | chr3 | Tbck | 0 | 0.182309179 | 0 | 23410 | 375 |
| chr3_132815349_132751187_+64162-Tbck | chr3 | Tbck | 0.202796353 | 0.182309179 | 0 | 64163 | 711 |
| chr3_132917553_132904658_-12895-Npnt | chr3 | Npnt | 0 | 0.182309179 | 0 | 12896 | 1080 |
| chr3_133488717_133485512_-3205-Tet2 | chr3 | Tet2 | 0.202796353 | 0.182309179 | 0.180646022 | 3206 | 3206 |
| chr3_135524627_135506909_+17718-Manba | chr3 | Manba | 0 | 0 | 0.180646022 | 17719 | 885 |
| chr3_135524627_135511803_+12824-Manba | chr3 | Manba | 0 | 0.182309179 | 0 | 12825 | 684 |
| chr3_135669339_135626595_-42744-Nfkb1 | chr3 | Nfkb1 | 0 | 0.182309179 | 0 | 42745 | 492 |
| chr3_135669339_135655500_-13839-Nfkb1 | chr3 | Nfkb1 | 0 | 0 | 0.722584089 | 13840 | 326 |
| chr3_136797926_136797726_+200-Ppp3ca | chr3 | Ppp3ca | 0 | 0.546927538 | 0 | 201 | 201 |
| chr3_136835594_136834544_-1050-na | chr3 | na | 0.202796353 | 0 | 0 | 1051 | 1051 |
| chr3_136881540_136797726_+83814-Ppp3ca | chr3 | Ppp3ca | 0 | 0.364618359 | 0 | 83815 | 724 |
| chr3_137908619_137885273_+23346-Dnajb14 | chr3 | Dnajb14 | 0 | 0 | 0.180646022 | 23347 | 1157 |
| chr3_137908619_137902187_+6432-Dnajb14 | chr3 | Dnajb14 | 0 | 0 | 0.180646022 | 6433 | 839 |
| chr3_138478930_138472151_-6779-Metap1 | chr3 | Metap1 | 0.811185411 | 0 | 0 | 6780 | 376 |
| chr3_138969975_138955322_-14653-Rap1gds1 | chr3 | Rap1gds1 | 0 | 0 | 0.361292045 | 14654 | 1004 |
| chr3_141857596_141856349_-1247-Bmpr1b | chr3 | Bmpr1b | 0.202796353 | 0 | 0 | 1248 | 491 |
| chr3_142352885_142352734_-151-Pdlim5 | chr3 | Pdlim5 | 0 | 0.364618359 | 0.180646022 | 152 | 152 |
| chr3_142839362_142828881_-10481-Pkn2 | chr3 | Pkn2 | 0 | 0 | 0.180646022 | 10482 | 633 |
| chr3_144712728_144705513_-7215-Sh3glb1 | chr3 | Sh3glb1 | 0 | 0.182309179 | 0 | 7216 | 498 |
| chr3_145315417_145313991_+1426-Col24a1 | chr3 | Col24a1 | 0 | 0 | 0.180646022 | 1427 | 1427 |
| chr3_145492347_145484646_+7701-Col24a1 | chr3 | Col24a1 | 0 | 0.182309179 | 0 | 7702 | 324 |
| chr3_145524299_145491067_+33232-Col24a1 | chr3 | Col24a1 | 0 | 0.182309179 | 0 | 33233 | 648 |
| chr3_146493843_146457078_-36765-Spata1 | chr3 | Spata1 | 0.202796353 | 0 | 0 | 36766 | 36766 |
| chr3_146493843_146463185_-30658-Spata1 | chr3 | Spata1 | 0 | 0.182309179 | 0 | 30659 | 1519 |
| chr3_146495151_146457078_-38073-Spata1 | chr3 | Spata1 | 0.202796353 | 0 | 0 | 38074 | 38074 |
| chr3_146495151_146487200_-7951-Spata1 | chr3 | Spata1 | 0.405592706 | 0 | 0 | 7952 | 7952 |
| chr3_146748047_146737934_-10113-Prkacb | chr3 | Prkacb | 0 | 0.182309179 | 0 | 10114 | 511 |
| chr3_146757768_146732382_-25386-Prkacb | chr3 | Prkacb | 0.202796353 | 0 | 0 | 25387 | 1216 |
| chr3_146915789_146886391_+29398-Ttll7 | chr3 | Ttll7 | 0 | 0 | 0.180646022 | 29399 | 29399 |
| chr3_146931613_146901324_+30289-Ttll7 | chr3 | Ttll7 | 0.405592706 | 0 | 0.180646022 | 30290 | 1153 |
| chr3_146961764_146901324_+60440-Ttll7 | chr3 | Ttll7 | 0 | 0.546927538 | 0 | 60441 | 2097 |
| chr3_148859533_148858733_-800-Adgrl2 | chr3 | Adgrl2 | 0.202796353 | 0 | 0 | 801 | 801 |
| chr3_152364047_152357840_+6207-Usp33 | chr3 | Usp33 | 0 | 0 | 0.361292045 | 6208 | 575 |
| chr3_152428832_152420015_+8817-Zzz3 | chr3 | Zzz3 | 0.405592706 | 0.364618359 | 0.180646022 | 8818 | 1836 |
| chr3_152428847_152420015_+8832-Zzz3 | chr3 | Zzz3 | 0 | 0 | 0.180646022 | 8833 | 1851 |
| chr3_152747773_152734513_+13260-Pigk | chr3 | Pigk | 0 | 0.182309179 | 0 | 13261 | 841 |
| chr3_152766523_152738186_+28337-Pigk | chr3 | Pigk | 0.202796353 | 0 | 0 | 28338 | 973 |
| chr3_152846670_152821528_-25142-St6galnac5 | chr3 | St6galnac5 | 0 | 0 | 0.180646022 | 25143 | 733 |
| chr3_19229768_19227476_-2292-Pde7a | chr3 | Pde7a | 0 | 0 | 0.180646022 | 2293 | 447 |
| chr3_19243201_19227476_-15725-Pde7a | chr3 | Pde7a | 0 | 0 | 0.180646022 | 15726 | 1503 |
| chr3_19672997_19670924_+2073-Trim55 | chr3 | Trim55 | 0 | 0 | 0.180646022 | 2074 | 624 |
| chr3_20108123_20064566_+43557-Hltf | chr3 | Hltf | 0 | 0 | 0.180646022 | 43558 | 2089 |
| chr3_20125477_19256778_-868699-Hps3 | chr3 | Hps3 | 0 | 0 | 0.361292045 | 868700 | 868700 |
| chr3_27470346_27451314_-19032-Fndc3b | chr3 | Fndc3b | 0.202796353 | 0 | 0 | 19033 | 1541 |
| chr3_27957337_27951487_+5850-na | chr3 | na | 0 | 0.09115459 | 0 | 5851 | 5851 |
| chr3_28056544_28044944_+11600-Pld1 | chr3 | Pld1 | 0.202796353 | 0 | 0 | 11601 | 469 |
| chr3_28120828_28085786_+35042-Pld1 | chr3 | Pld1 | 0 | 0.364618359 | 0 | 35043 | 861 |
| chr3_28577573_28493967_+83606-Tnik | chr3 | Tnik | 0 | 0 | 0.541938067 | 83607 | 1098 |
| chr3_28638517_28454908_+183609-Tnik | chr3 | Tnik | 0 | 0 | 0.180646022 | 183610 | 183610 |
| chr3_28638517_28493967_+144550-Tnik | chr3 | Tnik | 0 | 0 | 0.180646022 | 144551 | 2607 |
| chr3_29987177_29977259_-9918-Mecom | chr3 | Mecom | 0 | 0.182309179 | 0.180646022 | 9919 | 1750 |
| chr3_30238321_30237978_-343-Mecom | chr3 | Mecom | 0 | 0.182309179 | 0 | 344 | 344 |
| chr3_30942652_30907048_-35604-Phc3 | chr3 | Phc3 | 0 | 0 | 0.361292045 | 35605 | 2673 |
| chr3_30942652_30914095_-28557-Phc3 | chr3 | Phc3 | 0 | 0.182309179 | 0.180646022 | 28558 | 2230 |
| chr3_30951031_30914095_-36936-Phc3 | chr3 | Phc3 | 0 | 0 | 0.180646022 | 36937 | 2485 |
| chr3_32555541_32544067_+11474-Mfn1 | chr3 | Mfn1 | 0 | 0 | 0.180646022 | 11475 | 439 |
| chr3_32865966_32847810_+18156-Usp13 | chr3 | Usp13 | 0 | 0.182309179 | 0 | 18157 | 511 |
| chr3_32865966_32854556_+11410-Usp13 | chr3 | Usp13 | 0.405592706 | 0 | 0 | 11411 | 450 |
| chr3_32865966_32858736_+7230-Usp13 | chr3 | Usp13 | 0.202796353 | 0 | 0 | 7231 | 7231 |
| chr3_32876972_32861771_+15201-Usp13 | chr3 | Usp13 | 0 | 0 | 0.180646022 | 15202 | 423 |
| chr3_32881508_32861771_+19737-Usp13 | chr3 | Usp13 | 0 | 0 | 0.180646022 | 19738 | 611 |
| chr3_32886588_32861771_+24817-Usp13 | chr3 | Usp13 | 0.608389058 | 0 | 0.361292045 | 24818 | 777 |
| chr3_32886588_32876878_+9710-Usp13 | chr3 | Usp13 | 0 | 0 | 0.180646022 | 9711 | 449 |
| chr3_32919070_32894764_+24306-Usp13 | chr3 | Usp13 | 0.202796353 | 0 | 0 | 24307 | 1150 |
| chr3_32931742_32855268_+76474-Usp13 | chr3 | Usp13 | 0 | 0 | 0.180646022 | 76475 | 76475 |
| chr3_32931742_32914099_+17643-Usp13 | chr3 | Usp13 | 0.202796353 | 0 | 0 | 17644 | 691 |
| chr3_35841115_35831366_+9749-Atp11b | chr3 | Atp11b | 0 | 0.182309179 | 0 | 9750 | 895 |
| chr3_35855256_35831366_+23890-Atp11b | chr3 | Atp11b | 0.202796353 | 0 | 0 | 23891 | 1304 |
| chr3_36662933_36650048_-12885-Trpc3 | chr3 | Trpc3 | 0 | 0.182309179 | 0 | 12886 | 805 |
| chr3_41717864_41647445_-70419-Sclt1 | chr3 | Sclt1 | 0 | 0 | 0.180646022 | 70420 | 1539 |
| chr3_51326032_51294093_-31939-Elf2 | chr3 | Elf2 | 0.101398176 | 0 | 0 | 31940 | 577 |
| chr3_51326032_51299783_-26249-Elf2 | chr3 | Elf2 | 0.202796353 | 0 | 0 | 26250 | 584 |
| chr3_51326035_51294093_-31942-Elf2 | chr3 | Elf2 | 0.202796353 | 0.182309179 | 0.180646022 | 31943 | 580 |
| chr3_51456003_51438339_+17664-Naa15 | chr3 | Naa15 | 0 | 0.182309179 | 0 | 17665 | 1118 |
| chr3_51542833_51526845_-15988-Setd7 | chr3 | Setd7 | 0.202796353 | 0 | 0 | 15989 | 750 |
| chr3_52094068_52093182_-886-na | chr3 | na | 0.405592706 | 0 | 0 | 887 | 887 |
| chr3_5245645_5241670_+3975-Zfhx4 | chr3 | Zfhx4 | 0.608389058 | 1.276164256 | 0 | 3976 | 3133 |
| chr3_53012964_52998257_-14707-Cog6 | chr3 | Cog6 | 0 | 0.364618359 | 0 | 14708 | 797 |
| chr3_59042413_59031546_+10867-Med12l | chr3 | Med12l | 0 | 0 | 0.180646022 | 10868 | 490 |
| chr3_59093695_59091407_+2288-Med12l | chr3 | Med12l | 0 | 0.182309179 | 0 | 2289 | 497 |
| chr3_59235208_59227683_+7525-Med12l | chr3 | Med12l | 0 | 0 | 0.180646022 | 7526 | 575 |
| chr3_60370654_60370212_-442-na | chr3 | na | 0.202796353 | 0 | 0 | 443 | 443 |
| chr3_60529804_60528756_+1048-Mbnl1 | chr3 | Mbnl1 | 0.202796353 | 0.546927538 | 0 | 1049 | 1049 |
| chr3_60595764_60595594_+170-Mbnl1 | chr3 | Mbnl1 | 0 | 0 | 0.180646022 | 171 | 171 |
| chr3_60613520_60595594_+17926-Mbnl1 | chr3 | Mbnl1 | 0 | 0 | 0.180646022 | 17927 | 741 |
| chr3_60615773_60613263_+2510-Mbnl1 | chr3 | Mbnl1 | 0.202796353 | 0 | 0 | 2511 | 466 |
| chr3_63954004_63943222_-10782-Slc33a1 | chr3 | Slc33a1 | 0.405592706 | 0 | 0 | 10783 | 905 |
| chr3_63993973_63985600_+8373-Gmps | chr3 | Gmps | 0 | 0.546927538 | 0 | 8374 | 888 |
| chr3_64140804_64086471_+54333-Vmn2r1 | chr3 | Vmn2r1 | 0 | 0.182309179 | 0 | 54334 | 1284 |
| chr3_67007645_66994517_+13128-Rsrc1 | chr3 | Rsrc1 | 0 | 0.364618359 | 0 | 13129 | 13129 |
| chr3_79515249_79500338_-14911-Fnip2 | chr3 | Fnip2 | 0.202796353 | 0 | 0 | 14912 | 713 |
| chr3_79518191_79506207_-11984-na | chr3 | na | 0 | 0 | 0.090323011 | 11985 | 11985 |
| chr3_84519703_84509808_-9895-Arfip1 | chr3 | Arfip1 | 0 | 0 | 0.180646022 | 9896 | 555 |
| chr3_84904079_84863274_+40805-Fbxw7 | chr3 | Fbxw7 | 0 | 0.364618359 | 0 | 40806 | 40806 |
| chr3_84904079_84903501_+578-Fbxw7 | chr3 | Fbxw7 | 0.202796353 | 0 | 0.722584089 | 579 | 579 |
| chr3_85636539_85632682_-3857-na | chr3 | na | 0 | 0 | 0.361292045 | 3858 | 3858 |
| chr3_85676261_85672223_-4038-Fam160a1 | chr3 | Fam160a1 | 0.405592706 | 0 | 0 | 4039 | 1448 |
| chr3_85730885_85720416_-10469-Fam160a1 | chr3 | Fam160a1 | 0.608389058 | 0 | 0.541938067 | 10470 | 873 |
| chr3_85741374_85720416_-20958-Fam160a1 | chr3 | Fam160a1 | 0.405592706 | 0 | 0.180646022 | 20959 | 1097 |
| chr3_85741374_85730259_-11115-Fam160a1 | chr3 | Fam160a1 | 0.202796353 | 0 | 0.361292045 | 11116 | 851 |
| chr3_85817291_85730259_-87032-Fam160a1 | chr3 | Fam160a1 | 0 | 0.182309179 | 0 | 87033 | 87033 |
| chr3_86417996_86291427_+126569-Lrba | chr3 | Lrba | 0.405592706 | 0.364618359 | 0.180646022 | 126570 | 5211 |
| chr3_86445473_86291427_+154046-Lrba | chr3 | Lrba | 0 | 0.182309179 | 0 | 154047 | 5378 |
| chr3_86664590_86372078_+292512-Lrba | chr3 | Lrba | 0 | 0.364618359 | 0 | 292513 | 1925 |
| chr3_87100673_87097742_-2931-Kirrel | chr3 | Kirrel | 0.202796353 | 0.182309179 | 0 | 2932 | 2932 |
| chr3_88156525_88155678_+847-Mef2d | chr3 | Mef2d | 0 | 0 | 0.180646022 | 848 | 389 |
| chr3_88159592_88155678_+3914-Mef2d | chr3 | Mef2d | 0.202796353 | 0 | 0 | 3915 | 795 |
| chr3_88163087_88155678_+7409-Mef2d | chr3 | Mef2d | 0 | 0.182309179 | 0 | 7410 | 1137 |
| chr3_88351581_88350016_+1565-Smg5 | chr3 | Smg5 | 0.202796353 | 0.364618359 | 0 | 1566 | 950 |
| chr3_88353138_88346445_+6693-Smg5 | chr3 | Smg5 | 0.202796353 | 0 | 0 | 6694 | 1521 |
| chr3_88700514_88699951_+563-2810403A07Rik | chr3 | 2810403A07Rik | 0.405592706 | 0.455772948 | 0 | 564 | 312 |
| chr3_88907582_88905452_+2130-Gon4l | chr3 | Gon4l | 0.608389058 | 0 | 0 | 2131 | 322 |
| chr3_88985790_88964674_+21116-Ash1l | chr3 | Ash1l | 0.202796353 | 0 | 0.361292045 | 21117 | 21117 |
| chr3_88985790_88981233_+4557-Ash1l | chr3 | Ash1l | 0 | 0.182309179 | 0 | 4558 | 4558 |
| chr3_89007877_88964674_+43203-Ash1l | chr3 | Ash1l | 0.202796353 | 0.182309179 | 0.180646022 | 43204 | 43204 |
| chr3_89007877_88965812_+42065-Ash1l | chr3 | Ash1l | 0.202796353 | 0.546927538 | 0.541938067 | 42066 | 6061 |
| chr3_89007877_88999728_+8149-Ash1l | chr3 | Ash1l | 0 | 0 | 0.180646022 | 8150 | 986 |
| chr3_89007877_89007142_+735-Ash1l | chr3 | Ash1l | 0.202796353 | 0.364618359 | 0 | 736 | 736 |
| chr3_89023210_89007142_+16068-Ash1l | chr3 | Ash1l | 0 | 0 | 0.541938067 | 16069 | 916 |
| chr3_89033332_88965812_+67520-na | chr3 | na | 0 | 0.09115459 | 0 | 67521 | 67521 |
| chr3_89054517_88965812_+88705-Ash1l | chr3 | Ash1l | 0 | 0 | 0.180646022 | 88706 | 7295 |
| chr3_89058640_89023031_+35609-Ash1l | chr3 | Ash1l | 0 | 0.182309179 | 0 | 35610 | 1535 |
| chr3_89076277_89063786_+12491-Ash1l | chr3 | Ash1l | 0 | 0.182309179 | 0 | 12492 | 1382 |
| chr3_90047899_90034065_-13834-Ubap2l | chr3 | Ubap2l | 0 | 0 | 0.541938067 | 13835 | 776 |
| chr3_90278547_90276880_+1667-Dennd4b | chr3 | Dennd4b | 0.202796353 | 0 | 0 | 1668 | 1061 |
| chr3_90354510_90350387_+4123-Gatad2b | chr3 | Gatad2b | 0 | 0 | 0.541938067 | 4124 | 822 |
| chr3_9265420_9264535_+885-Zbtb10 | chr3 | Zbtb10 | 0.202796353 | 0 | 0.361292045 | 886 | 886 |
| chr3_94393140_94391074_+2066-Rorc | chr3 | Rorc | 0 | 0 | 0.180646022 | 2067 | 462 |
| chr3_94448034_94447260_+774-Mrpl9 | chr3 | Mrpl9 | 0 | 0.182309179 | 0 | 775 | 333 |
| chr3_94531389_94511042_-20347-Snx27 | chr3 | Snx27 | 0 | 0 | 0.180646022 | 20348 | 696 |
| chr3_94531389_94519048_-12341-Snx27 | chr3 | Snx27 | 1.41957447 | 0.364618359 | 0.541938067 | 12342 | 606 |
| chr3_94531389_94524186_-7203-Snx27 | chr3 | Snx27 | 0.202796353 | 0 | 0 | 7204 | 363 |
| chr3_94562000_94519048_-42952-Snx27 | chr3 | Snx27 | 0.405592706 | 0 | 0 | 42953 | 838 |
| chr3_94562000_94524186_-37814-Snx27 | chr3 | Snx27 | 0 | 0.182309179 | 0 | 37815 | 595 |
| chr3_94562284_94519048_-43236-Snx27 | chr3 | Snx27 | 0 | 0 | 0.180646022 | 43237 | 904 |
| chr3_94562284_94561769_-515-Snx27 | chr3 | Snx27 | 0 | 0.09115459 | 0 | 516 | 298 |
| chr3_94856201_94853950_+2251-Pogz | chr3 | Pogz | 0.202796353 | 0 | 0 | 2252 | 284 |
| chr3_94999113_94983983_+15130-Pi4kb | chr3 | Pi4kb | 0 | 0 | 0.180646022 | 15131 | 2043 |
| chr3_95036745_95032818_-3927-Psmd4 | chr3 | Psmd4 | 0.202796353 | 0.182309179 | 0 | 3928 | 1138 |
| chr3_95036745_95034566_-2179-Psmd4 | chr3 | Psmd4 | 0.202796353 | 0 | 0 | 2180 | 656 |
| chr3_95078182_95063655_-14527-Pip5k1a | chr3 | Pip5k1a | 0.405592706 | 0 | 0 | 14528 | 1695 |
| chr3_95082828_95072403_-10425-Pip5k1a | chr3 | Pip5k1a | 0 | 0.364618359 | 0 | 10426 | 368 |
| chr3_95476182_95466793_+9389-Arnt | chr3 | Arnt | 0.202796353 | 0 | 0 | 9390 | 576 |
| chr3_95490378_95470177_+20201-Arnt | chr3 | Arnt | 0.608389058 | 0 | 0 | 20202 | 1433 |
| chr3_96042928_96041338_-1590-Vps45 | chr3 | Vps45 | 0.202796353 | 0 | 0.180646022 | 1591 | 417 |
| chr3_96148355_96147532_-823-na | chr3 | na | 0.202796353 | 0 | 0 | 824 | 824 |
| chr3_97690853_96046352_-1644501-Chd1l | chr3 | Chd1l | 0 | 0.182309179 | 0 | 1644502 | 1644502 |
| chr3_97710444_97706872_-3572-Pde4dip | chr3 | Pde4dip | 0.811185411 | 0.182309179 | 0.180646022 | 3573 | 563 |
| chr3_97719717_97713068_-6649-Pde4dip | chr3 | Pde4dip | 0.202796353 | 0 | 0 | 6650 | 1511 |
| chr3_97724321_97713068_-11253-Pde4dip | chr3 | Pde4dip | 0 | 0.182309179 | 0 | 11254 | 1744 |
| chr3_97754263_97747765_-6498-Pde4dip | chr3 | Pde4dip | 0 | 0.182309179 | 0 | 6499 | 684 |
| chr3_97754545_97713068_-41477-Pde4dip | chr3 | Pde4dip | 0 | 0.182309179 | 0 | 41478 | 3301 |
| chr3_97754545_97741318_-13227-Pde4dip | chr3 | Pde4dip | 0 | 0.182309179 | 0 | 13228 | 1468 |
| chr3_97754545_97742489_-12056-Pde4dip | chr3 | Pde4dip | 0 | 0 | 0.180646022 | 12057 | 1340 |
| chr3_97796808_97792739_-4069-Pde4dip | chr3 | Pde4dip | 0 | 0.546927538 | 0 | 4070 | 495 |
| chr3_97824325_97824215_-110-Pde4dip | chr3 | Pde4dip | 0.202796353 | 0 | 0 | 111 | 111 |
| chr3_98072922_98070860_+2062-Notch2 | chr3 | Notch2 | 0 | 0.182309179 | 0 | 2063 | 596 |
| chr3_98135831_98111550_+24281-Notch2 | chr3 | Notch2 | 0 | 0.182309179 | 0 | 24282 | 2485 |
| chr3_99340647_99314744_+25903-Tbx15 | chr3 | Tbx15 | 0.405592706 | 0 | 0 | 25904 | 503 |
| chr4_100412140_100407813_+4327-Ror1 | chr4 | Ror1 | 0 | 0.182309179 | 0 | 4328 | 692 |
| chr4_101179794_101166543_-13251-Jak1 | chr4 | Jak1 | 0 | 0 | 0.180646022 | 13252 | 1105 |
| chr4_103072843_103058142_-14701-Wdr78 | chr4 | Wdr78 | 0 | 0 | 0.541938067 | 14702 | 14702 |
| chr4_103140901_103139358_+1543-Mier1 | chr4 | Mier1 | 0 | 0.182309179 | 0 | 1544 | 357 |
| chr4_103150628_103139358_+11270-Mier1 | chr4 | Mier1 | 0.405592706 | 0.182309179 | 0 | 11271 | 759 |
| chr4_103214092_103205106_-8986-Slc35d1 | chr4 | Slc35d1 | 0.202796353 | 0 | 0 | 8987 | 526 |
| chr4_103321868_103319022_+2846-Oma1 | chr4 | Oma1 | 0 | 0.182309179 | 0 | 2847 | 736 |
| chr4_103331379_103325019_+6360-Oma1 | chr4 | Oma1 | 0.608389058 | 0.182309179 | 0 | 6361 | 486 |
| chr4_103341833_103319022_+22811-Oma1 | chr4 | Oma1 | 0.202796353 | 0 | 0 | 22812 | 22812 |
| chr4_103356629_103353502_+3127-Oma1 | chr4 | Oma1 | 0 | 0 | 0.361292045 | 3128 | 3128 |
| chr4_106413674_106370069_+43605-Usp24 | chr4 | Usp24 | 0 | 0 | 0.180646022 | 43606 | 4332 |
| chr4_106430591_106409611_+20980-Usp24 | chr4 | Usp24 | 0.608389058 | 0.911545897 | 0.180646022 | 20981 | 1852 |
| chr4_108250873_108237342_-13531-Zyg11b | chr4 | Zyg11b | 0.405592706 | 0 | 0 | 13532 | 710 |
| chr4_108272383_108241821_-30562-Zyg11b | chr4 | Zyg11b | 0.202796353 | 0 | 0 | 30563 | 1916 |
| chr4_108272383_108250495_-21888-Zyg11b | chr4 | Zyg11b | 0 | 0 | 0.180646022 | 21889 | 1455 |
| chr4_108272383_108255201_-17182-Zyg11b | chr4 | Zyg11b | 0.202796353 | 0 | 0 | 17183 | 1239 |
| chr4_108272383_108265818_-6565-Zyg11b | chr4 | Zyg11b | 0.811185411 | 0.729236718 | 0.361292045 | 6566 | 921 |
| chr4_108503035_108486454_+16581-Zcchc11 | chr4 | Zcchc11 | 0 | 0.182309179 | 0 | 16582 | 670 |
| chr4_108628708_108623451_+5257-Cc2d1b | chr4 | Cc2d1b | 0 | 0.182309179 | 0 | 5258 | 1891 |
| chr4_108691791_108656927_-34864-Zfyve9 | chr4 | Zfyve9 | 0 | 0 | 0.180646022 | 34865 | 1215 |
| chr4_109024277_109013510_+10767-Nrd1 | chr4 | Nrd1 | 0 | 0.182309179 | 0 | 10768 | 845 |
| chr4_109060984_109058582_+2402-na | chr4 | na | 0 | 0.182309179 | 0 | 2403 | 2403 |
| chr4_109107949_109083089_-24860-Osbpl9 | chr4 | Osbpl9 | 0.405592706 | 0.729236718 | 0 | 24861 | 689 |
| chr4_109133820_109083089_-50731-Osbpl9 | chr4 | Osbpl9 | 0 | 0.182309179 | 0.180646022 | 50732 | 766 |
| chr4_109385562_109361670_+23892-Eps15 | chr4 | Eps15 | 0 | 0 | 0.180646022 | 23893 | 1516 |
| chr4_11218825_11213706_-5119-Ints8 | chr4 | Ints8 | 0 | 0.546927538 | 0 | 5120 | 342 |
| chr4_11248311_11218612_-29699-Ints8 | chr4 | Ints8 | 0 | 0 | 0.180646022 | 29700 | 2109 |
| chr4_11248311_11239307_-9004-Ints8 | chr4 | Ints8 | 0 | 0 | 0.722584089 | 9005 | 712 |
| chr4_11304390_11285054_-19336-Dpy19l4 | chr4 | Dpy19l4 | 0 | 0 | 0.180646022 | 19337 | 1202 |
| chr4_115860117_115857032_+3085-Mknk1 | chr4 | Mknk1 | 0.202796353 | 0 | 0 | 3086 | 200 |
| chr4_116515499_116510522_+4977-Ipp | chr4 | Ipp | 0 | 0.182309179 | 0 | 4978 | 774 |
| chr4_117589157_117552968_+36189-Eri3 | chr4 | Eri3 | 0.202796353 | 0 | 0 | 36190 | 1838 |
| chr4_117593162_117564621_+28541-Eri3 | chr4 | Eri3 | 0.202796353 | 0.182309179 | 0.180646022 | 28542 | 547 |
| chr4_117593162_117564627_+28535-Eri3 | chr4 | Eri3 | 0.202796353 | 0 | 0 | 28536 | 1854 |
| chr4_117615241_117564621_+50620-na | chr4 | na | 0 | 0.09115459 | 0 | 50621 | 50621 |
| chr4_117649406_117582600_+66806-Eri3 | chr4 | Eri3 | 0.202796353 | 0 | 0 | 66807 | 1755 |
| chr4_118622193_118621361_+832-Ebna1bp2 | chr4 | Ebna1bp2 | 0.405592706 | 0 | 0 | 833 | 297 |
| chr4_119533809_119533313_-496-AA415398 | chr4 | AA415398 | 0 | 0.729236718 | 0 | 497 | 306 |
| chr4_119585824_119560603_+25221-Foxj3 | chr4 | Foxj3 | 0 | 0.182309179 | 0 | 25222 | 461 |
| chr4_119620348_119616508_+3840-Foxj3 | chr4 | Foxj3 | 0 | 0.182309179 | 0 | 3841 | 724 |
| chr4_120508212_120462013_+46199-Scmh1 | chr4 | Scmh1 | 0 | 0.182309179 | 0 | 46200 | 1139 |
| chr4_123399509_123394070_-5439-Macf1 | chr4 | Macf1 | 0 | 0 | 0.180646022 | 5440 | 1188 |
| chr4_124746182_124743636_+2546-Inpp5b | chr4 | Inpp5b | 0 | 0.364618359 | 0 | 2547 | 239 |
| chr4_124821359_124820205_+1154-Mtf1 | chr4 | Mtf1 | 0 | 0.182309179 | 0 | 1155 | 371 |
| chr4_126125818_126123716_+2102-Stk40 | chr4 | Stk40 | 0 | 0 | 0.541938067 | 2103 | 350 |
| chr4_126275257_126272678_+2579-Trappc3 | chr4 | Trappc3 | 0 | 0.182309179 | 0 | 2580 | 497 |
| chr4_126370382_126355006_-15376-Ago3 | chr4 | Ago3 | 0 | 0 | 0.180646022 | 15377 | 722 |
| chr4_126377041_126346631_-30410-Ago3 | chr4 | Ago3 | 0.202796353 | 0 | 0 | 30411 | 1816 |
| chr4_126816474_126814256_+2218-AU040320 | chr4 | AU040320 | 0.405592706 | 0 | 0 | 2219 | 349 |
| chr4_126915665_126877629_-38036-Zmym4 | chr4 | Zmym4 | 0 | 0.182309179 | 0 | 38037 | 3111 |
| chr4_126915665_126899932_-15733-na | chr4 | na | 0.101398176 | 0 | 0 | 15734 | 15734 |
| chr4_126915665_126902543_-13122-Zmym4 | chr4 | Zmym4 | 0 | 0 | 0.180646022 | 13123 | 1747 |
| chr4_127100760_127078408_+22352-Zmym6 | chr4 | Zmym6 | 0.202796353 | 0 | 0 | 22353 | 1011 |
| chr4_127124480_127108548_+15932-Zmym6 | chr4 | Zmym6 | 0 | 0.182309179 | 0 | 15933 | 15933 |
| chr4_131897871_131891207_+6664-Srsf4 | chr4 | Srsf4 | 0 | 0 | 0.180646022 | 6665 | 471 |
| chr4_132000304_131967788_-32516-Epb41 | chr4 | Epb41 | 0 | 0.546927538 | 0 | 32517 | 1143 |
| chr4_132000304_131982453_-17851-Epb41 | chr4 | Epb41 | 0 | 0.182309179 | 0 | 17852 | 531 |
| chr4_132071171_132006958_-64213-na | chr4 | na | 0 | 0 | 0.180646022 | 64214 | 64214 |
| chr4_132377554_131989715_-387839-Gmeb1 | chr4 | Gmeb1 | 0 | 0 | 0.180646022 | 387840 | 387840 |
| chr4_133077145_133055013_+22132-Ahdc1 | chr4 | Ahdc1 | 0.202796353 | 0 | 0 | 22133 | 5521 |
| chr4_133185752_133184969_+783-Wasf2 | chr4 | Wasf2 | 0.202796353 | 0 | 0 | 784 | 289 |
| chr4_134154396_134148010_-6386-Cep85 | chr4 | Cep85 | 0 | 0 | 0.361292045 | 6387 | 746 |
| chr4_135338078_135329373_-8705-Srrm1 | chr4 | Srrm1 | 0 | 0 | 0.180646022 | 8706 | 588 |
| chr4_136257986_136253746_+4240-Tcea3 | chr4 | Tcea3 | 0 | 0 | 0.361292045 | 4241 | 443 |
| chr4_136264610_136253746_+10864-Tcea3 | chr4 | Tcea3 | 0 | 0 | 0.361292045 | 10865 | 607 |
| chr4_136545402_136539125_+6277-Luzp1 | chr4 | Luzp1 | 0 | 0 | 0.180646022 | 6278 | 3420 |
| chr4_136545402_136540356_+5046-Luzp1 | chr4 | Luzp1 | 0.202796353 | 0 | 0 | 5047 | 3328 |
| chr4_136545541_136539125_+6416-Luzp1 | chr4 | Luzp1 | 0 | 0.182309179 | 0.180646022 | 6417 | 3559 |
| chr4_136545541_136540356_+5185-Luzp1 | chr4 | Luzp1 | 0.202796353 | 0 | 0 | 5186 | 3467 |
| chr4_136577204_136568573_-8631-Kdm1a | chr4 | Kdm1a | 0 | 0 | 0.180646022 | 8632 | 590 |
| chr4_137510167_136255150_+1255017-na | chr4 | na | 0 | 0.09115459 | 0 | 1255018 | 1255018 |
| chr4_137523890_137510542_+13348-Hspg2 | chr4 | Hspg2 | 0.506990882 | 1.367318845 | 0.722584089 | 13349 | 13349 |
| chr4_137608182_137604523_+3659-Usp48 | chr4 | Usp48 | 0.405592706 | 0 | 0.180646022 | 3660 | 531 |
| chr4_137644498_137613682_+30816-Usp48 | chr4 | Usp48 | 0 | 0.182309179 | 0 | 30817 | 5811 |
| chr4_137644498_137634898_+9600-Usp48 | chr4 | Usp48 | 0 | 0.182309179 | 0 | 9601 | 664 |
| chr4_137655249_137621062_+34187-Usp48 | chr4 | Usp48 | 0 | 0.364618359 | 0 | 34188 | 2349 |
| chr4_138084370_138082893_+1477-Eif4g3 | chr4 | Eif4g3 | 0.405592706 | 0 | 0 | 1478 | 222 |
| chr4_138084370_138082914_+1456-Eif4g3 | chr4 | Eif4g3 | 0 | 0.546927538 | 0.180646022 | 1457 | 201 |
| chr4_138087565_138045343_+42222-Eif4g3 | chr4 | Eif4g3 | 0 | 0.273463769 | 0 | 42223 | 573 |
| chr4_138097359_138082893_+14466-Eif4g3 | chr4 | Eif4g3 | 0 | 0 | 0.180646022 | 14467 | 571 |
| chr4_138105436_138087533_+17903-na | chr4 | na | 0 | 0 | 0.180646022 | 17904 | 17904 |
| chr4_138105436_138096752_+8684-Eif4g3 | chr4 | Eif4g3 | 0.811185411 | 0 | 0 | 8685 | 516 |
| chr4_138126634_138096752_+29882-Eif4g3 | chr4 | Eif4g3 | 0 | 0 | 0.180646022 | 29883 | 1450 |
| chr4_138126634_138125810_+824-Eif4g3 | chr4 | Eif4g3 | 0.202796353 | 0 | 0 | 825 | 825 |
| chr4_138172677_138163447_+9230-Eif4g3 | chr4 | Eif4g3 | 0.202796353 | 0 | 0 | 9231 | 858 |
| chr4_138188222_138045343_+142879-Eif4g3 | chr4 | Eif4g3 | 0.202796353 | 0 | 0 | 142880 | 142880 |
| chr4_138241736_138221526_+20210-Hp1bp3 | chr4 | Hp1bp3 | 0.202796353 | 0 | 0 | 20211 | 1763 |
| chr4_138241792_138221526_+20266-Hp1bp3 | chr4 | Hp1bp3 | 0.202796353 | 0 | 0 | 20267 | 1819 |
| chr4_139367213_139365195_+2018-Emc1 | chr4 | Emc1 | 0 | 0.182309179 | 0 | 2019 | 632 |
| chr4_139393419_139388368_+5051-Ubr4 | chr4 | Ubr4 | 0 | 0.546927538 | 0 | 5052 | 842 |
| chr4_139402832_139399562_+3270-Ubr4 | chr4 | Ubr4 | 0 | 0.182309179 | 0 | 3271 | 790 |
| chr4_141180532_141167713_+12819-Fbxo42 | chr4 | Fbxo42 | 0 | 0.182309179 | 0 | 12820 | 517 |
| chr4_141522392_141516836_-5556-Spen | chr4 | Spen | 0 | 0.273463769 | 0 | 5557 | 804 |
| chr4_141695510_141692255_-3255-Ddi2 | chr4 | Ddi2 | 0.202796353 | 0 | 0 | 3256 | 294 |
| chr4_141698858_141692255_-6603-Ddi2 | chr4 | Ddi2 | 0.405592706 | 0 | 0 | 6604 | 423 |
| chr4_141708456_141692255_-16201-Ddi2 | chr4 | Ddi2 | 0.202796353 | 0 | 0 | 16202 | 915 |
| chr4_142411344_142410457_-887-na | chr4 | na | 0 | 0.182309179 | 0 | 888 | 888 |
| chr4_143180991_143170036_-10955-Prdm2 | chr4 | Prdm2 | 0 | 0 | 0.180646022 | 10956 | 372 |
| chr4_143180991_143179288_-1703-Prdm2 | chr4 | Prdm2 | 0.202796353 | 0 | 0 | 1704 | 219 |
| chr4_143194734_143170036_-24698-Prdm2 | chr4 | Prdm2 | 0.202796353 | 0.182309179 | 0 | 24699 | 446 |
| chr4_145020269_145016735_-3534-Vps13d | chr4 | Vps13d | 0 | 0.364618359 | 0 | 3535 | 329 |
| chr4_145088438_145086704_-1734-Vps13d | chr4 | Vps13d | 0 | 0 | 0.180646022 | 1735 | 567 |
| chr4_145100067_145016735_-83332-Vps13d | chr4 | Vps13d | 0.202796353 | 0 | 0 | 83333 | 4036 |
| chr4_145100067_145044961_-55106-Vps13d | chr4 | Vps13d | 0.202796353 | 0 | 0 | 55107 | 3707 |
| chr4_145100070_145044961_-55109-Vps13d | chr4 | Vps13d | 0.202796353 | 0 | 0.180646022 | 55110 | 3710 |
| chr4_145105921_145016735_-89186-Vps13d | chr4 | Vps13d | 0.405592706 | 0 | 0 | 89187 | 4237 |
| chr4_145156782_145154569_-2213-Vps13d | chr4 | Vps13d | 0.608389058 | 0.364618359 | 0.180646022 | 2214 | 2214 |
| chr4_152020708_152020190_-518-Zbtb48 | chr4 | Zbtb48 | 0.506990882 | 0 | 0 | 519 | 254 |
| chr4_152052709_152050535_+2174-Nol9 | chr4 | Nol9 | 0.405592706 | 0 | 0 | 2175 | 575 |
| chr4_152237766_152206764_+31002-Acot7 | chr4 | Acot7 | 0 | 0 | 0.722584089 | 31003 | 569 |
| chr4_152524368_152518093_+6275-Nphp4 | chr4 | Nphp4 | 0.405592706 | 0 | 0 | 6276 | 310 |
| chr4_154152870_154151628_+1242-Wrap73 | chr4 | Wrap73 | 0.202796353 | 0 | 0 | 1243 | 404 |
| chr4_155102200_155089486_+12714-Morn1 | chr4 | Morn1 | 0 | 0.182309179 | 0 | 12715 | 7976 |
| chr4_155160956_155157370_-3586-Ski | chr4 | Ski | 0.405592706 | 0 | 0 | 3587 | 1186 |
| chr4_155543193_155515631_+27562-Gnb1 | chr4 | Gnb1 | 0 | 0 | 0.180646022 | 27563 | 519 |
| chr4_155585449_155576993_+8456-Nadk | chr4 | Nadk | 0.608389058 | 0 | 0 | 8457 | 614 |
| chr4_155610636_155609839_+797-Slc35e2 | chr4 | Slc35e2 | 0 | 0.182309179 | 0 | 798 | 617 |
| chr4_155612737_155609839_+2898-Slc35e2 | chr4 | Slc35e2 | 0 | 0 | 0.180646022 | 2899 | 866 |
| chr4_155648404_155625727_+22677-Cdk11b | chr4 | Cdk11b | 0 | 0 | 0.180646022 | 22678 | 1742 |
| chr4_155843139_155842905_+234-Mxra8 | chr4 | Mxra8 | 0 | 0.182309179 | 0 | 235 | 158 |
| chr4_155855619_155820329_+35290-Dvl1 | chr4 | Dvl1 | 0.202796353 | 0 | 0 | 35291 | 35291 |
| chr4_155902416_155649012_+253404-Dvl1 | chr4 | Dvl1 | 0 | 0.182309179 | 0 | 253405 | 253405 |
| chr4_155902416_155896567_+5849-Acap3 | chr4 | Acap3 | 0 | 0.182309179 | 0.541938067 | 5850 | 1023 |
| chr4_19650440_19627633_-22807-Wwp1 | chr4 | Wwp1 | 0 | 0.364618359 | 0.180646022 | 22808 | 1537 |
| chr4_21993820_21982386_+11434-Faxc | chr4 | Faxc | 0.202796353 | 0 | 0 | 11435 | 640 |
| chr4_22377077_22375661_+1416-Fbxl4 | chr4 | Fbxl4 | 0.405592706 | 0 | 0 | 1417 | 707 |
| chr4_22403745_22375661_+28084-Fbxl4 | chr4 | Fbxl4 | 0 | 0.546927538 | 0 | 28085 | 1512 |
| chr4_22403745_22385907_+17838-Fbxl4 | chr4 | Fbxl4 | 0 | 0.182309179 | 0 | 17839 | 805 |
| chr4_22427462_22375661_+51801-Fbxl4 | chr4 | Fbxl4 | 0.202796353 | 0 | 0 | 51802 | 3021 |
| chr4_22427462_22376496_+50966-Fbxl4 | chr4 | Fbxl4 | 0.202796353 | 0 | 0 | 50967 | 2911 |
| chr4_32581045_32561778_+19267-Bach2 | chr4 | Bach2 | 0 | 0 | 0.361292045 | 19268 | 3026 |
| chr4_32646660_32643540_+3120-Casp8ap2 | chr4 | Casp8ap2 | 0 | 0.182309179 | 0 | 3121 | 3121 |
| chr4_32686424_32683543_+2881-Mdn1 | chr4 | Mdn1 | 0 | 0 | 0.541938067 | 2882 | 555 |
| chr4_33325147_33320519_+4628-Rngtt | chr4 | Rngtt | 0 | 0 | 0.722584089 | 4629 | 487 |
| chr4_33356196_33320519_+35677-Rngtt | chr4 | Rngtt | 0.202796353 | 0 | 0 | 35678 | 1152 |
| chr4_34595236_34579743_-15493-Orc3 | chr4 | Orc3 | 0 | 0 | 0.180646022 | 15494 | 803 |
| chr4_34656262_34647786_+8476-Rars2 | chr4 | Rars2 | 0 | 0.182309179 | 0 | 8477 | 537 |
| chr4_34848032_34839332_-8700-Zfp292 | chr4 | Zfp292 | 0.202796353 | 0 | 0 | 8701 | 8701 |
| chr4_34852406_34839332_-13074-Zfp292 | chr4 | Zfp292 | 0 | 0.364618359 | 0 | 13075 | 13075 |
| chr4_3570372_3569364_-1008-Tmem68 | chr4 | Tmem68 | 0.202796353 | 0 | 0 | 1009 | 441 |
| chr4_40167191_40163719_+3472-Aco1 | chr4 | Aco1 | 0.405592706 | 0 | 0 | 3473 | 434 |
| chr4_40193861_40180172_+13689-Aco1 | chr4 | Aco1 | 0 | 0.182309179 | 0 | 13690 | 1299 |
| chr4_41229848_41216566_-13282-Ubap2 | chr4 | Ubap2 | 0 | 0.182309179 | 0.361292045 | 13283 | 578 |
| chr4_43232608_43227925_+4683-Unc13b | chr4 | Unc13b | 0 | 0.09115459 | 0 | 4684 | 149 |
| chr4_43549529_43544280_-5249-Tln1 | chr4 | Tln1 | 0 | 0.182309179 | 0 | 5250 | 1925 |
| chr4_45992270_45989078_+3192-Tdrd7 | chr4 | Tdrd7 | 0.202796353 | 0 | 0 | 3193 | 397 |
| chr4_46029807_46025657_+4150-Tdrd7 | chr4 | Tdrd7 | 0.202796353 | 0 | 0 | 4151 | 664 |
| chr4_46097078_46092139_+4939-Tmod1 | chr4 | Tmod1 | 0 | 0 | 0.180646022 | 4940 | 383 |
| chr4_47396585_47383749_+12836-Tgfbr1 | chr4 | Tgfbr1 | 0.202796353 | 0 | 0 | 12837 | 720 |
| chr4_48140485_48131765_+8720-Stx17 | chr4 | Stx17 | 0 | 0 | 0.361292045 | 8721 | 403 |
| chr4_48167043_48131765_+35278-Stx17 | chr4 | Stx17 | 0 | 0.182309179 | 0 | 35279 | 742 |
| chr4_48470072_48456734_-13338-Tex10 | chr4 | Tex10 | 0 | 0.182309179 | 0 | 13339 | 1985 |
| chr4_53517681_53481464_+36217-Slc44a1 | chr4 | Slc44a1 | 0.405592706 | 0 | 0 | 36218 | 370 |
| chr4_53696978_53659513_+37465-Fsd1l | chr4 | Fsd1l | 0 | 0.182309179 | 0 | 37466 | 1036 |
| chr4_55014847_55007533_+7314-Zfp462 | chr4 | Zfp462 | 0 | 0 | 0.180646022 | 7315 | 6009 |
| chr4_55378289_55366777_+11512-Rad23b | chr4 | Rad23b | 1.216778117 | 0 | 0 | 11513 | 487 |
| chr4_55381116_55368045_+13071-Rad23b | chr4 | Rad23b | 0 | 0 | 0.361292045 | 13072 | 533 |
| chr4_55381116_55370204_+10912-Rad23b | chr4 | Rad23b | 0 | 0.182309179 | 0 | 10913 | 453 |
| chr4_55385601_55378234_+7367-Rad23b | chr4 | Rad23b | 0.608389058 | 0 | 0 | 7368 | 640 |
| chr4_55385601_55382480_+3121-Rad23b | chr4 | Rad23b | 0.202796353 | 0 | 0 | 3122 | 456 |
| chr4_56912130_56899026_-13104-Tmem245 | chr4 | Tmem245 | 0 | 0.182309179 | 0.180646022 | 13105 | 710 |
| chr4_56923508_56906215_-17293-Tmem245 | chr4 | Tmem245 | 0 | 0.182309179 | 0 | 17294 | 572 |
| chr4_57206304_57204907_-1397-Ptpn3 | chr4 | Ptpn3 | 0 | 0.729236718 | 0 | 1398 | 300 |
| chr4_57710143_57709451_+692-na | chr4 | na | 0 | 0 | 0.361292045 | 693 | 693 |
| chr4_58841605_58832704_-8901-AI314180 | chr4 | AI314180 | 0.202796353 | 0 | 0.180646022 | 8902 | 888 |
| chr4_58844202_58840675_-3527-AI314180 | chr4 | AI314180 | 0 | 0.364618359 | 0 | 3528 | 520 |
| chr4_58879134_58861525_-17609-AI314180 | chr4 | AI314180 | 0 | 0.182309179 | 0.361292045 | 17610 | 900 |
| chr4_59494610_59493186_-1424-Ptbp3 | chr4 | Ptbp3 | 0 | 0.546927538 | 0 | 1425 | 286 |
| chr4_59524476_59514274_-10202-na | chr4 | na | 0 | 0 | 0.090323011 | 10203 | 10203 |
| chr4_59691338_59686677_+4661-E130308A19Rik | chr4 | E130308A19Rik | 0 | 0.182309179 | 0 | 4662 | 1664 |
| chr4_62314425_62300696_-13729-Fkbp15 | chr4 | Fkbp15 | 0 | 0 | 0.180646022 | 13730 | 1954 |
| chr4_62336547_62329348_-7199-Fkbp15 | chr4 | Fkbp15 | 2.636352587 | 1.458473435 | 1.445168179 | 7200 | 556 |
| chr4_6440989_6432680_-8309-Nsmaf | chr4 | Nsmaf | 0 | 0.364618359 | 0 | 8310 | 498 |
| chr4_72170770_72169092_-1678-Tle1 | chr4 | Tle1 | 0 | 0.182309179 | 0 | 1679 | 168 |
| chr4_72170770_72169122_-1648-Tle1 | chr4 | Tle1 | 0.405592706 | 0 | 0 | 1649 | 138 |
| chr4_74311750_74298441_+13309-Kdm4c | chr4 | Kdm4c | 0 | 0.182309179 | 0 | 13310 | 348 |
| chr4_74315649_74271194_+44455-Kdm4c | chr4 | Kdm4c | 0 | 0.182309179 | 0 | 44456 | 777 |
| chr4_74315649_74280897_+34752-Kdm4c | chr4 | Kdm4c | 0 | 0.546927538 | 0.180646022 | 34753 | 601 |
| chr4_81308426_81297287_-11139-Mpdz | chr4 | Mpdz | 0 | 0 | 0.180646022 | 11140 | 629 |
| chr4_81310319_81303593_-6726-Mpdz | chr4 | Mpdz | 0 | 0.182309179 | 0 | 6727 | 624 |
| chr4_81335874_81303593_-32281-Mpdz | chr4 | Mpdz | 0.202796353 | 0 | 0 | 32282 | 1451 |
| chr4_81386478_81377964_-8514-Mpdz | chr4 | Mpdz | 0 | 0.182309179 | 0 | 8515 | 1110 |
| chr4_82383661_82310303_-73358-na | chr4 | na | 0 | 0 | 0.090323011 | 73359 | 73359 |
| chr4_86217073_86156635_+60438-Adamtsl1 | chr4 | Adamtsl1 | 0 | 0.364618359 | 0 | 60439 | 597 |
| chr4_86388548_86249763_+138785-Adamtsl1 | chr4 | Adamtsl1 | 0 | 0.364618359 | 0 | 138786 | 2932 |
| chr4_86781711_86774238_+7473-Dennd4c | chr4 | Dennd4c | 0 | 0 | 0.180646022 | 7474 | 815 |
| chr4_86781711_86779835_+1876-na | chr4 | na | 0 | 0.182309179 | 0 | 1877 | 1877 |
| chr4_86829666_86819862_+9804-Dennd4c | chr4 | Dennd4c | 0.202796353 | 0 | 0 | 9805 | 1894 |
| chr4_87794011_87766440_-27571-Mllt3 | chr4 | Mllt3 | 0 | 0.182309179 | 0 | 27572 | 27572 |
| chr4_8806361_8751324_+55037-Chd7 | chr4 | Chd7 | 0 | 0.729236718 | 0 | 55038 | 2593 |
| chr4_88129052_88115931_+13121-Focad | chr4 | Focad | 0 | 0.182309179 | 0 | 13122 | 353 |
| chr4_88186082_88174613_+11469-Focad | chr4 | Focad | 0 | 0.182309179 | 0.090323011 | 11470 | 703 |
| chr4_88197016_88121270_+75746-Focad | chr4 | Focad | 0 | 0.182309179 | 0 | 75747 | 1487 |
| chr4_88232782_88121270_+111512-Focad | chr4 | Focad | 0.202796353 | 0 | 0 | 111513 | 1952 |
| chr4_88232782_88196759_+36023-Focad | chr4 | Focad | 0.405592706 | 0 | 0 | 36024 | 723 |
| chr4_88393648_88121270_+272378-Focad | chr4 | Focad | 0.202796353 | 0 | 0 | 272379 | 4400 |
| chr4_94590074_94582481_-7593-Plaa | chr4 | Plaa | 0 | 0 | 0.180646022 | 7594 | 1268 |
| chr4_9532065_9474898_-57167-Asph | chr4 | Asph | 0.405592706 | 0 | 0 | 57168 | 977 |
| chr4_9532065_9508627_-23438-Asph | chr4 | Asph | 1.216778117 | 0 | 0 | 23439 | 615 |
| chr4_9532065_9529886_-2179-Asph | chr4 | Asph | 0.405592706 | 0.364618359 | 0.361292045 | 2180 | 288 |
| chr4_9604629_9583812_-20817-Asph | chr4 | Asph | 0 | 0.182309179 | 0 | 20818 | 405 |
| chr4_9607871_9508627_-99244-Asph | chr4 | Asph | 0 | 0.182309179 | 0 | 99245 | 1268 |
| chr4_9610938_9484472_-126466-Asph | chr4 | Asph | 0 | 0.09115459 | 0 | 126467 | 1500 |
| chr4_9610938_9508627_-102311-Asph | chr4 | Asph | 0.304194529 | 0 | 0 | 102312 | 1364 |
| chr4_9639347_9635901_-3446-Asph | chr4 | Asph | 0 | 0 | 0.361292045 | 3447 | 264 |
| chr4_98041679_97956374_+85305-Nfia | chr4 | Nfia | 0 | 0.182309179 | 0 | 85306 | 516 |
| chr4_98065446_98063013_+2433-Nfia | chr4 | Nfia | 0.202796353 | 0 | 0 | 2434 | 345 |
| chr4_98688230_98674194_+14036-Inadl | chr4 | Inadl | 0 | 0 | 0.180646022 | 14037 | 804 |
| chr4_99089621_99055322_-34299-Dock7 | chr4 | Dock7 | 0.202796353 | 0 | 0 | 34300 | 1644 |
| chr4_99224550_99212577_+11973-Atg4c | chr4 | Atg4c | 0.405592706 | 0 | 0 | 11974 | 992 |
| chr4_99228634_99212577_+16057-Atg4c | chr4 | Atg4c | 0.202796353 | 0.182309179 | 0 | 16058 | 1071 |
| chr4_99235186_99212577_+22609-Atg4c | chr4 | Atg4c | 0 | 0.182309179 | 0.180646022 | 22610 | 1268 |
| chr4_99258703_99212577_+46126-Atg4c | chr4 | Atg4c | 0 | 0 | 0.541938067 | 46127 | 1575 |
| chr4_99753191_99742355_+10836-Alg6 | chr4 | Alg6 | 0 | 0.364618359 | 0 | 10837 | 897 |
| chr4_99753191_99744490_+8701-Alg6 | chr4 | Alg6 | 0 | 0 | 0.541938067 | 8702 | 646 |
| chr4_99901777_99831390_+70387-Efcab7 | chr4 | Efcab7 | 0 | 0.182309179 | 0 | 70388 | 70388 |
| chr4_99964865_99961456_+3409-Pgm2 | chr4 | Pgm2 | 0 | 0.182309179 | 0 | 3410 | 627 |
| chr4_99967213_99961456_+5757-Pgm2 | chr4 | Pgm2 | 0 | 0 | 0.180646022 | 5758 | 782 |
| chr4_99970102_99961456_+8646-Pgm2 | chr4 | Pgm2 | 0.811185411 | 0 | 0.361292045 | 8647 | 898 |
| chr4_99979000_99961456_+17544-Pgm2 | chr4 | Pgm2 | 0.202796353 | 0 | 0 | 17545 | 1034 |
| chr4_99984103_99967059_+17044-Pgm2 | chr4 | Pgm2 | 1.013981764 | 1.093855076 | 0.722584089 | 17045 | 726 |
| chr4_99984103_99969987_+14116-Pgm2 | chr4 | Pgm2 | 0 | 0.182309179 | 0 | 14117 | 571 |
| chr4_99984103_99978865_+5238-Pgm2 | chr4 | Pgm2 | 0 | 0.182309179 | 0 | 5239 | 455 |
| chr5_100408113_100392863_-15250-Sec31a | chr5 | Sec31a | 0 | 0.182309179 | 0 | 15251 | 1198 |
| chr5_100537468_100528539_+8929-Cops4 | chr5 | Cops4 | 0 | 0.182309179 | 0 | 8930 | 732 |
| chr5_103834459_103825980_+8479-Aff1 | chr5 | Aff1 | 0 | 0.182309179 | 0 | 8480 | 1246 |
| chr5_103876289_103870529_-5760-Klhl8 | chr5 | Klhl8 | 0 | 0.364618359 | 0 | 5761 | 992 |
| chr5_103876289_103874203_-2086-Klhl8 | chr5 | Klhl8 | 0.202796353 | 0 | 0.361292045 | 2087 | 736 |
| chr5_103886290_103885907_-383-Klhl8 | chr5 | Klhl8 | 1.41957447 | 2.005400973 | 0.180646022 | 384 | 384 |
| chr5_103890781_103885907_-4874-Klhl8 | chr5 | Klhl8 | 0.608389058 | 0 | 0 | 4875 | 451 |
| chr5_104489414_104466883_+22531-Pkd2 | chr5 | Pkd2 | 0 | 0 | 0.180646022 | 22532 | 1303 |
| chr5_105896603_105886187_+10416-Zfp326 | chr5 | Zfp326 | 0 | 0 | 0.180646022 | 10417 | 865 |
| chr5_106638635_106619540_-19095-Zfp644 | chr5 | Zfp644 | 0.405592706 | 0.364618359 | 0.361292045 | 19096 | 3855 |
| chr5_106666845_106618071_-48774-na | chr5 | na | 0.101398176 | 0 | 0 | 48775 | 48775 |
| chr5_106666845_106619540_-47305-na | chr5 | na | 0 | 0 | 0.270969033 | 47306 | 47306 |
| chr5_106666845_106634831_-32014-na | chr5 | na | 0.202796353 | 0 | 0 | 32015 | 32015 |
| chr5_106669840_106666785_-3055-Zfp644 | chr5 | Zfp644 | 0 | 0.182309179 | 0 | 3056 | 3056 |
| chr5_106679743_106634831_-44912-Zfp644 | chr5 | Zfp644 | 0.202796353 | 0 | 0.180646022 | 44913 | 3757 |
| chr5_106682013_106634831_-47182-Zfp644 | chr5 | Zfp644 | 0 | 0.182309179 | 0 | 47183 | 47183 |
| chr5_106690877_106634831_-56046-Zfp644 | chr5 | Zfp644 | 0.202796353 | 0 | 0 | 56047 | 3905 |
| chr5_107266350_107266194_-156-Tgfbr3 | chr5 | Tgfbr3 | 0 | 0.182309179 | 0 | 157 | 157 |
| chr5_107813606_107788199_-25407-Evi5 | chr5 | Evi5 | 0 | 0 | 0.180646022 | 25408 | 973 |
| chr5_108088089_108073307_+14782-Mtf2 | chr5 | Mtf2 | 0.202796353 | 0 | 0 | 14783 | 775 |
| chr5_108094248_108073307_+20941-Mtf2 | chr5 | Mtf2 | 0 | 0 | 0.180646022 | 20942 | 1064 |
| chr5_110153280_110151527_+1753-Chfr | chr5 | Chfr | 0.202796353 | 0 | 0 | 1754 | 1105 |
| chr5_110267265_110265512_-1753-Pgam5 | chr5 | Pgam5 | 0 | 0 | 0.180646022 | 1754 | 528 |
| chr5_110742060_110719087_-22973-Ep400 | chr5 | Ep400 | 0 | 0 | 0.180646022 | 22974 | 1963 |
| chr5_110756766_110719087_-37679-Ep400 | chr5 | Ep400 | 0.811185411 | 0 | 0 | 37680 | 3444 |
| chr5_110756766_110741970_-14796-Ep400 | chr5 | Ep400 | 0 | 0 | 0.541938067 | 14797 | 1572 |
| chr5_110756766_110741970_-14796-na | chr5 | na | 0 | 0.09115459 | 0 | 14797 | 14797 |
| chr5_111085169_111085022_+147-Ttc28 | chr5 | Ttc28 | 0.202796353 | 0 | 0 | 148 | 148 |
| chr5_111235641_111223110_+12531-Ttc28 | chr5 | Ttc28 | 0.202796353 | 0 | 0 | 12532 | 2491 |
| chr5_112309887_112303900_+5987-Tpst2 | chr5 | Tpst2 | 0 | 0.182309179 | 0 | 5988 | 1183 |
| chr5_112717031_112715381_-1650-Myo18b | chr5 | Myo18b | 0.202796353 | 0 | 0 | 1651 | 183 |
| chr5_112766578_112692096_-74482-Myo18b | chr5 | Myo18b | 0.202796353 | 0 | 0 | 74483 | 74483 |
| chr5_112785667_112782585_-3082-Myo18b | chr5 | Myo18b | 0 | 0.182309179 | 0 | 3083 | 216 |
| chr5_112803308_112782585_-20723-Myo18b | chr5 | Myo18b | 0.405592706 | 0.182309179 | 0 | 20724 | 1050 |
| chr5_112828086_112809662_-18424-Myo18b | chr5 | Myo18b | 0.202796353 | 0 | 0 | 18425 | 539 |
| chr5_112846444_112827977_-18467-Myo18b | chr5 | Myo18b | 1.41957447 | 1.093855076 | 0.541938067 | 18468 | 1364 |
| chr5_112846444_112830160_-16284-Myo18b | chr5 | Myo18b | 2.027963528 | 1.458473435 | 2.890336357 | 16285 | 1254 |
| chr5_112868392_112782585_-85807-Myo18b | chr5 | Myo18b | 0.101398176 | 0 | 0 | 85808 | 3495 |
| chr5_112868392_112858402_-9990-Myo18b | chr5 | Myo18b | 0 | 0 | 0.180646022 | 9991 | 652 |
| chr5_112879578_112757455_-122123-Myo18b | chr5 | Myo18b | 0.405592706 | 0 | 0 | 122124 | 6397 |
| chr5_112879578_112871421_-8157-Myo18b | chr5 | Myo18b | 0 | 0.182309179 | 0 | 8158 | 2110 |
| chr5_113193907_113175205_-18702-2900026A02Rik | chr5 | 2900026A02Rik | 0.202796353 | 0 | 0 | 18703 | 4630 |
| chr5_114166140_114165509_+631-Acacb | chr5 | Acacb | 0.202796353 | 0 | 0 | 632 | 632 |
| chr5_114202475_114201537_+938-Acacb | chr5 | Acacb | 0.202796353 | 0 | 0 | 939 | 939 |
| chr5_114204822_114201764_+3058-Acacb | chr5 | Acacb | 0 | 0 | 0.180646022 | 3059 | 3059 |
| chr5_114715901_114711253_+4648-Tchp | chr5 | Tchp | 0 | 0 | 0.180646022 | 4649 | 480 |
| chr5_114769750_114764347_-5403-Git2 | chr5 | Git2 | 0 | 0.182309179 | 0 | 5404 | 571 |
| chr5_115173224_115166868_-6356-Cabp1 | chr5 | Cabp1 | 0 | 0.364618359 | 0 | 6357 | 6357 |
| chr5_115247286_115244021_-3265-Rnf10 | chr5 | Rnf10 | 0 | 0 | 0.180646022 | 3266 | 477 |
| chr5_115480525_115479602_-923-Sirt4 | chr5 | Sirt4 | 0.202796353 | 0.364618359 | 0.180646022 | 924 | 536 |
| chr5_115599266_115594993_+4273-Gcn1l1 | chr5 | Gcn1l1 | 0 | 0.182309179 | 0 | 4274 | 975 |
| chr5_117914442_117900471_+13971-Nos1 | chr5 | Nos1 | 0 | 0 | 0.180646022 | 13972 | 1149 |
| chr5_117936612_117919396_+17216-Nos1 | chr5 | Nos1 | 0.202796353 | 0 | 0 | 17217 | 704 |
| chr5_117947568_117945816_+1752-Nos1 | chr5 | Nos1 | 0.202796353 | 0 | 0 | 1753 | 271 |
| chr5_118118357_118113612_-4745-na | chr5 | na | 0 | 0.09115459 | 0 | 4746 | 4746 |
| chr5_118127115_118126808_-307-na | chr5 | na | 0 | 0.182309179 | 0 | 308 | 308 |
| chr5_118680971_118661475_+19496-Med13l | chr5 | Med13l | 0.202796353 | 0 | 0 | 19497 | 19497 |
| chr5_121143154_121132255_-10899-Ptpn11 | chr5 | Ptpn11 | 0 | 0 | 0.180646022 | 10900 | 2248 |
| chr5_121168043_121149103_-18940-Ptpn11 | chr5 | Ptpn11 | 0 | 0.182309179 | 0 | 18941 | 1087 |
| chr5_121266405_121258699_+7706-Gm15800 | chr5 | Gm15800 | 0.202796353 | 0 | 0 | 7707 | 640 |
| chr5_121281944_121277675_+4269-Gm15800 | chr5 | Gm15800 | 0 | 0 | 0.180646022 | 4270 | 414 |
| chr5_121421403_121420463_+940-Naa25 | chr5 | Naa25 | 0.202796353 | 0 | 0 | 941 | 283 |
| chr5_121797785_121785759_+12026-Atxn2 | chr5 | Atxn2 | 0 | 0 | 0.361292045 | 12027 | 768 |
| chr5_121797791_121749079_+48712-Atxn2 | chr5 | Atxn2 | 0 | 0.182309179 | 0 | 48713 | 2143 |
| chr5_121797791_121785759_+12032-Atxn2 | chr5 | Atxn2 | 0.608389058 | 0 | 0 | 12033 | 774 |
| chr5_121924915_121898277_-26638-Cux2 | chr5 | Cux2 | 0.202796353 | 0 | 0 | 26639 | 238 |
| chr5_122363634_122362634_-1000-na | chr5 | na | 0 | 0.182309179 | 0 | 1001 | 1001 |
| chr5_122560745_122551060_-9685-Ift81 | chr5 | Ift81 | 0.202796353 | 0 | 0 | 9686 | 435 |
| chr5_122818004_122800451_-17553-Anapc5 | chr5 | Anapc5 | 0.202796353 | 0.364618359 | 0.722584089 | 17554 | 898 |
| chr5_122818004_122814549_-3455-Anapc5 | chr5 | Anapc5 | 0 | 0.364618359 | 1.083876134 | 3456 | 251 |
| chr5_122987806_122961410_-26396-Kdm2b | chr5 | Kdm2b | 0.202796353 | 0 | 0.361292045 | 26397 | 450 |
| chr5_123099953_123085936_+14017-Tmem120b | chr5 | Tmem120b | 0 | 0.729236718 | 0 | 14018 | 284 |
| chr5_123262154_123253685_+8469-Wdr66 | chr5 | Wdr66 | 0 | 0 | 0.361292045 | 8470 | 8470 |
| chr5_123302837_123253685_+49152-Wdr66 | chr5 | Wdr66 | 0 | 0 | 0.180646022 | 49153 | 49153 |
| chr5_123615335_123590793_-24542-Clip1 | chr5 | Clip1 | 0.202796353 | 0 | 0 | 24543 | 2666 |
| chr5_123617822_123617336_-486-Clip1 | chr5 | Clip1 | 0 | 0.182309179 | 0 | 487 | 382 |
| chr5_123631169_123590793_-40376-Clip1 | chr5 | Clip1 | 0.202796353 | 1.458473435 | 1.625814201 | 40377 | 4409 |
| chr5_123631169_123603599_-27570-Clip1 | chr5 | Clip1 | 0.405592706 | 0.182309179 | 0 | 27571 | 4356 |
| chr5_123631169_123617336_-13833-Clip1 | chr5 | Clip1 | 0.608389058 | 0 | 0 | 13834 | 1743 |
| chr5_123631169_123621796_-9373-Clip1 | chr5 | Clip1 | 0 | 0 | 0.180646022 | 9374 | 1361 |
| chr5_123642616_123617336_-25280-Clip1 | chr5 | Clip1 | 0.202796353 | 0 | 0 | 25281 | 2244 |
| chr5_124143647_124140524_-3123-Pitpnm2 | chr5 | Pitpnm2 | 0.202796353 | 0 | 0 | 3124 | 565 |
| chr5_124398509_124391922_-6587-Sbno1 | chr5 | Sbno1 | 0 | 0 | 0.180646022 | 6588 | 921 |
| chr5_124405729_124376098_-29631-Sbno1 | chr5 | Sbno1 | 0 | 0.182309179 | 0 | 29632 | 3288 |
| chr5_124410379_124410070_-309-Sbno1 | chr5 | Sbno1 | 0 | 0 | 0.541938067 | 310 | 310 |
| chr5_124413289_124407091_-6198-Sbno1 | chr5 | Sbno1 | 0 | 0.364618359 | 0 | 6199 | 616 |
| chr5_124414527_124376098_-38429-Sbno1 | chr5 | Sbno1 | 0.202796353 | 0.546927538 | 0.361292045 | 38430 | 4033 |
| chr5_124712359_124645945_+66414-Atp6v0a2 | chr5 | Atp6v0a2 | 0 | 0 | 0.361292045 | 66415 | 458 |
| chr5_124718005_124645945_+72060-Atp6v0a2 | chr5 | Atp6v0a2 | 0 | 0.182309179 | 0.180646022 | 72061 | 1324 |
| chr5_125515130_125503183_+11947-Aacs | chr5 | Aacs | 0 | 0 | 0.180646022 | 11948 | 1311 |
| chr5_125623352_125622461_+891-Tmem132b | chr5 | Tmem132b | 0.405592706 | 0 | 0 | 892 | 892 |
| chr5_125638328_125622461_+15867-Tmem132b | chr5 | Tmem132b | 0 | 0.182309179 | 0 | 15868 | 1039 |
| chr5_129748033_129739472_+8561-na | chr5 | na | 0 | 0 | 0.090323011 | 8562 | 8562 |
| chr5_129748033_129744705_+3328-Gbas | chr5 | Gbas | 0 | 0.364618359 | 0 | 3329 | 339 |
| chr5_129757252_129753226_+4026-Gbas | chr5 | Gbas | 0 | 0 | 0.361292045 | 4027 | 423 |
| chr5_129757252_129753226_+4026-na | chr5 | na | 0 | 0.182309179 | 0 | 4027 | 4027 |
| chr5_129847086_129739472_+107614-Cct6a | chr5 | Cct6a | 0.202796353 | 0 | 0 | 107615 | 107615 |
| chr5_129867025_129865917_-1108-Phkg1 | chr5 | Phkg1 | 0 | 0 | 0.180646022 | 1109 | 409 |
| chr5_129998553_129997780_-773-Gusb | chr5 | Gusb | 0 | 0 | 0.361292045 | 774 | 470 |
| chr5_130232040_130229449_+2591-Tmem248 | chr5 | Tmem248 | 0.202796353 | 0.182309179 | 0 | 2592 | 464 |
| chr5_134171976_134163624_-8352-Wbscr16 | chr5 | Wbscr16 | 0.202796353 | 0 | 0 | 8353 | 653 |
| chr5_134246611_134243507_-3104-Gtf2i | chr5 | Gtf2i | 0 | 0 | 0.361292045 | 3105 | 501 |
| chr5_134256008_134243507_-12501-Gtf2i | chr5 | Gtf2i | 0 | 0.182309179 | 0 | 12502 | 1134 |
| chr5_134274595_134260567_-14028-Gtf2i | chr5 | Gtf2i | 0 | 0.182309179 | 0 | 14029 | 615 |
| chr5_134287043_134265737_-21306-Gtf2i | chr5 | Gtf2i | 0 | 0 | 0.541938067 | 21307 | 750 |
| chr5_134287043_134282787_-4256-Gtf2i | chr5 | Gtf2i | 0 | 0.182309179 | 0 | 4257 | 312 |
| chr5_134295613_134274539_-21074-Gtf2i | chr5 | Gtf2i | 0.202796353 | 0 | 0.361292045 | 21075 | 885 |
| chr5_134306731_134274539_-32192-na | chr5 | na | 0 | 0 | 0.180646022 | 32193 | 32193 |
| chr5_134395800_134389131_-6669-Gtf2ird1 | chr5 | Gtf2ird1 | 0 | 0.182309179 | 0 | 6670 | 438 |
| chr5_135124017_135121506_+2511-Mlxipl | chr5 | Mlxipl | 6.692279643 | 5.83389374 | 5.961318737 | 2512 | 501 |
| chr5_135124017_135123650_+367-Mlxipl | chr5 | Mlxipl | 0.202796353 | 0 | 0 | 368 | 283 |
| chr5_135128697_135121506_+7191-Mlxipl | chr5 | Mlxipl | 0 | 0.273463769 | 0 | 7192 | 686 |
| chr5_135223218_135216590_+6628-Baz1b | chr5 | Baz1b | 0.202796353 | 0 | 0.180646022 | 6629 | 1978 |
| chr5_135226612_135209043_+17569-Baz1b | chr5 | Baz1b | 0.405592706 | 0 | 0 | 17570 | 2530 |
| chr5_135226612_135210741_+15871-Baz1b | chr5 | Baz1b | 0 | 0.182309179 | 0 | 15872 | 2408 |
| chr5_135238066_135198171_+39895-Baz1b | chr5 | Baz1b | 0.202796353 | 0 | 0 | 39896 | 3504 |
| chr5_135384166_135376022_-8144-Pom121 | chr5 | Pom121 | 0.405592706 | 0 | 0 | 8145 | 8145 |
| chr5_135451635_135440022_-11613-Hip1 | chr5 | Hip1 | 0.202796353 | 0 | 0 | 11614 | 478 |
| chr5_135455145_135444735_-10410-Hip1 | chr5 | Hip1 | 0.202796353 | 0 | 0 | 10411 | 419 |
| chr5_135857547_135855785_-1762-na | chr5 | na | 0.202796353 | 0 | 0 | 1763 | 1763 |
| chr5_135911651_135909074_-2577-na | chr5 | na | 0.405592706 | 0 | 0 | 2578 | 2578 |
| chr5_136095608_136089353_+6255-Rasa4 | chr5 | Rasa4 | 0.405592706 | 0 | 0 | 6256 | 445 |
| chr5_136311560_136304723_-6837-Cux1 | chr5 | Cux1 | 0.405592706 | 0 | 0 | 6838 | 1227 |
| chr5_136340137_136304723_-35414-Cux1 | chr5 | Cux1 | 0 | 0 | 0.180646022 | 35415 | 2519 |
| chr5_136392393_136360002_-32391-na | chr5 | na | 0 | 0.09115459 | 0 | 32392 | 32392 |
| chr5_137035609_137035170_+439-na | chr5 | na | 0.202796353 | 0 | 0 | 440 | 440 |
| chr5_139184719_139181417_+3302-Dnaaf5 | chr5 | Dnaaf5 | 0 | 0 | 0.180646022 | 3303 | 349 |
| chr5_142172603_142161775_+10828-Sdk1 | chr5 | Sdk1 | 0 | 0 | 0.180646022 | 10829 | 487 |
| chr5_142507088_142503913_-3175-Radil | chr5 | Radil | 0 | 0.182309179 | 0 | 3176 | 668 |
| chr5_143087592_143068317_-19275-Rnf216 | chr5 | Rnf216 | 0 | 0.182309179 | 0 | 19276 | 911 |
| chr5_143098505_143075727_-22778-Rnf216 | chr5 | Rnf216 | 0 | 0.182309179 | 0.180646022 | 22779 | 1857 |
| chr5_143098505_143089743_-8762-Rnf216 | chr5 | Rnf216 | 0 | 0.182309179 | 0 | 8763 | 1145 |
| chr5_143265133_143262793_-2340-Zfp316 | chr5 | Zfp316 | 0.202796353 | 0 | 0 | 2341 | 850 |
| chr5_143484352_143478414_+5938-Daglb | chr5 | Daglb | 0.202796353 | 0 | 0 | 5939 | 378 |
| chr5_143886961_143871863_+15098-Eif2ak1 | chr5 | Eif2ak1 | 0 | 0 | 0.180646022 | 15099 | 1107 |
| chr5_143931122_143922016_+9106-Pms2 | chr5 | Pms2 | 0 | 0 | 0.180646022 | 9107 | 1671 |
| chr5_143942028_143922016_+20012-Pms2 | chr5 | Pms2 | 0 | 0.182309179 | 0 | 20013 | 2030 |
| chr5_143942028_143928942_+13086-Pms2 | chr5 | Pms2 | 0 | 0 | 0.180646022 | 13087 | 768 |
| chr5_143993259_143987199_-6060-Ccz1 | chr5 | Ccz1 | 0 | 0.182309179 | 0 | 6061 | 6061 |
| chr5_144166419_144143526_+22893-Lmtk2 | chr5 | Lmtk2 | 0 | 0 | 0.361292045 | 22894 | 622 |
| chr5_144176477_144143526_+32951-Lmtk2 | chr5 | Lmtk2 | 0.405592706 | 0 | 0 | 32952 | 3641 |
| chr5_144176477_144164606_+11871-Lmtk2 | chr5 | Lmtk2 | 0 | 0 | 0.361292045 | 11872 | 3226 |
| chr5_144902578_144894474_-8104-Smurf1 | chr5 | Smurf1 | 0 | 0 | 0.180646022 | 8105 | 751 |
| chr5_147467097_147453252_+13845-Pan3 | chr5 | Pan3 | 0 | 0 | 0.361292045 | 13846 | 300 |
| chr5_147488258_147450653_+37605-Pan3 | chr5 | Pan3 | 0 | 0.364618359 | 0 | 37606 | 570 |
| chr5_147531307_147519505_+11802-Pan3 | chr5 | Pan3 | 0.202796353 | 0.546927538 | 0.361292045 | 11803 | 1049 |
| chr5_148921412_148888879_-32533-Katnal1 | chr5 | Katnal1 | 0 | 0 | 0.180646022 | 32534 | 1155 |
| chr5_149739601_149726754_+12847-B3glct | chr5 | B3glct | 0 | 0.729236718 | 0 | 12848 | 503 |
| chr5_150378980_150358799_+20181-Fry | chr5 | Fry | 0.202796353 | 0 | 0 | 20182 | 766 |
| chr5_150395867_150380798_+15069-Fry | chr5 | Fry | 0.202796353 | 0 | 0 | 15070 | 1345 |
| chr5_150405421_150380798_+24623-Fry | chr5 | Fry | 0.202796353 | 0 | 0 | 24624 | 2367 |
| chr5_150437332_150433517_+3815-Fry | chr5 | Fry | 0.405592706 | 0.182309179 | 0 | 3816 | 915 |
| chr5_150471610_150466244_+5366-Fry | chr5 | Fry | 0 | 0.364618359 | 0 | 5367 | 717 |
| chr5_150471610_150469677_+1933-Fry | chr5 | Fry | 0.202796353 | 0 | 0 | 1934 | 357 |
| chr5_150560823_150550832_+9991-Brca2 | chr5 | Brca2 | 0 | 0 | 0.180646022 | 9992 | 1815 |
| chr5_150567768_150547941_+19827-Brca2 | chr5 | Brca2 | 0.202796353 | 0 | 0 | 19828 | 2636 |
| chr5_150608440_150604177_-4263-N4bp2l2 | chr5 | N4bp2l2 | 1.216778117 | 1.640782614 | 1.806460223 | 4264 | 1812 |
| chr5_150643315_150604177_-39138-N4bp2l2 | chr5 | N4bp2l2 | 0.202796353 | 0 | 0.361292045 | 39139 | 1959 |
| chr5_150783142_150728894_+54248-Pds5b | chr5 | Pds5b | 0.202796353 | 0 | 0 | 54249 | 2307 |
| chr5_16267475_16212876_+54599-Cacna2d1 | chr5 | Cacna2d1 | 0 | 0 | 0.180646022 | 54600 | 483 |
| chr5_16322595_16299929_+22666-Cacna2d1 | chr5 | Cacna2d1 | 0 | 0.182309179 | 0 | 22667 | 768 |
| chr5_20774275_20764214_-10061-Phtf2 | chr5 | Phtf2 | 0 | 0 | 0.180646022 | 10062 | 744 |
| chr5_20782491_20765758_-16733-Phtf2 | chr5 | Phtf2 | 0.202796353 | 0 | 0 | 16734 | 996 |
| chr5_20794481_20789629_-4852-Phtf2 | chr5 | Phtf2 | 0 | 0.546927538 | 0.180646022 | 4853 | 343 |
| chr5_20920162_20919525_-637-Rsbn1l | chr5 | Rsbn1l | 0.405592706 | 0 | 0 | 638 | 638 |
| chr5_23450336_23450152_+184-Kmt2e | chr5 | Kmt2e | 0.405592706 | 0 | 0 | 185 | 185 |
| chr5_23464932_23450152_+14780-Kmt2e | chr5 | Kmt2e | 0 | 0.182309179 | 0 | 14781 | 530 |
| chr5_23547067_23507058_-40009-Srpk2 | chr5 | Srpk2 | 0 | 0 | 0.180646022 | 40010 | 2321 |
| chr5_23576385_23546980_-29405-Srpk2 | chr5 | Srpk2 | 0 | 0.546927538 | 0 | 29406 | 29406 |
| chr5_23811034_23800595_+10439-Rint1 | chr5 | Rint1 | 0 | 0 | 0.361292045 | 10440 | 1198 |
| chr5_23991837_23979488_-12349-Fam126a | chr5 | Fam126a | 0 | 0.182309179 | 0 | 12350 | 940 |
| chr5_24141293_24126822_+14471-Klhl7 | chr5 | Klhl7 | 0 | 0 | 0.180646022 | 14472 | 816 |
| chr5_24483433_24476378_+7055-Agap3 | chr5 | Agap3 | 0 | 0.364618359 | 0 | 7056 | 2191 |
| chr5_25281400_25275900_-5500-Kmt2c | chr5 | Kmt2c | 0 | 0 | 1.083876134 | 5501 | 360 |
| chr5_25285183_25281661_-3522-Kmt2c | chr5 | Kmt2c | 0.202796353 | 0 | 0 | 3523 | 1397 |
| chr5_25285183_25281673_-3510-Kmt2c | chr5 | Kmt2c | 0 | 0 | 0.180646022 | 3511 | 1385 |
| chr5_25287845_25281232_-6613-Kmt2c | chr5 | Kmt2c | 0 | 0.546927538 | 0 | 6614 | 1814 |
| chr5_25338581_25298870_-39711-Kmt2c | chr5 | Kmt2c | 0 | 0.182309179 | 0 | 39712 | 7413 |
| chr5_25353408_25347361_-6047-Kmt2c | chr5 | Kmt2c | 0.202796353 | 0.364618359 | 0.180646022 | 6048 | 638 |
| chr5_25366196_25338413_-27783-Kmt2c | chr5 | Kmt2c | 0.202796353 | 0 | 0 | 27784 | 1729 |
| chr5_25366196_25347361_-18835-Kmt2c | chr5 | Kmt2c | 0 | 0 | 0.180646022 | 18836 | 1429 |
| chr5_25395551_25372766_-22785-Kmt2c | chr5 | Kmt2c | 0 | 0 | 0.361292045 | 22786 | 1668 |
| chr5_25812027_25798446_+13581-Actr3b | chr5 | Actr3b | 0.202796353 | 0.364618359 | 0 | 13582 | 292 |
| chr5_25825215_25809785_+15430-Actr3b | chr5 | Actr3b | 0 | 0.182309179 | 0 | 15431 | 440 |
| chr5_25832478_25829526_+2952-na | chr5 | na | 0 | 0.182309179 | 0 | 2953 | 2953 |
| chr5_27788777_27772004_-16773-Paxip1 | chr5 | Paxip1 | 0 | 0 | 0.180646022 | 16774 | 985 |
| chr5_28342514_28329382_+13132-Rbm33 | chr5 | Rbm33 | 0.202796353 | 0 | 0 | 13133 | 13133 |
| chr5_28342514_28331111_+11403-Rbm33 | chr5 | Rbm33 | 0 | 0.364618359 | 0 | 11404 | 638 |
| chr5_28362234_28358274_+3960-Rbm33 | chr5 | Rbm33 | 0 | 0 | 0.180646022 | 3961 | 498 |
| chr5_28394670_28331111_+63559-Rbm33 | chr5 | Rbm33 | 0 | 0.182309179 | 0 | 63560 | 3116 |
| chr5_28394670_28358274_+36396-Rbm33 | chr5 | Rbm33 | 0 | 0 | 0.541938067 | 36397 | 2306 |
| chr5_29323905_29287391_-36514-Lmbr1 | chr5 | Lmbr1 | 0 | 0.182309179 | 0 | 36515 | 438 |
| chr5_29346906_29287391_-59515-Lmbr1 | chr5 | Lmbr1 | 0.202796353 | 0 | 0 | 59516 | 578 |
| chr5_29346906_29346145_-761-Lmbr1 | chr5 | Lmbr1 | 0 | 0.182309179 | 0 | 762 | 762 |
| chr5_29365206_29287391_-77815-Lmbr1 | chr5 | Lmbr1 | 0.202796353 | 0 | 0 | 77816 | 977 |
| chr5_29365206_29323802_-41404-Lmbr1 | chr5 | Lmbr1 | 0 | 0.182309179 | 0 | 41405 | 643 |
| chr5_30145368_30134058_-11310-Hadha | chr5 | Hadha | 0 | 0.182309179 | 0 | 11311 | 738 |
| chr5_31673445_31659907_-13538-Rbks | chr5 | Rbks | 0.202796353 | 0 | 0 | 13539 | 425 |
| chr5_31831159_31766644_+64515-Bre | chr5 | Bre | 0.202796353 | 0 | 0 | 64516 | 64516 |
| chr5_32153959_32146825_+7134-Fosl2 | chr5 | Fosl2 | 0.202796353 | 0 | 0 | 7135 | 1649 |
| chr5_33267037_33259138_-7899-Ctbp1 | chr5 | Ctbp1 | 0 | 0 | 0.180646022 | 7900 | 507 |
| chr5_33269634_33259138_-10496-Ctbp1 | chr5 | Ctbp1 | 0 | 0 | 0.180646022 | 10497 | 700 |
| chr5_33614834_33607464_-7370-Fam53a | chr5 | Fam53a | 0.202796353 | 0.364618359 | 0 | 7371 | 1051 |
| chr5_33614834_33610432_-4402-Fam53a | chr5 | Fam53a | 0 | 0 | 0.180646022 | 4403 | 287 |
| chr5_34440590_34419998_+20592-Fam193a | chr5 | Fam193a | 0.405592706 | 0.364618359 | 0 | 20593 | 1268 |
| chr5_34444913_34410581_+34332-Fam193a | chr5 | Fam193a | 0 | 0 | 0.180646022 | 34333 | 2097 |
| chr5_34444913_34431132_+13781-Fam193a | chr5 | Fam193a | 0.202796353 | 0 | 0 | 13782 | 1293 |
| chr5_34444913_34436451_+8462-Fam193a | chr5 | Fam193a | 0 | 0.182309179 | 0 | 8463 | 1168 |
| chr5_34466447_34431132_+35315-Fam193a | chr5 | Fam193a | 0 | 0.182309179 | 0.180646022 | 35316 | 3355 |
| chr5_34475807_34463974_+11833-Fam193a | chr5 | Fam193a | 0 | 0.364618359 | 0 | 11834 | 1187 |
| chr5_34614360_34605828_+8532-Add1 | chr5 | Add1 | 0 | 0 | 0.180646022 | 8533 | 803 |
| chr5_34628513_34619350_+9163-Add1 | chr5 | Add1 | 0 | 0 | 0.180646022 | 9164 | 535 |
| chr5_34731570_34730005_+1565-Grk4 | chr5 | Grk4 | 0.202796353 | 0 | 0 | 1566 | 128 |
| chr5_34886600_34878803_+7797-Htt | chr5 | Htt | 0 | 0.182309179 | 0 | 7798 | 896 |
| chr5_3577344_3575623_+1721-1700109H08Rik | chr5 | 1700109H08Rik | 0 | 0 | 0.361292045 | 1722 | 261 |
| chr5_35852644_35852432_-212-na | chr5 | na | 0.101398176 | 0 | 0 | 213 | 213 |
| chr5_3615207_3603232_+11975-Pex1 | chr5 | Pex1 | 0 | 0 | 0.361292045 | 11976 | 1677 |
| chr5_3618913_3603232_+15681-Pex1 | chr5 | Pex1 | 0.202796353 | 0 | 0 | 15682 | 1774 |
| chr5_36514119_36505025_-9094-Tbc1d14 | chr5 | Tbc1d14 | 0 | 0.182309179 | 0 | 9095 | 487 |
| chr5_36572037_36571296_-741-Tbc1d14 | chr5 | Tbc1d14 | 0.202796353 | 0.729236718 | 0.361292045 | 742 | 742 |
| chr5_36577772_36571296_-6476-Tbc1d14 | chr5 | Tbc1d14 | 0 | 0 | 0.090323011 | 6477 | 6477 |
| chr5_4044070_4038529_+5541-Akap9 | chr5 | Akap9 | 0 | 0.182309179 | 0 | 5542 | 435 |
| chr5_4046941_4028374_+18567-Akap9 | chr5 | Akap9 | 0 | 0.182309179 | 0.180646022 | 18568 | 2427 |
| chr5_4072700_4067740_+4960-Akap9 | chr5 | Akap9 | 0.202796353 | 0.364618359 | 0 | 4961 | 703 |
| chr5_4072700_4067745_+4955-Akap9 | chr5 | Akap9 | 0.202796353 | 0 | 0 | 4956 | 698 |
| chr5_43191404_43165919_-25485-Gm7854 | chr5 | Gm7854 | 0 | 0.364618359 | 0 | 25486 | 479 |
| chr5_50013464_49990125_-23339-Adgra3 | chr5 | Adgra3 | 0.202796353 | 0 | 0 | 23340 | 1204 |
| chr5_51548691_51546267_-2424-Ppargc1a | chr5 | Ppargc1a | 0 | 0 | 0.270969033 | 2425 | 2425 |
| chr5_51548691_51548512_-179-Ppargc1a | chr5 | Ppargc1a | 0 | 0.364618359 | 0 | 180 | 180 |
| chr5_5249228_5227164_-22064-Cdk14 | chr5 | Cdk14 | 0 | 0 | 0.722584089 | 22065 | 341 |
| chr5_52819265_52804379_+14886-Zcchc4 | chr5 | Zcchc4 | 0 | 0.182309179 | 0 | 14887 | 805 |
| chr5_53174869_53121750_-53119-Sel1l3 | chr5 | Sel1l3 | 0 | 0.182309179 | 0 | 53120 | 1798 |
| chr5_53188082_53145435_-42647-Sel1l3 | chr5 | Sel1l3 | 0 | 0 | 0.180646022 | 42648 | 1343 |
| chr5_53188082_53170357_-17725-Sel1l3 | chr5 | Sel1l3 | 1.216778117 | 0.182309179 | 0.722584089 | 17726 | 831 |
| chr5_53188082_53181722_-6360-Sel1l3 | chr5 | Sel1l3 | 0.202796353 | 0.546927538 | 0 | 6361 | 424 |
| chr5_53200471_53170357_-30114-Sel1l3 | chr5 | Sel1l3 | 0.202796353 | 0 | 0 | 30115 | 1402 |
| chr5_53200471_53184764_-15707-Sel1l3 | chr5 | Sel1l3 | 0.202796353 | 0 | 0 | 15708 | 936 |
| chr5_53200471_53199901_-570-Sel1l3 | chr5 | Sel1l3 | 0.608389058 | 0.546927538 | 1.264522156 | 571 | 571 |
| chr5_54111110_54102575_+8535-Stim2 | chr5 | Stim2 | 0 | 0.182309179 | 0 | 8536 | 1116 |
| chr5_5489595_5450608_-38987-1700015F17Rik | chr5 | 1700015F17Rik | 0 | 0.364618359 | 0 | 38988 | 38988 |
| chr5_63898730_63876450_+22280-0610040J01Rik | chr5 | 0610040J01Rik | 0.202796353 | 0 | 0 | 22281 | 1022 |
| chr5_64294295_64260353_+33942-Tbc1d1 | chr5 | Tbc1d1 | 0 | 0.182309179 | 0 | 33943 | 33943 |
| chr5_65418608_65416825_-1783-Ugdh | chr5 | Ugdh | 0 | 0.182309179 | 0.180646022 | 1784 | 468 |
| chr5_65803567_65790018_+13549-N4bp2 | chr5 | N4bp2 | 0 | 0 | 0.180646022 | 13550 | 1591 |
| chr5_65814870_65788053_+26817-N4bp2 | chr5 | N4bp2 | 0 | 0 | 0.180646022 | 26818 | 4473 |
| chr5_66392335_66362718_-29617-Apbb2 | chr5 | Apbb2 | 0 | 0.182309179 | 0 | 29618 | 485 |
| chr5_66400281_66362718_-37563-Apbb2 | chr5 | Apbb2 | 0 | 0.364618359 | 0 | 37564 | 691 |
| chr5_66986537_66983837_+2700-Limch1 | chr5 | Limch1 | 1.013981764 | 2.734637691 | 1.264522156 | 2701 | 2701 |
| chr5_67776198_67702980_-73218-Atp8a1 | chr5 | Atp8a1 | 0 | 0.546927538 | 0 | 73219 | 1627 |
| chr5_67776198_67762516_-13682-Atp8a1 | chr5 | Atp8a1 | 1.013981764 | 0 | 0 | 13683 | 682 |
| chr5_67814723_67805657_-9066-Atp8a1 | chr5 | Atp8a1 | 0.202796353 | 0.182309179 | 0 | 9067 | 360 |
| chr5_69560515_69558185_+2330-Guf1 | chr5 | Guf1 | 0 | 0.364618359 | 0 | 2331 | 504 |
| chr5_71642326_71623855_-18471-Gabra4 | chr5 | Gabra4 | 0 | 0 | 0.180646022 | 18472 | 929 |
| chr5_73195113_73188360_-6753-Fryl | chr5 | Fryl | 0.202796353 | 0 | 0 | 6754 | 6754 |
| chr5_74547175_74545412_+1763-Fip1l1 | chr5 | Fip1l1 | 0 | 0 | 0.361292045 | 1764 | 373 |
| chr5_74572585_74536801_+35784-Fip1l1 | chr5 | Fip1l1 | 0 | 0.182309179 | 0 | 35785 | 1089 |
| chr5_74685872_74685396_-476-Lnx1 | chr5 | Lnx1 | 0.202796353 | 0 | 0 | 477 | 477 |
| chr5_76204816_76199345_+5471-Tmem165 | chr5 | Tmem165 | 0 | 0.182309179 | 0 | 5472 | 582 |
| chr5_76274043_76262675_-11368-Clock | chr5 | Clock | 0.101398176 | 0 | 0 | 11369 | 476 |
| chr5_76546131_76542051_+4080-Exoc1 | chr5 | Exoc1 | 0 | 0 | 0.180646022 | 4081 | 4081 |
| chr5_76559825_76549452_+10373-Exoc1 | chr5 | Exoc1 | 0.405592706 | 0 | 0 | 10374 | 573 |
| chr5_76996689_76990273_+6416-Srp72 | chr5 | Srp72 | 0 | 0 | 0.180646022 | 6417 | 592 |
| chr5_8160799_8149208_-11591-Adam22 | chr5 | Adam22 | 0.202796353 | 0 | 0 | 11592 | 280 |
| chr5_8907399_8899568_+7831-Abcb4 | chr5 | Abcb4 | 0 | 0.182309179 | 0 | 7832 | 401 |
| chr5_89084718_88934749_+149969-Slc4a4 | chr5 | Slc4a4 | 0 | 0.182309179 | 0 | 149970 | 904 |
| chr5_8910743_8909102_+1641-Abcb4 | chr5 | Abcb4 | 0 | 0.729236718 | 0.180646022 | 1642 | 297 |
| chr5_89149433_89084642_+64791-Slc4a4 | chr5 | Slc4a4 | 0.202796353 | 0 | 0 | 64792 | 901 |
| chr5_89179844_89046213_+133631-Slc4a4 | chr5 | Slc4a4 | 0.202796353 | 0 | 0 | 133632 | 1712 |
| chr5_90298134_90250375_-47759-Ankrd17 | chr5 | Ankrd17 | 0 | 0.182309179 | 0 | 47760 | 4475 |
| chr5_90304065_90278243_-25822-Ankrd17 | chr5 | Ankrd17 | 0 | 0.182309179 | 0 | 25823 | 2841 |
| chr5_90304065_90298540_-5525-Ankrd17 | chr5 | Ankrd17 | 0.405592706 | 0 | 0 | 5526 | 459 |
| chr5_96098313_96091584_-6729-Cnot6l | chr5 | Cnot6l | 0 | 0.182309179 | 0 | 6730 | 382 |
| chr5_96134132_96106216_-27916-Cnot6l | chr5 | Cnot6l | 0 | 0 | 0.180646022 | 27917 | 485 |
| chr5_97980034_97960610_-19424-Antxr2 | chr5 | Antxr2 | 0 | 0 | 0.361292045 | 19425 | 405 |
| chr6_100637122_100630883_-6239-Shq1 | chr6 | Shq1 | 0 | 0.182309179 | 0 | 6240 | 333 |
| chr6_100655371_100630883_-24488-Shq1 | chr6 | Shq1 | 0.608389058 | 0 | 0 | 24489 | 574 |
| chr6_101252578_101249147_-3431-na | chr6 | na | 0 | 0 | 0.090323011 | 3432 | 3432 |
| chr6_101252578_101251920_-658-na | chr6 | na | 0 | 0.182309179 | 0 | 659 | 659 |
| chr6_101354501_101277532_-76969-Pdzrn3 | chr6 | Pdzrn3 | 0.202796353 | 0 | 0 | 76970 | 76970 |
| chr6_113398884_113393103_+5781-Ttll3 | chr6 | Ttll3 | 0.202796353 | 0 | 0 | 5782 | 603 |
| chr6_114890806_114862201_-28605-Vgll4 | chr6 | Vgll4 | 0 | 0.182309179 | 0 | 28606 | 746 |
| chr6_115614685_113615776_+1998909-Atg7 | chr6 | Atg7 | 0 | 0.182309179 | 0 | 1998910 | 1998910 |
| chr6_115635080_115631215_-3865-Raf1 | chr6 | Raf1 | 0 | 0.364618359 | 0 | 3866 | 514 |
| chr6_115644642_115631215_-13427-Raf1 | chr6 | Raf1 | 0 | 0.182309179 | 0 | 13428 | 860 |
| chr6_115644642_115632897_-11745-Raf1 | chr6 | Raf1 | 0.202796353 | 0 | 0.180646022 | 11746 | 706 |
| chr6_115669424_115637599_-31825-na | chr6 | na | 0 | 0.09115459 | 0.090323011 | 31826 | 31826 |
| chr6_115877551_115875244_+2307-Ift122 | chr6 | Ift122 | 0 | 0.364618359 | 0 | 2308 | 156 |
| chr6_116107516_116042871_-64645-Tmcc1 | chr6 | Tmcc1 | 0.202796353 | 0 | 0 | 64646 | 1140 |
| chr6_116134457_116097914_-36543-Tmcc1 | chr6 | Tmcc1 | 0.202796353 | 0 | 0 | 36544 | 36544 |
| chr6_116138250_116133767_-4483-Tmcc1 | chr6 | Tmcc1 | 0.811185411 | 0 | 0 | 4484 | 747 |
| chr6_118398155_118388756_-9399-Bms1 | chr6 | Bms1 | 0.202796353 | 0 | 0.180646022 | 9400 | 1290 |
| chr6_119279526_119277062_+2464-Cacna2d4 | chr6 | Cacna2d4 | 0.202796353 | 0 | 0 | 2465 | 2465 |
| chr6_119282250_119267807_+14443-Cacna2d4 | chr6 | Cacna2d4 | 0.405592706 | 0 | 0 | 14444 | 1097 |
| chr6_11962789_11959929_+2860-Phf14 | chr6 | Phf14 | 0.405592706 | 0 | 0 | 2861 | 2861 |
| chr6_119761311_119694571_-66740-Erc1 | chr6 | Erc1 | 0 | 0.182309179 | 0 | 66741 | 1223 |
| chr6_119825209_119694571_-130638-Erc1 | chr6 | Erc1 | 0 | 0.182309179 | 0 | 130639 | 2947 |
| chr6_119825209_119713680_-111529-Erc1 | chr6 | Erc1 | 0 | 0 | 0.180646022 | 111530 | 2786 |
| chr6_119825209_119749838_-75371-Erc1 | chr6 | Erc1 | 0 | 0.182309179 | 0 | 75372 | 2312 |
| chr6_119825209_119796907_-28302-Erc1 | chr6 | Erc1 | 0.202796353 | 0.729236718 | 0 | 28303 | 1241 |
| chr6_119825819_119713680_-112139-Erc1 | chr6 | Erc1 | 0.405592706 | 0 | 0 | 112140 | 2925 |
| chr6_119825819_119722173_-103646-Erc1 | chr6 | Erc1 | 0.202796353 | 0 | 0 | 103647 | 2793 |
| chr6_119825819_119824386_-1433-Erc1 | chr6 | Erc1 | 0 | 0.911545897 | 0.180646022 | 1434 | 963 |
| chr6_119840161_119743288_-96873-Erc1 | chr6 | Erc1 | 0.202796353 | 0 | 0 | 96874 | 2595 |
| chr6_119914237_119911048_+3189-Rad52 | chr6 | Rad52 | 0.304194529 | 0.273463769 | 0.361292045 | 3190 | 537 |
| chr6_119916042_119911048_+4994-Rad52 | chr6 | Rad52 | 0.101398176 | 0 | 0 | 4995 | 732 |
| chr6_11997194_11986342_+10852-Phf14 | chr6 | Phf14 | 0.405592706 | 0 | 0 | 10853 | 1026 |
| chr6_119992560_119990092_-2468-Wnk1 | chr6 | Wnk1 | 0 | 0.182309179 | 0 | 2469 | 379 |
| chr6_12047944_11961550_+86394-Phf14 | chr6 | Phf14 | 0 | 0 | 0.180646022 | 86395 | 1593 |
| chr6_122833876_122828157_+5719-Foxj2 | chr6 | Foxj2 | 0.202796353 | 0 | 0 | 5720 | 828 |
| chr6_124636166_124542188_-93978-C1s2 | chr6 | C1s2 | 0 | 0.182309179 | 0 | 93979 | 93979 |
| chr6_124636166_124542193_-93973-C1s2 | chr6 | C1s2 | 1.825167175 | 1.093855076 | 1.264522156 | 93974 | 93974 |
| chr6_127478129_127468032_+10097-Parp11 | chr6 | Parp11 | 0 | 0 | 0.541938067 | 10098 | 651 |
| chr6_127491890_127468032_+23858-Parp11 | chr6 | Parp11 | 0 | 0.182309179 | 0 | 23859 | 1275 |
| chr6_129184313_129180633_+3680-Clec2d | chr6 | Clec2d | 0 | 0.364618359 | 0 | 3681 | 420 |
| chr6_131376180_131367765_-8415-Ybx3 | chr6 | Ybx3 | 0.202796353 | 0 | 0.361292045 | 8416 | 570 |
| chr6_131384566_131379358_-5208-Ybx3 | chr6 | Ybx3 | 0 | 0.364618359 | 0 | 5209 | 311 |
| chr6_134066465_134062064_+4401-na | chr6 | na | 0 | 0 | 0.180646022 | 4402 | 4402 |
| chr6_134542045_134541652_-393-Lrp6 | chr6 | Lrp6 | 0.202796353 | 0.364618359 | 0 | 394 | 394 |
| chr6_136566785_136559764_+7021-Atf7ip | chr6 | Atf7ip | 0.405592706 | 0.182309179 | 0.180646022 | 7022 | 2047 |
| chr6_136571538_136559764_+11774-Atf7ip | chr6 | Atf7ip | 0 | 0 | 0.361292045 | 11775 | 2113 |
| chr6_136582466_136559764_+22702-Atf7ip | chr6 | Atf7ip | 0 | 0 | 0.361292045 | 22703 | 2276 |
| chr6_140528633_140524865_+3768-Plekha5 | chr6 | Plekha5 | 0 | 0.364618359 | 0 | 3769 | 383 |
| chr6_140556070_140543720_+12350-Plekha5 | chr6 | Plekha5 | 0.405592706 | 0.546927538 | 0.361292045 | 12351 | 1135 |
| chr6_140568881_140543720_+25161-na | chr6 | na | 0 | 0.546927538 | 0.270969033 | 25162 | 25162 |
| chr6_140570186_140543720_+26466-Plekha5 | chr6 | Plekha5 | 0 | 0 | 0.180646022 | 26467 | 1450 |
| chr6_140580115_140543720_+36395-Plekha5 | chr6 | Plekha5 | 0 | 0 | 0.180646022 | 36396 | 36396 |
| chr6_140820889_140764278_+56611-Gm30215 | chr6 | Gm30215 | 0 | 0 | 0.180646022 | 56612 | 56612 |
| chr6_142644355_142628423_-15932-Abcc9 | chr6 | Abcc9 | 0 | 0 | 0.180646022 | 15933 | 672 |
| chr6_142687441_142639192_-48249-Abcc9 | chr6 | Abcc9 | 0 | 0.364618359 | 0 | 48250 | 2187 |
| chr6_142689130_142652527_-36603-Abcc9 | chr6 | Abcc9 | 0.202796353 | 0 | 0 | 36604 | 1783 |
| chr6_148415783_148411038_-4745-Tmtc1 | chr6 | Tmtc1 | 0.202796353 | 0 | 0 | 4746 | 455 |
| chr6_148425984_148411038_-14946-Tmtc1 | chr6 | Tmtc1 | 1.622370823 | 0.364618359 | 0.180646022 | 14947 | 633 |
| chr6_17492437_17487797_+4640-Met | chr6 | Met | 0 | 0.364618359 | 0 | 4641 | 1260 |
| chr6_17513541_17491227_+22314-Met | chr6 | Met | 0 | 0 | 0.180646022 | 22315 | 1403 |
| chr6_17527249_17487797_+39452-Met | chr6 | Met | 0 | 0.182309179 | 0 | 39453 | 1761 |
| chr6_17563719_17553239_+10480-Met | chr6 | Met | 0 | 0 | 0.180646022 | 10481 | 770 |
| chr6_17904969_17852258_+52711-St7 | chr6 | St7 | 0 | 0 | 0.361292045 | 52712 | 441 |
| chr6_21969435_21952129_+17306-Ing3 | chr6 | Ing3 | 0 | 0 | 0.180646022 | 17307 | 614 |
| chr6_22017086_22016903_+183-Cped1 | chr6 | Cped1 | 0.405592706 | 0 | 0 | 184 | 184 |
| chr6_22088857_22016903_+71954-Cped1 | chr6 | Cped1 | 0.202796353 | 0 | 0 | 71955 | 669 |
| chr6_22145687_22119462_+26225-Cped1 | chr6 | Cped1 | 0.202796353 | 0 | 0 | 26226 | 1125 |
| chr6_22145687_22132229_+13458-Cped1 | chr6 | Cped1 | 4.258723409 | 4.740038664 | 1.264522156 | 13459 | 636 |
| chr6_22145687_22143944_+1743-Cped1 | chr6 | Cped1 | 0 | 0.182309179 | 0 | 1744 | 356 |
| chr6_22222540_22132229_+90311-Cped1 | chr6 | Cped1 | 0 | 0.182309179 | 0.361292045 | 90312 | 891 |
| chr6_22237742_22233445_+4297-Cped1 | chr6 | Cped1 | 0 | 0.182309179 | 0 | 4298 | 558 |
| chr6_24634658_24624236_-10422-Wasl | chr6 | Wasl | 0 | 0.182309179 | 0 | 10423 | 574 |
| chr6_28545598_28522983_+22615-Snd1 | chr6 | Snd1 | 0 | 0.182309179 | 0 | 22616 | 22616 |
| chr6_28545598_28526053_+19545-Snd1 | chr6 | Snd1 | 0 | 0 | 0.180646022 | 19546 | 724 |
| chr6_29139626_29131267_-8359-Rbm28 | chr6 | Rbm28 | 0 | 0.182309179 | 0 | 8360 | 659 |
| chr6_29451793_29450814_+979-Flnc | chr6 | Flnc | 0.202796353 | 0 | 0 | 980 | 471 |
| chr6_29589229_29586038_-3191-Tnpo3 | chr6 | Tnpo3 | 0 | 0.364618359 | 0.180646022 | 3192 | 432 |
| chr6_29599698_29578463_-21235-Tnpo3 | chr6 | Tnpo3 | 0.202796353 | 0 | 0 | 21236 | 1050 |
| chr6_29953453_29939037_+14416-Strip2 | chr6 | Strip2 | 0 | 0 | 0.180646022 | 14417 | 400 |
| chr6_30102309_30095340_+6969-Nrf1 | chr6 | Nrf1 | 0.202796353 | 0 | 0 | 6970 | 383 |
| chr6_30126359_30095340_+31019-Nrf1 | chr6 | Nrf1 | 0 | 0 | 0.361292045 | 31020 | 1125 |
| chr6_30126359_30115013_+11346-Nrf1 | chr6 | Nrf1 | 0 | 0 | 0.903230112 | 11347 | 742 |
| chr6_31093595_31080865_-12730-Lncpint | chr6 | Lncpint | 0 | 1.093855076 | 1.083876134 | 12731 | 12731 |
| chr6_31101586_31093363_-8223-Lncpint | chr6 | Lncpint | 0 | 0.182309179 | 0 | 8224 | 8224 |
| chr6_31181149_31087492_-93657-Lncpint | chr6 | Lncpint | 0 | 0.182309179 | 0.541938067 | 93658 | 93658 |
| chr6_31181149_31093363_-87786-Lncpint | chr6 | Lncpint | 0 | 0.182309179 | 0 | 87787 | 87787 |
| chr6_31181149_31166476_-14673-Lncpint | chr6 | Lncpint | 0.405592706 | 0 | 0 | 14674 | 291 |
| chr6_31183553_31166476_-17077-Lncpint | chr6 | Lncpint | 0.202796353 | 0 | 0 | 17078 | 17078 |
| chr6_31197851_31093363_-104488-Lncpint | chr6 | Lncpint | 0 | 0 | 0.180646022 | 104489 | 104489 |
| chr6_31197851_31111034_-86817-Lncpint | chr6 | Lncpint | 0 | 0 | 0.180646022 | 86818 | 86818 |
| chr6_31197851_31121432_-76419-na | chr6 | na | 0 | 0 | 0.180646022 | 76420 | 76420 |
| chr6_31197851_31139729_-58122-Lncpint | chr6 | Lncpint | 0 | 0.182309179 | 0 | 58123 | 58123 |
| chr6_31197851_31163894_-33957-Lncpint | chr6 | Lncpint | 0.202796353 | 0 | 0 | 33958 | 33958 |
| chr6_31197851_31163894_-33957-na | chr6 | na | 0 | 0.09115459 | 0 | 33958 | 33958 |
| chr6_31197851_31166476_-31375-Lncpint | chr6 | Lncpint | 0.202796353 | 0.364618359 | 0 | 31376 | 360 |
| chr6_31197851_31181022_-16829-Lncpint | chr6 | Lncpint | 0 | 0.182309179 | 0.090323011 | 16830 | 200 |
| chr6_31207438_31166476_-40962-Lncpint | chr6 | Lncpint | 0.202796353 | 0 | 0 | 40963 | 40963 |
| chr6_31219135_31166476_-52659-Lncpint | chr6 | Lncpint | 0 | 0.182309179 | 0 | 52660 | 52660 |
| chr6_31337339_31285003_+52336-2210408F21Rik | chr6 | 2210408F21Rik | 0.608389058 | 0 | 0 | 52337 | 426 |
| chr6_31374760_31328289_+46471-2210408F21Rik | chr6 | 2210408F21Rik | 0.202796353 | 0 | 0 | 46472 | 46472 |
| chr6_31459067_31449496_+9571-Mkln1 | chr6 | Mkln1 | 0 | 0.364618359 | 0.361292045 | 9572 | 257 |
| chr6_31478056_31468131_+9925-Mkln1 | chr6 | Mkln1 | 0 | 0.182309179 | 0 | 9926 | 565 |
| chr6_33049424_32968201_-81223-Chchd3 | chr6 | Chchd3 | 0 | 0 | 0.180646022 | 81224 | 288 |
| chr6_34891537_34886066_-5471-Wdr91 | chr6 | Wdr91 | 0 | 0.182309179 | 0 | 5472 | 637 |
| chr6_35080226_35051062_-29164-Cnot4 | chr6 | Cnot4 | 0 | 0.182309179 | 0 | 29165 | 1719 |
| chr6_35080226_35064889_-15337-Cnot4 | chr6 | Cnot4 | 0 | 0 | 0.722584089 | 15338 | 913 |
| chr6_35080226_35064898_-15328-Cnot4 | chr6 | Cnot4 | 0 | 0.182309179 | 0.361292045 | 15329 | 904 |
| chr6_35080226_35077981_-2245-Cnot4 | chr6 | Cnot4 | 0.202796353 | 0 | 0.180646022 | 2246 | 464 |
| chr6_35192038_35186433_+5605-Nup205 | chr6 | Nup205 | 0 | 0.364618359 | 0 | 5606 | 871 |
| chr6_35192038_35189855_+2183-Nup205 | chr6 | Nup205 | 0 | 0 | 0.180646022 | 2184 | 394 |
| chr6_3555466_3545510_+9956-Vps50 | chr6 | Vps50 | 0 | 0 | 0.180646022 | 9957 | 419 |
| chr6_3562358_3522683_+39675-Vps50 | chr6 | Vps50 | 0 | 0.729236718 | 0 | 39676 | 912 |
| chr6_3571108_3545510_+25598-Vps50 | chr6 | Vps50 | 0 | 0 | 0.180646022 | 25599 | 913 |
| chr6_3571108_3565516_+5592-Vps50 | chr6 | Vps50 | 0 | 0.182309179 | 0 | 5593 | 403 |
| chr6_3578915_3522683_+56232-Vps50 | chr6 | Vps50 | 0.202796353 | 0 | 0 | 56233 | 1437 |
| chr6_3578915_3545510_+33405-Vps50 | chr6 | Vps50 | 0 | 0.729236718 | 0 | 33406 | 1035 |
| chr6_3578915_3551013_+27902-Vps50 | chr6 | Vps50 | 0.202796353 | 0 | 0 | 27903 | 902 |
| chr6_3594901_3588006_+6895-Vps50 | chr6 | Vps50 | 0 | 0.182309179 | 0 | 6896 | 486 |
| chr6_37931500_37927526_+3974-Trim24 | chr6 | Trim24 | 0 | 0 | 0.090323011 | 3975 | 262 |
| chr6_38464043_38440455_+23588-Ubn2 | chr6 | Ubn2 | 0.405592706 | 0.182309179 | 0 | 23589 | 927 |
| chr6_38470222_38440455_+29767-Ubn2 | chr6 | Ubn2 | 0.608389058 | 0.182309179 | 0.180646022 | 29768 | 998 |
| chr6_38470222_38452870_+17352-Ubn2 | chr6 | Ubn2 | 0.202796353 | 0 | 0 | 17353 | 905 |
| chr6_38470222_38461475_+8747-Ubn2 | chr6 | Ubn2 | 0.608389058 | 0.911545897 | 2.167752268 | 8748 | 803 |
| chr6_38470222_38462963_+7259-Ubn2 | chr6 | Ubn2 | 0 | 0.182309179 | 0 | 7260 | 665 |
| chr6_38484125_38461475_+22650-Ubn2 | chr6 | Ubn2 | 0.405592706 | 0 | 0 | 22651 | 1361 |
| chr6_38487150_38461475_+25675-Ubn2 | chr6 | Ubn2 | 0.608389058 | 0.364618359 | 0 | 25676 | 1404 |
| chr6_38491931_38440455_+51476-Ubn2 | chr6 | Ubn2 | 0.405592706 | 2.187710153 | 1.264522156 | 51477 | 3147 |
| chr6_38491931_38461475_+30456-Ubn2 | chr6 | Ubn2 | 0 | 0.911545897 | 0 | 30457 | 2952 |
| chr6_38502342_38436411_+65931-na | chr6 | na | 0 | 0 | 0.090323011 | 65932 | 65932 |
| chr6_38502408_38436411_+65997-Ubn2 | chr6 | Ubn2 | 0 | 0 | 0.180646022 | 65998 | 65998 |
| chr6_38502408_38440455_+61953-Ubn2 | chr6 | Ubn2 | 1.825167175 | 2.552328511 | 4.335504536 | 61954 | 3640 |
| chr6_38502408_38461475_+40933-Ubn2 | chr6 | Ubn2 | 0 | 0 | 0.361292045 | 40934 | 3445 |
| chr6_38589286_38568847_+20439-Luc7l2 | chr6 | Luc7l2 | 0 | 0 | 0.541938067 | 20440 | 2015 |
| chr6_38747997_38743076_-4921-Hipk2 | chr6 | Hipk2 | 0 | 0 | 0.180646022 | 4922 | 516 |
| chr6_38822269_38818230_-4039-Hipk2 | chr6 | Hipk2 | 0 | 0 | 0.361292045 | 4040 | 4040 |
| chr6_39338814_39336366_-2448-Slc37a3 | chr6 | Slc37a3 | 0.405592706 | 0 | 0 | 2449 | 1130 |
| chr6_39349518_39347289_-2229-Slc37a3 | chr6 | Slc37a3 | 0 | 0.182309179 | 0 | 2230 | 321 |
| chr6_42311250_42290587_+20663-Clcn1 | chr6 | Clcn1 | 0.202796353 | 0 | 0 | 20664 | 2120 |
| chr6_42357985_42350257_+7728-Zyx | chr6 | Zyx | 0.405592706 | 0 | 0 | 7729 | 1736 |
| chr6_4719496_4707074_-12422-Sgce | chr6 | Sgce | 0.202796353 | 0 | 0.361292045 | 12423 | 553 |
| chr6_47577667_47551732_-25935-Ezh2 | chr6 | Ezh2 | 0.202796353 | 0 | 0.180646022 | 25936 | 899 |
| chr6_50348138_50344874_-3264-Osbpl3 | chr6 | Osbpl3 | 0.202796353 | 0 | 0 | 3265 | 646 |
| chr6_50434755_50434499_-256-na | chr6 | na | 0 | 0 | 0.180646022 | 257 | 257 |
| chr6_52630349_52609145_-21204-Hibadh | chr6 | Hibadh | 0 | 0.364618359 | 0 | 21205 | 21205 |
| chr6_5489211_5486678_-2533-Pdk4 | chr6 | Pdk4 | 0 | 0 | 0.180646022 | 2534 | 401 |
| chr6_56860801_56849638_+11163-Fkbp9 | chr6 | Fkbp9 | 0.202796353 | 0 | 0 | 11164 | 818 |
| chr6_58759198_58720459_-38739-Gm38822 | chr6 | Gm38822 | 0 | 0 | 0.090323011 | 38740 | 38740 |
| chr6_58759198_58749465_-9733-Gm38822 | chr6 | Gm38822 | 0 | 0 | 0.180646022 | 9734 | 9734 |
| chr6_58890234_58876489_+13745-Herc3 | chr6 | Herc3 | 0 | 0 | 0.180646022 | 13746 | 874 |
| chr6_65058132_65052620_+5512-Smarcad1 | chr6 | Smarcad1 | 0 | 0.182309179 | 0 | 5513 | 411 |
| chr6_65092594_65058066_+34528-Smarcad1 | chr6 | Smarcad1 | 0.202796353 | 0 | 0 | 34529 | 1300 |
| chr6_65859478_65855949_+3529-Prdm5 | chr6 | Prdm5 | 0 | 0.546927538 | 0 | 3530 | 565 |
| chr6_65886178_65869181_+16997-Prdm5 | chr6 | Prdm5 | 0 | 0 | 0.180646022 | 16998 | 592 |
| chr6_65901859_65855949_+45910-Prdm5 | chr6 | Prdm5 | 0.405592706 | 0 | 0 | 45911 | 1323 |
| chr6_65901859_65881049_+20810-Prdm5 | chr6 | Prdm5 | 0 | 0.182309179 | 0 | 20811 | 435 |
| chr6_67064718_67043759_+20959-E230016M11Rik | chr6 | E230016M11Rik | 0 | 0 | 0.180646022 | 20960 | 20960 |
| chr6_67116767_67077248_+39519-E230016M11Rik | chr6 | E230016M11Rik | 0 | 0.182309179 | 0 | 39520 | 39520 |
| chr6_71239726_71237357_-2369-Smyd1 | chr6 | Smyd1 | 0 | 0.182309179 | 0.180646022 | 2370 | 522 |
| chr6_71510220_71505734_+4486-Rnf103 | chr6 | Rnf103 | 0.202796353 | 0.182309179 | 0 | 4487 | 1651 |
| chr6_71510220_71508869_+1351-na | chr6 | na | 0 | 0.182309179 | 0 | 1352 | 1352 |
| chr6_71607606_71599317_-8289-Kdm3a | chr6 | Kdm3a | 0.202796353 | 0 | 0 | 8290 | 993 |
| chr6_71871558_71868580_+2978-Immt | chr6 | Immt | 0 | 0.182309179 | 0 | 2979 | 356 |
| chr6_73271122_73256171_+14951-Suclg1 | chr6 | Suclg1 | 0.405592706 | 0 | 0 | 14952 | 728 |
| chr6_83404824_83385985_-18839-na | chr6 | na | 0 | 0.09115459 | 0 | 18840 | 18840 |
| chr6_83404824_83402667_-2157-Tet3 | chr6 | Tet3 | 0 | 0.364618359 | 0 | 2158 | 2158 |
| chr6_83930165_83928653_+1512-Zfp638 | chr6 | Zfp638 | 0 | 0.182309179 | 0.541938067 | 1513 | 1513 |
| chr6_83946363_83928653_+17710-Zfp638 | chr6 | Zfp638 | 0 | 0 | 0.180646022 | 17711 | 2344 |
| chr6_83947941_83942834_+5107-Zfp638 | chr6 | Zfp638 | 0.202796353 | 0 | 0 | 5108 | 853 |
| chr6_83953387_83928653_+24734-Zfp638 | chr6 | Zfp638 | 0 | 0 | 0.361292045 | 24735 | 2579 |
| chr6_83954761_83942834_+11927-Zfp638 | chr6 | Zfp638 | 0 | 0.182309179 | 0 | 11928 | 1016 |
| chr6_83969239_83942834_+26405-Zfp638 | chr6 | Zfp638 | 0 | 0 | 0.361292045 | 26406 | 1837 |
| chr6_84108202_84063321_+44881-Dysf | chr6 | Dysf | 0.202796353 | 0 | 0 | 44882 | 2268 |
| chr6_84152409_84129471_+22938-Dysf | chr6 | Dysf | 0.811185411 | 0 | 0 | 22939 | 1110 |
| chr6_84152409_84149675_+2734-Dysf | chr6 | Dysf | 0.912583588 | 1.276164256 | 0.722584089 | 2735 | 507 |
| chr6_84211267_84203183_+8084-Dysf | chr6 | Dysf | 0 | 0.182309179 | 0 | 8085 | 8085 |
| chr6_85269963_85256343_-13620-Sfxn5 | chr6 | Sfxn5 | 1.115379941 | 0.729236718 | 0.361292045 | 13621 | 268 |
| chr6_85289788_85229194_-60594-Sfxn5 | chr6 | Sfxn5 | 0 | 0.182309179 | 0 | 60595 | 756 |
| chr6_85289788_85256343_-33445-Sfxn5 | chr6 | Sfxn5 | 1.013981764 | 0 | 0 | 33446 | 376 |
| chr6_85342061_85341052_-1009-na | chr6 | na | 0.811185411 | 0.729236718 | 0.541938067 | 1010 | 1010 |
| chr6_85348991_85347780_-1211-Rab11fip5 | chr6 | Rab11fip5 | 0.405592706 | 0.729236718 | 0 | 1212 | 1113 |
| chr6_86967373_86944357_+23016-Aak1 | chr6 | Aak1 | 0.202796353 | 0 | 0 | 23017 | 1729 |
| chr6_86967373_86959032_+8341-Aak1 | chr6 | Aak1 | 0 | 0 | 0.180646022 | 8342 | 739 |
| chr6_86998517_86995840_+2677-na | chr6 | na | 0 | 0 | 0.361292045 | 2678 | 2678 |
| chr6_88298236_88281549_-16687-Eefsec | chr6 | Eefsec | 1.216778117 | 0 | 0.180646022 | 16688 | 817 |
| chr6_88358642_88281549_-77093-Eefsec | chr6 | Eefsec | 0 | 0 | 0.180646022 | 77094 | 1079 |
| chr6_88358642_88352446_-6196-Eefsec | chr6 | Eefsec | 0 | 0 | 0.180646022 | 6197 | 6197 |
| chr6_89470746_89464738_-6008-Chchd6 | chr6 | Chchd6 | 0.608389058 | 0 | 0 | 6009 | 6009 |
| chr6_90384252_90378784_+5468-Zxdc | chr6 | Zxdc | 0.202796353 | 0 | 0 | 5469 | 2592 |
| chr6_90491803_90485218_+6585-na | chr6 | na | 0 | 0 | 0.180646022 | 6586 | 6586 |
| chr6_90596973_90576628_+20345-Aldh1l1 | chr6 | Aldh1l1 | 0 | 0.729236718 | 0.180646022 | 20346 | 981 |
| chr6_90640963_90624812_+16151-Slc41a3 | chr6 | Slc41a3 | 0 | 0.364618359 | 0.180646022 | 16152 | 697 |
| chr6_90644233_90633611_+10622-Slc41a3 | chr6 | Slc41a3 | 0.202796353 | 0 | 0 | 10623 | 801 |
| chr6_91065303_91026805_-38498-Nup210 | chr6 | Nup210 | 0.405592706 | 0 | 0 | 38499 | 2675 |
| chr6_91087983_91036353_-51630-Nup210 | chr6 | Nup210 | 0.202796353 | 0 | 0 | 51631 | 3151 |
| chr6_91699032_91690480_+8552-Slc6a6 | chr6 | Slc6a6 | 0 | 0.182309179 | 0 | 8553 | 8553 |
| chr6_91713044_91711512_+1532-Slc6a6 | chr6 | Slc6a6 | 0.202796353 | 0 | 0.180646022 | 1533 | 1533 |
| chr6_92152417_92139780_+12637-Nr2c2 | chr6 | Nr2c2 | 0.202796353 | 0.182309179 | 0.180646022 | 12638 | 415 |
| chr6_92164293_92149604_+14689-Nr2c2 | chr6 | Nr2c2 | 0 | 0.364618359 | 0 | 14690 | 1598 |
| chr6_92816643_92807015_-9628-Adamts9 | chr6 | Adamts9 | 0 | 0.364618359 | 0 | 9629 | 345 |
| chr6_92860204_92849292_-10912-Adamts9 | chr6 | Adamts9 | 0 | 0.182309179 | 0 | 10913 | 870 |
| chr6_92894386_92887341_-7045-Adamts9 | chr6 | Adamts9 | 0 | 0.364618359 | 0 | 7046 | 679 |
| chr6_93201952_93192109_+9843-Gm38863 | chr6 | Gm38863 | 0 | 0 | 0.180646022 | 9844 | 9844 |
| chr6_93271754_93254714_+17040-na | chr6 | na | 0 | 0.182309179 | 0 | 17041 | 17041 |
| chr6_94050766_93682952_-367814-Magi1 | chr6 | Magi1 | 0 | 0 | 0.361292045 | 367815 | 367815 |
| chr6_94535768_94513805_+21963-Slc25a26 | chr6 | Slc25a26 | 0.608389058 | 0.364618359 | 0 | 21964 | 21964 |
| chr6_94565023_94534144_+30879-Slc25a26 | chr6 | Slc25a26 | 0 | 0.364618359 | 0 | 30880 | 334 |
| chr6_94576378_94549099_+27279-na | chr6 | na | 0 | 0.09115459 | 0 | 27280 | 27280 |
| chr6_94663998_94654847_-9151-Lrig1 | chr6 | Lrig1 | 0.608389058 | 0.09115459 | 0 | 9152 | 147 |
| chr6_98006197_97993127_+13070-na | chr6 | na | 0 | 0 | 0.090323011 | 13071 | 13071 |
| chr6_99402130_99371780_-30350-na | chr6 | na | 0.202796353 | 0 | 0 | 30351 | 30351 |
| chr6_99402130_99392615_-9515-na | chr6 | na | 0.405592706 | 0.364618359 | 0 | 9516 | 9516 |
| chr6_99460450_99435216_-25234-Foxp1 | chr6 | Foxp1 | 0.202796353 | 0 | 0 | 25235 | 25235 |
| chr7_100407829_100405958_+1871-C2cd3 | chr7 | C2cd3 | 0 | 0 | 0.361292045 | 1872 | 472 |
| chr7_100482755_100479374_+3381-Ucp3 | chr7 | Ucp3 | 0.202796353 | 0 | 0 | 3382 | 905 |
| chr7_101722833_101663894_+58939-Clpb | chr7 | Clpb | 0 | 0 | 0.180646022 | 58940 | 782 |
| chr7_102111339_102106667_+4672-Art1 | chr7 | Art1 | 0 | 0.182309179 | 0 | 4673 | 1326 |
| chr7_102176405_102160541_-15864-Nup98 | chr7 | Nup98 | 0.202796353 | 0 | 0 | 15865 | 556 |
| chr7_102186739_102185861_-878-Nup98 | chr7 | Nup98 | 0.202796353 | 0 | 0 | 879 | 289 |
| chr7_102386201_102354501_+31700-Stim1 | chr7 | Stim1 | 0 | 0 | 0.180646022 | 31701 | 246 |
| chr7_102415515_102354501_+61014-Stim1 | chr7 | Stim1 | 0 | 0.364618359 | 0 | 61015 | 652 |
| chr7_105390352_105381080_-9272-Fam160a2 | chr7 | Fam160a2 | 0.202796353 | 0 | 0 | 9273 | 2828 |
| chr7_107697698_107681762_+15936-Ppfibp2 | chr7 | Ppfibp2 | 0 | 0.182309179 | 0 | 15937 | 339 |
| chr7_107708872_107681762_+27110-Ppfibp2 | chr7 | Ppfibp2 | 0 | 0.182309179 | 0.090323011 | 27111 | 432 |
| chr7_109935553_109926827_-8726-Dennd5a | chr7 | Dennd5a | 0.202796353 | 0 | 0 | 8727 | 1028 |
| chr7_110442367_110399296_-43071-Sbf2 | chr7 | Sbf2 | 0 | 0 | 0.180646022 | 43072 | 954 |
| chr7_111077747_111076696_-1051-Eif4g2 | chr7 | Eif4g2 | 0 | 0 | 0.180646022 | 1052 | 594 |
| chr7_111080964_111079293_-1671-Eif4g2 | chr7 | Eif4g2 | 0.202796353 | 0 | 0 | 1672 | 310 |
| chr7_112104468_112101738_+2730-Usp47 | chr7 | Usp47 | 0 | 0.182309179 | 0 | 2731 | 492 |
| chr7_112842102_112759384_+82718-Tead1 | chr7 | Tead1 | 0 | 0.182309179 | 0 | 82719 | 447 |
| chr7_112861556_112839416_+22140-Tead1 | chr7 | Tead1 | 0.202796353 | 0 | 0 | 22141 | 572 |
| chr7_113234696_113225062_+9634-Arntl | chr7 | Arntl | 0.202796353 | 0 | 0 | 9635 | 9635 |
| chr7_114060419_114046647_-13772-Rras2 | chr7 | Rras2 | 0 | 0.182309179 | 0 | 13773 | 13773 |
| chr7_115701785_115659516_-42269-Sox6 | chr7 | Sox6 | 0 | 0 | 0.180646022 | 42270 | 332 |
| chr7_115777258_115777051_-207-Sox6 | chr7 | Sox6 | 0.405592706 | 0 | 0 | 208 | 208 |
| chr7_115801609_115701696_-99913-Sox6 | chr7 | Sox6 | 0.405592706 | 0 | 0 | 99914 | 539 |
| chr7_115801609_115801369_-240-Sox6 | chr7 | Sox6 | 0 | 0 | 0.180646022 | 241 | 241 |
| chr7_115975538_115809811_-165727-na | chr7 | na | 0.202796353 | 0 | 0 | 165728 | 165728 |
| chr7_115975538_115943894_-31644-na | chr7 | na | 0 | 0 | 0.090323011 | 31645 | 31645 |
| chr7_116021756_115777051_-244705-Sox6 | chr7 | Sox6 | 0.405592706 | 0 | 0 | 244706 | 244706 |
| chr7_116378763_116372795_-5968-Pik3c2a | chr7 | Pik3c2a | 0 | 0.182309179 | 0 | 5969 | 692 |
| chr7_118555968_118549935_-6033-Tmc7 | chr7 | Tmc7 | 0 | 0 | 0.361292045 | 6034 | 626 |
| chr7_118760245_118748126_+12119-9030624J02Rik | chr7 | 9030624J02Rik | 0 | 0 | 0.180646022 | 12120 | 439 |
| chr7_120889751_120879814_+9937-Eef2k | chr7 | Eef2k | 0 | 0 | 0.180646022 | 9938 | 1032 |
| chr7_120899546_120879814_+19732-Eef2k | chr7 | Eef2k | 0.405592706 | 0 | 0 | 19733 | 1660 |
| chr7_122010758_121997291_-13467-Gga2 | chr7 | Gga2 | 0 | 0 | 0.180646022 | 13468 | 1077 |
| chr7_122123708_122113250_-10458-Palb2 | chr7 | Palb2 | 0.202796353 | 0 | 0.361292045 | 10459 | 599 |
| chr7_123162877_123143750_+19127-Tnrc6a | chr7 | Tnrc6a | 0 | 0.546927538 | 0 | 19128 | 494 |
| chr7_123172118_123162494_+9624-Tnrc6a | chr7 | Tnrc6a | 0 | 0 | 0.180646022 | 9625 | 2967 |
| chr7_123331265_123318387_-12878-Arhgap17 | chr7 | Arhgap17 | 0.405592706 | 0 | 0 | 12879 | 520 |
| chr7_125770703_125752973_+17730-D430042O09Rik | chr7 | D430042O09Rik | 0 | 0 | 0.361292045 | 17731 | 477 |
| chr7_126153279_126128227_-25052-Xpo6 | chr7 | Xpo6 | 0 | 0.182309179 | 0 | 25053 | 1008 |
| chr7_126153279_126140565_-12714-Xpo6 | chr7 | Xpo6 | 0 | 0.364618359 | 0 | 12715 | 736 |
| chr7_126153279_126149234_-4045-Xpo6 | chr7 | Xpo6 | 0 | 0.182309179 | 0 | 4046 | 626 |
| chr7_126171142_126156724_-14418-Xpo6 | chr7 | Xpo6 | 0 | 0 | 0.180646022 | 14419 | 549 |
| chr7_126447516_126446871_-645-Atp2a1 | chr7 | Atp2a1 | 0 | 0 | 0.180646022 | 646 | 370 |
| chr7_126450550_126446871_-3679-Atp2a1 | chr7 | Atp2a1 | 0 | 0 | 0.180646022 | 3680 | 1561 |
| chr7_126450550_126450105_-445-Atp2a1 | chr7 | Atp2a1 | 0 | 0.182309179 | 0.180646022 | 446 | 345 |
| chr7_126452786_126446545_-6241-Atp2a1 | chr7 | Atp2a1 | 0.202796353 | 0 | 0 | 6242 | 1735 |
| chr7_126453450_126452655_-795-Atp2a1 | chr7 | Atp2a1 | 0.405592706 | 0 | 0 | 796 | 324 |
| chr7_126462187_126453023_-9164-Atp2a1 | chr7 | Atp2a1 | 0 | 0.182309179 | 0 | 9165 | 1151 |
| chr7_126557825_126551975_-5850-Eif3c | chr7 | Eif3c | 0 | 0.182309179 | 0 | 5851 | 1142 |
| chr7_126797776_126797644_-132-Aldoa | chr7 | Aldoa | 0 | 0.364618359 | 0 | 133 | 133 |
| chr7_127214130_127213610_+520-Mylpf | chr7 | Mylpf | 0 | 0.182309179 | 0 | 521 | 168 |
| chr7_127486889_127484542_+2347-Fbrs | chr7 | Fbrs | 1.013981764 | 0.364618359 | 0.180646022 | 2348 | 742 |
| chr7_127522129_127519581_+2548-Srcap | chr7 | Srcap | 0 | 0.364618359 | 0 | 2549 | 579 |
| chr7_127525905_127525293_+612-Srcap | chr7 | Srcap | 0 | 0.182309179 | 0 | 613 | 498 |
| chr7_127532219_127521989_+10230-Srcap | chr7 | Srcap | 0.202796353 | 0 | 0.180646022 | 10231 | 2037 |
| chr7_127532219_127530252_+1967-Srcap | chr7 | Srcap | 0 | 0.182309179 | 0 | 1968 | 1145 |
| chr7_127534961_127534548_+413-Srcap | chr7 | Srcap | 0 | 0.182309179 | 0 | 414 | 324 |
| chr7_127549735_127521989_+27746-Srcap | chr7 | Srcap | 0 | 0.182309179 | 0 | 27747 | 5635 |
| chr7_127553294_127552259_+1035-Srcap | chr7 | Srcap | 0.202796353 | 0 | 0 | 1036 | 602 |
| chr7_127563292_127552259_+11033-Srcap | chr7 | Srcap | 0.202796353 | 0 | 0 | 11034 | 11034 |
| chr7_127787586_127787126_+460-Setd1a | chr7 | Setd1a | 0.202796353 | 0 | 0 | 461 | 265 |
| chr7_128685284_128675298_+9986-Inpp5f | chr7 | Inpp5f | 0 | 0 | 0.180646022 | 9987 | 770 |
| chr7_128724208_128715946_-8262-Mcmbp | chr7 | Mcmbp | 0 | 0.182309179 | 0 | 8263 | 430 |
| chr7_128764149_128750059_+14090-Sec23ip | chr7 | Sec23ip | 0 | 0.182309179 | 0 | 14091 | 1584 |
| chr7_129613973_129600437_+13536-Wdr11 | chr7 | Wdr11 | 0.405592706 | 0 | 0 | 13537 | 1308 |
| chr7_130627499_130601837_+25662-Tacc2 | chr7 | Tacc2 | 0.405592706 | 0.182309179 | 0.722584089 | 25663 | 5324 |
| chr7_130627499_130619602_+7897-Tacc2 | chr7 | Tacc2 | 2.433556234 | 1.914246384 | 2.34839829 | 7898 | 7898 |
| chr7_130627499_130621721_+5778-Tacc2 | chr7 | Tacc2 | 6.895075996 | 3.281565229 | 7.045194871 | 5779 | 5223 |
| chr7_130659288_130621721_+37567-Tacc2 | chr7 | Tacc2 | 0.202796353 | 0 | 0 | 37568 | 37568 |
| chr7_130674739_130663016_+11723-Tacc2 | chr7 | Tacc2 | 0 | 0.182309179 | 0.541938067 | 11724 | 264 |
| chr7_130674739_130665372_+9367-Tacc2 | chr7 | Tacc2 | 0.202796353 | 0 | 0 | 9368 | 9368 |
| chr7_130744616_130739812_+4804-Tacc2 | chr7 | Tacc2 | 0 | 0.182309179 | 0 | 4805 | 581 |
| chr7_130753661_130739812_+13849-Tacc2 | chr7 | Tacc2 | 0 | 0.729236718 | 0.903230112 | 13850 | 846 |
| chr7_131361936_131350651_-11285-2310057M21Rik | chr7 | 2310057M21Rik | 0.405592706 | 0 | 0 | 11286 | 936 |
| chr7_132271001_132262683_-8318-Chst15 | chr7 | Chst15 | 0 | 0.182309179 | 0 | 8319 | 1641 |
| chr7_132760167_132759389_-778-Fam53b | chr7 | Fam53b | 0 | 0.182309179 | 0.180646022 | 779 | 779 |
| chr7_132779385_132771578_-7807-Fam53b | chr7 | Fam53b | 0.304194529 | 0.09115459 | 0 | 7808 | 302 |
| chr7_132950435_132949585_+850-Zranb1 | chr7 | Zranb1 | 0 | 0 | 0.361292045 | 851 | 851 |
| chr7_132960734_132948044_+12690-Zranb1 | chr7 | Zranb1 | 0 | 0.546927538 | 0.180646022 | 12691 | 12691 |
| chr7_132960734_132949585_+11149-Zranb1 | chr7 | Zranb1 | 0.608389058 | 0 | 0.180646022 | 11150 | 11150 |
| chr7_132972761_132949585_+23176-Zranb1 | chr7 | Zranb1 | 0.202796353 | 0 | 0 | 23177 | 1265 |
| chr7_132972761_132971186_+1575-Zranb1 | chr7 | Zranb1 | 0 | 0.09115459 | 0 | 1576 | 226 |
| chr7_132973623_132972185_+1438-Zranb1 | chr7 | Zranb1 | 0 | 0.182309179 | 0 | 1439 | 1439 |
| chr7_133656683_133650501_+6182-Edrf1 | chr7 | Edrf1 | 0 | 0.182309179 | 0 | 6183 | 894 |
| chr7_133749036_133733968_-15068-Dhx32 | chr7 | Dhx32 | 0.405592706 | 0 | 0.180646022 | 15069 | 1072 |
| chr7_134753434_134734050_+19384-Dock1 | chr7 | Dock1 | 0.202796353 | 0 | 0 | 19385 | 887 |
| chr7_134763973_134744950_+19023-Dock1 | chr7 | Dock1 | 0 | 0.182309179 | 0 | 19024 | 843 |
| chr7_134772475_134760812_+11663-Dock1 | chr7 | Dock1 | 0 | 0.546927538 | 0 | 11664 | 711 |
| chr7_134804650_134771467_+33183-Dock1 | chr7 | Dock1 | 0 | 0.182309179 | 0 | 33184 | 821 |
| chr7_139567437_139519431_+48006-Inpp5a | chr7 | Inpp5a | 0 | 0 | 0.180646022 | 48007 | 48007 |
| chr7_140113133_140109987_-3146-Echs1 | chr7 | Echs1 | 0 | 0 | 0.180646022 | 3147 | 651 |
| chr7_142016505_142016079_-426-Mob2 | chr7 | Mob2 | 0 | 0.182309179 | 0 | 427 | 255 |
| chr7_142025957_142009443_-16514-Mob2 | chr7 | Mob2 | 0.202796353 | 0 | 0 | 16515 | 644 |
| chr7_142382612_142380774_+1838-na | chr7 | na | 0.101398176 | 0 | 0 | 1839 | 1839 |
| chr7_144485208_144481085_-4123-Ppfia1 | chr7 | Ppfia1 | 0.405592706 | 0 | 0 | 4124 | 685 |
| chr7_144500403_144491452_-8951-Ppfia1 | chr7 | Ppfia1 | 0 | 0 | 0.180646022 | 8952 | 702 |
| chr7_144614924_144610843_-4081-Ano1 | chr7 | Ano1 | 0 | 0 | 0.180646022 | 4082 | 447 |
| chr7_144650554_144644708_-5846-Ano1 | chr7 | Ano1 | 0 | 0.182309179 | 0 | 5847 | 216 |
| chr7_19043575_19036662_+6913-Sympk | chr7 | Sympk | 0 | 0.546927538 | 0 | 6914 | 1073 |
| chr7_19261213_19260581_-632-Vasp | chr7 | Vasp | 0.202796353 | 0 | 0 | 633 | 381 |
| chr7_19415533_19414119_+1414-Ckm | chr7 | Ckm | 2.027963528 | 0.729236718 | 1.083876134 | 1415 | 500 |
| chr7_19416890_19414897_+1993-Ckm | chr7 | Ckm | 0.811185411 | 0.546927538 | 1.083876134 | 1994 | 460 |
| chr7_19420296_19416719_+3577-Ckm | chr7 | Ckm | 0 | 0 | 0.361292045 | 3578 | 486 |
| chr7_19421587_19416719_+4868-Ckm | chr7 | Ckm | 0.202796353 | 0 | 0 | 4869 | 4869 |
| chr7_19451756_19443117_-8639-Mark4 | chr7 | Mark4 | 0 | 0.182309179 | 0 | 8640 | 735 |
| chr7_25273633_25270835_+2798-Cic | chr7 | Cic | 0 | 0 | 0.180646022 | 2799 | 2799 |
| chr7_28750291_28748955_+1336-Sars2 | chr7 | Sars2 | 0 | 0 | 0.180646022 | 1337 | 401 |
| chr7_29009567_29008384_-1183-Ryr1 | chr7 | Ryr1 | 0.202796353 | 0 | 0 | 1184 | 343 |
| chr7_29040893_29035962_-4931-Ryr1 | chr7 | Ryr1 | 0.202796353 | 0 | 0 | 4932 | 4932 |
| chr7_29040893_29035980_-4913-Ryr1 | chr7 | Ryr1 | 18.45446811 | 12.03240584 | 13.36780565 | 4914 | 650 |
| chr7_29040893_29038347_-2546-na | chr7 | na | 0 | 0.09115459 | 0.090323011 | 2547 | 2547 |
| chr7_29043929_29024003_-19926-Ryr1 | chr7 | Ryr1 | 3.041945292 | 1.093855076 | 1.264522156 | 19927 | 1073 |
| chr7_29043929_29040388_-3541-Ryr1 | chr7 | Ryr1 | 2.027963528 | 0.364618359 | 0.903230112 | 3542 | 404 |
| chr7_29049041_29048778_-263-Ryr1 | chr7 | Ryr1 | 0.405592706 | 0 | 0 | 264 | 127 |
| chr7_29054996_29053062_-1934-Ryr1 | chr7 | Ryr1 | 0 | 0.182309179 | 0 | 1935 | 306 |
| chr7_29062224_29052014_-10210-Ryr1 | chr7 | Ryr1 | 0.608389058 | 0 | 0 | 10211 | 1669 |
| chr7_29065625_29035980_-29645-Ryr1 | chr7 | Ryr1 | 0 | 0.182309179 | 0.903230112 | 29646 | 3226 |
| chr7_29065625_29064429_-1196-Ryr1 | chr7 | Ryr1 | 0 | 0 | 0.361292045 | 1197 | 350 |
| chr7_29070183_29009055_-61128-Ryr1 | chr7 | Ryr1 | 0 | 0 | 0.180646022 | 61129 | 6489 |
| chr7_29074977_29064538_-10439-Ryr1 | chr7 | Ryr1 | 0 | 0 | 0.180646022 | 10440 | 2336 |
| chr7_29086324_29061508_-24816-Ryr1 | chr7 | Ryr1 | 0 | 0 | 0.180646022 | 24817 | 4748 |
| chr7_29086324_29064429_-21895-Ryr1 | chr7 | Ryr1 | 0 | 0.364618359 | 0 | 21896 | 4535 |
| chr7_29086324_29078519_-7805-Ryr1 | chr7 | Ryr1 | 0.202796353 | 0 | 0 | 7806 | 1611 |
| chr7_29086324_29083371_-2953-Ryr1 | chr7 | Ryr1 | 0 | 0.364618359 | 0 | 2954 | 877 |
| chr7_29102999_29075164_-27835-Ryr1 | chr7 | Ryr1 | 0.202796353 | 0 | 0 | 27836 | 4857 |
| chr7_29388151_29383196_-4955-Sipa1l3 | chr7 | Sipa1l3 | 0.202796353 | 0 | 0 | 4956 | 626 |
| chr7_30612474_30606875_-5599-Upk1a | chr7 | Upk1a | 0 | 0.182309179 | 0 | 5600 | 472 |
| chr7_34285953_34284702_-1251-4931406P16Rik | chr7 | 4931406P16Rik | 0 | 0.182309179 | 0.361292045 | 1252 | 1252 |
| chr7_34371184_34353378_-17806-Lsm14a | chr7 | Lsm14a | 0 | 0.182309179 | 0 | 17807 | 17807 |
| chr7_34371184_34353390_-17794-Lsm14a | chr7 | Lsm14a | 0.202796353 | 0.729236718 | 0.361292045 | 17795 | 848 |
| chr7_34375392_34351286_-24106-Lsm14a | chr7 | Lsm14a | 0 | 0 | 0.180646022 | 24107 | 1244 |
| chr7_34611935_34611801_-134-na | chr7 | na | 1.216778117 | 0.364618359 | 0.722584089 | 135 | 135 |
| chr7_35795618_35787718_-7900-Zfp507 | chr7 | Zfp507 | 0 | 0.364618359 | 0 | 7901 | 2326 |
| chr7_37969697_37961388_-8309-Uri1 | chr7 | Uri1 | 0.811185411 | 0.729236718 | 0.361292045 | 8310 | 1362 |
| chr7_37982948_37961388_-21560-Uri1 | chr7 | Uri1 | 1.216778117 | 1.276164256 | 0.180646022 | 21561 | 1577 |
| chr7_38054547_38050127_-4420-Gm30684 | chr7 | Gm30684 | 0 | 0 | 0.180646022 | 4421 | 470 |
| chr7_41628076_41611676_+16400-2610021A01Rik | chr7 | 2610021A01Rik | 0.202796353 | 0 | 0 | 16401 | 3852 |
| chr7_4444321_4434994_-9327-Rdh13 | chr7 | Rdh13 | 0 | 0.182309179 | 0 | 9328 | 415 |
| chr7_44505705_44503637_-2068-Mybpc2 | chr7 | Mybpc2 | 0.405592706 | 0 | 0 | 2069 | 624 |
| chr7_44505705_44504999_-706-Mybpc2 | chr7 | Mybpc2 | 0.202796353 | 0 | 0 | 707 | 437 |
| chr7_44515021_44508231_-6790-Mybpc2 | chr7 | Mybpc2 | 0.202796353 | 0.182309179 | 0 | 6791 | 1205 |
| chr7_44517004_44508969_-8035-Mybpc2 | chr7 | Mybpc2 | 0 | 0.182309179 | 0 | 8036 | 1628 |
| chr7_44520392_44514849_-5543-Mybpc2 | chr7 | Mybpc2 | 0 | 0.182309179 | 0 | 5544 | 791 |
| chr7_44520787_44520333_-454-Mybpc2 | chr7 | Mybpc2 | 0.101398176 | 0 | 0 | 455 | 109 |
| chr7_44665500_44652431_-13069-Myh14 | chr7 | Myh14 | 0.202796353 | 0 | 0 | 13070 | 1105 |
| chr7_44882866_44867060_-15806-Med25 | chr7 | Med25 | 0 | 0.09115459 | 0 | 15807 | 15807 |
| chr7_45149766_45142780_-6986-Aldh16a1 | chr7 | Aldh16a1 | 0.202796353 | 0 | 0 | 6987 | 2157 |
| chr7_45451914_45447898_+4016-Gys1 | chr7 | Gys1 | 0 | 0.182309179 | 0 | 4017 | 501 |
| chr7_45576002_45447898_+128104-Plekha4 | chr7 | Plekha4 | 0 | 0 | 0.361292045 | 128105 | 128105 |
| chr7_45588528_45575431_+13097-Bcat2 | chr7 | Bcat2 | 0 | 0.364618359 | 0 | 13098 | 1412 |
| chr7_46288985_46262640_+26345-Otog | chr7 | Otog | 0.202796353 | 0 | 0 | 26346 | 4040 |
| chr7_46515704_46511955_-3749-na | chr7 | na | 0.202796353 | 0 | 0 | 3750 | 3750 |
| chr7_46814204_46801672_+12532-Gtf2h1 | chr7 | Gtf2h1 | 0 | 0 | 0.180646022 | 12533 | 1154 |
| chr7_4682048_4677707_-4341-Hspbp1 | chr7 | Hspbp1 | 0 | 0.182309179 | 0 | 4342 | 430 |
| chr7_49430725_49420174_+10551-Nav2 | chr7 | Nav2 | 0 | 0 | 0.180646022 | 10552 | 10552 |
| chr7_49554259_49545800_+8459-Nav2 | chr7 | Nav2 | 0 | 0.182309179 | 0 | 8460 | 1559 |
| chr7_51566549_51546662_+19887-Ano5 | chr7 | Ano5 | 0 | 0 | 0.541938067 | 19888 | 814 |
| chr7_51570418_51546662_+23756-Ano5 | chr7 | Ano5 | 0 | 0 | 0.180646022 | 23757 | 966 |
| chr7_51585528_51546662_+38866-Ano5 | chr7 | Ano5 | 0 | 0.182309179 | 0 | 38867 | 1869 |
| chr7_64371659_64361940_-9719-Fan1 | chr7 | Fan1 | 0.811185411 | 0 | 0 | 9720 | 938 |
| chr7_66088949_66080332_+8617-Vimp | chr7 | Vimp | 0.202796353 | 0 | 0 | 8618 | 594 |
| chr7_66660255_66381452_-278803-Aldh1a3 | chr7 | Aldh1a3 | 0.202796353 | 0 | 0 | 278804 | 278804 |
| chr7_67268400_67264865_-3535-Mef2a | chr7 | Mef2a | 0.202796353 | 0.182309179 | 0.180646022 | 3536 | 412 |
| chr7_68259289_68249467_-9822-Pgpep1l | chr7 | Pgpep1l | 0.202796353 | 0 | 0 | 9823 | 9823 |
| chr7_73475579_73463636_-11943-Chd2 | chr7 | Chd2 | 0 | 0 | 0.180646022 | 11944 | 11944 |
| chr7_73484597_73468456_-16141-Chd2 | chr7 | Chd2 | 0 | 0 | 0.180646022 | 16142 | 1413 |
| chr7_73493641_73468456_-25185-Chd2 | chr7 | Chd2 | 0 | 0 | 0.180646022 | 25186 | 1911 |
| chr7_73519708_73490479_-29229-Chd2 | chr7 | Chd2 | 0 | 0 | 0.180646022 | 29230 | 1938 |
| chr7_73540904_73474506_-66398-Chd2 | chr7 | Chd2 | 0.202796353 | 0 | 0 | 66399 | 2947 |
| chr7_75615048_75579502_+35546-Akap13 | chr7 | Akap13 | 0 | 0.182309179 | 0 | 35547 | 3878 |
| chr7_75704539_75683394_+21145-Akap13 | chr7 | Akap13 | 0.202796353 | 0 | 0 | 21146 | 719 |
| chr7_75730611_75663412_+67199-Akap13 | chr7 | Akap13 | 0 | 0 | 0.180646022 | 67200 | 2641 |
| chr7_76335324_76325024_+10300-Agbl1 | chr7 | Agbl1 | 0 | 0 | 0.180646022 | 10301 | 226 |
| chr7_76523437_76519304_+4133-na | chr7 | na | 0.202796353 | 0 | 0 | 4134 | 4134 |
| chr7_76589549_76516777_+72772-Agbl1 | chr7 | Agbl1 | 0 | 0.182309179 | 0 | 72773 | 72773 |
| chr7_80370731_80369746_-985-Man2a2 | chr7 | Man2a2 | 0.405592706 | 0.364618359 | 0 | 986 | 986 |
| chr7_80397250_80395335_-1915-Furin | chr7 | Furin | 0.202796353 | 0 | 0.361292045 | 1916 | 498 |
| chr7_81039265_81037350_+1915-Zfp592 | chr7 | Zfp592 | 0 | 0.364618359 | 0.180646022 | 1916 | 874 |
| chr7_81078833_81076873_+1960-Alpk3 | chr7 | Alpk3 | 0.202796353 | 0 | 0 | 1961 | 1406 |
| chr7_81095054_81092147_+2907-Alpk3 | chr7 | Alpk3 | 0.405592706 | 0 | 0 | 2908 | 2186 |
| chr7_81095529_81092147_+3382-Alpk3 | chr7 | Alpk3 | 0 | 0.182309179 | 0 | 3383 | 2407 |
| chr7_81095693_81076873_+18820-Alpk3 | chr7 | Alpk3 | 0.202796353 | 0 | 0 | 18821 | 3849 |
| chr7_81095693_81092147_+3546-Alpk3 | chr7 | Alpk3 | 0 | 1.093855076 | 0.180646022 | 3547 | 2443 |
| chr7_81283013_81281178_+1835-Pde8a | chr7 | Pde8a | 0 | 0.182309179 | 0 | 1836 | 248 |
| chr7_81436377_81355875_-80502-Cpeb1 | chr7 | Cpeb1 | 0 | 0 | 0.180646022 | 80503 | 1200 |
| chr7_81436377_81372012_-64365-Cpeb1 | chr7 | Cpeb1 | 0.202796353 | 0 | 0 | 64366 | 367 |
| chr7_81436377_81431562_-4815-Cpeb1 | chr7 | Cpeb1 | 0 | 0 | 0.180646022 | 4816 | 4816 |
| chr7_81550025_81544960_-5065-Fsd2 | chr7 | Fsd2 | 0.202796353 | 0 | 0 | 5066 | 564 |
| chr7_82683081_82649526_+33555-Efl1 | chr7 | Efl1 | 0 | 0.182309179 | 0 | 33556 | 947 |
| chr7_82686764_82681352_+5412-Efl1 | chr7 | Efl1 | 0 | 0 | 0.361292045 | 5413 | 561 |
| chr7_89962361_89955531_-6830-Eed | chr7 | Eed | 0 | 0.364618359 | 0 | 6831 | 339 |
| chr7_89971605_89964729_-6876-Eed | chr7 | Eed | 0 | 0.182309179 | 0 | 6877 | 500 |
| chr7_89977049_89962256_-14793-Eed | chr7 | Eed | 0 | 0 | 0.180646022 | 14794 | 852 |
| chr7_90173075_90169182_+3893-Picalm | chr7 | Picalm | 0.202796353 | 0 | 0 | 3894 | 355 |
| chr7_90191753_90160448_+31305-Picalm | chr7 | Picalm | 0.405592706 | 0 | 0 | 31306 | 1649 |
| chr7_90197020_90160448_+36572-Picalm | chr7 | Picalm | 0.811185411 | 0.729236718 | 0.903230112 | 36573 | 1838 |
| chr7_90197020_90189150_+7870-Picalm | chr7 | Picalm | 0.811185411 | 0.364618359 | 0 | 7871 | 560 |
| chr7_91626813_91626329_+484-na | chr7 | na | 0 | 0 | 0.180646022 | 485 | 485 |
| chr7_92665865_92645990_-19875-Pcf11 | chr7 | Pcf11 | 0.202796353 | 0.729236718 | 0.722584089 | 19876 | 4647 |
| chr7_92665865_92648926_-16939-Pcf11 | chr7 | Pcf11 | 0 | 0.182309179 | 0 | 16940 | 4178 |
| chr7_97040036_97023618_+16418-Nars2 | chr7 | Nars2 | 0.202796353 | 0.182309179 | 0.180646022 | 16419 | 779 |
| chr7_97326060_97319967_-6093-Usp35 | chr7 | Usp35 | 0 | 0.182309179 | 0 | 6094 | 1293 |
| chr7_97664766_97653053_+11713-Rsf1 | chr7 | Rsf1 | 0 | 0.182309179 | 0.180646022 | 11714 | 2131 |
| chr7_97670882_97660813_+10069-Rsf1 | chr7 | Rsf1 | 0.202796353 | 0 | 0.180646022 | 10070 | 2161 |
| chr7_97679142_97660813_+18329-Rsf1 | chr7 | Rsf1 | 0.405592706 | 0 | 0 | 18330 | 2602 |
| chr7_98610865_98599752_-11113-Emsy | chr7 | Emsy | 0 | 0.182309179 | 0 | 11114 | 562 |
| chr7_99424229_99387104_+37125-Gdpd5 | chr7 | Gdpd5 | 0 | 0.182309179 | 0 | 37126 | 603 |
| chr7_99814307_99813193_-1114-na | chr7 | na | 0 | 0.182309179 | 0 | 1115 | 1115 |
| chr8_105202547_105178593_+23954-Cbfb | chr8 | Cbfb | 0.202796353 | 0.364618359 | 0 | 23955 | 330 |
| chr8_105202578_105178593_+23985-Cbfb | chr8 | Cbfb | 0 | 0.729236718 | 0 | 23986 | 361 |
| chr8_105666792_105663754_+3038-Ctcf | chr8 | Ctcf | 0.202796353 | 0.546927538 | 0 | 3039 | 1095 |
| chr8_105888755_105888388_+367-Edc4 | chr8 | Edc4 | 0.405592706 | 0 | 0 | 368 | 291 |
| chr8_106030443_106025906_+4537-Dus2 | chr8 | Dus2 | 0 | 0 | 0.361292045 | 4538 | 138 |
| chr8_106109129_106096164_+12965-Nfatc3 | chr8 | Nfatc3 | 0.405592706 | 0 | 0 | 12966 | 1502 |
| chr8_106725285_106695449_+29836-Tango6 | chr8 | Tango6 | 0.202796353 | 0 | 0 | 29837 | 1143 |
| chr8_106748895_106716609_+32286-Tango6 | chr8 | Tango6 | 0 | 0.182309179 | 0 | 32287 | 32287 |
| chr8_107315261_107314202_-1059-na | chr8 | na | 0 | 0 | 0.180646022 | 1060 | 1060 |
| chr8_107361824_107338809_+23015-Nfat5 | chr8 | Nfat5 | 0 | 0 | 0.180646022 | 23016 | 1518 |
| chr8_107458001_107324639_+133362-Nfat5 | chr8 | Nfat5 | 0.405592706 | 0 | 0 | 133363 | 133363 |
| chr8_109819042_109802947_+16095-Ap1g1 | chr8 | Ap1g1 | 0 | 0.364618359 | 0 | 16096 | 329 |
| chr8_111124612_111121148_+3464-Pdpr | chr8 | Pdpr | 0 | 0 | 1.083876134 | 3465 | 415 |
| chr8_111496895_111490292_-6603-Wdr59 | chr8 | Wdr59 | 0 | 0.182309179 | 0 | 6604 | 441 |
| chr8_11203008_11201658_-1350-Col4a1 | chr8 | Col4a1 | 0 | 0.546927538 | 0 | 1351 | 288 |
| chr8_114489004_114444468_+44536-Wwox | chr8 | Wwox | 0 | 0 | 0.180646022 | 44537 | 409 |
| chr8_114712251_114706201_+6050-Wwox | chr8 | Wwox | 0.405592706 | 0 | 0 | 6051 | 451 |
| chr8_11562334_11561418_+916-na | chr8 | na | 0 | 0 | 0.180646022 | 917 | 917 |
| chr8_117456957_117426490_+30467-Cmip | chr8 | Cmip | 0 | 0.182309179 | 0 | 30468 | 1524 |
| chr8_117621263_117612904_+8359-Plcg2 | chr8 | Plcg2 | 0.202796353 | 0 | 0 | 8360 | 518 |
| chr8_11800868_11785713_+15155-Arhgef7 | chr8 | Arhgef7 | 0.202796353 | 0 | 0 | 15156 | 482 |
| chr8_11809576_11782525_+27051-Arhgef7 | chr8 | Arhgef7 | 0 | 0 | 0.361292045 | 27052 | 935 |
| chr8_118505715_118505604_+111-Cdh13 | chr8 | Cdh13 | 0.304194529 | 0 | 0 | 112 | 112 |
| chr8_118757484_118757368_+116-Cdh13 | chr8 | Cdh13 | 0 | 0 | 0.180646022 | 117 | 117 |
| chr8_118968193_118505604_+462589-Cdh13 | chr8 | Cdh13 | 0.202796353 | 0 | 0 | 462590 | 736 |
| chr8_119535377_119531049_-4328-Mbtps1 | chr8 | Mbtps1 | 0.811185411 | 0.364618359 | 0 | 4329 | 417 |
| chr8_119538960_119531049_-7911-Mbtps1 | chr8 | Mbtps1 | 0 | 0.182309179 | 0 | 7912 | 602 |
| chr8_119546241_119515578_-30663-Mbtps1 | chr8 | Mbtps1 | 0 | 0.182309179 | 0 | 30664 | 2541 |
| chr8_119546241_119528924_-17317-Mbtps1 | chr8 | Mbtps1 | 0.405592706 | 0 | 0.180646022 | 17318 | 1430 |
| chr8_119546241_119535275_-10966-Mbtps1 | chr8 | Mbtps1 | 0 | 0.182309179 | 0 | 10967 | 971 |
| chr8_119546241_119538127_-8114-Mbtps1 | chr8 | Mbtps1 | 0.202796353 | 0 | 0 | 8115 | 868 |
| chr8_119546241_119541585_-4656-Mbtps1 | chr8 | Mbtps1 | 0.202796353 | 0.364618359 | 0 | 4657 | 683 |
| chr8_119942138_119931849_+10289-Usp10 | chr8 | Usp10 | 0.405592706 | 0 | 0.180646022 | 10290 | 1156 |
| chr8_120354179_120353990_+189-Gse1 | chr8 | Gse1 | 0 | 0.182309179 | 0 | 190 | 190 |
| chr8_120489151_120488708_+443-na | chr8 | na | 0 | 0 | 0.180646022 | 444 | 444 |
| chr8_121820188_121804947_-15241-Klhdc4 | chr8 | Klhdc4 | 0.405592706 | 0.364618359 | 0.903230112 | 15242 | 457 |
| chr8_121820220_121804947_-15273-Klhdc4 | chr8 | Klhdc4 | 0 | 0 | 0.180646022 | 15274 | 489 |
| chr8_122781588_122779946_+1642-Acsf3 | chr8 | Acsf3 | 0.405592706 | 0 | 0 | 1643 | 846 |
| chr8_123067097_123066473_+624-Spg7 | chr8 | Spg7 | 0 | 0 | 0.180646022 | 625 | 625 |
| chr8_124887819_124884965_+2854-Gnpat | chr8 | Gnpat | 0 | 0 | 0.180646022 | 2855 | 474 |
| chr8_125088448_125087454_+994-Disc1 | chr8 | Disc1 | 0 | 0 | 0.180646022 | 995 | 995 |
| chr8_125090442_125087454_+2988-Disc1 | chr8 | Disc1 | 0.405592706 | 0.729236718 | 0 | 2989 | 1065 |
| chr8_125155110_125087454_+67656-Disc1 | chr8 | Disc1 | 0.608389058 | 0 | 0 | 67657 | 1728 |
| chr8_125165181_125087454_+77727-Disc1 | chr8 | Disc1 | 0.202796353 | 0.182309179 | 0 | 77728 | 1917 |
| chr8_127409707_127378186_+31521-Pard3 | chr8 | Pard3 | 0.202796353 | 0.364618359 | 0 | 31522 | 1030 |
| chr8_127426753_127405375_+21378-Pard3 | chr8 | Pard3 | 0 | 0 | 0.180646022 | 21379 | 847 |
| chr8_127460384_127415570_+44814-Pard3 | chr8 | Pard3 | 0 | 0 | 0.180646022 | 44815 | 571 |
| chr8_128207761_128199298_-8463-LOC105243337 | chr8 | LOC105243337 | 0 | 0.182309179 | 0 | 8464 | 8464 |
| chr8_13167965_13167158_+807-Lamp1 | chr8 | Lamp1 | 0 | 0.182309179 | 0 | 808 | 379 |
| chr8_13281125_13271590_-9535-Dcun1d2 | chr8 | Dcun1d2 | 0 | 0 | 0.361292045 | 9536 | 517 |
| chr8_13313956_13303728_+10228-Tmco3 | chr8 | Tmco3 | 0.608389058 | 0 | 0 | 10229 | 655 |
| chr8_14954928_14945280_+9648-Arhgef10 | chr8 | Arhgef10 | 0 | 0.182309179 | 0 | 9649 | 480 |
| chr8_14982435_14979800_+2635-Arhgef10 | chr8 | Arhgef10 | 0 | 0 | 0.361292045 | 2636 | 502 |
| chr8_15070764_15065694_+5070-Myom2 | chr8 | Myom2 | 0 | 0 | 0.180646022 | 5071 | 546 |
| chr8_15085314_15063843_+21471-Myom2 | chr8 | Myom2 | 1.41957447 | 0.911545897 | 0.903230112 | 21472 | 1527 |
| chr8_15085314_15065694_+19620-Myom2 | chr8 | Myom2 | 0 | 0 | 0.180646022 | 19621 | 1409 |
| chr8_15104188_15083230_+20958-Myom2 | chr8 | Myom2 | 0.202796353 | 0 | 0 | 20959 | 1005 |
| chr8_22421450_22414716_+6734-Mrps31 | chr8 | Mrps31 | 0 | 0 | 1.083876134 | 6735 | 588 |
| chr8_23095310_23084719_+10591-Ank1 | chr8 | Ank1 | 0.202796353 | 0 | 0 | 10592 | 978 |
| chr8_23135834_23131438_+4396-Ank1 | chr8 | Ank1 | 0.202796353 | 0 | 0 | 4397 | 932 |
| chr8_25177994_25164389_-13605-Tacc1 | chr8 | Tacc1 | 0.202796353 | 0 | 0 | 13606 | 753 |
| chr8_25555774_25555495_+279-Fgfr1 | chr8 | Fgfr1 | 0 | 0.182309179 | 0 | 280 | 280 |
| chr8_25555774_25555508_+266-Fgfr1 | chr8 | Fgfr1 | 0.608389058 | 0 | 0 | 267 | 267 |
| chr8_25564484_25555508_+8976-Fgfr1 | chr8 | Fgfr1 | 0.202796353 | 0 | 0.722584089 | 8977 | 990 |
| chr8_25594166_25580316_-13850-Letm2 | chr8 | Letm2 | 0.202796353 | 0 | 0 | 13851 | 1258 |
| chr8_25649703_25640577_+9126-Whsc1l1 | chr8 | Whsc1l1 | 0.202796353 | 0.546927538 | 0 | 9127 | 984 |
| chr8_25649703_25640649_+9054-Whsc1l1 | chr8 | Whsc1l1 | 0 | 0.182309179 | 0 | 9055 | 912 |
| chr8_25659895_25640577_+19318-Whsc1l1 | chr8 | Whsc1l1 | 0 | 0.182309179 | 0 | 19319 | 1139 |
| chr8_25663256_25659741_+3515-Whsc1l1 | chr8 | Whsc1l1 | 0.202796353 | 0 | 0.361292045 | 3516 | 671 |
| chr8_25666156_25640577_+25579-Whsc1l1 | chr8 | Whsc1l1 | 0.811185411 | 0.729236718 | 0.541938067 | 25580 | 1782 |
| chr8_25675742_25640577_+35165-na | chr8 | na | 0.101398176 | 0 | 0 | 35166 | 35166 |
| chr8_25679994_25666030_+13964-Whsc1l1 | chr8 | Whsc1l1 | 0.405592706 | 0 | 0 | 13965 | 534 |
| chr8_25741354_25735745_-5609-Ddhd2 | chr8 | Ddhd2 | 0 | 0.546927538 | 0 | 5610 | 527 |
| chr8_25753173_25727630_-25543-Ddhd2 | chr8 | Ddhd2 | 0 | 0 | 0.180646022 | 25544 | 4022 |
| chr8_25753173_25736007_-17166-Ddhd2 | chr8 | Ddhd2 | 0 | 0 | 0.180646022 | 17167 | 1434 |
| chr8_26013555_26007187_-6368-Fnta | chr8 | Fnta | 0 | 0 | 1.083876134 | 6369 | 433 |
| chr8_31236430_31225802_+10628-Fut10 | chr8 | Fut10 | 0.405592706 | 0 | 0 | 10629 | 10629 |
| chr8_3198275_3173480_-24795-Insr | chr8 | Insr | 0.405592706 | 0 | 0 | 24796 | 1420 |
| chr8_3258934_3258383_-551-Insr | chr8 | Insr | 2.027963528 | 1.823091794 | 0.541938067 | 552 | 552 |
| chr8_33343692_33342977_-715-Wrn | chr8 | Wrn | 0 | 0.182309179 | 0 | 716 | 295 |
| chr8_33353399_33280772_-72627-Wrn | chr8 | Wrn | 0.202796353 | 0 | 0 | 72628 | 2889 |
| chr8_33611921_33610655_+1266-Ppp2cb | chr8 | Ppp2cb | 0.202796353 | 0 | 0.903230112 | 1267 | 384 |
| chr8_33866404_33862986_-3418-Rbpms | chr8 | Rbpms | 0 | 0 | 0.090323011 | 3419 | 180 |
| chr8_34140836_33834304_-306532-Dctn6 | chr8 | Dctn6 | 0.202796353 | 0 | 0 | 306533 | 306533 |
| chr8_34873214_34848582_-24632-Tnks | chr8 | Tnks | 0.202796353 | 0 | 0 | 24633 | 1801 |
| chr8_39071516_39046515_+25001-Tusc3 | chr8 | Tusc3 | 0 | 0 | 0.180646022 | 25002 | 570 |
| chr8_40905727_40904446_+1281-Slc7a2 | chr8 | Slc7a2 | 0 | 0 | 0.180646022 | 1282 | 357 |
| chr8_41041679_41015388_-26291-Mtus1 | chr8 | Mtus1 | 0 | 0.182309179 | 0 | 26292 | 489 |
| chr8_41056684_41048048_-8636-Mtus1 | chr8 | Mtus1 | 0.405592706 | 0 | 0 | 8637 | 321 |
| chr8_41084840_41048048_-36792-Mtus1 | chr8 | Mtus1 | 0.405592706 | 0 | 0 | 36793 | 2744 |
| chr8_41084840_41076232_-8608-Mtus1 | chr8 | Mtus1 | 0.811185411 | 1.367318845 | 1.264522156 | 8609 | 2423 |
| chr8_41084840_41082614_-2226-Mtus1 | chr8 | Mtus1 | 1.013981764 | 0.182309179 | 0.903230112 | 2227 | 2227 |
| chr8_41092005_41082614_-9391-na | chr8 | na | 0 | 0.09115459 | 0 | 9392 | 9392 |
| chr8_44953481_44950196_+3285-Fat1 | chr8 | Fat1 | 0 | 0 | 0.180646022 | 3286 | 3286 |
| chr8_45775703_45745420_+30283-Sorbs2 | chr8 | Sorbs2 | 0 | 0 | 0.361292045 | 30284 | 1481 |
| chr8_45775703_45759053_+16650-Sorbs2 | chr8 | Sorbs2 | 0 | 0 | 0.541938067 | 16651 | 714 |
| chr8_45783626_45745420_+38206-Sorbs2 | chr8 | Sorbs2 | 0 | 0.364618359 | 0 | 38207 | 1724 |
| chr8_45785325_45759053_+26272-Sorbs2 | chr8 | Sorbs2 | 0.202796353 | 0 | 0 | 26273 | 1014 |
| chr8_45792968_45770546_+22422-Sorbs2 | chr8 | Sorbs2 | 0 | 0 | 0.180646022 | 22423 | 873 |
| chr8_45801011_45783543_+17468-Sorbs2 | chr8 | Sorbs2 | 0 | 0 | 0.180646022 | 17469 | 2099 |
| chr8_45805853_45745420_+60433-na | chr8 | na | 0.101398176 | 0 | 0 | 60434 | 60434 |
| chr8_45986363_45983523_+2840-Ufsp2 | chr8 | Ufsp2 | 0 | 0.182309179 | 0 | 2841 | 2841 |
| chr8_46493026_46492800_+226-Acsl1 | chr8 | Acsl1 | 0.405592706 | 0 | 0 | 227 | 227 |
| chr8_46823995_46793493_+30502-Irf2 | chr8 | Irf2 | 0 | 0 | 0.180646022 | 30503 | 30503 |
| chr8_46837838_46793493_+44345-Irf2 | chr8 | Irf2 | 0.202796353 | 0 | 0 | 44346 | 700 |
| chr8_47513428_47493239_-20189-Trappc11 | chr8 | Trappc11 | 0.202796353 | 0 | 0 | 20190 | 1936 |
| chr8_47514877_47513283_-1594-Trappc11 | chr8 | Trappc11 | 0.405592706 | 0 | 0 | 1595 | 454 |
| chr8_47920698_47899436_-21262-Wwc2 | chr8 | Wwc2 | 0 | 0.364618359 | 0 | 21263 | 391 |
| chr8_55111085_55101811_-9274-na | chr8 | na | 0 | 0 | 0.180646022 | 9275 | 9275 |
| chr8_61054622_61049784_+4838-Nek1 | chr8 | Nek1 | 0.202796353 | 0 | 0 | 4839 | 399 |
| chr8_61089799_61054520_+35279-Nek1 | chr8 | Nek1 | 0 | 0 | 0.180646022 | 35280 | 863 |
| chr8_68000825_67960387_-40438-Psd3 | chr8 | Psd3 | 0 | 0 | 0.180646022 | 40439 | 1568 |
| chr8_68121628_68108661_-12967-Psd3 | chr8 | Psd3 | 0.202796353 | 0 | 0 | 12968 | 12968 |
| chr8_68158433_68120748_-37685-Psd3 | chr8 | Psd3 | 0 | 0.182309179 | 0 | 37686 | 37686 |
| chr8_68162612_68120748_-41864-Psd3 | chr8 | Psd3 | 0 | 0.182309179 | 0 | 41865 | 928 |
| chr8_68461851_68460924_-927-Csgalnact1 | chr8 | Csgalnact1 | 0.608389058 | 0 | 0 | 928 | 928 |
| chr8_69798513_69797225_+1288-Atp13a1 | chr8 | Atp13a1 | 0.202796353 | 0 | 0 | 1289 | 420 |
| chr8_69950754_69935779_-14975-Gatad2a | chr8 | Gatad2a | 0.202796353 | 0 | 0 | 14976 | 360 |
| chr8_70252101_70251614_+487-Sugp2 | chr8 | Sugp2 | 0 | 0 | 0.180646022 | 488 | 488 |
| chr8_70914994_70911846_+3148-Map1s | chr8 | Map1s | 0.202796353 | 0 | 0.180646022 | 3149 | 2651 |
| chr8_71334394_71327880_+6514-Myo9b | chr8 | Myo9b | 0 | 0.182309179 | 0 | 6515 | 803 |
| chr8_71339020_71333254_+5766-Myo9b | chr8 | Myo9b | 0.202796353 | 0 | 0 | 5767 | 648 |
| chr8_72177670_72168427_+9243-Rab8a | chr8 | Rab8a | 0.405592706 | 0 | 0 | 9244 | 407 |
| chr8_72400233_72367904_-32329-Eps15l1 | chr8 | Eps15l1 | 0 | 0 | 0.180646022 | 32330 | 6029 |
| chr8_72400233_72389408_-10825-Eps15l1 | chr8 | Eps15l1 | 0.202796353 | 0 | 0 | 10826 | 525 |
| chr8_72662444_72653486_+8958-Nwd1 | chr8 | Nwd1 | 0 | 0.182309179 | 0 | 8959 | 501 |
| chr8_75030122_75023998_+6124-Hmgxb4 | chr8 | Hmgxb4 | 0 | 0 | 0.361292045 | 6125 | 427 |
| chr8_77217585_77085527_+132058-Nr3c2 | chr8 | Nr3c2 | 0 | 0 | 0.180646022 | 132059 | 1030 |
| chr8_77351672_77310731_-40941-Arhgap10 | chr8 | Arhgap10 | 1.216778117 | 0 | 0 | 40942 | 476 |
| chr8_77351672_77344640_-7032-Arhgap10 | chr8 | Arhgap10 | 0 | 0.546927538 | 0.541938067 | 7033 | 325 |
| chr8_77358634_77310731_-47903-Arhgap10 | chr8 | Arhgap10 | 0.202796353 | 0 | 0 | 47904 | 564 |
| chr8_77358634_77344640_-13994-Arhgap10 | chr8 | Arhgap10 | 1.216778117 | 0 | 0 | 13995 | 413 |
| chr8_77365160_77351614_-13546-na | chr8 | na | 0.202796353 | 0 | 0 | 13547 | 13547 |
| chr8_77365160_77358547_-6613-Arhgap10 | chr8 | Arhgap10 | 0.202796353 | 0.182309179 | 0.180646022 | 6614 | 163 |
| chr8_77411128_77344640_-66488-Arhgap10 | chr8 | Arhgap10 | 0 | 0 | 0.361292045 | 66489 | 884 |
| chr8_77411128_77382725_-28403-Arhgap10 | chr8 | Arhgap10 | 1.013981764 | 1.640782614 | 2.34839829 | 28404 | 396 |
| chr8_77411128_77384751_-26377-Arhgap10 | chr8 | Arhgap10 | 2.230759881 | 0.729236718 | 1.987106246 | 26378 | 330 |
| chr8_77413627_77344640_-68987-Arhgap10 | chr8 | Arhgap10 | 0 | 0.364618359 | 0 | 68988 | 1014 |
| chr8_77413627_77382725_-30902-Arhgap10 | chr8 | Arhgap10 | 0.405592706 | 0 | 0.180646022 | 30903 | 526 |
| chr8_77420158_77358547_-61611-Arhgap10 | chr8 | Arhgap10 | 0 | 0 | 0.180646022 | 61612 | 776 |
| chr8_77420747_77382725_-38022-Arhgap10 | chr8 | Arhgap10 | 0.202796353 | 0 | 0 | 38023 | 742 |
| chr8_77420747_77384751_-35996-Arhgap10 | chr8 | Arhgap10 | 0.405592706 | 0 | 0 | 35997 | 676 |
| chr8_77420747_77409548_-11199-Arhgap10 | chr8 | Arhgap10 | 0 | 0.364618359 | 0 | 11200 | 548 |
| chr8_77426431_77409548_-16883-Arhgap10 | chr8 | Arhgap10 | 0 | 0 | 0.180646022 | 16884 | 650 |
| chr8_77432811_77409548_-23263-Arhgap10 | chr8 | Arhgap10 | 0.202796353 | 0 | 0 | 23264 | 722 |
| chr8_77450923_77384751_-66172-Arhgap10 | chr8 | Arhgap10 | 0.202796353 | 0 | 0 | 66173 | 1008 |
| chr8_78578757_78515586_+63171-Slc10a7 | chr8 | Slc10a7 | 0 | 0.182309179 | 0 | 63172 | 335 |
| chr8_78589249_78578719_+10530-Slc10a7 | chr8 | Slc10a7 | 0.101398176 | 0 | 0 | 10531 | 139 |
| chr8_78706948_78515586_+191362-Slc10a7 | chr8 | Slc10a7 | 0 | 0.182309179 | 0 | 191363 | 847 |
| chr8_79356469_79343654_-12815-Smad1 | chr8 | Smad1 | 0.202796353 | 0 | 0 | 12816 | 854 |
| chr8_80800395_80784627_-15768-Gab1 | chr8 | Gab1 | 0.202796353 | 0 | 0 | 15769 | 1516 |
| chr8_80800395_80788490_-11905-Gab1 | chr8 | Gab1 | 0.405592706 | 0 | 0 | 11906 | 1126 |
| chr8_80800395_80791389_-9006-Gab1 | chr8 | Gab1 | 0 | 0 | 0.361292045 | 9007 | 521 |
| chr8_81743814_81676391_+67423-Inpp4b | chr8 | Inpp4b | 0 | 0.364618359 | 0 | 67424 | 363 |
| chr8_83042782_82990347_+52435-Rnf150 | chr8 | Rnf150 | 0.405592706 | 0 | 0 | 52436 | 753 |
| chr8_83042782_83036025_+6757-Rnf150 | chr8 | Rnf150 | 0.202796353 | 0.364618359 | 0 | 6758 | 308 |
| chr8_83590830_83582397_+8433-LOC105246804 | chr8 | LOC105246804 | 0 | 0.364618359 | 0 | 8434 | 368 |
| chr8_83728211_83728095_-116-Adgre5 | chr8 | Adgre5 | 0.202796353 | 0 | 0 | 117 | 117 |
| chr8_84727967_84721643_-6324-Nfix | chr8 | Nfix | 0.405592706 | 0 | 0 | 6325 | 695 |
| chr8_84772315_84762018_-10297-Nfix | chr8 | Nfix | 0.304194529 | 0 | 0.451615056 | 10298 | 10298 |
| chr8_84779275_84771784_-7491-Nfix | chr8 | Nfix | 0.202796353 | 0.273463769 | 0 | 7492 | 7492 |
| chr8_85519622_85509179_+10443-Gpt2 | chr8 | Gpt2 | 0.202796353 | 0 | 0 | 10444 | 767 |
| chr8_85553410_85546513_-6897-Dnaja2 | chr8 | Dnaja2 | 0.202796353 | 0.182309179 | 0.722584089 | 6898 | 636 |
| chr8_85726290_85722631_-3659-Itfg1 | chr8 | Itfg1 | 0 | 0.182309179 | 0 | 3660 | 326 |
| chr8_85879810_85848674_+31136-na | chr8 | na | 0 | 0.182309179 | 0 | 31137 | 31137 |
| chr8_85879810_85875634_+4176-Phkb | chr8 | Phkb | 0.202796353 | 0.729236718 | 0.180646022 | 4177 | 329 |
| chr8_85901957_85896430_+5527-Phkb | chr8 | Phkb | 0 | 0 | 0.180646022 | 5528 | 189 |
| chr8_85922242_85870152_+52090-Phkb | chr8 | Phkb | 0.202796353 | 0 | 0 | 52091 | 52091 |
| chr8_85967677_85946052_+21625-Phkb | chr8 | Phkb | 0.202796353 | 0 | 0 | 21626 | 21626 |
| chr8_85970997_85848674_+122323-na | chr8 | na | 0.101398176 | 0 | 0.180646022 | 122324 | 122324 |
| chr8_85970997_85875634_+95363-Phkb | chr8 | Phkb | 0.202796353 | 0 | 0 | 95364 | 1382 |
| chr8_85970997_85922127_+48870-Phkb | chr8 | Phkb | 0 | 0 | 0.361292045 | 48871 | 864 |
| chr8_85970997_85940841_+30156-Phkb | chr8 | Phkb | 0.405592706 | 0.364618359 | 0.361292045 | 30157 | 684 |
| chr8_85970997_85942192_+28805-Phkb | chr8 | Phkb | 0.202796353 | 0 | 0 | 28806 | 588 |
| chr8_86049819_86011017_+38802-Phkb | chr8 | Phkb | 0 | 0.182309179 | 0 | 38803 | 1437 |
| chr8_86049819_86016859_+32960-Phkb | chr8 | Phkb | 0 | 0.364618359 | 0 | 32961 | 1287 |
| chr8_8641256_8639206_-2050-Efnb2 | chr8 | Efnb2 | 0 | 0 | 0.180646022 | 2051 | 457 |
| chr8_86673069_86631386_+41683-Lonp2 | chr8 | Lonp2 | 0.405592706 | 0.182309179 | 0.361292045 | 41684 | 1562 |
| chr8_86673069_86665691_+7378-Lonp2 | chr8 | Lonp2 | 0 | 0.546927538 | 0.180646022 | 7379 | 412 |
| chr8_88158265_88156462_+1803-Heatr3 | chr8 | Heatr3 | 0 | 0 | 0.090323011 | 1804 | 469 |
| chr8_88723212_88705226_+17986-Cyld | chr8 | Cyld | 0 | 0.182309179 | 0 | 17987 | 1285 |
| chr8_91006710_90994460_+12250-Chd9 | chr8 | Chd9 | 0 | 0.182309179 | 0 | 12251 | 1332 |
| chr8_94286617_94281271_+5346-Nup93 | chr8 | Nup93 | 0 | 0.364618359 | 0 | 5347 | 192 |
| chr8_95465710_95455858_-9852-Csnk2a2 | chr8 | Csnk2a2 | 0.202796353 | 0 | 0 | 9853 | 607 |
| chr8_95749074_95748259_-815-Cnot1 | chr8 | Cnot1 | 0 | 0.182309179 | 0 | 816 | 372 |
| chr9_100803354_100776756_+26598-Stag1 | chr9 | Stag1 | 0 | 0.182309179 | 0 | 26599 | 508 |
| chr9_100866214_100705153_+161061-Stag1 | chr9 | Stag1 | 0 | 0 | 0.180646022 | 161062 | 1574 |
| chr9_101124015_101100571_+23444-Msl2 | chr9 | Msl2 | 0 | 0.182309179 | 0 | 23445 | 23445 |
| chr9_101148738_101125023_-23715-Ppp2r3a | chr9 | Ppp2r3a | 0 | 0.364618359 | 0 | 23716 | 698 |
| chr9_101153874_101125023_-28851-Ppp2r3a | chr9 | Ppp2r3a | 0.405592706 | 0.364618359 | 1.264522156 | 28852 | 785 |
| chr9_104126788_104113462_+13326-Acad11 | chr9 | Acad11 | 0.405592706 | 0 | 0 | 13327 | 814 |
| chr9_104186789_104182505_-4284-Dnajc13 | chr9 | Dnajc13 | 0 | 0 | 0.180646022 | 4285 | 400 |
| chr9_105467817_105459660_-8157-Atp2c1 | chr9 | Atp2c1 | 0 | 0 | 0.180646022 | 8158 | 414 |
| chr9_105494597_105431495_-63102-Atp2c1 | chr9 | Atp2c1 | 0.202796353 | 0 | 0 | 63103 | 2025 |
| chr9_106185318_106180566_+4752-Wdr82 | chr9 | Wdr82 | 0 | 0.182309179 | 0 | 4753 | 510 |
| chr9_106186512_106183616_+2896-Wdr82 | chr9 | Wdr82 | 0.202796353 | 0 | 0.180646022 | 2897 | 586 |
| chr9_106246978_106243162_-3816-Alas1 | chr9 | Alas1 | 0.202796353 | 0 | 0 | 3817 | 454 |
| chr9_106830934_106829061_+1873-Vprbp | chr9 | Vprbp | 0 | 0.182309179 | 0 | 1874 | 195 |
| chr9_106854294_106844136_+10158-Vprbp | chr9 | Vprbp | 0.202796353 | 0 | 0 | 10159 | 844 |
| chr9_107592203_107591568_+635-Ifrd2 | chr9 | Ifrd2 | 0 | 0.182309179 | 0 | 636 | 267 |
| chr9_107754182_107746019_-8163-Rbm5 | chr9 | Rbm5 | 0.202796353 | 0 | 0 | 8164 | 923 |
| chr9_108374318_108350910_+23408-Usp4 | chr9 | Usp4 | 0.202796353 | 0 | 0 | 23409 | 1590 |
| chr9_108489374_108482881_+6493-Lamb2 | chr9 | Lamb2 | 0 | 0.182309179 | 0 | 6494 | 3550 |
| chr9_108544977_108528589_+16388-Qrich1 | chr9 | Qrich1 | 0.202796353 | 0.546927538 | 0.541938067 | 16389 | 1938 |
| chr9_109979091_109944884_+34207-LOC102639109 | chr9 | LOC102639109 | 0.202796353 | 0 | 0 | 34208 | 34208 |
| chr9_109979091_109944884_+34207-Map4 | chr9 | Map4 | 0 | 0 | 0.180646022 | 34208 | 34208 |
| chr9_109999840_109978850_+20990-Map4 | chr9 | Map4 | 0.202796353 | 0.546927538 | 0 | 20991 | 311 |
| chr9_110027792_109978850_+48942-Map4 | chr9 | Map4 | 0 | 0.182309179 | 0 | 48943 | 548 |
| chr9_110036855_109999772_+37083-Map4 | chr9 | Map4 | 0 | 0 | 0.180646022 | 37084 | 1689 |
| chr9_110040467_109978850_+61617-Map4 | chr9 | Map4 | 0 | 0.182309179 | 0.361292045 | 61618 | 61618 |
| chr9_110040467_110026101_+14366-Map4 | chr9 | Map4 | 0 | 0 | 0.180646022 | 14367 | 14367 |
| chr9_110043765_109978850_+64915-na | chr9 | na | 0 | 0.09115459 | 0 | 64916 | 64916 |
| chr9_110068985_110063017_+5968-Map4 | chr9 | Map4 | 0.608389058 | 0 | 0.903230112 | 5969 | 866 |
| chr9_110068985_110067485_+1500-Map4 | chr9 | Map4 | 0 | 0.364618359 | 0 | 1501 | 537 |
| chr9_110072705_110063017_+9688-Map4 | chr9 | Map4 | 0.202796353 | 0 | 0 | 9689 | 1073 |
| chr9_110098859_110091419_-7440-Dhx30 | chr9 | Dhx30 | 0 | 0 | 0.361292045 | 7441 | 815 |
| chr9_110185936_110182922_+3014-Smarcc1 | chr9 | Smarcc1 | 0 | 0 | 0.180646022 | 3015 | 308 |
| chr9_110310311_110310072_-239-na | chr9 | na | 0 | 0 | 0.361292045 | 240 | 240 |
| chr9_110439058_110436011_-3047-Klhl18 | chr9 | Klhl18 | 0 | 0.182309179 | 0 | 3048 | 536 |
| chr9_110553234_110547209_+6025-Setd2 | chr9 | Setd2 | 0 | 0.546927538 | 0 | 6026 | 4418 |
| chr9_110574274_110537362_+36912-Setd2 | chr9 | Setd2 | 0.202796353 | 0 | 0 | 36913 | 36913 |
| chr9_110574274_110545276_+28998-Setd2 | chr9 | Setd2 | 0.202796353 | 0 | 0 | 28999 | 5947 |
| chr9_110574274_110573612_+662-Setd2 | chr9 | Setd2 | 0 | 0 | 0.361292045 | 663 | 663 |
| chr9_110599253_110571947_+27306-Setd2 | chr9 | Setd2 | 0 | 0 | 0.180646022 | 27307 | 2035 |
| chr9_110637685_110636891_-794-Nbeal2 | chr9 | Nbeal2 | 0 | 0.182309179 | 0 | 795 | 498 |
| chr9_110970159_110960802_+9357-Lrrc2 | chr9 | Lrrc2 | 0 | 0 | 0.180646022 | 9358 | 648 |
| chr9_110980963_110979470_+1493-Lrrc2 | chr9 | Lrrc2 | 0 | 0 | 0.180646022 | 1494 | 293 |
| chr9_111180272_111175777_+4495-Lrrfip2 | chr9 | Lrrfip2 | 0 | 1.093855076 | 0 | 4496 | 342 |
| chr9_111199835_111182819_+17016-Lrrfip2 | chr9 | Lrrfip2 | 0 | 0.273463769 | 0 | 17017 | 573 |
| chr9_111205847_111182819_+23028-Lrrfip2 | chr9 | Lrrfip2 | 0.405592706 | 0 | 0.541938067 | 23029 | 666 |
| chr9_111205847_111182819_+23028-na | chr9 | na | 0 | 0.182309179 | 0 | 23029 | 23029 |
| chr9_111205847_111184190_+21657-Lrrfip2 | chr9 | Lrrfip2 | 0.608389058 | 1.093855076 | 1.083876134 | 21658 | 588 |
| chr9_111205847_111188762_+17085-Lrrfip2 | chr9 | Lrrfip2 | 0.202796353 | 0.546927538 | 1.083876134 | 17086 | 498 |
| chr9_113899599_113889958_+9641-Clasp2 | chr9 | Clasp2 | 1.013981764 | 1.458473435 | 0.180646022 | 9642 | 531 |
| chr9_114459725_114450597_+9128-Glb1 | chr9 | Glb1 | 0.202796353 | 0 | 0 | 9129 | 414 |
| chr9_115966323_115937142_+29181-Gadl1 | chr9 | Gadl1 | 0.202796353 | 0 | 0 | 29182 | 1013 |
| chr9_117882102_117881779_-323-LOC108167721 | chr9 | LOC108167721 | 0.202796353 | 0 | 0 | 324 | 324 |
| chr9_118534705_118527010_+7695-Golga4 | chr9 | Golga4 | 0.405592706 | 0 | 0.180646022 | 7696 | 516 |
| chr9_119304917_119284546_-20371-Oxsr1 | chr9 | Oxsr1 | 0.405592706 | 0 | 0 | 20372 | 493 |
| chr9_119916833_119902286_+14547-Wdr48 | chr9 | Wdr48 | 0.608389058 | 0 | 0 | 14548 | 1233 |
| chr9_119918557_119902286_+16271-Wdr48 | chr9 | Wdr48 | 0.202796353 | 0 | 0 | 16272 | 1426 |
| chr9_121319952_121318105_+1847-na | chr9 | na | 0 | 0 | 0.180646022 | 1848 | 1848 |
| chr9_121431555_121364001_+67554-Trak1 | chr9 | Trak1 | 0 | 0 | 0.180646022 | 67555 | 632 |
| chr9_121741743_121728578_+13165-Nktr | chr9 | Nktr | 0 | 0.182309179 | 0 | 13166 | 417 |
| chr9_121745988_121731478_+14510-Nktr | chr9 | Nktr | 0 | 0.364618359 | 0 | 14511 | 731 |
| chr9_122137601_122131893_+5708-Snrk | chr9 | Snrk | 0 | 0 | 5.058088625 | 5709 | 985 |
| chr9_122157297_122136906_+20391-Snrk | chr9 | Snrk | 0 | 0 | 0.180646022 | 20392 | 838 |
| chr9_122275663_122201771_-73892-Ano10 | chr9 | Ano10 | 0.405592706 | 0 | 0 | 73893 | 1928 |
| chr9_122857638_122856699_-939-Zfp445 | chr9 | Zfp445 | 0.101398176 | 0 | 0 | 940 | 391 |
| chr9_123395011_123377701_+17310-Lars2 | chr9 | Lars2 | 0 | 0.182309179 | 0 | 17311 | 282 |
| chr9_123500232_122157156_+1343076-Kif15 | chr9 | Kif15 | 0 | 0 | 0.180646022 | 1343077 | 1343077 |
| chr9_123500232_123409614_+90618-Lars2 | chr9 | Lars2 | 0.202796353 | 0 | 0 | 90619 | 90619 |
| chr9_123591015_123552767_+38248-Sacm1l | chr9 | Sacm1l | 0 | 0.182309179 | 0 | 38249 | 1410 |
| chr9_14575439_14547690_-27749-Amotl1 | chr9 | Amotl1 | 0.405592706 | 0 | 0.361292045 | 27750 | 1852 |
| chr9_14596600_14592754_-3846-Amotl1 | chr9 | Amotl1 | 0.405592706 | 0.729236718 | 0 | 3847 | 1108 |
| chr9_21205413_21192395_+13018-Pde4a | chr9 | Pde4a | 0 | 0 | 0.180646022 | 13019 | 1419 |
| chr9_21660871_21655627_+5244-Smarca4 | chr9 | Smarca4 | 0 | 0.182309179 | 0 | 5245 | 437 |
| chr9_22579593_22567705_+11888-Bbs9 | chr9 | Bbs9 | 0.811185411 | 0.182309179 | 0 | 11889 | 574 |
| chr9_22659145_22643745_+15400-Bbs9 | chr9 | Bbs9 | 0.811185411 | 0.546927538 | 0.361292045 | 15401 | 460 |
| chr9_22793933_22643745_+150188-Bbs9 | chr9 | Bbs9 | 0.202796353 | 0 | 0 | 150189 | 969 |
| chr9_25125023_25109440_-15583-Herpud2 | chr9 | Herpud2 | 0.202796353 | 0 | 0 | 15584 | 714 |
| chr9_31405657_31394703_+10954-Nfrkb | chr9 | Nfrkb | 0 | 0 | 0.180646022 | 10955 | 1756 |
| chr9_32250787_32246447_+4340-Arhgap32 | chr9 | Arhgap32 | 0.405592706 | 0.182309179 | 0.180646022 | 4341 | 679 |
| chr9_32476773_32461251_-15522-Fli1 | chr9 | Fli1 | 0 | 0 | 0.180646022 | 15523 | 571 |
| chr9_3450151_3441055_+9096-Cwf19l2 | chr9 | Cwf19l2 | 0 | 0 | 0.361292045 | 9097 | 433 |
| chr9_3460131_3441055_+19076-Cwf19l2 | chr9 | Cwf19l2 | 0.405592706 | 0.182309179 | 0 | 19077 | 1000 |
| chr9_3477962_3475483_+2479-Cwf19l2 | chr9 | Cwf19l2 | 0 | 0.182309179 | 0 | 2480 | 248 |
| chr9_34985144_34983621_-1523-Kirrel3os | chr9 | Kirrel3os | 0 | 0.182309179 | 0 | 1524 | 488 |
| chr9_35210165_35207479_-2686-Foxred1 | chr9 | Foxred1 | 0.202796353 | 0 | 0 | 2687 | 910 |
| chr9_36911263_36909629_-1634-Pknox2 | chr9 | Pknox2 | 0 | 0.364618359 | 0.180646022 | 1635 | 348 |
| chr9_3759881_3759396_+485-Gucy1a2 | chr9 | Gucy1a2 | 0 | 0.364618359 | 0 | 486 | 486 |
| chr9_42444588_42437115_-7473-Tbcel | chr9 | Tbcel | 0.202796353 | 0 | 0 | 7474 | 683 |
| chr9_43008111_43005544_-2567-Arhgef12 | chr9 | Arhgef12 | 0 | 0.182309179 | 0 | 2568 | 306 |
| chr9_43020783_43008016_-12767-Arhgef12 | chr9 | Arhgef12 | 0 | 0 | 0.180646022 | 12768 | 689 |
| chr9_43027295_42972185_-55110-Arhgef12 | chr9 | Arhgef12 | 0.405592706 | 0 | 0 | 55111 | 3855 |
| chr9_43496442_43495825_+617-na | chr9 | na | 0.202796353 | 0 | 0 | 618 | 618 |
| chr9_44091322_44089132_+2190-Usp2 | chr9 | Usp2 | 0 | 0.729236718 | 0 | 2191 | 398 |
| chr9_44816659_44809064_-7595-Kmt2a | chr9 | Kmt2a | 0.202796353 | 0 | 0 | 7596 | 808 |
| chr9_44826959_44824325_-2634-Kmt2a | chr9 | Kmt2a | 0 | 0 | 0.361292045 | 2635 | 494 |
| chr9_44829268_44828784_-484-Kmt2a | chr9 | Kmt2a | 0.202796353 | 0 | 0 | 485 | 298 |
| chr9_44829271_44828784_-487-Kmt2a | chr9 | Kmt2a | 0.405592706 | 0.729236718 | 0 | 488 | 301 |
| chr9_44830123_44828784_-1339-Kmt2a | chr9 | Kmt2a | 0 | 0.182309179 | 0 | 1340 | 375 |
| chr9_45869544_45866566_-2978-Rnf214 | chr9 | Rnf214 | 0 | 0.182309179 | 0 | 2979 | 1634 |
| chr9_45900256_45869179_-31077-Rnf214 | chr9 | Rnf214 | 0.405592706 | 0 | 0 | 31078 | 1228 |
| chr9_45900256_45890804_-9452-Rnf214 | chr9 | Rnf214 | 0 | 0.182309179 | 0.180646022 | 9453 | 852 |
| chr9_45911249_45908528_+2721-Pcsk7 | chr9 | Pcsk7 | 0 | 0.182309179 | 0 | 2722 | 902 |
| chr9_47813945_47787971_+25974-Cadm1 | chr9 | Cadm1 | 0 | 0.182309179 | 0 | 25975 | 870 |
| chr9_48743561_48717283_-26278-Zbtb16 | chr9 | Zbtb16 | 0.202796353 | 0 | 0 | 26279 | 185 |
| chr9_48833099_48717283_-115816-Zbtb16 | chr9 | Zbtb16 | 0.202796353 | 0.182309179 | 0 | 115817 | 1542 |
| chr9_48833099_48743464_-89635-Zbtb16 | chr9 | Zbtb16 | 0 | 0.364618359 | 0 | 89636 | 1455 |
| chr9_48833099_48831743_-1356-Zbtb16 | chr9 | Zbtb16 | 0 | 0.182309179 | 0 | 1357 | 1357 |
| chr9_49035960_49023906_+12054-Usp28 | chr9 | Usp28 | 0.202796353 | 0 | 0 | 12055 | 1570 |
| chr9_50524779_50504621_-20158-Pts | chr9 | Pts | 0.202796353 | 0 | 0 | 20159 | 20159 |
| chr9_50917652_50917014_-638-Sik2 | chr9 | Sik2 | 0 | 0 | 0.361292045 | 639 | 249 |
| chr9_53209776_53204059_-5717-Ddx10 | chr9 | Ddx10 | 0.405592706 | 0 | 0 | 5718 | 649 |
| chr9_53225635_53204059_-21576-Ddx10 | chr9 | Ddx10 | 0 | 0.182309179 | 0 | 21577 | 999 |
| chr9_53453670_53453353_-317-Atm | chr9 | Atm | 0 | 0.182309179 | 0 | 318 | 222 |
| chr9_53491040_53441674_-49366-Atm | chr9 | Atm | 0 | 0.182309179 | 0 | 49367 | 4750 |
| chr9_54428123_54423230_-4893-Dmxl2 | chr9 | Dmxl2 | 0 | 0 | 0.180646022 | 4894 | 334 |
| chr9_54900572_54883897_+16675-Ireb2 | chr9 | Ireb2 | 0.202796353 | 0 | 0 | 16676 | 1663 |
| chr9_55602921_55580207_-22714-Scaper | chr9 | Scaper | 0 | 0.182309179 | 0.180646022 | 22715 | 541 |
| chr9_55816809_55807013_-9796-Scaper | chr9 | Scaper | 0 | 0.182309179 | 0 | 9797 | 642 |
| chr9_56260762_56257527_-3235-Peak1 | chr9 | Peak1 | 0 | 0 | 0.180646022 | 3236 | 3236 |
| chr9_56488829_56467358_+21471-Hmg20a | chr9 | Hmg20a | 0.405592706 | 0 | 0 | 21472 | 908 |
| chr9_57119187_57117382_+1805-Sin3a | chr9 | Sin3a | 0.202796353 | 0 | 0 | 1806 | 371 |
| chr9_58651939_58618663_+33276-Nptn | chr9 | Nptn | 0.608389058 | 0.364618359 | 0 | 33277 | 1216 |
| chr9_58652009_58618663_+33346-Nptn | chr9 | Nptn | 0.405592706 | 0.364618359 | 0.361292045 | 33347 | 1286 |
| chr9_59313236_59303489_+9747-Adpgk | chr9 | Adpgk | 0.405592706 | 0.364618359 | 0.180646022 | 9748 | 381 |
| chr9_59396611_59394878_-1733-Arih1 | chr9 | Arih1 | 0 | 0 | 0.180646022 | 1734 | 374 |
| chr9_59405855_59396351_-9504-Arih1 | chr9 | Arih1 | 0.202796353 | 0.182309179 | 0 | 9505 | 450 |
| chr9_59633604_59632916_+688-Parp6 | chr9 | Parp6 | 0 | 0 | 0.180646022 | 689 | 189 |
| chr9_60854503_60850130_+4373-Uaca | chr9 | Uaca | 0.202796353 | 0.182309179 | 0.541938067 | 4374 | 366 |
| chr9_62923655_62919351_-4304-Pias1 | chr9 | Pias1 | 0 | 0.182309179 | 0 | 4305 | 359 |
| chr9_6293937_6243233_+50704-Pdgfd | chr9 | Pdgfd | 0.202796353 | 0 | 0 | 50705 | 50705 |
| chr9_63343456_63322203_-21253-Map2k5 | chr9 | Map2k5 | 0 | 0 | 0.180646022 | 21254 | 293 |
| chr9_63358060_63338085_-19975-Map2k5 | chr9 | Map2k5 | 0 | 0 | 0.361292045 | 19976 | 247 |
| chr9_64330915_64322402_-8513-Dis3l | chr9 | Dis3l | 0 | 0.182309179 | 0 | 8514 | 701 |
| chr9_64873139_64857667_+15472-Dennd4a | chr9 | Dennd4a | 0 | 0 | 0.180646022 | 15473 | 913 |
| chr9_64910185_64894398_+15787-Dennd4a | chr9 | Dennd4a | 0 | 0 | 0.361292045 | 15788 | 2279 |
| chr9_65045788_65036969_+8819-Dpp8 | chr9 | Dpp8 | 0 | 0 | 0.180646022 | 8820 | 729 |
| chr9_65075888_65034875_+41013-Dpp8 | chr9 | Dpp8 | 0 | 0 | 0.180646022 | 41014 | 3215 |
| chr9_65121920_65113259_+8661-Igdcc4 | chr9 | Igdcc4 | 0.202796353 | 0 | 0 | 8662 | 774 |
| chr9_65441688_65439530_+2158-Mtfmt | chr9 | Mtfmt | 0 | 0.182309179 | 0 | 2159 | 302 |
| chr9_65481968_65475840_+6128-Spg21 | chr9 | Spg21 | 0 | 0.182309179 | 0 | 6129 | 363 |
| chr9_65697669_65697032_-637-Zfp609 | chr9 | Zfp609 | 0.202796353 | 0 | 0 | 638 | 393 |
| chr9_65701188_65698838_-2350-Zfp609 | chr9 | Zfp609 | 0.202796353 | 0 | 0 | 2351 | 2351 |
| chr9_66433844_66399768_+34076-Herc1 | chr9 | Herc1 | 0 | 0.182309179 | 0 | 34077 | 2401 |
| chr9_66434598_66416184_+18414-Herc1 | chr9 | Herc1 | 0 | 0 | 0.180646022 | 18415 | 1717 |
| chr9_66458425_66450703_+7722-Herc1 | chr9 | Herc1 | 0 | 0.182309179 | 0 | 7723 | 1485 |
| chr9_66465581_66450703_+14878-Herc1 | chr9 | Herc1 | 1.216778117 | 0 | 0 | 14879 | 2354 |
| chr9_66547955_66540062_-7893-Usp3 | chr9 | Usp3 | 0 | 0.182309179 | 0 | 7894 | 477 |
| chr9_66566903_66540062_-26841-na | chr9 | na | 0 | 0 | 0.180646022 | 26842 | 26842 |
| chr9_67047911_67047786_-125-Tpm1 | chr9 | Tpm1 | 0 | 0.182309179 | 0 | 126 | 126 |
| chr9_67266923_67261891_-5032-Tln2 | chr9 | Tln2 | 0 | 0.364618359 | 0 | 5033 | 498 |
| chr9_67397683_67386557_-11126-Tln2 | chr9 | Tln2 | 0 | 0.182309179 | 0 | 11127 | 824 |
| chr9_67890389_67876227_+14162-Vps13c | chr9 | Vps13c | 0 | 0 | 0.361292045 | 14163 | 891 |
| chr9_68665154_68663345_+1809-Rora | chr9 | Rora | 0 | 0 | 0.090323011 | 1810 | 1436 |
| chr9_68677130_68663345_+13785-na | chr9 | na | 0.101398176 | 0 | 0 | 13786 | 13786 |
| chr9_68677130_68676649_+481-na | chr9 | na | 0 | 0.364618359 | 0 | 482 | 482 |
| chr9_68793029_68733842_+59187-9530091C08Rik | chr9 | 9530091C08Rik | 0.405592706 | 0 | 0 | 59188 | 59188 |
| chr9_69417109_69410112_+6997-Ice2 | chr9 | Ice2 | 0 | 0 | 0.180646022 | 6998 | 1797 |
| chr9_69999270_69997081_+2189-Bnip2 | chr9 | Bnip2 | 0 | 0 | 0.180646022 | 2190 | 354 |
| chr9_70000945_69999094_+1851-Bnip2 | chr9 | Bnip2 | 0.608389058 | 0 | 0 | 1852 | 280 |
| chr9_70327276_70322392_+4884-Myo1e | chr9 | Myo1e | 0.202796353 | 0 | 0 | 4885 | 400 |
| chr9_70337809_70324833_+12976-Myo1e | chr9 | Myo1e | 0.202796353 | 0 | 0 | 12977 | 546 |
| chr9_70443750_70442272_-1478-Rnf111 | chr9 | Rnf111 | 0 | 0 | 0.180646022 | 1479 | 502 |
| chr9_70589117_70579282_+9835-Sltm | chr9 | Sltm | 0.202796353 | 0 | 0 | 9836 | 1795 |
| chr9_70616906_70607458_-9448-Fam63b | chr9 | Fam63b | 0.405592706 | 0 | 0 | 9449 | 518 |
| chr9_70644946_70607458_-37488-Fam63b | chr9 | Fam63b | 0.202796353 | 0 | 0 | 37489 | 921 |
| chr9_72233329_72230266_-3063-Gm27188 | chr9 | Gm27188 | 0 | 0 | 0.180646022 | 3064 | 348 |
| chr9_72593360_72577039_+16321-Rfx7 | chr9 | Rfx7 | 0 | 0 | 0.361292045 | 16322 | 240 |
| chr9_72677531_72669952_+7579-Nedd4 | chr9 | Nedd4 | 0 | 0 | 0.180646022 | 7580 | 357 |
| chr9_72686114_72669952_+16162-Nedd4 | chr9 | Nedd4 | 0.405592706 | 0.182309179 | 0 | 16163 | 411 |
| chr9_72710539_72709112_+1427-Nedd4 | chr9 | Nedd4 | 0 | 0 | 0.180646022 | 1428 | 113 |
| chr9_72726530_72721301_+5229-Nedd4 | chr9 | Nedd4 | 0 | 0.182309179 | 0 | 5230 | 469 |
| chr9_73015429_73010297_+5132-Ccpg1 | chr9 | Ccpg1 | 0 | 0.09115459 | 0 | 5133 | 3616 |
| chr9_73016275_73007393_+8882-na | chr9 | na | 0 | 0 | 0.090323011 | 8883 | 8883 |
| chr9_75131208_75116186_+15022-Myo5a | chr9 | Myo5a | 0 | 0 | 0.180646022 | 15023 | 15023 |
| chr9_75273720_75251441_+22279-Myo5c | chr9 | Myo5c | 0 | 0 | 0.180646022 | 22280 | 1640 |
| chr9_77190737_77138350_-52387-Mlip | chr9 | Mlip | 0 | 0.182309179 | 0 | 52388 | 636 |
| chr9_77190737_77164765_-25972-Mlip | chr9 | Mlip | 1.825167175 | 0.911545897 | 0 | 25973 | 429 |
| chr9_77217048_77164765_-52283-Mlip | chr9 | Mlip | 0 | 0.364618359 | 0 | 52284 | 501 |
| chr9_77217048_77173959_-43089-Mlip | chr9 | Mlip | 0 | 0 | 0.180646022 | 43090 | 372 |
| chr9_77230954_77164765_-66189-Mlip | chr9 | Mlip | 1.013981764 | 0 | 0.541938067 | 66190 | 1971 |
| chr9_77230954_77181352_-49602-Mlip | chr9 | Mlip | 0.608389058 | 0 | 0 | 49603 | 1797 |
| chr9_77230954_77216977_-13977-Mlip | chr9 | Mlip | 3.041945292 | 2.91694687 | 3.793566469 | 13978 | 1542 |
| chr9_77231029_77216977_-14052-Mlip | chr9 | Mlip | 0 | 0.364618359 | 1.083876134 | 14053 | 1617 |
| chr9_78095998_78095145_-853-Fbxo9 | chr9 | Fbxo9 | 0 | 0.182309179 | 0 | 854 | 246 |
| chr9_7827009_7825615_-1394-Birc2 | chr9 | Birc2 | 0.101398176 | 0.09115459 | 0.361292045 | 1395 | 128 |
| chr9_78461037_78457210_+3827-Mto1 | chr9 | Mto1 | 0.202796353 | 0 | 0 | 3828 | 651 |
| chr9_79820706_79815840_-4866-Filip1 | chr9 | Filip1 | 0.608389058 | 0.182309179 | 0.541938067 | 4867 | 2867 |
| chr9_79820706_79817898_-2808-Filip1 | chr9 | Filip1 | 0 | 0.182309179 | 0 | 2809 | 2809 |
| chr9_80103764_80089849_+13915-Senp6 | chr9 | Senp6 | 0 | 0 | 0.180646022 | 13916 | 401 |
| chr9_80140906_80126143_+14763-Senp6 | chr9 | Senp6 | 0 | 0.182309179 | 0 | 14764 | 1058 |
| chr9_80269722_80262274_+7448-Myo6 | chr9 | Myo6 | 0 | 0.182309179 | 0 | 7449 | 389 |
| chr9_80276401_80266126_+10275-Myo6 | chr9 | Myo6 | 0 | 0 | 0.361292045 | 10276 | 531 |
| chr9_80288216_80281461_+6755-Myo6 | chr9 | Myo6 | 0.405592706 | 0 | 0 | 6756 | 790 |
[truncated: 10,953 more chars]
