# Supplementary material for: Comprehensive analysis of circular RNA profiles in skeletal muscles of aging mice and after aerobic exercise intervention
Source: Aging (Albany NY). 2020 Mar 17;12(6):5071–90. doi: 10.18632/aging.102932 (PMC7138574; doi:10.18632/aging.102932)
Supplement: Supplementary Tables 2-4, 6 [file aging-12-102932-s001..pdf]

SUPPLEMENTARY TABLES

Supplementary Table 2. The differential circRNA profiles in Qu muscles of aging mice compared with young mice.

ID	Log2FC	P-Value	GeneName	Type
chr17_30923856_30513556_-410300-Glo1	11.34485	0.000103	GLO1	Up
chr11_31061586_31055458_+6128-Asb3	11.22942	0.000195	ASB3	Up
chr15_95920378_95864225_+56153-Ano6	10.96651	0.000753	ANO6	Up
chr11_51734661_51729452_-5209-Sec24a	10.64476	0.00325	SEC24a	Up
chr2_76856366_76847388_-8978-Ttn	10.64476	0.00325	TTN	Up
chr5_106666845_106634831_-32014-Zfp644	10.45224	0.007065	ZFP644	Up
chr18_82682287_82664838_-17449-Zfp236	10.45224	0.007065	ZFP236	Up
chr12_53142768_53139381_+3387-Akap6	10.23002	0.015841	AKAP6	Up
chr9_16378254_16374949_-3305-Fat3	10.23002	0.015841	FAT3	Up
chr7_81095693_81092147_+3546-Alpk3	10.23002	0.015841	ALPK3	Up
chr6_31093595_31080865_-12730-Lncpint	10.23002	0.015841	LNCPINT	Up
chr16_43577748_43569681_+8067-Zbtb20	9.967226	0.036493	ZBTB20	Up
chr5_106666845_106618071_-48774-Zfp644	9.967226	0.036493	ZFP644	Up
chr11_50308929_50304326_-4603-Canx	9.967226	0.036493	CANX	Up
chr6_119825819_119824386_-1433-Erc1	9.967226	0.036493	ERC1	Up
chr11_107620214_107592706_+27508-Helz	9.967226	0.036493	HELZ	Up
chr12_35107556_35085939_+21617-Snx13	9.967226	0.036493	SNX13	Up
chr13_59800716_59789848_-10868-Zcchc6	9.967226	0.036493	ZCCHC6	Up
chr17_86120746_86098512_-22234-Srbd1	3.163531	0.017703	SRBD1	Up
chr2_76856366_76853605_-2761-Ttn	3.163531	0.017703	TTN	Up
chr14_21637640_21626719_+10921-Kat6b	3.038825	1.92E-05	KAT6b	Up
chr6_88358642_88355483_-3159-Eefsec	2.993706	0.032088	EEFSEC	Up
chr5_123631169_123590793_-40376-Clip1	2.993706	0.032088	CLIP1	Up
chr6_38491931_38440455_+51476-Ubn2	2.581961	0.018557	UBN2	Up
chr12_51661713_51647994_-13719-Strn3	2.220505	0.029079	STRN3	Up
chr12_16568519_16560982_-7537-Lpin1	2.11363	0.042959	LPIN1	Up
chr7_132950435_132948044_+2391-Zranb1	2.069291	0.022835	ZRANB1	Up
chr2_20860036_20854935_-5101-Arhgap21	1.999227	0.021494	ARHGAP21	Up
chr2_114058656_114050289_-8367-Actc1	-2.14554	0.018028	ACTC1	Down
chr11_67246541_67181035_+65506-Myh1	-2.30153	0.039703	MYH1	Down
chr7_29043929_29040388_-3541-Ryr1	-2.31905	0.047472	RYR1	Down
chr3_51326035_51299783_-26252-Elf2	-2.41309	0.013261	ELF2	Down
chr16_32961744_32950292_+11452-Lrch3	-2.45649	0.029717	LRCH3	Down
chr1_91108672_91088634_+20038-Lrrfip1	-2.58296	0.006091	LRRFIP1	Down
chr2_163376375_163375604_-771-Jph2	-3.16353	0.017703	JPH2	Down
chr11_67297622_67252555_+45067-Myh8	-3.28732	9.31E-07	MYH8	Down
chr1_151478625_151476140_-2485-Rnf2	-9.96723	0.036493	RNF2	Down
chr2_132558682_132544392_-14290-Gpcpd1	-9.96723	0.036493	GPCPD1	Down
chr15_100476886_100469685_+7201-Letmd1	-9.96723	0.036493	LETMD1	Down
chr9_77230954_77164765_-66189-Mlip	-9.96723	0.036493	MLIP	Down
chr15_82857284_82825231_-32053-Tcf20	-10.23	0.015841	TCF20	Down
chr9_56147578_56145489_-2089-Tspan3	-10.23	0.015841	TSPAN3	Down
chr18_80917878_80858544_-59334-Atp9b	-10.23	0.015841	ATP9b	Down
chr6_88298236_88281549_-16687-Eefsec	-10.23	0.015841	EEFSEC	Down
chr17_25218755_25199767_+18988-Unkl	-10.23	0.015841	UNKL	Down
chr6_134513419_134506196_-7223-Lrp6	-10.4522	0.007065	LRP6	Down
chr2_76897400_76897122_-278-Ttn	-10.4522	0.007065	TTN	Down
chr9_22887996_22887581_+415-Bbs9	-10.9665	0.000753	BBS9	Down
chr2_52303735_52302442_-1293-Neb	-11.8922	3.35E-06	NEB	Down

Supplementary Table 3. The differential circRNA profiles in Qu muscles of aging mice with aerobic exercise compared with aging mice.

ID	Log2FC	P-Value	GeneName	Type
chr18_80917878_80858544_-59334-Atp9b	11.05142672	0.000399475	ATP9b	Up
chr1_125579327_125568547_+10780-Slc35f5	10.72966856	0.00216554	SLC35F5	Up
chr9_22887996_22887581_+415-Bbs9	10.05210633	0.034667196	BBS9	Up
chr1_93576112_93567417_+8695-Farp2	10.05210633	0.034667196	FARP2	Up
chr9_110068985_110063017_+5968-Map4	10.05210633	0.034667196	MAP4	Up
chr8_33611921_33610655_+1266-Ppp2cb	10.05210633	0.034667196	PPP2CB	Up
chr11_67191466_67096301_+95165-Myh2	2.46165636	0.00035862	MYH2	Up
chr11_67297622_67252555_+45067-Myh8	2.278621188	0.001506383	MYH8	Up
chr1_172187460_172173943_+13517-Dcaf8	2.167943537	0.04730565	DCAF8	Up
chr11_67204463_67176537_+27926-Myh2	2.08301854	0.008370838	MYH2	Up
chr5_103886290_103885907_-383-Klhl8	-3.283417049	0.012402727	KLHL8	Down
chr4_9610938_9583812_-27126-Asph	-10.1451308	0.033703803	ASPH	Down
chr1_92148494_92141520_-6974-Hdac4	-10.1451308	0.033703803	HDAC4	Down
chr9_42451762_42437115_-14647-Tbcel	-10.1451308	0.033703803	TBCEL	Down
chr17_71810982_71800799_+10183-Clip4	-10.1451308	0.033703803	CLIP4	Down
chr15_3551685_3388648_-163037-Ghr	-10.1451308	0.033703803	GHR	Down
chr19_42564341_42562552_+1789-R3hcc1l	-10.1451308	0.033703803	R3HCC1L	Down
chr1_185267279_185266901_+378-Rab3gap2	-10.1451308	0.033703803	RAB3GAP2	Down
chr3_5245645_5241670_+3975-Zfhx4	-10.3673412	0.015337107	ZFHX4	Down
chr8_120553802_120553584_+218-Gse1	-10.88158669	0.001656524	GSE1	Down
chr11_59061687_59055377_-6310-Obscn	-16.1991578	0	OBSCN	Down

Supplementary Table 4. Overlapped circRNA featured opposite expression pattern among groups of young, aging, aging with exercise intervention.

ID	Aging vs Young (Log2FC)	Aging vs Young (Fold Change)	Type	Exercise vs Aging (Log2FC)	Exercise vs Aging (Fold Change)	Type
chr11_67297622_67252555 _+45067-MYH8	-3.287322706	0.102427662	down	2.278621188	4.852140037	up
chr18_80917878_80858544 _-59334-ATP9b	-10.23002044	0.000832639	down	11.05142672	2122.320344	up
chr9_22887996_22887581_ +415-BBS9	-10.96650545	0.00049975	down	10.05210633	1061.660172	up

Supplementary Table 6. List of primers for Real time PCR, conventional RT-PCR and plasmid construction.

**Real time PCR primers**		
**Gene**	**Forward primer**	**Reverse primer**
m-Foxo3	TTCAACAGTACCGTGTTTGGAC	AGTGTGACACGGAAGAGAAGGT
m-Atrogin	AGCGCTTCTTGGATGAGAAA	GGCTGCTGAACAGATTCTCC
m-Pgc1α	ACCATGACTACTGTCAGTCACTC	GTCACAGGAGGCATCTTTGAAG
m-Mfn1	AACCGAGAAGCTGCAGATGA	TCAACTTGTTGGCACAGTCG
m-Atpase	GACATGGGCACAATGCAGG	GCAGGGTCAGTCAGGTCATCA
m-Gapdh	ACAACTTTGGCATTGTGGAA	GATGCAGGGATGATGTTCTG
CircBBS9	AATGAGTTGAGGGGAGAGGC	CTGGGATTGGAGTACAGCCA
CircATP9b	AGGCCTTTCTGTCTTGTGGT	AATGGGCAGACTCATCCTCC
CircMYH8	AGGCAGAGGAGGACAAAGTC	GACTCTTGGGCCAGTTTCAG
m-Dnmt3a	GAGGGAACTGAGACCCCAC	CTGGAAGGTGAGTCTTGGCA
m-Adcy1	GTCACCTTCGTGTCCTATGCC	TTCACACCAAAGAAGAGCAGG
m-Cacna1e	GATGGAGACTCGGACCAGAG	TGACCGTGAAACAGTTCTGCC
m-Adcyap1r1	CTGCGTGCAGAAATGCTACTG	AGCCGTAGAGTAATGGTGGATAG
m-Ctnnd1	GTGGAAACCTACACCGAGGAG	CGTCTAGTGGTCCCATCATCTG
m-Dad1	TGAAGTTGCTGGACGCCTATC	AAGCCAGAGAGGAACGAGTTG
m-Gys1	GAACGCAGTGCTTTTCGAGG	CCAGATAGTAGTTGTCACCCCAT
m-18s	GGGAGCCTGAGAAACGGC	GGGTCGGGAGTGGGTAATTT
**RT PCR primers for validation**		
**CircBBS9**	**Forward primer**	**Reverse primer**
Divergent	AATGAGTTGAGGGGAGAGGC	CTGGGATTGGAGTACAGCCA
Convergent	ACAAGCACCTCATGACCGAG	CCAGAGATGAGCTTGGCACA
**Clone PCR primers**		
**Gene**	**Forward primer**	**Reverse primer**
Clone-CircBBS9	CGGAATTCTGAAATATGCTATCTTACA<br>GGTGGCTGTACTCCAATCCCAGAG	GGAATTCCATATGTCAAGAAAAAATATA<br>TTCACTCCAGAGATGAGCTTGGCACAGC
