# Supplementary material for: Comprehensive analysis of circular RNA profiles in skeletal muscles of aging mice and after aerobic exercise intervention
Source: Aging (Albany NY). 2020 Mar 17;12(6):5071–90. doi: 10.18632/aging.102932 (PMC7138574; doi:10.18632/aging.102932)
Supplement: Supplementary Table 5 [file aging-12-102932-s002..docx]

**Supplementary Table 5. circBBS9-miRNA-mRNA network.**

| **SUID** | **AverageShortest Path Length** | **Betweenness Centrality** | **Closeness Centrality** | **Degree** | **Eccentricity** | **Name** | **Neighborhood Connectivity** | **Number Of Directed Edges** | **Radiality** | **Stress** | **Topological Coefficient** |
| --- | --- | --- | --- | --- | --- | --- | --- | --- | --- | --- | --- |
| 1629 | 1.79451181 | 0.12328847 | 0.55725462 | 940 | 3 | mmu-miR-7662-5p | 5.54361702 | 940 | 0.80137205 | 395169466 | 0.50484634 |
| 1631 | 1.79451181 | 0.12328847 | 0.55725462 | 940 | 3 | mmu-miR-7028-5p | 5.54361702 | 940 | 0.80137205 | 395169466 | 0.50484634 |
| 1634 | 1.79451181 | 0.12328847 | 0.55725462 | 940 | 3 | mmu-miR-6911-5p | 5.54361702 | 940 | 0.80137205 | 395169466 | 0.50484634 |
| 1630 | 1.79578813 | 0.12286357 | 0.55685856 | 939 | 3 | mmu-miR-7079-5p | 5.54632588 | 939 | 0.80105297 | 393999520 | 0.50514732 |
| 1639 | 1.85322272 | 0.22756139 | 0.53960055 | 894 | 3 | mmu-miR-383-3p | 4.78411633 | 894 | 0.78669432 | 611439218 | 0.42045737 |
| 1637 | 2.07657945 | 0.1440846 | 0.48156116 | 719 | 3 | mmu-miR-3065-3p | 4.71210014 | 719 | 0.73085514 | 407356560 | 0.41245557 |
| 1636 | 2.12763242 | 0.12981956 | 0.470006 | 679 | 3 | mmu-miR-3100-5p | 4.75846834 | 679 | 0.7180919 | 370236252 | 0.41760759 |
| 1633 | 2.25654116 | 0.09327697 | 0.44315611 | 578 | 3 | mmu-miR-6930-3p | 4.75259516 | 578 | 0.68586471 | 273918628 | 0.41695502 |
| 1632 | 2.32673899 | 0.07909644 | 0.42978607 | 523 | 3 | mmu-miR-7020-5p | 4.87189293 | 523 | 0.66831525 | 239653558 | 0.43021033 |
| 1635 | 2.82705807 | 0.00533321 | 0.3537246 | 131 | 3 | mmu-miR-423-3p | 5.21374046 | 131 | 0.54323548 | 17996446 | 0.46819338 |
| 1627 | 1.99361838 | 6.75E-04 | 0.50160051 | 10 | 2 | Hic2 | 728.3 | 10 | 0.75159541 | 6253212 | 0.46711625 |
| 1628 | 1.99361838 | 6.75E-04 | 0.50160051 | 10 | 2 | Fbxo41 | 728.3 | 10 | 0.75159541 | 6253212 | 0.46711625 |
| 1638 | 1.99361838 | 6.75E-04 | 0.50160051 | 10 | 2 | Dnmt3a | 728.3 | 10 | 0.75159541 | 6253212 | 0.46711625 |
| 1608 | 1.9948947 | 6.18E-04 | 0.50127959 | 9 | 3 | Mapkbp1 | 794.6666667 | 9 | 0.75127632 | 5928388 | 0.50974095 |
| 1609 | 1.99617103 | 5.27E-04 | 0.50095908 | 9 | 4 | Kcng4 | 729.3333333 | 9 | 0.75095724 | 4849582 | 0.46808055 |
| 1610 | 1.9948947 | 6.18E-04 | 0.50127959 | 9 | 3 | Igf2bp1 | 794.6666667 | 9 | 0.75127632 | 5928388 | 0.50974095 |
| 1611 | 1.9948947 | 6.18E-04 | 0.50127959 | 9 | 3 | H13 | 794.6666667 | 9 | 0.75127632 | 5928388 | 0.50974095 |
| 1612 | 1.99617103 | 5.27E-04 | 0.50095908 | 9 | 4 | Fign | 729.3333333 | 9 | 0.75095724 | 4849582 | 0.46808055 |
| 1613 | 1.99617103 | 5.27E-04 | 0.50095908 | 9 | 4 | Endov | 729.3333333 | 9 | 0.75095724 | 4849582 | 0.46808055 |
| 1614 | 1.9948947 | 5.17E-04 | 0.50127959 | 9 | 3 | Dpy19l3 | 709.8888889 | 9 | 0.75127632 | 4709398 | 0.45529152 |
| 1615 | 1.9948947 | 6.18E-04 | 0.50127959 | 9 | 3 | Dhdds | 794.6666667 | 9 | 0.75127632 | 5928388 | 0.50974095 |
| 1616 | 1.9948947 | 6.18E-04 | 0.50127959 | 9 | 3 | Cnr2 | 794.6666667 | 9 | 0.75127632 | 5928388 | 0.50974095 |
| 1617 | 1.9948947 | 6.18E-04 | 0.50127959 | 9 | 3 | Cbx4 | 794.6666667 | 9 | 0.75127632 | 5928388 | 0.50974095 |
| 1618 | 1.9948947 | 6.18E-04 | 0.50127959 | 9 | 3 | Cacna1e | 794.6666667 | 9 | 0.75127632 | 5928388 | 0.50974095 |
| 1619 | 1.9948947 | 5.50E-04 | 0.50127959 | 9 | 3 | Bcl2l13 | 745 | 9 | 0.75127632 | 5071236 | 0.477842 |
| 1620 | 1.9948947 | 6.18E-04 | 0.50127959 | 9 | 3 | Arid5a | 794.6666667 | 9 | 0.75127632 | 5928388 | 0.50974095 |
| 1621 | 1.99617103 | 5.27E-04 | 0.50095908 | 9 | 4 | Ago2 | 729.3333333 | 9 | 0.75095724 | 4849582 | 0.46808055 |
| 1622 | 1.9948947 | 6.18E-04 | 0.50127959 | 9 | 3 | Aff4 | 794.6666667 | 9 | 0.75127632 | 5928388 | 0.50974095 |
| 1623 | 1.9948947 | 5.50E-04 | 0.50127959 | 9 | 3 | Adrbk2 | 745 | 9 | 0.75127632 | 5071236 | 0.477842 |
| 1624 | 1.99617103 | 5.27E-04 | 0.50095908 | 9 | 4 | Adcy1 | 729.3333333 | 9 | 0.75095724 | 4849582 | 0.46808055 |
| 1625 | 1.9948947 | 6.18E-04 | 0.50127959 | 9 | 3 | Aak1 | 794.6666667 | 9 | 0.75127632 | 5928388 | 0.50974095 |
| 1541 | 1.99617103 | 5.06E-04 | 0.50095908 | 8 | 3 | Mapre1 | 828.625 | 8 | 0.75095724 | 4881882 | 0.53155106 |
| 1542 | 1.99617103 | 4.64E-04 | 0.50095908 | 8 | 3 | Map1a | 782.25 | 8 | 0.75095724 | 4402554 | 0.50176622 |
| 1543 | 1.99617103 | 4.95E-04 | 0.50095908 | 8 | 3 | Luc7l | 821.75 | 8 | 0.75095724 | 4760636 | 0.52713552 |
| 1544 | 1.99617103 | 4.95E-04 | 0.50095908 | 8 | 3 | Lpp | 821.75 | 8 | 0.75095724 | 4760636 | 0.52713552 |
| 1545 | 1.99617103 | 4.29E-04 | 0.50095908 | 8 | 3 | Lpin1 | 760.125 | 8 | 0.75095724 | 3939126 | 0.4875562 |
| 1546 | 1.99617103 | 4.95E-04 | 0.50095908 | 8 | 3 | LOC101055680 | 821.75 | 8 | 0.75095724 | 4760636 | 0.52713552 |
| 1547 | 1.99617103 | 4.95E-04 | 0.50095908 | 8 | 3 | Lman2 | 821.75 | 8 | 0.75095724 | 4760636 | 0.52713552 |
| 1548 | 1.99744735 | 4.17E-04 | 0.50063898 | 8 | 4 | Lif | 755.125 | 8 | 0.75063816 | 3840074 | 0.48465617 |
| 1549 | 1.99617103 | 4.95E-04 | 0.50095908 | 8 | 3 | Lep | 821.75 | 8 | 0.75095724 | 4760636 | 0.52713552 |
| 1550 | 1.99617103 | 4.29E-04 | 0.50095908 | 8 | 3 | Ky | 760.125 | 8 | 0.75095724 | 3939126 | 0.4875562 |
| 1551 | 1.99617103 | 4.95E-04 | 0.50095908 | 8 | 3 | Kmt2a | 821.75 | 8 | 0.75095724 | 4760636 | 0.52713552 |
| 1552 | 1.99617103 | 4.95E-04 | 0.50095908 | 8 | 3 | Kif21b | 821.75 | 8 | 0.75095724 | 4760636 | 0.52713552 |
| 1553 | 1.99744735 | 4.73E-04 | 0.50063898 | 8 | 4 | Kif1a | 804.125 | 8 | 0.75063816 | 4539776 | 0.51614717 |
| 1554 | 1.99617103 | 5.06E-04 | 0.50095908 | 8 | 3 | Kdm4a | 828.625 | 8 | 0.75095724 | 4881882 | 0.53155106 |
| 1555 | 1.99617103 | 4.95E-04 | 0.50095908 | 8 | 3 | Kcnv2 | 821.75 | 8 | 0.75095724 | 4760636 | 0.52713552 |
| 1556 | 1.99744735 | 4.73E-04 | 0.50063898 | 8 | 4 | Kcnb1 | 804.125 | 8 | 0.75063816 | 4539776 | 0.51614717 |
| 1557 | 1.99617103 | 4.84E-04 | 0.50095908 | 8 | 3 | Kcna2 | 809.125 | 8 | 0.75095724 | 4636774 | 0.51902697 |
| 1558 | 1.99617103 | 4.84E-04 | 0.50095908 | 8 | 3 | Kcna1 | 809.125 | 8 | 0.75095724 | 4636774 | 0.51902697 |
| 1559 | 1.99744735 | 4.73E-04 | 0.50063898 | 8 | 4 | Kazn | 804.125 | 8 | 0.75063816 | 4539776 | 0.51614717 |
| 1560 | 1.99617103 | 5.06E-04 | 0.50095908 | 8 | 3 | Jakmip3 | 828.625 | 8 | 0.75095724 | 4881882 | 0.53155106 |
| 1561 | 1.99617103 | 4.95E-04 | 0.50095908 | 8 | 3 | Ildr2 | 821.75 | 8 | 0.75095724 | 4760636 | 0.52713552 |
| 1562 | 1.99617103 | 5.06E-04 | 0.50095908 | 8 | 3 | Iffo2 | 828.625 | 8 | 0.75095724 | 4881882 | 0.53155106 |
| 1563 | 1.99617103 | 4.84E-04 | 0.50095908 | 8 | 3 | Hs3st3b1 | 809.125 | 8 | 0.75095724 | 4636774 | 0.51902697 |
| 1564 | 1.99617103 | 4.18E-04 | 0.50095908 | 8 | 3 | Hrk | 753.25 | 8 | 0.75095724 | 3814004 | 0.48314066 |
| 1565 | 1.99617103 | 5.06E-04 | 0.50095908 | 8 | 3 | Homer2 | 828.625 | 8 | 0.75095724 | 4881882 | 0.53155106 |
| 1566 | 1.99617103 | 4.95E-04 | 0.50095908 | 8 | 3 | Hjurp | 821.75 | 8 | 0.75095724 | 4760636 | 0.52713552 |
| 1567 | 1.99744735 | 3.95E-04 | 0.50063898 | 8 | 4 | Gys1 | 735.625 | 8 | 0.75063816 | 3591332 | 0.47212404 |
| 1568 | 1.99617103 | 4.64E-04 | 0.50095908 | 8 | 3 | Gucy2e | 782.25 | 8 | 0.75095724 | 4402554 | 0.50176622 |
| 1569 | 1.99744735 | 4.06E-04 | 0.50063898 | 8 | 4 | Gse1 | 748.25 | 8 | 0.75063816 | 3713742 | 0.48023779 |
| 1570 | 1.99617103 | 5.06E-04 | 0.50095908 | 8 | 3 | Grm1 | 828.625 | 8 | 0.75095724 | 4881882 | 0.53155106 |
| 1571 | 1.99617103 | 5.06E-04 | 0.50095908 | 8 | 3 | Grik3 | 828.625 | 8 | 0.75095724 | 4881882 | 0.53155106 |
| 1572 | 1.99744735 | 4.73E-04 | 0.50063898 | 8 | 4 | Gprc5c | 804.125 | 8 | 0.75063816 | 4539776 | 0.51614717 |
| 1573 | 1.99617103 | 4.84E-04 | 0.50095908 | 8 | 3 | Fzd3 | 809.125 | 8 | 0.75095724 | 4636774 | 0.51902697 |
| 1574 | 1.99617103 | 5.06E-04 | 0.50095908 | 8 | 3 | Fbrs | 828.625 | 8 | 0.75095724 | 4881882 | 0.53155106 |
| 1575 | 1.99744735 | 4.06E-04 | 0.50063898 | 8 | 4 | Fam198a | 748.25 | 8 | 0.75063816 | 3713742 | 0.48023779 |
| 1576 | 1.99617103 | 4.95E-04 | 0.50095908 | 8 | 3 | Fam168b | 821.75 | 8 | 0.75095724 | 4760636 | 0.52713552 |
| 1577 | 1.99617103 | 4.84E-04 | 0.50095908 | 8 | 3 | Faim2 | 809.125 | 8 | 0.75095724 | 4636774 | 0.51902697 |
| 1578 | 1.99617103 | 5.06E-04 | 0.50095908 | 8 | 3 | Exoc8 | 828.625 | 8 | 0.75095724 | 4881882 | 0.53155106 |
| 1579 | 1.99744735 | 3.95E-04 | 0.50063898 | 8 | 4 | Elavl3 | 735.625 | 8 | 0.75063816 | 3591332 | 0.47212404 |
| 1580 | 1.99617103 | 5.06E-04 | 0.50095908 | 8 | 3 | Dnajc1 | 828.625 | 8 | 0.75095724 | 4881882 | 0.53155106 |
| 1581 | 1.99617103 | 4.95E-04 | 0.50095908 | 8 | 3 | Dido1 | 821.75 | 8 | 0.75095724 | 4760636 | 0.52713552 |
| 1582 | 1.99617103 | 4.95E-04 | 0.50095908 | 8 | 3 | Dad1 | 821.75 | 8 | 0.75095724 | 4760636 | 0.52713552 |
| 1583 | 1.99617103 | 4.95E-04 | 0.50095908 | 8 | 3 | Ctnnd1 | 821.75 | 8 | 0.75095724 | 4760636 | 0.52713552 |
| 1584 | 1.99744735 | 3.73E-04 | 0.50063898 | 8 | 4 | Ctif | 708.75 | 8 | 0.75063816 | 3353178 | 0.45485219 |
| 1585 | 1.99617103 | 4.95E-04 | 0.50095908 | 8 | 3 | Ctdsp2 | 821.75 | 8 | 0.75095724 | 4760636 | 0.52713552 |
| 1586 | 1.99744735 | 3.95E-04 | 0.50063898 | 8 | 4 | Csnk1g1 | 735.625 | 8 | 0.75063816 | 3591332 | 0.47212404 |
| 1587 | 1.99617103 | 4.84E-04 | 0.50095908 | 8 | 3 | Cntn2 | 809.125 | 8 | 0.75095724 | 4636774 | 0.51902697 |
| 1588 | 1.99617103 | 4.95E-04 | 0.50095908 | 8 | 3 | Chst2 | 821.75 | 8 | 0.75095724 | 4760636 | 0.52713552 |
| 1589 | 1.99617103 | 4.84E-04 | 0.50095908 | 8 | 3 | Chid1 | 809.125 | 8 | 0.75095724 | 4636774 | 0.51902697 |
| 1590 | 1.99617103 | 5.06E-04 | 0.50095908 | 8 | 3 | Cenpo | 828.625 | 8 | 0.75095724 | 4881882 | 0.53155106 |
| 1591 | 1.99617103 | 4.84E-04 | 0.50095908 | 8 | 3 | Cdyl2 | 809.125 | 8 | 0.75095724 | 4636774 | 0.51902697 |
| 1592 | 1.99617103 | 5.06E-04 | 0.50095908 | 8 | 3 | Cds2 | 828.625 | 8 | 0.75095724 | 4881882 | 0.53155106 |
| 1593 | 1.99617103 | 5.06E-04 | 0.50095908 | 8 | 3 | Cd2ap | 828.625 | 8 | 0.75095724 | 4881882 | 0.53155106 |
| 1594 | 1.99617103 | 4.29E-04 | 0.50095908 | 8 | 3 | Casz1 | 760.125 | 8 | 0.75095724 | 3939126 | 0.4875562 |
| 1595 | 1.99617103 | 5.06E-04 | 0.50095908 | 8 | 3 | Capn5 | 828.625 | 8 | 0.75095724 | 4881882 | 0.53155106 |
| 1596 | 1.99617103 | 4.95E-04 | 0.50095908 | 8 | 3 | Btbd9 | 821.75 | 8 | 0.75095724 | 4760636 | 0.52713552 |
| 1597 | 1.99744735 | 4.17E-04 | 0.50063898 | 8 | 4 | Atxn1 | 755.125 | 8 | 0.75063816 | 3840074 | 0.48465617 |
| 1598 | 1.99617103 | 4.95E-04 | 0.50095908 | 8 | 3 | Atp6v0e2 | 821.75 | 8 | 0.75095724 | 4760636 | 0.52713552 |
| 1599 | 1.99617103 | 5.06E-04 | 0.50095908 | 8 | 3 | Arrb1 | 828.625 | 8 | 0.75095724 | 4881882 | 0.53155106 |
| 1600 | 1.99617103 | 4.95E-04 | 0.50095908 | 8 | 3 | Ap1g1 | 821.75 | 8 | 0.75095724 | 4760636 | 0.52713552 |
| 1601 | 1.99617103 | 4.95E-04 | 0.50095908 | 8 | 3 | Ano5 | 821.75 | 8 | 0.75095724 | 4760636 | 0.52713552 |
| 1602 | 1.99744735 | 3.95E-04 | 0.50063898 | 8 | 4 | Anapc11 | 735.625 | 8 | 0.75063816 | 3591332 | 0.47212404 |
| 1603 | 1.99617103 | 5.06E-04 | 0.50095908 | 8 | 3 | Afap1 | 828.625 | 8 | 0.75095724 | 4881882 | 0.53155106 |
| 1604 | 1.99617103 | 4.84E-04 | 0.50095908 | 8 | 3 | Adcyap1r1 | 809.125 | 8 | 0.75095724 | 4636774 | 0.51902697 |
| 1605 | 1.99617103 | 4.39E-04 | 0.50095908 | 8 | 3 | 3110062M04Rik | 772.75 | 8 | 0.75095724 | 4055930 | 0.49566474 |
| 1606 | 1.99617103 | 5.06E-04 | 0.50095908 | 8 | 3 | 2900026A02Rik | 828.625 | 8 | 0.75095724 | 4881882 | 0.53155106 |
| 1607 | 1.99617103 | 4.95E-04 | 0.50095908 | 8 | 3 | 1700025G04Rik | 821.75 | 8 | 0.75095724 | 4760636 | 0.52713552 |
| 1413 | 2.00638162 | 3.44E-04 | 0.49840967 | 7 | 4 | Mark2 | 822 | 7 | 0.74840459 | 3296052 | 0.52967742 |
| 1414 | 2.00127632 | 3.67E-04 | 0.49968112 | 7 | 4 | Mapt | 842.1428571 | 7 | 0.74968092 | 3517930 | 0.54127597 |
| 1415 | 1.99872368 | 3.88E-04 | 0.50031928 | 7 | 4 | Map3k9 | 864.4285714 | 7 | 0.75031908 | 3757504 | 0.55490268 |
| 1416 | 1.99872368 | 3.88E-04 | 0.50031928 | 7 | 4 | Map2k4 | 864.4285714 | 7 | 0.75031908 | 3757504 | 0.55490268 |
| 1417 | 2.00893427 | 3.24E-04 | 0.49777637 | 7 | 4 | Lyg2 | 791.2857143 | 7 | 0.74776643 | 3061352 | 0.51052049 |
| 1418 | 2.00255265 | 3.77E-04 | 0.49936265 | 7 | 4 | Lsm11 | 850 | 7 | 0.74936184 | 3641002 | 0.54668384 |
| 1419 | 2.00638162 | 3.44E-04 | 0.49840967 | 7 | 4 | Lrpap1 | 822 | 7 | 0.74840459 | 3296052 | 0.52967742 |
| 1420 | 2.00127632 | 3.67E-04 | 0.49968112 | 7 | 4 | Lrp4 | 842.1428571 | 7 | 0.74968092 | 3517930 | 0.54127597 |
| 1421 | 1.99872368 | 3.88E-04 | 0.50031928 | 7 | 4 | Lpgat1 | 864.4285714 | 7 | 0.75031908 | 3757504 | 0.55490268 |
| 1422 | 2.00255265 | 3.77E-04 | 0.49936265 | 7 | 4 | Lpar2 | 850 | 7 | 0.74936184 | 3641002 | 0.54668384 |
| 1423 | 2.01786854 | 3.12E-04 | 0.49557242 | 7 | 4 | Lnx2 | 786.1428571 | 7 | 0.74553287 | 2849432 | 0.50950218 |
| 1424 | 1.99872368 | 3.88E-04 | 0.50031928 | 7 | 4 | Lifr | 864.4285714 | 7 | 0.75031908 | 3757504 | 0.55490268 |
| 1425 | 2.00255265 | 3.77E-04 | 0.49936265 | 7 | 4 | Kdm2a | 850 | 7 | 0.74936184 | 3641002 | 0.54668384 |
| 1426 | 2.08551372 | 2.57E-04 | 0.47949816 | 7 | 4 | Kcnq5 | 727.4285714 | 7 | 0.72862157 | 2264282 | 0.48819124 |
| 1427 | 2.00255265 | 3.77E-04 | 0.49936265 | 7 | 4 | Kcnj12 | 850 | 7 | 0.74936184 | 3641002 | 0.54668384 |
| 1428 | 2.0051053 | 3.36E-04 | 0.49872693 | 7 | 4 | Kcnj1 | 797 | 7 | 0.74872368 | 3167038 | 0.51321728 |
| 1429 | 2.00127632 | 3.67E-04 | 0.49968112 | 7 | 4 | Kcnc1 | 842.1428571 | 7 | 0.74968092 | 3517930 | 0.54127597 |
| 1430 | 1.99872368 | 3.88E-04 | 0.50031928 | 7 | 4 | Irgq | 864.4285714 | 7 | 0.75031908 | 3757504 | 0.55490268 |
| 1431 | 2.00127632 | 3.67E-04 | 0.49968112 | 7 | 4 | Irf2 | 842.1428571 | 7 | 0.74968092 | 3517930 | 0.54127597 |
| 1432 | 2.00638162 | 3.44E-04 | 0.49840967 | 7 | 4 | Iqsec2 | 822 | 7 | 0.74840459 | 3296052 | 0.52967742 |
| 1433 | 2.0051053 | 3.66E-04 | 0.49872693 | 7 | 4 | Ipcef1 | 844.2857143 | 7 | 0.74872368 | 3542442 | 0.54370452 |
| 1434 | 2.00127632 | 3.67E-04 | 0.49968112 | 7 | 4 | Ino80 | 842.1428571 | 7 | 0.74968092 | 3517930 | 0.54127597 |
| 1435 | 2.00127632 | 3.55E-04 | 0.49968112 | 7 | 4 | Ing5 | 836.4285714 | 7 | 0.74968092 | 3418160 | 0.53759882 |
| 1436 | 2.00638162 | 3.44E-04 | 0.49840967 | 7 | 4 | Igfbp5 | 822 | 7 | 0.74840459 | 3296052 | 0.52967742 |
| 1437 | 1.99872368 | 3.88E-04 | 0.50031928 | 7 | 4 | Ifitm10 | 864.4285714 | 7 | 0.75031908 | 3757504 | 0.55490268 |
| 1438 | 1.99872368 | 3.88E-04 | 0.50031928 | 7 | 4 | Htt | 864.4285714 | 7 | 0.75031908 | 3757504 | 0.55490268 |
| 1439 | 2.00255265 | 3.77E-04 | 0.49936265 | 7 | 4 | Hif3a | 850 | 7 | 0.74936184 | 3641002 | 0.54668384 |
| 1440 | 1.99872368 | 3.88E-04 | 0.50031928 | 7 | 4 | Helb | 864.4285714 | 7 | 0.75031908 | 3757504 | 0.55490268 |
| 1441 | 2.00255265 | 3.77E-04 | 0.49936265 | 7 | 4 | Hbs1l | 850 | 7 | 0.74936184 | 3641002 | 0.54668384 |
| 1442 | 2.00255265 | 3.77E-04 | 0.49936265 | 7 | 4 | Gxylt1 | 850 | 7 | 0.74936184 | 3641002 | 0.54668384 |
| 1443 | 2.0051053 | 3.66E-04 | 0.49872693 | 7 | 4 | Grm6 | 844.2857143 | 7 | 0.74872368 | 3542442 | 0.54370452 |
| 1444 | 1.99872368 | 3.88E-04 | 0.50031928 | 7 | 4 | Grin3a | 864.4285714 | 7 | 0.75031908 | 3757504 | 0.55490268 |
| 1445 | 2.00127632 | 3.46E-04 | 0.49968112 | 7 | 4 | Gpsm1 | 811.4285714 | 7 | 0.74968092 | 3281746 | 0.52151131 |
| 1446 | 1.99872368 | 3.88E-04 | 0.50031928 | 7 | 4 | Gpr146 | 864.4285714 | 7 | 0.75031908 | 3757504 | 0.55490268 |
| 1447 | 2.00127632 | 3.46E-04 | 0.49968112 | 7 | 4 | Gnao1 | 811.4285714 | 7 | 0.74968092 | 3281746 | 0.52151131 |
| 1448 | 1.99872368 | 3.88E-04 | 0.50031928 | 7 | 4 | Gna13 | 864.4285714 | 7 | 0.75031908 | 3757504 | 0.55490268 |
| 1449 | 1.99872368 | 3.88E-04 | 0.50031928 | 7 | 4 | Gm20604 | 864.4285714 | 7 | 0.75031908 | 3757504 | 0.55490268 |
| 1450 | 1.99872368 | 3.88E-04 | 0.50031928 | 7 | 4 | Gltscr1l | 864.4285714 | 7 | 0.75031908 | 3757504 | 0.55490268 |
| 1451 | 1.99872368 | 3.88E-04 | 0.50031928 | 7 | 4 | Gjd2 | 864.4285714 | 7 | 0.75031908 | 3757504 | 0.55490268 |
| 1452 | 2.00127632 | 3.67E-04 | 0.49968112 | 7 | 4 | Gigyf1 | 842.1428571 | 7 | 0.74968092 | 3517930 | 0.54127597 |
| 1453 | 2.0051053 | 3.66E-04 | 0.49872693 | 7 | 4 | Gdf2 | 844.2857143 | 7 | 0.74872368 | 3542442 | 0.54370452 |
| 1454 | 2.0051053 | 3.66E-04 | 0.49872693 | 7 | 4 | Gcnt4 | 844.2857143 | 7 | 0.74872368 | 3542442 | 0.54370452 |
| 1455 | 2.00255265 | 3.77E-04 | 0.49936265 | 7 | 4 | Galnt6 | 850 | 7 | 0.74936184 | 3641002 | 0.54668384 |
| 1456 | 2.0051053 | 3.36E-04 | 0.49872693 | 7 | 4 | Gad2 | 797 | 7 | 0.74872368 | 3167038 | 0.51321728 |
| 1457 | 2.00255265 | 3.77E-04 | 0.49936265 | 7 | 4 | Fyttd1 | 850 | 7 | 0.74936184 | 3641002 | 0.54668384 |
| 1458 | 2.00255265 | 3.77E-04 | 0.49936265 | 7 | 4 | Foxj2 | 850 | 7 | 0.74936184 | 3641002 | 0.54668384 |
| 1459 | 2.00255265 | 3.77E-04 | 0.49936265 | 7 | 4 | Fibcd1 | 850 | 7 | 0.74936184 | 3641002 | 0.54668384 |
| 1460 | 2.0051053 | 3.57E-04 | 0.49872693 | 7 | 4 | Fermt1 | 819.2857143 | 7 | 0.74872368 | 3411330 | 0.52758589 |
| 1461 | 2.0051053 | 3.57E-04 | 0.49872693 | 7 | 4 | Fem1a | 819.2857143 | 7 | 0.74872368 | 3411330 | 0.52758589 |
| 1462 | 2.00255265 | 3.77E-04 | 0.49936265 | 7 | 4 | Fbxo25 | 850 | 7 | 0.74936184 | 3641002 | 0.54668384 |
| 1463 | 2.0051053 | 3.66E-04 | 0.49872693 | 7 | 4 | Fbxl17 | 844.2857143 | 7 | 0.74872368 | 3542442 | 0.54370452 |
| 1464 | 2.0051053 | 3.66E-04 | 0.49872693 | 7 | 4 | Fat2 | 844.2857143 | 7 | 0.74872368 | 3542442 | 0.54370452 |
| 1465 | 2.00127632 | 3.46E-04 | 0.49968112 | 7 | 4 | Fam163a | 811.4285714 | 7 | 0.74968092 | 3281746 | 0.52151131 |
| 1466 | 2.00127632 | 3.67E-04 | 0.49968112 | 7 | 4 | Fam102a | 842.1428571 | 7 | 0.74968092 | 3517930 | 0.54127597 |
| 1467 | 1.99872368 | 3.88E-04 | 0.50031928 | 7 | 4 | Extl3 | 864.4285714 | 7 | 0.75031908 | 3757504 | 0.55490268 |
| 1468 | 2.0051053 | 3.66E-04 | 0.49872693 | 7 | 4 | Ept1 | 844.2857143 | 7 | 0.74872368 | 3542442 | 0.54370452 |
| 1469 | 2.00638162 | 3.44E-04 | 0.49840967 | 7 | 4 | Entpd6 | 822 | 7 | 0.74840459 | 3296052 | 0.52967742 |
| 1470 | 2.0051053 | 3.66E-04 | 0.49872693 | 7 | 4 | Elmsan1 | 844.2857143 | 7 | 0.74872368 | 3542442 | 0.54370452 |
| 1471 | 2.01914486 | 3.01E-04 | 0.49525917 | 7 | 4 | Ehd3 | 780.4285714 | 7 | 0.74521378 | 2747608 | 0.50612245 |
| 1472 | 2.0051053 | 3.36E-04 | 0.49872693 | 7 | 4 | Efna2 | 797 | 7 | 0.74872368 | 3167038 | 0.51321728 |
| 1473 | 2.00893427 | 3.24E-04 | 0.49777637 | 7 | 4 | E130309D14Rik | 791.2857143 | 7 | 0.74776643 | 3061352 | 0.51052049 |
| 1474 | 2.00255265 | 3.77E-04 | 0.49936265 | 7 | 4 | Dsc3 | 850 | 7 | 0.74936184 | 3641002 | 0.54668384 |
| 1475 | 1.99872368 | 3.88E-04 | 0.50031928 | 7 | 4 | Dlk1 | 864.4285714 | 7 | 0.75031908 | 3757504 | 0.55490268 |
| 1476 | 2.0051053 | 3.66E-04 | 0.49872693 | 7 | 4 | Diras2 | 844.2857143 | 7 | 0.74872368 | 3542442 | 0.54370452 |
| 1477 | 2.00127632 | 3.55E-04 | 0.49968112 | 7 | 4 | Dhcr24 | 836.4285714 | 7 | 0.74968092 | 3418160 | 0.53759882 |
| 1478 | 2.01914486 | 3.01E-04 | 0.49525917 | 7 | 4 | Ddn | 780.4285714 | 7 | 0.74521378 | 2747608 | 0.50612245 |
| 1479 | 2.01786854 | 3.12E-04 | 0.49557242 | 7 | 4 | Dclk1 | 786.1428571 | 7 | 0.74553287 | 2849432 | 0.50950218 |
| 1480 | 2.00255265 | 3.77E-04 | 0.49936265 | 7 | 4 | Dap3 | 850 | 7 | 0.74936184 | 3641002 | 0.54668384 |
| 1481 | 2.0051053 | 3.66E-04 | 0.49872693 | 7 | 4 | D430041D05Rik | 844.2857143 | 7 | 0.74872368 | 3542442 | 0.54370452 |
| 1482 | 2.00255265 | 3.77E-04 | 0.49936265 | 7 | 4 | Cyp2b19 | 850 | 7 | 0.74936184 | 3641002 | 0.54668384 |
| 1483 | 2.00255265 | 3.77E-04 | 0.49936265 | 7 | 4 | Cyp26b1 | 850 | 7 | 0.74936184 | 3641002 | 0.54668384 |
| 1484 | 1.99872368 | 3.88E-04 | 0.50031928 | 7 | 4 | Cybrd1 | 864.4285714 | 7 | 0.75031908 | 3757504 | 0.55490268 |
| 1485 | 2.00127632 | 3.67E-04 | 0.49968112 | 7 | 4 | Cxx1b | 842.1428571 | 7 | 0.74968092 | 3517930 | 0.54127597 |
| 1486 | 2.00127632 | 3.67E-04 | 0.49968112 | 7 | 4 | Cxx1a | 842.1428571 | 7 | 0.74968092 | 3517930 | 0.54127597 |
| 1487 | 2.0051053 | 3.66E-04 | 0.49872693 | 7 | 4 | Cxcl12 | 844.2857143 | 7 | 0.74872368 | 3542442 | 0.54370452 |
| 1488 | 2.03318443 | 2.79E-04 | 0.4918393 | 7 | 4 | Crispld2 | 758.1428571 | 7 | 0.74170389 | 2504400 | 0.49518826 |
| 1489 | 2.00127632 | 3.55E-04 | 0.49968112 | 7 | 4 | Crat | 836.4285714 | 7 | 0.74968092 | 3418160 | 0.53759882 |
| 1490 | 1.99872368 | 3.88E-04 | 0.50031928 | 7 | 4 | Cpd | 864.4285714 | 7 | 0.75031908 | 3757504 | 0.55490268 |
| 1491 | 2.0051053 | 3.36E-04 | 0.49872693 | 7 | 4 | Cops7a | 797 | 7 | 0.74872368 | 3167038 | 0.51321728 |
| 1492 | 2.00127632 | 3.67E-04 | 0.49968112 | 7 | 4 | Col27a1 | 842.1428571 | 7 | 0.74968092 | 3517930 | 0.54127597 |
| 1493 | 2.00127632 | 3.67E-04 | 0.49968112 | 7 | 4 | Cog3 | 842.1428571 | 7 | 0.74968092 | 3517930 | 0.54127597 |
| 1494 | 2.01914486 | 3.01E-04 | 0.49525917 | 7 | 4 | Clec16a | 780.4285714 | 7 | 0.74521378 | 2747608 | 0.50612245 |
| 1495 | 2.0051053 | 3.66E-04 | 0.49872693 | 7 | 4 | Chst15 | 844.2857143 | 7 | 0.74872368 | 3542442 | 0.54370452 |
| 1496 | 2.03318443 | 2.79E-04 | 0.4918393 | 7 | 4 | Chst11 | 758.1428571 | 7 | 0.74170389 | 2504400 | 0.49518826 |
| 1497 | 2.00255265 | 3.77E-04 | 0.49936265 | 7 | 4 | Chrnd | 850 | 7 | 0.74936184 | 3641002 | 0.54668384 |
| 1498 | 2.0051053 | 3.36E-04 | 0.49872693 | 7 | 4 | Chd5 | 797 | 7 | 0.74872368 | 3167038 | 0.51321728 |
| 1499 | 1.99872368 | 3.88E-04 | 0.50031928 | 7 | 4 | Cerk | 864.4285714 | 7 | 0.75031908 | 3757504 | 0.55490268 |
| 1500 | 2.00127632 | 3.67E-04 | 0.49968112 | 7 | 4 | Cdk5r1 | 842.1428571 | 7 | 0.74968092 | 3517930 | 0.54127597 |
| 1501 | 1.99872368 | 3.88E-04 | 0.50031928 | 7 | 4 | Cd99l2 | 864.4285714 | 7 | 0.75031908 | 3757504 | 0.55490268 |
| 1502 | 2.0051053 | 3.66E-04 | 0.49872693 | 7 | 4 | Cacng5 | 844.2857143 | 7 | 0.74872368 | 3542442 | 0.54370452 |
| 1503 | 2.00127632 | 3.55E-04 | 0.49968112 | 7 | 4 | C1qtnf6 | 836.4285714 | 7 | 0.74968092 | 3418160 | 0.53759882 |
| 1504 | 2.02935546 | 2.90E-04 | 0.4927673 | 7 | 4 | C130074G19Rik | 766 | 7 | 0.74266114 | 2632558 | 0.49934726 |
| 1505 | 2.00638162 | 3.44E-04 | 0.49840967 | 7 | 4 | Bsn | 822 | 7 | 0.74840459 | 3296052 | 0.52967742 |
| 1506 | 2.00127632 | 3.67E-04 | 0.49968112 | 7 | 4 | Bsdc1 | 842.1428571 | 7 | 0.74968092 | 3517930 | 0.54127597 |
| 1507 | 2.0051053 | 3.66E-04 | 0.49872693 | 7 | 4 | Bend4 | 844.2857143 | 7 | 0.74872368 | 3542442 | 0.54370452 |
| 1508 | 1.99872368 | 3.88E-04 | 0.50031928 | 7 | 4 | Bcl2l11 | 864.4285714 | 7 | 0.75031908 | 3757504 | 0.55490268 |
| 1509 | 2.0051053 | 3.36E-04 | 0.49872693 | 7 | 4 | Bcl11b | 797 | 7 | 0.74872368 | 3167038 | 0.51321728 |
| 1510 | 2.00255265 | 3.77E-04 | 0.49936265 | 7 | 4 | Bai1 | 850 | 7 | 0.74936184 | 3641002 | 0.54668384 |
| 1511 | 1.99872368 | 3.88E-04 | 0.50031928 | 7 | 4 | Bag2 | 864.4285714 | 7 | 0.75031908 | 3757504 | 0.55490268 |
| 1512 | 2.00893427 | 3.24E-04 | 0.49777637 | 7 | 4 | Bach2 | 791.2857143 | 7 | 0.74776643 | 3061352 | 0.51052049 |
| 1513 | 2.0051053 | 3.66E-04 | 0.49872693 | 7 | 4 | B4galt5 | 844.2857143 | 7 | 0.74872368 | 3542442 | 0.54370452 |
| 1514 | 2.0051053 | 3.66E-04 | 0.49872693 | 7 | 4 | AW549877 | 844.2857143 | 7 | 0.74872368 | 3542442 | 0.54370452 |
| 1515 | 2.00127632 | 3.67E-04 | 0.49968112 | 7 | 4 | Atp1a2 | 842.1428571 | 7 | 0.74968092 | 3517930 | 0.54127597 |
| 1516 | 2.00893427 | 3.24E-04 | 0.49777637 | 7 | 4 | Atg13 | 791.2857143 | 7 | 0.74776643 | 3061352 | 0.51052049 |
| 1517 | 1.99872368 | 3.88E-04 | 0.50031928 | 7 | 4 | Atg12 | 864.4285714 | 7 | 0.75031908 | 3757504 | 0.55490268 |
| 1518 | 2.01914486 | 3.01E-04 | 0.49525917 | 7 | 4 | Atf6 | 780.4285714 | 7 | 0.74521378 | 2747608 | 0.50612245 |
| 1519 | 2.00127632 | 3.67E-04 | 0.49968112 | 7 | 4 | Asb8 | 842.1428571 | 7 | 0.74968092 | 3517930 | 0.54127597 |
| 1520 | 2.0051053 | 3.57E-04 | 0.49872693 | 7 | 4 | Armc2 | 819.2857143 | 7 | 0.74872368 | 3411330 | 0.52758589 |
| 1521 | 1.99872368 | 3.88E-04 | 0.50031928 | 7 | 4 | Arid1b | 864.4285714 | 7 | 0.75031908 | 3757504 | 0.55490268 |
| 1522 | 2.10848756 | 2.47E-04 | 0.47427361 | 7 | 4 | Arhgef17 | 713 | 7 | 0.72287811 | 2151026 | 0.48435374 |
| 1523 | 1.99872368 | 3.88E-04 | 0.50031928 | 7 | 4 | Arf2 | 864.4285714 | 7 | 0.75031908 | 3757504 | 0.55490268 |
| 1524 | 2.00255265 | 3.77E-04 | 0.49936265 | 7 | 4 | Arap1 | 850 | 7 | 0.74936184 | 3641002 | 0.54668384 |
| 1525 | 1.99872368 | 3.88E-04 | 0.50031928 | 7 | 4 | Aph1b | 864.4285714 | 7 | 0.75031908 | 3757504 | 0.55490268 |
| 1526 | 2.01786854 | 3.12E-04 | 0.49557242 | 7 | 4 | Aph1a | 786.1428571 | 7 | 0.74553287 | 2849432 | 0.50950218 |
| 1527 | 2.05615826 | 2.69E-04 | 0.48634389 | 7 | 4 | Anks1b | 733.1428571 | 7 | 0.73596043 | 2373232 | 0.48454193 |
| 1528 | 2.00638162 | 3.44E-04 | 0.49840967 | 7 | 4 | Ankrd6 | 822 | 7 | 0.74840459 | 3296052 | 0.52967742 |
| 1529 | 2.00127632 | 3.67E-04 | 0.49968112 | 7 | 4 | Ankfy1 | 842.1428571 | 7 | 0.74968092 | 3517930 | 0.54127597 |
| 1530 | 1.99872368 | 3.88E-04 | 0.50031928 | 7 | 4 | Ampd2 | 864.4285714 | 7 | 0.75031908 | 3757504 | 0.55490268 |
| 1531 | 2.00127632 | 3.67E-04 | 0.49968112 | 7 | 4 | Amer1 | 842.1428571 | 7 | 0.74968092 | 3517930 | 0.54127597 |
| 1532 | 2.0051053 | 3.36E-04 | 0.49872693 | 7 | 4 | Ak1 | 797 | 7 | 0.74872368 | 3167038 | 0.51321728 |
| 1533 | 2.00127632 | 3.46E-04 | 0.49968112 | 7 | 4 | Aif1l | 811.4285714 | 7 | 0.74968092 | 3281746 | 0.52151131 |
| 1534 | 1.99872368 | 3.88E-04 | 0.50031928 | 7 | 4 | Agpat5 | 864.4285714 | 7 | 0.75031908 | 3757504 | 0.55490268 |
| 1535 | 2.0051053 | 3.66E-04 | 0.49872693 | 7 | 4 | Adarb1 | 844.2857143 | 7 | 0.74872368 | 3542442 | 0.54370452 |
| 1536 | 2.00893427 | 3.24E-04 | 0.49777637 | 7 | 4 | Adam19 | 791.2857143 | 7 | 0.74776643 | 3061352 | 0.51052049 |
| 1537 | 2.03318443 | 2.79E-04 | 0.4918393 | 7 | 4 | Abl1 | 758.1428571 | 7 | 0.74170389 | 2504400 | 0.49518826 |
| 1538 | 2.00255265 | 3.77E-04 | 0.49936265 | 7 | 4 | A330050F15Rik | 850 | 7 | 0.74936184 | 3641002 | 0.54668384 |
| 1539 | 2.0051053 | 3.36E-04 | 0.49872693 | 7 | 4 | 9130019O22Rik | 797 | 7 | 0.74872368 | 3167038 | 0.51321728 |
| 1540 | 2.04084237 | 2.81E-04 | 0.48999375 | 7 | 4 | 5031439G07Rik | 741 | 7 | 0.73978941 | 2506692 | 0.48588313 |
| 1208 | 2.0319081 | 2.64E-04 | 0.49214824 | 6 | 4 | Mapk1 | 895.3333333 | 6 | 0.74202297 | 2565532 | 0.58414979 |
| 1209 | 2.0319081 | 2.53E-04 | 0.49214824 | 6 | 4 | Map3k7 | 888.6666667 | 6 | 0.74202297 | 2464200 | 0.57979534 |
| 1210 | 2.06253989 | 2.44E-04 | 0.48483911 | 6 | 4 | Map3k3 | 859.5 | 6 | 0.73436503 | 2333896 | 0.56967485 |
| 1211 | 2.06253989 | 2.44E-04 | 0.48483911 | 6 | 4 | Mal | 859.5 | 6 | 0.73436503 | 2333896 | 0.56967485 |
| 1212 | 2.0319081 | 2.64E-04 | 0.49214824 | 6 | 4 | Lrrc3 | 895.3333333 | 6 | 0.74202297 | 2565532 | 0.58414979 |
| 1213 | 2.0319081 | 2.64E-04 | 0.49214824 | 6 | 4 | Lrp6 | 895.3333333 | 6 | 0.74202297 | 2565532 | 0.58414979 |
| 1214 | 2.06509253 | 2.34E-04 | 0.4842398 | 6 | 4 | Lrba | 842.6666667 | 6 | 0.73372687 | 2226548 | 0.55924695 |
| 1215 | 2.0319081 | 2.53E-04 | 0.49214824 | 6 | 4 | Lphn1 | 888.6666667 | 6 | 0.74202297 | 2464200 | 0.57979534 |
| 1216 | 2.0319081 | 2.64E-04 | 0.49214824 | 6 | 4 | LOC101056447 | 895.3333333 | 6 | 0.74202297 | 2565532 | 0.58414979 |
| 1217 | 2.0319081 | 2.64E-04 | 0.49214824 | 6 | 4 | LOC101055946 | 895.3333333 | 6 | 0.74202297 | 2565532 | 0.58414979 |
| 1218 | 2.0319081 | 2.64E-04 | 0.49214824 | 6 | 4 | LOC101055798 | 895.3333333 | 6 | 0.74202297 | 2565532 | 0.58414979 |
| 1219 | 2.13784301 | 2.01E-04 | 0.46776119 | 6 | 4 | LOC101055678 | 810 | 6 | 0.71553925 | 1873726 | 0.55870166 |
| 1220 | 2.05105297 | 2.43E-04 | 0.48755445 | 6 | 4 | Lenep | 871.8333333 | 6 | 0.73723676 | 2349452 | 0.57442832 |
| 1221 | 2.06253989 | 2.44E-04 | 0.48483911 | 6 | 4 | Lef1 | 859.5 | 6 | 0.73436503 | 2333896 | 0.56967485 |
| 1222 | 2.04850032 | 2.31E-04 | 0.48816199 | 6 | 4 | Ldb3 | 862.6666667 | 6 | 0.73787492 | 2223344 | 0.56763285 |
| 1223 | 2.0319081 | 2.64E-04 | 0.49214824 | 6 | 4 | Lbh | 895.3333333 | 6 | 0.74202297 | 2565532 | 0.58414979 |
| 1224 | 2.07657945 | 2.23E-04 | 0.48156116 | 6 | 4 | Larp1 | 833.5 | 6 | 0.73085514 | 2095138 | 0.55648396 |
| 1225 | 2.05105297 | 2.43E-04 | 0.48755445 | 6 | 4 | L3mbtl2 | 871.8333333 | 6 | 0.73723676 | 2349452 | 0.57442832 |
| 1226 | 2.06509253 | 2.34E-04 | 0.4842398 | 6 | 4 | Krt75 | 842.6666667 | 6 | 0.73372687 | 2226548 | 0.55924695 |
| 1227 | 2.04850032 | 2.31E-04 | 0.48816199 | 6 | 4 | Kremen1 | 862.6666667 | 6 | 0.73787492 | 2223344 | 0.56763285 |
| 1228 | 2.0319081 | 2.64E-04 | 0.49214824 | 6 | 4 | Knop1 | 895.3333333 | 6 | 0.74202297 | 2565532 | 0.58414979 |
| 1229 | 2.07657945 | 2.23E-04 | 0.48156116 | 6 | 4 | Klf16 | 833.5 | 6 | 0.73085514 | 2095138 | 0.55648396 |
| 1230 | 2.11997447 | 1.79E-04 | 0.47170379 | 6 | 4 | Kctd10 | 797.3333333 | 6 | 0.72000638 | 1589000 | 0.54468764 |
| 1231 | 2.05105297 | 2.43E-04 | 0.48755445 | 6 | 4 | Kcnk5 | 871.8333333 | 6 | 0.73723676 | 2349452 | 0.57442832 |
| 1232 | 2.0319081 | 2.64E-04 | 0.49214824 | 6 | 4 | Kbtbd13 | 895.3333333 | 6 | 0.74202297 | 2565532 | 0.58414979 |
| 1233 | 2.05105297 | 2.43E-04 | 0.48755445 | 6 | 4 | Jrk | 871.8333333 | 6 | 0.73723676 | 2349452 | 0.57442832 |
| 1234 | 2.0319081 | 2.53E-04 | 0.49214824 | 6 | 4 | Jam2 | 888.6666667 | 6 | 0.74202297 | 2464200 | 0.57979534 |
| 1235 | 2.10465858 | 2.11E-04 | 0.47513645 | 6 | 4 | Itga3 | 826.8333333 | 6 | 0.72383535 | 1986680 | 0.56026685 |
| 1236 | 2.0319081 | 2.53E-04 | 0.49214824 | 6 | 4 | Irs1 | 888.6666667 | 6 | 0.74202297 | 2464200 | 0.57979534 |
| 1237 | 2.0319081 | 2.64E-04 | 0.49214824 | 6 | 4 | Irgm2 | 895.3333333 | 6 | 0.74202297 | 2565532 | 0.58414979 |
| 1238 | 2.0319081 | 2.53E-04 | 0.49214824 | 6 | 4 | Iqsec1 | 888.6666667 | 6 | 0.74202297 | 2464200 | 0.57979534 |
| 1239 | 2.04850032 | 2.31E-04 | 0.48816199 | 6 | 4 | Iqgap1 | 862.6666667 | 6 | 0.73787492 | 2223344 | 0.56763285 |
| 1240 | 2.0319081 | 2.53E-04 | 0.49214824 | 6 | 4 | Ints2 | 888.6666667 | 6 | 0.74202297 | 2464200 | 0.57979534 |
| 1241 | 2.10465858 | 2.11E-04 | 0.47513645 | 6 | 4 | Il20ra | 826.8333333 | 6 | 0.72383535 | 1986680 | 0.56026685 |
| 1242 | 2.07657945 | 2.23E-04 | 0.48156116 | 6 | 4 | Ikbke | 833.5 | 6 | 0.73085514 | 2095138 | 0.55648396 |
| 1243 | 2.0319081 | 2.64E-04 | 0.49214824 | 6 | 4 | Igsf3 | 895.3333333 | 6 | 0.74202297 | 2565532 | 0.58414979 |
| 1244 | 2.06253989 | 2.44E-04 | 0.48483911 | 6 | 4 | Igfbpl1 | 859.5 | 6 | 0.73436503 | 2333896 | 0.56967485 |
| 1245 | 2.0319081 | 2.53E-04 | 0.49214824 | 6 | 4 | Ift46 | 888.6666667 | 6 | 0.74202297 | 2464200 | 0.57979534 |
| 1246 | 2.0319081 | 2.53E-04 | 0.49214824 | 6 | 4 | Icos | 888.6666667 | 6 | 0.74202297 | 2464200 | 0.57979534 |
| 1247 | 2.27696235 | 1.48E-04 | 0.43918161 | 6 | 4 | Ick | 744.6666667 | 6 | 0.68075941 | 1247534 | 0.55538959 |
| 1248 | 2.07657945 | 2.23E-04 | 0.48156116 | 6 | 4 | Htr1a | 833.5 | 6 | 0.73085514 | 2095138 | 0.55648396 |
| 1249 | 2.0319081 | 2.64E-04 | 0.49214824 | 6 | 4 | Hs2st1 | 895.3333333 | 6 | 0.74202297 | 2565532 | 0.58414979 |
| 1250 | 2.0319081 | 2.53E-04 | 0.49214824 | 6 | 4 | Hoxb5 | 888.6666667 | 6 | 0.74202297 | 2464200 | 0.57979534 |
| 1251 | 2.08296107 | 2.22E-04 | 0.48008578 | 6 | 4 | Hoxb1 | 836 | 6 | 0.72925973 | 2119300 | 0.56002683 |
| 1252 | 2.06253989 | 2.44E-04 | 0.48483911 | 6 | 4 | Hmgxb3 | 859.5 | 6 | 0.73436503 | 2333896 | 0.56967485 |
| 1253 | 2.04850032 | 2.31E-04 | 0.48816199 | 6 | 4 | Hmgcs2 | 862.6666667 | 6 | 0.73787492 | 2223344 | 0.56763285 |
| 1254 | 2.04850032 | 2.31E-04 | 0.48816199 | 6 | 4 | Hivep3 | 862.6666667 | 6 | 0.73787492 | 2223344 | 0.56763285 |
| 1255 | 2.05105297 | 2.43E-04 | 0.48755445 | 6 | 4 | Hhip | 871.8333333 | 6 | 0.73723676 | 2349452 | 0.57442832 |
| 1256 | 2.29738354 | 6.55E-05 | 0.43527778 | 6 | 4 | Hdlbp | 587.3333333 | 6 | 0.67565412 | 565870 | 0.44318468 |
| 1257 | 2.13784301 | 2.01E-04 | 0.46776119 | 6 | 4 | Hdac7 | 810 | 6 | 0.71553925 | 1873726 | 0.55870166 |
| 1258 | 2.05105297 | 2.43E-04 | 0.48755445 | 6 | 4 | Hcrtr1 | 871.8333333 | 6 | 0.73723676 | 2349452 | 0.57442832 |
| 1259 | 2.06509253 | 2.34E-04 | 0.4842398 | 6 | 4 | Gucd1 | 842.6666667 | 6 | 0.73372687 | 2226548 | 0.55924695 |
| 1260 | 2.06253989 | 2.44E-04 | 0.48483911 | 6 | 4 | Gtf3c5 | 859.5 | 6 | 0.73436503 | 2333896 | 0.56967485 |
| 1261 | 2.06253989 | 2.44E-04 | 0.48483911 | 6 | 4 | Grin2b | 859.5 | 6 | 0.73436503 | 2333896 | 0.56967485 |
| 1262 | 2.04850032 | 2.31E-04 | 0.48816199 | 6 | 4 | Greb1l | 862.6666667 | 6 | 0.73787492 | 2223344 | 0.56763285 |
| 1263 | 2.08296107 | 2.22E-04 | 0.48008578 | 6 | 4 | Gpr152 | 836 | 6 | 0.72925973 | 2119300 | 0.56002683 |
| 1264 | 2.0319081 | 2.53E-04 | 0.49214824 | 6 | 4 | Gpr116 | 888.6666667 | 6 | 0.74202297 | 2464200 | 0.57979534 |
| 1265 | 2.0319081 | 2.53E-04 | 0.49214824 | 6 | 4 | Gpd2 | 888.6666667 | 6 | 0.74202297 | 2464200 | 0.57979534 |
| 1266 | 2.05105297 | 2.43E-04 | 0.48755445 | 6 | 4 | Golgb1 | 871.8333333 | 6 | 0.73723676 | 2349452 | 0.57442832 |
| 1267 | 2.08296107 | 2.22E-04 | 0.48008578 | 6 | 4 | Gng7 | 836 | 6 | 0.72925973 | 2119300 | 0.56002683 |
| 1268 | 2.0319081 | 2.53E-04 | 0.49214824 | 6 | 4 | Gne | 888.6666667 | 6 | 0.74202297 | 2464200 | 0.57979534 |
| 1269 | 2.13784301 | 2.01E-04 | 0.46776119 | 6 | 4 | Gm13083 | 810 | 6 | 0.71553925 | 1873726 | 0.55870166 |
| 1270 | 2.0319081 | 2.53E-04 | 0.49214824 | 6 | 4 | Gjb2 | 888.6666667 | 6 | 0.74202297 | 2464200 | 0.57979534 |
| 1271 | 2.0319081 | 2.53E-04 | 0.49214824 | 6 | 4 | Gimap8 | 888.6666667 | 6 | 0.74202297 | 2464200 | 0.57979534 |
| 1272 | 2.07657945 | 2.23E-04 | 0.48156116 | 6 | 4 | Ggta1 | 833.5 | 6 | 0.73085514 | 2095138 | 0.55648396 |
| 1273 | 2.05105297 | 2.43E-04 | 0.48755445 | 6 | 4 | Gga1 | 871.8333333 | 6 | 0.73723676 | 2349452 | 0.57442832 |
| 1274 | 2.10465858 | 2.11E-04 | 0.47513645 | 6 | 4 | Gfap | 826.8333333 | 6 | 0.72383535 | 1986680 | 0.56026685 |
| 1275 | 2.08296107 | 2.22E-04 | 0.48008578 | 6 | 4 | Gatsl2 | 836 | 6 | 0.72925973 | 2119300 | 0.56002683 |
| 1276 | 2.04850032 | 2.31E-04 | 0.48816199 | 6 | 4 | Gas2l1 | 862.6666667 | 6 | 0.73787492 | 2223344 | 0.56763285 |
| 1277 | 2.06253989 | 2.44E-04 | 0.48483911 | 6 | 4 | Galnt9 | 859.5 | 6 | 0.73436503 | 2333896 | 0.56967485 |
| 1278 | 2.0319081 | 2.64E-04 | 0.49214824 | 6 | 4 | Fras1 | 895.3333333 | 6 | 0.74202297 | 2565532 | 0.58414979 |
| 1279 | 2.0319081 | 2.53E-04 | 0.49214824 | 6 | 4 | Foxm1 | 888.6666667 | 6 | 0.74202297 | 2464200 | 0.57979534 |
| 1280 | 2.05105297 | 2.43E-04 | 0.48755445 | 6 | 4 | Foxk1 | 871.8333333 | 6 | 0.73723676 | 2349452 | 0.57442832 |
| 1281 | 2.18506701 | 1.70E-04 | 0.45765187 | 6 | 4 | Fnip2 | 768.1666667 | 6 | 0.70373325 | 1463942 | 0.54370423 |
| 1282 | 2.06509253 | 2.34E-04 | 0.4842398 | 6 | 4 | Fmn1 | 842.6666667 | 6 | 0.73372687 | 2226548 | 0.55924695 |
| 1283 | 2.13784301 | 2.01E-04 | 0.46776119 | 6 | 4 | Flt1 | 810 | 6 | 0.71553925 | 1873726 | 0.55870166 |
| 1284 | 2.0319081 | 2.64E-04 | 0.49214824 | 6 | 4 | Fgfr1op | 895.3333333 | 6 | 0.74202297 | 2565532 | 0.58414979 |
| 1285 | 2.04850032 | 2.31E-04 | 0.48816199 | 6 | 4 | Fgfr1 | 862.6666667 | 6 | 0.73787492 | 2223344 | 0.56763285 |
| 1286 | 2.35098915 | 1.35E-04 | 0.42535288 | 6 | 4 | Fgf16 | 735.5 | 6 | 0.66225271 | 1111038 | 0.57338017 |
| 1287 | 2.08296107 | 2.22E-04 | 0.48008578 | 6 | 4 | Fgd6 | 836 | 6 | 0.72925973 | 2119300 | 0.56002683 |
| 1288 | 2.22846203 | 1.57E-04 | 0.44873998 | 6 | 4 | Fgd3 | 761.5 | 6 | 0.69288449 | 1353430 | 0.55228758 |
| 1289 | 2.0319081 | 2.64E-04 | 0.49214824 | 6 | 4 | Fem1b | 895.3333333 | 6 | 0.74202297 | 2565532 | 0.58414979 |
| 1290 | 2.11997447 | 1.79E-04 | 0.47170379 | 6 | 4 | Fbxo31 | 797.3333333 | 6 | 0.72000638 | 1589000 | 0.54468764 |
| 1291 | 2.06253989 | 2.44E-04 | 0.48483911 | 6 | 4 | Fblim1 | 859.5 | 6 | 0.73436503 | 2333896 | 0.56967485 |
| 1292 | 2.0319081 | 2.64E-04 | 0.49214824 | 6 | 4 | Faxc | 895.3333333 | 6 | 0.74202297 | 2565532 | 0.58414979 |
| 1293 | 2.18506701 | 1.70E-04 | 0.45765187 | 6 | 4 | Fam49a | 768.1666667 | 6 | 0.70373325 | 1463942 | 0.54370423 |
| 1294 | 2.0319081 | 2.53E-04 | 0.49214824 | 6 | 4 | Fam26e | 888.6666667 | 6 | 0.74202297 | 2464200 | 0.57979534 |
| 1295 | 2.08296107 | 2.22E-04 | 0.48008578 | 6 | 4 | Fam227a | 836 | 6 | 0.72925973 | 2119300 | 0.56002683 |
| 1296 | 2.0319081 | 2.53E-04 | 0.49214824 | 6 | 4 | Fam212b | 888.6666667 | 6 | 0.74202297 | 2464200 | 0.57979534 |
| 1297 | 2.05105297 | 2.43E-04 | 0.48755445 | 6 | 4 | Fam207a | 871.8333333 | 6 | 0.73723676 | 2349452 | 0.57442832 |
| 1298 | 2.22846203 | 1.57E-04 | 0.44873998 | 6 | 4 | Fam118a | 761.5 | 6 | 0.69288449 | 1353430 | 0.55228758 |
| 1299 | 2.0319081 | 2.64E-04 | 0.49214824 | 6 | 4 | Esrrb | 895.3333333 | 6 | 0.74202297 | 2565532 | 0.58414979 |
| 1300 | 2.05105297 | 2.43E-04 | 0.48755445 | 6 | 4 | Ercc4 | 871.8333333 | 6 | 0.73723676 | 2349452 | 0.57442832 |
| 1301 | 2.0319081 | 2.64E-04 | 0.49214824 | 6 | 4 | Eps15l1 | 895.3333333 | 6 | 0.74202297 | 2565532 | 0.58414979 |
| 1302 | 2.07657945 | 2.23E-04 | 0.48156116 | 6 | 4 | Epm2aip1 | 833.5 | 6 | 0.73085514 | 2095138 | 0.55648396 |
| 1303 | 2.10465858 | 2.11E-04 | 0.47513645 | 6 | 4 | Epha8 | 826.8333333 | 6 | 0.72383535 | 1986680 | 0.56026685 |
| 1304 | 2.07657945 | 2.23E-04 | 0.48156116 | 6 | 4 | Epha10 | 833.5 | 6 | 0.73085514 | 2095138 | 0.55648396 |
| 1305 | 2.0319081 | 2.64E-04 | 0.49214824 | 6 | 4 | Epb4.1l5 | 895.3333333 | 6 | 0.74202297 | 2565532 | 0.58414979 |
| 1306 | 2.10465858 | 2.11E-04 | 0.47513645 | 6 | 4 | Epb4.1 | 826.8333333 | 6 | 0.72383535 | 1986680 | 0.56026685 |
| 1307 | 2.0319081 | 2.64E-04 | 0.49214824 | 6 | 4 | Elf5 | 895.3333333 | 6 | 0.74202297 | 2565532 | 0.58414979 |
| 1308 | 2.08296107 | 2.22E-04 | 0.48008578 | 6 | 4 | Elf4 | 836 | 6 | 0.72925973 | 2119300 | 0.56002683 |
| 1309 | 2.06253989 | 2.44E-04 | 0.48483911 | 6 | 4 | Elac1 | 859.5 | 6 | 0.73436503 | 2333896 | 0.56967485 |
| 1310 | 2.0319081 | 2.53E-04 | 0.49214824 | 6 | 4 | Ehd4 | 888.6666667 | 6 | 0.74202297 | 2464200 | 0.57979534 |
| 1311 | 2.04850032 | 2.31E-04 | 0.48816199 | 6 | 4 | Efhd2 | 862.6666667 | 6 | 0.73787492 | 2223344 | 0.56763285 |
| 1312 | 2.0319081 | 2.64E-04 | 0.49214824 | 6 | 4 | Echdc3 | 895.3333333 | 6 | 0.74202297 | 2565532 | 0.58414979 |
| 1313 | 2.06253989 | 2.44E-04 | 0.48483911 | 6 | 4 | Ece1 | 859.5 | 6 | 0.73436503 | 2333896 | 0.56967485 |
| 1314 | 2.04850032 | 2.31E-04 | 0.48816199 | 6 | 4 | Eaf1 | 862.6666667 | 6 | 0.73787492 | 2223344 | 0.56763285 |
| 1315 | 2.05105297 | 2.43E-04 | 0.48755445 | 6 | 4 | Dynlt1a | 871.8333333 | 6 | 0.73723676 | 2349452 | 0.57442832 |
| 1316 | 2.06253989 | 2.44E-04 | 0.48483911 | 6 | 4 | Dscr3 | 859.5 | 6 | 0.73436503 | 2333896 | 0.56967485 |
| 1317 | 2.07657945 | 2.23E-04 | 0.48156116 | 6 | 4 | Dock5 | 833.5 | 6 | 0.73085514 | 2095138 | 0.55648396 |
| 1318 | 2.07657945 | 2.23E-04 | 0.48156116 | 6 | 4 | Dnajc5g | 833.5 | 6 | 0.73085514 | 2095138 | 0.55648396 |
| 1319 | 2.07657945 | 2.23E-04 | 0.48156116 | 6 | 4 | Dip2b | 833.5 | 6 | 0.73085514 | 2095138 | 0.55648396 |
| 1320 | 2.05105297 | 2.43E-04 | 0.48755445 | 6 | 4 | Diexf | 871.8333333 | 6 | 0.73723676 | 2349452 | 0.57442832 |
| 1321 | 2.05105297 | 2.43E-04 | 0.48755445 | 6 | 4 | Diap1 | 871.8333333 | 6 | 0.73723676 | 2349452 | 0.57442832 |
| 1322 | 2.0319081 | 2.53E-04 | 0.49214824 | 6 | 4 | Dhx35 | 888.6666667 | 6 | 0.74202297 | 2464200 | 0.57979534 |
| 1323 | 2.0319081 | 2.53E-04 | 0.49214824 | 6 | 4 | Dgkd | 888.6666667 | 6 | 0.74202297 | 2464200 | 0.57979534 |
| 1324 | 2.0319081 | 2.64E-04 | 0.49214824 | 6 | 4 | Dennd6b | 895.3333333 | 6 | 0.74202297 | 2565532 | 0.58414979 |
| 1325 | 2.0319081 | 2.64E-04 | 0.49214824 | 6 | 4 | Dennd4a | 895.3333333 | 6 | 0.74202297 | 2565532 | 0.58414979 |
| 1326 | 2.06253989 | 2.44E-04 | 0.48483911 | 6 | 4 | Ddx6 | 859.5 | 6 | 0.73436503 | 2333896 | 0.56967485 |
| 1327 | 2.05105297 | 2.43E-04 | 0.48755445 | 6 | 4 | Dcp2 | 871.8333333 | 6 | 0.73723676 | 2349452 | 0.57442832 |
| 1328 | 2.0319081 | 2.64E-04 | 0.49214824 | 6 | 4 | Dcp1a | 895.3333333 | 6 | 0.74202297 | 2565532 | 0.58414979 |
| 1329 | 2.0319081 | 2.64E-04 | 0.49214824 | 6 | 4 | Dclk2 | 895.3333333 | 6 | 0.74202297 | 2565532 | 0.58414979 |
| 1330 | 2.05105297 | 2.43E-04 | 0.48755445 | 6 | 4 | Dbnl | 871.8333333 | 6 | 0.73723676 | 2349452 | 0.57442832 |
| 1331 | 2.0319081 | 2.53E-04 | 0.49214824 | 6 | 4 | D130043K22Rik | 888.6666667 | 6 | 0.74202297 | 2464200 | 0.57979534 |
| 1332 | 2.05105297 | 2.43E-04 | 0.48755445 | 6 | 4 | Cyp2ab1 | 871.8333333 | 6 | 0.73723676 | 2349452 | 0.57442832 |
| 1333 | 2.0319081 | 2.64E-04 | 0.49214824 | 6 | 4 | Cyb561d1 | 895.3333333 | 6 | 0.74202297 | 2565532 | 0.58414979 |
| 1334 | 2.0319081 | 2.53E-04 | 0.49214824 | 6 | 4 | Cxcr5 | 888.6666667 | 6 | 0.74202297 | 2464200 | 0.57979534 |
| 1335 | 2.10465858 | 2.11E-04 | 0.47513645 | 6 | 4 | Csf1 | 826.8333333 | 6 | 0.72383535 | 1986680 | 0.56026685 |
| 1336 | 2.0319081 | 2.53E-04 | 0.49214824 | 6 | 4 | Crybg3 | 888.6666667 | 6 | 0.74202297 | 2464200 | 0.57979534 |
| 1337 | 2.0319081 | 2.53E-04 | 0.49214824 | 6 | 4 | Cry2 | 888.6666667 | 6 | 0.74202297 | 2464200 | 0.57979534 |
| 1338 | 2.29738354 | 6.55E-05 | 0.43527778 | 6 | 4 | Crtc1 | 587.3333333 | 6 | 0.67565412 | 565870 | 0.44318468 |
| 1339 | 2.05105297 | 2.43E-04 | 0.48755445 | 6 | 4 | Creb5 | 871.8333333 | 6 | 0.73723676 | 2349452 | 0.57442832 |
| 1340 | 2.13784301 | 2.01E-04 | 0.46776119 | 6 | 4 | Cpne5 | 810 | 6 | 0.71553925 | 1873726 | 0.55870166 |
| 1341 | 2.11997447 | 1.79E-04 | 0.47170379 | 6 | 4 | Cops2 | 797.3333333 | 6 | 0.72000638 | 1589000 | 0.54468764 |
| 1342 | 2.0319081 | 2.64E-04 | 0.49214824 | 6 | 4 | Col24a1 | 895.3333333 | 6 | 0.74202297 | 2565532 | 0.58414979 |
| 1343 | 2.0319081 | 2.53E-04 | 0.49214824 | 6 | 4 | Col19a1 | 888.6666667 | 6 | 0.74202297 | 2464200 | 0.57979534 |
| 1344 | 2.04850032 | 2.31E-04 | 0.48816199 | 6 | 4 | Clcn3 | 862.6666667 | 6 | 0.73787492 | 2223344 | 0.56763285 |
| 1345 | 2.0319081 | 2.64E-04 | 0.49214824 | 6 | 4 | Ciapin1 | 895.3333333 | 6 | 0.74202297 | 2565532 | 0.58414979 |
| 1346 | 2.05105297 | 2.43E-04 | 0.48755445 | 6 | 4 | Chst10 | 871.8333333 | 6 | 0.73723676 | 2349452 | 0.57442832 |
| 1347 | 2.10465858 | 2.11E-04 | 0.47513645 | 6 | 4 | Chrnb3 | 826.8333333 | 6 | 0.72383535 | 1986680 | 0.56026685 |
| 1348 | 2.06509253 | 2.34E-04 | 0.4842398 | 6 | 4 | Chrm4 | 842.6666667 | 6 | 0.73372687 | 2226548 | 0.55924695 |
| 1349 | 2.0319081 | 2.53E-04 | 0.49214824 | 6 | 4 | Chrm1 | 888.6666667 | 6 | 0.74202297 | 2464200 | 0.57979534 |
| 1350 | 2.04850032 | 2.31E-04 | 0.48816199 | 6 | 4 | Chmp3 | 862.6666667 | 6 | 0.73787492 | 2223344 | 0.56763285 |
| 1351 | 2.0319081 | 2.53E-04 | 0.49214824 | 6 | 4 | Cers3 | 888.6666667 | 6 | 0.74202297 | 2464200 | 0.57979534 |
| 1352 | 2.05105297 | 2.43E-04 | 0.48755445 | 6 | 4 | Celsr1 | 871.8333333 | 6 | 0.73723676 | 2349452 | 0.57442832 |
| 1353 | 2.0319081 | 2.64E-04 | 0.49214824 | 6 | 4 | Cds1 | 895.3333333 | 6 | 0.74202297 | 2565532 | 0.58414979 |
| 1354 | 2.0319081 | 2.53E-04 | 0.49214824 | 6 | 4 | Cdk19 | 888.6666667 | 6 | 0.74202297 | 2464200 | 0.57979534 |
| 1355 | 2.13784301 | 2.01E-04 | 0.46776119 | 6 | 4 | Cdip1 | 810 | 6 | 0.71553925 | 1873726 | 0.55870166 |
| 1356 | 2.04850032 | 2.31E-04 | 0.48816199 | 6 | 4 | Cdh5 | 862.6666667 | 6 | 0.73787492 | 2223344 | 0.56763285 |
| 1357 | 2.06253989 | 2.44E-04 | 0.48483911 | 6 | 4 | Cdh4 | 859.5 | 6 | 0.73436503 | 2333896 | 0.56967485 |
| 1358 | 2.0319081 | 2.64E-04 | 0.49214824 | 6 | 4 | Cdca5 | 895.3333333 | 6 | 0.74202297 | 2565532 | 0.58414979 |
| 1359 | 2.0319081 | 2.64E-04 | 0.49214824 | 6 | 4 | Cdc42se2 | 895.3333333 | 6 | 0.74202297 | 2565532 | 0.58414979 |
| 1360 | 2.10465858 | 2.11E-04 | 0.47513645 | 6 | 4 | Cdc42se1 | 826.8333333 | 6 | 0.72383535 | 1986680 | 0.56026685 |
| 1361 | 2.27696235 | 1.48E-04 | 0.43918161 | 6 | 4 | Cdc25b | 744.6666667 | 6 | 0.68075941 | 1247534 | 0.55538959 |
| 1362 | 2.13784301 | 2.01E-04 | 0.46776119 | 6 | 4 | Cd8a | 810 | 6 | 0.71553925 | 1873726 | 0.55870166 |
| 1363 | 2.13784301 | 2.01E-04 | 0.46776119 | 6 | 4 | Cd300lb | 810 | 6 | 0.71553925 | 1873726 | 0.55870166 |
| 1364 | 2.0319081 | 2.64E-04 | 0.49214824 | 6 | 4 | Ccnj | 895.3333333 | 6 | 0.74202297 | 2565532 | 0.58414979 |
| 1365 | 2.0319081 | 2.64E-04 | 0.49214824 | 6 | 4 | Ccdc50 | 895.3333333 | 6 | 0.74202297 | 2565532 | 0.58414979 |
| 1366 | 2.04850032 | 2.31E-04 | 0.48816199 | 6 | 4 | Ccdc148 | 862.6666667 | 6 | 0.73787492 | 2223344 | 0.56763285 |
| 1367 | 2.06509253 | 2.34E-04 | 0.4842398 | 6 | 4 | Cbln3 | 842.6666667 | 6 | 0.73372687 | 2226548 | 0.55924695 |
| 1368 | 2.06253989 | 2.44E-04 | 0.48483911 | 6 | 4 | Bsnd | 859.5 | 6 | 0.73436503 | 2333896 | 0.56967485 |
| 1369 | 2.04850032 | 2.31E-04 | 0.48816199 | 6 | 4 | Brd3 | 862.6666667 | 6 | 0.73787492 | 2223344 | 0.56763285 |
| 1370 | 2.10465858 | 2.11E-04 | 0.47513645 | 6 | 4 | Brap | 826.8333333 | 6 | 0.72383535 | 1986680 | 0.56026685 |
| 1371 | 2.27696235 | 1.48E-04 | 0.43918161 | 6 | 4 | Bhlha15 | 744.6666667 | 6 | 0.68075941 | 1247534 | 0.55538959 |
| 1372 | 2.04850032 | 2.31E-04 | 0.48816199 | 6 | 4 | Bcl2l1 | 862.6666667 | 6 | 0.73787492 | 2223344 | 0.56763285 |
| 1373 | 2.04850032 | 2.31E-04 | 0.48816199 | 6 | 4 | BC018242 | 862.6666667 | 6 | 0.73787492 | 2223344 | 0.56763285 |
| 1374 | 2.13784301 | 2.01E-04 | 0.46776119 | 6 | 4 | Atp6v0a2 | 810 | 6 | 0.71553925 | 1873726 | 0.55870166 |
| 1375 | 2.07657945 | 2.23E-04 | 0.48156116 | 6 | 4 | Atp2b4 | 833.5 | 6 | 0.73085514 | 2095138 | 0.55648396 |
| 1376 | 2.0319081 | 2.53E-04 | 0.49214824 | 6 | 4 | Atg2b | 888.6666667 | 6 | 0.74202297 | 2464200 | 0.57979534 |
| 1377 | 2.05105297 | 2.43E-04 | 0.48755445 | 6 | 4 | Arsb | 871.8333333 | 6 | 0.73723676 | 2349452 | 0.57442832 |
| 1378 | 2.08296107 | 2.22E-04 | 0.48008578 | 6 | 4 | Arid3a | 836 | 6 | 0.72925973 | 2119300 | 0.56002683 |
| 1379 | 2.10465858 | 2.11E-04 | 0.47513645 | 6 | 4 | Aqp5 | 826.8333333 | 6 | 0.72383535 | 1986680 | 0.56026685 |
| 1380 | 2.0319081 | 2.64E-04 | 0.49214824 | 6 | 4 | Apol6 | 895.3333333 | 6 | 0.74202297 | 2565532 | 0.58414979 |
| 1381 | 2.05105297 | 2.43E-04 | 0.48755445 | 6 | 4 | Alx1 | 871.8333333 | 6 | 0.73723676 | 2349452 | 0.57442832 |
| 1382 | 2.0319081 | 2.53E-04 | 0.49214824 | 6 | 4 | Als2cl | 888.6666667 | 6 | 0.74202297 | 2464200 | 0.57979534 |
| 1383 | 2.07657945 | 2.23E-04 | 0.48156116 | 6 | 4 | Als2 | 833.5 | 6 | 0.73085514 | 2095138 | 0.55648396 |
| 1384 | 2.0319081 | 2.53E-04 | 0.49214824 | 6 | 4 | Aldh4a1 | 888.6666667 | 6 | 0.74202297 | 2464200 | 0.57979534 |
| 1385 | 2.04850032 | 2.31E-04 | 0.48816199 | 6 | 4 | Alad | 862.6666667 | 6 | 0.73787492 | 2223344 | 0.56763285 |
| 1386 | 2.04850032 | 2.31E-04 | 0.48816199 | 6 | 4 | Akt2 | 862.6666667 | 6 | 0.73787492 | 2223344 | 0.56763285 |
| 1387 | 2.06509253 | 2.34E-04 | 0.4842398 | 6 | 4 | Akap7 | 842.6666667 | 6 | 0.73372687 | 2226548 | 0.55924695 |
| 1388 | 2.13784301 | 2.01E-04 | 0.46776119 | 6 | 4 | Akap2 | 810 | 6 | 0.71553925 | 1873726 | 0.55870166 |
| 1389 | 2.0319081 | 2.64E-04 | 0.49214824 | 6 | 4 | Ak3 | 895.3333333 | 6 | 0.74202297 | 2565532 | 0.58414979 |
| 1390 | 2.0319081 | 2.64E-04 | 0.49214824 | 6 | 4 | Ahsa2 | 895.3333333 | 6 | 0.74202297 | 2565532 | 0.58414979 |
| 1391 | 2.13784301 | 2.01E-04 | 0.46776119 | 6 | 4 | Agtrap | 810 | 6 | 0.71553925 | 1873726 | 0.55870166 |
| 1392 | 2.06253989 | 2.44E-04 | 0.48483911 | 6 | 4 | Agrn | 859.5 | 6 | 0.73436503 | 2333896 | 0.56967485 |
| 1393 | 2.05105297 | 2.43E-04 | 0.48755445 | 6 | 4 | Afg3l1 | 871.8333333 | 6 | 0.73723676 | 2349452 | 0.57442832 |
| 1394 | 2.06253989 | 2.44E-04 | 0.48483911 | 6 | 4 | Adra2a | 859.5 | 6 | 0.73436503 | 2333896 | 0.56967485 |
| 1395 | 2.08296107 | 2.22E-04 | 0.48008578 | 6 | 4 | Adcy3 | 836 | 6 | 0.72925973 | 2119300 | 0.56002683 |
| 1396 | 2.10465858 | 2.11E-04 | 0.47513645 | 6 | 4 | Adarb2 | 826.8333333 | 6 | 0.72383535 | 1986680 | 0.56026685 |
| 1397 | 2.07657945 | 2.23E-04 | 0.48156116 | 6 | 4 | Adam11 | 833.5 | 6 | 0.73085514 | 2095138 | 0.55648396 |
| 1398 | 2.29738354 | 6.55E-05 | 0.43527778 | 6 | 4 | Acot11 | 587.3333333 | 6 | 0.67565412 | 565870 | 0.44318468 |
| 1399 | 2.08296107 | 2.22E-04 | 0.48008578 | 6 | 4 | Acad8 | 836 | 6 | 0.72925973 | 2119300 | 0.56002683 |
| 1400 | 2.06253989 | 2.44E-04 | 0.48483911 | 6 | 4 | Ablim1 | 859.5 | 6 | 0.73436503 | 2333896 | 0.56967485 |
| 1401 | 2.13784301 | 2.01E-04 | 0.46776119 | 6 | 4 | Abhd15 | 810 | 6 | 0.71553925 | 1873726 | 0.55870166 |
| 1402 | 2.10465858 | 2.11E-04 | 0.47513645 | 6 | 4 | Abcg4 | 826.8333333 | 6 | 0.72383535 | 1986680 | 0.56026685 |
| 1403 | 2.0319081 | 2.53E-04 | 0.49214824 | 6 | 4 | Abcf3 | 888.6666667 | 6 | 0.74202297 | 2464200 | 0.57979534 |
| 1404 | 2.22846203 | 1.57E-04 | 0.44873998 | 6 | 4 | A3galt2 | 761.5 | 6 | 0.69288449 | 1353430 | 0.55228758 |
| 1405 | 2.0319081 | 2.53E-04 | 0.49214824 | 6 | 4 | 9830107B12Rik | 888.6666667 | 6 | 0.74202297 | 2464200 | 0.57979534 |
| 1406 | 2.07657945 | 2.23E-04 | 0.48156116 | 6 | 4 | 9630033F20Rik | 833.5 | 6 | 0.73085514 | 2095138 | 0.55648396 |
| 1407 | 2.10465858 | 2.11E-04 | 0.47513645 | 6 | 4 | 9530068E07Rik | 826.8333333 | 6 | 0.72383535 | 1986680 | 0.56026685 |
| 1408 | 2.10465858 | 2.11E-04 | 0.47513645 | 6 | 4 | 7420426K07Rik | 826.8333333 | 6 | 0.72383535 | 1986680 | 0.56026685 |
| 1409 | 2.13784301 | 2.01E-04 | 0.46776119 | 6 | 4 | 4930571K23Rik | 810 | 6 | 0.71553925 | 1873726 | 0.55870166 |
| 1410 | 2.08296107 | 2.22E-04 | 0.48008578 | 6 | 4 | 2410004P03Rik | 836 | 6 | 0.72925973 | 2119300 | 0.56002683 |
| 1411 | 2.04850032 | 2.31E-04 | 0.48816199 | 6 | 4 | 1700101E01Rik | 862.6666667 | 6 | 0.73787492 | 2223344 | 0.56763285 |
| 1412 | 2.08296107 | 2.22E-04 | 0.48008578 | 6 | 4 | 1600014C10Rik | 836 | 6 | 0.72925973 | 2119300 | 0.56002683 |
| 882 | 2.30248883 | 4.85E-05 | 0.43431264 | 5 | 4 | Mapk10 | 678.6 | 5 | 0.67437779 | 491948 | 0.51333333 |
| 883 | 2.35226548 | 4.30E-05 | 0.42512208 | 5 | 4 | Map6 | 600.2 | 5 | 0.66193363 | 355134 | 0.46775956 |
| 884 | 2.41863433 | 4.12E-05 | 0.41345646 | 5 | 4 | Map4 | 526 | 5 | 0.64534142 | 342156 | 0.42717657 |
| 885 | 2.23101468 | 1.26E-04 | 0.44822654 | 5 | 4 | Map3k7cl | 895.6 | 5 | 0.69224633 | 1198022 | 0.65014535 |
| 886 | 2.23101468 | 1.26E-04 | 0.44822654 | 5 | 4 | Map3k14 | 895.6 | 5 | 0.69224633 | 1198022 | 0.65014535 |
| 887 | 2.40204212 | 9.21E-05 | 0.41631243 | 5 | 4 | Man2b1 | 856.4 | 5 | 0.64948947 | 847962 | 0.68872786 |
| 888 | 2.2744097 | 1.14E-04 | 0.43967452 | 5 | 4 | Mall | 887.6 | 5 | 0.68139757 | 1088002 | 0.66065574 |
| 889 | 2.2744097 | 1.14E-04 | 0.43967452 | 5 | 4 | Macrod1 | 887.6 | 5 | 0.68139757 | 1088002 | 0.66065574 |
| 890 | 2.15443523 | 1.34E-04 | 0.46415877 | 5 | 4 | Lypla2 | 930.6 | 5 | 0.71139119 | 1320118 | 0.64735376 |
| 891 | 2.33439694 | 1.04E-04 | 0.42837616 | 5 | 4 | Lrtm2 | 867.4 | 5 | 0.66640077 | 982408 | 0.66903475 |
| 892 | 2.15443523 | 1.34E-04 | 0.46415877 | 5 | 4 | Lrrc30 | 930.6 | 5 | 0.71139119 | 1320118 | 0.64735376 |
| 893 | 2.40204212 | 9.21E-05 | 0.41631243 | 5 | 4 | Lrp10 | 856.4 | 5 | 0.64948947 | 847962 | 0.68872786 |
| 894 | 2.40204212 | 9.21E-05 | 0.41631243 | 5 | 4 | Lrit1 | 856.4 | 5 | 0.64948947 | 847962 | 0.68872786 |
| 895 | 2.2744097 | 1.14E-04 | 0.43967452 | 5 | 4 | Lrch4 | 887.6 | 5 | 0.68139757 | 1088002 | 0.66065574 |
| 896 | 2.33439694 | 1.04E-04 | 0.42837616 | 5 | 4 | Lphn3 | 867.4 | 5 | 0.66640077 | 982408 | 0.66903475 |
| 897 | 2.15443523 | 1.34E-04 | 0.46415877 | 5 | 4 | Lonrf1 | 930.6 | 5 | 0.71139119 | 1320118 | 0.64735376 |
| 898 | 2.40204212 | 9.21E-05 | 0.41631243 | 5 | 4 | LOC101056654 | 856.4 | 5 | 0.64948947 | 847962 | 0.68872786 |
| 899 | 2.23101468 | 1.26E-04 | 0.44822654 | 5 | 4 | LOC101056016 | 895.6 | 5 | 0.69224633 | 1198022 | 0.65014535 |
| 900 | 2.15443523 | 1.34E-04 | 0.46415877 | 5 | 4 | LOC101055726 | 930.6 | 5 | 0.71139119 | 1320118 | 0.64735376 |
| 901 | 2.2744097 | 1.14E-04 | 0.43967452 | 5 | 4 | LOC101055647 | 887.6 | 5 | 0.68139757 | 1088002 | 0.66065574 |
| 902 | 2.2744097 | 1.14E-04 | 0.43967452 | 5 | 4 | LOC100861722 | 887.6 | 5 | 0.68139757 | 1088002 | 0.66065574 |
| 903 | 2.30248883 | 4.85E-05 | 0.43431264 | 5 | 4 | Lmtk2 | 678.6 | 5 | 0.67437779 | 491948 | 0.51333333 |
| 904 | 2.23101468 | 1.26E-04 | 0.44822654 | 5 | 4 | Lims2 | 895.6 | 5 | 0.69224633 | 1198022 | 0.65014535 |
| 905 | 2.15443523 | 1.34E-04 | 0.46415877 | 5 | 4 | Lig3 | 930.6 | 5 | 0.71139119 | 1320118 | 0.64735376 |
| 906 | 2.23101468 | 1.26E-04 | 0.44822654 | 5 | 4 | Lfng | 895.6 | 5 | 0.69224633 | 1198022 | 0.65014535 |
| 907 | 2.15443523 | 1.34E-04 | 0.46415877 | 5 | 4 | Ldoc1 | 930.6 | 5 | 0.71139119 | 1320118 | 0.64735376 |
| 908 | 2.2744097 | 1.14E-04 | 0.43967452 | 5 | 4 | Lancl1 | 887.6 | 5 | 0.68139757 | 1088002 | 0.66065574 |
| 909 | 2.23101468 | 1.26E-04 | 0.44822654 | 5 | 4 | Lactb2 | 895.6 | 5 | 0.69224633 | 1198022 | 0.65014535 |
| 910 | 2.30248883 | 4.85E-05 | 0.43431264 | 5 | 4 | Klhdc10 | 678.6 | 5 | 0.67437779 | 491948 | 0.51333333 |
| 911 | 2.2744097 | 1.14E-04 | 0.43967452 | 5 | 4 | Klc2 | 887.6 | 5 | 0.68139757 | 1088002 | 0.66065574 |
| 912 | 2.30248883 | 4.85E-05 | 0.43431264 | 5 | 4 | Kirrel | 678.6 | 5 | 0.67437779 | 491948 | 0.51333333 |
| 913 | 2.2744097 | 1.14E-04 | 0.43967452 | 5 | 4 | Kctd21 | 887.6 | 5 | 0.68139757 | 1088002 | 0.66065574 |
| 914 | 2.15443523 | 1.34E-04 | 0.46415877 | 5 | 4 | Kctd12b | 930.6 | 5 | 0.71139119 | 1320118 | 0.64735376 |
| 915 | 2.15443523 | 1.34E-04 | 0.46415877 | 5 | 4 | Kctd12 | 930.6 | 5 | 0.71139119 | 1320118 | 0.64735376 |
| 916 | 2.23101468 | 1.26E-04 | 0.44822654 | 5 | 4 | Kcnq1 | 895.6 | 5 | 0.69224633 | 1198022 | 0.65014535 |
| 917 | 2.23101468 | 1.26E-04 | 0.44822654 | 5 | 4 | Kcnk2 | 895.6 | 5 | 0.69224633 | 1198022 | 0.65014535 |
| 918 | 2.36375239 | 4.30E-05 | 0.42305616 | 5 | 4 | Kcnj5 | 561 | 5 | 0.6590619 | 360244 | 0.44025157 |
| 919 | 2.15443523 | 1.34E-04 | 0.46415877 | 5 | 4 | Kcnj10 | 930.6 | 5 | 0.71139119 | 1320118 | 0.64735376 |
| 920 | 2.15443523 | 1.34E-04 | 0.46415877 | 5 | 4 | Kcnd3 | 930.6 | 5 | 0.71139119 | 1320118 | 0.64735376 |
| 921 | 2.15443523 | 1.34E-04 | 0.46415877 | 5 | 4 | Kcnc4 | 930.6 | 5 | 0.71139119 | 1320118 | 0.64735376 |
| 922 | 2.30248883 | 4.85E-05 | 0.43431264 | 5 | 4 | Kcnc2 | 678.6 | 5 | 0.67437779 | 491948 | 0.51333333 |
| 923 | 2.2744097 | 1.14E-04 | 0.43967452 | 5 | 4 | Kat7 | 887.6 | 5 | 0.68139757 | 1088002 | 0.66065574 |
| 924 | 2.40204212 | 9.21E-05 | 0.41631243 | 5 | 4 | Kank2 | 856.4 | 5 | 0.64948947 | 847962 | 0.68872786 |
| 925 | 2.15443523 | 1.34E-04 | 0.46415877 | 5 | 4 | Jun | 930.6 | 5 | 0.71139119 | 1320118 | 0.64735376 |
| 926 | 2.33439694 | 1.04E-04 | 0.42837616 | 5 | 4 | Jph2 | 867.4 | 5 | 0.66640077 | 982408 | 0.66903475 |
| 927 | 2.23101468 | 1.26E-04 | 0.44822654 | 5 | 4 | Jdp2 | 895.6 | 5 | 0.69224633 | 1198022 | 0.65014535 |
| 928 | 2.30248883 | 4.85E-05 | 0.43431264 | 5 | 4 | Itsn1 | 678.6 | 5 | 0.67437779 | 491948 | 0.51333333 |
| 929 | 2.40204212 | 9.21E-05 | 0.41631243 | 5 | 4 | Irx4 | 856.4 | 5 | 0.64948947 | 847962 | 0.68872786 |
| 930 | 2.35226548 | 4.30E-05 | 0.42512208 | 5 | 4 | Irf1 | 600.2 | 5 | 0.66193363 | 355134 | 0.46775956 |
| 931 | 2.23101468 | 1.26E-04 | 0.44822654 | 5 | 4 | Iqce | 895.6 | 5 | 0.69224633 | 1198022 | 0.65014535 |
| 932 | 2.2744097 | 1.14E-04 | 0.43967452 | 5 | 4 | Ipo9 | 887.6 | 5 | 0.68139757 | 1088002 | 0.66065574 |
| 933 | 2.15443523 | 1.34E-04 | 0.46415877 | 5 | 4 | Ipo4 | 930.6 | 5 | 0.71139119 | 1320118 | 0.64735376 |
| 934 | 2.23101468 | 1.26E-04 | 0.44822654 | 5 | 4 | Ip6k3 | 895.6 | 5 | 0.69224633 | 1198022 | 0.65014535 |
| 935 | 2.15443523 | 1.34E-04 | 0.46415877 | 5 | 4 | Inf2 | 930.6 | 5 | 0.71139119 | 1320118 | 0.64735376 |
| 936 | 2.23101468 | 1.26E-04 | 0.44822654 | 5 | 4 | Impa1 | 895.6 | 5 | 0.69224633 | 1198022 | 0.65014535 |
| 937 | 2.40204212 | 9.21E-05 | 0.41631243 | 5 | 4 | Ilk | 856.4 | 5 | 0.64948947 | 847962 | 0.68872786 |
| 938 | 2.2744097 | 1.14E-04 | 0.43967452 | 5 | 4 | Il33 | 887.6 | 5 | 0.68139757 | 1088002 | 0.66065574 |
| 939 | 2.2744097 | 1.14E-04 | 0.43967452 | 5 | 4 | Il23a | 887.6 | 5 | 0.68139757 | 1088002 | 0.66065574 |
| 940 | 2.40204212 | 9.21E-05 | 0.41631243 | 5 | 4 | Ikzf4 | 856.4 | 5 | 0.64948947 | 847962 | 0.68872786 |
| 941 | 2.15443523 | 1.34E-04 | 0.46415877 | 5 | 4 | Igf1r | 930.6 | 5 | 0.71139119 | 1320118 | 0.64735376 |
| 942 | 2.2744097 | 1.14E-04 | 0.43967452 | 5 | 4 | Iffo1 | 887.6 | 5 | 0.68139757 | 1088002 | 0.66065574 |
| 943 | 2.2744097 | 1.14E-04 | 0.43967452 | 5 | 4 | Idh3a | 887.6 | 5 | 0.68139757 | 1088002 | 0.66065574 |
| 944 | 2.2744097 | 1.14E-04 | 0.43967452 | 5 | 4 | Hyou1 | 887.6 | 5 | 0.68139757 | 1088002 | 0.66065574 |
| 945 | 2.23101468 | 1.26E-04 | 0.44822654 | 5 | 4 | Hs3st3a1 | 895.6 | 5 | 0.69224633 | 1198022 | 0.65014535 |
| 946 | 2.15443523 | 1.34E-04 | 0.46415877 | 5 | 4 | Hs1bp3 | 930.6 | 5 | 0.71139119 | 1320118 | 0.64735376 |
| 947 | 2.15443523 | 1.34E-04 | 0.46415877 | 5 | 4 | Hpcal1 | 930.6 | 5 | 0.71139119 | 1320118 | 0.64735376 |
| 948 | 2.40204212 | 9.21E-05 | 0.41631243 | 5 | 4 | Hoxc8 | 856.4 | 5 | 0.64948947 | 847962 | 0.68872786 |
| 949 | 2.23101468 | 1.26E-04 | 0.44822654 | 5 | 4 | Homez | 895.6 | 5 | 0.69224633 | 1198022 | 0.65014535 |
| 950 | 2.15443523 | 1.34E-04 | 0.46415877 | 5 | 4 | Hmgcll1 | 930.6 | 5 | 0.71139119 | 1320118 | 0.64735376 |
| 951 | 2.33439694 | 1.04E-04 | 0.42837616 | 5 | 4 | Hlcs | 867.4 | 5 | 0.66640077 | 982408 | 0.66903475 |
| 952 | 2.23101468 | 1.26E-04 | 0.44822654 | 5 | 4 | Hip1r | 895.6 | 5 | 0.69224633 | 1198022 | 0.65014535 |
| 953 | 2.30248883 | 4.85E-05 | 0.43431264 | 5 | 4 | Hif1an | 678.6 | 5 | 0.67437779 | 491948 | 0.51333333 |
| 954 | 2.2744097 | 1.14E-04 | 0.43967452 | 5 | 4 | Hecw1 | 887.6 | 5 | 0.68139757 | 1088002 | 0.66065574 |
| 955 | 2.15443523 | 1.34E-04 | 0.46415877 | 5 | 4 | Heatr5a | 930.6 | 5 | 0.71139119 | 1320118 | 0.64735376 |
| 956 | 2.15443523 | 1.34E-04 | 0.46415877 | 5 | 4 | Hdac3 | 930.6 | 5 | 0.71139119 | 1320118 | 0.64735376 |
| 957 | 2.30248883 | 4.85E-05 | 0.43431264 | 5 | 4 | Has3 | 678.6 | 5 | 0.67437779 | 491948 | 0.51333333 |
| 958 | 2.33439694 | 1.04E-04 | 0.42837616 | 5 | 4 | Hadha | 867.4 | 5 | 0.66640077 | 982408 | 0.66903475 |
| 959 | 2.15443523 | 1.34E-04 | 0.46415877 | 5 | 4 | Gstm3 | 930.6 | 5 | 0.71139119 | 1320118 | 0.64735376 |
| 960 | 2.15443523 | 1.34E-04 | 0.46415877 | 5 | 4 | Gsg1l | 930.6 | 5 | 0.71139119 | 1320118 | 0.64735376 |
| 961 | 2.23101468 | 1.26E-04 | 0.44822654 | 5 | 4 | Gria4 | 895.6 | 5 | 0.69224633 | 1198022 | 0.65014535 |
| 962 | 2.40204212 | 9.21E-05 | 0.41631243 | 5 | 4 | Gria1 | 856.4 | 5 | 0.64948947 | 847962 | 0.68872786 |
| 963 | 2.2744097 | 1.14E-04 | 0.43967452 | 5 | 4 | Gramd3 | 887.6 | 5 | 0.68139757 | 1088002 | 0.66065574 |
| 964 | 2.30248883 | 4.85E-05 | 0.43431264 | 5 | 4 | Gprin1 | 678.6 | 5 | 0.67437779 | 491948 | 0.51333333 |
| 965 | 2.15443523 | 1.34E-04 | 0.46415877 | 5 | 4 | Gpr137b | 930.6 | 5 | 0.71139119 | 1320118 | 0.64735376 |
| 966 | 2.33439694 | 1.04E-04 | 0.42837616 | 5 | 4 | Gpr133 | 867.4 | 5 | 0.66640077 | 982408 | 0.66903475 |
| 967 | 2.15443523 | 1.34E-04 | 0.46415877 | 5 | 4 | Gpld1 | 930.6 | 5 | 0.71139119 | 1320118 | 0.64735376 |
| 968 | 2.40204212 | 9.21E-05 | 0.41631243 | 5 | 4 | Gpatch8 | 856.4 | 5 | 0.64948947 | 847962 | 0.68872786 |
| 969 | 2.69176771 | 4.04E-05 | 0.37150308 | 5 | 4 | Gorasp1 | 778 | 5 | 0.57705807 | 250920 | 0.76551724 |
| 970 | 2.15443523 | 1.34E-04 | 0.46415877 | 5 | 4 | Gnat2 | 930.6 | 5 | 0.71139119 | 1320118 | 0.64735376 |
| 971 | 2.33439694 | 1.04E-04 | 0.42837616 | 5 | 4 | Gnal | 867.4 | 5 | 0.66640077 | 982408 | 0.66903475 |
| 972 | 2.23101468 | 1.26E-04 | 0.44822654 | 5 | 4 | Gm6034 | 895.6 | 5 | 0.69224633 | 1198022 | 0.65014535 |
| 973 | 2.15443523 | 1.34E-04 | 0.46415877 | 5 | 4 | Gm5878 | 930.6 | 5 | 0.71139119 | 1320118 | 0.64735376 |
| 974 | 2.33439694 | 1.04E-04 | 0.42837616 | 5 | 4 | Gm4981 | 867.4 | 5 | 0.66640077 | 982408 | 0.66903475 |
| 975 | 2.2744097 | 1.14E-04 | 0.43967452 | 5 | 4 | Gm4847 | 887.6 | 5 | 0.68139757 | 1088002 | 0.66065574 |
| 976 | 2.23101468 | 1.26E-04 | 0.44822654 | 5 | 4 | Gm22 | 895.6 | 5 | 0.69224633 | 1198022 | 0.65014535 |
| 977 | 2.33439694 | 1.04E-04 | 0.42837616 | 5 | 4 | Gm10465 | 867.4 | 5 | 0.66640077 | 982408 | 0.66903475 |
| 978 | 2.23101468 | 1.26E-04 | 0.44822654 | 5 | 4 | Gja8 | 895.6 | 5 | 0.69224633 | 1198022 | 0.65014535 |
| 979 | 2.69176771 | 4.04E-05 | 0.37150308 | 5 | 4 | Git1 | 778 | 5 | 0.57705807 | 250920 | 0.76551724 |
| 980 | 2.15443523 | 1.34E-04 | 0.46415877 | 5 | 4 | Ghsr | 930.6 | 5 | 0.71139119 | 1320118 | 0.64735376 |
| 981 | 2.33439694 | 1.04E-04 | 0.42837616 | 5 | 4 | Gemin8 | 867.4 | 5 | 0.66640077 | 982408 | 0.66903475 |
| 982 | 2.15443523 | 1.34E-04 | 0.46415877 | 5 | 4 | Gcat | 930.6 | 5 | 0.71139119 | 1320118 | 0.64735376 |
| 983 | 2.15443523 | 1.34E-04 | 0.46415877 | 5 | 4 | Gbe1 | 930.6 | 5 | 0.71139119 | 1320118 | 0.64735376 |
| 984 | 2.35226548 | 4.30E-05 | 0.42512208 | 5 | 4 | Gas7 | 600.2 | 5 | 0.66193363 | 355134 | 0.46775956 |
| 985 | 2.33439694 | 1.04E-04 | 0.42837616 | 5 | 4 | Galt | 867.4 | 5 | 0.66640077 | 982408 | 0.66903475 |
| 986 | 2.37396299 | 4.23E-05 | 0.42123656 | 5 | 4 | Galnt10 | 569 | 5 | 0.65650925 | 347714 | 0.44936709 |
| 987 | 2.33439694 | 1.04E-04 | 0.42837616 | 5 | 4 | Gale | 867.4 | 5 | 0.66640077 | 982408 | 0.66903475 |
| 988 | 2.2744097 | 1.14E-04 | 0.43967452 | 5 | 4 | Gadl1 | 887.6 | 5 | 0.68139757 | 1088002 | 0.66065574 |
| 989 | 2.30248883 | 4.85E-05 | 0.43431264 | 5 | 4 | Gabpb2 | 678.6 | 5 | 0.67437779 | 491948 | 0.51333333 |
| 990 | 2.15443523 | 1.34E-04 | 0.46415877 | 5 | 4 | Gabarapl1 | 930.6 | 5 | 0.71139119 | 1320118 | 0.64735376 |
| 991 | 2.30248883 | 4.85E-05 | 0.43431264 | 5 | 4 | Fzd5 | 678.6 | 5 | 0.67437779 | 491948 | 0.51333333 |
| 992 | 2.40204212 | 9.21E-05 | 0.41631243 | 5 | 4 | Fsbp | 856.4 | 5 | 0.64948947 | 847962 | 0.68872786 |
| 993 | 2.23101468 | 1.26E-04 | 0.44822654 | 5 | 4 | Frem2 | 895.6 | 5 | 0.69224633 | 1198022 | 0.65014535 |
| 994 | 2.2744097 | 1.14E-04 | 0.43967452 | 5 | 4 | Foxr1 | 887.6 | 5 | 0.68139757 | 1088002 | 0.66065574 |
| 995 | 2.2744097 | 1.14E-04 | 0.43967452 | 5 | 4 | Foxp4 | 887.6 | 5 | 0.68139757 | 1088002 | 0.66065574 |
| 996 | 2.33439694 | 1.04E-04 | 0.42837616 | 5 | 4 | Foxp3 | 867.4 | 5 | 0.66640077 | 982408 | 0.66903475 |
| 997 | 2.15443523 | 1.34E-04 | 0.46415877 | 5 | 4 | Foxn3 | 930.6 | 5 | 0.71139119 | 1320118 | 0.64735376 |
| 998 | 2.15443523 | 1.34E-04 | 0.46415877 | 5 | 4 | Foxl1 | 930.6 | 5 | 0.71139119 | 1320118 | 0.64735376 |
| 999 | 2.40204212 | 9.21E-05 | 0.41631243 | 5 | 4 | Flot1 | 856.4 | 5 | 0.64948947 | 847962 | 0.68872786 |
| 1000 | 2.2744097 | 1.14E-04 | 0.43967452 | 5 | 4 | Flcn | 887.6 | 5 | 0.68139757 | 1088002 | 0.66065574 |
| 1001 | 2.33439694 | 1.04E-04 | 0.42837616 | 5 | 4 | Fkrp | 867.4 | 5 | 0.66640077 | 982408 | 0.66903475 |
| 1002 | 2.33439694 | 1.04E-04 | 0.42837616 | 5 | 4 | Fgf23 | 867.4 | 5 | 0.66640077 | 982408 | 0.66903475 |
| 1003 | 2.40204212 | 9.21E-05 | 0.41631243 | 5 | 4 | Fev | 856.4 | 5 | 0.64948947 | 847962 | 0.68872786 |
| 1004 | 2.33439694 | 1.04E-04 | 0.42837616 | 5 | 4 | Fcrl5 | 867.4 | 5 | 0.66640077 | 982408 | 0.66903475 |
| 1005 | 2.40204212 | 9.21E-05 | 0.41631243 | 5 | 4 | Fbxl16 | 856.4 | 5 | 0.64948947 | 847962 | 0.68872786 |
| 1006 | 2.15443523 | 1.34E-04 | 0.46415877 | 5 | 4 | Fam63b | 930.6 | 5 | 0.71139119 | 1320118 | 0.64735376 |
| 1007 | 2.40204212 | 9.21E-05 | 0.41631243 | 5 | 4 | Fam46b | 856.4 | 5 | 0.64948947 | 847962 | 0.68872786 |
| 1008 | 2.33439694 | 1.04E-04 | 0.42837616 | 5 | 4 | Fam19a3 | 867.4 | 5 | 0.66640077 | 982408 | 0.66903475 |
| 1009 | 2.33439694 | 1.04E-04 | 0.42837616 | 5 | 4 | Fam175b | 867.4 | 5 | 0.66640077 | 982408 | 0.66903475 |
| 1010 | 2.15443523 | 1.34E-04 | 0.46415877 | 5 | 4 | Fam174b | 930.6 | 5 | 0.71139119 | 1320118 | 0.64735376 |
| 1011 | 2.40204212 | 9.21E-05 | 0.41631243 | 5 | 4 | Fam161b | 856.4 | 5 | 0.64948947 | 847962 | 0.68872786 |
| 1012 | 2.23101468 | 1.26E-04 | 0.44822654 | 5 | 4 | Fam124a | 895.6 | 5 | 0.69224633 | 1198022 | 0.65014535 |
| 1013 | 2.2744097 | 1.14E-04 | 0.43967452 | 5 | 4 | Fam117a | 887.6 | 5 | 0.68139757 | 1088002 | 0.66065574 |
| 1014 | 2.2744097 | 1.14E-04 | 0.43967452 | 5 | 4 | Fam111a | 887.6 | 5 | 0.68139757 | 1088002 | 0.66065574 |
| 1015 | 2.23101468 | 1.26E-04 | 0.44822654 | 5 | 4 | Fads3 | 895.6 | 5 | 0.69224633 | 1198022 | 0.65014535 |
| 1016 | 2.33439694 | 1.04E-04 | 0.42837616 | 5 | 4 | F10 | 867.4 | 5 | 0.66640077 | 982408 | 0.66903475 |
| 1017 | 2.23101468 | 1.26E-04 | 0.44822654 | 5 | 4 | Exo1 | 895.6 | 5 | 0.69224633 | 1198022 | 0.65014535 |
| 1018 | 2.33439694 | 1.04E-04 | 0.42837616 | 5 | 4 | Etv5 | 867.4 | 5 | 0.66640077 | 982408 | 0.66903475 |
| 1019 | 2.15443523 | 1.34E-04 | 0.46415877 | 5 | 4 | Esrrg | 930.6 | 5 | 0.71139119 | 1320118 | 0.64735376 |
| 1020 | 2.15443523 | 1.34E-04 | 0.46415877 | 5 | 4 | Eri3 | 930.6 | 5 | 0.71139119 | 1320118 | 0.64735376 |
| 1021 | 2.23101468 | 1.26E-04 | 0.44822654 | 5 | 4 | Ergic2 | 895.6 | 5 | 0.69224633 | 1198022 | 0.65014535 |
| 1022 | 2.23101468 | 1.26E-04 | 0.44822654 | 5 | 4 | Ercc8 | 895.6 | 5 | 0.69224633 | 1198022 | 0.65014535 |
| 1023 | 2.23101468 | 1.26E-04 | 0.44822654 | 5 | 4 | Erbb2 | 895.6 | 5 | 0.69224633 | 1198022 | 0.65014535 |
| 1024 | 2.2744097 | 1.14E-04 | 0.43967452 | 5 | 4 | Epgn | 887.6 | 5 | 0.68139757 | 1088002 | 0.66065574 |
| 1025 | 2.69176771 | 4.04E-05 | 0.37150308 | 5 | 4 | Enc1 | 778 | 5 | 0.57705807 | 250920 | 0.76551724 |
| 1026 | 2.23101468 | 1.26E-04 | 0.44822654 | 5 | 4 | Emr4 | 895.6 | 5 | 0.69224633 | 1198022 | 0.65014535 |
| 1027 | 2.40204212 | 9.21E-05 | 0.41631243 | 5 | 4 | Elovl6 | 856.4 | 5 | 0.64948947 | 847962 | 0.68872786 |
| 1028 | 2.15443523 | 1.34E-04 | 0.46415877 | 5 | 4 | Eif5 | 930.6 | 5 | 0.71139119 | 1320118 | 0.64735376 |
| 1029 | 2.33439694 | 1.04E-04 | 0.42837616 | 5 | 4 | Eif3b | 867.4 | 5 | 0.66640077 | 982408 | 0.66903475 |
| 1030 | 2.15443523 | 1.34E-04 | 0.46415877 | 5 | 4 | Eid1 | 930.6 | 5 | 0.71139119 | 1320118 | 0.64735376 |
| 1031 | 2.15443523 | 1.34E-04 | 0.46415877 | 5 | 4 | Ehd1 | 930.6 | 5 | 0.71139119 | 1320118 | 0.64735376 |
| 1032 | 2.15443523 | 1.34E-04 | 0.46415877 | 5 | 4 | Efna5 | 930.6 | 5 | 0.71139119 | 1320118 | 0.64735376 |
| 1033 | 2.40204212 | 9.21E-05 | 0.41631243 | 5 | 4 | Eefsec | 856.4 | 5 | 0.64948947 | 847962 | 0.68872786 |
| 1034 | 2.30248883 | 4.85E-05 | 0.43431264 | 5 | 4 | Edaradd | 678.6 | 5 | 0.67437779 | 491948 | 0.51333333 |
| 1035 | 2.2744097 | 1.14E-04 | 0.43967452 | 5 | 4 | Ece2 | 887.6 | 5 | 0.68139757 | 1088002 | 0.66065574 |
| 1036 | 2.2744097 | 1.14E-04 | 0.43967452 | 5 | 4 | E2f6 | 887.6 | 5 | 0.68139757 | 1088002 | 0.66065574 |
| 1037 | 2.15443523 | 1.34E-04 | 0.46415877 | 5 | 4 | Dzank1 | 930.6 | 5 | 0.71139119 | 1320118 | 0.64735376 |
| 1038 | 2.15443523 | 1.34E-04 | 0.46415877 | 5 | 4 | Dusp11 | 930.6 | 5 | 0.71139119 | 1320118 | 0.64735376 |
| 1039 | 2.30248883 | 4.85E-05 | 0.43431264 | 5 | 4 | Dtx4 | 678.6 | 5 | 0.67437779 | 491948 | 0.51333333 |
| 1040 | 2.15443523 | 1.34E-04 | 0.46415877 | 5 | 4 | Dtd2 | 930.6 | 5 | 0.71139119 | 1320118 | 0.64735376 |
| 1041 | 2.23101468 | 1.26E-04 | 0.44822654 | 5 | 4 | Dpep1 | 895.6 | 5 | 0.69224633 | 1198022 | 0.65014535 |
| 1042 | 2.15443523 | 1.34E-04 | 0.46415877 | 5 | 4 | Dok5 | 930.6 | 5 | 0.71139119 | 1320118 | 0.64735376 |
| 1043 | 2.23101468 | 1.26E-04 | 0.44822654 | 5 | 4 | Dnm2 | 895.6 | 5 | 0.69224633 | 1198022 | 0.65014535 |
| 1044 | 2.40204212 | 9.21E-05 | 0.41631243 | 5 | 4 | Dnm1 | 856.4 | 5 | 0.64948947 | 847962 | 0.68872786 |
| 1045 | 2.2744097 | 1.14E-04 | 0.43967452 | 5 | 4 | Dnajb9 | 887.6 | 5 | 0.68139757 | 1088002 | 0.66065574 |
| 1046 | 2.23101468 | 1.26E-04 | 0.44822654 | 5 | 4 | Dnajb2 | 895.6 | 5 | 0.69224633 | 1198022 | 0.65014535 |
| 1047 | 2.40204212 | 9.21E-05 | 0.41631243 | 5 | 4 | Dlgap4 | 856.4 | 5 | 0.64948947 | 847962 | 0.68872786 |
| 1048 | 2.2744097 | 1.14E-04 | 0.43967452 | 5 | 4 | Dlgap3 | 887.6 | 5 | 0.68139757 | 1088002 | 0.66065574 |
| 1049 | 2.33439694 | 1.04E-04 | 0.42837616 | 5 | 4 | Dhdh | 867.4 | 5 | 0.66640077 | 982408 | 0.66903475 |
| 1050 | 2.2744097 | 1.14E-04 | 0.43967452 | 5 | 4 | Desi2 | 887.6 | 5 | 0.68139757 | 1088002 | 0.66065574 |
| 1051 | 2.23101468 | 1.26E-04 | 0.44822654 | 5 | 4 | Dedd | 895.6 | 5 | 0.69224633 | 1198022 | 0.65014535 |
| 1052 | 2.15443523 | 1.34E-04 | 0.46415877 | 5 | 4 | Ddx52 | 930.6 | 5 | 0.71139119 | 1320118 | 0.64735376 |
| 1053 | 2.2744097 | 1.14E-04 | 0.43967452 | 5 | 4 | Ddr1 | 887.6 | 5 | 0.68139757 | 1088002 | 0.66065574 |
| 1054 | 2.23101468 | 1.26E-04 | 0.44822654 | 5 | 4 | Ddhd1 | 895.6 | 5 | 0.69224633 | 1198022 | 0.65014535 |
| 1055 | 2.33439694 | 1.04E-04 | 0.42837616 | 5 | 4 | Dcp1b | 867.4 | 5 | 0.66640077 | 982408 | 0.66903475 |
| 1056 | 2.15443523 | 1.34E-04 | 0.46415877 | 5 | 4 | Dck | 930.6 | 5 | 0.71139119 | 1320118 | 0.64735376 |
| 1057 | 2.15443523 | 1.34E-04 | 0.46415877 | 5 | 4 | Dazap2 | 930.6 | 5 | 0.71139119 | 1320118 | 0.64735376 |
| 1058 | 2.2744097 | 1.14E-04 | 0.43967452 | 5 | 4 | Dapp1 | 887.6 | 5 | 0.68139757 | 1088002 | 0.66065574 |
| 1059 | 2.33439694 | 1.04E-04 | 0.42837616 | 5 | 4 | Dagla | 867.4 | 5 | 0.66640077 | 982408 | 0.66903475 |
| 1060 | 2.23101468 | 1.26E-04 | 0.44822654 | 5 | 4 | Dag1 | 895.6 | 5 | 0.69224633 | 1198022 | 0.65014535 |
| 1061 | 2.30248883 | 4.85E-05 | 0.43431264 | 5 | 4 | Dab2ip | 678.6 | 5 | 0.67437779 | 491948 | 0.51333333 |
| 1062 | 2.30248883 | 4.85E-05 | 0.43431264 | 5 | 4 | Dab1 | 678.6 | 5 | 0.67437779 | 491948 | 0.51333333 |
| 1063 | 2.15443523 | 1.34E-04 | 0.46415877 | 5 | 4 | D730048I06Rik | 930.6 | 5 | 0.71139119 | 1320118 | 0.64735376 |
| 1064 | 2.35226548 | 4.30E-05 | 0.42512208 | 5 | 4 | D630045J12Rik | 600.2 | 5 | 0.66193363 | 355134 | 0.46775956 |
| 1065 | 2.33439694 | 1.04E-04 | 0.42837616 | 5 | 4 | D430042O09Rik | 867.4 | 5 | 0.66640077 | 982408 | 0.66903475 |
| 1066 | 2.30248883 | 4.85E-05 | 0.43431264 | 5 | 4 | D3Bwg0562e | 678.6 | 5 | 0.67437779 | 491948 | 0.51333333 |
| 1067 | 2.2744097 | 1.14E-04 | 0.43967452 | 5 | 4 | D2Wsu81e | 887.6 | 5 | 0.68139757 | 1088002 | 0.66065574 |
| 1068 | 2.2744097 | 1.14E-04 | 0.43967452 | 5 | 4 | D230025D16Rik | 887.6 | 5 | 0.68139757 | 1088002 | 0.66065574 |
| 1069 | 2.2744097 | 1.14E-04 | 0.43967452 | 5 | 4 | Ctu1 | 887.6 | 5 | 0.68139757 | 1088002 | 0.66065574 |
| 1070 | 2.30248883 | 4.85E-05 | 0.43431264 | 5 | 4 | Ctsb | 678.6 | 5 | 0.67437779 | 491948 | 0.51333333 |
| 1071 | 2.40204212 | 9.21E-05 | 0.41631243 | 5 | 4 | Ctsa | 856.4 | 5 | 0.64948947 | 847962 | 0.68872786 |
| 1072 | 2.2744097 | 1.14E-04 | 0.43967452 | 5 | 4 | Ctps2 | 887.6 | 5 | 0.68139757 | 1088002 | 0.66065574 |
| 1073 | 2.15443523 | 1.34E-04 | 0.46415877 | 5 | 4 | Cstf2 | 930.6 | 5 | 0.71139119 | 1320118 | 0.64735376 |
| 1074 | 2.2744097 | 1.14E-04 | 0.43967452 | 5 | 4 | Csrp1 | 887.6 | 5 | 0.68139757 | 1088002 | 0.66065574 |
| 1075 | 2.33439694 | 1.04E-04 | 0.42837616 | 5 | 4 | Csnk1e | 867.4 | 5 | 0.66640077 | 982408 | 0.66903475 |
| 1076 | 2.40204212 | 9.21E-05 | 0.41631243 | 5 | 4 | Csdc2 | 856.4 | 5 | 0.64948947 | 847962 | 0.68872786 |
| 1077 | 2.15443523 | 1.34E-04 | 0.46415877 | 5 | 4 | Crbn | 930.6 | 5 | 0.71139119 | 1320118 | 0.64735376 |
| 1078 | 2.15443523 | 1.34E-04 | 0.46415877 | 5 | 4 | Cox19 | 930.6 | 5 | 0.71139119 | 1320118 | 0.64735376 |
| 1079 | 2.15443523 | 1.34E-04 | 0.46415877 | 5 | 4 | Col8a2 | 930.6 | 5 | 0.71139119 | 1320118 | 0.64735376 |
| 1080 | 2.2744097 | 1.14E-04 | 0.43967452 | 5 | 4 | Col6a5 | 887.6 | 5 | 0.68139757 | 1088002 | 0.66065574 |
| 1081 | 2.15443523 | 1.34E-04 | 0.46415877 | 5 | 4 | Col4a1 | 930.6 | 5 | 0.71139119 | 1320118 | 0.64735376 |
| 1082 | 2.15443523 | 1.34E-04 | 0.46415877 | 5 | 4 | Cnot4 | 930.6 | 5 | 0.71139119 | 1320118 | 0.64735376 |
| 1083 | 2.2744097 | 1.14E-04 | 0.43967452 | 5 | 4 | Cnnm1 | 887.6 | 5 | 0.68139757 | 1088002 | 0.66065574 |
| 1084 | 2.40204212 | 9.21E-05 | 0.41631243 | 5 | 4 | Cnih2 | 856.4 | 5 | 0.64948947 | 847962 | 0.68872786 |
| 1085 | 2.15443523 | 1.34E-04 | 0.46415877 | 5 | 4 | Cnih | 930.6 | 5 | 0.71139119 | 1320118 | 0.64735376 |
| 1086 | 2.15443523 | 1.34E-04 | 0.46415877 | 5 | 4 | Cnep1r1 | 930.6 | 5 | 0.71139119 | 1320118 | 0.64735376 |
| 1087 | 2.2744097 | 1.14E-04 | 0.43967452 | 5 | 4 | Cndp1 | 887.6 | 5 | 0.68139757 | 1088002 | 0.66065574 |
| 1088 | 2.33439694 | 1.04E-04 | 0.42837616 | 5 | 4 | Cltb | 867.4 | 5 | 0.66640077 | 982408 | 0.66903475 |
| 1089 | 2.15443523 | 1.34E-04 | 0.46415877 | 5 | 4 | Clns1a | 930.6 | 5 | 0.71139119 | 1320118 | 0.64735376 |
| 1090 | 2.35226548 | 4.30E-05 | 0.42512208 | 5 | 4 | Cln8 | 600.2 | 5 | 0.66193363 | 355134 | 0.46775956 |
| 1091 | 2.30248883 | 4.85E-05 | 0.43431264 | 5 | 4 | Clmn | 678.6 | 5 | 0.67437779 | 491948 | 0.51333333 |
| 1092 | 2.40204212 | 9.21E-05 | 0.41631243 | 5 | 4 | Clip2 | 856.4 | 5 | 0.64948947 | 847962 | 0.68872786 |
| 1093 | 2.33439694 | 1.04E-04 | 0.42837616 | 5 | 4 | Clec9a | 867.4 | 5 | 0.66640077 | 982408 | 0.66903475 |
| 1094 | 2.15443523 | 1.34E-04 | 0.46415877 | 5 | 4 | Cldn9 | 930.6 | 5 | 0.71139119 | 1320118 | 0.64735376 |
| 1095 | 2.40204212 | 9.21E-05 | 0.41631243 | 5 | 4 | Chst14 | 856.4 | 5 | 0.64948947 | 847962 | 0.68872786 |
| 1096 | 2.30248883 | 4.85E-05 | 0.43431264 | 5 | 4 | Chrna4 | 678.6 | 5 | 0.67437779 | 491948 | 0.51333333 |
| 1097 | 2.33439694 | 1.04E-04 | 0.42837616 | 5 | 4 | Chrm3 | 867.4 | 5 | 0.66640077 | 982408 | 0.66903475 |
| 1098 | 2.37396299 | 4.23E-05 | 0.42123656 | 5 | 4 | Chdh | 569 | 5 | 0.65650925 | 347714 | 0.44936709 |
| 1099 | 2.40204212 | 9.21E-05 | 0.41631243 | 5 | 4 | Chat | 856.4 | 5 | 0.64948947 | 847962 | 0.68872786 |
| 1100 | 2.2744097 | 1.14E-04 | 0.43967452 | 5 | 4 | Cggbp1 | 887.6 | 5 | 0.68139757 | 1088002 | 0.66065574 |
| 1101 | 2.15443523 | 1.34E-04 | 0.46415877 | 5 | 4 | Ces3a | 930.6 | 5 | 0.71139119 | 1320118 | 0.64735376 |
| 1102 | 2.15443523 | 1.34E-04 | 0.46415877 | 5 | 4 | Cep164 | 930.6 | 5 | 0.71139119 | 1320118 | 0.64735376 |
| 1103 | 2.15443523 | 1.34E-04 | 0.46415877 | 5 | 4 | Cep152 | 930.6 | 5 | 0.71139119 | 1320118 | 0.64735376 |
| 1104 | 2.2744097 | 1.14E-04 | 0.43967452 | 5 | 4 | Celf3 | 887.6 | 5 | 0.68139757 | 1088002 | 0.66065574 |
| 1105 | 2.2744097 | 1.14E-04 | 0.43967452 | 5 | 4 | Cdsn | 887.6 | 5 | 0.68139757 | 1088002 | 0.66065574 |
| 1106 | 2.33439694 | 1.04E-04 | 0.42837616 | 5 | 4 | Cdk9 | 867.4 | 5 | 0.66640077 | 982408 | 0.66903475 |
| 1107 | 2.2744097 | 1.14E-04 | 0.43967452 | 5 | 4 | Cdh12 | 887.6 | 5 | 0.68139757 | 1088002 | 0.66065574 |
| 1108 | 2.33439694 | 1.04E-04 | 0.42837616 | 5 | 4 | Cd74 | 867.4 | 5 | 0.66640077 | 982408 | 0.66903475 |
| 1109 | 2.2744097 | 1.14E-04 | 0.43967452 | 5 | 4 | Cd300ld | 887.6 | 5 | 0.68139757 | 1088002 | 0.66065574 |
| 1110 | 2.15443523 | 1.34E-04 | 0.46415877 | 5 | 4 | Cd300a | 930.6 | 5 | 0.71139119 | 1320118 | 0.64735376 |
| 1111 | 2.23101468 | 1.26E-04 | 0.44822654 | 5 | 4 | Cd276 | 895.6 | 5 | 0.69224633 | 1198022 | 0.65014535 |
| 1112 | 2.15443523 | 1.34E-04 | 0.46415877 | 5 | 4 | Cd151 | 930.6 | 5 | 0.71139119 | 1320118 | 0.64735376 |
| 1113 | 2.15443523 | 1.34E-04 | 0.46415877 | 5 | 4 | Ccser2 | 930.6 | 5 | 0.71139119 | 1320118 | 0.64735376 |
| 1114 | 2.23101468 | 1.26E-04 | 0.44822654 | 5 | 4 | Ccnyl1 | 895.6 | 5 | 0.69224633 | 1198022 | 0.65014535 |
| 1115 | 2.40204212 | 9.21E-05 | 0.41631243 | 5 | 4 | Ccny | 856.4 | 5 | 0.64948947 | 847962 | 0.68872786 |
| 1116 | 2.23101468 | 1.26E-04 | 0.44822654 | 5 | 4 | Ccdc166 | 895.6 | 5 | 0.69224633 | 1198022 | 0.65014535 |
| 1117 | 2.30248883 | 4.85E-05 | 0.43431264 | 5 | 4 | Cbx6 | 678.6 | 5 | 0.67437779 | 491948 | 0.51333333 |
| 1118 | 2.30248883 | 4.85E-05 | 0.43431264 | 5 | 4 | Cbfa2t3 | 678.6 | 5 | 0.67437779 | 491948 | 0.51333333 |
| 1119 | 2.34971283 | 4.41E-05 | 0.42558392 | 5 | 4 | Cask | 589.2 | 5 | 0.66257179 | 366284 | 0.45845674 |
| 1120 | 2.15443523 | 1.34E-04 | 0.46415877 | 5 | 4 | Casd1 | 930.6 | 5 | 0.71139119 | 1320118 | 0.64735376 |
| 1121 | 2.15443523 | 1.34E-04 | 0.46415877 | 5 | 4 | Cars | 930.6 | 5 | 0.71139119 | 1320118 | 0.64735376 |
| 1122 | 2.15443523 | 1.34E-04 | 0.46415877 | 5 | 4 | Car12 | 930.6 | 5 | 0.71139119 | 1320118 | 0.64735376 |
| 1123 | 2.15443523 | 1.34E-04 | 0.46415877 | 5 | 4 | Caprin1 | 930.6 | 5 | 0.71139119 | 1320118 | 0.64735376 |
| 1124 | 2.23101468 | 1.26E-04 | 0.44822654 | 5 | 4 | Camk2a | 895.6 | 5 | 0.69224633 | 1198022 | 0.65014535 |
| 1125 | 2.23101468 | 1.26E-04 | 0.44822654 | 5 | 4 | Calcrl | 895.6 | 5 | 0.69224633 | 1198022 | 0.65014535 |
| 1126 | 2.15443523 | 1.34E-04 | 0.46415877 | 5 | 4 | Cadm3 | 930.6 | 5 | 0.71139119 | 1320118 | 0.64735376 |
| 1127 | 2.2744097 | 1.14E-04 | 0.43967452 | 5 | 4 | Cacng3 | 887.6 | 5 | 0.68139757 | 1088002 | 0.66065574 |
| 1128 | 2.40204212 | 9.21E-05 | 0.41631243 | 5 | 4 | Cacna1h | 856.4 | 5 | 0.64948947 | 847962 | 0.68872786 |
| 1129 | 2.15443523 | 1.34E-04 | 0.46415877 | 5 | 4 | Cab39 | 930.6 | 5 | 0.71139119 | 1320118 | 0.64735376 |
| 1130 | 2.2744097 | 1.14E-04 | 0.43967452 | 5 | 4 | C6 | 887.6 | 5 | 0.68139757 | 1088002 | 0.66065574 |
| 1131 | 2.15443523 | 1.34E-04 | 0.46415877 | 5 | 4 | C2cd5 | 930.6 | 5 | 0.71139119 | 1320118 | 0.64735376 |
| 1132 | 2.23101468 | 1.26E-04 | 0.44822654 | 5 | 4 | C030046E11Rik | 895.6 | 5 | 0.69224633 | 1198022 | 0.65014535 |
| 1133 | 2.33439694 | 1.04E-04 | 0.42837616 | 5 | 4 | Btbd11 | 867.4 | 5 | 0.66640077 | 982408 | 0.66903475 |
| 1134 | 2.40204212 | 9.21E-05 | 0.41631243 | 5 | 4 | Brms1l | 856.4 | 5 | 0.64948947 | 847962 | 0.68872786 |
| 1135 | 2.2744097 | 1.14E-04 | 0.43967452 | 5 | 4 | Bnc1 | 887.6 | 5 | 0.68139757 | 1088002 | 0.66065574 |
| 1136 | 2.33439694 | 1.04E-04 | 0.42837616 | 5 | 4 | Bmp7 | 867.4 | 5 | 0.66640077 | 982408 | 0.66903475 |
| 1137 | 2.15443523 | 1.34E-04 | 0.46415877 | 5 | 4 | Bcr | 930.6 | 5 | 0.71139119 | 1320118 | 0.64735376 |
| 1138 | 2.2744097 | 1.14E-04 | 0.43967452 | 5 | 4 | Bcas3 | 887.6 | 5 | 0.68139757 | 1088002 | 0.66065574 |
| 1139 | 2.15443523 | 1.34E-04 | 0.46415877 | 5 | 4 | Bcam | 930.6 | 5 | 0.71139119 | 1320118 | 0.64735376 |
| 1140 | 2.15443523 | 1.34E-04 | 0.46415877 | 5 | 4 | BC089597 | 930.6 | 5 | 0.71139119 | 1320118 | 0.64735376 |
| 1141 | 2.2744097 | 1.14E-04 | 0.43967452 | 5 | 4 | BC048403 | 887.6 | 5 | 0.68139757 | 1088002 | 0.66065574 |
| 1142 | 2.15443523 | 1.34E-04 | 0.46415877 | 5 | 4 | BC035044 | 930.6 | 5 | 0.71139119 | 1320118 | 0.64735376 |
| 1143 | 2.30248883 | 4.85E-05 | 0.43431264 | 5 | 4 | Bbs9 | 678.6 | 5 | 0.67437779 | 491948 | 0.51333333 |
| 1144 | 2.2744097 | 1.14E-04 | 0.43967452 | 5 | 4 | Bak1 | 887.6 | 5 | 0.68139757 | 1088002 | 0.66065574 |
| 1145 | 2.23101468 | 1.26E-04 | 0.44822654 | 5 | 4 | Baiap2 | 895.6 | 5 | 0.69224633 | 1198022 | 0.65014535 |
| 1146 | 2.23101468 | 1.26E-04 | 0.44822654 | 5 | 4 | Bace1 | 895.6 | 5 | 0.69224633 | 1198022 | 0.65014535 |
| 1147 | 2.15443523 | 1.34E-04 | 0.46415877 | 5 | 4 | B4galt1 | 930.6 | 5 | 0.71139119 | 1320118 | 0.64735376 |
| 1148 | 2.40204212 | 9.21E-05 | 0.41631243 | 5 | 4 | B3gnt9 | 856.4 | 5 | 0.64948947 | 847962 | 0.68872786 |
| 1149 | 2.15443523 | 1.34E-04 | 0.46415877 | 5 | 4 | B3galt1 | 930.6 | 5 | 0.71139119 | 1320118 | 0.64735376 |
| 1150 | 2.2744097 | 1.14E-04 | 0.43967452 | 5 | 4 | AW551984 | 887.6 | 5 | 0.68139757 | 1088002 | 0.66065574 |
| 1151 | 2.40204212 | 9.21E-05 | 0.41631243 | 5 | 4 | AU023871 | 856.4 | 5 | 0.64948947 | 847962 | 0.68872786 |
| 1152 | 2.2744097 | 1.14E-04 | 0.43967452 | 5 | 4 | Atp6v0a4 | 887.6 | 5 | 0.68139757 | 1088002 | 0.66065574 |
| 1153 | 2.2744097 | 1.14E-04 | 0.43967452 | 5 | 4 | Atp2a3 | 887.6 | 5 | 0.68139757 | 1088002 | 0.66065574 |
| 1154 | 2.33439694 | 1.04E-04 | 0.42837616 | 5 | 4 | Atoh8 | 867.4 | 5 | 0.66640077 | 982408 | 0.66903475 |
| 1155 | 2.15443523 | 1.34E-04 | 0.46415877 | 5 | 4 | Atmin | 930.6 | 5 | 0.71139119 | 1320118 | 0.64735376 |
| 1156 | 2.23101468 | 1.26E-04 | 0.44822654 | 5 | 4 | Athl1 | 895.6 | 5 | 0.69224633 | 1198022 | 0.65014535 |
| 1157 | 2.40204212 | 9.21E-05 | 0.41631243 | 5 | 4 | Ate1 | 856.4 | 5 | 0.64948947 | 847962 | 0.68872786 |
| 1158 | 2.15443523 | 1.34E-04 | 0.46415877 | 5 | 4 | Asph | 930.6 | 5 | 0.71139119 | 1320118 | 0.64735376 |
| 1159 | 2.33439694 | 1.04E-04 | 0.42837616 | 5 | 4 | Arpp21 | 867.4 | 5 | 0.66640077 | 982408 | 0.66903475 |
| 1160 | 2.40204212 | 9.21E-05 | 0.41631243 | 5 | 4 | Arpp19 | 856.4 | 5 | 0.64948947 | 847962 | 0.68872786 |
| 1161 | 2.40204212 | 9.21E-05 | 0.41631243 | 5 | 4 | Arl3 | 856.4 | 5 | 0.64948947 | 847962 | 0.68872786 |
| 1162 | 2.23101468 | 1.26E-04 | 0.44822654 | 5 | 4 | Arhgef5 | 895.6 | 5 | 0.69224633 | 1198022 | 0.65014535 |
| 1163 | 2.15443523 | 1.34E-04 | 0.46415877 | 5 | 4 | Arhgap4 | 930.6 | 5 | 0.71139119 | 1320118 | 0.64735376 |
| 1164 | 2.15443523 | 1.34E-04 | 0.46415877 | 5 | 4 | Arfgap2 | 930.6 | 5 | 0.71139119 | 1320118 | 0.64735376 |
| 1165 | 2.15443523 | 1.34E-04 | 0.46415877 | 5 | 4 | Arf3 | 930.6 | 5 | 0.71139119 | 1320118 | 0.64735376 |
| 1166 | 2.15443523 | 1.34E-04 | 0.46415877 | 5 | 4 | Arc | 930.6 | 5 | 0.71139119 | 1320118 | 0.64735376 |
| 1167 | 2.30248883 | 4.85E-05 | 0.43431264 | 5 | 4 | Ap3s2 | 678.6 | 5 | 0.67437779 | 491948 | 0.51333333 |
| 1168 | 2.15443523 | 1.34E-04 | 0.46415877 | 5 | 4 | Aoah | 930.6 | 5 | 0.71139119 | 1320118 | 0.64735376 |
| 1169 | 2.40204212 | 9.21E-05 | 0.41631243 | 5 | 4 | Ankrd12 | 856.4 | 5 | 0.64948947 | 847962 | 0.68872786 |
| 1170 | 2.40204212 | 9.21E-05 | 0.41631243 | 5 | 4 | Anapc15 | 856.4 | 5 | 0.64948947 | 847962 | 0.68872786 |
| 1171 | 2.33439694 | 1.04E-04 | 0.42837616 | 5 | 4 | Amer3 | 867.4 | 5 | 0.66640077 | 982408 | 0.66903475 |
| 1172 | 2.15443523 | 1.34E-04 | 0.46415877 | 5 | 4 | Aldh8a1 | 930.6 | 5 | 0.71139119 | 1320118 | 0.64735376 |
| 1173 | 2.15443523 | 1.34E-04 | 0.46415877 | 5 | 4 | Akt1 | 930.6 | 5 | 0.71139119 | 1320118 | 0.64735376 |
| 1174 | 2.15443523 | 1.34E-04 | 0.46415877 | 5 | 4 | AI467606 | 930.6 | 5 | 0.71139119 | 1320118 | 0.64735376 |
| 1175 | 2.15443523 | 1.34E-04 | 0.46415877 | 5 | 4 | Ahi1 | 930.6 | 5 | 0.71139119 | 1320118 | 0.64735376 |
| 1176 | 2.15443523 | 1.34E-04 | 0.46415877 | 5 | 4 | Agpat3 | 930.6 | 5 | 0.71139119 | 1320118 | 0.64735376 |
| 1177 | 2.15443523 | 1.34E-04 | 0.46415877 | 5 | 4 | Agl | 930.6 | 5 | 0.71139119 | 1320118 | 0.64735376 |
| 1178 | 2.40204212 | 9.21E-05 | 0.41631243 | 5 | 4 | Agap2 | 856.4 | 5 | 0.64948947 | 847962 | 0.68872786 |
| 1179 | 2.23101468 | 1.26E-04 | 0.44822654 | 5 | 4 | Ado | 895.6 | 5 | 0.69224633 | 1198022 | 0.65014535 |
| 1180 | 2.30248883 | 4.85E-05 | 0.43431264 | 5 | 4 | Add2 | 678.6 | 5 | 0.67437779 | 491948 | 0.51333333 |
| 1181 | 2.23101468 | 1.26E-04 | 0.44822654 | 5 | 4 | Adcyap1 | 895.6 | 5 | 0.69224633 | 1198022 | 0.65014535 |
| 1182 | 2.33439694 | 1.04E-04 | 0.42837616 | 5 | 4 | Adcy9 | 867.4 | 5 | 0.66640077 | 982408 | 0.66903475 |
| 1183 | 2.23101468 | 1.26E-04 | 0.44822654 | 5 | 4 | Adcy7 | 895.6 | 5 | 0.69224633 | 1198022 | 0.65014535 |
| 1184 | 2.33439694 | 1.04E-04 | 0.42837616 | 5 | 4 | Adap2 | 867.4 | 5 | 0.66640077 | 982408 | 0.66903475 |
| 1185 | 2.15443523 | 1.34E-04 | 0.46415877 | 5 | 4 | Adamts6 | 930.6 | 5 | 0.71139119 | 1320118 | 0.64735376 |
| 1186 | 2.30248883 | 4.85E-05 | 0.43431264 | 5 | 4 | Adam22 | 678.6 | 5 | 0.67437779 | 491948 | 0.51333333 |
| 1187 | 2.23101468 | 1.26E-04 | 0.44822654 | 5 | 4 | Adam17 | 895.6 | 5 | 0.69224633 | 1198022 | 0.65014535 |
| 1188 | 2.30248883 | 4.85E-05 | 0.43431264 | 5 | 4 | Adam12 | 678.6 | 5 | 0.67437779 | 491948 | 0.51333333 |
| 1189 | 2.15443523 | 1.34E-04 | 0.46415877 | 5 | 4 | Actn4 | 930.6 | 5 | 0.71139119 | 1320118 | 0.64735376 |
| 1190 | 2.23101468 | 1.26E-04 | 0.44822654 | 5 | 4 | Acsf2 | 895.6 | 5 | 0.69224633 | 1198022 | 0.65014535 |
| 1191 | 2.40204212 | 9.21E-05 | 0.41631243 | 5 | 4 | Acnat1 | 856.4 | 5 | 0.64948947 | 847962 | 0.68872786 |
| 1192 | 2.2744097 | 1.14E-04 | 0.43967452 | 5 | 4 | Acat1 | 887.6 | 5 | 0.68139757 | 1088002 | 0.66065574 |
| 1193 | 2.30248883 | 4.85E-05 | 0.43431264 | 5 | 4 | Abl2 | 678.6 | 5 | 0.67437779 | 491948 | 0.51333333 |
| 1194 | 2.40204212 | 9.21E-05 | 0.41631243 | 5 | 4 | Abhd6 | 856.4 | 5 | 0.64948947 | 847962 | 0.68872786 |
| 1195 | 2.2744097 | 1.14E-04 | 0.43967452 | 5 | 4 | Abhd17c | 887.6 | 5 | 0.68139757 | 1088002 | 0.66065574 |
| 1196 | 2.15443523 | 1.34E-04 | 0.46415877 | 5 | 4 | Abcd2 | 930.6 | 5 | 0.71139119 | 1320118 | 0.64735376 |
| 1197 | 2.33439694 | 1.04E-04 | 0.42837616 | 5 | 4 | Abat | 867.4 | 5 | 0.66640077 | 982408 | 0.66903475 |
| 1198 | 2.2744097 | 1.14E-04 | 0.43967452 | 5 | 4 | A230046K03Rik | 887.6 | 5 | 0.68139757 | 1088002 | 0.66065574 |
| 1199 | 2.2744097 | 1.14E-04 | 0.43967452 | 5 | 4 | 9030025P20Rik | 887.6 | 5 | 0.68139757 | 1088002 | 0.66065574 |
| 1200 | 2.34971283 | 4.41E-05 | 0.42558392 | 5 | 4 | 6430548M08Rik | 589.2 | 5 | 0.66257179 | 366284 | 0.45845674 |
| 1201 | 2.15443523 | 1.34E-04 | 0.46415877 | 5 | 4 | 6330403A02Rik | 930.6 | 5 | 0.71139119 | 1320118 | 0.64735376 |
| 1202 | 2.40204212 | 9.21E-05 | 0.41631243 | 5 | 4 | 4933434I20Rik | 856.4 | 5 | 0.64948947 | 847962 | 0.68872786 |
| 1203 | 2.23101468 | 1.26E-04 | 0.44822654 | 5 | 4 | 4933427D14Rik | 895.6 | 5 | 0.69224633 | 1198022 | 0.65014535 |
| 1204 | 2.2744097 | 1.14E-04 | 0.43967452 | 5 | 4 | 4930432M17Rik | 887.6 | 5 | 0.68139757 | 1088002 | 0.66065574 |
| 1205 | 2.23101468 | 1.26E-04 | 0.44822654 | 5 | 4 | 4921536K21Rik | 895.6 | 5 | 0.69224633 | 1198022 | 0.65014535 |
| 1206 | 2.33439694 | 1.04E-04 | 0.42837616 | 5 | 4 | 2010109I03Rik | 867.4 | 5 | 0.66640077 | 982408 | 0.66903475 |
| 1207 | 2.40204212 | 9.21E-05 | 0.41631243 | 5 | 4 | 43534 | 856.4 | 5 | 0.64948947 | 847962 | 0.68872786 |
| 514 | 2.79004467 | 1.00E-08 | 0.3584172 | 4 | 4 | Mars2 | 939.75 | 4 | 0.55248883 | 18 | 0.99973376 |
| 515 | 2.4275686 | 2.84E-05 | 0.41193481 | 4 | 4 | Mapk8ip3 | 624.75 | 4 | 0.64310785 | 286214 | 0.51001635 |
| 516 | 2.79004467 | 1.00E-08 | 0.3584172 | 4 | 4 | Mapk8ip1 | 939.75 | 4 | 0.55248883 | 18 | 0.99973376 |
| 517 | 2.79004467 | 1.00E-08 | 0.3584172 | 4 | 4 | Map7 | 939.75 | 4 | 0.55248883 | 18 | 0.99973376 |
| 518 | 2.79004467 | 1.00E-08 | 0.3584172 | 4 | 4 | Map3k15 | 939.75 | 4 | 0.55248883 | 18 | 0.99973376 |
| 519 | 2.53095086 | 2.33E-05 | 0.39510842 | 4 | 4 | Map2k7 | 477.75 | 4 | 0.61726228 | 183940 | 0.41746935 |
| 520 | 2.37141034 | 2.95E-05 | 0.42168999 | 4 | 4 | Manea | 668.5 | 4 | 0.65714742 | 301340 | 0.52683504 |
| 521 | 2.79004467 | 1.00E-08 | 0.3584172 | 4 | 4 | Lzts3 | 939.75 | 4 | 0.55248883 | 18 | 0.99973376 |
| 522 | 2.38289726 | 2.86E-05 | 0.4196572 | 4 | 4 | Luzp1 | 678.5 | 4 | 0.65427569 | 288318 | 0.53855326 |
| 523 | 2.79004467 | 1.00E-08 | 0.3584172 | 4 | 4 | Lsm4 | 939.75 | 4 | 0.55248883 | 18 | 0.99973376 |
| 524 | 2.79004467 | 1.00E-08 | 0.3584172 | 4 | 4 | Lrrc66 | 939.75 | 4 | 0.55248883 | 18 | 0.99973376 |
| 525 | 2.3586471 | 3.03E-05 | 0.42397186 | 4 | 4 | Lrrc28 | 703.75 | 4 | 0.66033823 | 306586 | 0.55031323 |
| 526 | 2.3586471 | 3.03E-05 | 0.42397186 | 4 | 4 | Loxl1 | 703.75 | 4 | 0.66033823 | 306586 | 0.55031323 |
| 527 | 2.79004467 | 1.00E-08 | 0.3584172 | 4 | 4 | LOC101056015 | 939.75 | 4 | 0.55248883 | 18 | 0.99973376 |
| 528 | 2.79004467 | 1.00E-08 | 0.3584172 | 4 | 4 | LOC101055644 | 939.75 | 4 | 0.55248883 | 18 | 0.99973376 |
| 529 | 2.79004467 | 1.00E-08 | 0.3584172 | 4 | 4 | LOC100862570 | 939.75 | 4 | 0.55248883 | 18 | 0.99973376 |
| 530 | 2.79004467 | 1.00E-08 | 0.3584172 | 4 | 4 | LOC100047632 | 939.75 | 4 | 0.55248883 | 18 | 0.99973376 |
| 531 | 2.79004467 | 1.00E-08 | 0.3584172 | 4 | 4 | LOC100046684 | 939.75 | 4 | 0.55248883 | 18 | 0.99973376 |
| 532 | 2.38289726 | 2.86E-05 | 0.4196572 | 4 | 4 | Lnp | 678.5 | 4 | 0.65427569 | 288318 | 0.53855326 |
| 533 | 2.79004467 | 1.00E-08 | 0.3584172 | 4 | 4 | Lin54 | 939.75 | 4 | 0.55248883 | 18 | 0.99973376 |
| 534 | 2.3586471 | 2.87E-05 | 0.42397186 | 4 | 4 | Lin28a | 717.5 | 4 | 0.66033823 | 293386 | 0.56108066 |
| 535 | 2.37141034 | 2.95E-05 | 0.42168999 | 4 | 4 | Limk2 | 668.5 | 4 | 0.65714742 | 301340 | 0.52683504 |
| 536 | 2.50031908 | 2.43E-05 | 0.39994895 | 4 | 4 | Lhx3 | 513 | 4 | 0.62492023 | 189514 | 0.43910806 |
| 537 | 2.44160817 | 2.56E-05 | 0.40956613 | 4 | 4 | Lhfpl4 | 566.75 | 4 | 0.63959796 | 197036 | 0.46679043 |
| 538 | 2.79004467 | 1.00E-08 | 0.3584172 | 4 | 4 | Lgals9 | 939.75 | 4 | 0.55248883 | 18 | 0.99973376 |
| 539 | 2.79004467 | 1.00E-08 | 0.3584172 | 4 | 4 | Lce3e | 939.75 | 4 | 0.55248883 | 18 | 0.99973376 |
| 540 | 2.79004467 | 1.00E-08 | 0.3584172 | 4 | 4 | Lamp2 | 939.75 | 4 | 0.55248883 | 18 | 0.99973376 |
| 541 | 2.79004467 | 1.00E-08 | 0.3584172 | 4 | 4 | Lad1 | 939.75 | 4 | 0.55248883 | 18 | 0.99973376 |
| 542 | 2.38289726 | 2.86E-05 | 0.4196572 | 4 | 4 | L3mbtl4 | 678.5 | 4 | 0.65427569 | 288318 | 0.53855326 |
| 543 | 2.79004467 | 1.00E-08 | 0.3584172 | 4 | 4 | L2hgdh | 939.75 | 4 | 0.55248883 | 18 | 0.99973376 |
| 544 | 2.79004467 | 1.00E-08 | 0.3584172 | 4 | 4 | Krtap5-3 | 939.75 | 4 | 0.55248883 | 18 | 0.99973376 |
| 545 | 2.79004467 | 1.00E-08 | 0.3584172 | 4 | 4 | Krt8 | 939.75 | 4 | 0.55248883 | 18 | 0.99973376 |
| 546 | 2.3586471 | 3.03E-05 | 0.42397186 | 4 | 4 | Kpna6 | 703.75 | 4 | 0.66033823 | 306586 | 0.55031323 |
| 547 | 2.53095086 | 2.33E-05 | 0.39510842 | 4 | 4 | Klhl29 | 477.75 | 4 | 0.61726228 | 183940 | 0.41746935 |
| 548 | 2.3586471 | 2.87E-05 | 0.42397186 | 4 | 4 | Klf13 | 717.5 | 4 | 0.66033823 | 293386 | 0.56108066 |
| 549 | 2.4275686 | 2.84E-05 | 0.41193481 | 4 | 4 | Kif5a | 624.75 | 4 | 0.64310785 | 286214 | 0.51001635 |
| 550 | 2.3586471 | 3.03E-05 | 0.42397186 | 4 | 4 | Kif1b | 703.75 | 4 | 0.66033823 | 306586 | 0.55031323 |
| 551 | 2.3586471 | 3.03E-05 | 0.42397186 | 4 | 4 | Kif14 | 703.75 | 4 | 0.66033823 | 306586 | 0.55031323 |
| 552 | 2.3586471 | 2.87E-05 | 0.42397186 | 4 | 4 | Kdsr | 717.5 | 4 | 0.66033823 | 293386 | 0.56108066 |
| 553 | 2.79004467 | 1.00E-08 | 0.3584172 | 4 | 4 | Kcnj4 | 939.75 | 4 | 0.55248883 | 18 | 0.99973376 |
| 554 | 2.79004467 | 1.00E-08 | 0.3584172 | 4 | 4 | Kcnip4 | 939.75 | 4 | 0.55248883 | 18 | 0.99973376 |
| 555 | 2.53733248 | 2.34E-05 | 0.39411469 | 4 | 4 | Kcnab2 | 487.75 | 4 | 0.61566688 | 180098 | 0.42810026 |
| 556 | 2.4275686 | 2.84E-05 | 0.41193481 | 4 | 4 | Kbtbd11 | 624.75 | 4 | 0.64310785 | 286214 | 0.51001635 |
| 557 | 2.79004467 | 1.00E-08 | 0.3584172 | 4 | 4 | Katnal2 | 939.75 | 4 | 0.55248883 | 18 | 0.99973376 |
| 558 | 2.3586471 | 2.87E-05 | 0.42397186 | 4 | 4 | Jmy | 717.5 | 4 | 0.66033823 | 293386 | 0.56108066 |
| 559 | 2.79004467 | 1.00E-08 | 0.3584172 | 4 | 4 | Jmjd8 | 939.75 | 4 | 0.55248883 | 18 | 0.99973376 |
| 560 | 2.46330568 | 2.45E-05 | 0.40595855 | 4 | 4 | Iws1 | 531.5 | 4 | 0.63417358 | 189978 | 0.44393305 |
| 561 | 2.79004467 | 1.00E-08 | 0.3584172 | 4 | 4 | Ivd | 939.75 | 4 | 0.55248883 | 18 | 0.99973376 |
| 562 | 2.4275686 | 2.84E-05 | 0.41193481 | 4 | 4 | Itpripl2 | 624.75 | 4 | 0.64310785 | 286214 | 0.51001635 |
| 563 | 2.3586471 | 3.03E-05 | 0.42397186 | 4 | 4 | Itpr2 | 703.75 | 4 | 0.66033823 | 306586 | 0.55031323 |
| 564 | 2.79004467 | 1.00E-08 | 0.3584172 | 4 | 4 | Itpr1 | 939.75 | 4 | 0.55248883 | 18 | 0.99973376 |
| 565 | 2.79004467 | 1.00E-08 | 0.3584172 | 4 | 4 | Itm2c | 939.75 | 4 | 0.55248883 | 18 | 0.99973376 |
| 566 | 2.37141034 | 2.95E-05 | 0.42168999 | 4 | 4 | Itih5 | 668.5 | 4 | 0.65714742 | 301340 | 0.52683504 |
| 567 | 2.79004467 | 1.00E-08 | 0.3584172 | 4 | 4 | Itgb8 | 939.75 | 4 | 0.55248883 | 18 | 0.99973376 |
| 568 | 2.79004467 | 1.00E-08 | 0.3584172 | 4 | 4 | Itgax | 939.75 | 4 | 0.55248883 | 18 | 0.99973376 |
| 569 | 2.79004467 | 1.00E-08 | 0.3584172 | 4 | 4 | Itga8 | 939.75 | 4 | 0.55248883 | 18 | 0.99973376 |
| 570 | 2.79004467 | 1.00E-08 | 0.3584172 | 4 | 4 | Itga10 | 939.75 | 4 | 0.55248883 | 18 | 0.99973376 |
| 571 | 2.79004467 | 1.00E-08 | 0.3584172 | 4 | 4 | Isyna1 | 939.75 | 4 | 0.55248883 | 18 | 0.99973376 |
| 572 | 2.3586471 | 2.87E-05 | 0.42397186 | 4 | 4 | Irf2bp2 | 717.5 | 4 | 0.66033823 | 293386 | 0.56108066 |
| 573 | 2.79004467 | 1.00E-08 | 0.3584172 | 4 | 4 | Ipo8 | 939.75 | 4 | 0.55248883 | 18 | 0.99973376 |
| 574 | 2.42118698 | 2.60E-05 | 0.41302056 | 4 | 4 | Ipmk | 605.75 | 4 | 0.64470325 | 198922 | 0.49246743 |
| 575 | 2.79004467 | 1.00E-08 | 0.3584172 | 4 | 4 | Ints9 | 939.75 | 4 | 0.55248883 | 18 | 0.99973376 |
| 576 | 2.79004467 | 1.00E-08 | 0.3584172 | 4 | 4 | Inpp5b | 939.75 | 4 | 0.55248883 | 18 | 0.99973376 |
| 577 | 2.79004467 | 1.00E-08 | 0.3584172 | 4 | 4 | Ing3 | 939.75 | 4 | 0.55248883 | 18 | 0.99973376 |
| 578 | 2.3586471 | 2.87E-05 | 0.42397186 | 4 | 4 | Il1rl1 | 717.5 | 4 | 0.66033823 | 293386 | 0.56108066 |
| 579 | 2.79004467 | 1.00E-08 | 0.3584172 | 4 | 4 | Il17re | 939.75 | 4 | 0.55248883 | 18 | 0.99973376 |
| 580 | 2.3586471 | 2.87E-05 | 0.42397186 | 4 | 4 | Il17rd | 717.5 | 4 | 0.66033823 | 293386 | 0.56108066 |
| 581 | 2.38289726 | 2.86E-05 | 0.4196572 | 4 | 4 | Ikbkg | 678.5 | 4 | 0.65427569 | 288318 | 0.53855326 |
| 582 | 2.3586471 | 2.87E-05 | 0.42397186 | 4 | 4 | Igf1 | 717.5 | 4 | 0.66033823 | 293386 | 0.56108066 |
| 583 | 2.79004467 | 1.00E-08 | 0.3584172 | 4 | 4 | Ier3ip1 | 939.75 | 4 | 0.55248883 | 18 | 0.99973376 |
| 584 | 2.3586471 | 2.87E-05 | 0.42397186 | 4 | 4 | Idua | 717.5 | 4 | 0.66033823 | 293386 | 0.56108066 |
| 585 | 2.3586471 | 3.03E-05 | 0.42397186 | 4 | 4 | Ide | 703.75 | 4 | 0.66033823 | 306586 | 0.55031323 |
| 586 | 2.79004467 | 1.00E-08 | 0.3584172 | 4 | 4 | Hsd11b2 | 939.75 | 4 | 0.55248883 | 18 | 0.99973376 |
| 587 | 2.79004467 | 1.00E-08 | 0.3584172 | 4 | 4 | Hs6st1 | 939.75 | 4 | 0.55248883 | 18 | 0.99973376 |
| 588 | 2.3586471 | 3.03E-05 | 0.42397186 | 4 | 4 | Hpcal4 | 703.75 | 4 | 0.66033823 | 306586 | 0.55031323 |
| 589 | 2.3586471 | 3.03E-05 | 0.42397186 | 4 | 4 | Hoxc5 | 703.75 | 4 | 0.66033823 | 306586 | 0.55031323 |
| 590 | 2.3586471 | 2.87E-05 | 0.42397186 | 4 | 4 | Hook3 | 717.5 | 4 | 0.66033823 | 293386 | 0.56108066 |
| 591 | 2.3586471 | 3.03E-05 | 0.42397186 | 4 | 4 | Hnf4a | 703.75 | 4 | 0.66033823 | 306586 | 0.55031323 |
| 592 | 2.79004467 | 1.00E-08 | 0.3584172 | 4 | 4 | Hn1l | 939.75 | 4 | 0.55248883 | 18 | 0.99973376 |
| 593 | 2.79004467 | 1.00E-08 | 0.3584172 | 4 | 4 | Hmga1-rs1 | 939.75 | 4 | 0.55248883 | 18 | 0.99973376 |
| 594 | 2.79004467 | 1.00E-08 | 0.3584172 | 4 | 4 | Hmga1 | 939.75 | 4 | 0.55248883 | 18 | 0.99973376 |
| 595 | 2.3586471 | 2.87E-05 | 0.42397186 | 4 | 4 | Hist1h2ac | 717.5 | 4 | 0.66033823 | 293386 | 0.56108066 |
| 596 | 2.79004467 | 1.00E-08 | 0.3584172 | 4 | 4 | Hhla1 | 939.75 | 4 | 0.55248883 | 18 | 0.99973376 |
| 597 | 2.37141034 | 2.95E-05 | 0.42168999 | 4 | 4 | Heyl | 668.5 | 4 | 0.65714742 | 301340 | 0.52683504 |
| 598 | 2.79004467 | 1.00E-08 | 0.3584172 | 4 | 4 | Hes5 | 939.75 | 4 | 0.55248883 | 18 | 0.99973376 |
| 599 | 2.79004467 | 1.00E-08 | 0.3584172 | 4 | 4 | Henmt1 | 939.75 | 4 | 0.55248883 | 18 | 0.99973376 |
| 600 | 2.79004467 | 1.00E-08 | 0.3584172 | 4 | 4 | Helz | 939.75 | 4 | 0.55248883 | 18 | 0.99973376 |
| 601 | 2.79004467 | 1.00E-08 | 0.3584172 | 4 | 4 | Hcn2 | 939.75 | 4 | 0.55248883 | 18 | 0.99973376 |
| 602 | 2.79004467 | 1.00E-08 | 0.3584172 | 4 | 4 | Hap1 | 939.75 | 4 | 0.55248883 | 18 | 0.99973376 |
| 603 | 2.79004467 | 1.00E-08 | 0.3584172 | 4 | 4 | Haao | 939.75 | 4 | 0.55248883 | 18 | 0.99973376 |
| 604 | 2.79004467 | 1.00E-08 | 0.3584172 | 4 | 4 | H60a | 939.75 | 4 | 0.55248883 | 18 | 0.99973376 |
| 605 | 2.79004467 | 1.00E-08 | 0.3584172 | 4 | 4 | H2-M10.6 | 939.75 | 4 | 0.55248883 | 18 | 0.99973376 |
| 606 | 2.79004467 | 1.00E-08 | 0.3584172 | 4 | 4 | Guca2a | 939.75 | 4 | 0.55248883 | 18 | 0.99973376 |
| 607 | 2.79004467 | 1.00E-08 | 0.3584172 | 4 | 4 | Gstm1 | 939.75 | 4 | 0.55248883 | 18 | 0.99973376 |
| 608 | 2.79004467 | 1.00E-08 | 0.3584172 | 4 | 4 | Gsdmcl1 | 939.75 | 4 | 0.55248883 | 18 | 0.99973376 |
| 609 | 2.3586471 | 2.87E-05 | 0.42397186 | 4 | 4 | Grk1 | 717.5 | 4 | 0.66033823 | 293386 | 0.56108066 |
| 610 | 2.3586471 | 3.03E-05 | 0.42397186 | 4 | 4 | Gria2 | 703.75 | 4 | 0.66033823 | 306586 | 0.55031323 |
| 611 | 2.43650287 | 2.54E-05 | 0.41042431 | 4 | 4 | Grhl2 | 570.5 | 4 | 0.64087428 | 198680 | 0.46833882 |
| 612 | 2.38289726 | 2.86E-05 | 0.4196572 | 4 | 4 | Gramd4 | 678.5 | 4 | 0.65427569 | 288318 | 0.53855326 |
| 613 | 2.79004467 | 1.00E-08 | 0.3584172 | 4 | 4 | Gpx2 | 939.75 | 4 | 0.55248883 | 18 | 0.99973376 |
| 614 | 2.42501595 | 2.61E-05 | 0.41236842 | 4 | 4 | Gprc5b | 556.75 | 4 | 0.64374601 | 206794 | 0.45367347 |
| 615 | 2.79004467 | 1.00E-08 | 0.3584172 | 4 | 4 | Gpr83 | 939.75 | 4 | 0.55248883 | 18 | 0.99973376 |
| 616 | 2.79004467 | 1.00E-08 | 0.3584172 | 4 | 4 | Gpr45 | 939.75 | 4 | 0.55248883 | 18 | 0.99973376 |
| 617 | 2.79004467 | 1.00E-08 | 0.3584172 | 4 | 4 | Gpr25 | 939.75 | 4 | 0.55248883 | 18 | 0.99973376 |
| 618 | 2.79004467 | 1.00E-08 | 0.3584172 | 4 | 4 | Gpr111 | 939.75 | 4 | 0.55248883 | 18 | 0.99973376 |
| 619 | 2.79004467 | 1.00E-08 | 0.3584172 | 4 | 4 | Gpkow | 939.75 | 4 | 0.55248883 | 18 | 0.99973376 |
| 620 | 2.79004467 | 1.00E-08 | 0.3584172 | 4 | 4 | Gpd1 | 939.75 | 4 | 0.55248883 | 18 | 0.99973376 |
| 621 | 2.79004467 | 1.00E-08 | 0.3584172 | 4 | 4 | Gpbp1l1 | 939.75 | 4 | 0.55248883 | 18 | 0.99973376 |
| 622 | 2.4275686 | 2.84E-05 | 0.41193481 | 4 | 4 | Gpatch2l | 624.75 | 4 | 0.64310785 | 286214 | 0.51001635 |
| 623 | 2.3586471 | 2.87E-05 | 0.42397186 | 4 | 4 | Gpatch2 | 717.5 | 4 | 0.66033823 | 293386 | 0.56108066 |
| 624 | 2.79004467 | 1.00E-08 | 0.3584172 | 4 | 4 | Gp9 | 939.75 | 4 | 0.55248883 | 18 | 0.99973376 |
| 625 | 2.79004467 | 1.00E-08 | 0.3584172 | 4 | 4 | Gnb3 | 939.75 | 4 | 0.55248883 | 18 | 0.99973376 |
| 626 | 2.38289726 | 2.86E-05 | 0.4196572 | 4 | 4 | Gnas | 678.5 | 4 | 0.65427569 | 288318 | 0.53855326 |
| 627 | 2.79004467 | 1.00E-08 | 0.3584172 | 4 | 4 | Gm9229 | 939.75 | 4 | 0.55248883 | 18 | 0.99973376 |
| 628 | 2.3586471 | 2.87E-05 | 0.42397186 | 4 | 4 | Gm7694 | 717.5 | 4 | 0.66033823 | 293386 | 0.56108066 |
| 629 | 2.79004467 | 1.00E-08 | 0.3584172 | 4 | 4 | Gm6588 | 939.75 | 4 | 0.55248883 | 18 | 0.99973376 |
| 630 | 2.79004467 | 1.00E-08 | 0.3584172 | 4 | 4 | Gm6583 | 939.75 | 4 | 0.55248883 | 18 | 0.99973376 |
| 631 | 2.79004467 | 1.00E-08 | 0.3584172 | 4 | 4 | Gm6086 | 939.75 | 4 | 0.55248883 | 18 | 0.99973376 |
| 632 | 2.79004467 | 1.00E-08 | 0.3584172 | 4 | 4 | Gm20149 | 939.75 | 4 | 0.55248883 | 18 | 0.99973376 |
| 633 | 2.79004467 | 1.00E-08 | 0.3584172 | 4 | 4 | Gm20033 | 939.75 | 4 | 0.55248883 | 18 | 0.99973376 |
| 634 | 2.79004467 | 1.00E-08 | 0.3584172 | 4 | 4 | Gm15688 | 939.75 | 4 | 0.55248883 | 18 | 0.99973376 |
| 635 | 2.79004467 | 1.00E-08 | 0.3584172 | 4 | 4 | Gm14085 | 939.75 | 4 | 0.55248883 | 18 | 0.99973376 |
| 636 | 2.79004467 | 1.00E-08 | 0.3584172 | 4 | 4 | Gm13288 | 939.75 | 4 | 0.55248883 | 18 | 0.99973376 |
| 637 | 2.79004467 | 1.00E-08 | 0.3584172 | 4 | 4 | Gm13123 | 939.75 | 4 | 0.55248883 | 18 | 0.99973376 |
| 638 | 2.79004467 | 1.00E-08 | 0.3584172 | 4 | 4 | Gm12794 | 939.75 | 4 | 0.55248883 | 18 | 0.99973376 |
| 639 | 2.4275686 | 2.84E-05 | 0.41193481 | 4 | 4 | Gm1078 | 624.75 | 4 | 0.64310785 | 286214 | 0.51001635 |
| 640 | 2.79004467 | 1.00E-08 | 0.3584172 | 4 | 4 | Glud1 | 939.75 | 4 | 0.55248883 | 18 | 0.99973376 |
| 641 | 2.79004467 | 1.00E-08 | 0.3584172 | 4 | 4 | Gli3 | 939.75 | 4 | 0.55248883 | 18 | 0.99973376 |
| 642 | 2.79004467 | 1.00E-08 | 0.3584172 | 4 | 4 | Gimap3 | 939.75 | 4 | 0.55248883 | 18 | 0.99973376 |
| 643 | 2.3586471 | 2.87E-05 | 0.42397186 | 4 | 4 | Gga2 | 717.5 | 4 | 0.66033823 | 293386 | 0.56108066 |
| 644 | 2.79004467 | 1.00E-08 | 0.3584172 | 4 | 4 | Gfod2 | 939.75 | 4 | 0.55248883 | 18 | 0.99973376 |
| 645 | 2.37141034 | 2.95E-05 | 0.42168999 | 4 | 4 | Gfod1 | 668.5 | 4 | 0.65714742 | 301340 | 0.52683504 |
| 646 | 2.3586471 | 2.87E-05 | 0.42397186 | 4 | 4 | Gdap1l1 | 717.5 | 4 | 0.66033823 | 293386 | 0.56108066 |
| 647 | 2.79004467 | 1.00E-08 | 0.3584172 | 4 | 4 | Gcsam | 939.75 | 4 | 0.55248883 | 18 | 0.99973376 |
| 648 | 2.79004467 | 1.00E-08 | 0.3584172 | 4 | 4 | Gcn1l1 | 939.75 | 4 | 0.55248883 | 18 | 0.99973376 |
| 649 | 2.79004467 | 1.00E-08 | 0.3584172 | 4 | 4 | Gcdh | 939.75 | 4 | 0.55248883 | 18 | 0.99973376 |
| 650 | 2.79004467 | 1.00E-08 | 0.3584172 | 4 | 4 | Gbp7 | 939.75 | 4 | 0.55248883 | 18 | 0.99973376 |
| 651 | 2.79004467 | 1.00E-08 | 0.3584172 | 4 | 4 | Gbp11 | 939.75 | 4 | 0.55248883 | 18 | 0.99973376 |
| 652 | 2.38289726 | 2.86E-05 | 0.4196572 | 4 | 4 | Gata2 | 678.5 | 4 | 0.65427569 | 288318 | 0.53855326 |
| 653 | 2.79004467 | 1.00E-08 | 0.3584172 | 4 | 4 | Galnt16 | 939.75 | 4 | 0.55248883 | 18 | 0.99973376 |
| 654 | 2.79004467 | 1.00E-08 | 0.3584172 | 4 | 4 | Gal3st2 | 939.75 | 4 | 0.55248883 | 18 | 0.99973376 |
| 655 | 2.79004467 | 1.00E-08 | 0.3584172 | 4 | 4 | Gabra3 | 939.75 | 4 | 0.55248883 | 18 | 0.99973376 |
| 656 | 2.79004467 | 1.00E-08 | 0.3584172 | 4 | 4 | Fzd4 | 939.75 | 4 | 0.55248883 | 18 | 0.99973376 |
| 657 | 2.79004467 | 1.00E-08 | 0.3584172 | 4 | 4 | Fxyd1 | 939.75 | 4 | 0.55248883 | 18 | 0.99973376 |
| 658 | 2.79004467 | 1.00E-08 | 0.3584172 | 4 | 4 | Fsd1 | 939.75 | 4 | 0.55248883 | 18 | 0.99973376 |
| 659 | 2.3586471 | 2.87E-05 | 0.42397186 | 4 | 4 | Frmd8 | 717.5 | 4 | 0.66033823 | 293386 | 0.56108066 |
| 660 | 2.3586471 | 2.87E-05 | 0.42397186 | 4 | 4 | Foxk2 | 717.5 | 4 | 0.66033823 | 293386 | 0.56108066 |
| 661 | 2.46330568 | 2.45E-05 | 0.40595855 | 4 | 4 | Fndc9 | 531.5 | 4 | 0.63417358 | 189978 | 0.44393305 |
| 662 | 2.79004467 | 1.00E-08 | 0.3584172 | 4 | 4 | Fgl2 | 939.75 | 4 | 0.55248883 | 18 | 0.99973376 |
| 663 | 2.79004467 | 1.00E-08 | 0.3584172 | 4 | 4 | Fgfr3 | 939.75 | 4 | 0.55248883 | 18 | 0.99973376 |
| 664 | 2.79004467 | 1.00E-08 | 0.3584172 | 4 | 4 | Fgf9 | 939.75 | 4 | 0.55248883 | 18 | 0.99973376 |
| 665 | 2.79004467 | 1.00E-08 | 0.3584172 | 4 | 4 | Fgd4 | 939.75 | 4 | 0.55248883 | 18 | 0.99973376 |
| 666 | 2.3586471 | 2.87E-05 | 0.42397186 | 4 | 4 | Fbxw8 | 717.5 | 4 | 0.66033823 | 293386 | 0.56108066 |
| 667 | 2.3586471 | 3.03E-05 | 0.42397186 | 4 | 4 | Fat3 | 703.75 | 4 | 0.66033823 | 306586 | 0.55031323 |
| 668 | 2.79004467 | 1.00E-08 | 0.3584172 | 4 | 4 | Fas | 939.75 | 4 | 0.55248883 | 18 | 0.99973376 |
| 669 | 2.79004467 | 1.00E-08 | 0.3584172 | 4 | 4 | Farsb | 939.75 | 4 | 0.55248883 | 18 | 0.99973376 |
| 670 | 2.79004467 | 1.00E-08 | 0.3584172 | 4 | 4 | Fam78a | 939.75 | 4 | 0.55248883 | 18 | 0.99973376 |
| 671 | 2.79004467 | 1.00E-08 | 0.3584172 | 4 | 4 | Fam76b | 939.75 | 4 | 0.55248883 | 18 | 0.99973376 |
| 672 | 2.38289726 | 2.86E-05 | 0.4196572 | 4 | 4 | Fam210b | 678.5 | 4 | 0.65427569 | 288318 | 0.53855326 |
| 673 | 2.3586471 | 2.87E-05 | 0.42397186 | 4 | 4 | Fam210a | 717.5 | 4 | 0.66033823 | 293386 | 0.56108066 |
| 674 | 2.42118698 | 2.60E-05 | 0.41302056 | 4 | 4 | Fam20b | 605.75 | 4 | 0.64470325 | 198922 | 0.49246743 |
| 675 | 2.79004467 | 1.00E-08 | 0.3584172 | 4 | 4 | Fam189a1 | 939.75 | 4 | 0.55248883 | 18 | 0.99973376 |
| 676 | 2.3586471 | 2.87E-05 | 0.42397186 | 4 | 4 | Fam188b | 717.5 | 4 | 0.66033823 | 293386 | 0.56108066 |
| 677 | 2.79004467 | 1.00E-08 | 0.3584172 | 4 | 4 | Fam181a | 939.75 | 4 | 0.55248883 | 18 | 0.99973376 |
| 678 | 2.79004467 | 1.00E-08 | 0.3584172 | 4 | 4 | Fam178a | 939.75 | 4 | 0.55248883 | 18 | 0.99973376 |
| 679 | 2.79004467 | 1.00E-08 | 0.3584172 | 4 | 4 | Fam170b | 939.75 | 4 | 0.55248883 | 18 | 0.99973376 |
| 680 | 2.4275686 | 2.84E-05 | 0.41193481 | 4 | 4 | Fam168a | 624.75 | 4 | 0.64310785 | 286214 | 0.51001635 |
| 681 | 2.3586471 | 2.87E-05 | 0.42397186 | 4 | 4 | Fam149a | 717.5 | 4 | 0.66033823 | 293386 | 0.56108066 |
| 682 | 2.79004467 | 1.00E-08 | 0.3584172 | 4 | 4 | Fam124b | 939.75 | 4 | 0.55248883 | 18 | 0.99973376 |
| 683 | 2.79004467 | 1.00E-08 | 0.3584172 | 4 | 4 | Fabp12 | 939.75 | 4 | 0.55248883 | 18 | 0.99973376 |
| 684 | 2.79004467 | 1.00E-08 | 0.3584172 | 4 | 4 | Fa2h | 939.75 | 4 | 0.55248883 | 18 | 0.99973376 |
| 685 | 2.3586471 | 3.03E-05 | 0.42397186 | 4 | 4 | Eya3 | 703.75 | 4 | 0.66033823 | 306586 | 0.55031323 |
| 686 | 2.38289726 | 2.86E-05 | 0.4196572 | 4 | 4 | Ets1 | 678.5 | 4 | 0.65427569 | 288318 | 0.53855326 |
| 687 | 2.79004467 | 1.00E-08 | 0.3584172 | 4 | 4 | Epm2a | 939.75 | 4 | 0.55248883 | 18 | 0.99973376 |
| 688 | 2.3586471 | 3.03E-05 | 0.42397186 | 4 | 4 | Endod1 | 703.75 | 4 | 0.66033823 | 306586 | 0.55031323 |
| 689 | 2.79004467 | 1.00E-08 | 0.3584172 | 4 | 4 | Elof1 | 939.75 | 4 | 0.55248883 | 18 | 0.99973376 |
| 690 | 2.38289726 | 2.86E-05 | 0.4196572 | 4 | 4 | Eif4e2 | 678.5 | 4 | 0.65427569 | 288318 | 0.53855326 |
| 691 | 2.79004467 | 1.00E-08 | 0.3584172 | 4 | 4 | Efcab14 | 939.75 | 4 | 0.55248883 | 18 | 0.99973376 |
| 692 | 2.79004467 | 1.00E-08 | 0.3584172 | 4 | 4 | Edn2 | 939.75 | 4 | 0.55248883 | 18 | 0.99973376 |
| 693 | 2.3586471 | 3.03E-05 | 0.42397186 | 4 | 4 | Edem1 | 703.75 | 4 | 0.66033823 | 306586 | 0.55031323 |
| 694 | 2.79004467 | 1.00E-08 | 0.3584172 | 4 | 4 | E430018J23Rik | 939.75 | 4 | 0.55248883 | 18 | 0.99973376 |
| 695 | 2.79004467 | 1.00E-08 | 0.3584172 | 4 | 4 | Dynlrb1 | 939.75 | 4 | 0.55248883 | 18 | 0.99973376 |
| 696 | 2.4275686 | 2.84E-05 | 0.41193481 | 4 | 4 | Dusp18 | 624.75 | 4 | 0.64310785 | 286214 | 0.51001635 |
| 697 | 2.79004467 | 1.00E-08 | 0.3584172 | 4 | 4 | Duox1 | 939.75 | 4 | 0.55248883 | 18 | 0.99973376 |
| 698 | 2.79004467 | 1.00E-08 | 0.3584172 | 4 | 4 | Drg1 | 939.75 | 4 | 0.55248883 | 18 | 0.99973376 |
| 699 | 2.79004467 | 1.00E-08 | 0.3584172 | 4 | 4 | Dpy19l2 | 939.75 | 4 | 0.55248883 | 18 | 0.99973376 |
| 700 | 2.79004467 | 1.00E-08 | 0.3584172 | 4 | 4 | Dpcr1 | 939.75 | 4 | 0.55248883 | 18 | 0.99973376 |
| 701 | 2.38289726 | 2.86E-05 | 0.4196572 | 4 | 4 | Dolpp1 | 678.5 | 4 | 0.65427569 | 288318 | 0.53855326 |
| 702 | 2.3586471 | 2.87E-05 | 0.42397186 | 4 | 4 | Dnajc5 | 717.5 | 4 | 0.66033823 | 293386 | 0.56108066 |
| 703 | 2.3586471 | 2.87E-05 | 0.42397186 | 4 | 4 | Dnajc27 | 717.5 | 4 | 0.66033823 | 293386 | 0.56108066 |
| 704 | 2.43650287 | 2.54E-05 | 0.41042431 | 4 | 4 | Dnajc18 | 570.5 | 4 | 0.64087428 | 198680 | 0.46833882 |
| 705 | 2.3586471 | 3.03E-05 | 0.42397186 | 4 | 4 | Dnaja1 | 703.75 | 4 | 0.66033823 | 306586 | 0.55031323 |
| 706 | 2.79004467 | 1.00E-08 | 0.3584172 | 4 | 4 | Dnaaf3 | 939.75 | 4 | 0.55248883 | 18 | 0.99973376 |
| 707 | 2.79004467 | 1.00E-08 | 0.3584172 | 4 | 4 | Dmpk | 939.75 | 4 | 0.55248883 | 18 | 0.99973376 |
| 708 | 2.79004467 | 1.00E-08 | 0.3584172 | 4 | 4 | Dlx4 | 939.75 | 4 | 0.55248883 | 18 | 0.99973376 |
| 709 | 2.79004467 | 1.00E-08 | 0.3584172 | 4 | 4 | Dlx1 | 939.75 | 4 | 0.55248883 | 18 | 0.99973376 |
| 710 | 2.79004467 | 1.00E-08 | 0.3584172 | 4 | 4 | Dlg2 | 939.75 | 4 | 0.55248883 | 18 | 0.99973376 |
| 711 | 2.46330568 | 2.45E-05 | 0.40595855 | 4 | 4 | Dip2c | 531.5 | 4 | 0.63417358 | 189978 | 0.44393305 |
| 712 | 2.79004467 | 1.00E-08 | 0.3584172 | 4 | 4 | Dhx8 | 939.75 | 4 | 0.55248883 | 18 | 0.99973376 |
| 713 | 2.79004467 | 1.00E-08 | 0.3584172 | 4 | 4 | Dhx40 | 939.75 | 4 | 0.55248883 | 18 | 0.99973376 |
| 714 | 2.79004467 | 1.00E-08 | 0.3584172 | 4 | 4 | Dhrs13 | 939.75 | 4 | 0.55248883 | 18 | 0.99973376 |
| 715 | 2.37141034 | 2.95E-05 | 0.42168999 | 4 | 4 | Dgcr2 | 668.5 | 4 | 0.65714742 | 301340 | 0.52683504 |
| 716 | 2.79004467 | 1.00E-08 | 0.3584172 | 4 | 4 | Derl3 | 939.75 | 4 | 0.55248883 | 18 | 0.99973376 |
| 717 | 2.3586471 | 2.87E-05 | 0.42397186 | 4 | 4 | Dennd1b | 717.5 | 4 | 0.66033823 | 293386 | 0.56108066 |
| 718 | 2.79004467 | 1.00E-08 | 0.3584172 | 4 | 4 | Decr1 | 939.75 | 4 | 0.55248883 | 18 | 0.99973376 |
| 719 | 2.79004467 | 1.00E-08 | 0.3584172 | 4 | 4 | Ddo | 939.75 | 4 | 0.55248883 | 18 | 0.99973376 |
| 720 | 2.79004467 | 1.00E-08 | 0.3584172 | 4 | 4 | Ddi2 | 939.75 | 4 | 0.55248883 | 18 | 0.99973376 |
| 721 | 2.79004467 | 1.00E-08 | 0.3584172 | 4 | 4 | Dcun1d3 | 939.75 | 4 | 0.55248883 | 18 | 0.99973376 |
| 722 | 2.38289726 | 2.86E-05 | 0.4196572 | 4 | 4 | Dctn4 | 678.5 | 4 | 0.65427569 | 288318 | 0.53855326 |
| 723 | 2.3586471 | 2.87E-05 | 0.42397186 | 4 | 4 | Dcbld2 | 717.5 | 4 | 0.66033823 | 293386 | 0.56108066 |
| 724 | 2.3586471 | 2.87E-05 | 0.42397186 | 4 | 4 | Dcaf17 | 717.5 | 4 | 0.66033823 | 293386 | 0.56108066 |
| 725 | 2.79004467 | 1.00E-08 | 0.3584172 | 4 | 4 | Dbx2 | 939.75 | 4 | 0.55248883 | 18 | 0.99973376 |
| 726 | 2.3586471 | 3.03E-05 | 0.42397186 | 4 | 4 | Daam1 | 703.75 | 4 | 0.66033823 | 306586 | 0.55031323 |
| 727 | 2.79004467 | 1.00E-08 | 0.3584172 | 4 | 4 | D1Ertd622e | 939.75 | 4 | 0.55248883 | 18 | 0.99973376 |
| 728 | 2.79004467 | 1.00E-08 | 0.3584172 | 4 | 4 | D10Jhu81e | 939.75 | 4 | 0.55248883 | 18 | 0.99973376 |
| 729 | 2.3586471 | 3.03E-05 | 0.42397186 | 4 | 4 | Cyth2 | 703.75 | 4 | 0.66033823 | 306586 | 0.55031323 |
| 730 | 2.79004467 | 1.00E-08 | 0.3584172 | 4 | 4 | Cyp4v3 | 939.75 | 4 | 0.55248883 | 18 | 0.99973376 |
| 731 | 2.79004467 | 1.00E-08 | 0.3584172 | 4 | 4 | Cyp2c65 | 939.75 | 4 | 0.55248883 | 18 | 0.99973376 |
| 732 | 2.79004467 | 1.00E-08 | 0.3584172 | 4 | 4 | Cyc1 | 939.75 | 4 | 0.55248883 | 18 | 0.99973376 |
| 733 | 2.79004467 | 1.00E-08 | 0.3584172 | 4 | 4 | Cyb5r3 | 939.75 | 4 | 0.55248883 | 18 | 0.99973376 |
| 734 | 2.79004467 | 1.00E-08 | 0.3584172 | 4 | 4 | Cxcl16 | 939.75 | 4 | 0.55248883 | 18 | 0.99973376 |
| 735 | 2.79004467 | 1.00E-08 | 0.3584172 | 4 | 4 | Cx3cr1 | 939.75 | 4 | 0.55248883 | 18 | 0.99973376 |
| 736 | 2.79004467 | 1.00E-08 | 0.3584172 | 4 | 4 | Ctsw | 939.75 | 4 | 0.55248883 | 18 | 0.99973376 |
| 737 | 2.3586471 | 2.87E-05 | 0.42397186 | 4 | 4 | Ctdspl | 717.5 | 4 | 0.66033823 | 293386 | 0.56108066 |
| 738 | 2.37141034 | 2.95E-05 | 0.42168999 | 4 | 4 | Crtc3 | 668.5 | 4 | 0.65714742 | 301340 | 0.52683504 |
| 739 | 2.46330568 | 2.45E-05 | 0.40595855 | 4 | 4 | Crkl | 531.5 | 4 | 0.63417358 | 189978 | 0.44393305 |
| 740 | 2.79004467 | 1.00E-08 | 0.3584172 | 4 | 4 | Crip2 | 939.75 | 4 | 0.55248883 | 18 | 0.99973376 |
| 741 | 2.37141034 | 2.95E-05 | 0.42168999 | 4 | 4 | Crb2 | 668.5 | 4 | 0.65714742 | 301340 | 0.52683504 |
| 742 | 2.79004467 | 1.00E-08 | 0.3584172 | 4 | 4 | Cpped1 | 939.75 | 4 | 0.55248883 | 18 | 0.99973376 |
| 743 | 2.3586471 | 2.87E-05 | 0.42397186 | 4 | 4 | Cox10 | 717.5 | 4 | 0.66033823 | 293386 | 0.56108066 |
| 744 | 2.79004467 | 1.00E-08 | 0.3584172 | 4 | 4 | Copg2 | 939.75 | 4 | 0.55248883 | 18 | 0.99973376 |
| 745 | 2.38289726 | 2.86E-05 | 0.4196572 | 4 | 4 | Col5a1 | 678.5 | 4 | 0.65427569 | 288318 | 0.53855326 |
| 746 | 2.79004467 | 1.00E-08 | 0.3584172 | 4 | 4 | Cog2 | 939.75 | 4 | 0.55248883 | 18 | 0.99973376 |
| 747 | 2.3586471 | 2.87E-05 | 0.42397186 | 4 | 4 | Coa5 | 717.5 | 4 | 0.66033823 | 293386 | 0.56108066 |
| 748 | 2.79004467 | 1.00E-08 | 0.3584172 | 4 | 4 | Coa3 | 939.75 | 4 | 0.55248883 | 18 | 0.99973376 |
| 749 | 2.79004467 | 1.00E-08 | 0.3584172 | 4 | 4 | Cntd1 | 939.75 | 4 | 0.55248883 | 18 | 0.99973376 |
| 750 | 2.79004467 | 1.00E-08 | 0.3584172 | 4 | 4 | Cnksr2 | 939.75 | 4 | 0.55248883 | 18 | 0.99973376 |
| 751 | 2.3586471 | 2.87E-05 | 0.42397186 | 4 | 4 | Cnga3 | 717.5 | 4 | 0.66033823 | 293386 | 0.56108066 |
| 752 | 2.79004467 | 1.00E-08 | 0.3584172 | 4 | 4 | Clec5a | 939.75 | 4 | 0.55248883 | 18 | 0.99973376 |
| 753 | 2.79004467 | 1.00E-08 | 0.3584172 | 4 | 4 | Clec11a | 939.75 | 4 | 0.55248883 | 18 | 0.99973376 |
| 754 | 2.79004467 | 1.00E-08 | 0.3584172 | 4 | 4 | Clcnkb | 939.75 | 4 | 0.55248883 | 18 | 0.99973376 |
| 755 | 2.79004467 | 1.00E-08 | 0.3584172 | 4 | 4 | Chst4 | 939.75 | 4 | 0.55248883 | 18 | 0.99973376 |
| 756 | 2.3586471 | 2.87E-05 | 0.42397186 | 4 | 4 | Chrnb2 | 717.5 | 4 | 0.66033823 | 293386 | 0.56108066 |
| 757 | 2.79004467 | 1.00E-08 | 0.3584172 | 4 | 4 | Chrna3 | 939.75 | 4 | 0.55248883 | 18 | 0.99973376 |
| 758 | 2.3586471 | 2.87E-05 | 0.42397186 | 4 | 4 | Chic1 | 717.5 | 4 | 0.66033823 | 293386 | 0.56108066 |
| 759 | 2.79004467 | 1.00E-08 | 0.3584172 | 4 | 4 | Chga | 939.75 | 4 | 0.55248883 | 18 | 0.99973376 |
| 760 | 2.79004467 | 1.00E-08 | 0.3584172 | 4 | 4 | Cenpa | 939.75 | 4 | 0.55248883 | 18 | 0.99973376 |
| 761 | 2.3586471 | 2.87E-05 | 0.42397186 | 4 | 4 | Celf1 | 717.5 | 4 | 0.66033823 | 293386 | 0.56108066 |
| 762 | 2.79004467 | 1.00E-08 | 0.3584172 | 4 | 4 | Ceacam2 | 939.75 | 4 | 0.55248883 | 18 | 0.99973376 |
| 763 | 2.79004467 | 1.00E-08 | 0.3584172 | 4 | 4 | Ceacam1 | 939.75 | 4 | 0.55248883 | 18 | 0.99973376 |
| 764 | 2.79004467 | 1.00E-08 | 0.3584172 | 4 | 4 | Cdkn2a | 939.75 | 4 | 0.55248883 | 18 | 0.99973376 |
| 765 | 2.3586471 | 2.87E-05 | 0.42397186 | 4 | 4 | Cdh1 | 717.5 | 4 | 0.66033823 | 293386 | 0.56108066 |
| 766 | 2.79004467 | 1.00E-08 | 0.3584172 | 4 | 4 | Cd3d | 939.75 | 4 | 0.55248883 | 18 | 0.99973376 |
| 767 | 2.3586471 | 2.87E-05 | 0.42397186 | 4 | 4 | Cd38 | 717.5 | 4 | 0.66033823 | 293386 | 0.56108066 |
| 768 | 2.79004467 | 1.00E-08 | 0.3584172 | 4 | 4 | Cd300lg | 939.75 | 4 | 0.55248883 | 18 | 0.99973376 |
| 769 | 2.79004467 | 1.00E-08 | 0.3584172 | 4 | 4 | Cd300e | 939.75 | 4 | 0.55248883 | 18 | 0.99973376 |
| 770 | 2.44671347 | 2.48E-05 | 0.40871153 | 4 | 4 | Cd244 | 580.5 | 4 | 0.63832163 | 187712 | 0.47971854 |
| 771 | 2.3586471 | 3.03E-05 | 0.42397186 | 4 | 4 | Ccnd2 | 703.75 | 4 | 0.66033823 | 306586 | 0.55031323 |
| 772 | 2.79004467 | 1.00E-08 | 0.3584172 | 4 | 4 | Ccl4 | 939.75 | 4 | 0.55248883 | 18 | 0.99973376 |
| 773 | 2.3586471 | 2.87E-05 | 0.42397186 | 4 | 4 | Ccdc97 | 717.5 | 4 | 0.66033823 | 293386 | 0.56108066 |
| 774 | 2.3586471 | 3.03E-05 | 0.42397186 | 4 | 4 | Ccdc93 | 703.75 | 4 | 0.66033823 | 306586 | 0.55031323 |
| 775 | 2.37141034 | 2.95E-05 | 0.42168999 | 4 | 4 | Ccdc86 | 668.5 | 4 | 0.65714742 | 301340 | 0.52683504 |
| 776 | 2.79004467 | 1.00E-08 | 0.3584172 | 4 | 4 | Ccdc71l | 939.75 | 4 | 0.55248883 | 18 | 0.99973376 |
| 777 | 2.79004467 | 1.00E-08 | 0.3584172 | 4 | 4 | Ccdc58 | 939.75 | 4 | 0.55248883 | 18 | 0.99973376 |
| 778 | 2.79004467 | 1.00E-08 | 0.3584172 | 4 | 4 | Ccdc55 | 939.75 | 4 | 0.55248883 | 18 | 0.99973376 |
| 779 | 2.79004467 | 1.00E-08 | 0.3584172 | 4 | 4 | Ccdc132 | 939.75 | 4 | 0.55248883 | 18 | 0.99973376 |
| 780 | 2.37141034 | 2.95E-05 | 0.42168999 | 4 | 4 | Ccbe1 | 668.5 | 4 | 0.65714742 | 301340 | 0.52683504 |
| 781 | 2.3586471 | 2.87E-05 | 0.42397186 | 4 | 4 | Cbx5 | 717.5 | 4 | 0.66033823 | 293386 | 0.56108066 |
| 782 | 2.3586471 | 3.03E-05 | 0.42397186 | 4 | 4 | Cbfa2t2 | 703.75 | 4 | 0.66033823 | 306586 | 0.55031323 |
| 783 | 2.79004467 | 1.00E-08 | 0.3584172 | 4 | 4 | Casq2 | 939.75 | 4 | 0.55248883 | 18 | 0.99973376 |
| 784 | 2.79004467 | 1.00E-08 | 0.3584172 | 4 | 4 | Casp14 | 939.75 | 4 | 0.55248883 | 18 | 0.99973376 |
| 785 | 2.4275686 | 2.84E-05 | 0.41193481 | 4 | 4 | Card10 | 624.75 | 4 | 0.64310785 | 286214 | 0.51001635 |
| 786 | 2.3586471 | 2.87E-05 | 0.42397186 | 4 | 4 | Camk4 | 717.5 | 4 | 0.66033823 | 293386 | 0.56108066 |
| 787 | 2.79004467 | 1.00E-08 | 0.3584172 | 4 | 4 | Camk2b | 939.75 | 4 | 0.55248883 | 18 | 0.99973376 |
| 788 | 2.3586471 | 3.03E-05 | 0.42397186 | 4 | 4 | Camk1d | 703.75 | 4 | 0.66033823 | 306586 | 0.55031323 |
| 789 | 2.79004467 | 1.00E-08 | 0.3584172 | 4 | 4 | Calr | 939.75 | 4 | 0.55248883 | 18 | 0.99973376 |
| 790 | 2.38289726 | 2.86E-05 | 0.4196572 | 4 | 4 | Cacna2d4 | 678.5 | 4 | 0.65427569 | 288318 | 0.53855326 |
| 791 | 2.79004467 | 1.00E-08 | 0.3584172 | 4 | 4 | C87436 | 939.75 | 4 | 0.55248883 | 18 | 0.99973376 |
| 792 | 2.3586471 | 3.03E-05 | 0.42397186 | 4 | 4 | C230081A13Rik | 703.75 | 4 | 0.66033823 | 306586 | 0.55031323 |
| 793 | 2.37141034 | 2.95E-05 | 0.42168999 | 4 | 4 | C1qtnf1 | 668.5 | 4 | 0.65714742 | 301340 | 0.52683504 |
| 794 | 2.79004467 | 1.00E-08 | 0.3584172 | 4 | 4 | Bub3 | 939.75 | 4 | 0.55248883 | 18 | 0.99973376 |
| 795 | 2.3586471 | 3.03E-05 | 0.42397186 | 4 | 4 | Btrc | 703.75 | 4 | 0.66033823 | 306586 | 0.55031323 |
| 796 | 2.3586471 | 2.87E-05 | 0.42397186 | 4 | 4 | Bri3bp | 717.5 | 4 | 0.66033823 | 293386 | 0.56108066 |
| 797 | 2.3586471 | 3.03E-05 | 0.42397186 | 4 | 4 | Braf | 703.75 | 4 | 0.66033823 | 306586 | 0.55031323 |
| 798 | 2.79004467 | 1.00E-08 | 0.3584172 | 4 | 4 | Bpifb9b | 939.75 | 4 | 0.55248883 | 18 | 0.99973376 |
| 799 | 2.79004467 | 1.00E-08 | 0.3584172 | 4 | 4 | Bpifb9a | 939.75 | 4 | 0.55248883 | 18 | 0.99973376 |
| 800 | 2.3586471 | 3.03E-05 | 0.42397186 | 4 | 4 | Bmpr2 | 703.75 | 4 | 0.66033823 | 306586 | 0.55031323 |
| 801 | 2.79004467 | 1.00E-08 | 0.3584172 | 4 | 4 | Bmpr1a | 939.75 | 4 | 0.55248883 | 18 | 0.99973376 |
| 802 | 2.79004467 | 1.00E-08 | 0.3584172 | 4 | 4 | Bmp8a | 939.75 | 4 | 0.55248883 | 18 | 0.99973376 |
| 803 | 2.79004467 | 1.00E-08 | 0.3584172 | 4 | 4 | Bhlhe40 | 939.75 | 4 | 0.55248883 | 18 | 0.99973376 |
| 804 | 2.79004467 | 1.00E-08 | 0.3584172 | 4 | 4 | Becn1 | 939.75 | 4 | 0.55248883 | 18 | 0.99973376 |
| 805 | 2.79004467 | 1.00E-08 | 0.3584172 | 4 | 4 | Bcl3 | 939.75 | 4 | 0.55248883 | 18 | 0.99973376 |
| 806 | 2.79004467 | 1.00E-08 | 0.3584172 | 4 | 4 | BC049352 | 939.75 | 4 | 0.55248883 | 18 | 0.99973376 |
| 807 | 2.37141034 | 2.95E-05 | 0.42168999 | 4 | 4 | BC026590 | 668.5 | 4 | 0.65714742 | 301340 | 0.52683504 |
| 808 | 2.4275686 | 2.84E-05 | 0.41193481 | 4 | 4 | BC017643 | 624.75 | 4 | 0.64310785 | 286214 | 0.51001635 |
| 809 | 2.79004467 | 1.00E-08 | 0.3584172 | 4 | 4 | BC003965 | 939.75 | 4 | 0.55248883 | 18 | 0.99973376 |
| 810 | 2.3586471 | 2.87E-05 | 0.42397186 | 4 | 4 | Baz1b | 717.5 | 4 | 0.66033823 | 293386 | 0.56108066 |
| 811 | 2.79004467 | 1.00E-08 | 0.3584172 | 4 | 4 | Bank1 | 939.75 | 4 | 0.55248883 | 18 | 0.99973376 |
| 812 | 2.79004467 | 1.00E-08 | 0.3584172 | 4 | 4 | Bach1 | 939.75 | 4 | 0.55248883 | 18 | 0.99973376 |
| 813 | 2.79004467 | 1.00E-08 | 0.3584172 | 4 | 4 | B9d1 | 939.75 | 4 | 0.55248883 | 18 | 0.99973376 |
| 814 | 2.3586471 | 2.87E-05 | 0.42397186 | 4 | 4 | B4galt6 | 717.5 | 4 | 0.66033823 | 293386 | 0.56108066 |
| 815 | 2.79004467 | 1.00E-08 | 0.3584172 | 4 | 4 | B3gat3 | 939.75 | 4 | 0.55248883 | 18 | 0.99973376 |
| 816 | 2.79004467 | 1.00E-08 | 0.3584172 | 4 | 4 | AW146154 | 939.75 | 4 | 0.55248883 | 18 | 0.99973376 |
| 817 | 2.79004467 | 1.00E-08 | 0.3584172 | 4 | 4 | Atpaf2 | 939.75 | 4 | 0.55248883 | 18 | 0.99973376 |
| 818 | 2.79004467 | 1.00E-08 | 0.3584172 | 4 | 4 | Atp6v1f | 939.75 | 4 | 0.55248883 | 18 | 0.99973376 |
| 819 | 2.3586471 | 2.87E-05 | 0.42397186 | 4 | 4 | Atg14 | 717.5 | 4 | 0.66033823 | 293386 | 0.56108066 |
| 820 | 2.79004467 | 1.00E-08 | 0.3584172 | 4 | 4 | Atf7ip | 939.75 | 4 | 0.55248883 | 18 | 0.99973376 |
| 821 | 2.79004467 | 1.00E-08 | 0.3584172 | 4 | 4 | Asf1b | 939.75 | 4 | 0.55248883 | 18 | 0.99973376 |
| 822 | 2.38289726 | 2.86E-05 | 0.4196572 | 4 | 4 | Arl4c | 678.5 | 4 | 0.65427569 | 288318 | 0.53855326 |
| 823 | 2.79004467 | 1.00E-08 | 0.3584172 | 4 | 4 | Arid3b | 939.75 | 4 | 0.55248883 | 18 | 0.99973376 |
| 824 | 2.3586471 | 3.03E-05 | 0.42397186 | 4 | 4 | Arhgef18 | 703.75 | 4 | 0.66033823 | 306586 | 0.55031323 |
| 825 | 2.44160817 | 2.56E-05 | 0.40956613 | 4 | 4 | Arhgdia | 566.75 | 4 | 0.63959796 | 197036 | 0.46679043 |
| 826 | 2.3586471 | 2.87E-05 | 0.42397186 | 4 | 4 | Arhgap32 | 717.5 | 4 | 0.66033823 | 293386 | 0.56108066 |
| 827 | 2.3586471 | 3.03E-05 | 0.42397186 | 4 | 4 | Aptx | 703.75 | 4 | 0.66033823 | 306586 | 0.55031323 |
| 828 | 2.79004467 | 1.00E-08 | 0.3584172 | 4 | 4 | Aprt | 939.75 | 4 | 0.55248883 | 18 | 0.99973376 |
| 829 | 2.79004467 | 1.00E-08 | 0.3584172 | 4 | 4 | Apoa4 | 939.75 | 4 | 0.55248883 | 18 | 0.99973376 |
| 830 | 2.37141034 | 2.95E-05 | 0.42168999 | 4 | 4 | Aph1c | 668.5 | 4 | 0.65714742 | 301340 | 0.52683504 |
| 831 | 2.79004467 | 1.00E-08 | 0.3584172 | 4 | 4 | Apc2 | 939.75 | 4 | 0.55248883 | 18 | 0.99973376 |
| 832 | 2.3586471 | 3.03E-05 | 0.42397186 | 4 | 4 | Ap2b1 | 703.75 | 4 | 0.66033823 | 306586 | 0.55031323 |
| 833 | 2.79004467 | 1.00E-08 | 0.3584172 | 4 | 4 | Anxa8 | 939.75 | 4 | 0.55248883 | 18 | 0.99973376 |
| 834 | 2.79004467 | 1.00E-08 | 0.3584172 | 4 | 4 | Anks6 | 939.75 | 4 | 0.55248883 | 18 | 0.99973376 |
| 835 | 2.79004467 | 1.00E-08 | 0.3584172 | 4 | 4 | Ankrd66 | 939.75 | 4 | 0.55248883 | 18 | 0.99973376 |
| 836 | 2.79004467 | 1.00E-08 | 0.3584172 | 4 | 4 | Ankdd1b | 939.75 | 4 | 0.55248883 | 18 | 0.99973376 |
| 837 | 2.79004467 | 1.00E-08 | 0.3584172 | 4 | 4 | Amdhd1 | 939.75 | 4 | 0.55248883 | 18 | 0.99973376 |
| 838 | 2.79004467 | 1.00E-08 | 0.3584172 | 4 | 4 | Alpi | 939.75 | 4 | 0.55248883 | 18 | 0.99973376 |
| 839 | 2.79004467 | 1.00E-08 | 0.3584172 | 4 | 4 | Alox12e | 939.75 | 4 | 0.55248883 | 18 | 0.99973376 |
| 840 | 2.79004467 | 1.00E-08 | 0.3584172 | 4 | 4 | Alox12 | 939.75 | 4 | 0.55248883 | 18 | 0.99973376 |
| 841 | 2.79004467 | 1.00E-08 | 0.3584172 | 4 | 4 | Aldob | 939.75 | 4 | 0.55248883 | 18 | 0.99973376 |
| 842 | 2.79004467 | 1.00E-08 | 0.3584172 | 4 | 4 | Aldh3b1 | 939.75 | 4 | 0.55248883 | 18 | 0.99973376 |
| 843 | 2.79004467 | 1.00E-08 | 0.3584172 | 4 | 4 | Aldh2 | 939.75 | 4 | 0.55248883 | 18 | 0.99973376 |
| 844 | 2.79004467 | 1.00E-08 | 0.3584172 | 4 | 4 | Aldh1a3 | 939.75 | 4 | 0.55248883 | 18 | 0.99973376 |
| 845 | 2.79004467 | 1.00E-08 | 0.3584172 | 4 | 4 | Akr1b8 | 939.75 | 4 | 0.55248883 | 18 | 0.99973376 |
| 846 | 2.79004467 | 1.00E-08 | 0.3584172 | 4 | 4 | Akap3 | 939.75 | 4 | 0.55248883 | 18 | 0.99973376 |
| 847 | 2.79004467 | 1.00E-08 | 0.3584172 | 4 | 4 | Akap10 | 939.75 | 4 | 0.55248883 | 18 | 0.99973376 |
| 848 | 2.79004467 | 1.00E-08 | 0.3584172 | 4 | 4 | AI987944 | 939.75 | 4 | 0.55248883 | 18 | 0.99973376 |
| 849 | 2.3586471 | 2.87E-05 | 0.42397186 | 4 | 4 | AI846148 | 717.5 | 4 | 0.66033823 | 293386 | 0.56108066 |
| 850 | 2.79004467 | 1.00E-08 | 0.3584172 | 4 | 4 | Ago3 | 939.75 | 4 | 0.55248883 | 18 | 0.99973376 |
| 851 | 2.42501595 | 2.61E-05 | 0.41236842 | 4 | 4 | Ago1 | 556.75 | 4 | 0.64374601 | 206794 | 0.45367347 |
| 852 | 2.79004467 | 1.00E-08 | 0.3584172 | 4 | 4 | Agap3 | 939.75 | 4 | 0.55248883 | 18 | 0.99973376 |
| 853 | 2.3586471 | 2.87E-05 | 0.42397186 | 4 | 4 | Agap1 | 717.5 | 4 | 0.66033823 | 293386 | 0.56108066 |
| 854 | 2.79004467 | 1.00E-08 | 0.3584172 | 4 | 4 | Add1 | 939.75 | 4 | 0.55248883 | 18 | 0.99973376 |
| 855 | 2.79004467 | 1.00E-08 | 0.3584172 | 4 | 4 | Aco2 | 939.75 | 4 | 0.55248883 | 18 | 0.99973376 |
| 856 | 2.79004467 | 1.00E-08 | 0.3584172 | 4 | 4 | Acaa2 | 939.75 | 4 | 0.55248883 | 18 | 0.99973376 |
| 857 | 2.79004467 | 1.00E-08 | 0.3584172 | 4 | 4 | Abce1 | 939.75 | 4 | 0.55248883 | 18 | 0.99973376 |
| 858 | 2.79004467 | 1.00E-08 | 0.3584172 | 4 | 4 | Abcc6 | 939.75 | 4 | 0.55248883 | 18 | 0.99973376 |
| 859 | 2.3586471 | 2.87E-05 | 0.42397186 | 4 | 4 | Abcc5 | 717.5 | 4 | 0.66033823 | 293386 | 0.56108066 |
| 860 | 2.79004467 | 1.00E-08 | 0.3584172 | 4 | 4 | Abcb9 | 939.75 | 4 | 0.55248883 | 18 | 0.99973376 |
| 861 | 2.79004467 | 1.00E-08 | 0.3584172 | 4 | 4 | Abca2 | 939.75 | 4 | 0.55248883 | 18 | 0.99973376 |
| 862 | 2.3586471 | 2.87E-05 | 0.42397186 | 4 | 4 | Aagab | 717.5 | 4 | 0.66033823 | 293386 | 0.56108066 |
| 863 | 2.4275686 | 2.84E-05 | 0.41193481 | 4 | 4 | 8430427H17Rik | 624.75 | 4 | 0.64310785 | 286214 | 0.51001635 |
| 864 | 2.79004467 | 1.00E-08 | 0.3584172 | 4 | 4 | 5830473C10Rik | 939.75 | 4 | 0.55248883 | 18 | 0.99973376 |
| 865 | 2.79004467 | 1.00E-08 | 0.3584172 | 4 | 4 | 5830403L16Rik | 939.75 | 4 | 0.55248883 | 18 | 0.99973376 |
| 866 | 2.79004467 | 1.00E-08 | 0.3584172 | 4 | 4 | 4931429L15Rik | 939.75 | 4 | 0.55248883 | 18 | 0.99973376 |
| 867 | 2.79004467 | 1.00E-08 | 0.3584172 | 4 | 4 | 4930519G04Rik | 939.75 | 4 | 0.55248883 | 18 | 0.99973376 |
| 868 | 2.79004467 | 1.00E-08 | 0.3584172 | 4 | 4 | 4930422G04Rik | 939.75 | 4 | 0.55248883 | 18 | 0.99973376 |
| 869 | 2.43650287 | 2.54E-05 | 0.41042431 | 4 | 4 | 4632428N05Rik | 570.5 | 4 | 0.64087428 | 198680 | 0.46833882 |
| 870 | 2.79004467 | 1.00E-08 | 0.3584172 | 4 | 4 | 2610528A11Rik | 939.75 | 4 | 0.55248883 | 18 | 0.99973376 |
| 871 | 2.79004467 | 1.00E-08 | 0.3584172 | 4 | 4 | 2610034B18Rik | 939.75 | 4 | 0.55248883 | 18 | 0.99973376 |
| 872 | 2.79004467 | 1.00E-08 | 0.3584172 | 4 | 4 | 2310067B10Rik | 939.75 | 4 | 0.55248883 | 18 | 0.99973376 |
| 873 | 2.79004467 | 1.00E-08 | 0.3584172 | 4 | 4 | 2310047M10Rik | 939.75 | 4 | 0.55248883 | 18 | 0.99973376 |
| 874 | 2.79004467 | 1.00E-08 | 0.3584172 | 4 | 4 | 2310033P09Rik | 939.75 | 4 | 0.55248883 | 18 | 0.99973376 |
| 875 | 2.37141034 | 2.95E-05 | 0.42168999 | 4 | 4 | 1810041L15Rik | 668.5 | 4 | 0.65714742 | 301340 | 0.52683504 |
| 876 | 2.79004467 | 1.00E-08 | 0.3584172 | 4 | 4 | 1810037I17Rik | 939.75 | 4 | 0.55248883 | 18 | 0.99973376 |
| 877 | 2.79004467 | 1.00E-08 | 0.3584172 | 4 | 4 | 1700071K01Rik | 939.75 | 4 | 0.55248883 | 18 | 0.99973376 |
| 878 | 2.79004467 | 1.00E-08 | 0.3584172 | 4 | 4 | 1700019N19Rik | 939.75 | 4 | 0.55248883 | 18 | 0.99973376 |
| 879 | 2.79004467 | 1.00E-08 | 0.3584172 | 4 | 4 | 1700018B08Rik | 939.75 | 4 | 0.55248883 | 18 | 0.99973376 |
| 880 | 2.79004467 | 1.00E-08 | 0.3584172 | 4 | 4 | 1110065P20Rik | 939.75 | 4 | 0.55248883 | 18 | 0.99973376 |
| 881 | 2.79004467 | 1.00E-08 | 0.3584172 | 4 | 4 | 0610011F06Rik | 939.75 | 4 | 0.55248883 | 18 | 0.99973376 |
| 72 | 2.44033184 | 1.59E-05 | 0.40978033 | 3 | 4 | Marcksl1 | 698.6666667 | 3 | 0.63991704 | 162114 | 0.57468424 |
| 73 | 2.46330568 | 1.38E-05 | 0.40595855 | 3 | 4 | Mapkapk3 | 730.3333333 | 3 | 0.63417358 | 140490 | 0.60981048 |
| 74 | 2.43395022 | 1.49E-05 | 0.41085475 | 3 | 4 | Mapkap1 | 764 | 3 | 0.64151244 | 151398 | 0.62592289 |
| 75 | 2.57817486 | 1.24E-05 | 0.38787129 | 3 | 4 | Mapk14 | 516 | 3 | 0.60545629 | 85436 | 0.46564195 |
| 76 | 2.46330568 | 1.38E-05 | 0.40595855 | 3 | 4 | Map9 | 730.3333333 | 3 | 0.63417358 | 140490 | 0.60981048 |
| 77 | 2.43395022 | 1.49E-05 | 0.41085475 | 3 | 4 | Map3k2 | 764 | 3 | 0.64151244 | 151398 | 0.62592289 |
| 78 | 2.52074027 | 1.41E-05 | 0.39670886 | 3 | 4 | Maneal | 658.6666667 | 3 | 0.61981493 | 142934 | 0.5713872 |
| 79 | 2.45309509 | 1.47E-05 | 0.40764828 | 3 | 4 | Man2a2 | 717 | 3 | 0.63672623 | 151950 | 0.59468439 |
| 80 | 2.52074027 | 1.41E-05 | 0.39670886 | 3 | 4 | Man1c1 | 658.6666667 | 3 | 0.61981493 | 142934 | 0.5713872 |
| 81 | 2.56285897 | 1.39E-05 | 0.39018924 | 3 | 4 | Maml3 | 606.6666667 | 3 | 0.60928526 | 138682 | 0.5417412 |
| 82 | 2.44033184 | 1.59E-05 | 0.40978033 | 3 | 4 | Maml2 | 698.6666667 | 3 | 0.63991704 | 162114 | 0.57468424 |
| 83 | 2.52074027 | 1.41E-05 | 0.39670886 | 3 | 4 | Maff | 658.6666667 | 3 | 0.61981493 | 142934 | 0.5713872 |
| 84 | 2.45309509 | 1.47E-05 | 0.40764828 | 3 | 4 | Mab21l3 | 717 | 3 | 0.63672623 | 151950 | 0.59468439 |
| 85 | 2.44033184 | 1.59E-05 | 0.40978033 | 3 | 4 | Lysmd4 | 698.6666667 | 3 | 0.63991704 | 162114 | 0.57468424 |
| 86 | 2.48245054 | 1.44E-05 | 0.40282776 | 3 | 4 | Lyrm9 | 665 | 3 | 0.62938736 | 145600 | 0.56223539 |
| 87 | 2.43395022 | 1.49E-05 | 0.41085475 | 3 | 4 | Lyrm7 | 764 | 3 | 0.64151244 | 151398 | 0.62592289 |
| 88 | 2.55647735 | 1.41E-05 | 0.39116326 | 3 | 4 | Ly6e | 593.3333333 | 3 | 0.61088066 | 143016 | 0.52745622 |
| 89 | 2.45309509 | 1.47E-05 | 0.40764828 | 3 | 4 | Luc7l2 | 717 | 3 | 0.63672623 | 151950 | 0.59468439 |
| 90 | 2.46330568 | 1.38E-05 | 0.40595855 | 3 | 4 | Lrrc71 | 730.3333333 | 3 | 0.63417358 | 140490 | 0.60981048 |
| 91 | 2.46330568 | 1.38E-05 | 0.40595855 | 3 | 4 | Lrrc58 | 730.3333333 | 3 | 0.63417358 | 140490 | 0.60981048 |
| 92 | 2.73388641 | 1.10E-05 | 0.36577965 | 3 | 4 | Lrch3 | 444.3333333 | 3 | 0.5665284 | 77434 | 0.45054201 |
| 93 | 2.43395022 | 1.49E-05 | 0.41085475 | 3 | 4 | Lox | 764 | 3 | 0.64151244 | 151398 | 0.62592289 |
| 94 | 2.52074027 | 1.41E-05 | 0.39670886 | 3 | 4 | LOC101056104 | 658.6666667 | 3 | 0.61981493 | 142934 | 0.5713872 |
| 95 | 2.51818762 | 1.47E-05 | 0.397111 | 3 | 4 | LOC100862594 | 640.3333333 | 3 | 0.6204531 | 147796 | 0.55449552 |
| 96 | 2.43395022 | 1.49E-05 | 0.41085475 | 3 | 4 | LOC100504821 | 764 | 3 | 0.64151244 | 151398 | 0.62592289 |
| 97 | 2.54499043 | 1.30E-05 | 0.39292879 | 3 | 4 | Lman2l | 568 | 3 | 0.61375239 | 88604 | 0.50088339 |
| 98 | 2.45820038 | 1.51E-05 | 0.40680166 | 3 | 4 | Llgl1 | 712 | 3 | 0.6354499 | 151864 | 0.5925 |
| 99 | 2.48245054 | 1.44E-05 | 0.40282776 | 3 | 4 | Lins | 665 | 3 | 0.62938736 | 145600 | 0.56223539 |
| 100 | 2.48245054 | 1.44E-05 | 0.40282776 | 3 | 4 | Lingo3 | 665 | 3 | 0.62938736 | 145600 | 0.56223539 |
| 101 | 2.54499043 | 1.30E-05 | 0.39292879 | 3 | 4 | Lin7a | 568 | 3 | 0.61375239 | 88604 | 0.50088339 |
| 102 | 2.46330568 | 1.38E-05 | 0.40595855 | 3 | 4 | Limd1 | 730.3333333 | 3 | 0.63417358 | 140490 | 0.60981048 |
| 103 | 2.45309509 | 1.47E-05 | 0.40764828 | 3 | 4 | Lgi1 | 717 | 3 | 0.63672623 | 151950 | 0.59468439 |
| 104 | 2.44033184 | 1.59E-05 | 0.40978033 | 3 | 4 | Leng8 | 698.6666667 | 3 | 0.63991704 | 162114 | 0.57468424 |
| 105 | 2.43395022 | 1.49E-05 | 0.41085475 | 3 | 4 | Lclat1 | 764 | 3 | 0.64151244 | 151398 | 0.62592289 |
| 106 | 2.51818762 | 1.47E-05 | 0.397111 | 3 | 4 | Lasp1 | 640.3333333 | 3 | 0.6204531 | 147796 | 0.55449552 |
| 107 | 2.46330568 | 1.38E-05 | 0.40595855 | 3 | 4 | L1cam | 730.3333333 | 3 | 0.63417358 | 140490 | 0.60981048 |
| 108 | 2.55775367 | 1.25E-05 | 0.39096806 | 3 | 4 | Ksr1 | 581.3333333 | 3 | 0.61056158 | 80408 | 0.51723113 |
| 109 | 2.44033184 | 1.59E-05 | 0.40978033 | 3 | 4 | Kras | 698.6666667 | 3 | 0.63991704 | 162114 | 0.57468424 |
| 110 | 2.45820038 | 1.51E-05 | 0.40680166 | 3 | 4 | Kndc1 | 712 | 3 | 0.6354499 | 151864 | 0.5925 |
| 111 | 2.43395022 | 1.49E-05 | 0.41085475 | 3 | 4 | Klrk1 | 764 | 3 | 0.64151244 | 151398 | 0.62592289 |
| 112 | 2.52074027 | 1.41E-05 | 0.39670886 | 3 | 4 | Klhl40 | 658.6666667 | 3 | 0.61981493 | 142934 | 0.5713872 |
| 113 | 2.43395022 | 1.49E-05 | 0.41085475 | 3 | 4 | Klhl24 | 764 | 3 | 0.64151244 | 151398 | 0.62592289 |
| 114 | 2.46330568 | 1.38E-05 | 0.40595855 | 3 | 4 | Klhl12 | 730.3333333 | 3 | 0.63417358 | 140490 | 0.60981048 |
| 115 | 2.55647735 | 1.41E-05 | 0.39116326 | 3 | 4 | Klhdc8a | 593.3333333 | 3 | 0.61088066 | 143016 | 0.52745622 |
| 116 | 2.51818762 | 1.47E-05 | 0.397111 | 3 | 4 | Klf8 | 640.3333333 | 3 | 0.6204531 | 147796 | 0.55449552 |
| 117 | 2.44033184 | 1.59E-05 | 0.40978033 | 3 | 4 | Klf17 | 698.6666667 | 3 | 0.63991704 | 162114 | 0.57468424 |
| 118 | 2.45820038 | 1.51E-05 | 0.40680166 | 3 | 4 | Kirrel3 | 712 | 3 | 0.6354499 | 151864 | 0.5925 |
| 119 | 2.52074027 | 1.41E-05 | 0.39670886 | 3 | 4 | Kif5b | 658.6666667 | 3 | 0.61981493 | 142934 | 0.5713872 |
| 120 | 2.51818762 | 1.47E-05 | 0.397111 | 3 | 4 | Kif3c | 640.3333333 | 3 | 0.6204531 | 147796 | 0.55449552 |
| 121 | 2.45820038 | 1.51E-05 | 0.40680166 | 3 | 4 | Kif3a | 712 | 3 | 0.6354499 | 151864 | 0.5925 |
| 122 | 2.48245054 | 1.44E-05 | 0.40282776 | 3 | 4 | Kif1c | 665 | 3 | 0.62938736 | 145600 | 0.56223539 |
| 123 | 2.43395022 | 1.49E-05 | 0.41085475 | 3 | 4 | Kdelr2 | 764 | 3 | 0.64151244 | 151398 | 0.62592289 |
| 124 | 2.52074027 | 1.41E-05 | 0.39670886 | 3 | 4 | Kctd11 | 658.6666667 | 3 | 0.61981493 | 142934 | 0.5713872 |
| 125 | 2.55775367 | 1.25E-05 | 0.39096806 | 3 | 4 | Kcnn4 | 581.3333333 | 3 | 0.61056158 | 80408 | 0.51723113 |
| 126 | 2.45309509 | 1.47E-05 | 0.40764828 | 3 | 4 | Kcnn3 | 717 | 3 | 0.63672623 | 151950 | 0.59468439 |
| 127 | 2.45820038 | 1.51E-05 | 0.40680166 | 3 | 4 | Kcnmb1 | 712 | 3 | 0.6354499 | 151864 | 0.5925 |
| 128 | 2.55647735 | 1.41E-05 | 0.39116326 | 3 | 4 | Kcnj9 | 593.3333333 | 3 | 0.61088066 | 143016 | 0.52745622 |
| 129 | 2.51818762 | 1.47E-05 | 0.397111 | 3 | 4 | Kcnc3 | 640.3333333 | 3 | 0.6204531 | 147796 | 0.55449552 |
| 130 | 2.46330568 | 1.38E-05 | 0.40595855 | 3 | 4 | Kcna7 | 730.3333333 | 3 | 0.63417358 | 140490 | 0.60981048 |
| 131 | 2.45309509 | 1.47E-05 | 0.40764828 | 3 | 4 | Kcna6 | 717 | 3 | 0.63672623 | 151950 | 0.59468439 |
| 132 | 2.8181238 | 1.03E-05 | 0.35484601 | 3 | 4 | Katnal1 | 410.6666667 | 3 | 0.54546905 | 69772 | 0.44625999 |
| 133 | 2.51818762 | 1.47E-05 | 0.397111 | 3 | 4 | Kansl1l | 640.3333333 | 3 | 0.6204531 | 147796 | 0.55449552 |
| 134 | 2.48245054 | 1.44E-05 | 0.40282776 | 3 | 4 | Kalrn | 665 | 3 | 0.62938736 | 145600 | 0.56223539 |
| 135 | 2.46330568 | 1.38E-05 | 0.40595855 | 3 | 4 | Jhdm1d | 730.3333333 | 3 | 0.63417358 | 140490 | 0.60981048 |
| 136 | 2.45820038 | 1.51E-05 | 0.40680166 | 3 | 4 | Jarid2 | 712 | 3 | 0.6354499 | 151864 | 0.5925 |
| 137 | 2.71474154 | 1.12E-05 | 0.36835919 | 3 | 4 | Itprip | 457.6666667 | 3 | 0.57131461 | 76364 | 0.45712379 |
| 138 | 2.54499043 | 1.30E-05 | 0.39292879 | 3 | 4 | Itpr3 | 568 | 3 | 0.61375239 | 88604 | 0.50088339 |
| 139 | 2.56285897 | 1.39E-05 | 0.39018924 | 3 | 4 | Itgb3 | 606.6666667 | 3 | 0.60928526 | 138682 | 0.5417412 |
| 140 | 2.45309509 | 1.47E-05 | 0.40764828 | 3 | 4 | Itgal | 717 | 3 | 0.63672623 | 151950 | 0.59468439 |
| 141 | 2.45820038 | 1.51E-05 | 0.40680166 | 3 | 4 | Itga9 | 712 | 3 | 0.6354499 | 151864 | 0.5925 |
| 142 | 2.45820038 | 1.51E-05 | 0.40680166 | 3 | 4 | Itga4 | 712 | 3 | 0.6354499 | 151864 | 0.5925 |
| 143 | 2.55647735 | 1.41E-05 | 0.39116326 | 3 | 4 | Itga11 | 593.3333333 | 3 | 0.61088066 | 143016 | 0.52745622 |
| 144 | 2.43395022 | 1.49E-05 | 0.41085475 | 3 | 4 | Ip6k1 | 764 | 3 | 0.64151244 | 151398 | 0.62592289 |
| 145 | 2.46330568 | 1.38E-05 | 0.40595855 | 3 | 4 | Intu | 730.3333333 | 3 | 0.63417358 | 140490 | 0.60981048 |
| 146 | 2.43395022 | 1.49E-05 | 0.41085475 | 3 | 4 | Inpp5d | 764 | 3 | 0.64151244 | 151398 | 0.62592289 |
| 147 | 2.58966177 | 1.20E-05 | 0.38615081 | 3 | 4 | Inip | 534.3333333 | 3 | 0.60258456 | 79148 | 0.48617442 |
| 148 | 2.44033184 | 1.59E-05 | 0.40978033 | 3 | 4 | Impact | 698.6666667 | 3 | 0.63991704 | 162114 | 0.57468424 |
| 149 | 2.46330568 | 1.38E-05 | 0.40595855 | 3 | 4 | Il6st | 730.3333333 | 3 | 0.63417358 | 140490 | 0.60981048 |
| 150 | 2.46330568 | 1.38E-05 | 0.40595855 | 3 | 4 | Il6ra | 730.3333333 | 3 | 0.63417358 | 140490 | 0.60981048 |
| 151 | 2.56285897 | 1.39E-05 | 0.39018924 | 3 | 4 | Il10ra | 606.6666667 | 3 | 0.60928526 | 138682 | 0.5417412 |
| 152 | 2.43395022 | 1.49E-05 | 0.41085475 | 3 | 4 | Ikzf1 | 764 | 3 | 0.64151244 | 151398 | 0.62592289 |
| 153 | 2.45820038 | 1.51E-05 | 0.40680166 | 3 | 4 | Ighmbp2 | 712 | 3 | 0.6354499 | 151864 | 0.5925 |
| 154 | 2.56285897 | 1.39E-05 | 0.39018924 | 3 | 4 | Igf2 | 606.6666667 | 3 | 0.60928526 | 138682 | 0.5417412 |
| 155 | 2.51818762 | 1.47E-05 | 0.397111 | 3 | 4 | Ifnar1 | 640.3333333 | 3 | 0.6204531 | 147796 | 0.55449552 |
| 156 | 2.46330568 | 1.38E-05 | 0.40595855 | 3 | 4 | Icmt | 730.3333333 | 3 | 0.63417358 | 140490 | 0.60981048 |
| 157 | 2.43395022 | 1.49E-05 | 0.41085475 | 3 | 4 | Hus1 | 764 | 3 | 0.64151244 | 151398 | 0.62592289 |
| 158 | 2.46330568 | 1.38E-05 | 0.40595855 | 3 | 4 | Hunk | 730.3333333 | 3 | 0.63417358 | 140490 | 0.60981048 |
| 159 | 2.43395022 | 1.49E-05 | 0.41085475 | 3 | 4 | Htr2c | 764 | 3 | 0.64151244 | 151398 | 0.62592289 |
| 160 | 2.44033184 | 1.59E-05 | 0.40978033 | 3 | 4 | Hspg2 | 698.6666667 | 3 | 0.63991704 | 162114 | 0.57468424 |
| 161 | 2.52074027 | 1.41E-05 | 0.39670886 | 3 | 4 | Hspa4 | 658.6666667 | 3 | 0.61981493 | 142934 | 0.5713872 |
| 162 | 2.57817486 | 1.24E-05 | 0.38787129 | 3 | 4 | Hsd17b7 | 516 | 3 | 0.60545629 | 85436 | 0.46564195 |
| 163 | 2.45820038 | 1.51E-05 | 0.40680166 | 3 | 4 | Hsbp1 | 712 | 3 | 0.6354499 | 151864 | 0.5925 |
| 164 | 2.46330568 | 1.38E-05 | 0.40595855 | 3 | 4 | Hpgds | 730.3333333 | 3 | 0.63417358 | 140490 | 0.60981048 |
| 165 | 2.51818762 | 1.47E-05 | 0.397111 | 3 | 4 | Hp1bp3 | 640.3333333 | 3 | 0.6204531 | 147796 | 0.55449552 |
| 166 | 2.51818762 | 1.47E-05 | 0.397111 | 3 | 4 | Hoxb3 | 640.3333333 | 3 | 0.6204531 | 147796 | 0.55449552 |
| 167 | 2.43395022 | 1.49E-05 | 0.41085475 | 3 | 4 | Hoga1 | 764 | 3 | 0.64151244 | 151398 | 0.62592289 |
| 168 | 2.43395022 | 1.49E-05 | 0.41085475 | 3 | 4 | Hnrnpa3 | 764 | 3 | 0.64151244 | 151398 | 0.62592289 |
| 169 | 2.46330568 | 1.38E-05 | 0.40595855 | 3 | 4 | Hnf4g | 730.3333333 | 3 | 0.63417358 | 140490 | 0.60981048 |
| 170 | 2.71474154 | 1.12E-05 | 0.36835919 | 3 | 4 | Hnf1a | 457.6666667 | 3 | 0.57131461 | 76364 | 0.45712379 |
| 171 | 2.71474154 | 1.12E-05 | 0.36835919 | 3 | 4 | Hmga2 | 457.6666667 | 3 | 0.57131461 | 76364 | 0.45712379 |
| 172 | 2.45820038 | 1.51E-05 | 0.40680166 | 3 | 4 | Hist1h1c | 712 | 3 | 0.6354499 | 151864 | 0.5925 |
| 173 | 2.46330568 | 1.38E-05 | 0.40595855 | 3 | 4 | Hexim2 | 730.3333333 | 3 | 0.63417358 | 140490 | 0.60981048 |
| 174 | 2.45309509 | 1.47E-05 | 0.40764828 | 3 | 4 | Heca | 717 | 3 | 0.63672623 | 151950 | 0.59468439 |
| 175 | 2.43395022 | 1.49E-05 | 0.41085475 | 3 | 4 | Hdhd2 | 764 | 3 | 0.64151244 | 151398 | 0.62592289 |
| 176 | 2.45309509 | 1.47E-05 | 0.40764828 | 3 | 4 | Hdgfrp3 | 717 | 3 | 0.63672623 | 151950 | 0.59468439 |
| 177 | 2.43395022 | 1.49E-05 | 0.41085475 | 3 | 4 | Hapln4 | 764 | 3 | 0.64151244 | 151398 | 0.62592289 |
| 178 | 2.46330568 | 1.38E-05 | 0.40595855 | 3 | 4 | Gzf1 | 730.3333333 | 3 | 0.63417358 | 140490 | 0.60981048 |
| 179 | 2.44033184 | 1.59E-05 | 0.40978033 | 3 | 4 | Gtf3c4 | 698.6666667 | 3 | 0.63991704 | 162114 | 0.57468424 |
| 180 | 2.46330568 | 1.38E-05 | 0.40595855 | 3 | 4 | Gtf2h3 | 730.3333333 | 3 | 0.63417358 | 140490 | 0.60981048 |
| 181 | 2.45820038 | 1.51E-05 | 0.40680166 | 3 | 4 | Gsk3b | 712 | 3 | 0.6354499 | 151864 | 0.5925 |
| 182 | 2.44033184 | 1.59E-05 | 0.40978033 | 3 | 4 | Gpr56 | 698.6666667 | 3 | 0.63991704 | 162114 | 0.57468424 |
| 183 | 2.52074027 | 1.41E-05 | 0.39670886 | 3 | 4 | Gpr179 | 658.6666667 | 3 | 0.61981493 | 142934 | 0.5713872 |
| 184 | 2.45820038 | 1.51E-05 | 0.40680166 | 3 | 4 | Gpr17 | 712 | 3 | 0.6354499 | 151864 | 0.5925 |
| 185 | 2.54499043 | 1.30E-05 | 0.39292879 | 3 | 4 | Gpr126 | 568 | 3 | 0.61375239 | 88604 | 0.50088339 |
| 186 | 2.51818762 | 1.47E-05 | 0.397111 | 3 | 4 | Gpr124 | 640.3333333 | 3 | 0.6204531 | 147796 | 0.55449552 |
| 187 | 2.44033184 | 1.59E-05 | 0.40978033 | 3 | 4 | Gpr123 | 698.6666667 | 3 | 0.63991704 | 162114 | 0.57468424 |
| 188 | 2.43395022 | 1.49E-05 | 0.41085475 | 3 | 4 | Gpr101 | 764 | 3 | 0.64151244 | 151398 | 0.62592289 |
| 189 | 2.45309509 | 1.47E-05 | 0.40764828 | 3 | 4 | Gpm6a | 717 | 3 | 0.63672623 | 151950 | 0.59468439 |
| 190 | 2.43395022 | 1.49E-05 | 0.41085475 | 3 | 4 | Gper1 | 764 | 3 | 0.64151244 | 151398 | 0.62592289 |
| 191 | 2.45820038 | 1.51E-05 | 0.40680166 | 3 | 4 | Gpd1l | 712 | 3 | 0.6354499 | 151864 | 0.5925 |
| 192 | 2.55647735 | 1.41E-05 | 0.39116326 | 3 | 4 | Gpc6 | 593.3333333 | 3 | 0.61088066 | 143016 | 0.52745622 |
| 193 | 2.44033184 | 1.59E-05 | 0.40978033 | 3 | 4 | Gpalpp1 | 698.6666667 | 3 | 0.63991704 | 162114 | 0.57468424 |
| 194 | 2.48245054 | 1.44E-05 | 0.40282776 | 3 | 4 | Gosr1 | 665 | 3 | 0.62938736 | 145600 | 0.56223539 |
| 195 | 2.43395022 | 1.49E-05 | 0.41085475 | 3 | 4 | Gopc | 764 | 3 | 0.64151244 | 151398 | 0.62592289 |
| 196 | 2.48245054 | 1.44E-05 | 0.40282776 | 3 | 4 | Golga2 | 665 | 3 | 0.62938736 | 145600 | 0.56223539 |
| 197 | 2.7070836 | 1.13E-05 | 0.36940123 | 3 | 4 | Gns | 462.6666667 | 3 | 0.5732291 | 77658 | 0.45936982 |
| 198 | 2.45309509 | 1.47E-05 | 0.40764828 | 3 | 4 | Gng2 | 717 | 3 | 0.63672623 | 151950 | 0.59468439 |
| 199 | 2.46330568 | 1.38E-05 | 0.40595855 | 3 | 4 | Gnb1l | 730.3333333 | 3 | 0.63417358 | 140490 | 0.60981048 |
| 200 | 2.48245054 | 1.44E-05 | 0.40282776 | 3 | 4 | Gmeb2 | 665 | 3 | 0.62938736 | 145600 | 0.56223539 |
| 201 | 2.44033184 | 1.59E-05 | 0.40978033 | 3 | 4 | Gm7969 | 698.6666667 | 3 | 0.63991704 | 162114 | 0.57468424 |
| 202 | 2.45820038 | 1.51E-05 | 0.40680166 | 3 | 4 | Gm608 | 712 | 3 | 0.6354499 | 151864 | 0.5925 |
| 203 | 2.46330568 | 1.38E-05 | 0.40595855 | 3 | 4 | Gm14322 | 730.3333333 | 3 | 0.63417358 | 140490 | 0.60981048 |
| 204 | 2.46330568 | 1.38E-05 | 0.40595855 | 3 | 4 | Gm11541 | 730.3333333 | 3 | 0.63417358 | 140490 | 0.60981048 |
| 205 | 2.52074027 | 1.41E-05 | 0.39670886 | 3 | 4 | Gm10113 | 658.6666667 | 3 | 0.61981493 | 142934 | 0.5713872 |
| 206 | 2.48245054 | 1.44E-05 | 0.40282776 | 3 | 4 | Glp2r | 665 | 3 | 0.62938736 | 145600 | 0.56223539 |
| 207 | 2.43395022 | 1.49E-05 | 0.41085475 | 3 | 4 | Glb1l2 | 764 | 3 | 0.64151244 | 151398 | 0.62592289 |
| 208 | 2.51818762 | 1.47E-05 | 0.397111 | 3 | 4 | Gk5 | 640.3333333 | 3 | 0.6204531 | 147796 | 0.55449552 |
| 209 | 2.51818762 | 1.47E-05 | 0.397111 | 3 | 4 | Git2 | 640.3333333 | 3 | 0.6204531 | 147796 | 0.55449552 |
| 210 | 2.45820038 | 1.51E-05 | 0.40680166 | 3 | 4 | Ggt5 | 712 | 3 | 0.6354499 | 151864 | 0.5925 |
| 211 | 2.57817486 | 1.24E-05 | 0.38787129 | 3 | 4 | Gfpt1 | 516 | 3 | 0.60545629 | 85436 | 0.46564195 |
| 212 | 2.45309509 | 1.47E-05 | 0.40764828 | 3 | 4 | Gemin5 | 717 | 3 | 0.63672623 | 151950 | 0.59468439 |
| 213 | 2.45309509 | 1.47E-05 | 0.40764828 | 3 | 4 | Gdnf | 717 | 3 | 0.63672623 | 151950 | 0.59468439 |
| 214 | 2.46330568 | 1.38E-05 | 0.40595855 | 3 | 4 | Gdf6 | 730.3333333 | 3 | 0.63417358 | 140490 | 0.60981048 |
| 215 | 2.43395022 | 1.49E-05 | 0.41085475 | 3 | 4 | Gcnt3 | 764 | 3 | 0.64151244 | 151398 | 0.62592289 |
| 216 | 2.45309509 | 1.47E-05 | 0.40764828 | 3 | 4 | Gcnt1 | 717 | 3 | 0.63672623 | 151950 | 0.59468439 |
| 217 | 2.45309509 | 1.47E-05 | 0.40764828 | 3 | 4 | Gch1 | 717 | 3 | 0.63672623 | 151950 | 0.59468439 |
| 218 | 2.51818762 | 1.47E-05 | 0.397111 | 3 | 4 | Gcc2 | 640.3333333 | 3 | 0.6204531 | 147796 | 0.55449552 |
| 219 | 2.43395022 | 1.49E-05 | 0.41085475 | 3 | 4 | Gbp4 | 764 | 3 | 0.64151244 | 151398 | 0.62592289 |
| 220 | 2.43395022 | 1.49E-05 | 0.41085475 | 3 | 4 | Gan | 764 | 3 | 0.64151244 | 151398 | 0.62592289 |
| 221 | 2.43395022 | 1.49E-05 | 0.41085475 | 3 | 4 | Galnt4 | 764 | 3 | 0.64151244 | 151398 | 0.62592289 |
| 222 | 2.55775367 | 1.25E-05 | 0.39096806 | 3 | 4 | Galnt2 | 581.3333333 | 3 | 0.61056158 | 80408 | 0.51723113 |
| 223 | 2.45309509 | 1.47E-05 | 0.40764828 | 3 | 4 | Gabrb2 | 717 | 3 | 0.63672623 | 151950 | 0.59468439 |
| 224 | 2.44033184 | 1.59E-05 | 0.40978033 | 3 | 4 | Gab2 | 698.6666667 | 3 | 0.63991704 | 162114 | 0.57468424 |
| 225 | 2.43395022 | 1.49E-05 | 0.41085475 | 3 | 4 | Gab1 | 764 | 3 | 0.64151244 | 151398 | 0.62592289 |
| 226 | 2.46330568 | 1.38E-05 | 0.40595855 | 3 | 4 | Fyco1 | 730.3333333 | 3 | 0.63417358 | 140490 | 0.60981048 |
| 227 | 2.43395022 | 1.49E-05 | 0.41085475 | 3 | 4 | Fut9 | 764 | 3 | 0.64151244 | 151398 | 0.62592289 |
| 228 | 2.43395022 | 1.49E-05 | 0.41085475 | 3 | 4 | Fubp1 | 764 | 3 | 0.64151244 | 151398 | 0.62592289 |
| 229 | 2.51818762 | 1.47E-05 | 0.397111 | 3 | 4 | Ftsj2 | 640.3333333 | 3 | 0.6204531 | 147796 | 0.55449552 |
| 230 | 2.44033184 | 1.59E-05 | 0.40978033 | 3 | 4 | Fto | 698.6666667 | 3 | 0.63991704 | 162114 | 0.57468424 |
| 231 | 2.73388641 | 1.10E-05 | 0.36577965 | 3 | 4 | Fstl3 | 444.3333333 | 3 | 0.5665284 | 77434 | 0.45054201 |
| 232 | 2.52074027 | 1.41E-05 | 0.39670886 | 3 | 4 | Fsd1l | 658.6666667 | 3 | 0.61981493 | 142934 | 0.5713872 |
| 233 | 2.45309509 | 1.47E-05 | 0.40764828 | 3 | 4 | Frmpd4 | 717 | 3 | 0.63672623 | 151950 | 0.59468439 |
| 234 | 2.56285897 | 1.39E-05 | 0.39018924 | 3 | 4 | Frat1 | 606.6666667 | 3 | 0.60928526 | 138682 | 0.5417412 |
| 235 | 2.45309509 | 1.47E-05 | 0.40764828 | 3 | 4 | Foxo4 | 717 | 3 | 0.63672623 | 151950 | 0.59468439 |
| 236 | 2.46330568 | 1.38E-05 | 0.40595855 | 3 | 4 | Foxf1 | 730.3333333 | 3 | 0.63417358 | 140490 | 0.60981048 |
| 237 | 2.44033184 | 1.59E-05 | 0.40978033 | 3 | 4 | Fosl2 | 698.6666667 | 3 | 0.63991704 | 162114 | 0.57468424 |
| 238 | 2.43395022 | 1.49E-05 | 0.41085475 | 3 | 4 | Fosb | 764 | 3 | 0.64151244 | 151398 | 0.62592289 |
| 239 | 2.46330568 | 1.38E-05 | 0.40595855 | 3 | 4 | Fntb | 730.3333333 | 3 | 0.63417358 | 140490 | 0.60981048 |
| 240 | 2.43395022 | 1.49E-05 | 0.41085475 | 3 | 4 | Fnta | 764 | 3 | 0.64151244 | 151398 | 0.62592289 |
| 241 | 2.45309509 | 1.47E-05 | 0.40764828 | 3 | 4 | Fndc3b | 717 | 3 | 0.63672623 | 151950 | 0.59468439 |
| 242 | 2.52074027 | 1.41E-05 | 0.39670886 | 3 | 4 | Fnbp1 | 658.6666667 | 3 | 0.61981493 | 142934 | 0.5713872 |
| 243 | 2.44033184 | 1.59E-05 | 0.40978033 | 3 | 4 | Fn3k | 698.6666667 | 3 | 0.63991704 | 162114 | 0.57468424 |
| 244 | 2.56285897 | 1.39E-05 | 0.39018924 | 3 | 4 | Flrt1 | 606.6666667 | 3 | 0.60928526 | 138682 | 0.5417412 |
| 245 | 2.44033184 | 1.59E-05 | 0.40978033 | 3 | 4 | Fkbp5 | 698.6666667 | 3 | 0.63991704 | 162114 | 0.57468424 |
| 246 | 2.46330568 | 1.38E-05 | 0.40595855 | 3 | 4 | Fignl2 | 730.3333333 | 3 | 0.63417358 | 140490 | 0.60981048 |
| 247 | 2.46330568 | 1.38E-05 | 0.40595855 | 3 | 4 | Fhdc1 | 730.3333333 | 3 | 0.63417358 | 140490 | 0.60981048 |
| 248 | 2.65220166 | 1.16E-05 | 0.37704524 | 3 | 4 | Fgfr2 | 509.6666667 | 3 | 0.58694959 | 77434 | 0.48536896 |
| 249 | 2.46330568 | 1.38E-05 | 0.40595855 | 3 | 4 | Fdft1 | 730.3333333 | 3 | 0.63417358 | 140490 | 0.60981048 |
| 250 | 2.55647735 | 1.41E-05 | 0.39116326 | 3 | 4 | Fchsd2 | 593.3333333 | 3 | 0.61088066 | 143016 | 0.52745622 |
| 251 | 2.55647735 | 1.41E-05 | 0.39116326 | 3 | 4 | Fcer2a | 593.3333333 | 3 | 0.61088066 | 143016 | 0.52745622 |
| 252 | 2.43395022 | 1.49E-05 | 0.41085475 | 3 | 4 | Fbxw11 | 764 | 3 | 0.64151244 | 151398 | 0.62592289 |
| 253 | 2.45820038 | 1.51E-05 | 0.40680166 | 3 | 4 | Fbxo10 | 712 | 3 | 0.6354499 | 151864 | 0.5925 |
| 254 | 2.52074027 | 1.41E-05 | 0.39670886 | 3 | 4 | Fbxl22 | 658.6666667 | 3 | 0.61981493 | 142934 | 0.5713872 |
| 255 | 2.45820038 | 1.51E-05 | 0.40680166 | 3 | 4 | Fbxl20 | 712 | 3 | 0.6354499 | 151864 | 0.5925 |
| 256 | 2.56285897 | 1.39E-05 | 0.39018924 | 3 | 4 | Fbxl19 | 606.6666667 | 3 | 0.60928526 | 138682 | 0.5417412 |
| 257 | 2.46330568 | 1.38E-05 | 0.40595855 | 3 | 4 | Fam83g | 730.3333333 | 3 | 0.63417358 | 140490 | 0.60981048 |
| 258 | 2.45820038 | 1.51E-05 | 0.40680166 | 3 | 4 | Fam76a | 712 | 3 | 0.6354499 | 151864 | 0.5925 |
| 259 | 2.46330568 | 1.38E-05 | 0.40595855 | 3 | 4 | Fam71e2 | 730.3333333 | 3 | 0.63417358 | 140490 | 0.60981048 |
| 260 | 2.43395022 | 1.49E-05 | 0.41085475 | 3 | 4 | Fam65b | 764 | 3 | 0.64151244 | 151398 | 0.62592289 |
| 261 | 2.45309509 | 1.47E-05 | 0.40764828 | 3 | 4 | Fam53c | 717 | 3 | 0.63672623 | 151950 | 0.59468439 |
| 262 | 2.65220166 | 1.16E-05 | 0.37704524 | 3 | 4 | Fam228a | 509.6666667 | 3 | 0.58694959 | 77434 | 0.48536896 |
| 263 | 2.52074027 | 1.41E-05 | 0.39670886 | 3 | 4 | Fam219b | 658.6666667 | 3 | 0.61981493 | 142934 | 0.5713872 |
| 264 | 2.43395022 | 1.49E-05 | 0.41085475 | 3 | 4 | Fam135a | 764 | 3 | 0.64151244 | 151398 | 0.62592289 |
| 265 | 2.46330568 | 1.38E-05 | 0.40595855 | 3 | 4 | Fam134c | 730.3333333 | 3 | 0.63417358 | 140490 | 0.60981048 |
| 266 | 2.43395022 | 1.49E-05 | 0.41085475 | 3 | 4 | Fam126b | 764 | 3 | 0.64151244 | 151398 | 0.62592289 |
| 267 | 2.43395022 | 1.49E-05 | 0.41085475 | 3 | 4 | Fam126a | 764 | 3 | 0.64151244 | 151398 | 0.62592289 |
| 268 | 2.55647735 | 1.41E-05 | 0.39116326 | 3 | 4 | Fam107a | 593.3333333 | 3 | 0.61088066 | 143016 | 0.52745622 |
| 269 | 2.46330568 | 1.38E-05 | 0.40595855 | 3 | 4 | Fam104a | 730.3333333 | 3 | 0.63417358 | 140490 | 0.60981048 |
| 270 | 2.55775367 | 1.25E-05 | 0.39096806 | 3 | 4 | Fam101b | 581.3333333 | 3 | 0.61056158 | 80408 | 0.51723113 |
| 271 | 2.46330568 | 1.38E-05 | 0.40595855 | 3 | 4 | Faf2 | 730.3333333 | 3 | 0.63417358 | 140490 | 0.60981048 |
| 272 | 2.52074027 | 1.41E-05 | 0.39670886 | 3 | 4 | Extl1 | 658.6666667 | 3 | 0.61981493 | 142934 | 0.5713872 |
| 273 | 2.43395022 | 1.49E-05 | 0.41085475 | 3 | 4 | Exph5 | 764 | 3 | 0.64151244 | 151398 | 0.62592289 |
| 274 | 2.43395022 | 1.49E-05 | 0.41085475 | 3 | 4 | Exog | 764 | 3 | 0.64151244 | 151398 | 0.62592289 |
| 275 | 2.48245054 | 1.44E-05 | 0.40282776 | 3 | 4 | Evi5 | 665 | 3 | 0.62938736 | 145600 | 0.56223539 |
| 276 | 2.57817486 | 1.24E-05 | 0.38787129 | 3 | 4 | Etv6 | 516 | 3 | 0.60545629 | 85436 | 0.46564195 |
| 277 | 2.46330568 | 1.38E-05 | 0.40595855 | 3 | 4 | Etv4 | 730.3333333 | 3 | 0.63417358 | 140490 | 0.60981048 |
| 278 | 2.45309509 | 1.47E-05 | 0.40764828 | 3 | 4 | Etv3 | 717 | 3 | 0.63672623 | 151950 | 0.59468439 |
| 279 | 2.46330568 | 1.38E-05 | 0.40595855 | 3 | 4 | Esyt2 | 730.3333333 | 3 | 0.63417358 | 140490 | 0.60981048 |
| 280 | 2.43395022 | 1.49E-05 | 0.41085475 | 3 | 4 | Esr2 | 764 | 3 | 0.64151244 | 151398 | 0.62592289 |
| 281 | 2.46330568 | 1.38E-05 | 0.40595855 | 3 | 4 | Ern1 | 730.3333333 | 3 | 0.63417358 | 140490 | 0.60981048 |
| 282 | 2.43395022 | 1.49E-05 | 0.41085475 | 3 | 4 | Erlin2 | 764 | 3 | 0.64151244 | 151398 | 0.62592289 |
| 283 | 2.44033184 | 1.59E-05 | 0.40978033 | 3 | 4 | Ergic1 | 698.6666667 | 3 | 0.63991704 | 162114 | 0.57468424 |
| 284 | 2.48245054 | 1.44E-05 | 0.40282776 | 3 | 4 | Epn2 | 665 | 3 | 0.62938736 | 145600 | 0.56223539 |
| 285 | 2.56285897 | 1.39E-05 | 0.39018924 | 3 | 4 | Epb4.1l1 | 606.6666667 | 3 | 0.60928526 | 138682 | 0.5417412 |
| 286 | 2.43395022 | 1.49E-05 | 0.41085475 | 3 | 4 | Enox2 | 764 | 3 | 0.64151244 | 151398 | 0.62592289 |
| 287 | 2.45820038 | 1.51E-05 | 0.40680166 | 3 | 4 | Emilin2 | 712 | 3 | 0.6354499 | 151864 | 0.5925 |
| 288 | 2.43395022 | 1.49E-05 | 0.41085475 | 3 | 4 | Emc8 | 764 | 3 | 0.64151244 | 151398 | 0.62592289 |
| 289 | 2.45309509 | 1.47E-05 | 0.40764828 | 3 | 4 | Elp6 | 717 | 3 | 0.63672623 | 151950 | 0.59468439 |
| 290 | 2.46330568 | 1.38E-05 | 0.40595855 | 3 | 4 | Elp3 | 730.3333333 | 3 | 0.63417358 | 140490 | 0.60981048 |
| 291 | 2.48245054 | 1.44E-05 | 0.40282776 | 3 | 4 | Elovl7 | 665 | 3 | 0.62938736 | 145600 | 0.56223539 |
| 292 | 2.45820038 | 1.51E-05 | 0.40680166 | 3 | 4 | Elmo2 | 712 | 3 | 0.6354499 | 151864 | 0.5925 |
| 293 | 2.43395022 | 1.49E-05 | 0.41085475 | 3 | 4 | Elk3 | 764 | 3 | 0.64151244 | 151398 | 0.62592289 |
| 294 | 2.43395022 | 1.49E-05 | 0.41085475 | 3 | 4 | Eif5a2 | 764 | 3 | 0.64151244 | 151398 | 0.62592289 |
| 295 | 2.46330568 | 1.38E-05 | 0.40595855 | 3 | 4 | Eif4h | 730.3333333 | 3 | 0.63417358 | 140490 | 0.60981048 |
| 296 | 2.51818762 | 1.47E-05 | 0.397111 | 3 | 4 | Eif2b3 | 640.3333333 | 3 | 0.6204531 | 147796 | 0.55449552 |
| 297 | 2.7070836 | 1.13E-05 | 0.36940123 | 3 | 4 | Ehf | 462.6666667 | 3 | 0.5732291 | 77658 | 0.45936982 |
| 298 | 2.71474154 | 1.12E-05 | 0.36835919 | 3 | 4 | Efnb2 | 457.6666667 | 3 | 0.57131461 | 76364 | 0.45712379 |
| 299 | 2.46330568 | 1.38E-05 | 0.40595855 | 3 | 4 | Eef2k | 730.3333333 | 3 | 0.63417358 | 140490 | 0.60981048 |
| 300 | 2.43395022 | 1.49E-05 | 0.41085475 | 3 | 4 | Eda2r | 764 | 3 | 0.64151244 | 151398 | 0.62592289 |
| 301 | 2.45820038 | 1.51E-05 | 0.40680166 | 3 | 4 | E130003G02Rik | 712 | 3 | 0.6354499 | 151864 | 0.5925 |
| 302 | 2.45309509 | 1.47E-05 | 0.40764828 | 3 | 4 | Dyrk1a | 717 | 3 | 0.63672623 | 151950 | 0.59468439 |
| 303 | 2.56285897 | 1.39E-05 | 0.39018924 | 3 | 4 | Dusp7 | 606.6666667 | 3 | 0.60928526 | 138682 | 0.5417412 |
| 304 | 2.45309509 | 1.47E-05 | 0.40764828 | 3 | 4 | Drp2 | 717 | 3 | 0.63672623 | 151950 | 0.59468439 |
| 305 | 2.55647735 | 1.41E-05 | 0.39116326 | 3 | 4 | Draxin | 593.3333333 | 3 | 0.61088066 | 143016 | 0.52745622 |
| 306 | 2.46330568 | 1.38E-05 | 0.40595855 | 3 | 4 | Dpf3 | 730.3333333 | 3 | 0.63417358 | 140490 | 0.60981048 |
| 307 | 2.48245054 | 1.44E-05 | 0.40282776 | 3 | 4 | Dock3 | 665 | 3 | 0.62938736 | 145600 | 0.56223539 |
| 308 | 2.44033184 | 1.59E-05 | 0.40978033 | 3 | 4 | Dnali1 | 698.6666667 | 3 | 0.63991704 | 162114 | 0.57468424 |
| 309 | 2.55647735 | 1.41E-05 | 0.39116326 | 3 | 4 | Dnajb5 | 593.3333333 | 3 | 0.61088066 | 143016 | 0.52745622 |
| 310 | 2.54499043 | 1.30E-05 | 0.39292879 | 3 | 4 | Dlgap1 | 568 | 3 | 0.61375239 | 88604 | 0.50088339 |
| 311 | 2.45820038 | 1.51E-05 | 0.40680166 | 3 | 4 | Dlg5 | 712 | 3 | 0.6354499 | 151864 | 0.5925 |
| 312 | 2.45820038 | 1.51E-05 | 0.40680166 | 3 | 4 | Diras1 | 712 | 3 | 0.6354499 | 151864 | 0.5925 |
| 313 | 2.52074027 | 1.41E-05 | 0.39670886 | 3 | 4 | Dgcr14 | 658.6666667 | 3 | 0.61981493 | 142934 | 0.5713872 |
| 314 | 2.43395022 | 1.49E-05 | 0.41085475 | 3 | 4 | Depdc5 | 764 | 3 | 0.64151244 | 151398 | 0.62592289 |
| 315 | 2.43395022 | 1.49E-05 | 0.41085475 | 3 | 4 | Dennd2c | 764 | 3 | 0.64151244 | 151398 | 0.62592289 |
| 316 | 2.45309509 | 1.47E-05 | 0.40764828 | 3 | 4 | Ddx21 | 717 | 3 | 0.63672623 | 151950 | 0.59468439 |
| 317 | 2.45309509 | 1.47E-05 | 0.40764828 | 3 | 4 | Ddx19b | 717 | 3 | 0.63672623 | 151950 | 0.59468439 |
| 318 | 2.45309509 | 1.47E-05 | 0.40764828 | 3 | 4 | Dcun1d4 | 717 | 3 | 0.63672623 | 151950 | 0.59468439 |
| 319 | 2.73388641 | 1.10E-05 | 0.36577965 | 3 | 4 | Dcaf8 | 444.3333333 | 3 | 0.5665284 | 77434 | 0.45054201 |
| 320 | 2.52074027 | 1.41E-05 | 0.39670886 | 3 | 4 | Dbt | 658.6666667 | 3 | 0.61981493 | 142934 | 0.5713872 |
| 321 | 2.43395022 | 1.49E-05 | 0.41085475 | 3 | 4 | Dapk1 | 764 | 3 | 0.64151244 | 151398 | 0.62592289 |
| 322 | 2.46330568 | 1.38E-05 | 0.40595855 | 3 | 4 | D5Ertd579e | 730.3333333 | 3 | 0.63417358 | 140490 | 0.60981048 |
| 323 | 2.46330568 | 1.38E-05 | 0.40595855 | 3 | 4 | D430019H16Rik | 730.3333333 | 3 | 0.63417358 | 140490 | 0.60981048 |
| 324 | 2.44033184 | 1.59E-05 | 0.40978033 | 3 | 4 | D2hgdh | 698.6666667 | 3 | 0.63991704 | 162114 | 0.57468424 |
| 325 | 2.51818762 | 1.47E-05 | 0.397111 | 3 | 4 | Cytip | 640.3333333 | 3 | 0.6204531 | 147796 | 0.55449552 |
| 326 | 2.46330568 | 1.38E-05 | 0.40595855 | 3 | 4 | Cyth4 | 730.3333333 | 3 | 0.63417358 | 140490 | 0.60981048 |
| 327 | 2.56285897 | 1.39E-05 | 0.39018924 | 3 | 4 | Cyp51 | 606.6666667 | 3 | 0.60928526 | 138682 | 0.5417412 |
| 328 | 2.46330568 | 1.38E-05 | 0.40595855 | 3 | 4 | Cyld | 730.3333333 | 3 | 0.63417358 | 140490 | 0.60981048 |
| 329 | 2.52074027 | 1.41E-05 | 0.39670886 | 3 | 4 | Cyb5b | 658.6666667 | 3 | 0.61981493 | 142934 | 0.5713872 |
| 330 | 2.44033184 | 1.59E-05 | 0.40978033 | 3 | 4 | Cxxc4 | 698.6666667 | 3 | 0.63991704 | 162114 | 0.57468424 |
| 331 | 2.46330568 | 1.38E-05 | 0.40595855 | 3 | 4 | Cxadr | 730.3333333 | 3 | 0.63417358 | 140490 | 0.60981048 |
| 332 | 2.65220166 | 1.16E-05 | 0.37704524 | 3 | 4 | Cuedc2 | 509.6666667 | 3 | 0.58694959 | 77434 | 0.48536896 |
| 333 | 2.48245054 | 1.44E-05 | 0.40282776 | 3 | 4 | Ctsd | 665 | 3 | 0.62938736 | 145600 | 0.56223539 |
| 334 | 2.45820038 | 1.51E-05 | 0.40680166 | 3 | 4 | Ctns | 712 | 3 | 0.6354499 | 151864 | 0.5925 |
| 335 | 2.43395022 | 1.49E-05 | 0.41085475 | 3 | 4 | Csrnp3 | 764 | 3 | 0.64151244 | 151398 | 0.62592289 |
| 336 | 2.68410976 | 1.14E-05 | 0.37256301 | 3 | 4 | Cspg4 | 476 | 3 | 0.57897256 | 75378 | 0.46432063 |
| 337 | 2.48245054 | 1.44E-05 | 0.40282776 | 3 | 4 | Csk | 665 | 3 | 0.62938736 | 145600 | 0.56223539 |
| 338 | 2.52074027 | 1.41E-05 | 0.39670886 | 3 | 4 | Csf3 | 658.6666667 | 3 | 0.61981493 | 142934 | 0.5713872 |
| 339 | 2.45820038 | 1.51E-05 | 0.40680166 | 3 | 4 | Crxos1 | 712 | 3 | 0.6354499 | 151864 | 0.5925 |
| 340 | 2.46330568 | 1.38E-05 | 0.40595855 | 3 | 4 | Crx | 730.3333333 | 3 | 0.63417358 | 140490 | 0.60981048 |
| 341 | 2.46330568 | 1.38E-05 | 0.40595855 | 3 | 4 | Crk | 730.3333333 | 3 | 0.63417358 | 140490 | 0.60981048 |
| 342 | 2.45309509 | 1.47E-05 | 0.40764828 | 3 | 4 | Creg2 | 717 | 3 | 0.63672623 | 151950 | 0.59468439 |
| 343 | 2.44033184 | 1.59E-05 | 0.40978033 | 3 | 4 | Crebrf | 698.6666667 | 3 | 0.63991704 | 162114 | 0.57468424 |
| 344 | 2.46330568 | 1.38E-05 | 0.40595855 | 3 | 4 | Creb1 | 730.3333333 | 3 | 0.63417358 | 140490 | 0.60981048 |
| 345 | 2.45309509 | 1.47E-05 | 0.40764828 | 3 | 4 | Cradd | 717 | 3 | 0.63672623 | 151950 | 0.59468439 |
| 346 | 2.45309509 | 1.47E-05 | 0.40764828 | 3 | 4 | Cpox | 717 | 3 | 0.63672623 | 151950 | 0.59468439 |
| 347 | 2.43395022 | 1.49E-05 | 0.41085475 | 3 | 4 | Cplx3 | 764 | 3 | 0.64151244 | 151398 | 0.62592289 |
| 348 | 2.46330568 | 1.38E-05 | 0.40595855 | 3 | 4 | Cpeb3 | 730.3333333 | 3 | 0.63417358 | 140490 | 0.60981048 |
| 349 | 2.52074027 | 1.41E-05 | 0.39670886 | 3 | 4 | Cox15 | 658.6666667 | 3 | 0.61981493 | 142934 | 0.5713872 |
| 350 | 2.57817486 | 1.24E-05 | 0.38787129 | 3 | 4 | Coro2a | 516 | 3 | 0.60545629 | 85436 | 0.46564195 |
| 351 | 2.45820038 | 1.51E-05 | 0.40680166 | 3 | 4 | Col26a1 | 712 | 3 | 0.6354499 | 151864 | 0.5925 |
| 352 | 2.43395022 | 1.49E-05 | 0.41085475 | 3 | 4 | Cnih4 | 764 | 3 | 0.64151244 | 151398 | 0.62592289 |
| 353 | 2.73388641 | 1.10E-05 | 0.36577965 | 3 | 4 | Cngb1 | 444.3333333 | 3 | 0.5665284 | 77434 | 0.45054201 |
| 354 | 2.52074027 | 1.41E-05 | 0.39670886 | 3 | 4 | Cmpk2 | 658.6666667 | 3 | 0.61981493 | 142934 | 0.5713872 |
| 355 | 2.45309509 | 1.47E-05 | 0.40764828 | 3 | 4 | Clec12a | 717 | 3 | 0.63672623 | 151950 | 0.59468439 |
| 356 | 2.55775367 | 1.25E-05 | 0.39096806 | 3 | 4 | Cldn4 | 581.3333333 | 3 | 0.61056158 | 80408 | 0.51723113 |
| 357 | 2.71474154 | 1.12E-05 | 0.36835919 | 3 | 4 | Chst3 | 457.6666667 | 3 | 0.57131461 | 76364 | 0.45712379 |
| 358 | 2.44033184 | 1.59E-05 | 0.40978033 | 3 | 4 | Chn1 | 698.6666667 | 3 | 0.63991704 | 162114 | 0.57468424 |
| 359 | 2.48245054 | 1.44E-05 | 0.40282776 | 3 | 4 | Chchd5 | 665 | 3 | 0.62938736 | 145600 | 0.56223539 |
| 360 | 2.48245054 | 1.44E-05 | 0.40282776 | 3 | 4 | Cflar | 665 | 3 | 0.62938736 | 145600 | 0.56223539 |
| 361 | 2.43395022 | 1.49E-05 | 0.41085475 | 3 | 4 | Cep72 | 764 | 3 | 0.64151244 | 151398 | 0.62592289 |
| 362 | 2.45309509 | 1.47E-05 | 0.40764828 | 3 | 4 | Cep68 | 717 | 3 | 0.63672623 | 151950 | 0.59468439 |
| 363 | 2.48245054 | 1.44E-05 | 0.40282776 | 3 | 4 | Cep170b | 665 | 3 | 0.62938736 | 145600 | 0.56223539 |
| 364 | 2.44033184 | 1.59E-05 | 0.40978033 | 3 | 4 | Cenpf | 698.6666667 | 3 | 0.63991704 | 162114 | 0.57468424 |
| 365 | 2.48245054 | 1.44E-05 | 0.40282776 | 3 | 4 | Celf5 | 665 | 3 | 0.62938736 | 145600 | 0.56223539 |
| 366 | 2.44033184 | 1.59E-05 | 0.40978033 | 3 | 4 | Celf2 | 698.6666667 | 3 | 0.63991704 | 162114 | 0.57468424 |
| 367 | 2.45309509 | 1.47E-05 | 0.40764828 | 3 | 4 | Cecr6 | 717 | 3 | 0.63672623 | 151950 | 0.59468439 |
| 368 | 2.45820038 | 1.51E-05 | 0.40680166 | 3 | 4 | Cecr2 | 712 | 3 | 0.6354499 | 151864 | 0.5925 |
| 369 | 2.46330568 | 1.38E-05 | 0.40595855 | 3 | 4 | Cdv3 | 730.3333333 | 3 | 0.63417358 | 140490 | 0.60981048 |
| 370 | 2.45820038 | 1.51E-05 | 0.40680166 | 3 | 4 | Cdk5r2 | 712 | 3 | 0.6354499 | 151864 | 0.5925 |
| 371 | 2.51818762 | 1.47E-05 | 0.397111 | 3 | 4 | Cdk2 | 640.3333333 | 3 | 0.6204531 | 147796 | 0.55449552 |
| 372 | 2.52074027 | 1.41E-05 | 0.39670886 | 3 | 4 | Cdk18 | 658.6666667 | 3 | 0.61981493 | 142934 | 0.5713872 |
| 373 | 2.55647735 | 1.41E-05 | 0.39116326 | 3 | 4 | Cdipt | 593.3333333 | 3 | 0.61088066 | 143016 | 0.52745622 |
| 374 | 2.45309509 | 1.47E-05 | 0.40764828 | 3 | 4 | Cdca4 | 717 | 3 | 0.63672623 | 151950 | 0.59468439 |
| 375 | 2.43395022 | 1.49E-05 | 0.41085475 | 3 | 4 | Cdc37l1 | 764 | 3 | 0.64151244 | 151398 | 0.62592289 |
| 376 | 2.52074027 | 1.41E-05 | 0.39670886 | 3 | 4 | Cdc37 | 658.6666667 | 3 | 0.61981493 | 142934 | 0.5713872 |
| 377 | 2.43395022 | 1.49E-05 | 0.41085475 | 3 | 4 | Cdc23 | 764 | 3 | 0.64151244 | 151398 | 0.62592289 |
| 378 | 2.48245054 | 1.44E-05 | 0.40282776 | 3 | 4 | Cdadc1 | 665 | 3 | 0.62938736 | 145600 | 0.56223539 |
| 379 | 2.45309509 | 1.47E-05 | 0.40764828 | 3 | 4 | Cd2bp2 | 717 | 3 | 0.63672623 | 151950 | 0.59468439 |
| 380 | 2.43395022 | 1.49E-05 | 0.41085475 | 3 | 4 | Ccdc85b | 764 | 3 | 0.64151244 | 151398 | 0.62592289 |
| 381 | 2.43395022 | 1.49E-05 | 0.41085475 | 3 | 4 | Ccdc85a | 764 | 3 | 0.64151244 | 151398 | 0.62592289 |
| 382 | 2.55775367 | 1.25E-05 | 0.39096806 | 3 | 4 | Ccdc6 | 581.3333333 | 3 | 0.61056158 | 80408 | 0.51723113 |
| 383 | 2.55647735 | 1.41E-05 | 0.39116326 | 3 | 4 | Ccdc177 | 593.3333333 | 3 | 0.61088066 | 143016 | 0.52745622 |
| 384 | 2.48245054 | 1.44E-05 | 0.40282776 | 3 | 4 | Ccdc167 | 665 | 3 | 0.62938736 | 145600 | 0.56223539 |
| 385 | 2.48245054 | 1.44E-05 | 0.40282776 | 3 | 4 | Cbx7 | 665 | 3 | 0.62938736 | 145600 | 0.56223539 |
| 386 | 2.45820038 | 1.51E-05 | 0.40680166 | 3 | 4 | Cbx2 | 712 | 3 | 0.6354499 | 151864 | 0.5925 |
| 387 | 2.43395022 | 1.49E-05 | 0.41085475 | 3 | 4 | Cblb | 764 | 3 | 0.64151244 | 151398 | 0.62592289 |
| 388 | 2.45309509 | 1.47E-05 | 0.40764828 | 3 | 4 | Casp2 | 717 | 3 | 0.63672623 | 151950 | 0.59468439 |
| 389 | 2.56285897 | 1.39E-05 | 0.39018924 | 3 | 4 | Caskin1 | 606.6666667 | 3 | 0.60928526 | 138682 | 0.5417412 |
| 390 | 2.46330568 | 1.38E-05 | 0.40595855 | 3 | 4 | Car5b | 730.3333333 | 3 | 0.63417358 | 140490 | 0.60981048 |
| 391 | 2.45820038 | 1.51E-05 | 0.40680166 | 3 | 4 | Car10 | 712 | 3 | 0.6354499 | 151864 | 0.5925 |
| 392 | 2.51818762 | 1.47E-05 | 0.397111 | 3 | 4 | Capn13 | 640.3333333 | 3 | 0.6204531 | 147796 | 0.55449552 |
| 393 | 2.58966177 | 1.20E-05 | 0.38615081 | 3 | 4 | Calm1 | 534.3333333 | 3 | 0.60258456 | 79148 | 0.48617442 |
| 394 | 2.48245054 | 1.44E-05 | 0.40282776 | 3 | 4 | Calcoco2 | 665 | 3 | 0.62938736 | 145600 | 0.56223539 |
| 395 | 2.52074027 | 1.41E-05 | 0.39670886 | 3 | 4 | Cacul1 | 658.6666667 | 3 | 0.61981493 | 142934 | 0.5713872 |
| 396 | 2.48245054 | 1.44E-05 | 0.40282776 | 3 | 4 | Cacna2d2 | 665 | 3 | 0.62938736 | 145600 | 0.56223539 |
| 397 | 2.45309509 | 1.47E-05 | 0.40764828 | 3 | 4 | Cacna1d | 717 | 3 | 0.63672623 | 151950 | 0.59468439 |
| 398 | 2.43395022 | 1.49E-05 | 0.41085475 | 3 | 4 | C77370 | 764 | 3 | 0.64151244 | 151398 | 0.62592289 |
| 399 | 2.48245054 | 1.44E-05 | 0.40282776 | 3 | 4 | Bysl | 665 | 3 | 0.62938736 | 145600 | 0.56223539 |
| 400 | 2.46330568 | 1.38E-05 | 0.40595855 | 3 | 4 | Bud13 | 730.3333333 | 3 | 0.63417358 | 140490 | 0.60981048 |
| 401 | 2.45820038 | 1.51E-05 | 0.40680166 | 3 | 4 | Btla | 712 | 3 | 0.6354499 | 151864 | 0.5925 |
| 402 | 2.46330568 | 1.38E-05 | 0.40595855 | 3 | 4 | Brat1 | 730.3333333 | 3 | 0.63417358 | 140490 | 0.60981048 |
| 403 | 2.56285897 | 1.39E-05 | 0.39018924 | 3 | 4 | Bmp8b | 606.6666667 | 3 | 0.60928526 | 138682 | 0.5417412 |
| 404 | 2.51818762 | 1.47E-05 | 0.397111 | 3 | 4 | Bmf | 640.3333333 | 3 | 0.6204531 | 147796 | 0.55449552 |
| 405 | 2.43395022 | 1.49E-05 | 0.41085475 | 3 | 4 | Bloc1s6 | 764 | 3 | 0.64151244 | 151398 | 0.62592289 |
| 406 | 2.43395022 | 1.49E-05 | 0.41085475 | 3 | 4 | Bid | 764 | 3 | 0.64151244 | 151398 | 0.62592289 |
| 407 | 2.48245054 | 1.44E-05 | 0.40282776 | 3 | 4 | Bicd2 | 665 | 3 | 0.62938736 | 145600 | 0.56223539 |
| 408 | 2.45309509 | 1.47E-05 | 0.40764828 | 3 | 4 | Bend3 | 717 | 3 | 0.63672623 | 151950 | 0.59468439 |
| 409 | 2.45820038 | 1.51E-05 | 0.40680166 | 3 | 4 | Bdnf | 712 | 3 | 0.6354499 | 151864 | 0.5925 |
| 410 | 2.56285897 | 1.39E-05 | 0.39018924 | 3 | 4 | Bche | 606.6666667 | 3 | 0.60928526 | 138682 | 0.5417412 |
| 411 | 2.45309509 | 1.47E-05 | 0.40764828 | 3 | 4 | Bcat1 | 717 | 3 | 0.63672623 | 151950 | 0.59468439 |
| 412 | 2.791321 | 0 | 0.35825332 | 3 | 4 | BC051628 | 940 | 3 | 0.55216975 | 6 | 1 |
| 413 | 2.55775367 | 1.25E-05 | 0.39096806 | 3 | 4 | BC021891 | 581.3333333 | 3 | 0.61056158 | 80408 | 0.51723113 |
| 414 | 2.43395022 | 1.49E-05 | 0.41085475 | 3 | 4 | Bbx | 764 | 3 | 0.64151244 | 151398 | 0.62592289 |
| 415 | 2.45820038 | 1.51E-05 | 0.40680166 | 3 | 4 | Bbs1 | 712 | 3 | 0.6354499 | 151864 | 0.5925 |
| 416 | 2.65220166 | 1.16E-05 | 0.37704524 | 3 | 4 | Banp | 509.6666667 | 3 | 0.58694959 | 77434 | 0.48536896 |
| 417 | 2.48245054 | 1.44E-05 | 0.40282776 | 3 | 4 | Baiap3 | 665 | 3 | 0.62938736 | 145600 | 0.56223539 |
| 418 | 2.56285897 | 1.39E-05 | 0.39018924 | 3 | 4 | Bahd1 | 606.6666667 | 3 | 0.60928526 | 138682 | 0.5417412 |
| 419 | 2.43395022 | 1.49E-05 | 0.41085475 | 3 | 4 | B630005N14Rik | 764 | 3 | 0.64151244 | 151398 | 0.62592289 |
| 420 | 2.45820038 | 1.51E-05 | 0.40680166 | 3 | 4 | B430306N03Rik | 712 | 3 | 0.6354499 | 151864 | 0.5925 |
| 421 | 2.46330568 | 1.38E-05 | 0.40595855 | 3 | 4 | B230219D22Rik | 730.3333333 | 3 | 0.63417358 | 140490 | 0.60981048 |
| 422 | 2.43395022 | 1.49E-05 | 0.41085475 | 3 | 4 | Atxn7l3b | 764 | 3 | 0.64151244 | 151398 | 0.62592289 |
| 423 | 2.55775367 | 1.25E-05 | 0.39096806 | 3 | 4 | Atxn3 | 581.3333333 | 3 | 0.61056158 | 80408 | 0.51723113 |
| 424 | 2.43395022 | 1.49E-05 | 0.41085475 | 3 | 4 | Atxn1l | 764 | 3 | 0.64151244 | 151398 | 0.62592289 |
| 425 | 2.46330568 | 1.38E-05 | 0.40595855 | 3 | 4 | Atrnl1 | 730.3333333 | 3 | 0.63417358 | 140490 | 0.60981048 |
| 426 | 2.43395022 | 1.49E-05 | 0.41085475 | 3 | 4 | Atrn | 764 | 3 | 0.64151244 | 151398 | 0.62592289 |
| 427 | 2.48245054 | 1.44E-05 | 0.40282776 | 3 | 4 | Atp9b | 665 | 3 | 0.62938736 | 145600 | 0.56223539 |
| 428 | 2.45820038 | 1.51E-05 | 0.40680166 | 3 | 4 | Atp6v1b2 | 712 | 3 | 0.6354499 | 151864 | 0.5925 |
| 429 | 2.45820038 | 1.51E-05 | 0.40680166 | 3 | 4 | Atp6v0a1 | 712 | 3 | 0.6354499 | 151864 | 0.5925 |
| 430 | 2.51818762 | 1.47E-05 | 0.397111 | 3 | 4 | Atp11a | 640.3333333 | 3 | 0.6204531 | 147796 | 0.55449552 |
| 431 | 2.46330568 | 1.38E-05 | 0.40595855 | 3 | 4 | Atl3 | 730.3333333 | 3 | 0.63417358 | 140490 | 0.60981048 |
| 432 | 2.45820038 | 1.51E-05 | 0.40680166 | 3 | 4 | Atg7 | 712 | 3 | 0.6354499 | 151864 | 0.5925 |
| 433 | 2.55647735 | 1.41E-05 | 0.39116326 | 3 | 4 | Atg4b | 593.3333333 | 3 | 0.61088066 | 143016 | 0.52745622 |
| 434 | 2.43395022 | 1.49E-05 | 0.41085475 | 3 | 4 | Asxl3 | 764 | 3 | 0.64151244 | 151398 | 0.62592289 |
| 435 | 2.48245054 | 1.44E-05 | 0.40282776 | 3 | 4 | Asb7 | 665 | 3 | 0.62938736 | 145600 | 0.56223539 |
| 436 | 2.46330568 | 1.38E-05 | 0.40595855 | 3 | 4 | Asap3 | 730.3333333 | 3 | 0.63417358 | 140490 | 0.60981048 |
| 437 | 2.48245054 | 1.44E-05 | 0.40282776 | 3 | 4 | Arnt2 | 665 | 3 | 0.62938736 | 145600 | 0.56223539 |
| 438 | 2.45309509 | 1.47E-05 | 0.40764828 | 3 | 4 | Armc9 | 717 | 3 | 0.63672623 | 151950 | 0.59468439 |
| 439 | 2.65220166 | 1.16E-05 | 0.37704524 | 3 | 4 | Arid4a | 509.6666667 | 3 | 0.58694959 | 77434 | 0.48536896 |
| 440 | 2.43395022 | 1.49E-05 | 0.41085475 | 3 | 4 | Arhgef7 | 764 | 3 | 0.64151244 | 151398 | 0.62592289 |
| 441 | 2.45309509 | 1.47E-05 | 0.40764828 | 3 | 4 | Arhgef3 | 717 | 3 | 0.63672623 | 151950 | 0.59468439 |
| 442 | 2.45820038 | 1.51E-05 | 0.40680166 | 3 | 4 | Arhgef12 | 712 | 3 | 0.6354499 | 151864 | 0.5925 |
| 443 | 2.46330568 | 1.38E-05 | 0.40595855 | 3 | 4 | Arhgap26 | 730.3333333 | 3 | 0.63417358 | 140490 | 0.60981048 |
| 444 | 2.46330568 | 1.38E-05 | 0.40595855 | 3 | 4 | Arhgap19 | 730.3333333 | 3 | 0.63417358 | 140490 | 0.60981048 |
| 445 | 2.43395022 | 1.49E-05 | 0.41085475 | 3 | 4 | Arfip2 | 764 | 3 | 0.64151244 | 151398 | 0.62592289 |
| 446 | 2.43395022 | 1.49E-05 | 0.41085475 | 3 | 4 | Aqp6 | 764 | 3 | 0.64151244 | 151398 | 0.62592289 |
| 447 | 2.56285897 | 1.39E-05 | 0.39018924 | 3 | 4 | Aqp1 | 606.6666667 | 3 | 0.60928526 | 138682 | 0.5417412 |
| 448 | 2.44033184 | 1.59E-05 | 0.40978033 | 3 | 4 | Apba1 | 698.6666667 | 3 | 0.63991704 | 162114 | 0.57468424 |
| 449 | 2.46330568 | 1.38E-05 | 0.40595855 | 3 | 4 | Ap4e1 | 730.3333333 | 3 | 0.63417358 | 140490 | 0.60981048 |
| 450 | 2.43395022 | 1.49E-05 | 0.41085475 | 3 | 4 | Antxr1 | 764 | 3 | 0.64151244 | 151398 | 0.62592289 |
| 451 | 2.43395022 | 1.49E-05 | 0.41085475 | 3 | 4 | Ano6 | 764 | 3 | 0.64151244 | 151398 | 0.62592289 |
| 452 | 2.46330568 | 1.38E-05 | 0.40595855 | 3 | 4 | Anln | 730.3333333 | 3 | 0.63417358 | 140490 | 0.60981048 |
| 453 | 2.45820038 | 1.51E-05 | 0.40680166 | 3 | 4 | Ankrd52 | 712 | 3 | 0.6354499 | 151864 | 0.5925 |
| 454 | 2.52074027 | 1.41E-05 | 0.39670886 | 3 | 4 | Ankrd50 | 658.6666667 | 3 | 0.61981493 | 142934 | 0.5713872 |
| 455 | 2.43395022 | 1.49E-05 | 0.41085475 | 3 | 4 | Ankrd40 | 764 | 3 | 0.64151244 | 151398 | 0.62592289 |
| 456 | 2.44033184 | 1.59E-05 | 0.40978033 | 3 | 4 | Ankrd33b | 698.6666667 | 3 | 0.63991704 | 162114 | 0.57468424 |
| 457 | 2.45309509 | 1.47E-05 | 0.40764828 | 3 | 4 | Ank2 | 717 | 3 | 0.63672623 | 151950 | 0.59468439 |
| 458 | 2.43395022 | 1.49E-05 | 0.41085475 | 3 | 4 | Angptl4 | 764 | 3 | 0.64151244 | 151398 | 0.62592289 |
| 459 | 2.56285897 | 1.39E-05 | 0.39018924 | 3 | 4 | Angptl2 | 606.6666667 | 3 | 0.60928526 | 138682 | 0.5417412 |
| 460 | 2.43395022 | 1.49E-05 | 0.41085475 | 3 | 4 | Anapc1 | 764 | 3 | 0.64151244 | 151398 | 0.62592289 |
| 461 | 2.45309509 | 1.47E-05 | 0.40764828 | 3 | 4 | Amz1 | 717 | 3 | 0.63672623 | 151950 | 0.59468439 |
| 462 | 2.48245054 | 1.44E-05 | 0.40282776 | 3 | 4 | Amotl1 | 665 | 3 | 0.62938736 | 145600 | 0.56223539 |
| 463 | 2.43395022 | 1.49E-05 | 0.41085475 | 3 | 4 | Ammecr1l | 764 | 3 | 0.64151244 | 151398 | 0.62592289 |
| 464 | 2.48245054 | 1.44E-05 | 0.40282776 | 3 | 4 | Amigo1 | 665 | 3 | 0.62938736 | 145600 | 0.56223539 |
| 465 | 2.55647735 | 1.41E-05 | 0.39116326 | 3 | 4 | Alpl | 593.3333333 | 3 | 0.61088066 | 143016 | 0.52745622 |
| 466 | 2.48245054 | 1.44E-05 | 0.40282776 | 3 | 4 | Alkbh5 | 665 | 3 | 0.62938736 | 145600 | 0.56223539 |
| 467 | 2.52074027 | 1.41E-05 | 0.39670886 | 3 | 4 | Alg11 | 658.6666667 | 3 | 0.61981493 | 142934 | 0.5713872 |
| 468 | 2.68410976 | 1.14E-05 | 0.37256301 | 3 | 4 | Aldh3a2 | 476 | 3 | 0.57897256 | 75378 | 0.46432063 |
| 469 | 2.44033184 | 1.59E-05 | 0.40978033 | 3 | 4 | Akt3 | 698.6666667 | 3 | 0.63991704 | 162114 | 0.57468424 |
| 470 | 2.46330568 | 1.38E-05 | 0.40595855 | 3 | 4 | Akr1d1 | 730.3333333 | 3 | 0.63417358 | 140490 | 0.60981048 |
| 471 | 2.45820038 | 1.51E-05 | 0.40680166 | 3 | 4 | Aifm2 | 712 | 3 | 0.6354499 | 151864 | 0.5925 |
| 472 | 2.54499043 | 1.30E-05 | 0.39292879 | 3 | 4 | AI597479 | 568 | 3 | 0.61375239 | 88604 | 0.50088339 |
| 473 | 2.43395022 | 1.49E-05 | 0.41085475 | 3 | 4 | AI593442 | 764 | 3 | 0.64151244 | 151398 | 0.62592289 |
| 474 | 2.55647735 | 1.41E-05 | 0.39116326 | 3 | 4 | Ahrr | 593.3333333 | 3 | 0.61088066 | 143016 | 0.52745622 |
| 475 | 2.51818762 | 1.47E-05 | 0.397111 | 3 | 4 | Agps | 640.3333333 | 3 | 0.6204531 | 147796 | 0.55449552 |
| 476 | 2.43395022 | 1.49E-05 | 0.41085475 | 3 | 4 | Aebp2 | 764 | 3 | 0.64151244 | 151398 | 0.62592289 |
| 477 | 2.45309509 | 1.47E-05 | 0.40764828 | 3 | 4 | Adra1a | 717 | 3 | 0.63672623 | 151950 | 0.59468439 |
| 478 | 2.45820038 | 1.51E-05 | 0.40680166 | 3 | 4 | Adora1 | 712 | 3 | 0.6354499 | 151864 | 0.5925 |
| 479 | 2.51818762 | 1.47E-05 | 0.397111 | 3 | 4 | Adipor2 | 640.3333333 | 3 | 0.6204531 | 147796 | 0.55449552 |
| 480 | 2.58966177 | 1.20E-05 | 0.38615081 | 3 | 4 | Adck3 | 534.3333333 | 3 | 0.60258456 | 79148 | 0.48617442 |
| 481 | 2.48245054 | 1.44E-05 | 0.40282776 | 3 | 4 | Adar | 665 | 3 | 0.62938736 | 145600 | 0.56223539 |
| 482 | 2.65220166 | 1.16E-05 | 0.37704524 | 3 | 4 | Adamts2 | 509.6666667 | 3 | 0.58694959 | 77434 | 0.48536896 |
| 483 | 2.43395022 | 1.49E-05 | 0.41085475 | 3 | 4 | Acvr1c | 764 | 3 | 0.64151244 | 151398 | 0.62592289 |
| 484 | 2.45820038 | 1.51E-05 | 0.40680166 | 3 | 4 | Acvr1b | 712 | 3 | 0.6354499 | 151864 | 0.5925 |
| 485 | 2.43395022 | 1.49E-05 | 0.41085475 | 3 | 4 | Actr10 | 764 | 3 | 0.64151244 | 151398 | 0.62592289 |
| 486 | 2.7070836 | 1.13E-05 | 0.36940123 | 3 | 4 | Acp2 | 462.6666667 | 3 | 0.5732291 | 77658 | 0.45936982 |
| 487 | 2.43395022 | 1.49E-05 | 0.41085475 | 3 | 4 | Acadsb | 764 | 3 | 0.64151244 | 151398 | 0.62592289 |
| 488 | 2.45309509 | 1.47E-05 | 0.40764828 | 3 | 4 | Acads | 717 | 3 | 0.63672623 | 151950 | 0.59468439 |
| 489 | 2.55647735 | 1.41E-05 | 0.39116326 | 3 | 4 | Acacb | 593.3333333 | 3 | 0.61088066 | 143016 | 0.52745622 |
| 490 | 2.43395022 | 1.49E-05 | 0.41085475 | 3 | 4 | Abi2 | 764 | 3 | 0.64151244 | 151398 | 0.62592289 |
| 491 | 2.57817486 | 1.24E-05 | 0.38787129 | 3 | 4 | Abcc4 | 516 | 3 | 0.60545629 | 85436 | 0.46564195 |
| 492 | 2.46330568 | 1.38E-05 | 0.40595855 | 3 | 4 | Abca1 | 730.3333333 | 3 | 0.63417358 | 140490 | 0.60981048 |
| 493 | 2.45309509 | 1.47E-05 | 0.40764828 | 3 | 4 | Aar2 | 717 | 3 | 0.63672623 | 151950 | 0.59468439 |
| 494 | 2.44033184 | 1.59E-05 | 0.40978033 | 3 | 4 | A630081J09Rik | 698.6666667 | 3 | 0.63991704 | 162114 | 0.57468424 |
| 495 | 2.43395022 | 1.49E-05 | 0.41085475 | 3 | 4 | 9430020K01Rik | 764 | 3 | 0.64151244 | 151398 | 0.62592289 |
| 496 | 2.44033184 | 1.59E-05 | 0.40978033 | 3 | 4 | 6030458C11Rik | 698.6666667 | 3 | 0.63991704 | 162114 | 0.57468424 |
| 497 | 2.46330568 | 1.38E-05 | 0.40595855 | 3 | 4 | 4933426M11Rik | 730.3333333 | 3 | 0.63417358 | 140490 | 0.60981048 |
| 498 | 2.46330568 | 1.38E-05 | 0.40595855 | 3 | 4 | 4931406P16Rik | 730.3333333 | 3 | 0.63417358 | 140490 | 0.60981048 |
| 499 | 2.45820038 | 1.51E-05 | 0.40680166 | 3 | 4 | 4931406B18Rik | 712 | 3 | 0.6354499 | 151864 | 0.5925 |
| 500 | 2.52074027 | 1.41E-05 | 0.39670886 | 3 | 4 | 4930427A07Rik | 658.6666667 | 3 | 0.61981493 | 142934 | 0.5713872 |
| 501 | 2.51818762 | 1.47E-05 | 0.397111 | 3 | 4 | 3110035E14Rik | 640.3333333 | 3 | 0.6204531 | 147796 | 0.55449552 |
| 502 | 2.44033184 | 1.59E-05 | 0.40978033 | 3 | 4 | 2810459M11Rik | 698.6666667 | 3 | 0.63991704 | 162114 | 0.57468424 |
| 503 | 2.48245054 | 1.44E-05 | 0.40282776 | 3 | 4 | 2700081O15Rik | 665 | 3 | 0.62938736 | 145600 | 0.56223539 |
| 504 | 2.43395022 | 1.49E-05 | 0.41085475 | 3 | 4 | 2700050L05Rik | 764 | 3 | 0.64151244 | 151398 | 0.62592289 |
| 505 | 2.43395022 | 1.49E-05 | 0.41085475 | 3 | 4 | 2510009E07Rik | 764 | 3 | 0.64151244 | 151398 | 0.62592289 |
| 506 | 2.46330568 | 1.38E-05 | 0.40595855 | 3 | 4 | 2410131K14Rik | 730.3333333 | 3 | 0.63417358 | 140490 | 0.60981048 |
| 507 | 2.51818762 | 1.47E-05 | 0.397111 | 3 | 4 | 2310022B05Rik | 640.3333333 | 3 | 0.6204531 | 147796 | 0.55449552 |
| 508 | 2.48245054 | 1.44E-05 | 0.40282776 | 3 | 4 | 2010107G12Rik | 665 | 3 | 0.62938736 | 145600 | 0.56223539 |
| 509 | 2.44033184 | 1.59E-05 | 0.40978033 | 3 | 4 | 1700066B19Rik | 698.6666667 | 3 | 0.63991704 | 162114 | 0.57468424 |
| 510 | 2.44033184 | 1.59E-05 | 0.40978033 | 3 | 4 | 1700017B05Rik | 698.6666667 | 3 | 0.63991704 | 162114 | 0.57468424 |
| 511 | 2.43395022 | 1.49E-05 | 0.41085475 | 3 | 4 | 1200014J11Rik | 764 | 3 | 0.64151244 | 151398 | 0.62592289 |
| 512 | 2.43395022 | 1.49E-05 | 0.41085475 | 3 | 4 | 1110059G10Rik | 764 | 3 | 0.64151244 | 151398 | 0.62592289 |
| 513 | 2.46330568 | 1.38E-05 | 0.40595855 | 3 | 4 | 0610030E20Rik | 730.3333333 | 3 | 0.63417358 | 140490 | 0.60981048 |
